# ePresentation

**DOI:** 10.1111/ene.70191

**Published:** 2025-06-21

## Saturday, June 21, 2025

## Ageing and dementia 1

## EPR‐001

### Blood pTau217 distinguishes amyloid‐positive from amyloid‐negative subjects across the Alzheimer's disease continuum

#### A. Antonioni ^1^; E. Raho^1^; F. Di Lorenzo^2^; L. Manzoli^3^; M. Flacco^4^; G. Koch^1^

##### ^1^Department of Neuroscience and Rehabilitation, University of Ferrara, Ferrara, Italy; ^2^Department of Behavioral and Clinical Neurology, Santa Lucia Foundation IRCCS, Rome, Italy; ^3^Department of Medical and Surgical Sciences, University of Bologna, Bologna, Italy; ^4^Department of Environmental and Prevention Sciences, University of Ferrara, Ferrara, Italy

**Background and aims:** Alzheimer's disease (AD) is the leading cause of dementia worldwide, and cost‐effective tools to detect amyloid pathology, particularly in its early stages, are urgently needed. Blood‐based Tau phosphorylated at threonine 217 (pTau217) seems promising, but its reliability as a proxy for cerebrospinal fluid (CSF) status and ability to identify patients within the AD spectrum remain unclear.

**Methods:** We performed a systematic review and meta‐analysis on the potential of blood pTau217 to differentiate amyloid‐positive (A+) and amyloid‐negative (A‐) subjects. We included original studies reporting quantitative data on pTau217 concentrations in both blood and CSF in the AD continuum. The single‐group meta‐analysis computed the pooled pTau217 levels in blood and in CSF, separately in the A+ and A‐ groups, while the head‐to‐head meta‐analysis compared the mean pTau217 concentrations in the A+ versus A‐ subjects, both in blood and CSF, stratifying by assessment method in both cases.

**Results:** Ten studies (819 A+; 1,055 A−) were included. The mean pTau217 levels resulted higher in CSF than in blood and, crucially, in A+ individuals than in A– ones, regardless of the laboratory method employed, including Meso Scale Discovery (MSD), Single Molecule Array for Protein Detection (Simoa), and immunoprecipitation with mass spectrometry. Most importantly, all these laboratory techniques reliably distinguished A+ from A– subjects, whether applied to CSF or blood samples.

**Conclusion:** Blood‐based pTau217 is a reliable marker of amyloid pathology and might be a non‐invasive, scalable biomarker for early AD detection, reducing the reliance on more invasive, expansive, and less accessible methods.

**Disclosure:** Nothing to disclose.

## EPR‐002

### Exome sequencing identifies a rare damaging variant in GRIN2C in familial late‐onset Alzheimer's disease

#### E. Rubino ^1^; M. Italia^2^; E. Giorgio^3^; S. Boschi^1^; P. Dimartino^3^; T. Pippucci^4^; F. Roveta^1^; C. Cambria^5^; G. Elia^1^; A. Marcinnò^1^; S. Gallone^1^; E. Rogaeva^6^; F. Antonucci^5^; A. Brusco^1^; F. Gardoni^2^; I. Rainero^1^

##### ^1^Department of Neuroscience “Rita Levi Montalcini”, University of Turin, Via Cherasco 15, Turin 10126, Italy; ^2^Department of Pharmacological and Biomolecular Sciences, University of Milan, Via Balzaretti 9, 20133 Milan, Italy; ^3^Department of Molecular Medicine, University of Pavia, Viia Forlanini 6, 27100 Pavia, Italy; ^4^Medical Genetics Unit, IRCCS Azienda Ospedaliero‐Universitaria, via Albertoni 15, 40138 Bologna, Italy; ^5^Department of Medical Biotechnology and Translational Medicine (BIOMETRA), University of Milan, Via Festa del Perdono 7, 20122 Milan, Italy; ^6^Tanz Centre for Research in Neurodegenerative Diseases, University of Toronto, King's College Circle 1, M5S1A8 Toronto, Ontario, Canada

**Background and aims:** Alzheimer's disease (AD) is a progressive neurodegenerative disorder influenced by both genetic and environmental factors. While early‐onset AD has well‐established genetic determinants, the genetic basis of late‐onset AD remains unclear. This study examined a large Italian family with late‐onset autosomal dominant AD, identifying a novel rare missense variant in GRIN2C gene.

**Methods:** Affected family members were screened for genetic variants in APP, PSEN1, and PSEN2, as well as 77 genes associated with neurodegenerative diseases using the NeuroX array assay. Exome sequencing was performed on three patients and two healthy relatives. Bioinformatics analyses were conducted. Functional studies were performed in primary neuronal cultures assessing the impact of the identified variant through immunocytochemistry and electrophysiology.

**Results:** No pathogenic variants were found in APP, PSEN1, PSEN2 or in genes screened using the NeuroX array. Exome sequencing revealed the c.3215C > T p.(A1072V) variant in GRIN2C gene (NM 000835.6), encoding the glutamate ionotropic N‐Methyl‐D‐aspartate receptor (NMDA) type subunit 2C (GluN2C). This variant segregated with AD in six affected members and was absent in nine healthy relatives. Primary rat hippocampal neurons overexpressing the variant showed increased NMDAR‐induced currents, indicating altered glutamatergic transmission. Surface expression assays revealed a higher surface/total ratio of mutant GluN2C, correlating with increased NMDAR current. Immunocytochemistry showed a reduced colocalization of mutant GluN2C with 14‐3‐3 proteins, suggesting impaired NMDAR trafficking.

**Conclusion:** This study identifies a rare missense variant in GRIN2C associated with late‐onset autosomal dominant AD. Our findings underscore the importance of GRIN2C‐containing NMDA receptors in glutamatergic signaling and their potential role in AD pathogenesis.

**Disclosure:** Nothing to disclose.

## EPR‐003

### Preliminary RCT insights from a 12‐week app‐based multidomain intervention in patients with cognitive decline

#### G. Nelles^1^; F. Bicu ^2^; V. Weil^2^; T. Steinmann^2^; D. Stein^2^; A. Quante^3^; A. Bicu^4^; M. Polidori^5^

##### ^1^Neuromed Campus, Cologne, Germany, ^2^memodio GmbH, Potsdam, Brandenburg, Germany; ^3^Friedrich von Bodelschwingh Klinik, Berlin, Germany; ^4^University of Heidelberg, Medical Faculty Mannheim, Mannheim, Germany; ^5^University Hospital of Cologne, Cologne, Germany

**Background and aims:** With an aging population and limited pharmacological treatments, non‐pharmacological interventions for cognitive decline are increasingly important. The MEMODIO app was developed as a multidomain digital health intervention for individuals with mild cognitive impairment (MCI) or mild dementia. This interim analysis presents initial results from an ongoing RCT (data collected until October 2024).

**Figure:**
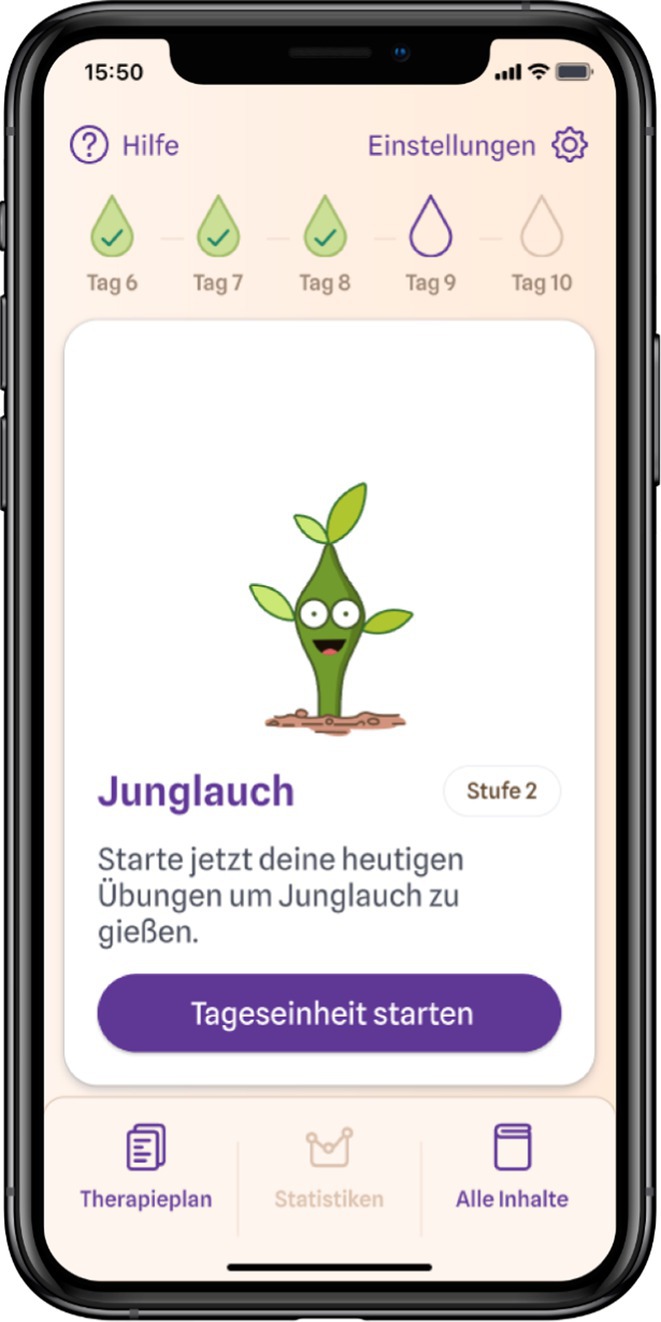
**FIGURE 1** Screenshot Health App

**Methods:** The study includes 140 patients with MCI (MoCA 21‐25) or mild dementia (MoCA 14‐20), randomized to an intervention group (IG) using MEMODIO alongside standard care or a standard of care group (SoC). MEMODIO provides a 12‐week program incorporating cognitive training, physical exercises, psychoeducation on brain‐healthy diets, and risk factor management. Assessments occurred at baseline and post‐intervention using MoCA, A‐IADL‐Q‐SV, DEMQOL, and PAQ 50+.

**Results:** Among 69 analyzed patients (mean age: 74.39 years, 32 female), 42 had MCI and 27 had dementia. Preliminary results show a statistically significant MoCA improvement in MCI patients in the IG (‐1.162 ± 3.08 SoC vs. 1.375 ± 2.286 IG, *p* = 0.000). Quality of life, physical activity, and daily functioning did not significantly change at interim evaluation.

**Figure:**
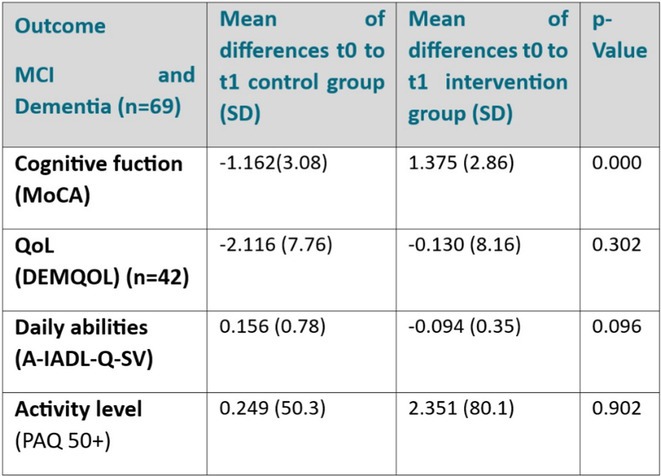
**TABLE 1** Results.

**Figure:**
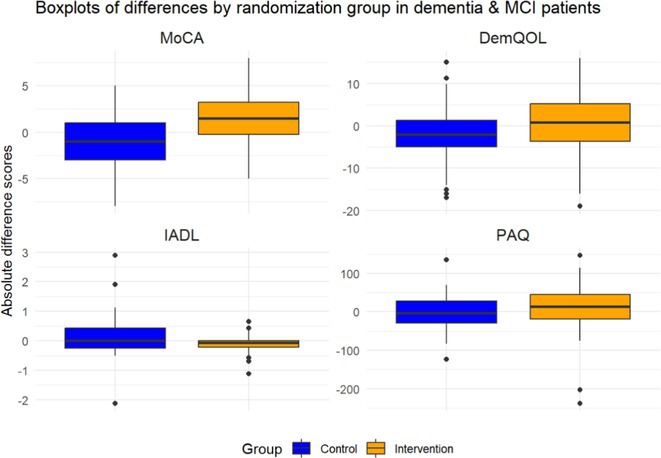
**FIGURE 2** Boxplots Results

**Conclusion:** MEMODIO significantly improved cognitive function in MCI patients, outperforming standard care alone. Ongoing analyses will assess its impact on quality of life, daily activities, and long‐term therapeutic potential.

**Disclosure:** G. Nelles: PI of MEMODIO@APP_CARE, F. Bicu: Shareholder of memodio GmbH, A. Quante: None Declared, T. Steinmann: Employee of memodio GmbH, D. Stein: Shareholder of memodio GmbH, V. Weil: Employee of memodio GmbH, A. Bicu: None Declared, C. Polidori: None Declared

## EPR‐004

### Cholinergic dysfunction as a biomarker of the Alzheimer's continuum: Insights into early‐stage cognitive decline

#### G. Foresti ^1^; A. Rizzardi^1^; A. Benussi^2^; S. Caratozzolo^1^; C. Tolassi^1^; A. Pilotto^1^; A. Padovani^1^

##### ^1^Department of Clinical and Experimental Sciences, Neurology Unit, University of Brescia, Italy; ^2^Department of Medical and Surgical Sciences and Health, University of Trieste, Italy

**Background and aims:** Subjective cognitive decline (SCD), characterized by self‐reported cognitive dysfunction despite normal performance on standardized tests, is a heterogeneous condition associated with an increased risk of developing mild cognitive impairment (MCI) and Alzheimer's disease (AD). This study aimed to characterize intracortical inhibition and facilitation across the AD continuum using non‐invasive transcranial magnetic stimulation (TMS), with a focus on identifying neurophysiological dysfunction as an early biomarker.

**Methods:** Fifty‐eight participants were enrolled, including 20 healthy controls (HC), 10 SCD, 13 MCI, and 15 AD patients, confirmed by cerebrospinal fluid (CSF) analysis. All underwent an extensive neuropsychological assessment and TMS paired‐pulse protocols to assess short interval intracortical inhibition (SICI), intracortical facilitation (ICF), and short latency afferent inhibition (SAI), reflecting GABAergic, glutamatergic, and cholinergic circuits, respectively.

**Results:** TMS revealed significant cholinergic‐mediated intracortical inhibition deficits across patient groups. Furthermore, SCD patients showed significantly higher SAI values than HC (*p* < 0.05), but comparable to MCI (*p* = 0.408), suggesting early cortical inhibitory dysfunction. Notably, 50% of SCD participants exhibited SAI alterations despite normal AD CSF markers, indicating that SAI modifications may reflect broader brain health alterations beyond AD‐related changes. Partial correlation adjusted for age and sex revealed a positive relationship between MoCA scores and SAI values.

**Conclusion:** These findings highlight that cholinergic dysfunction in SCD may serve as an early biomarker of cognitive impairment, providing insights into its pathophysiology and identifying at‐risk individuals. Further investigations are needed to explore cholinergic‐targeted interventions to prevent or slow the progression of cognitive decline.

**Disclosure:** Nothing to disclose.

## EPR‐005

### Proteomic changes in prion disease associated with prion‐specific, V2 strain‐related secondary tauopathy

#### G. Bentivenga ^1^; A. Mammana^2^; D. Gogishvili^3^; S. Baiardi^1^; E. Vittoriosi^2^; A. Mastrangelo^1^; A. Ranieri^2^; S. Abeln^3^; S. Capellari^1^; P. Parchi^1^

##### ^1^Department of Biomedical and Neuromotor Sciences (DiBiNeM), Bologna University, Bologna, Italy; ^2^IRCCS, Istituto delle Scienze Neurologiche di Bologna, Bologna, Italy; ^3^AI Technology for Life, Department of Computing and Information Sciences, Biology Department, Utrecht University, Utrecht, Netherlands

**Background and aims:** Sporadic Creutzfeldt‐Jakob disease (sCJD) is a rare neurodegenerative disorder related to prion protein misfolding. Interestingly, a secondary prion‐specific tauopathy can occur in sCJD, especially in the subtypes related to the V2 strain (i.e., VV2 and MV2K). By employing a high‐throughput proteomics technology (proximity extension assay) on cerebrospinal fluid (CSF) samples from a broad sCJD cohort, we aimed to characterize in vivo the molecular events associated with secondary tauopathy.

**Methods:** We assayed 797 proteins in the CSF samples of 67 patients with a definite or probable clinical diagnosis of V2‐sCJD (34 VV2 and 33 MV2K). Increased CSF p‐tau181 levels defined the presence of secondary tauopathy (T+ vs. T‐ status). Linear models adjusting for age, sex, and sCJD subtype, were used to identify the differentially expressed proteins (DEPs) between T+ and T‐ sCJD cases. Enrichment analyses were performed with the Gene Ontology database.

**Results:** 36/67 (53.7%) patients were classified as T+. We found 294 DEPs between T+ and T‐ sCJD cases. All proteins but two (NBL1 and APLP1) were positively associated with T+ status (Figure 1). Enrichment analyses on upregulated proteins highlighted various biological processes related to synaptic organization and neuronal morphogenesis (Figure 2). The top DEPs are involved in protein folding regulation (FKBP4), cell signaling (NTRK2, NTRK3, TNFRSF11A), ribose metabolism (RBKS), and membrane transportation (SCARB2).

**Figure:**
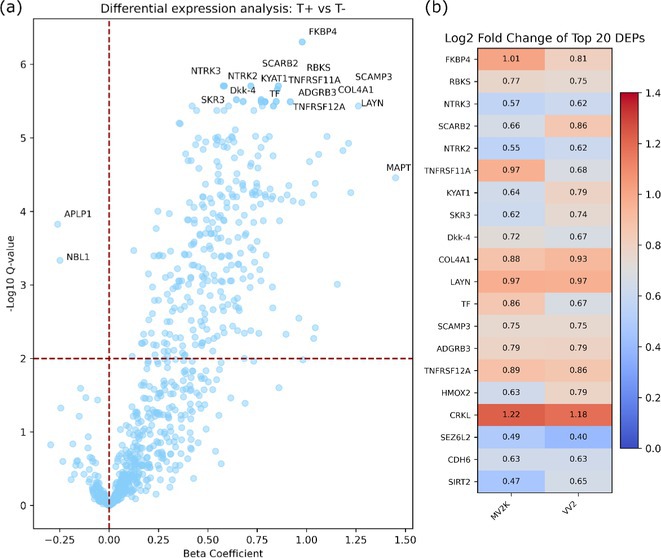
**FIGURE 1** Volcano plot showing the DEPs between T+ and T‐ sCJD patients. The top dysregulated proteins are marked with protein names (a). Heatmap showing the Log2‐Fold change distribution of the top DEPs in VV2 and MV2K (b). DEPs, differentially expressed proteins.

**Figure:**
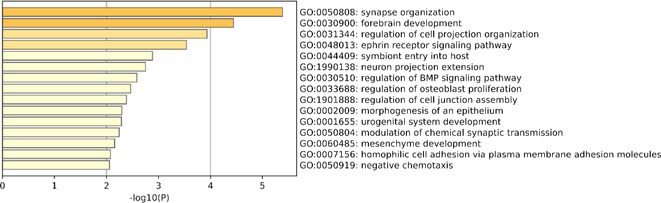
**FIGURE 2** Bar graphs showing the biological pathways enriched among proteins upregulated in T+ sCJD. Functional enrichment was performed using Metascape selecting GO Biological Processes as ontology source and setting 797 assayed proteins as enrichment background.

**Conclusion:** We unveil a distinct protein signature associated with sCJD secondary tauopathy in vivo, shedding new light on the complex molecular events related to tau misfolding in prion disease.

**Disclosure:** P.P. is supported by the Ministero della Salute (Ricerca Corrente), and the #NextGenerationEU (NGEU) funded by the Ministry of University and Research (MUR), National Recovery and Resilience Plan (NRRP), project MNESYS (PE0000006).

## EPR‐006

### Sex differences in the efficacy of anti‐amyloid monoclonal antibodies

#### J. Martinkova ^1^; A. Ferrari^2^; R. Marongiu^3^; A. Santuccione Chadha^2^

##### ^1^Department of Neurology, Second Faculty of Medicine, Charles University, Motol University Hospital, Prague, Czechia; ^2^Women's Brain Foundation; ^3^Department of Neurological surgery, Department of Genetic Medicine, Feil Family Brain and Mind institute, Weill Cornell Medical College, New York, USA

**Background and aims:** As first disease‐modifying drugs approved for the treatment Alzheimer's disease (AD), anti‐amyloid monoclonal antibodies (MABs) represent a milestone in patient care. Sex was noted to modify biomarker deposition, progression and risk, however, sex differences in MABs efficacy have not been sufficiently explored. In this meta‐analysis, we aimed to analyze all available sex‐disaggregated data for MABs efficacy in the treatment of AD.

**Methods:** We searched clinicaltrials.gov, Alzforum Therapeutics and PubMed databases to identify publications of phase III MABs clinical trials. We further supplemented our search with reference search and conference presentations. We selected those publications which presented any form of sex‐disaggregated data on efficacy. We then extracted sex‐disaggregated efficacy data for all available endpoints. We subsequently conducted a random effects meta‐regression using mean treatment effects for each endpoint.

**Results:** We identified 13 publications presenting first full results of a phase III MABs clinical trial, of which 5 (results of aducanumab, donanemab, gantenerumab, lecanemab, and solanezumab clinical trials) presented sex‐disaggregated results. The meta‐analysis revealed statistically significant sex differences for mean treatment effects of all analyzed endpoints, with greater efficacy in males and limited efficacy in females (ADAS‐Cog *p* = 0.042, ADCS‐ADL *p* = 0.002, CDR SoB *p* = 0.008).

**Conclusion:** Our results stress the importance of considering patient sex in anti‐amyloid efficacy analyses. Further analyses of detailed sex‐disaggregated efficacy and safety results are needed to make personalized risk/benefit assessments.

**Disclosure:** JNM was supported by project nr. LX22NPO5107 (MEYS): Financed by EU – Next Generation EU.

## EPR‐007

### CSF biomarkers profiling in cerebral amyloid angiopathy: Relationship with phenotype and hemorrhagic risk

#### M. Cotta Ramusino ^1^; M. Losa^2^; I. Cama^3^; L. Gualco^4^; I. Gandoglia^5^; F. Massa^2^; A. Donniaquio^6^; P. Mortola^2^; L. Argenti^2^; L. Lombardo^2^; V. Pelagotti^2^; G. Bozzo^2^; B. Orso^2^; P. Mattioli^2^; D. Arnaldi^2^; A. Cirone^5^; D. Plantone^7^; L. Lorenzini^8^; L. Falcitano^5^; F. Mazzacane^1^; G. Perini^1^; A. Costa^1^; M. Del Sette^5^; L. Farina^9^; M. Pardini^2^

##### ^1^Research Unit of Clinical Neuroscience of Dementia, IRCCS Mondino Foundation, Pavia, Italy; ^2^Department of Neuroscience, Rehabilitation, Ophthalmology, Genetics, Maternal and Child Health (DINOGMI), University of Genoa, Genoa, Italy; ^3^Dipartimento di Matematica (DIMA), Università di Genova, Genova, Italy; ^4^Neuroradiology Unit, Azienda Ospedaliero‐Universitaria Santi Antonio e Biagio e Cesare Arrigo, Alessandria, Italy; ^5^IRCCS Ospedale Policlinico San Martino, Genoa, Italy; ^6^E.O. Ospedali Galliera, Genoa, Italy; ^7^Department of Medicine, Surgery & Neuroscience, University of Siena, Siena, Italy; ^8^Dept. of Radiology and Nuclear Medicine, Amsterdam University Medical Centers, Vrije Universiteit, Amsterdam, The Netherlands; ^9^Advanced Imaging and Radiomics Center, Neuroradiology Department, IRCCS Mondino Foundation, Pavia, Italy

**Background and aims:** Cerebral amyloid angiopathy (CAA) is diagnosed in vivo according to Boston Criteria, but it remains unclear whether CSF profile can reliably provide phenotypic and prognostic insight. In this study, we explored the potential of core CSF biomarkers in identifying CAA phenotypes and providing support to hemorrhagic risk stratification.

**Methods:** We enrolled probable CAA patients (Boston Criteria 2.0) and, as control group, age‐matched AD patients without radiological signs of CAA, gathering clinical, neuroimaging and follow up data, together with core CSF biomarkers (Aβ42, Aβ40, pTau181, total‐Tau). We grouped CAA patients based on the AT(N) classification (A+CAA vs. A‐CAA and A+T+CAA vs. A+T‐CAA), to explore clinical and radiological differences. Unsupervised clustering, a data‐driven method, was applied to identify biological CAA subgroups on CSF biomarkers levels.

**Results:** CAA (*n* = 71, 71.77 ± 8.45 years) exhibited lower levels of Aβ40 (*p* < 0.001), Aβ42 (*p* = 0.013), total‐Tau (*p* = 0.040), and pTau181 (*p* < 0.001) compared to AD (*n* = 32, 72.97 ± 4.85 years), with similar Aβ42/40 ratio (*p* = 0.303). A+CAA showed higher cortical superficial siderosis prevalence than A‐CAA (67% vs. 25%; *p* = 0.016). A+T‐CAA subjects showed higher hemorrhagic risk over time than A+T+CAA (29 vs. 7 events per 100 patient‐year, *p* = 0.010; survival analysis, log‐rank test: *p* = 0.013; hazard ratio: 6.30; 95%CI: 1.18–33.72; *p* = 0.031). Unsupervised clustering identified two CSF‐based CAA subgroups, defined as “pure CAA” and “CAA‐AD”. The pure CAA group showed greater hemorrhagic risk during follow‐up compared to CAA‐AD (22 events per 100 patient‐years vs. zero events; *p* = 0.017; survival analysis, log‐rank test: *p* = 0.011).

**Conclusion:** CSF‐based profiling effectively identified CAA with different natural history, providing a promising tool for hemorrhagic risk stratification.

**Disclosure:** MP reports fees from Novartis, Lilly, Eisai, Biogen and research support from Novartis and Nutricia. FM received speaker honoraria from Roche Diagnostics S.p.A and Eli Lilly S.p.A.

## EPR‐008

### Adiponectin as a potential therapeutic target for cerebrovascular dysfunction in Alzheimer's disease

#### W. Zou ^1^; L. Yick^1^; Z. Zhang^1^; J. Kwan^1^; R. Ng^2^; K. Chan^1^

##### ^1^Department of Medicine, Li Ka Shing Faculty of Medicine, The University of Hong Kong, Hong Kong, China; ^2^Division of Neuroscience, School of Biological Sciences, The University of Manchester, UK

**Background and aims:** Cerebrovascular dysfunction are increasingly recognized as a critical aspect of Alzheimer's disease (AD) pathophysiology. Adiponectin (APN), an adipocyte‐secreted hormone, exhibits neuroprotective properties against amyloid beta (Abeta) toxicity. However, its influence on cerebrovascular dysfunction in AD remains largely unexplored.

**Methods:** APN‐deficient AD (5xFAD;APN‐/‐) mice were generated by crossbreeding 5xFAD and APN knockout (APN‐/‐) mice. Cerebrovascular integrity was assessed through cerebral blood flow (CBF), neurovascular coupling (NVC), cerebral amyloid angiopathy (CAA), and blood‐brain barrier (BBB) permeability. Additionally, 5xFAD mice received intravenous APN to evaluate its effects on CBF and NVC. Primary mouse brain endothelial cells were treated with Human Abeta40 oligomers, with or without APN, to assess the impact of APN on tight junction proteins (TJPs) expression and endothelial barrier integrity.

**Results:** 5xFAD; APN‐/‐ mice showed more severe NVC impairment as early as 6 months, and significantly lower resting CBF at 9 months than 5xFAD mice. Earlier and severer BBB leakage was observed in 5xFAD;APN‐/‐ mice, alongside increased TJPs reduction. Additionally, more Abeta deposition was observed within the cerebral vessels of 5xFAD; APN‐/‐ mice at 6 months, indicating aggravated CAA pathology. Intravenous APN administration mitigated CBF reduction and NVC impairment in 5xFAD mice. In vitro results showed that Abeta40 reduced TJPs expression and compromised endothelial barrier integrity, which was significantly improved by APN pretreatment.

**Conclusion:** These findings suggest that APN is beneficial for maintaining cerebrovascular integrity, highlighting its potential as a therapeutic target for cerebrovascular dysfunction associated with AD.

**Disclosure:** This work was supported by funding for research in AD and dementia from Chan Kin Shing Charitable Trust and private donation of W C S Fung.

## EPR‐009

### Comparing diagnostic accuracy between plasma and cerebrospinal fluid biomarkers in Alzheimer's disease

#### T. Lombardo ^1^; M. Michelutti^1^; B. Toffoletto^2^; F. Sirianni^2^; V. Cenacchi^1^; L. Pelusi^1^; A. Perego^2^; A. Menichelli^1^; T. Cattaruzza^1^; A. Benussi^1^; P. Manganotti^1^

##### ^1^1Neurology Unit, Department of Medical, Surgical and Health Sciences, University of Trieste, Trieste, Italy; ^2^Unit of Laboratory Medicine, Department of “Medicina dei Servizi” – ASUGI, Trieste, Italy

**Background and aims:** Recently developed plasma biomarkers for Alzheimer's disease (AD) show promise for improving the screening process in clinical settings, facilitating earlier access to emerging treatments. This study aimed to assess the concentrations of plasma AD biomarkers in relation to cerebrospinal fluid (CSF) biomarkers, evaluating their correlation and diagnostic accuracy for AD diagnosis.

**Methods:** We included 52 participants (18 with suspected AD, 11 with mild cognitive impairment, and 28 healthy controls) from the Memory Clinic at the University of Trieste. Participants underwent lumbar puncture for CSF analysis, and plasma AD biomarkers (Aβ42, Aβ40, p‐Tau181) were tested using the LUMIPULSE immunoassay. CSF biomarkers included Aβ42, Aβ40, Aβ42/Aβ40 ratio, t‐Tau, and p‐Tau181.

**Figure:**
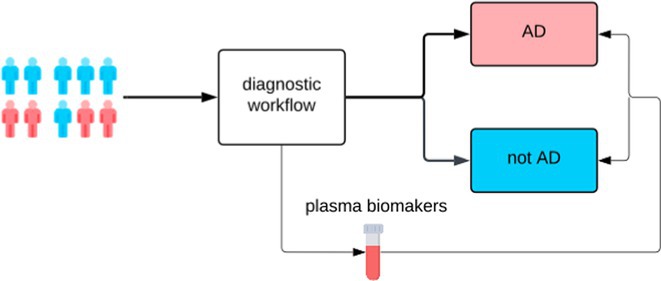
**FIGURE 1** Study design

**Results:** Plasma p‐Tau181 concentrations were significantly higher in amyloid‐positive (A+) individuals (*p* < 0.001) and increased in the A+/T+ group (*p* < 0.001), but not in A+/T‐. Plasma p‐Tau181 showed a strong correlation with its CSF counterpart and with the CSF Aβ42/40 ratio. No significant correlation was found between plasma and CSF Aβ42/Aβ40 ratios. Receiver operating characteristic (ROC) analyses indicated plasma p‐Tau181's high diagnostic accuracy, with an AUC of 0.82 for differentiating A+ versus A‐ and 0.83 for A+/T+ versus other CSF A/T statuses.

**Figure:**
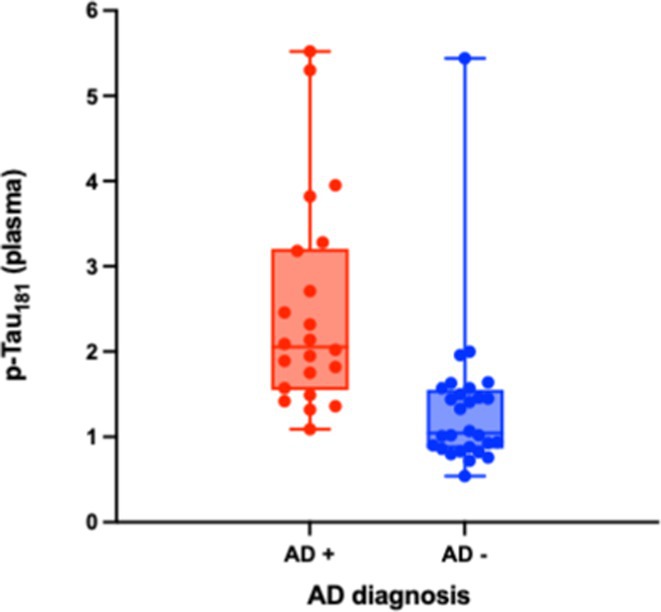
**FIGURE 2** Plasma biomarkers concentrations according to AD diagnosis.

**Figure:**
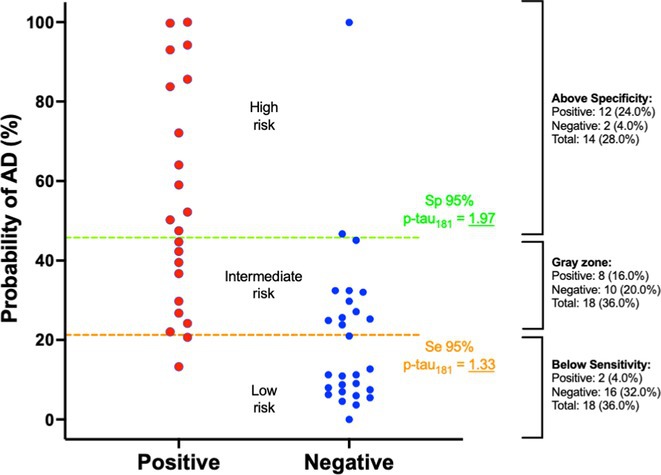
**FIGURE 3** Plasma p‐Tau181 based risk stratification for AD positivity.

**Conclusion:** Plasma p‐Tau181 is a reliable biomarker with strong correlation to CSF biomarkers and high diagnostic accuracy, supporting its use in AD screening and diagnosis.

**Disclosure:** Nothing to disclose.

## Autonomic nervous system diseases

## EPR‐010

### MeDeMSA Care protocol: personalized best medical care with integrated telemedicine and mobile palliative support in MSA

#### A. Fanciulli ^1^; B. Caliò^1^; S. Schmidt^1^; G. Goebel^1^; F. Leys^1^; K. Breitegger^2^; A. Blum^1^; O. Galvan^1^; A. Herms^1^; B. Hoegl^1^; F. Jagusch^1^; S. Kiechl^1^; M. Koegl^3^; I. Kuchin^4^; S. Lorenzl^5^; W. Poewe^1^; G. Rumpold^1^; A. Schrag^6^; K. Seppi^1^; U. Siebert^4^; M. Schmidhuber^2^; P. Schwingenschuh^3^; B. Jahn^4^; F. Krismer^1^; G. Wenning^1^

##### ^1^Medical University of Innsbruck, Austria; ^2^University of Graz, Austria; ^3^Medical University of Graz, Austria; ^4^UMIT Tirol; ^5^Paracelsus Medical University of Salzburg, Austria; ^6^University College London, UK

**Background and aims:** Multiple system atrophy (MSA) represents a major management challenge due to its variable clinical presentation. At the wheelchair‐bound stage, barriers often hinder specialized care, leaving patients and caregivers to face complications and fear alone.

**Methods:** This 18‐month, monocentric, randomized, open‐label study evaluates the impact of a personalized, multidisciplinary treatment plan, integrating mobile palliative care and telemedicine, on the quality of life (QoL) of MSA individuals, compared to a historical European MSA cohort. Forty‐six participants will undergo baseline clinical, psychological, and neuro‐rehabilitation assessments, along with an online interview to identify individual healthcare preferences. These assessments will guide individualized therapeutic plans, including palliative care, self‐directed physio‐, speech, and occupational exercises. Follow‐up visits at 6, 12, and 18 months will reassess needs and adapt plans to address disease progression and changing preferences, ensuring continuous personalization. Repeated interviews at 12 months, phone‐calls and satisfaction surveys at Months 1, 7, 13, and 18 will monitor compliance, identify barriers, and gather feedback. Additionally, 23 participants will receive monthly and on‐demand telemedicine visits. Informal caregivers will join an 18‐month observational study assessing their QoL and burden through repeated evaluations, offering insights into evolving challenges.

**Results:** MeDeMSA Care startedin April 2023 and will last 60 months. To date, 20 MSA individuals (10 randomized to telemedicine) and 17 informal caregivers were actively recruited.

**Conclusion:** We hypothesize that multidisciplinary, patient‐centered care with integrated telemedicine and mobile palliative support warrants continuity of care and improves the QoL of MSA individuals throughout the disease course. Its acceptance, safety, and cost‐effectiveness will also be assessed.

**Disclosure:** Funded by the FWF‐Austrian Science Fund (FG 2700‐B).

## EPR‐011

### Multi‐level spinal cord neuromodulation effectively treats postural orthostatic tachycardia syndrome (POTS)

#### C. Rizea ^1^; J. Paz^1^; I. Huertas^2^

##### ^1^Hospital Universitario La Paz, Madrid, Spain; ^2^Boston Scientific

**Background and aims:** Postural orthostatic tachycardia syndrome (POTS) is a complex disorder that causes invalidating symptoms (e.g., tachycardia, presyncope, fatigue, dyspnea) after assuming an upright position. In severe cases, patients may become functionally disabled and develop other serious dysautonomia symptoms such as gastrointestinal. It mostly affects young women of childbearing age and prevalence has been reported to spike after COVID. Currently, there is no approved treatment, and management relies on lifestyle and diet changes, heart medications, and other supportive measures that, unfortunately, do not control the symptoms very often.

**Methods:** We are treating patients with severe POTS with a multi‐level Spinal Cord Stimulation (SCS) approach: 4‐port system and thoracic and cervical electrodes. Standard assessments for POTS are performed pre‐ and post‐SCS (1, 3, 6, 12 months): pain (VAS), quality‐of‐life (SF‐36), autonomic dysfunction (BASQ), and heart rate response to tilt (∆HR).

**Results:** To date, 2 subjects (2 female, 28yo) have been implanted with success. Several other patients will be implanted in the coming months. The first patient (3y evolution) cardinal symptoms included POT (∆HR = 70bpm), frequent fainting, and dysautonomia with severe sensory deficits, gastrointestinal and urogenital issues. After SCS, response to tilt normalized (∆HR = 21bpm, 1 month; ∆HR = 12bpm, 12 months), thus no longer meeting criteria for POTS. Dysautonomia symptoms and Quality‐of‐life also largely improved. The second patient (12y evolution with 7y in wheelchair) had also severe POT (∆HR = 45bpm) and multi‐system dysautonomia. After SCS, similarly, response to tilt decreased (∆HR = 30bpm, 1 month) and dysautonomia and Quality‐of‐life largely improved.

**Figure:**
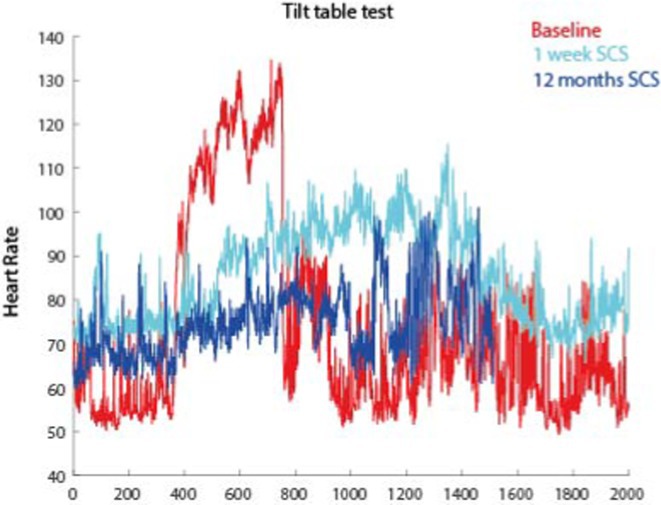
**FIGURE 1** Long‐term heart rate response to tilt (before and after SCS) of our first patient.

**Conclusion:** Neuromodulation via the spinal cord holds big promise to effectively treat POTS and associated dysautonomia.

**Disclosure:** Nothing to disclose.

## EPR‐012

### Central autonomic network connectivity: Abnormalities in multiple sclerosis and aerobic training effects

#### G. Guido ^1^; P. Valsasina^2^; T. Morozumi^3^; P. Preziosa^1^; F. Romanò^2^; M. Filippi^4^; M. Rocca^1^

##### ^1^Neuroimaging Research Unit, Division of Neuroscience, and Neurology Unit, IRCCS San Raffaele Scientific Institute, and Vita‐Salute San Raffaele University, Milan, Italy; ^2^Neuroimaging Research Unit, Division of Neuroscience, IRCCS San Raffaele Scientific Institute, Milan, Italy; ^3^Neuroimaging Research Unit, Division of Neuroscience, IRCCS San Raffaele Scientific Institute, and Vita‐Salute San Raffaele University, Milan, Italy; ^4^Neurology Unit, Neurorehabilitation Unit, Neurophysiology Service, and Neuroimaging Research Unit, Division of Neuroscience, IRCCS San Raffaele Scientific Institute, and Vita‐Salute San Raffaele University, Milan, Italy

**Background and aims:** Autonomic dysfunction is common in multiple sclerosis (MS); however, functional abnormalities of the central autonomic network (CAN) are still not investigated in this disease. Here, we explored resting state (RS) functional connectivity (FC) of the CAN in MS and its potential modification after aerobic training (AT).

**Methods:** A total of 75 MS patients (38 relapsing‐remitting [RR] and 37 progressive [P]) underwent 3T RS functional MRI at baseline and after 2 (RRMS) or 3 months (PMS) of AT. Sixty‐seven matched healthy controls (HC) served as baseline RS FC reference. Seed‐based RS FC analysis used core CAN modulatory regions: left/right ventromedial pre‐frontal cortex (vmPFC), mid‐cingulate cortex (MCC), amygdala, hypothalamus, anterior and posterior insula.

**Results:** Compared to HC and PPMS patients (conjunction analysis, *p* < 0.001), RRMS patients were characterized by increased RS FC of the left hypothalamus with the cerebellum. In PMS, compared to RRMS and HC (conjunction analysis, *p* < 0.001), we found increased RS FC of the bilateral insula and amygdala with ipsilateral deep gray matter nuclei, of the vmPFC with posterior cingulate cortex and angular gyrus, and of the MCC with the cerebellum. PMS also showed decreased RS FC of the bilateral insula with cerebellum, and of the MCC with insula. After AT, decreased hypothalamic network RS FC in RRMS was observed, while no changes were detected in PMS.

**Conclusion:** CAN dysregulation is present in MS, with distinct RS FC abnormalities characterizing RRMS and PMS patients. AT may be insufficient to modulate CAN RS FC in progressive patients.

**Disclosure:** Funding. Grants from Italian Ministry of Health (GR‐2019‐12369599) and MS Society of Canada (EGID3185). Disclosures. GG, PV, TM, FR have nothing to disclose. PP received speaker honoraria from Roche, Biogen, Novartis, Merck, Bristol Myers Squibb, Genzyme, Horizon, and Sanofi. He received research support from Italian Ministry of Health and Fondazione Italiana Sclerosi Multipla (FISM). MF received compensation for consulting services or speaking activities from Alexion, Almirall, Bayer, Biogen, Celgene, Chiesi Italia SpA, Eli Lilly, Genzyme, Janssen, Merck‐Serono, Neopharmed Gentili, Novartis, Novo Nordisk, Roche, Sanofi Takeda, and TEVA; Advisory Boards for Alexion, Biogen, Bristol‐Myers Squibb, Merck, Novartis, Roche, Sanofi, Sanofi‐Aventis, Sanofi‐Genzyme, Takeda; scientific direction of educational events for Biogen, Merck, Roche, Celgene, Bristol‐Myers Squibb, Lilly, Novartis, Sanofi‐Genzyme; he receives research support from Biogen Idec, Merck‐Serono, Novartis, Roche, the Italian Ministry of Health, the Italian Ministry of University and Research, and FISM. MAR received consulting fees from Biogen, Bristol Myers Squibb, Eli Lilly, Janssen, Roche, and speaker honoraria from AstraZaneca, Biogen, Bristol Myers Squibb, Bromatech, Celgene, Genzyme, Horizon Therapeutics Italy, Merck Serono SpA, Novartis, Roche, Sanofi and Teva, she receives research support from the MS Society of Canada, the Italian Ministry of Health, the Italian Ministry of University and Research, and FISM.

## EPR‐013

### Cardiovascular autonomic failure in isolated REM sleep behavior disorder and Parkinson Disease: A prospective evaluation

#### L. Baldelli ^4^; L. Sambati^2^; F. Di Laudo^1^; I. Cani^4^; G. Giannini^4^; P. Guaraldi^2^; G. Mainieri^2^; G. Loddo^3^; B. Calò^1^; E. Umbertini^1^; G. Carrozzo^1^; A. Cecere^2^; F. Mignani^2^; P. Cortelli^4^; F. Provini^4^; G. Calandra‐Buonaura^4^

##### ^1^Department of Biomedical and Neuromotor Sciences, University of Bologna, Bologna, Italy; ^2^IRCCS Istituto delle Scienze Neurologiche di Bologna, Bologna, Italy; ^3^Dipartimento delle Cure Primarie, Azienda USL di Bologna, Bologna, Italy; ^4^Department of Biomedical and Neuromotor Sciences, University of Bologna, Bologna, Italy and IRCCS Istituto delle Scienze Neurologiche di Bologna, Bologna, Italy

**Background and aims:** Isolated REM sleep behavior disorder (iRBD) is prodromal to synucleinopathies, including Parkinson's disease (PD). PD with RBD in the early phase (PD+RBD) is associated with more severe symptoms, including cardiovascular autonomic failure (cAF). Whether cAF is more related to RBD or to PD has to be confirmed.

**Methods:** One hundred early ( < 3 years) PD (20 with RBD), and 40 iRBD were prospectively evaluated with cardiovascular reflex tests (CRTs) at baseline and after 1.85 ± 0.60 years. Mixed‐effects sex‐ and age‐adjusted regression models assessed baseline and longitudinal differences.

**Results:** At baseline iRBD (mean age 66.57 ± 5.99 years, 17.5% female) exhibited more severe cAF than PD (62.53 ± 8.23 years, 35.0% females), with more frequent neurogenic orthostatic hypotension (nOH – 15.0%vs. 4.0%, *p* = 0.022) and abnormal blood pressure responses to CRTs (pathological Valsalva Maneuver – VM overshoot in 47.4% vs. 18.0%, *p* = 0.001). The prevalence and severity of cAF was similar between iRBD and PD+RBD (nOH – 20%, *p* = 0.563; pathological VM overshoot – 50.0%, *p* = 0.708). Longitudinal data demonstrated progressive deterioration of baroreflex function, with increased prevalence of nOH in iRBD and PD+RBD (incident nOH in 4 and 3 patients respectively; yearly odds ratios ‐ OR = 5.47 *p* = 0.003 and 2.30 *p* = 0.046), not significant in PD‐RBD and PD as a whole (OR = 1.80 and 0.99, *p* = 0.165 and 0.983). Prevalence of pathological VM overshoot increased only in PD+RBD (OR = 7.83, *p* = 0.041).

**Figure:**
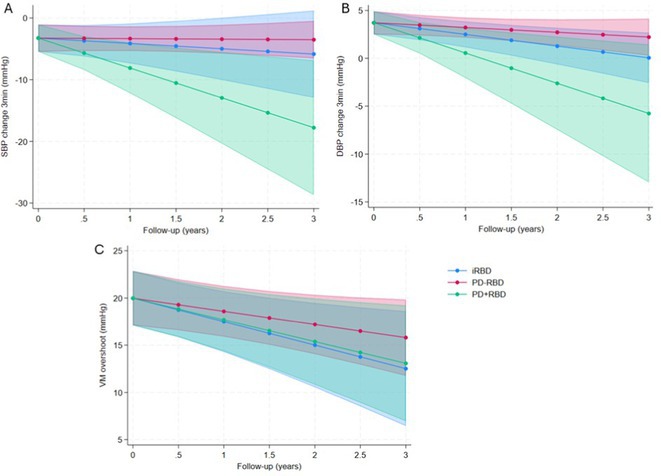
**FIGURE 1** Longitudinal model of systolic (A) and diastolic (B) blood pressure change at third minute during tilt test and VM overshoot (C) changes over the years in iRBD, PD patients with (PD+RBD) and without RBD (PD‐RBD).

**Conclusion:** The neurodegeneration underlying cAF is more closely associated with RBD than with PD phenotype. Autonomic dysfunction worsens over time predominantly in the presence of RBD, regardless of phenoconversion status, highlighting RBD as a key driver of autonomic failure.

**Disclosure:** Nothing to disclose.

## EPR‐014

### Types of pain in multiple system atrophy

#### N. Campese ^1^; M. Quamar^2^; A. Chiriac^2^; G. Göbel^1^; J. Wanschitz^1^; A. Schlager^1^; B. Caliò^1^; F. Leys^1^; P. Bower^3^; L. Kellerman^3^; L. Zamarian^1^; K. Bannister^2^; A. Schrag^4^; R. Freeman^5^; H. Kaufmann^6^; R. Granata^1^; S. Kiechl^1^; W. Poewe^1^; K. Seppi^1^; G. Wenning^1^; R. Chaudhuri^2^; A. Fanciulli^1^

##### ^1^Medical University of Innsbruck, IInnsbruck, Austria; ^2^King's College London, London, UK; ^3^Mission MSA, McLean, USA; ^4^University College London, London, UK; ^5^Harvard Medical School, Boston, Massachusetts, USA; ^6^New York University Grossman School of Medicine, New York, USA

**Background and aims:** Pain affects 87% of individuals with multiple system atrophy (MSA), but which pain types mostly contribute to pain burden remains unclear. Here we estimated the prevalence of different pain types in MSA.

**Methods:** We analyzed the prevalence of different pain types classified by the King's Parkinson's Disease Pain Questionnaire (KPPQ), and of further putative MSA‐specific causes (i.e., coat‐hanger pain, pain related to catheterization, bladder infections and spasms, pressure sores, bruises, cold hands and feet) in MSA subjects, who answered a web‐based survey in 2023. MSA individuals were matched for gender, age ( ± 3years), and disease‐duration ( ± 2years) with PD subjects and HCs from the King's college who had completed the KPPQ.

**Results:** Among 264 MSA individuals who accessed our survey, 194 were retained after data cleaning, of which 157 with completed KPPQ. Nocturnal (73%), musculoskeletal (63%), fluctuation‐related pain (62%) and, among MSA‐related types, coat‐hanger pain (59%), pain related to cold‐hands/feet (48%), and to bruises (44%) occurred most frequently. In the matched subgroup (*n* = 96), all pain types were more frequent in MSA compared to HCs, except musculoskeletal pain, which was as frequent in MSA as in HCs (63% vs. 66%, *p* = 0.722) but more common in PD than in MSA (78% vs. 63%, *p* = 0.023). Orofacial pain was more frequent in MSA compared to PD (32% vs. 12%, *p* < 0.001).

**Conclusion:** Both disease‐related (e.g., orthostatic hypotension‐related coat‐hanger pain) and unrelated (e.g., musculoskeletal) pain types contribute to pain burden in MSA. Tailored tools may help identify disease‐specific pain types that may benefit from optimized symptomatic management of core motor and non‐motor features.

**Disclosure:** Nothing to disclose related to the content of this study.

## EPR‐015

### Sudomotor innervation in al amyloidosis

#### P. Kokotis ^1^; C. Bountziouka^1^; D. Fotiou^2^; E. Kastritis^2^

##### ^1^First Department of Neurology, National and Κapodistrian University of Athens, Athens, Greece; ^2^Department of Clinical Therapeutics, National and Kapodistrian University of Athens, Athens, Greece

**Background and aims:** Autonomic nervous system (ANS) is often involved in AL amyloidosis patients. Sudomotor innervation (SI) has recently been established as a reliable index of ANS evaluation. The aim of this study was to assess the SI in AL amyloidosis patients.

**Methods:** This study included forty‐eight recently diagnosed consecutive patients (21men) mean age 59.33 (range 46‐70 years), and 31 age and gender matched controls (12 men) mean age 56,64 (range46‐79 years). Skin biopsy at the distal leg performed in all subjects and stained with PGP 9.5 panaxonal marker. We used a standardized grid of circles superimposed upon the 20x image immunofluorescent specimen to create a simple pattern of circles over the sweat gland. The percentage of nerve fibers crossed circles was used to quantify the SI.

**Results:** Average SI was significantly lower for the patients: 21.49 ± 12.74 versus 30 ± 8.95, *p* = 0.002 (t‐test). Patients with intraepidermal nerve fiber density (IENFD) reduction under the lower for their age limits, showed significantly reduced SG density: 18.09 ± 10.98 versus 28.29 ± 13.6, *p* = 0.007 (t‐test). In contrary autonomic symptoms were not associated with reduced SI 25.86 ± 16.8 versus 19.89 ± 10.81, *p* = 0.22 (t‐test).

**Figure:**
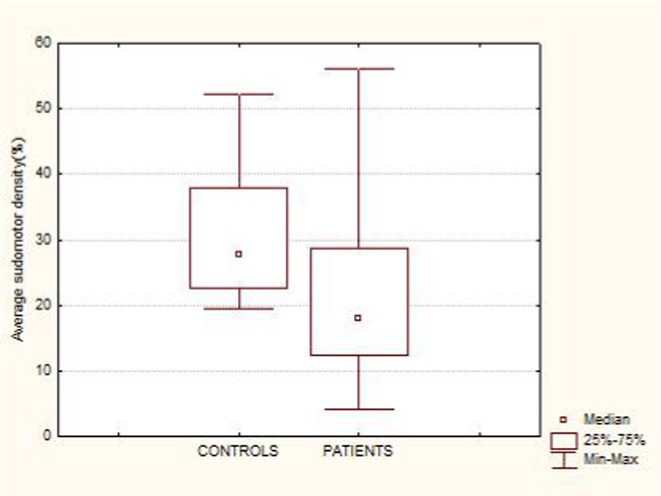
**FIGURE 1** Comparison of sudomotor nerve average density in skin biopsy for control and AL amyloidosis patients.

**Figure:**
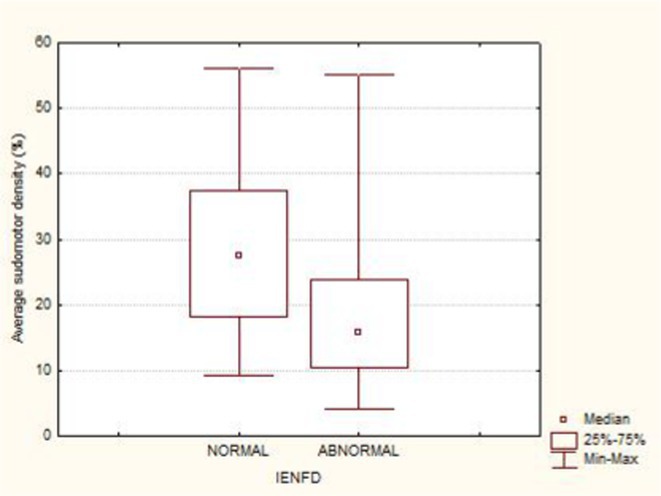
**FIGURE 2** Comparison of sudomotor nerve average density for AL amyloidosis patients with normal and abnormal IENFD in skin biopsy

**Figure:**
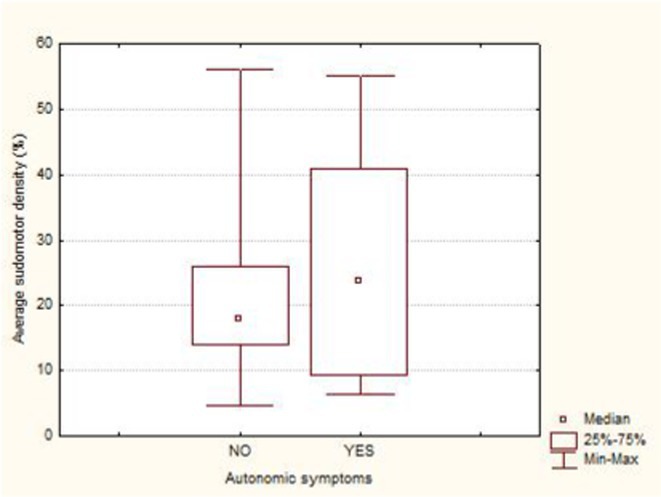
**FIGURE 3** Comparison of sudomotor nerve average density in skin biopsy for AL amyloidosis patients with and without autonomic symptoms

**Conclusion:** SI is significantly reduced in AL amyloidosis patients, specifically those with reduced IENFD, indicating ANS involvement mostly associated with small fiber neuropathy. SI could help with early recognition of small nerve fiber involvement in AL amyloidosis patients and potentially serve as a biomarker for their prognosis.

**Disclosure:** Nothing to disclose.

## EPR‐016

### Autonomic cardiovascular reflexes tests in sarcoidosis patients

#### P. Kokotis; A. Tsakali; E. Gialafos

##### First Department of Neurology, National and Kapodistrian University of Athena, Athens, Greece

**Background and aims:** Methods The peripheral nervous system is often involved in sarcoidosis, including the autonomic nervous system (ANS). ANS symptoms are mainly associated with small nerve fiber neuropathy (SFN). Most studies investigated heart rate variability (HRV) in patients with sarcoidosis but not the autonomic cardiovascular reflexes tests. The aim of this study was to evaluate the ANS function in patients with sarcoidosis, using the objective Ewing.

**Methods:** Autonomic cardiovascular reflexes tests (active standing, deep breathing, hand grip and Valsalva maneuver) were performed in 49 patients (24 men) with sarcoidosis mean age 46.52 years (range 29‐73). Patients presented mainly with lungs and less cardiac involvement, but without obvious ANS symptoms except in 2 of them.

**Results:** The reduction of blood pressure below the normal limits during active standing was the most often abnormal finding in 40/49 patients (81.63%). The other tests were mostly normal, but in total 2 or more results were abnormal for 35/49 patients (71.42%), indicating definite ANS dysfunction according to Ewing criteria.

**Figure:**
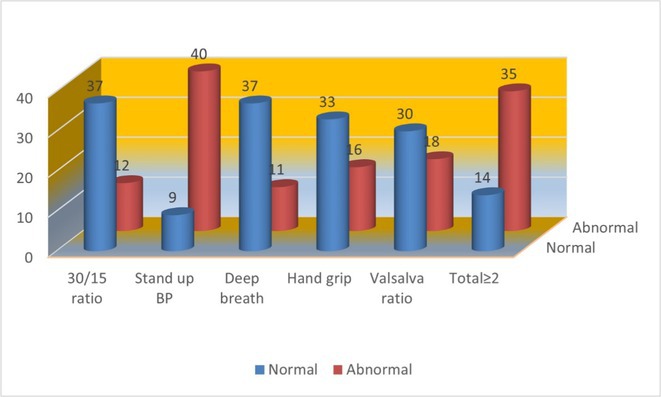
**FIGURE 1** The numbers of sarcoidosis patients with normal and abnormal Ewing tests results.

**Conclusion:** Our results reveal that most sarcoidosis patients present autonomic dysfunction more often than the reports for autonomic symptoms in patients with diagnosis of SFN. The active standing blood pressure reduction has been revealed as the most sensitive Ewing test result for the evaluation of ANS involvement in sarcoidosis. This might serve as a useful biomarker for monitoring as well as for prognosis of the patients with systemic sarcoidosis.

**Disclosure:** Nothing to disclose.

## EPR‐017

### Multiple system atrophy associated with postganglionic cardiovascular denervation: A distinct subtype?

#### R. Telese; G. Devigili; V. Leta; L. Romito; R. Cilia; C. Fabiana; A. Braccia; N. Golfré Andreasi; G. Gaudiano; R. Eleopra; A. Elia

##### “C. Besta” Neurological Institute, Department of Clinical Neurosciences, Parkinson and Movement Disorders Unit, Milan, Italy.

**Background and aims:** Cardiovascular autonomic dysfunction in Multiple System Atrophy (MSA) is mostly related to preganglionic degeneration. However, reduced tracer uptake on iodine‐123‐metaiodobenzylguanidine(123I‐MIBG) cardiac scintigraphy, reflecting postganglionic compromise, has been reported in up to one third of MSA patients. Whether these patients have a different phenotype is unclear. The aim of this study was to outline clinical/investigational features and autonomic profile of patients affected by MSA and postganglionic sympathetic denervation.

**Methods:** A retrospective study on patients affected by MSA, who underwent cardiac 123I‐MIBG scintigraphy, was performed. Clinical features, scale scores, MRI markers, plasma catecholamine values, and cardiovascular autonomic tests findings were compared among patients with and without cardiac postganglionic denervation.

**Results:** Fifty‐three patients were included, 42(79.2%) with normal(N) and 11(20.8%) with reduced(R) cardiac sympathetic innervation. 43 patients had parkinsonian and 10 had cerebellar phenotypes. R was associated with hyposmia (patient‐reported). Heart rate variability analysis showed that patients with R had reduced LF/HF (low frequencies/high frequencies) ratios while standing on tilt‐test. A sub‐analysis on patients with MSA‐P diagnosis confirmed the association between R and hyposmia; patients with MSA‐P and N had more severe and earlier incidence of dysphagia. Other variables examined were comparable among groups.

**Conclusion:** Postganglionic cardiovascular denervation in patients affected by MSA was associated with self‐reported hyposmia, atypical for MSA. Preganglionic degeneration was associated with earlier dysphagia, congruent with “pure” MSA. Patients with R had reduced LF/HF while standing on tilt‐test, reflecting sympathetic dysfunction. MSA associated with postganglionic sympathetic denervation may therefore constitute a distinct subtype, but the underlying mechanism remains unclear and needs further investigation.

**Disclosure:** The authors declare no disclosure.

## EPR‐018

### Autonomic challenges reveal recovery of cardiovascular autonomic dysfunction three and six months after stroke

#### R. Wang ^1^; J. Koehn^1^; B. Kallmünzer^1^; M. Köhrmann^2^; C. Blinzler^1^; M. Hilz^1^

##### ^1^Department of Neurology, University of Erlangen‐Nuremberg, Erlangen, Germany; ^2^Department of Neurology, University Hospital Essen, Essen, Germany

**Background and aims:** Stroke may cause cardiovascular autonomic dysfunction (CAD). We previously showed that CAD at rest may recover within days. It is unclear whether autonomic challenge‐maneuvers uncover post‐stroke CAD after several months. Therefore, we assessed cardiovascular autonomic modulation in stroke patients during autonomic challenges within one week, three and six months after stroke‐onset.

**Methods:** In 65 patients with ischemic stroke [26 women, mean age 64.2 ± 8.6 years, median NIHSS 1], we recorded RR‐intervals (RRI), systolic, diastolic blood‐pressure (BPsys, BPdia), and respiration during metronomic‐deep‐breathing (MDB), Valsalva‐maneuver, and standing‐up within one week, three and six months after stroke‐onset. We calculated E/I‐ratios, Valsalva‐ratios, and 30/15‐ratios. Values lower than the age‐dependent reference values of our laboratory were considered abnormal.

**Results:** Within one week, three and six months after stroke‐onset, E/I‐ratios were abnormal in 9/65, 3/65, and 3/65 patients respectively; Valsalva‐ratios were abnormal in 4/65, 0/65, and 0/65 patients respectively, 30/15‐ratios were abnormal in 2/65, 1/65, and 1/65 patients respectively. Three months after stroke, E/I‐ratios, Valsalva‐ratios, and 30/15‐ratios were significantly higher than the respective values assessed within the first week after stroke. Six months after stroke, Valsalva‐ratios and 30/15‐ratios also were higher than the respective values of the first week assessment.

**Conclusion:** The autonomic challenge‐maneuvers unveiled CAD only in rather few patients, probably due to the low stroke‐severity. MDB was most sensitive and demonstrated post‐stroke CAD in 13.8% of patients during the first week, in 6.2 % three and six months after stroke. Valsalva‐ratios and 30/15‐ratios upon standing‐up were less sensitive but also showed CAD‐recovery after three and six months.

**Disclosure:** Nothing to disclose.

## Cerebrovascular diseases 1

## EPR‐019

### Symptomatic intracerebral hemorrhage and CSF biomarkers in cerebral amyoid angiopathy

#### B. Storti^1^; A. Francia ^5^; G. Marinoni^1^; B. Cefaloni^2^; N. Rifino^1^; G. Boncoraglio^1^; A. Indaco^3^; G. Di Fede^3^; M. Stanziano^4^; I. Canavero^1^; A. Bersano^1^

##### ^1^Cerebrovascular Unit, Fondazione IRCCS Istituto Neurologico Carlo Besta, Milan, Italy; ^2^Dipartimento di Matematica, Politecnico di Milano, Milan, Italy; ^3^Neuropathology Unit, Fondazione IRCCS Istituto Neurologico Carlo Besta, Milan, Italy; ^4^Neuroradiology Unit, Fondazione IRCCS Istituto Neurologico Carlo Besta, Milan, Italy; ^5^Department of Biomedical and Clinical Sciences, University of Milan, Italy

**Background and aims:** Cerebral amyloid angiopathy (CAA) typically presents as lobar intracerebral hemorrhage (ICH) or cognitive impairment. The Boston criteria 2.0 are highly accurate in the setting of hemorrhagic phenotypes but yield a much lower diagnostic value in non‐hemorrhagic CAA cases. Cerebrospinal fluid (CSF) biomarkers may provide valuable supportive evidence.

**Methods:** We collected prospective clinical, radiological data along with molecular CSF biomarkers (amyloid‐beta‐40, amyloid‐beta‐42, t‐ Tau, p‐Tau, amyloid‐beta‐42/amyloid‐beta‐40 and p‐Tau/amyloid‐beta‐42 ratio) from a cohort of CAA patients recruited in our cerebrovascular outpatient clinic. Patients were divided into two groups (CAA‐ICH+ and CAA‐ICH‐, respectively), based on whether they had a symptomatic lobar ICH before the lumbar puncture.

**Results:** Fifty‐four patients were included: 35 CAA‐ICH+ and 19 CAA‐ICH‐ (male 62.86% vs. 47.37%, *p* = 0.42, mean age 63.37 vs. 67.79, *p* = 0.13, respectively). Mean age at lumbar puncture was not significantly different (63.80 vs. 67.89, *p* = 0.17, ICH+ vs. ICH‐). The number of patients with cognitive impairment was similar in ICH+ and ICH‐ groups (57.14% vs. 47.37% *p* = 0.68, respectively). Levels of CSF amyloid‐beta‐42, t‐Tau, p‐Tau and the ratios of amyloid‐beta‐42/amyloid‐beta‐40 and p‐Tau/amyloid‐beta‐42 were comparable in the two groups. However, the mean value of CSF amyloid‐beta‐40 in CAA‐ICH+ was significantly lower than in CAA‐ICH‐ (4701.45 vs. 6651, *p* = 0.0016).

**Conclusion:** Overall patients with CAA are characterized by low levels of CSF Abeta‐40 and Abeta‐42. Interestingly, patients who suffered a symptomatic lobar hemorrhage showed lower CSF Abeta‐40 levels compared to non‐hemorrhagic cases. CSF biomarkers levels could support the diagnosis of non‐hemorrhagic CAA cases and stratify the hemorrhagic risk.

**Disclosure:** Nothing to disclose.

## EPR‐020

### Monogenic stroke in the young‐age stroke in Skåne study

#### A. Ilinca

##### Department of Clinical Sciences Lund, Neurology, Skåne University Hospital, Lund University, Lund, Sweden

**Background and aims:** Our previous studies indicated that 47% of patients with a stroke before age 56 have a positive family history of stroke. Monogenic conditions may play an important role for these groups of patients.

**Methods:** Since January 2021, our database includes patients under 56 years of age at their first stroke episode. We recruit patients from Skåne Region who were in contact with our hospital. We collect information on their vascular risk factors (hypertension, diabetes mellitus, hypercholesterolemia, heart disease, history of smoking), clinical stroke characteristics and comorbidities. Medical records for both living and deceased family members are reviewed whenever available. Affected and unaffected family members from selected probands are also included. All persons included in the study are examined by one neurologist (the first author). Blood samples are collected from all participants. Whole genome sequencing (WGS) is performed and analyzed using our updated Stroke Gene Panels.

**Results:** Until December 2024, 187 probands were included in the study, 39% over 49 years and 61 % were men. The etiology remained “undetermined embolic” for 40% of them, while 5% had non‐genetic causes related to secondary anti‐phospholipidic syndrome, exposure to toxic substances, paramalignant syndrome, malignancy or cerebral vasculitis. WGS data of 114 probands with heredity for stroke or for similar vascular diseases with the proband or without classical vascular risk factors is analyzed.

**Conclusion:** We show clinical and genetic data of a larger group of patients with early stroke, systematically included under 4 years interval. Results from 50 patients were previously published, PMID:39498567.

**Disclosure:** Nothing to disclose.

## EPR‐021

### Single‐cell RNA‐seq analysis reveals ferroptosis of venous endothelial cells in cerebral amyloid angiopathy

#### C. Wu; X. Ye; Y. Jia; S. Huang; S. Zhu

##### Department of Neurology, Tongji Hospital, Tongji Medical College, Huazhong University of Science and Technology, Wuhan, China

**Background and aims:** CAA is an age‐related cerebral small vessel disease (CSVD) defined by β‐amyloid (Aβ) deposition in cortical and leptomeningeal vessels. The integrity of both the structure and function of the neurovascular unit is critical for the efficient clearance of excess Aβ accumulated in the brain. Characteristics of cerebrovascular cells in CAA remain poorly understood at single‐cell resolution due to their sparsity and dispersion.

**Methods:** Purified microvessels from the cerebral cortex of three groups of 11‐month‐old male APP23 transgenic mice and age‐ and sex‐matched wild‐type C57BL6J mice were collected for single‐cell RNA sequencing (scRNA‐seq) analysis. Our findings were verified using western blotting and immunofluorescence.

**Results:** A total of ~26,000 cerebrovascular cells across 8 subtypes were captured, categorized into three meta clusters, including endothelial cells (arteries, veins and capillaries), mural cells (smooth muscle cells and pericytes), and immune cells (microglia, monocytes and B/NK cells). Endothelial cells (ECs) were particularly decreased in the APP23‐Tg group. Functional enrichment analysis indicated the exclusively activated ferroptosis in venous ECs, especially in the APP23‐Tg group. Western blotting and immunofluorescence further validated our findings. Intercellular communication network indicated the intense crosstalk between venous ECs and microglia. Mechanically, elevated Il1b from microglia binds to the Il1r1 of venous ECs in the APP23‐Tg group to stimulate the downstream NF‐κB signaling pathway, leading to ferroptosis of venous ECs.

**Figure:**
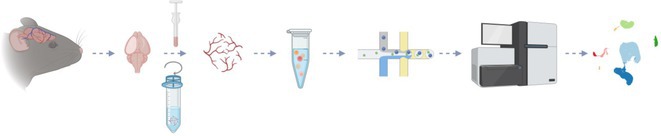
**FIGURE 1** Single cell RNA‐Seq reveals ferroptosis of venous endothelial cells

**Figure:**
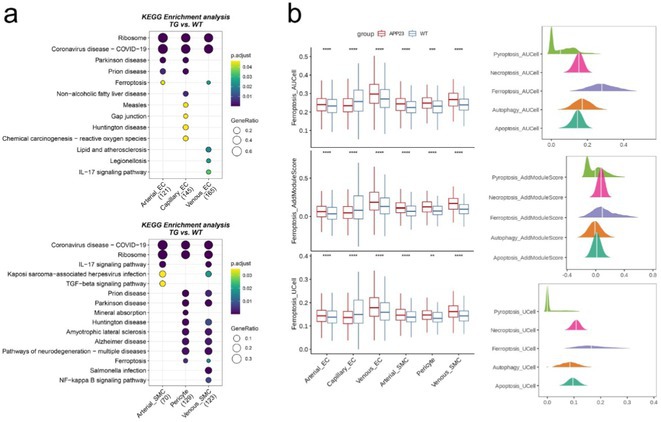
**FIGURE 2** Results

**Conclusion:** Our study discovered the occurrence of ferroptosis in cerebral venous ECs in a CAA animal model, and targeting this process may offer a promising therapeutic strategy.

**Disclosure:** Nothing to disclose.

## EPR‐022

### Pentraxin 3 and stroke: A systematic review and meta‐analysis

#### E. Divina ^1^; R. Permata^1^; P. Prawiroharjo^2^

##### ^1^Faculty of Medicine, University of Indonesia, Jakarta, Indonesia; ^2^Department of Neurology, dr Cipto Mangunkusumo National Hospital, Jakarta, Indonesia

**Background and aims:** Pentraxin 3 (PTX3), a key component of the long pentraxin family, has emerged as a novel biomarker in inflammatory and vascular diseases. Unlike C‐reactive protein (CRP), PTX3 is produced locally at sites of inflammation, making it a more specific indicator of vascular injury and immune response. Recent studies suggest PTX3 plays a critical role in cerebrovascular pathology, particularly in stroke. Elevated PTX3 levels have been associated with worse stroke severity and poor functional outcomes. As a potential predictor of stroke prognosis, PTX3 could provide valuable insights for risk stratification and targeted therapeutic interventions.

**Methods:** A systematic review was conducted using five databases (Pubmed, Proquest, Scopus, Cochrane, and Clinical Key–MEDLINE) and individual searches on 20th January 2025. Keywords include (“Stroke” OR “Cerebrovascular Accident” OR “Cerebrovascular Disorders”) AND (“Pentraxin‐3” OR “PTX3”).

**Results:** After removing duplicates, we obtained 613 articles from databases and individual searches. After undergoing abstract and full‐text screening, this study discussed 9 articles with various results of pentraxin–3. From the combined 7,030 samples, we found a significant mean difference between the PTX–3 level of stroke patients and non‐stroke patients (1,48; 95% CI 1,38–1,58) with an overall effect Z‐score 21.01 (*p*‐value < 0.0001). Pentraxin–3 was also found having a significant effect on mortality rate based on various studies (z score 6.01; 95% CI 5.96–6,06; *p*‐value < 0.001).

**Figure:**
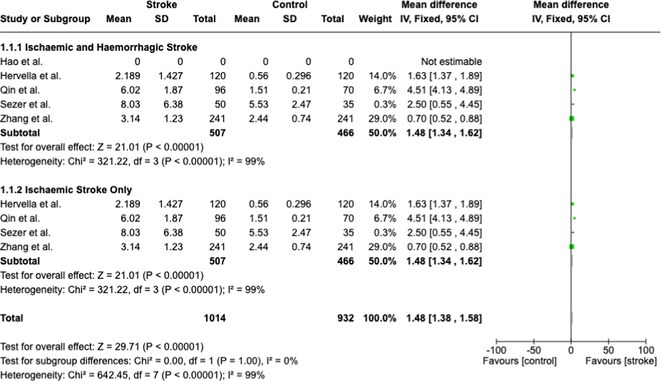
**FIGURE 1** Meta‐analysis of mean differences between PTX–3 of stroke and non‐stroke patients

**Figure:**
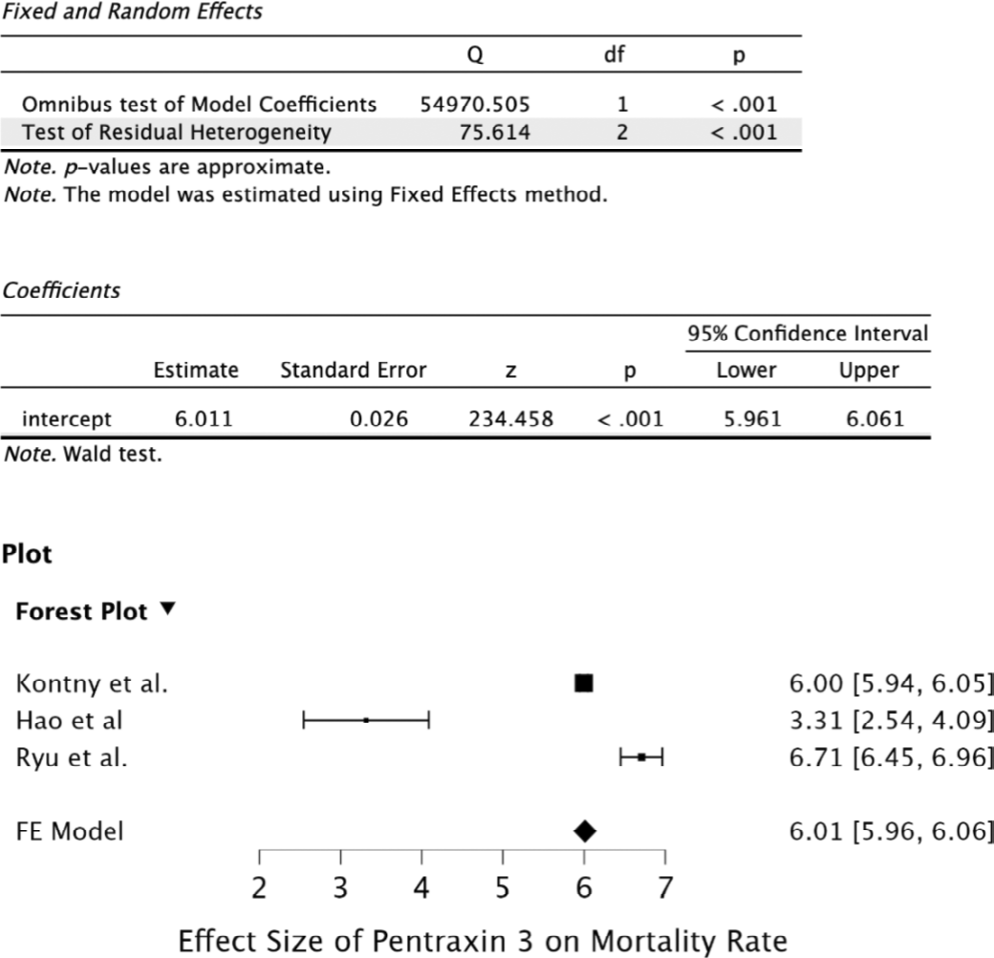
**FIGURE 2** Meta–analysis of PTX–3 effect on mortality rate based on various studies

**Conclusion:** This is the first systematic review and meta‐analysis of pentraxin–3 effect on stroke. Our findings suggest that PTX–3 serves as a novel and reliable predictor for stroke outcome.

**Disclosure:** Nothing to disclose.

## EPR‐023

### Cortical excitability continuum in ALS: The MEP/CMAP ratio as a prognostic and phenotypic stratification marker

#### G. Senerchia ^1^; F. Ranieri^2^; R. Dubbioso^1^

##### ^1^Department of Neurosciences, Reproductive Sciences and Odontostomatology, University of Naples “Federico II”, Naples, Italy; ^2^Unit of Neurology, Department of Neuroscience, Biomedicine and Movement Sciences, University of Verona, Italy

**Background and aims:** Amyotrophic lateral sclerosis (ALS) is a heterogeneous neurodegenerative disease characterized by progressive motor neuron degeneration. Cortical excitability, ranging from hyperexcitability to hypoexcitability, is increasingly recognized as a driver of disease progression and a potential prognostic marker. The motor‐evoked potential (MEP)/compound muscle action potential (CMAP) ratio offers a clinically accessible measure to assess upper and lower motor neuron function. This study aimed to evaluate the clinical utility of the MEP/CMAP ratio in stratifying ALS patients by phenotype, disease stage, and survival.

**Methods:** This multicenter, retrospective study analyzed 743 ALS patients from 16 Italian tertiary referral centers. The MEP/CMAP ratio, recorded from upper limb muscles, was categorized into hyperexcitable, normal, and hypoexcitable states. Patients were classified into classical ALS, bulbar, flail arm/leg, lower motor neuron, pyramidal, and primary lateral sclerosis phenotypes. Disease staging followed the King's clinical system, and survival was analyzed using Kaplan–Meier and Cox proportional hazards models.

**Results:** The MEP/CMAP ratio significantly differed across ALS phenotypes (*p* < 0.0001), with hyperexcitability predominant in LMN, flail, classical, and bulbar ALS, while hypoexcitability was more common in pyramidal and PLS phenotypes. Hypoexcitability increased with advancing disease stages (*p* < 0.0001). Hyperexcitable patients had shorter survival (*p* = 0.003), with a significant difference even within the first year (*p* = 0.006). Cox regression confirmed MEP/CMAP ratio as an independent survival predictor (HR = 1.82, 95% CI: 1.2–2.74, *p* = 0.006).

**Figure:**
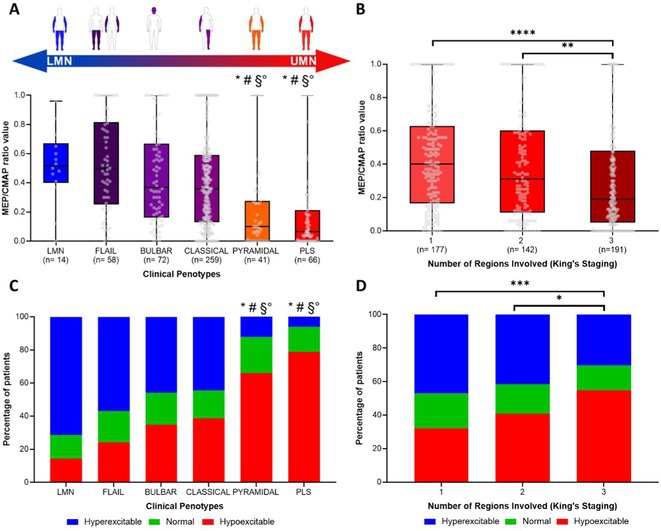
**FIGURE 1** Cortical excitability spectrum across region spreading and phenotypes in ALS

**Figure:**
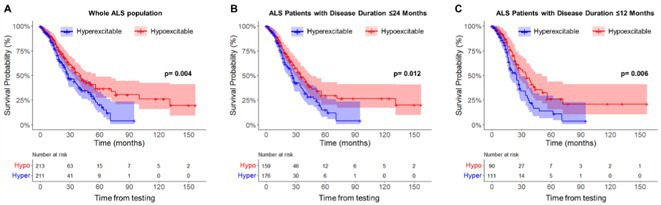
**FIGURE 2** Kaplan–meier survival analysis of ALS patients stratified by cortical excitability

**Conclusion:** The MEP/CMAP ratio is a valuable biomarker for stratifying ALS patients, supporting its integration into routine clinical practice to enhance personalized disease management.

**Disclosure:** Nothing to disclose.

## EPR‐024

### Occludin as potential predictor of outcome in acute ischemic stroke: Preliminary results from the NIMBLE study

#### G. Scrima ^1^; A. Gori^2^; B. Piccardi^3^; A. Sodero^1^; G. Pracucci^1^; L. Tudisco^3^; C. Rapillo^4^; B. Giusti^2^; I. Lombardo^5^; G. Busto^5^; E. Fainardi^6^; V. Palumbo^3^; C. Sarti^1^

##### ^1^NEUROFARBA department, University of Florence, Florence, Italy; ^2^Department of experimental and clinical medicine, University of Florence, Florence, Italy; ^3^Stroke Unit, Careggi University Hospital, Florence, Italy; ^4^Stroke Unit, Humanitas Research Hospital, Milan Italy; ^5^Neuroradiology, Careggi University Hospital, Florence, Italy; ^6^Department of Biomedical, Experimental and Clinical Sciences, University of Florence, Florence, Italy

**Background and aims:** Identifying biomarkers that could predict functional outcomes is crucial in acute ischemic stroke (IS) management. The NIMBLE Study aimed to integrate clinical and preclinical stroke research to identify such biomarkers, both serological and neuroradiological, and their interactions. In this preliminary analysis, we evaluated the association between serological biomarkers and the three‐month functional outcome of acute IS patients.

**Methods:** Monocentric prospective observational study set in Careggi University Hospital enrolling consecutive patients with acute (≤12 hours) anterior circulation IS. Serological biomarkers (pro‐and anti‐inflammatory cytokines and chemokines, metalloproteases and their inhibitors, endothelial dysfunction markers, and tight junction proteins) were obtained at basal and 24 hours. Three‐month mRS > 2 was considered an unfavorable outcome.

**Results:** We enrolled 213 patients, median age was 80 years, 46% women, median baseline NIHSS was 10. Recanalization treatment was administered to 150 patients. Higher presenting basal NIHSS, higher pre‐stroke mRS, atrial fibrillation and higher baseline occludin levels independently predicted 3‐months poor outcome (*p*‐value, OR [95% CI]: *p* = 0.028, 3.28 [1.13 – 9.48]; *p* < 0.001, 1.14 [1.08‐1.20]; *p* < 0.001, 3.23 [2.01‐5.19]; *p* = 0.004, 5.65 [1.74‐18.35], respectively).

**Conclusion:** Our preliminary results show that higher baseline levels of occludin appear to predict clinical outcomes after IS, along with other well‐known clinical prognostic factors. Rapid measurement of occludin could help integrate this biomarker into decision‐making algorithms for recanalization therapies and therapeutic management. Ongoing analyses are evaluating the association between occludin and neuroradiological markers of IS complications, such as hemorrhagic transformation and cerebral edema.

**Disclosure:** Nothing to disclose.

## EPR‐025

### Surgical outcome of cerebral amyloid angiopathy‐related cerebral hemorrhage—A multicenter comparative study

#### K. Chikh ^1^; J. Burel^2^; A. Nikiema^3^; H. Bulteau^4^; D. Maltete^1^; D. Wallon^1^; R. Aboukais^4^; T. Gaberel^3^; D. Stéphane^5^; L. Grangeon^1^

##### ^1^Univ Rouen Normandie, Inserm U1245 and University Hospital of Rouen, Department of Neurology, F‐76000 Rouen, France; ^2^Univ Rouen Normandie, University Hospital of Rouen, Department of Radiology, F‐76000 Rouen, France; ^3^Univ Caen Normandie, Inserm U1237 and University Hospital of Caen, Department of Neurosurgery, F‐14000 Caen, France; ^4^Univ Lille, Inserm U1189 and University Hospital of Lille, Department of Neurosurgery, F‐59000 Lille, France; ^5^Univ Rouen Normandie, Inserm U1245 and University Hospital of Rouen, Department of Neurosurgery, F‐76000 Rouen, France

**Background and aims:** Neurosurgeons are reluctant to perform surgery for lobar intracerebral hemorrhages (ICH) associated to cerebral amyloid angiopathy (CAA) due to a suspected high risk of postoperative rebleeding. Diagnosis of CAA is increasing with an aging population and external Edinburgh criteria validation on computed tomography (CT) scan. We assessed the postoperative risk of CAA‐ICH compared to non‐related CAA‐ICH.

**Methods:** We included patients admitted between 2008 and 2022 for spontaneous lobar ICH who underwent surgery at three university hospitals. A single‐blinded neuroradiologist analyzed the Edinburgh criteria on the initial CT scan before surgery and assessed rebleeding on a repeat CT scan performed within 48 hours after surgery. Patients were classified into the “CAA group” according to the Edinburgh or Boston criteria, and into the “non‐CAA group” if they had another cause of ICH.

**Figure:**
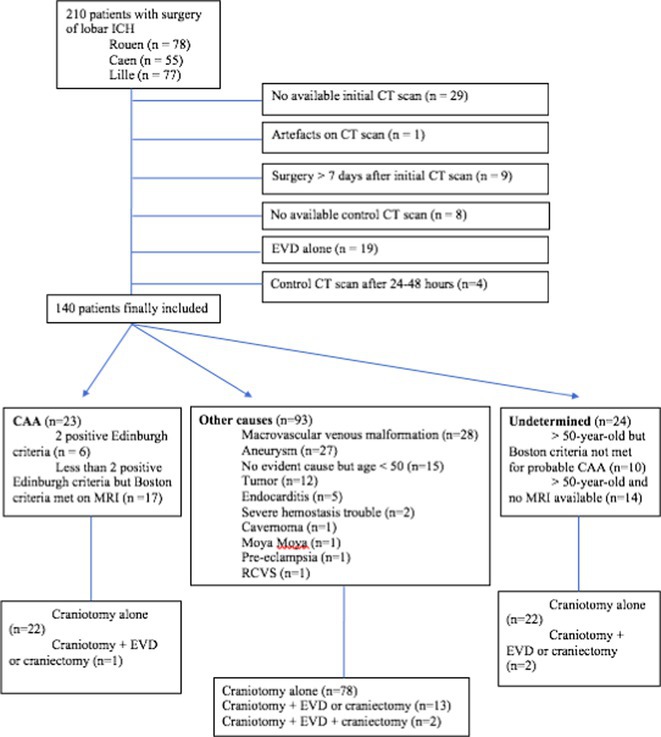
**FIGURE 1** Flowchart

**Results:** A total of 140 patients were included, with 23 in the CAA group, 93 in the non‐CAA group, and 24 in the undetermined group. The postoperative rebleeding rate at 24‐48 hours did not differ significantly between groups (13% in the CAA group vs. 15% in the non‐CAA group, *p* > 0.99). The overall rate of rebleeding associated with clinical deterioration did not differ between groups (9% in the CAA group vs. 6% in the non‐CAA group, *p* = 0.66).

**Figure:**
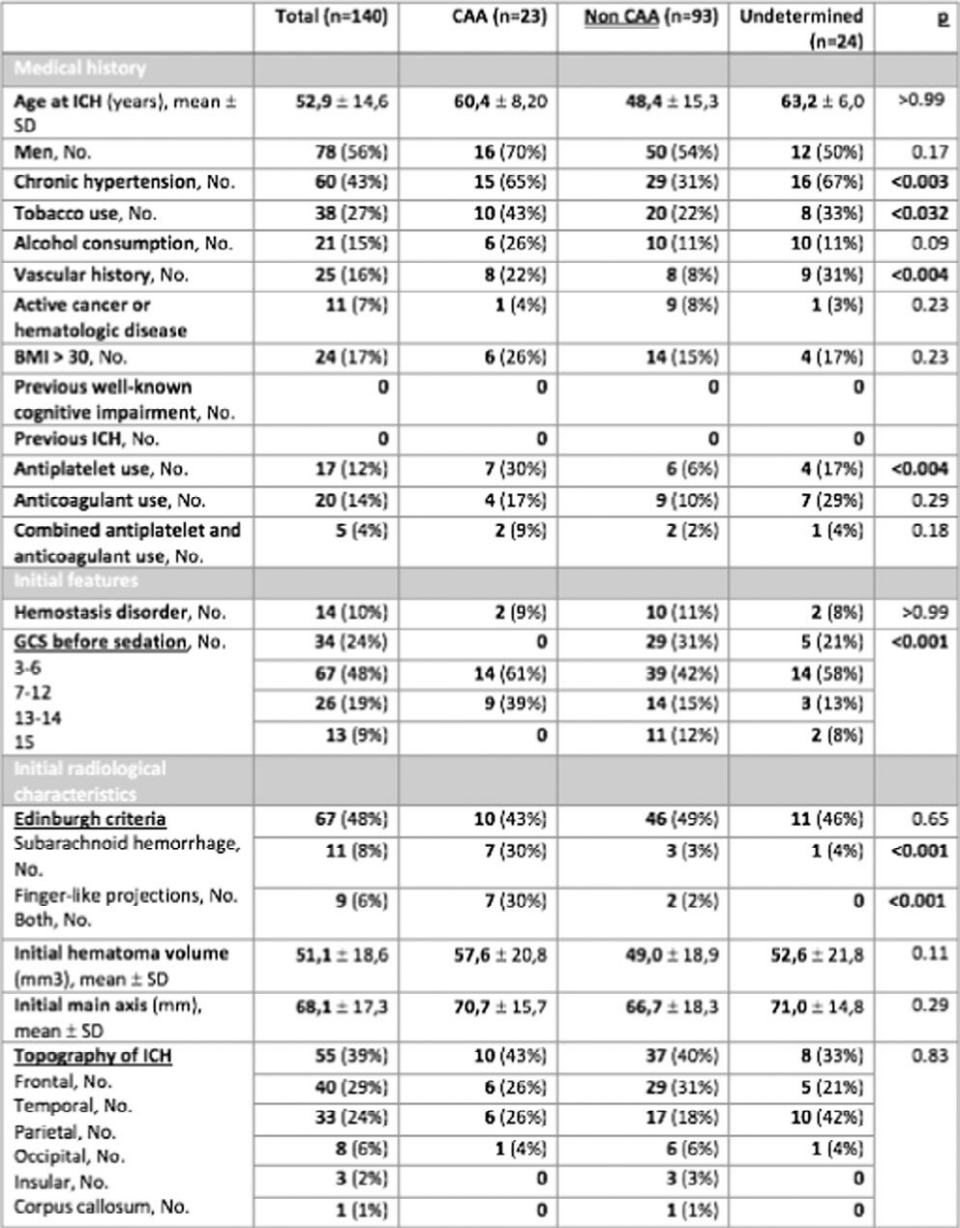
**TABLE 1** Demographics and pre‐operative information in the three groups.

**Figure:**
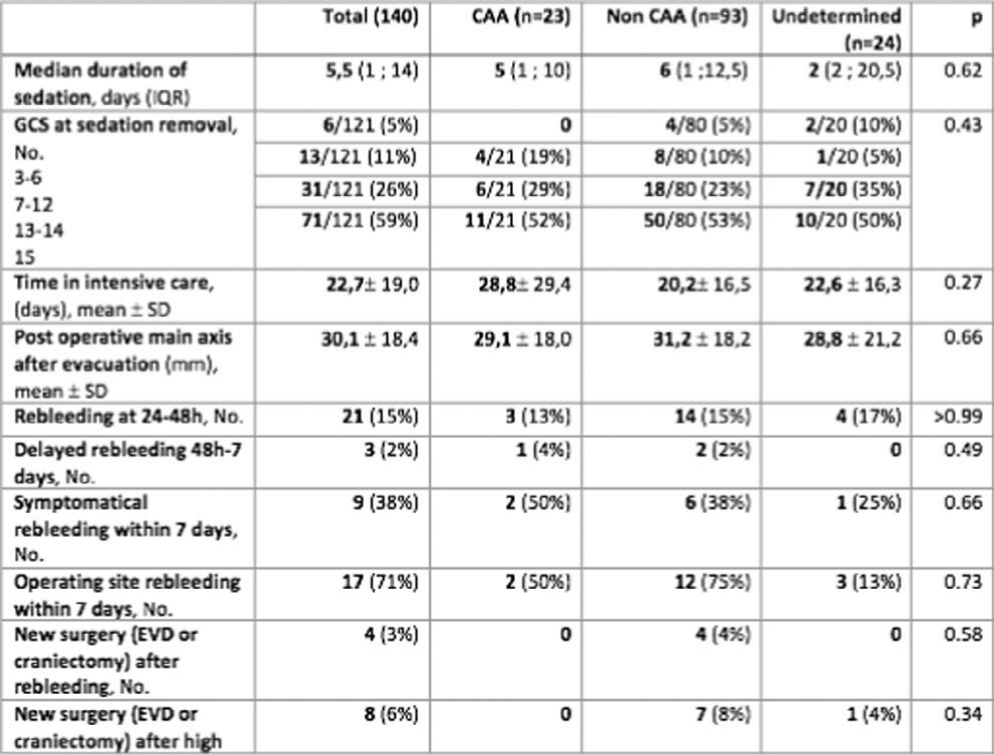
**TABLE 2** Postoperative outcome in the three groups

**Conclusion:** We did not find a significant difference in the postoperative rebleeding rate after ICH associated with CAA compared to other causes.

**Disclosure:** Nothing to disclose.

## EPR‐026

### Changes in management of intracerebral hemorrhage over 7 years in a population‐based stroke registry

#### P. Colantuono ^1^; L. D'Anna^2^; M. Foschi^1^; M. Adipietro^1^; S. Lancia^1^; L. Mammarella^4^; S. Sacco^1^; R. Ornello^1^

##### ^1^Department of Biotechnological and Applied Clinical Sciences – University of L’Aquila, Via Vetoio 1, L’Aquila, Italy; ^2^Department of Stroke and Neuroscience, Charing Cross Hospital, Imperial College London NHS Healthcare Trust, London, UK; ^4^Servizio Flussi Informativi e Statistica Sanitaria, Azienda Sanitaria Locale Avezzano‐Sulmona‐L’Aquila, L’Aquila, Italy

**Background and aims:** Comprehensive care bundles including rapid blood pressure management, anticoagulation reversal, neurosurgical consultation, control of blood glucose and body temperature, can improve short‐ and medium‐term outcomes in patients with intracerebral hemorrhage (ICH). This study assessed how the acute management of ICH practices evolved in a real‐world setting over seven years characterized by global changes in ICH care.

**Methods:** This study analyzed key clinical parameters of ICH management—blood pressure, blood glucose, body temperature—from a population‐based stroke registry (2018‐2024) which were classified into three periods: 2018‐2019, 2020‐2022, and 2023‐2024, reflecting the evolution of the “bundle of care” approach to ICH.

**Results:** We included 545 patients with ICH (55.4% male, median age 75.4 years, interquartile range 69‐85). After 24 hours from ICH, the proportion of patients with blood pressure control (systolic blood pressure < 140 mg/dl) improved from 35.0% in the 2018‐2019 period, to 36.7% in the 2020‐2022 period, and to 48.8% in the 2023‐2024 period (*p* = 0.069); the proportion of patients with blood glucose control ( < 108 mg/dl) after 24 hours from ICH increased from 19.9%, to 23%, to 37.8% (*p* < 0.001); the proportion of patients with normal body temperature ( < 37.0*C) increased from 53.9%, to 61.3%, to 80.5% (*p* < 0.001). Those changes had an impact on 30‐day survival after ICH which changed from 65.5% in the 2018‐2019, to 64% in the 2020‐2022, to 78% in the 2023‐2024 period (*p* = 0.045).

**Figure:**
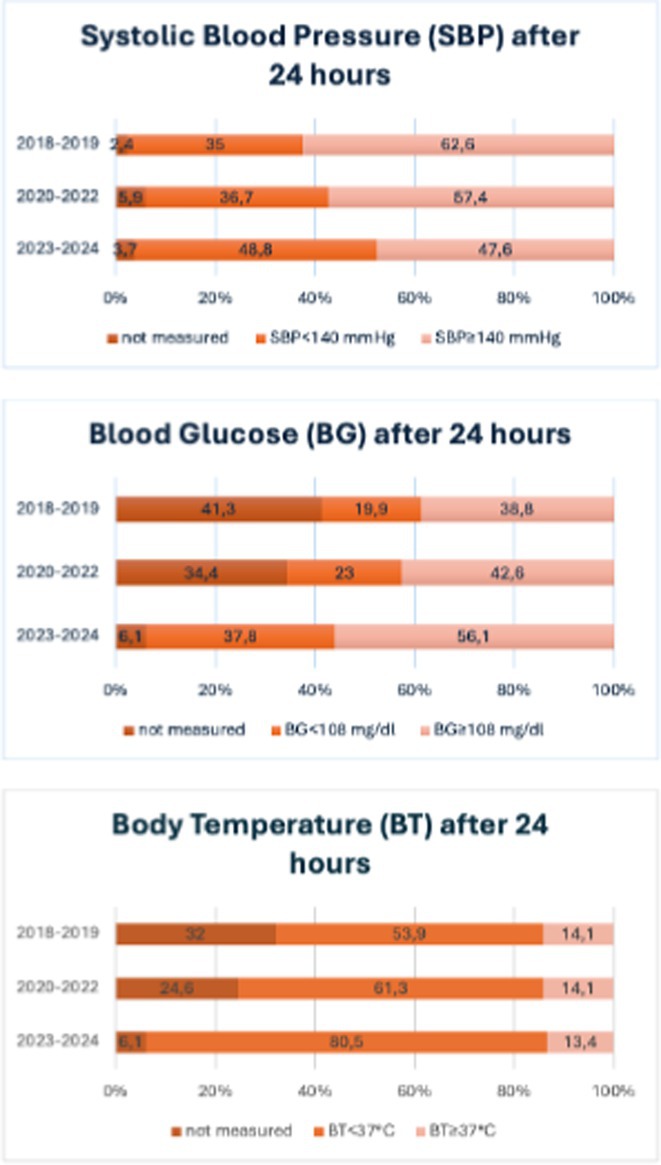
**FIGURE 1** Parameters of intracerebral hemorrhage management at 24 hours from symptom onset during the three study periods (2018‐2019, 2020‐2022, 2023‐2024)

**Figure:**
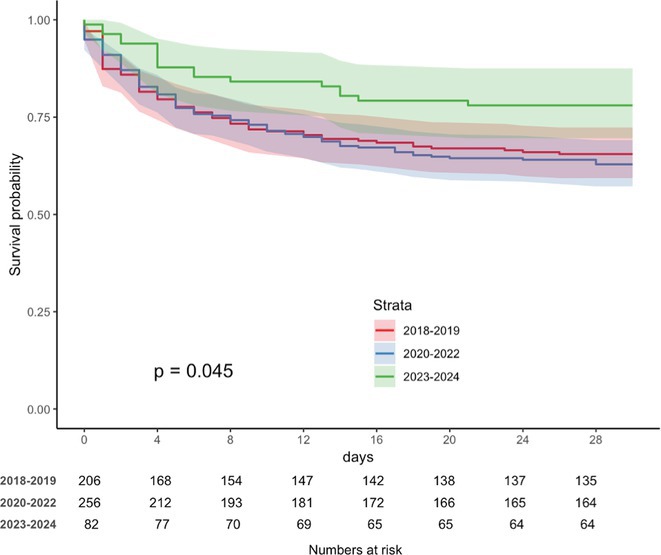
**FIGURE 2** Thirty‐day case fatality rates during the three study periods (2018‐2019, 2020‐2022, 2023‐2024)

**Conclusion:** This real‐world study demonstrates improvements over time in parameters of acute ICH management with consequent improvements in early prognosis.

**Disclosure:** Nothing to disclose.

## EPR‐027

### Clinical and neuroimaging implications of anterior temporal pole white matter hyperintensity in CADASIL

#### W. Ou Yang ^1^; Y. Liao^1^; S. Hsu^2^; Y. Lee^1^

##### ^1^Department of Neurology, Taipei Veterans General Hospital, Taipei, Taiwan; ^2^Department of Neurology, Fu Jen Catholic University Hospital, New Taipei City, Taiwan

**Background and aims:** White matter hyperintensity (WMH) in anterior temporal pole is the hallmark feature of cerebral autosomal dominant arteriopathy with subcortical infarcts and leukoencephalopathy (CADASIL). This study investigates clinical and radiographic differences in CADASIL patients with and without anterior temporal pole WMH.

**Methods:** This retrospective cross‐sectional study included 518 genetically confirmed CADASIL patients from Taipei Veterans General Hospital. Clinical characteristics, cerebral microbleeds (CMBs), lacunes, and WMH volumes were analyzed. WMH burden was assessed using WMH/intracranial volume (WMH/ICV) ratios. Statistical analyses included chi‐squared and Student's t‐tests.

**Results:** Among the 518 patients, epidermal growth factor‐like repeats (EGFr) 1–6 mutations were more frequently associated with anterior temporal pole WMH than EGFr 7–34 (*p* = 2.6× 10^10). Patients with anterior temporal pole WMH had significant higher prevalence of stroke, cognitive decline, and headaches (*p* < 0.05). Additionally, patients with anterior temporal pole WMH were more likely to have worse disability (modified Rankin Scale > = 3, *p* = 0.02), more lacunes (*p* = 5.1x10^ ‐8), higher Fazekas scores at periventricular regions and deep white matter (*p* = 1.4x10^ ‐21 and 7.9x10^ ‐20), and greater WMH/ICV ratios (*p* = 1.7x10^ ‐23). The same analyses were conducted in 429 patients with p.R544C, the most common mutation in Taiwan. It revealed consistent trends and significant differences between patients with or without anterior temporal pole WMH.

**Figure:**
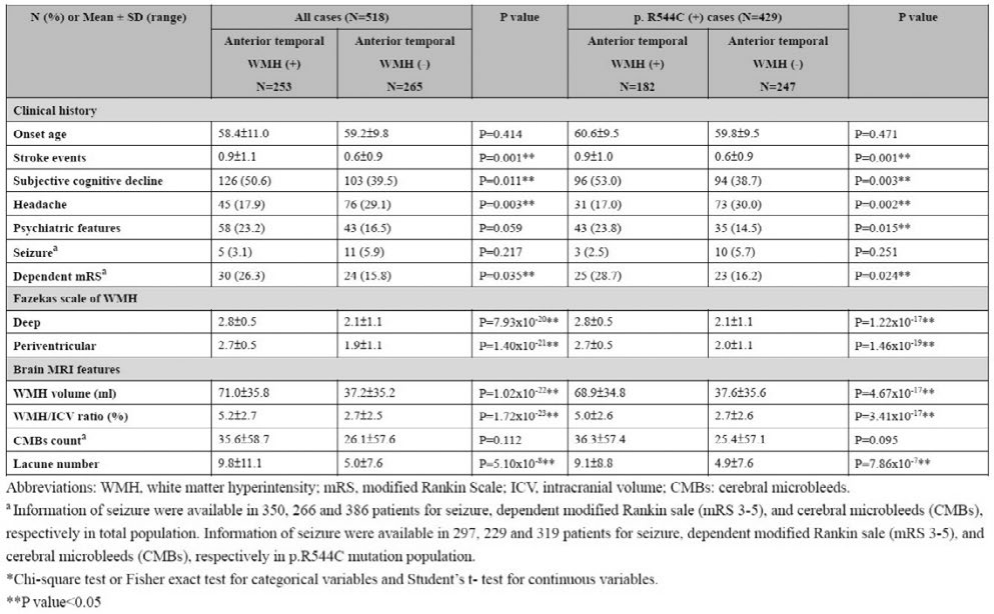
**TABLE 1** Clinical and neuroimaging features of CADASIL patients.

**Conclusion:** Anterior temporal pole WMH is significantly associated with higher burden of MRI markers of small vessel disease and worse clinical outcomes in CADASIL patients. Further studies are needed to assess its long‐term impact.

**Disclosure:** None.

## Pain

## EPR‐028

### The role of dopamine in peripheral mechanisms of migraine: insight from DAT‐HET rat model

#### S. Svitko^1^; K. Shaidullova^1^; A. Yakubova ^2^; G. Sitdikova^3^

##### ^1^Department of Animal and Human Physiology, Kazan Federal University, Kazan, Russia; ^2^Openlab “Gene and Cell Technologies”, Kazan Federal University, Kazan, Russia; ^3^Sirius University of Science and Technology, Sochi, Russia

**Background and aims:** Dopamine is considered to play role in the pain transmission in the central nervous system. However, the role of dopamine in regulation of trigeminal‐vascular system, a source of migraine‐related pain signals, has not been investigated. The aim of study was to evaluate the nociceptive activity of meningeal afferents and the effects of classic algogen serotonin in DAT‐HET rats with decreased expression of dopamine transporter (heterozygous rats from DAT‐KO).

**Methods:** Electrophysiological recordings of action potentials (APs) from V1 branch of trigeminal nerve after serotonin (20 μM) application were conducted using isolated half‐skull rat preparation with intact dura mater (male rats, P 40‐45, wild type (WT) and DAT‐HET groups).

**Results:** In WT group, serotonin increases AP frequency from 275 ± 53 to 486 ± 114 APs (*p* = 0.004, *n* = 12), (Fig. 1A) after 5 minutes and to 711 ± 153 APs after 20 minutes of application (*p* = 0.002, *n* = 12). Peak AP value of serotonin effect was 762 ± 156 APs (*n* = 12, *p* = 0.002), (Fig. 1B). In DAT‐HET group, the baseline AP frequency was higher compared to control group and serotonin increased AP frequency from 624 ± 130 to 750 ± 149 APs (*n* = 6) after 5 minutes and to 1228 ± 575 APs after 20 minutes (*n* = 6). The peak value of AP during serotonin application was 1765 ± 527 APs per 5 minutes (*n* = 6, *p* = 0.036).

**Figure:**
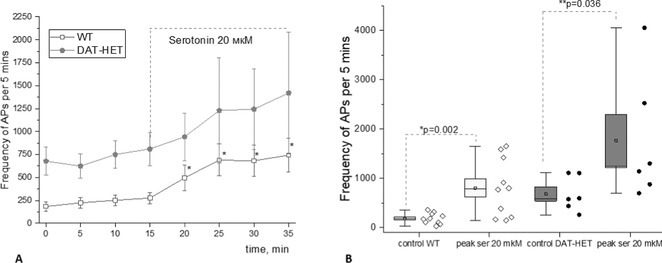
**FIGURE 1** Effects of serotonin on AP frequency in trigeminal afferents of WT and DAT‐HET rats: A – Dynamics of AP frequency before and during serotonin application; B – Peak frequency of APs after serotonin application compared to baseline control (*p* < 0.05).

**Conclusion:** Thus, DAT‐HET rats demonstrated higher rate of AP generation, which proposes higher excitability of nociceptive afferents. Serotonin demonstrated pro‐nociceptive effects in both control and DAT‐HET groups.

**Disclosure:** This study was supported by Russian Science Foundation #23‐15‐00328.

## EPR‐029

### Capsaicin 8% completely blocks activation of polymodal nociceptors by heat, but only marginally by slow depolarization

#### D. Litewczuk ^1^; S. Soares^2^; A. Truini^1^; M. Schmelz^2^; R. Rukwied^2^

##### ^1^Department of Human Neuroscience, Sapienza University of Rome, Rome, Italy; ^2^Experimental Pain Research, Mannheim Center for Translational Neuroscience, Heidelberg University, Heidelberg, Germany

**Background and aims:** This study aimed to evaluate (i) the effect of topical capsaicin on mechanical activation of polymodal nociceptors; (ii) sensitization of C‐nociceptors outside the capsaicin‐denervated skin area; (iii) the differential temporal recovery of capsaicin‐induced sensory impairments.

**Methods:** Two 8% capsaicin patches were applied to the volar forearm skin of 15 healthy human volunteers and renewed for 4 consecutive days. Subjects were assessed weekly over 84 days for pain NRS (0‐10) and axon reflex flare (Moor LDI) after electrical, thermal and mechanical impact stimulation, within and just outside the capsaicin treated skin sites. Single electrical sinusoidal (0.5 sec, 1 Hz) and continuous 4 Hz sinusoidal stimuli (2.5 and 60 sec) were used to activate polymodal and silent C‐nociceptors.

**Results:** Capsaicin abolished heat pain and axon reflex flare responses, partially blocked pain by slow depolarizing stimuli, but had only negligible effects on mechanical impact pain. Sinusoidal pain and in particular flare responses showed a slow temporal recovery during 84 days. Acute secondary punctate hyperalgesia was reported during the initial application phase of capsaicin but lasting hypersensitivity around the application sites was not found.

**Conclusion:** We did not evidence for increased axonal transport into non‐treated branches of nociceptors that could sensitize the skin surrounding the capsaicin application site. However, our results suggest that there are differential back‐up mechanisms for transduction of heat, slow depolarizing electrical stimuli and mechanical impact stimuli in polymodal nociceptors. The specificity of evoked pain tests to assess nociceptor excitability may provide clinically major implications.

**Disclosure:** Nothing to disclose.

## EPR‐030

### Gabapentinoids use and abuse in the neuropathic pain unit setting

#### E. Evangelisti; G. Di Pietro; D. Litewczuk; P. Falco; N. Esposito; E. Galosi; G. De Stefano; G. Di Stefano; C. Leone; A. Truini

##### Department of Human Neuroscience, Sapienza University of Rome, Rome, Italy

**Background and aims:** The gabapentinoids (GBPs) pregabalin and gabapentin are increasingly prescribed for various clinical conditions. However, concerns about their potential for misuse and abuse have emerged in recent years. Given their approved and off‐label uses, it is essential to identify patients at risk for such issues.

**Methods:** In this ongoing observational study, we assess the efficacy and safety of GBPs in a Neuropathic Pain Unit. Patients referred to the Department of Human Neuroscience at Sapienza University of Rome are being recruited and evaluated using a structured questionnaire addressing the main aspects of the GBPs treatment: anamnestic information, comorbidities, the pain condition related to the GBP prescription, S‐DN4, treatment information, adverse event, efficacy, and use disorder.

**Results:** As of now, 119 patients have been enrolled (median age 61, IQR 52‐72; 41 males, 77 females). Most patients were diagnosed with peripheral neuropathy (50%), fibromyalgia (19%), or radiculopathy (16%). Of these, 93 were prescribed pregabalin and 26 gabapentin. Seventy‐eight patients (57%) reported adverse events, mainly somnolence (47%), confusion (29%), and dizziness (22%), though 89% did not discontinue treatment due to these effects. Patients' pain relief ratings were: much improved (35.6%), minimally improved (26.3%), or unchanged (22.9%). Notably, 9.2% of patients showed signs of GBPs use disorder, and 13.4% reported taking the medication differently than prescribed.

**Conclusion:** Although patient recruitment is ongoing and final data will be presented at the congress, these early findings suggest that the risk of misuse and developing use disorder should be considered when prescribing GBPs in a neuropathic pain setting

**Disclosure:** Nothing to disclose.

## EPR‐031

### Spinal cord stimulation for painful diabetic neuropathy: A systematic review and meta‐analysis

#### H. Atwan

##### Faculty of Medicine, Assiut University, Assiut, Egypt

**Background and aims:** Painful diabetic neuropathy (PDN) is a debilitating diabetes complication that severely impacts quality of life. Conventional treatments often provide inadequate relief. Spinal cord stimulation (SCS) has emerged as a promising option, but its efficacy and safety require further evaluation.

**Methods:** We conducted a systematic review and meta‐analysis of randomized controlled trials (RCTs) and prospective observational studies on SCS for PDN. A comprehensive database search identified 19 studies: 3 RCTs with follow‐up studies (312 patients) and 13 observational studies (245 patients). Outcomes included pain reduction, quality‐of‐life improvements, and adverse events.

**Results:** Meta‐analysis of RCTs revealed a significant reduction in pain with SCS compared to conventional medical management (CMM) (*p* < 0.00001; MD: ‐5.29, 95% CI: ‐5.84 to ‐4.73). At 6 months, 72.5% of SCS‐treated patients achieved ≥50% pain relief, compared to only 4.7% in the CMM group (*p* < 0.00001; RR: 14.86, 95% CI: 6.98–31.63). SCS also led to a significant 14.13% improvement in EQ‐5D utility scores (*p* < 0.00001) and enhanced patient‐reported outcomes. Both RCTs and observational studies showed a 50.99% reduction in pain from baseline at 6 months (*p* < 0.00001; MD: 50.99, 95% CI: 47.55–54.44), with 72.4% of SCS‐treated patients achieving ≥50% pain relief at 6 months and 57.7% maintaining this response at 12 months.

**Figure:**
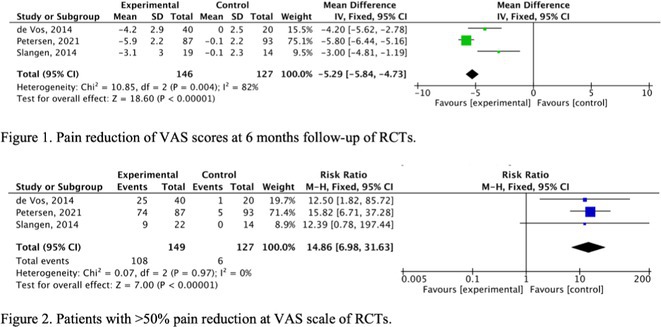
**FIGURE 1** Pain reduction.

**Figure:**
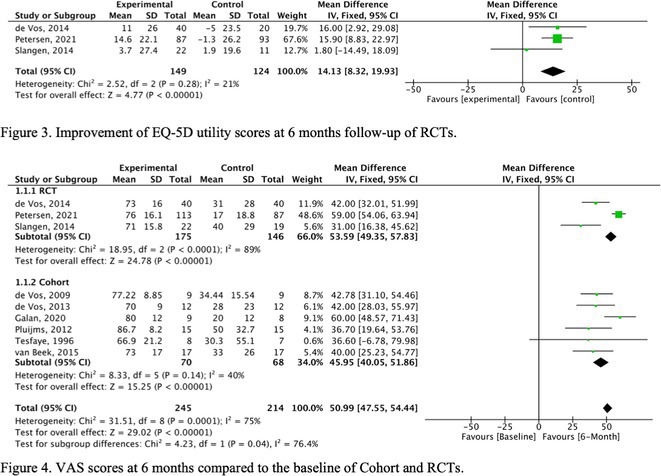
**FIGURE 2** Pain reduction and quality of life improvement.

**Conclusion:** SCS offers significant pain relief and quality‐of‐life improvements in PDN. While observational studies suggest durable benefits, large‐scale RCTs with extended follow‐up are needed to establish SCS as a standard treatment.

**Disclosure:** Nothing to disclose.

## EPR‐032

### The periaqueductal gray density, glymphatic dysfunction, emotional and sleep disorders in patients with chronic pain

#### E. Bоsiakova ^1^; O. Alenikova^1^; M. Dymkovskaya^1^; O. Zmachynskaya^1^; A. Zelenko^2^; S. Tolkach^2^; E. Mikitchuk^3^

##### ^1^Republican Research and Clinical Center of Neurology and Neurosurgery; ^2^Republican Center for Hygiene, Epidemiology and Public Health; ^3^Belarusian State University

**Background and aims:** Chronic pain (CP) is a complex process that is influenced by sleep and emotional disorders, and vice versa. Sleep disturbances and CP negatively affect brain homeostasis by reducing glymphatic clearance. Additionally, CP reorganizes the brain structures involved in emotional state and pain modulation. The periaqueductal gray (PAG) plays an important role in anxiety, depression and sleep disorders, as well as brain waste clearance due to its high aquaporin‐4 expression.

**Methods:** 28 patients with CP (CPgroup) and 20 age‐match controls were examined by using various questionnaires, polysomnography and neuroimaging studies (table 1).

**Figure:**
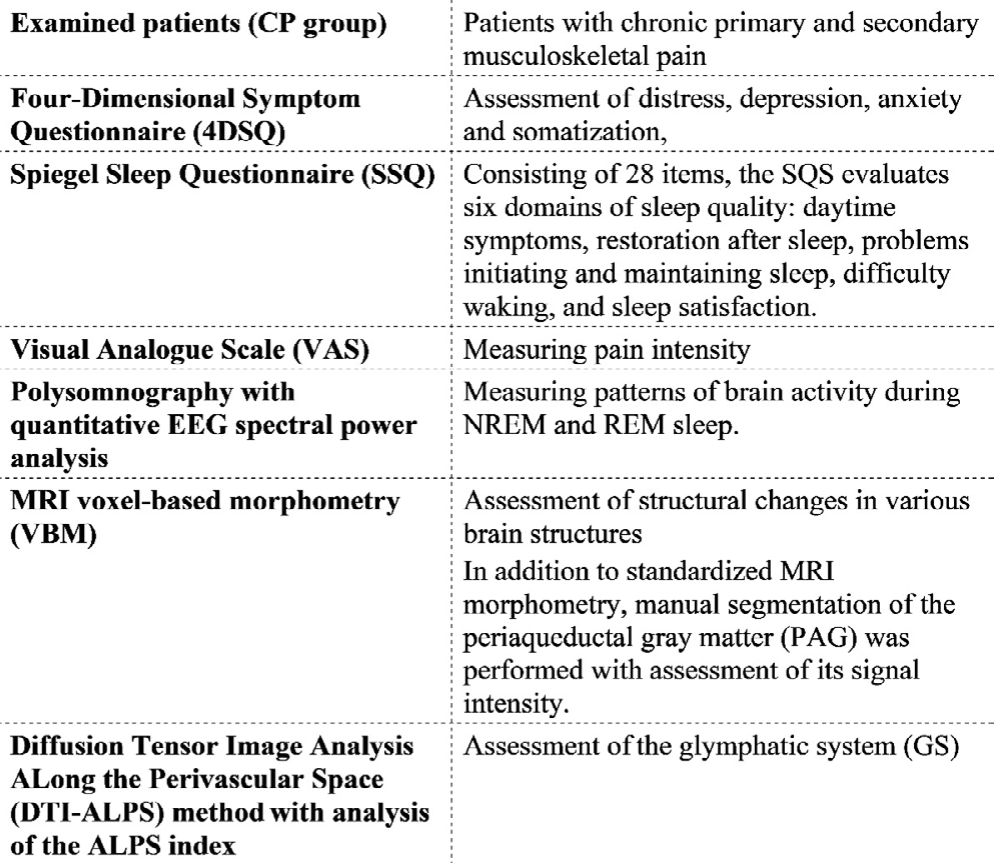
**TABLE 1** Research methods.

**Results:** CPgroup had a higher score on the 4DSQtotal as well as on distress, depression, anxiety and somatization subscales compared to controls (table 2). Both objective and subjective indicators of sleep disturbance were worse in the CPgroup. Slow‐wave activity significantly declined especially over frontal regions during NREM with an increase in α‐band power over the parietal regions during REM sleep in CPgroup (figure 2). Neuroimaging revealed an increase in PAG density and a decrease in the ALPS index in CPpatients compared to controls. PAG density had positive correlation with 4DSQ and VAS and negative correlation with the ALPS index and sleep disturbance indicators (figure 3).

**Figure:**
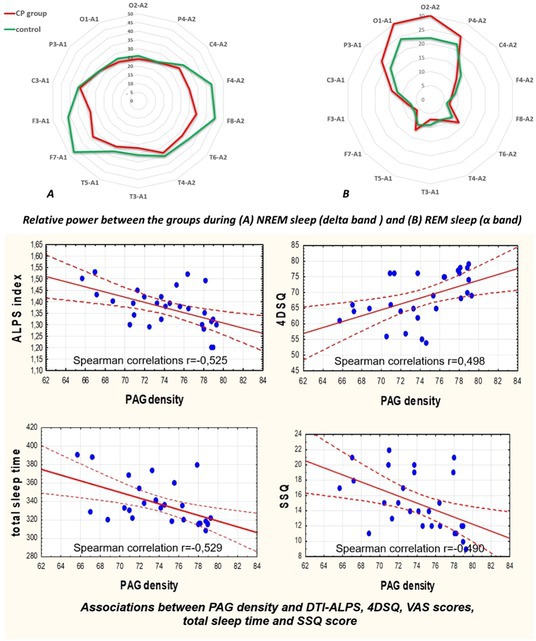
**FIGURE 1** Graphical representation of the obtained results

**Figure:**
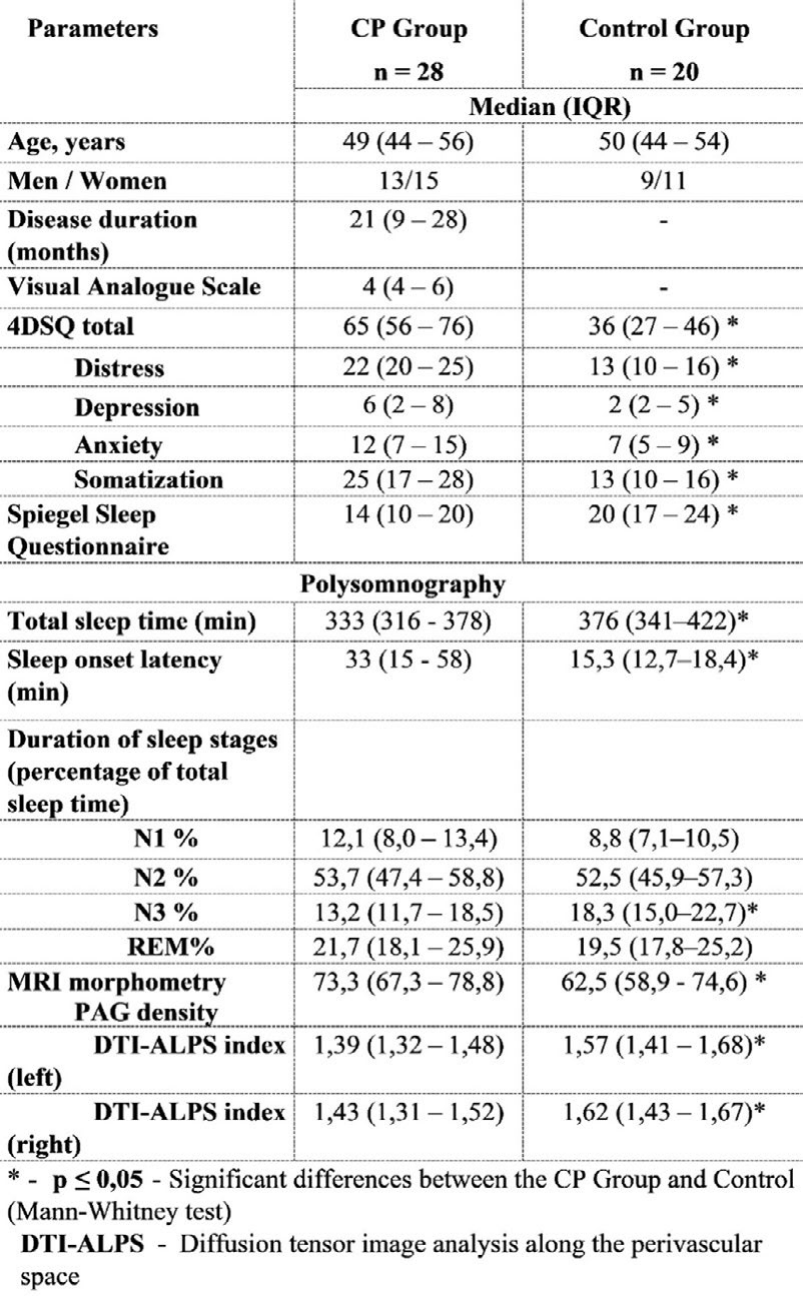
**TABLE 2** Comparative assessment of examined patients with chronic pain and controls.

**Conclusion:** The relationship between PAG density and emotional changes, pain severity and sleep parameters, as well as glymphatic clearance, indicates that PAG participates in pain chronicity. Therefore, targeting PAG can be considered one of the promising methods for CP treating.

**Disclosure:** The authors declare that they have no conflict of interest.

## EPR‐034

### Distinguishing small fiber neuropathy and fibromyalgia‐related small fiber pathology: Insights from SKIN BIOPSY Analysis

#### P. Falco; E. Galosi; G. Di Pietro; G. De Stefano; D. Litewczuk; E. Evangelisti; G. Di Stefano; A. Truini

##### Department of Human Neuroscience, Sapienza University, Rome, Italy

**Background and aims:** Small fiber neuropathy and fibromyalgia syndrome frequently share overlapping clinical features, including reduced intraepidermal nerve fiber density, as assessed by skin biopsy. Clinical observations suggest that the reduction in intraepidermal nerve fiber density is generally more severe in small fiber neuropathy than in fibromyalgia, which is commonly classified as a small fiber pathology. This study aimed to compare the severity of intraepidermal nerve fiber density reduction in patients with small fiber neuropathy and those with fibromyalgia‐related small fiber pathology, and to evaluate whether the difference could aid in distinguishing between the two conditions.

**Methods:** We retrospectively analyzed data from 132 patients diagnosed with small fiber neuropathy and 180 patients diagnosed with fibromyalgia, including 73 with diagnosed intraepidermal nerve fiber density reduction. The severity of intraepidermal nerve fiber density reduction was compared between the 132 patients with small fiber neuropathy and the 73 with fibromyalgia‐related small fiber pathology.

**Results:** We found that the reduction in intraepidermal nerve fiber density was significantly more severe in patients with small fiber neuropathy compared to those with diagnosed fibromyalgia‐related small fiber pathology. ROC analysis showed that a reduction exceeding 38% relative to normative ranges, while having relatively low sensitivity (48%), had high specificity (94%) and positive predictive value (94%) for identifying small fiber neuropathy.

**Figure:**
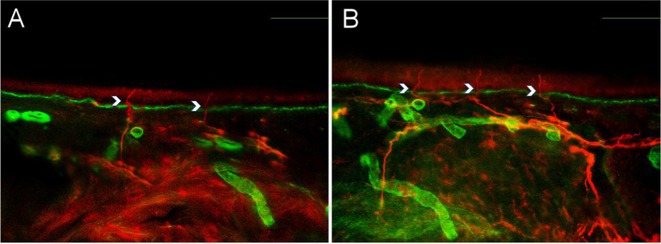
**FIGURE 1** Intraepidermal nerve fiber density in small fiber neuropathy and small fiber pathology.

**Figure:**
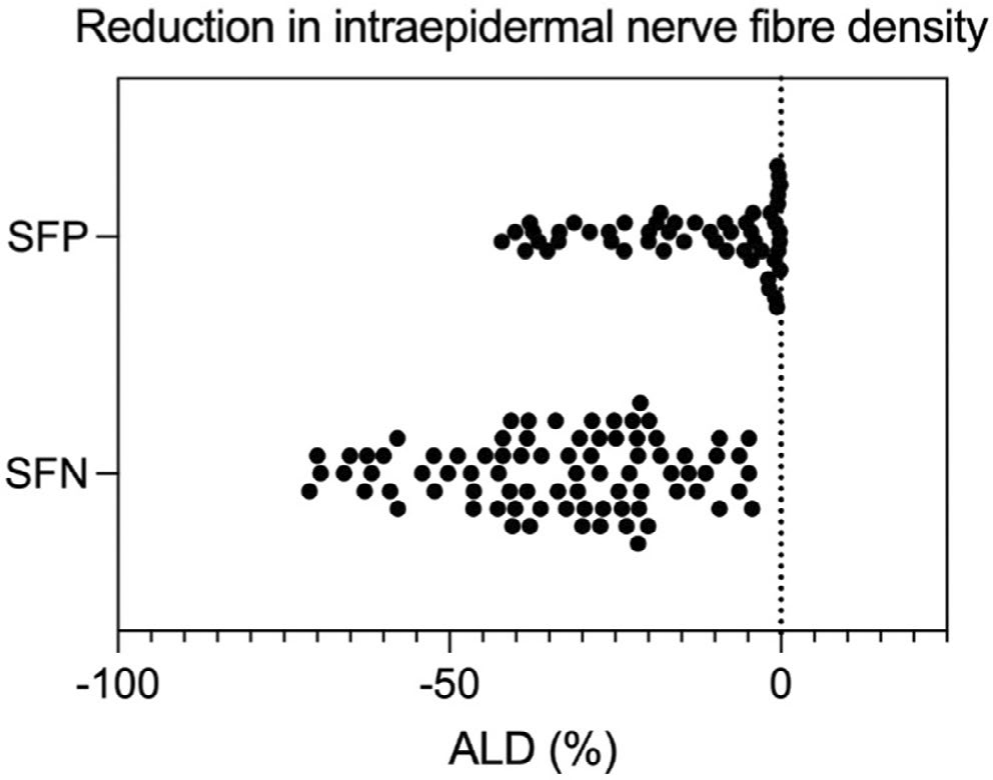
**FIGURE 2** Reduction in intraepidermal nerve fiber density in patients with fibromyalgia and small fiber neuropathy.

**Figure:**
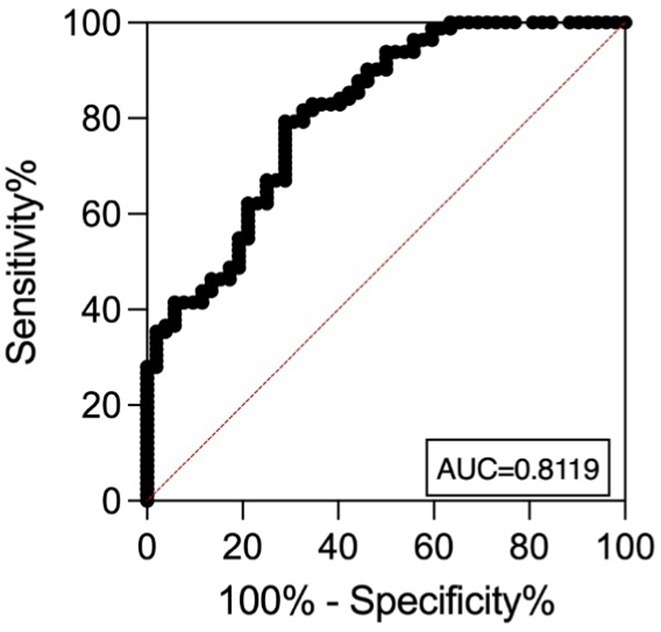
**FIGURE 3** ROC curve for the accuracy of intraepidermal nerve fiber density reduction for distinguishing fibromyalgia‐related small fiber pathology and small fiber neuropathy.

**Conclusion:** Our study demonstrated that intraepidermal nerve fiber density reduction is more severe in small fiber neuropathy than in fibromyalgia syndrome. A reduction exceeding 38% relative to normative ranges is a reliable indicator of small fiber neuropathy.

**Disclosure:** Nothing to disclose.

## Headache 1

## EPR‐036

### Home‐based biofeedback therapy for migraine prevention in episodic migraine: A randomized, waitlist‐controlled trial

#### A. Poole ^1^; A. Stubberud^1^; M. Simpson^2^; L. Øie^1^; M. Bjørk^1^; E. Kristoffersen^1^; K. Vetvik^1^; A. Olsen^1^; I. Larsen^1^; M. Linde^1^; E. Tronvik^1^; T. Wergeland^1^

##### ^1^Norwegian Centre for Headache Research (NorHead), Norwegian University of Science and Technology, Trondheim, Norway; ^2^Department of Public Health and Nursing, Faculty of Medicine and Health Sciences, Norwegian University of Science and Technology, Trondheim, Norway

**Background and aims:** This study evaluated the efficacy and safety of a new medical device for therapist‐independent biofeedback for preventing episodic migraine in adults.

**Methods:** This was a nation‐wide, open‐label, randomized, waitlist‐controlled multicenter trial in Norway. Participants were randomly assigned (1:1) to daily biofeedback sessions via the “Cerebri” medical device or waitlist control. The primary endpoint was the change in the mean number of migraine days from baseline to the last 28 days during the 12‐week treatment phase. The primary analysis was conducted on the intent‐to‐treat population. ClinicalTrials.gov (NCT05616741).

**Results:** 279 were randomized to receive Cerebri treatment (*n* = 140) or control (*n* = 139). Mean age (female percentage) were 41.9 years (92.9%) in the biofeedback group, and 40.6 years (91.4%) in the control group. Baseline migraine days averaged 4.8 in the biofeedback group and 4.6 in the control group. At Weeks 9–12, the Cerebri group had a reduction of 0.9 days (95% CI ‐1.4 to ‐0.5), with no significant change in the control group (95% CI ‐0.5 to 0.4). The between‐group difference was ‐0.9 days (95% CI ‐1.5 to ‐0.3, *p* = 0.002). 30% responder rates were 44.3% for Cerebri versus 29.9% for control (OR 1.86, *p* = 0.033). Among 140 participants, there were 139 treatment‐emergent adverse events (AEs): 132 unrelated, 5 possibly related, and 2 causally related (pruritus, lightheadedness). Two unrelated serious AEs occurred (appendicitis, menorrhagia).

**Conclusion:** The “Cerebri” biofeedback treatment is a safe and effective non‐pharmacological preventive treatment for adults with episodic migraine.

**Disclosure:** The Sponsor of the study is Nordic Brain Tech AS (contact information: info@nordicbraintech.com), a spin‐off company from The Norwegian University of Science and Technology (NTNU) and St. Olavs Hospital in Norway. The company was founded in 2019 to commercialize a research‐based intervention for treating migraines using remote biofeedback. The sponsor has no role in data collection, analysis, and interpretation of data and has no authority pertaining to publication of results. The coordinating investigator has access to the final trial data set. No contractual agreement limits this access. Data management according to the data management plan is conducted by the sponsor. Nordic Brain Tech AS, NTNU, and The Central Norway Regional Health Authority may benefit financially from the commercialization of the proposed treatment. A commercial license agreement exists between Nordic Brain Tech AS and NTNU Technology Transfer AS on the commercialization of the treatment. EAT, AO, AS are cofounders and shareholders of Nordic Brain Tech AS. EAT, AO, AS and ML hold a patent related to the Cerebri invention described in this study. In addition, EAT, AO, AS, and ML are coinventors of the proposed treatment in this study and may benefit financially from a license agreement between Nordic Brain Tech AS and NTNU. ACP, ESK, MHB, LRØ, ICKL, KGV and TWM have no ownership interest and does not own stocks of Nordic Brain tech AS or other companies developing biofeedback treatment.

## EPR‐037

### BIOmarkers of MIGraine response to erenumab (BIOMIGA project): Preliminary assessment of the structural MRI study

#### D. Martinelli ^1^; G. Castellazzi^1^; H. Basedau^2^; E. Caronna^3^; M. Pocora^1^; R. Greco^1^; V. Gallardo Lopez^3^; L. Asskour^3^; E. Ginè Ciprès^3^; A. Alpuente^3^; M. Torres‐Ferrus^3^; A. Zanaboni^1^; J. Mehnert^2^; P. Pozo‐rosich^3^; C. Tassorelli^4^

##### ^1^IRCCS C. Mondino Foundation, Pavia, Italy; ^2^University Medical Center Hamburg‐Eppendorf, Institute of Systems Neuroscience, Hamburg, Germany; ^3^Headache and Neurological Pain Research Group, Vall d’Hebron Research Institute, Department of Neuroscience, Universitat Autònoma de Barcelona, Barcelona, Spain; Neurology Department, Hospital Universitari Vall d’Hebron, Barcelona, Spain; ^4^IRCCS C. Mondino Foundation, Pavia, Italy, University of Pavia, Brain and Behavioral Science Department, Pavia, Italy

**Background and aims:** The BIOMIGA project aimed to identify multimodal biomarkers predicting response to erenumab, an anti‐CGRP monoclonal antibody, in subjects with migraine. To do a preliminary analysis of the structural neuroimaging data comparing healthy controls with people with migraine and, within, this latter group, responders with non‐responders to ereneumab.

**Methods:** After preregistration (clinical trials: NCT04503083) data were collected in three European headache centers (Italy, Spain, Germany). T1 3D images were acquired with 3T MRI scanners at baseline and after a 3‐month treatment with erenumab. Patients were classified as responders if a reduction ≥ 50% monthly headache/days was achieved. Matched healthy subjects were assessed as a control group. Images were analyzed with FreeSurfer to assess cortical thickness differences.

**Results:** The population consisted of 155 subjects living with migraine and 78 matched healthy subjects. At baseline, in the right hemisphere, people living with migraine exhibited a statistically significant increased cortical thickness in regions such as the subparietal sulcus, precuneus, and posterior ventral cingulate gyrus compared to controls. Similarly, in the left hemisphere, regions such as the intraparietal sulcus and precuneus showed significant cortical thickening, as presented in Table 1. After the 3‐month treatment, no significant differences in cortical thickness were detected when comparing responders with non‐responders.

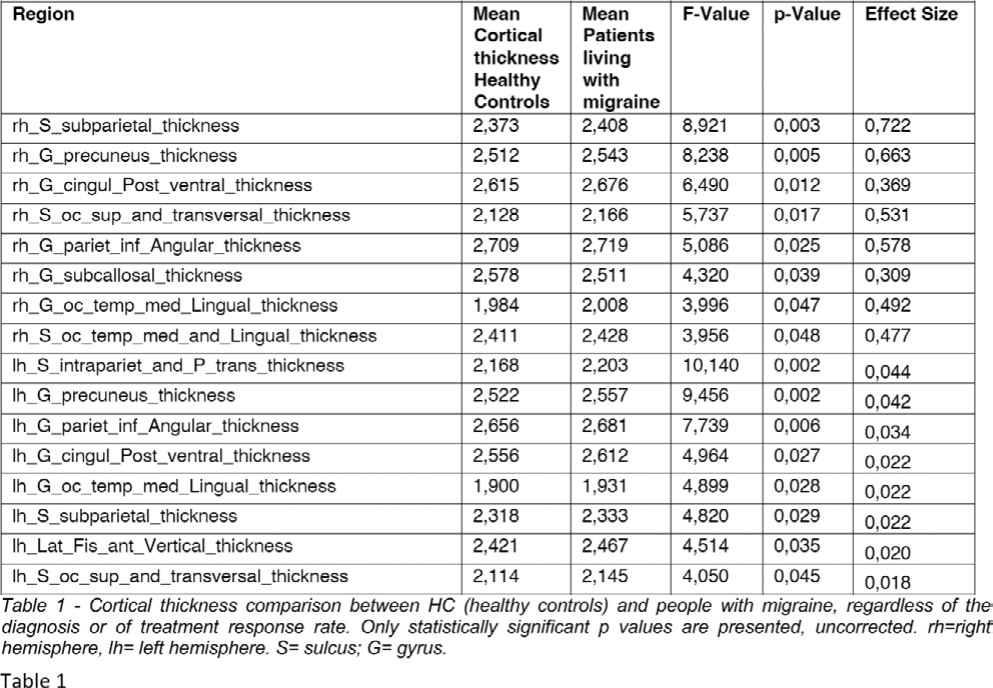

**Conclusion:** Migraine is associated with structural alterations in widespread gray matter regions of the brain. Moreover, the results suggest that 3‐month treatment with erenumab is not sufficient to induce structural changes in the cortical regions evaluated. Founding: Biomiga was funded by ERANet Neuron.

**Disclosure:** DM declares honoraria from Lundbeck and AbbVie HB declares honoraria from Novartis, Teva, Lundbeck and Eli Lilly P.P.‐R. declares honoraria from AbbVie, Amgen, Dr Reddy's, Eli Lilly, Lundbeck, Medscape, Novartis, Organon, Pfizer and Teva Pharmaceuticals. Her research group has received research grants from AbbVie, AGAUR, EraNet Neuron, FEDER RIS3CAT, Instituto Investigación Carlos III, MICINN, Novartis, and Teva Pharmaceuticals, and has received funding for clinical trials from AbbVie, Amgen, Biohaven, Eli Lilly, Lundbeck, Novartis, Pfizer and Teva Pharmaceuticals. She is the Honorary Secretary of the International Headache Society, is on the editorial board of Revista de Neurologia, is an associate editor for Cephalalgia, Headache, Neurologia, Frontiers of Neurology, and is an advisor of the Scientific Committee of the Editorial Board of The Journal of Headache and Pain. CT has received, in the last 3 years, personal fees for the participation in advisory boards or for speaking at sponsored symposia from AbbVie, Eli Lilly, Ipsen, Lundbeck, Medscape, Pfizer, and Teva. Her research group has received research grants from AbbVie, EraNet Neuron, Migraine Research Foundation and competitive grant from the Italian Ministry of Health. Her institution has received payments for clinical trials from AbbVie, Biohaven, Eli Lilly, Ipsen, Lundbeck, Pfizer and Teva. She is past‐President of the International Headache Society, Associate Editor of Cephalalgia.

## EPR‐038

### Fremanezumab initiation at early disease stages may improve migraine outcomes: Post hoc analysis of the PEARL study

#### M. Ashina^1^; D. Mitsikostas^2^; P. Pozo‐Rosich^3^; C. Tassorelli^4^; P. Kokturk^5^; H. Akcicek ^5^

##### ^1^Department of Neurology, Danish Headache Center, Copenhagen University Hospital– Rigshospitalet Glostrup, Copenhagen, Denmark; Department of Clinical Medicine, University of Copenhagen, Copenhagen, Denmark; ^2^Department of First Neurology, Aeginition Hospital, National and Kapodistrian University of Athens, Athens, Greece; ^3^Headache Unit and Research Group, Vall d’Hebron Hospital and Research Institute, Universitat Autonoma de Barcelona, Barcelona, Spain; ^4^Department of Brain and Behavioral Sciences, University of Pavia, Pavia, Italy; IRCCS C. Mondino Foundation, Pavia, Italy; ^5^Teva Netherlands B.V., Haarlem, The Netherlands

**Background and aims:** Due to reimbursement limitations, patients with migraine may only have access to preventive treatment with calcitonin gene‐related peptide pathway monoclonal antibodies after multiple failures of traditional preventive treatments. PEARL (EUPAS35111) was a 24‐month, prospective, observational study that evaluated the effectiveness and safety of fremanezumab for episodic and chronic migraine (EM, CM) prevention.

**Methods:** This post hoc analysis evaluated the impact of migraine type (EM, CM) and prior preventive treatment failures (0–2 or > = 3) on the proportion of participants with a > = 50% reduction in monthly migraine days (MMD) during the 6‐month period following fremanezumab initiation (PEARL primary endpoint). In addition, a multivariate logistic regression model was used to identify independent variables associated with treatment response.

**Results:** Overall, 1128 participants (EM, *n* = 374; CM, *n* = 754) were included. During the 6‐month treatment period, a > = 50% reduction in MMD was achieved in 68.4% of participants with EM and 0–2 treatment failures, compared with 50.5% of participants with CM and > = 3 treatment failures; this trend was observed over 24 months (Figure 1). Factors associated with a higher likelihood of achieving a 50% reduction in MMD were EM versus CM and no prior use of onabotulinumtoxinA (Table 1).

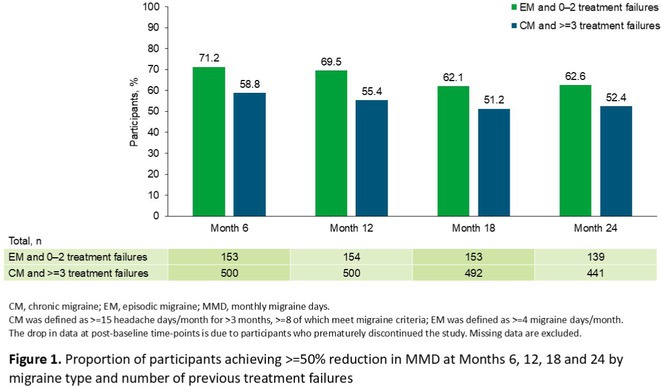

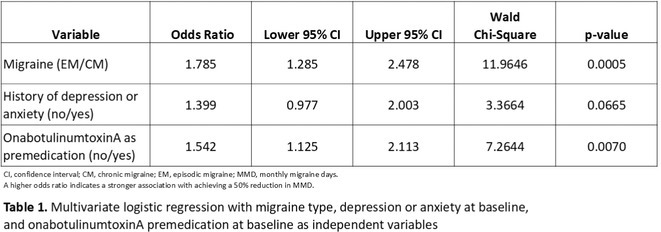

**Conclusion:** This analysis suggests a trend toward enhanced effectiveness of fremanezumab in patients with EM and fewer prior treatment failures, compared with CM and more treatment failures. Further prospective research is needed to fully interpret and confirm a true causal relationship.

**Disclosure:** MA: AbbVie, Amgen, AstraZeneca, Eli Lilly, GlaxoSmithKline, Lundbeck, Novartis, Novo Nordisk Foundation, Pfizer, Teva Pharmaceuticals. DDM: Allergan, Amgen, Bayer, Biogen, Cefaly, electroCore, Eli Lilly, Genesis Pharma, Merck Serono, Merz, Mylan, Novartis, Roche, Sanofi Genzyme, Specifar, Teva Pharmaceuticals. PPR: AbbVie, AGAUR, Amgen, Biohaven EraNet NEURON, Chiesi, Eli Lilly, Lundbeck Instituto Investigación Carlos III, MINECO, Novartis, Pfizer, RIS3CAT FEDER, Teva Pharmaceuticals. CT: AbbVie, Chordate, Dompé, Eli Lilly, Ipsen, Lundbeck, Novartis, Pfizer, Teva Pharmaceuticals, European Commission, Italian Ministry of Health, Migraine Research Foundation. PK, HA: Teva Pharmaceuticals. This study was funded by Teva Pharmaceuticals.

## EPR‐039

### Real‐world effectiveness of fremanezumab in migraine prevention: Final outcomes of the Pan‐European PEARL study

#### M. Ashina ^1^; D. Mitsikostas^2^; F. Amin^1^; P. Kokturk^3^; C. Schankin^4^; G. Sahin^5^; P. Pozo‐Rosich^6^; P. Dorman^7^; T. Nežádal^8^; I. Pavão Martins^9^; M. Sumelahti^10^; V. Ramirez Campos^11^; H. Akcicek^3^; C. Tassorelli^12^

##### ^1^Department of Neurology, Danish Headache Center, Copenhagen University Hospital– Rigshospitalet Glostrup, Copenhagen, Denmark; Department of Clinical Medicine, University of Copenhagen, Copenhagen, Denmark; ^2^Department of First Neurology, Aeginition Hospital, National and Kapodistrian University of Athens, Athens, Greece; ^3^Teva Netherlands B.V., Haarlem, The Netherlands; ^4^Department of Neurology, Inselspital, University Hospital Bern, University of Bern, Bern, Switzerland; ^5^Department of Clinical Sciences of Lund, Lund University, Skåneuro Neurology Clinic, Lund, Sweden; ^6^Headache Unit and Research Group, Vall d’Hebron Hospital and Research Institute, Universitat Autonoma de Barcelona, Barcelona, Spain; ^7^The Newcastle upon Tyne Hospitals NHS Foundation Trust, Newcastle upon Tyne, UK; ^8^Institute of Neuropsychiatric Care, First Faculty of Medicine, Charles University, Prague, Czechia; ^9^Centro de Estudos Egas Moniz, Faculty of Medicine, University of Lisbon, Lisbon, Portugal, ^10^Faculty of Medicine and Health Technology, University of Tampere, Tampere, Pirkanmaa, Finland, ^11^Teva Branded Pharmaceutical Products R&D, Inc., West Chester, USA, ^12^Department of Brain and Behavioral Sciences, University of Pavia, Pavia, Italy; IRCCS C. Mondino Foundation, Pavia, Italy

**Background and aims:** PEARL (EUPAS35111) was a 24‐month observational, prospective, Phase 4 study evaluating the real‐world effectiveness and safety of fremanezumab for episodic and chronic migraine (EM, CM) prevention. Here we report effectiveness, and treatment adherence and persistence outcomes from the PEARL study after 24 months of follow up.

**Methods:** Eligible participants were adults with EM or CM receiving fremanezumab for migraine prevention, who maintained a daily headache diary prior to and throughout the study period. The primary endpoint was the proportion of participants with > = 50% reduction in monthly migraine days (MMD) during the 6‐month period after fremanezumab initiation. Secondary endpoints across Months 1–24 included mean change from baseline in MMD, and treatment adherence (participants who took their prescribed dose within  ± 5 days of the scheduled monthly/quarterly dosing regimen, per injection) and persistence.

**Results:** Of 1140 participants enrolled, 1129 were included in the effectiveness analysis (EM, 33.1%; CM, 66.9%; 87.2% female). In participants with available data, 56.5% (637/1128) achieved > = 50% MMD reduction from baseline during the 6‐month period after fremanezumab initiation (Figure 1). Proportions of participants reaching > = 50% reduction in MMD were sustained across Months 1 to 24 (Figure 2), as were reductions in mean MMD (Figure 3). Adherence rates remained high throughout the study (~90%) and 75.6% (854/1129) of participants completed the study.

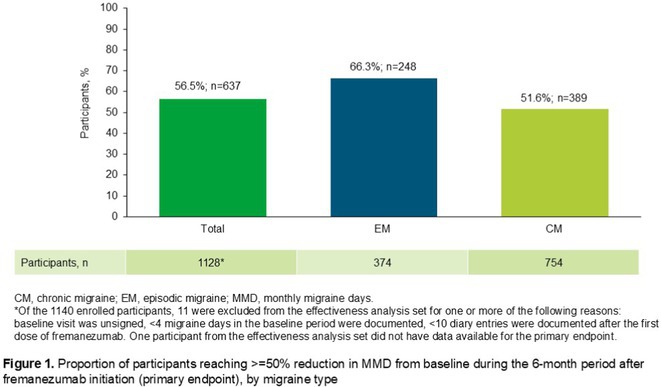

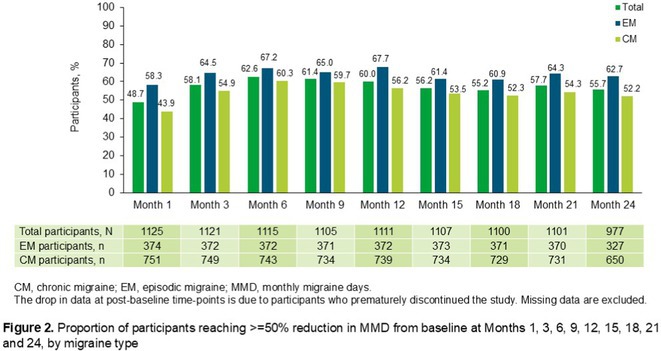

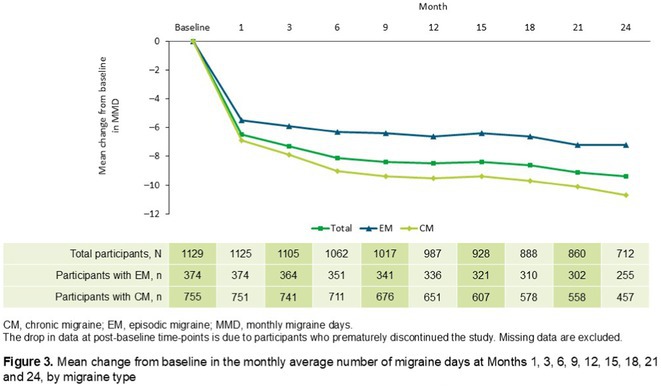

**Conclusion:** These findings underscore the sustained effectiveness and robust adherence of long‐term fremanezumab treatment in migraine prevention.

**Disclosure:** MA: AbbVie, Amgen, AstraZeneca, Eli Lilly, GlaxoSmithKline, Lundbeck, Novartis, Novo Nordisk Foundation, Pfizer, Teva Pharmaceuticals. DM: Allergan, Amgen, Bayer, Biogen, Cefaly, electroCore, Eli Lilly, Genesis Pharma, Merck Serono, Merz, Mylan, Novartis, Roche, Sanofi Genzyme, Specifar, Teva Pharmaceuticals. FMA: AbbVie, Eli Lilly, Lundbeck, Novartis, Pfizer, Teva Pharmaceuticals. CJS: AbbVie, Allergan, Almirall, Amgen, Eli Lilly, Grünenthal, Lundbeck, MindMed, Novartis, Pfizer, Teva Pharmaceuticals, Zynnon, Baasch‐Medicus Foundation, Eye on Vision Foundation, German Migraine and Headache Society. GS: AbbVie, Lundbeck, Novartis, Pfizer, Teva Pharmaceuticals, Vinnova, Lund University, Swedish Neurological Association. PPR: AbbVie, Amgen, Biohaven EraNet NEURON, Chiesi, Eli Lilly, Lundbeck, Instituto Investigación Carlos III, MINECO, Novartis, Pfizer, RIS3CAT FEDER, Teva Pharmaceuticals. PJD: AbbVie, electroCore, Eli Lilly, Lundbeck, Novartis, Pfizer, Teva Pharmaceuticals. TN: AbbVie, Amgen, Eli Lilly, Glenmark, Lundbeck, Neurocrine Novartis, Organon, Pfizer, Teva Pharmaceuticals, UCB. IPM: AbbVie, Allergan, Eli Lilly, Lundbeck, Novartis, Organon, Pfizer, Teva Pharmaceuticals. MLS: AbbVie, Eli Lilly, Lundbeck, Novartis, Pfizer, Teva Pharmaceuticals. CT: AbbVie, Chordate, Dompé, Eli Lilly, Ipsen, Lundbeck, Novartis, Pfizer, Teva Pharmaceuticals, European Commission, Italian Ministry of Health, Migraine Research Foundation. PK, VRC, HA, study funding: Teva Pharmaceuticals.

## EPR‐040

### Efficacy and safety of eptinezumab in chronic migraine: Randomized controlled trial in a predominantly Asian population

#### S. Yu^1^; Y. Matsumori^2^; B. Kim^3^; A. Gryglas‐Dworak^4^; G. Giorgadze^5^; P. Pozo‐Rosich ^6^; M. Josiassen^7^; K. Ranc^7^; A. Ettrup^7^; A. Mittoux^7^; B. Sperling^7^; T. Takeshima^8^

##### ^1^Chinese PLA General Hospital, Beijing, China; ^2^Sendai Headache and Cranial Nerves Clinic, Sendai, Japan; ^3^Eulji University School of Medicine, Seoul, Republic of Korea; ^4^MIGRE Polskie Centrum Leczenia Migreny, Wrocław, Poland; ^5^Aversi Clinic LTD, Tbilisi, Georgia; ^6^Vall d’Hebron University Hospital, Quirón Hospital, Barcelona, Spain; ^7^H. Lundbeck A/S, Copenhagen, Denmark; ^8^Tominaga Hospital, Osaka, Japan

**Background and aims:** This phase 3 clinical trial evaluated the efficacy and safety of eptinezumab for preventive migraine treatment in a predominantly Asian population with chronic migraine (CM).

**Methods:** SUNRISE (NCT04921384) comprised 4 periods: 28‐day screening (baseline); 12‐week double‐blind, placebo‐controlled; 12‐week dose‐blind extension; and 8‐week safety follow‐up. Adults (18‐75y) diagnosed with CM were randomized 1:1:1 to IV eptinezumab 100mg, 300mg, or placebo at baseline. P‐values are versus placebo.

**Results:** Of 983 participants randomized, 939 (95.5%) completed the placebo‐controlled period. Participants were 63.5%/36.5% Asian/European with 17.4 mean monthly migraine days (MMDs) at baseline. Both eptinezumab doses met the primary and all key secondary efficacy endpoints. Primary endpoint: The mean change from baseline in MMDs (Weeks1‐12) were 7.2 (100mg; *p* < 0.0001), 7.5 (300mg; *p* < 0.0001), and 4.8 (placebo). Key secondary endpoints: Eptinezumab demonstrated > 2‐fold higher odds versus placebo for > = 50% (Weeks1‐12, *p* < 0.0001) and > = 75% (Weeks1‐4, *p* < 0.0001; Weeks1‐12, *p* < 0.0001) reduction in MMDs, as well as lower Day 1 migraine rate (*p* < 0.01). Improvements across patient‐reported outcomes showed better effect with eptinezumab than placebo. The rate of treatment‐emergent adverse events (TEAEs) was comparable across groups (100mg, 37.6%; 300mg, 32.2%; placebo, 33.5%), with few serious TEAEs ( < 2%) or TEAEs leading to withdrawal ( < 2%). The most common TEAE was COVID‐19 (100mg, 5.5%; 300mg, 4.6%; placebo, 4.3%).

**Conclusion:** Eptinezumab 100mg and 300mg demonstrated statistically significant greater reductions in monthly migraine days versus placebo in a predominantly Asian chronic migraine population, with efficacy observed as early as Day 1 and sustained through 12 weeks, with a well‐tolerated safety profile consistent with previous trials.

**Disclosure:** Trial sponsored by Lundbeck

## EPR‐041

### Eptinezumab and patient education in chronic migraine and medication‐overuse headache: The randomized RESOLUTION trial

#### R. Jensen ^1^; C. Lundqvist^2^; H. Schytz^1^; C. Tassorelli^4^; F. Vernieri^6^; M. Lantéri‐Minet^8^; G. Terwindt^10^; A. Blumenfeld^11^; S. Tepper^12^; M. Josiassen^13^; G. Jansson^13^; A. Ettrup^13^; A. Mittoux^13^; R. Lipton^14^

##### ^1^Danish Headache Center, Department of Neurology, Rigshospitalet‐Glostrup, University of Copenhagen, Copenhagen, Denmark; ^2^Departments of Neurology and Health Services Research, Akershus University Hospital, Lørenskog, Norway; ^4^Department of Brain and Behavioral Sciences, University of Pavia, Pavia, Italy; ^6^Unit of Headache and Neurosonology, Fondazione Policlinico Campus Bio‐Medico, Rome, Italy; ^8^Pain Department and FHU InovPain, Centre Hospitalier Universitaire de Nice, Nice, France, ^10^Department of Neurology, Leiden University Medical Centre, Leiden, Netherlands, ^11^The Los Angeles Headache Center, Los Angeles, USA, ^12^The New England Institute for Neurology and Headache, Stamford, USA, ^13^H. Lundbeck A/S, Copenhagen, Denmark, ^14^Department of Neurology, Albert Einstein College of Medicine, New York, USA

**Background and aims:** RESOLUTION was a phase 4 clinical trial to evaluate efficacy and safety of eptinezumab added to a Brief Educational Intervention in patients with chronic migraine (CM) and medication‐overuse headache (MOH).

**Methods:** RESOLUTION (NCT05452239) comprised four periods: 28‐day screening (baseline); 12‐week double‐lind, placebo‐controlled; 12‐week open‐label extension; and 8‐week safety follow‐up. Adults (18‐75y) with CM and MOH (excluding opioid‐overuse headache) were randomized (1:1) to infusion with eptinezumab 100 mg or placebo, with all participants receiving Brief Educational Intervention about MOH prior to infusion. The primary endpoint was change from baseline in monthly migraine days (MMDs) across Weeks1‐4.

**Results:** Of 608 participants randomized, 596 (98.0%) completed the placebo‐controlled period. Mean MMDs, monthly headache days (MHDs), and monthly acute medication days (MAMDs) in the total population were 20.9, 21.7, and 20.1, respectively, at baseline. The eptinezumab arm met primary endpoint, with mean change from baseline in MMDs (Weeks1‐4) of 6.9 versus 3.7 with placebo (*p* < 0.0001); it also met all key secondary endpoints (all *p* < 0.0001 vs. placebo): greater reductions in MMDs (Weeks1‐12), MHDs (Weeks1‐4, Weeks1‐12), MAMDs (Weeks1‐4, Weeks1‐12), and average daily pain (Weeks1‐2) and fewer participants still fulfilling CM and MOH criteria (Weeks1‐4, Weeks1‐12). The proportion of participants with treatment‐emergent adverse events was similar between arms (eptinezumab, 41.9%; placebo, 36.9%).

**Conclusion:** Eptinezumab was superior to placebo in reducing monthly migraine days, headache days, and acute medication use in patients with CM and MOH also receiving Brief Educational Intervention. These effects were evident across Weeks1‐4, sustained across Weeks1‐12, and extended to all key secondary endpoints.

**Disclosure:** Trial sponsored by Lundbeck.

## EPR‐042

### Long‐term safety and effectiveness of rimegepant for the preventive treatment of migraine in Japan

#### Y. Matsumori ^1^; S. Kitamura^2^; T. Yamamoto^3^; T. Ishikawa^4^; Y. Hoshino^4^; H. Yoshimatsu^4^; A. Thiry^5^; A. Arakawa^4^; T. Fullerton^5^; F. Sakai^6^; T. Takeshima^7^

##### ^1^Sendai Headache and Neurology Clinic, Sendai‐shi, Miyagi, Japan; ^2^Department of Neurology, Konan Medical Center, Kobe‐shi, Hyogo, Japan; ^3^Department of Neurology, Saitama Medical University, Iruma‐gun, Saitama, Japan; ^4^Pfizer R&D Japan, Shibuya‐ku, Tokyo, Japan; ^5^Pfizer, Groton, USA; ^6^Saitama International Headache Center, Saitama Neuropsychiatric Institute, Saitama‐shi, Saitama, Japan; ^7^Headache Center, Department of Neurology, Tominaga Hospital, Osaka‐shi, Osaka, Japan

**Background and aims:** The aim was to assess the long‐term safety and effectiveness of rimegepant (RIM) 75 mg taken up to once daily for up to 40 weeks for migraine in Japan.

**Methods:** This open‐label (OL) extension of a phase 3, randomized, double‐blind (DB), placebo (PBO)‐controlled study (NCT05399485) included adults in Japan with a history of 4–18 migraine attacks/month of moderate or severe pain intensity. After 12 weeks of DB treatment with RIM 75 mg or PBO every other day (EOD), participants could continue OL treatment for up to 40 weeks, taking RIM 75 mg EOD and additionally as needed on nonscheduled dosing days (maximum 1 dose of RIM 75 mg per calendar day).

**Results:** Of 496 participants who received DB treatment, 458 participants were treated with OL RIM for a mean (standard deviation) of 37.5 (6.3) weeks. The most common adverse events (AEs) were nasopharyngitis (25.1%) and COVID‐19 (13.1%). The rate of AEs leading to RIM discontinuation was 1.3% and the rate of serious AEs was 0.9% (Table 1). There were no deaths. Aminotransferases > 3× the upper limit of normal (ULN) occurred in 4 participants (0.9%); none of these participants had concurrent elevations with total bilirubin > 2× ULN (Table 2). A mean reduction of 4.5 in monthly migraine days from the observation phase was observed through the overall OL phase (Table 3).

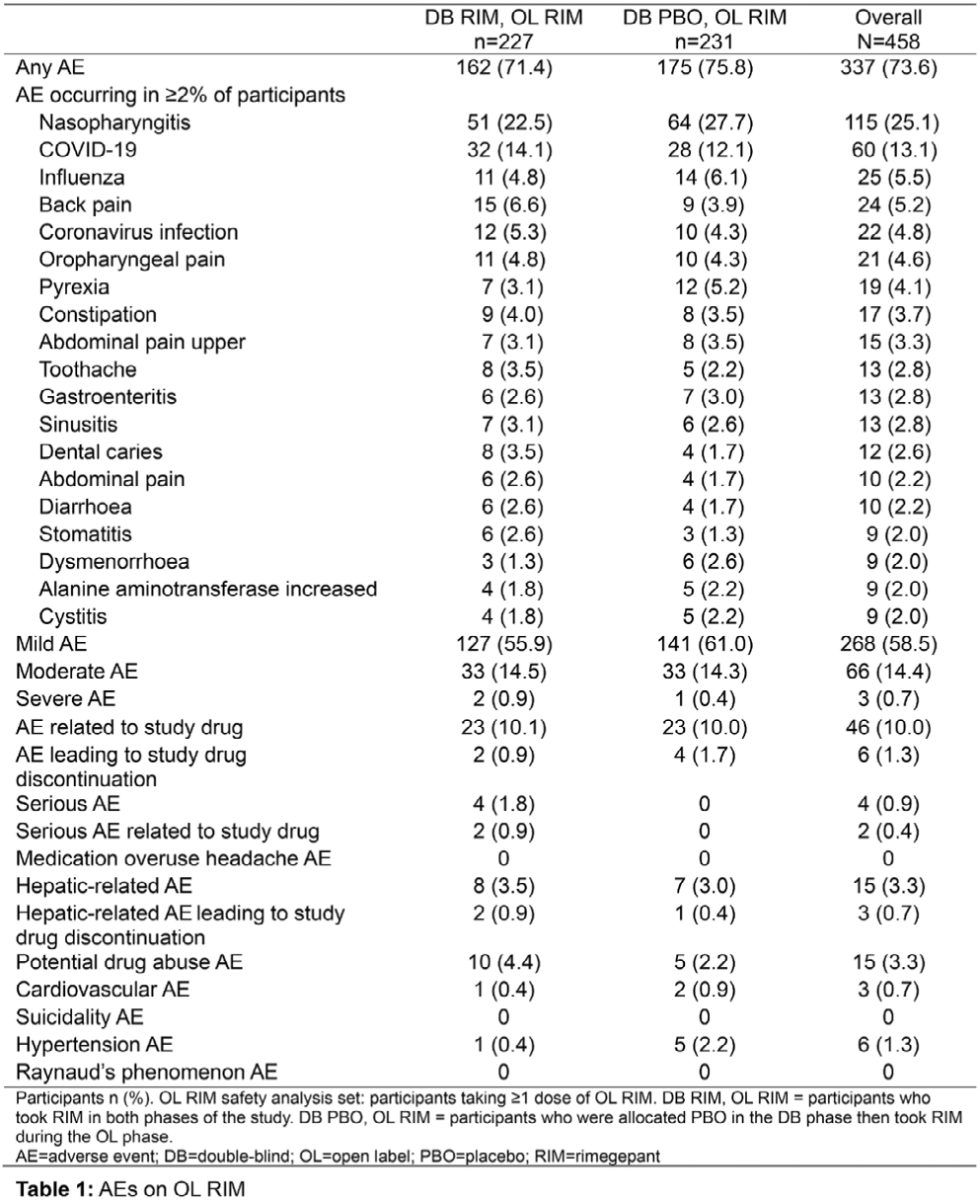

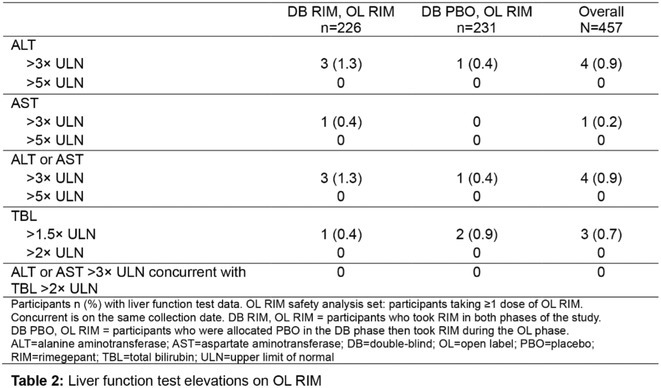

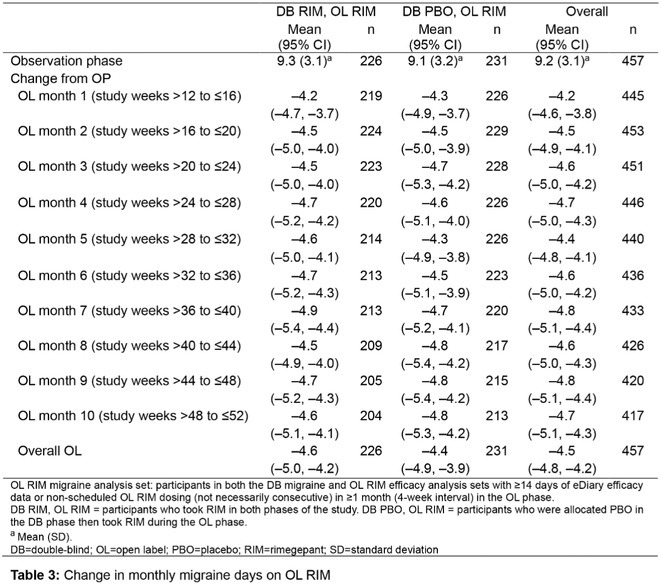

**Conclusion:** Sustained effectiveness was observed in the treatment with OL RIM 75 mg taken up to once daily for up to 40 weeks with a favorable safety profile.

**Disclosure:** Funded by Pfizer. YM received personal consultancy fees from Amgen Astellas BioPharma K.K., Daiichi Sankyo Company, Limited, Eli Lilly Japan K.K. and Otsuka Pharmaceutical Co., Ltd. during the conduct of the study. SK is a consultant for Eli Lilly Japan K.K. TY reports no conflicts of interest. TI is an employee of Pfizer and owns stock/options in Pfizer. YH is an employee of Pfizer and owns stock/options in Pfizer. HY is an employee of Pfizer and owns stock/options in Pfizer. AT was an employee of Biohaven Pharmaceuticals, owns stock in Biohaven Ltd, is an employee of Pfizer, and owns stock/options in Pfizer. AA is an employee of Pfizer and owns stock/options in Pfizer. TF is an employee of Pfizer and owns stock/options in Pfizer. FS is a consultant for Amgen, Otsuka, and Eli Lilly. TT is an advisor for Sawai, Teijin, and Hedgcock MedTech; and reports honoraria (Otsuka, Amgen, Eli Lilly Japan, and Daiichi‐Sankyo) and grants for commissioned/joint research (Pfizer, Lundbeck, AbbVie, Eli Lilly Japan).

## EPR‐043

### Clinical predictors for efficacy of erenumab for migraine: A Registry for Migraine (REFORM) study

#### W. Karlsson; M. Ashina; R. Christensen; H. Al‐Khazali; H. Ashina

##### Department of Neurology, Danish Headache Center, Copenhagen University Hospital – Rigshospitalet, Copenhagen, Denmark

**Background and aims:** Erenumab is effective for migraine prevention, but many patients experience variable outcomes or no benefit. This study aimed to identify clinical factors associated with therapeutic response to erenumab using data from a large cohort of patients with migraine.

**Methods:** A single‐center, prospective study included 570 adults with ≥4 monthly migraine days, treated with 140 mg of erenumab monthly for 24 weeks. Participants recorded their responses in headache diaries, and outcomes were classified based on achieving a ≥50% reduction in monthly migraine days between weeks 13 and 24. Logistic regression analysis was used to identify clinical factors associated with response.

**Results:** Among participants, 298 (52.3%) were responders, and 272 (47.7%) were non‐responders. Factors associated with a lower likelihood of response included chronic migraine (odds ratio [OR] 0.63), daily headache (OR 0.41), and failure of ≥3 preventive medications (OR 0.54). Conversely, higher age (10‐year increase: OR 1.22) predicted better outcomes. Early responders (≥50% reduction within weeks 1–12) were less likely to have chronic migraine, had lower Migraine Disability Assessment Scores, more often experienced unilateral headache, and had lower allodynia scores compared to late responders.

**Conclusion:** In conclusion, chronic migraine, daily headache, and multiple preventive medication failures were associated with poorer responses to erenumab, while older age predicted better outcomes. Further research is needed to establish the optimal timepoint for evaluating efficacy of erenumab and to integrate novel clinical and biomarker data to enhance predictive accuracy.

**Disclosure:** WKK and HMA reports receiving personal fees from Lundbeck and Pfizer, outside of the submitted work. MA reports having been a consultant, speaker, or scientific advisor for AbbVie, Amgen, Astra Zeneca, Eli Lilly, GlaxoSmithKline, Lundbeck, Novartis, Pfizer, and Teva. MA also reports being a primary investigator for ongoing AbbVie and Pfizer trials. MA is supported through the Lundbeck Foundation Professor Grant (R310‐2018‐3711) and serves as an Associate Editor of the Brain and The Journal of Headache and Pain. RHC reports having received a travel grant from the Augustinus Foundation. HA reports receiving personal fees from Lundbeck, Pfizer, and Teva, outside of the submitted work.

## EPR‐044

### Profiles of migraine patients treated with triptan: A study based on data from the French Nationwide claims database

#### X. Moisset ^1^; M. Lanteri‐Minet^2^; C. Lucas^3^; J. Mawet^4^; M. Dalichampt^5^

##### ^1^Clermont Auvergne University, University Hospital Center of Clermont‐Ferrand, Inserm, Neuro‐Dol, Clermont‐Ferrand, France; ^2^Pain Department and FHU InovPain, CHU Nice and Côte Azur University, Nice, France; and INSERM U1107 Migraine and Trigeminal Pain, Auvergne University, Clermont‐ Ferrand, France; ^3^Pain Unit, Neurosurgery department, Lille University Hospital, Lille, France; ^4^Department of Neurology, Lariboisière Hospital, AP‐HP, Paris, France; ^5^Regional Health Observatory, Pays de la Loire, Nantes, France

**Background and aims:** Although recommendations for the diagnosis and management of migraine have been published, it is managed in a very heterogeneous way. Here, we describe the profiles and care pathways of migraine patients treated with triptan in France.

**Methods:** Retrospective cohort study of migraine patients treated with at least one triptan, identified in the 2/100th French Nationwide claims database (ESND) from 2016 to 2021 and analyzed from 2006 to 2022.

**Results:** Among the 37,884 migraine patients identified, we found that 76% were women. The mean age was 36.6 years at the first triptan. Annual mean number of general practitioner consultations before and after the first triptan dispensing were 5.4 and 6.1, 2.5 and 3.3 in psychiatry, and 0.3 and 0.5 in neurology. Sick leave concerned 43% and 52% of patients. Preventive migraine treatments were dispensed to 9% of patients within the year before the first triptan dispensing and to 39% after. NSAIDs were dispensed to 52% of patients before and to 89% after. Triptan treatment was permanently stopped within 4 months of dispensing for 39% of patients, while 61% of patients continued it during the follow‐up.

**Conclusion:** We highlight here the heterogeneous management of triptan users and the change of care pathways after the first triptan dispensing.

**Disclosure:** This research was conducted with support from Orion Pharma. The authors are members of Orion's advisory board XM has received personal fees from Allergan‐Abbvie, Aptis Pharma, Biogen, BMS, Grünenthal, HAS, Lilly, Lundbeck, Teva, Merck‐Serono, Novartis, Pfizer, Roche, and Sanofi‐Genzyme / grants from APICIL, region Auvergne‐Rhone‐Alpes, contrat Interface Inserm / non‐financial support from SOS Oxygène, not related to the submitted work MLM: Personal fees* and research support for FHU InovPain University Côte d’Azur° with: Abbvie/Allergan*, Amgen*°, Astellas*°, ATI*°, BMS°, Biogen°, Boehringer*°, Boston Scientific*, CoLucid°, Convergence°, Glaxo‐SmithKline*, Grunenthal*°, Eli Lilly*°, IPSEN*, Lundbeck*°, Medtronic*°, Menari*, MSD*, Novartis*°, Orion Pharma*, Perfood*, Pfizer*°, ReckittBenckiser*, Saint‐Jude*, Sanofi‐Aventis*, Teva*°, UCB*°, UPSA*, Zambon*; Grants: DGOS (PHRC), ANSM, SFETD, Fondation APICIL, Migraine Foundation JM has received financial supports for boards and meeting presentations from Lilly, Abbvie, TEVA, Lundbeck, Pfizer, Orion Pharma / travel fees for congress from Ipsen Pharmaceuticals, Abbvie, Lundbeck, SOS Oxygen, Dr Reddy's CL has received personal fees from Allergan‐Abbvie, Homeperf, Lilly, Lundbeck, Teva, Orion, Pfizer; Past President of the French Headache Society

## Movement disorders 1

## EPR‐045

### Plasma neuronal biomarkers in Parkinson's disease with and without GBA1 mutations: A multicenter study

#### A. Magliozzi ^1^; A. Lupini^1^; C. Zizzo^2^; C. Tolassi^1^; C. Zatti^1^; A. Galli^1^; G. Anzini^3^; S. Toro^3^; G. Duro^2^; A. Di Fonzo^4^; M. Marano^3^; A. Pilotto^1^; A. Padovani^1^

##### ^1^Department of continuity of care and frailty, Neurology Unit, ASST Spedali Civili of Brescia, Brescia, Italy; ^2^Institute for Biomedical Research and Innovation (IRIB‐CNR), National Research Council of Italy, Palermo, Italy; ^3^Department of Neurology, Università Campus Bio‐Medico di Roma, Roma, Italy; ^4^Neuroscience Section, Department of Pathophysiology and Transplantation, Dino Ferrari Center, University of Milan, Milan, Italy

**Background and aims:** Mutations in the GBA1 gene are the most significant genetic risk factor for Parkinson's disease (PD), contributing to lysosomal dysfunction and impaired glucocerebrosidase (GCase) activity, leading to alpha‐synuclein accumulation. Limited studies have evaluated the effect of GBA1 mutations on plasma biomarkers of neurodegeneration and glial activation.

**Methods:** We enrolled 122 PD patients from two Italian centers, including 41 GBA1 mutation carriers and 81 non‐carriers. Motor symptoms were assessed using MDS‐UPDRS III, and non‐motor symptoms with NMSS and MoCA. GCase activity and Lyso‐GBA1 levels were measured in a subset of patients. Plasma levels of GFAP, NfL, and pTau181 were quantified using the SIMOA assay.

**Results:** No significant differences were found in motor features between groups. However, GBA1 carriers exhibited more severe hallucinations, mood disturbances, and cognitive impairment (NMSS domains 3 and 4, *p* = 0.005, *p* = 0.008). Plasma biomarker levels did not significantly differ between groups. In unadjusted analysis, MoCA scores negatively correlated with NfL and GFAP, with a stronger association in GBA1 carriers (GFAP, *p* = 0.001, ρ = ‐0.583; NfL, *p* = 0.002, ρ = ‐0.542) compared to non‐carriers (GFAP, *p* = 0.017, ρ = ‐0.416; NfL, *p* = 0.075, ρ = ‐0.309).

**Conclusion:** GBA1 mutation carriers exhibited a more aggressive non‐motor phenotype. However, plasma biomarker levels did not significantly differ between groups. The stronger correlation between clinical features and biomarkers in GBA1 carriers suggests a potential impact of the mutation on disease progression. Larger studies are needed to confirm these findings.

**Disclosure:** Nothing to disclose.

## EPR‐046

### Micrographia in Parkinson's disease: Automatic recognition through artificial intelligence

#### G. Pinola ^1^; G. Saurio^2^; F. Asci^1^; M. Falletti^1^; A. Zampogna^1^; M. Patera^1^; F. Fattapposta^1^; S. Scardapane^2^; A. Suppa^1^

##### ^1^Department of Human Neurosciences, Sapienza University of Rome, Rome, Italy; ^2^Department of Information, Electronic and Communication Engineering (DIET), Sapienza University of Rome, Rome, Italy

**Background and aims:** Parkinson's disease (PD) leads to handwriting abnormalities primarily characterized by micrographia. Whether micrographia manifests early in PD, worsens throughout the disease, and lastly responds to L‐Dopa is still under scientific debate. Owing to the biological complexity of human handwriting, innovative technological strategies are warranted to examine micrographia objectively in PD and fill the current knowledge gaps.

**Methods:** A total of 57 PD patients on chronic L‐Dopa treatment were enrolled, including 30 patients in the early stages (H&Y≤2) and 27 in the mid‐advanced stages (H&Y > 2), alongside 25 age‐ and sex‐matched controls. Participants completed two standardized handwriting tasks in an ecological scenario. Handwriting samples were examined through clinically‐based (i.e., perceptual) and artificial intelligence (AI)‐based (automatic) procedures. Consistent (i.e., average stroke size) and progressive micrographia (sequential changes in stroke size) were both evaluated. Receiver operating characteristic (ROC) curves were used to evaluate the accuracy of the convolutional neural network (CNN) in classifying handwriting in PD and controls.

**Figure:**
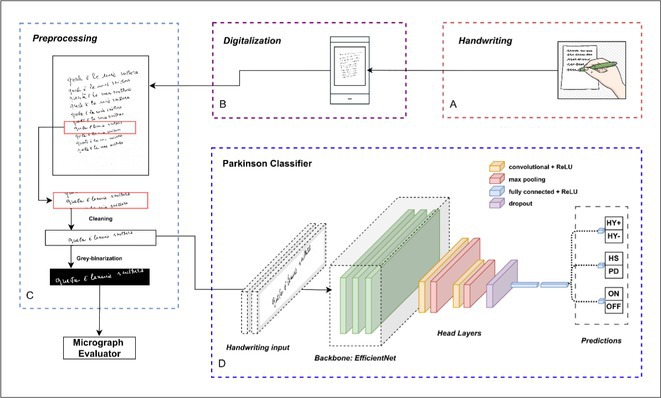
**FIGURE 1** Dataset building: (A) Acquisition of handwriting samples. (B) Digitalization of the handwriting samples. (C) Preprocessing of the handwriting samples. (D) Model of deep learning architecture.

**Results:** Clinically and AI‐based analysis revealed a general reduction in stroke size in PD supporting the concept of parkinsonian micrographia. Compared with perceptual analysis, AI‐based analysis clarified that micrographia manifests early during the disease, progressively worsens and poorly responds to L‐Dopa. The AI models achieved high accuracy in distinguishing PD patients from controls (91%), and moderate accuracy in differentiating early from mid‐advanced PD (77%). Lastly, the AI model failed in discriminating patients in OFF and ON states.

**Conclusion:** AI‐based handwriting analysis is a valuable tool for detecting and quantifying micrographia in PD.

**Disclosure:** Nothing to disclose.

## EPR‐047

### Optimal cut‐off for the mini mental state examination and montreal cognitive assessment in multiple system atrophy

#### I. Carotenuto ^1^; S. Cuoco^1^; M. Russillo^1^; V. Andreozzi^1^; M. Picillo^1^; M. Amboni^1^; R. Erro^1^; A. Soricelli^2^; P. Barone^1^; M. Pellecchia^1^

##### ^1^Neurological Clinic, AOU San Giovanni di Dio e Ruggi d'Aragona, Salerno, Italy; ^2^IRCCS SynlabSDN, Naples, Italy

**Background and aims:** Mild cognitive impairment (MCI) and dementia are reported in up to 44% and 7% of patients with Multiple system atrophy (MSA), respectively. No study has explored the sensitivity and discriminative power of Mini Mental State Examination (MMSE) and Montreal Cognitive Assessment (MOCA) to detect MCI and dementia in MSA. We aimed to identify the optimal cut‐off of MMSE and MOCA in order to distinguish MSA patients with MCI and dementia from patients with normal cognition (NC). The fluency item of MOCA was also assessed separately for the same purpose.

**Methods:** Sixty‐two MSA patients underwent a comprehensive II level neuropsychological evaluation, in order to diagnose dementia, MCI or NC according to DSM‐5. ROC analyses were used to establish the optimal cut‐off scores for MMSE, MOCA and fluency item of MOCA for MCI and dementia, respectively.

**Results:** According to the II level neuropsychological evaluation, 4.8% of MSA patients were demented and 53,2% had MCI (Tab.1). The optimal cut‐offs for MMSE to identify dementia (AUC = 0.915) and MCI (AUC = 0.698) were 20.5 and 26.5, respectively. The optimal cut‐offs for MOCA to detect dementia (AUC = 0.919) and MCI (AUC = 0.702) were 14.0 and 19.5, respectively ROC analysis suggested that both tests were more accurate to identify MCI than dementia (Fig. 1; Fig. 2). The optimal cut‐off for MOCA fluency item to identify MCI was 8.5 words (AUC = 0.717).

**Figure:**
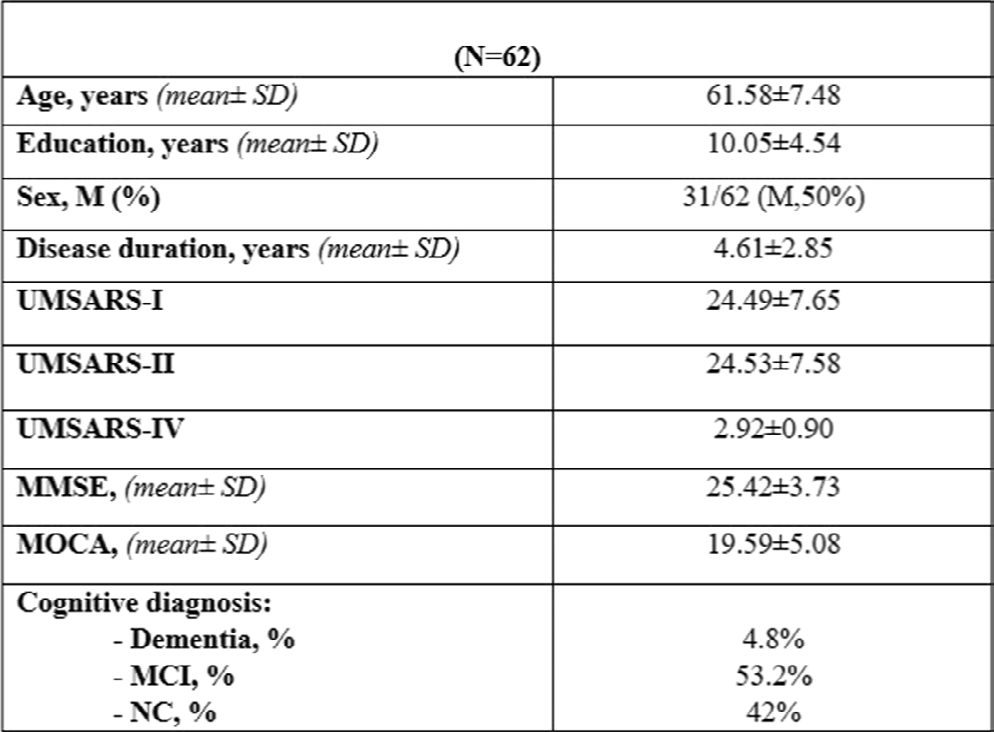
**TABLE 1** Demographic and clinical characteristics of MSA patients Abbreviations: M, males; MCI, mild cognitive impairment; MMSE, Mini Mental State Examination; MOCA, Montreal Cognitive Assessment; N, number; NC, normal cognition; SD, standard deviation; UM.

**Figure:**
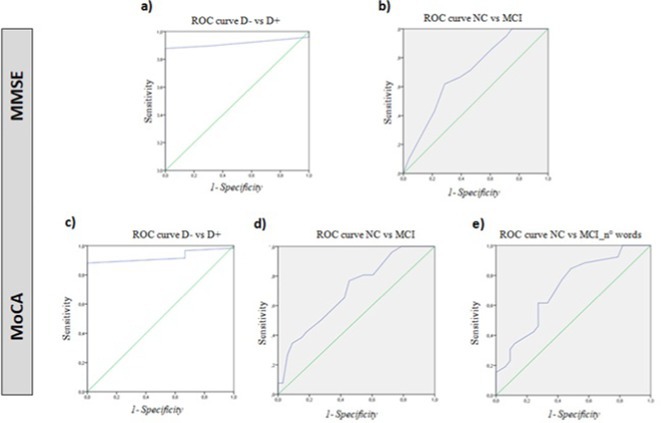
**Figure 1** ROC curves for dementia (a) and MCI (a) for MMSE total score and MOCA total score (c, d) and number of words in fluency items (e) Abbreviations: D‐, absence of dementia; D+, presence of dementia; MCI, mild cognitive impairment; MMSE, Mini mental.

**Figure:**
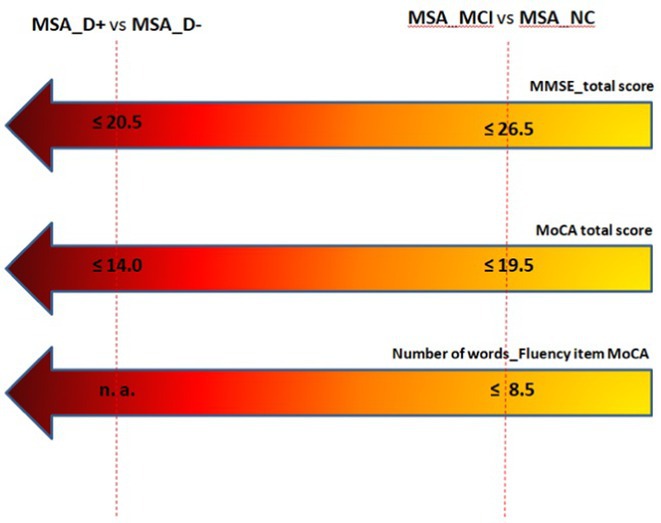
**FIGURE 2** Optimal cut‐offs for MMSE and MOCA to identify dementia and MCI in MSA. Abbreviations: D‐, absence of dementia; D+, presence of dementia; MCI, mild cognitive impairment; MMSE, Mini mental state examination; MOCA, Montreal Cognitive Assessment; M

**Conclusion:** Our findings support MMSE and MOCA as easy and accurate instruments to detect MCI and dementia in MSA. MOCA fluency item is also a reliable tool to detect MCI in the same population.

**Disclosure:** Nothing to disclose.

## EPR‐048

### Prediction of motor progression in Parkinson's disease using clinical data and brain MRI‐derived features

#### J. Simarro; T. Billiet; T. Phan; N. Barros; R. Khan; A. Ribbens

##### icometrix, Leuven, Belgium

**Background and aims:** The MDS‐UPDRS‐III is a standard tool for assessing motor symptoms in Parkinson's disease (PD) and is often employed as an endpoint in clinical trials. This study examines the progression in MDS‐UPDRS‐III scores among patients with early‐stage PD. We hypothesize that baseline clinical data and brain MRI‐derived features can predict motor progression in patients with early‐stage PD.

**Methods:** Data from 519 patients with early‐stage PD (Hoehn & Yahr stage < 2.5, MOCA > 22, mean age 62  ± 9 years, disease duration 1.3  ± 1.6 years) were retrospectively analyzed. Data originated from the PPMI database and Stanford University. Progression in motor signs between 1 and 3 years was evaluated using two criteria: (1) any increase (change > 0) in MDS‐UPDRS‐III scores from baseline, and (2) a clinically significant increase (change > = 5) in MDS‐UPDRS‐III scores from baseline. A Random Forest classifier was trained and validated using five‐fold cross‐validation. Input variables included baseline clinical data (Hoehn & Yahr stage, MDS‐UPDRS‐III, age) and head‐size‐normalized volumes of cortical lobes and subcortical structures (caudate nucleus, putamen, hippocampus, thalamus, and lateral ventricles) obtained with the icobrain software. Model performance was assessed using the mean and 95% confidence interval of the area under the receiver operating characteristic curve (AUC), accuracy, specificity, sensitivity, and precision.

**Results:** Table 1 summarizes the performance metrics, showing an AUC of 0.76 for predicting MDS‐UPDRS‐III change and 0.66 for predicting severe changes.

**Figure:**
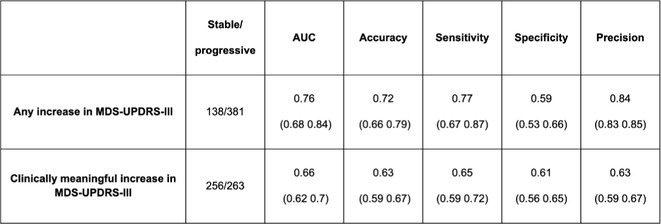
**TABLE 1** The mean and 95% confidence interval of a random forest classifier's performance, trained on baseline clinical data and MRI‐derived features to predict MDS‐UPDRS‐III changes. AUC = area under the receiver operating characteristic curve.

**Conclusion:** Baseline clinical data combined with MRI‐derived features have the potential to predict future changes in motor symptoms in early‐stage PD.

**Disclosure:** The author declares that financial support was received for the research by icometrix.

## EPR‐049

### Switching from placebo to opicapone in non‐fluctuating Parkinson's disease patients: Findings from EPSILON study

#### J. Ferreira ^1^; O. Rascol^2^; F. Stocchi^3^; A. Antonini^4^; G. Castilla‐Fernández^5^; H. Brigas^6^; J. Moreira^6^; J. Rocha^6^; M. Fonsecca^5^; J. Holenz^6^; W. Poewe^7^

##### ^1^IMM ‐ Instituto de Medicina Molecular João Lobo Antunes, Faculdade de Medicina, Universidade Lisboa, Lisbon, Portugal; ^2^University of Toulouse, University Hospital of Toulouse, INSERM, Clinical Investigation Center CIC1436 Departments of Neurosciences and Clinical Pharmacology and NS‐Park/FCRIN network, Toulouse, France; ^3^Department of Neurology, IRCCS San Raffaele Pisana, Rome, Italy; ^4^Department of Neurosciences, University of Padova, Padova, Italy; ^5^BIAL R&D Investments, S.A., Portugal; ^6^BIAL – Portela & Ca S.A., Coronado, Portugal; ^7^Department of Neurology, Medical University of Innsbruck, Innsbruck, Austria

**Background and aims:** In the EPSILON study, opicapone (OPC) was more efficacious than placebo in improving motor impairments in levodopa‐treated Parkinson's disease (PD) patients without motor complications. Findings from the open‐label extension (OLE) phase are reported.

**Methods:** In the randomized, double‐blind (DB), placebo‐controlled EPSILON study, levodopa‐treated PD patients without motor complications received OPC 50 mg or placebo for 24 weeks. After completing the DB phase, patients entered the OLE and received OPC 50 mg. Changes from OLE baseline to Week 52 in Movement Disorder Society‐Unified PD Rating Scale (MDS‐UPDRS)‐Part III and Part IV (OLE key endpoint) scores were evaluated. Safety/tolerability were secondary endpoints.

**Results:** From OLE baseline to Week 52, there was a mean (standard error) reduction of ‐2.1 ( ± 7.9) in MDS‐UPDRS‐Part III score in patients transitioning from placebo to OPC (PLC‐OPC group, *N* = 155), but those who received OPC in the DB phase (OPC‐OPC, *N* = 151) showed numerically greater improvements in the score at Week 52 (least‐squares mean difference of ‐1.3 points [95%CI: ‐3.3,0.7; *p* = 0.196] vs. PLC‐OPC) (Figure 1). MDS‐UPDRS‐Part IV total scores remained low (Figure 2a), with a higher proportion of patients remaining free of motor complication (MDS‐UPDRS‐Part IV score = 0) in the OPC‐OPC than in the PLC‐OPC group (80.2% vs. 69.7%, Figure 2b). Time to fluctuations, dyskinesias and dystonia did not significantly differ between groups (Figure 2c). OPC was well‐tolerated.

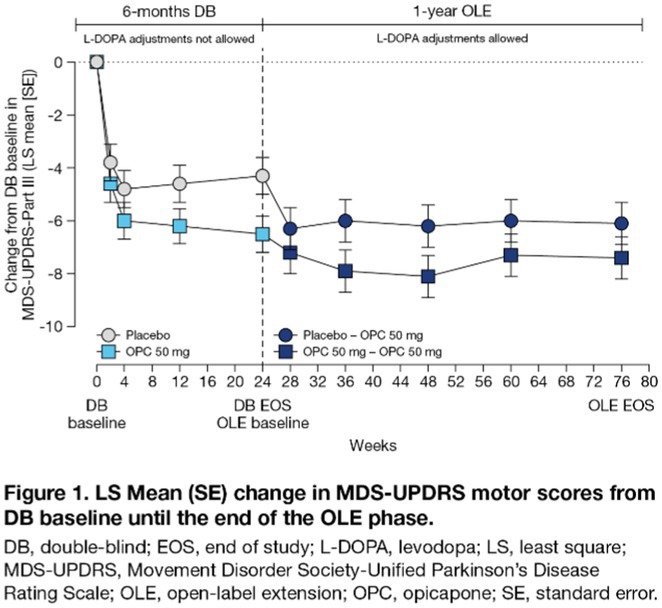

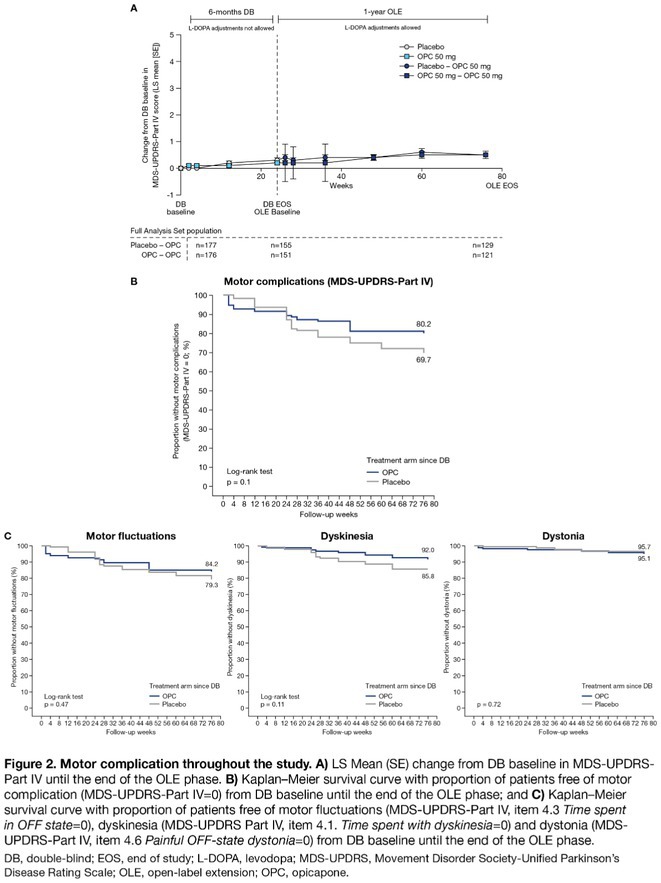

**Conclusion:** OPC provided sustained motor benefits with a higher proportion of patients free of motor complications compared with those initially on placebo. These results support early OPC use in levodopa‐treated PD patients without motor fluctuations.

**Disclosure:** JJF received grants/compensation from GlaxoSmithKline, Grunenthal, Fundação MSD, TEVA, MSD, Allergan, Novartis, Medtronic, GlaxoSmithKline, Novartis, TEVA, Lundbeck, Solvay, BIAL, Merck, Merz, Ipsen, Biogen, Acadia, Allergan, Abbvie, Sunovion Pharma, Zambon, Affiris and Angelini. OR has received compensation from AbbVie, Adamas, Acorda, Addex, AlzProtect, ApoPharma, AstraZeneca, Axovant, BIAL, Biogen, Britannia, Buckwang, CereSpir, Clevexel, Denali, INC Research, IPMDS, Lundbeck, Lupin, Merck, MundiPharma, NeurATRIS, NeuroDerm, Novartis, ONO Pharma, Osmotica, Parexel, Pfizer, Prexton Therapeutics, Quintiles, Roche, Sanofi, Servier, Sunovion, Theranexus, Takeda, Teva, UCB, Vectura, Watermark Research, XenoPort and Zambon; grants from CHU, France‐Parkinson, INSERM, Michael J. Fox Foundation and Cure Parkinson UK. FS received honoraria from Lundbeck, UCB, Chiesi, Zambon, Britannia, Cynapsus, Sunovion, Kyowa, Abbvie, Neuroderm, Biogen and BIAL. AA received compensation/support from UCB, Boehringer Ingelheim, Britannia, AbbVie, Zambon, BIAL, NeuroDerm, Theravance Biopharma and Roche, Chiesi Pharma, Lundbeck and Horizon 2020. WP received fees/honoraria from Alterity, AbbVie, Affiris, AstraZeneca, Axovant, BIAL, Biogen, Britannia, Lilly, Lundbeck, NeuroDerm, Neurocrine, Denali Pharma, Orion Pharma, Roche, Stada, Sunovion, Takeda, UCB and Zambon; grant from Michael J. Fox Foundation, FP7 and Horizon 2020. GCF, HB, JM, FR, MMF and JH are employees of Bial. Study supported by Bial.

## EPR‐050

### Abstract withdrawn

## EPR‐051

### The OLE portion of the PROOF‐HD trial shows persistent benefits of pridopidine on function, cognition, and motor in HD

#### R. Reilmann ^2^; M. Geva1, A. Feigin^3^; A. Rosser^4^; S. Kostyk^5^; K. Chen^1^; A. Tan^1^; A. Cruz‐Herranz^1^; D. Boot^1^; Y. Cohen^1^; Y. Goldberg^1^; M. Hayden^1^

##### ^1^Prilenia Therapeutics B.V., Naarden, The Netherlands; ^2^George Huntington Institute, Muenster, Germany; ^3^NYU Langone Health, New York, USA; ^4^University of Cardiff, Cardiff, UK; ^5^Ohio State University College of Medicine, Columbus, USA

**Background and aims:** Pridopidine is a highly selective and potent S1R agonist in clinical development for Huntington's disease (HD) and ALS.

**Methods:** PROOF‐HD was a Ph3, global, double‐blind, placebo‐controlled trial assessing pridopidine (45 mg bid) in early manifest HD. The double‐blind (DBP) duration was 65‐78wks, followed by an open‐label period (OLE) (total 104 weeks). Key endpoints include change from baseline through wk104 in functional capacity (TFC), progression (cUHDRS), cognition (SWR), and motor (Q‐Motor). Prespecified subgroup analysis excluded participants on antidopaminergics (ADMs; VMAT2 inhibitors and antipsychotics).

**Results:** Pridopidine was well tolerated with a safety profile comparable to placebo. Pridopidine did not show significant benefits in all subjects. However, in prespecified subjects off ADMs, pridopidine was superior to placebo across key independent measures of HD progression, at all timepoints through the DBP. In subjects off ADMs treated with pridopidine during the DBP and OLE show persistent benefits lasting through wk104 across all endpoints compared to a propensity matched cohort from both the ENROLL‐HD and TRACK‐HD observational studies. Pridopidine demonstrated improvements in cUHDRS (ENROLL‐HD: Δ0.90, *p* < 0.0001; TRACK‐HD: Δ1.19, *p* < 0.0001), TFC (ENROLL‐HD: Δ0.76, *p* < 0.0001; TRACK‐HD: Δ0.71, *p* = 0.0004), SWR (ENROLL‐HD: Δ6.47, *p* < 0.0001; TRACK‐HD: Δ8.16, *p* = 0.0003), and Q‐Motor FT IOI (ENROLL: did not assess Q‐Motor; TRACK‐HD: Δ‐77.12 ms, *p* < 0.0001). Pridopidine inhibits CYP2D6 and its concomitant use with certain ADMs (CYP2D6 metabolized) increases exposure of ADMs. Participants on recommended adjusted doses of ADMs (per regulatory label guidance) maintain positive benefits of pridopidine.

**Conclusion:** Pridopidine shows consistent, sustained and clinically meaningful benefits across multiple endpoints of HD progression through 2 years.

**Disclosure:** This study was sponsored by Prilenia Therapeutics.

## EPR‐052

### Effect of 24‐hour subcutaneous levodopa/carbidopa infusion (ND0612) on motor fluctuations in the Phase 3 BouNDless study

#### O. Rascol^1^; A. Ellenbogen^2^; R. Hauser^3^; F. Stocchi^4^; R. Pahwa^5^; J. Ferreira ^6^; K. Kieburtz^7^; A. Albanese^8^; L. Salin^9^; N. Sasson^9^; L. Adar^9^; N. Vostokova^9^; N. Lopes^9^; J. Pereira^10^; A. Espay^11^

##### ^1^University of Toulouse 3, University Hospital of Toulouse, INSERM, Clinical Investigation Center CIC1436 Toulouse, France; ^2^Quest Research Institute, Farmington Hills, USA; ^3^University of South Florida, Tampa, USA; ^4^University San Raffaele Roma and Institute for Research and Medical Care IRCCS San Raffaele, Roma, Italy; ^5^University of Kansas Medical Center, Kansas City, USA; ^6^Laboratory of Clinical Pharmacology and Therapeutics, Faculdade de Medicina, Universidade de Lisboa, Lisbon, Portugal; ^7^Clintrex Research Corp, Sarasota, USA; ^8^Department of Neurology, IRCCS Istituto Clinico Humanitas. Rozzano MI, Italy; ^9^NeuroDerm Ltd., Rehovot, Israel, ^10^Mitsubishi Tanabe Pharma America, Inc., Jersey City, USA, ^11^James J. and Joan A. Gardner Center for Parkinson's Disease and Movement Disorders, University of Cincinnati, Cincinnati, USA

**Background and aims:** Treatment with investigational ND0612 added 1.72h of Good ON‐time over immediate‐release levodopa/carbidopa (IR‐LD/CD; *p* < 0.0001) in the phase 3 BouNDless study (NCT04006210). Patients who completed the double‐blind phase of this study were eligible to enter into the ND0612 open‐label extension (OLE) phase ( < = 54 months). Here, we report the effect of ND0612 on OFF and ON episodes, their duration, and transitions in the BouNDless study and 1‐year OLE phase.

**Methods:** Data collection involved post hoc analysis of diary data and descriptive analysis of the number and duration of episodes spent in any PD motor state. The total number of transitions between motor states were analyzed by baseline‐adjusted Poisson Regression. Analysis of OLE phase outcomes was performed until the last patient completed 1‐year following OLE enrollment. Changes in diary states were measured from ND0612 initiation in the run‐in phase of the study.

**Results:** At the end of the double‐blind phase, ND0612 treatment led to fewer OFF episodes/day and shorter OFF duration (2.4 vs. 3.3; 3.8h vs. 5.2h), fewer episodes and prolonged duration of ‘ON without dyskinesia’ (2.7 vs. 3.1; 9.4h vs. 7.4h) and fewer daily transitions between OFF and ON (5.3 vs. 7.1) compared with IR‐LD/CD (*p* < 0.0001). At the end of OLE Month 12, the LS mean ± SE changes in OFF‐time (−1.86 ± 0.18h), Good ON‐time (+1.96 ± 0.18h), and ON‐time without any dyskinesia (+2.19 ± 0.26h) supported the sustained effect of ND0612 (*p* < 0.0001).

**Conclusion:** These data confirm the effectiveness of the ND0612 regimen in reducing OFF, improving ON, and reducing the transitions between them, compared to IR‐LD/CD.

**Disclosure:** OR, ALE, RAH, FS, RP, JJF, KK, AA, NG and AJE were investigators in the study and they or their institutions report fees from NeuroDerm. LS, NS, LA, NV and NL are employed by NeuroDerm. JP is employed by Mitsubishi Tanabe Pharma America, Inc.

## EPR‐053

### Patient experience and satisfaction with once‐daily deutetrabenazine for tardive dyskinesia: Age subgroup analysis

#### R. Jain^1^; M. Konings^2^; S. Thompson^3^; A. Yang^2^; S. Kotak^4^; P. Gandhi ^3^

##### ^1^Department of Psychiatry, Texas Tech University School of Medicine‐Permian Basin, Midland, USA; ^2^Teva Branded Pharmaceutical Products R&D, Inc., West Chester, USA; ^3^Teva Branded Pharmaceutical Products R&D, Inc., Parsippany, USA; ^4^Yorker Health Corp., Glen Rock, USA

**Background and aims:** Deutetrabenazine is approved by the United States Food and Drug Administration for the treatment of tardive dyskinesia (TD) and Huntington disease (HD)‐related chorea. This analysis explored patient‐reported ease of use, effectiveness, and satisfaction with once‐daily (QD) deutetrabenazine extended‐release tablets from participants with TD by age subgroup ( < 65 or ≥65 years).

**Methods:** This non‐interventional, prospective, cross‐sectional, institutional review board–approved survey included adults with TD or HD‐related chorea taking deutetrabenazine QD. Participants in Shared Solutions patient support program who completed/were due for their 12‐week nurse outreach phone call were eligible. Participants who consented received survey materials by mail/email.

**Results:** Among 209 participants with TD, 41.6% were aged ≥65 years. 73.6% of TD patients aged ≥65 years and 77.9% of those aged < 65 years reported that their extra movements were very much/much improved with deutetrabenazine QD. Patients reported that these improvements led to positive impacts across several quality‐of‐life domains (Table 1). Nearly all (≥65 years: 98.9%; < 65 years: 98.4%) reported that deutetrabenazine QD was very/somewhat easy to use. Satisfaction with deutetrabenazine QD was high; 86.2% of patients aged ≥65 years and 91.8% aged < 65 years reported overall satisfaction with deutetrabenazine QD, and most (≥65 years: 95.4%; < 65 years: 97.5%) strongly agreed/agreed that they will continue taking deutetrabenazine QD (Table 2).

**Figure:**
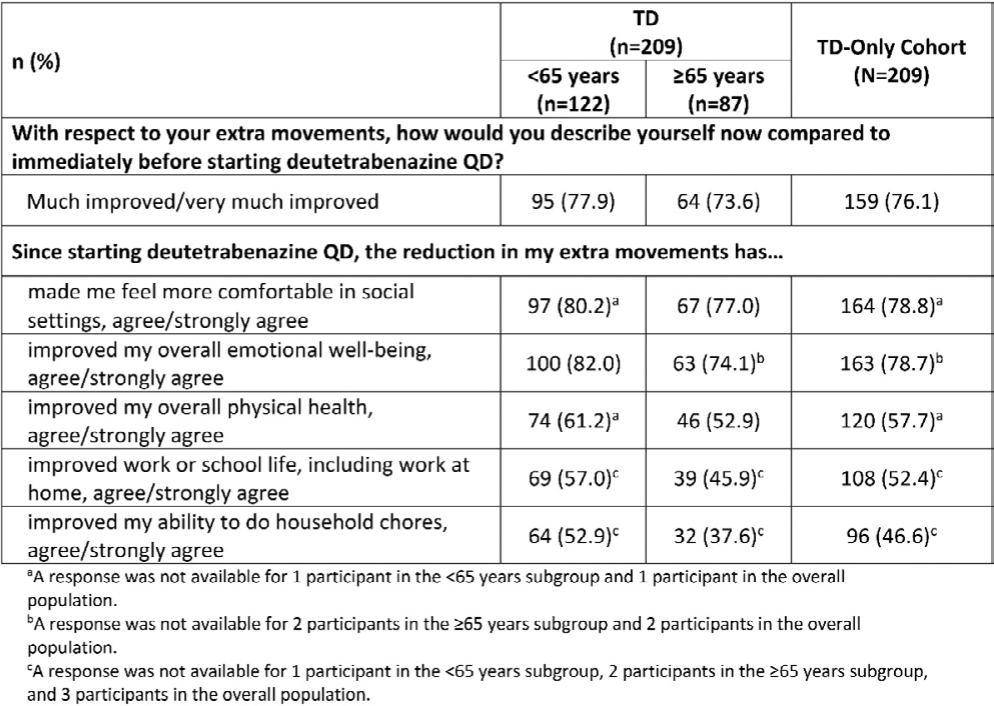
**TABLE 1** Participant‐reported improvement and effects on quality‐of‐life domains.

**Figure:**
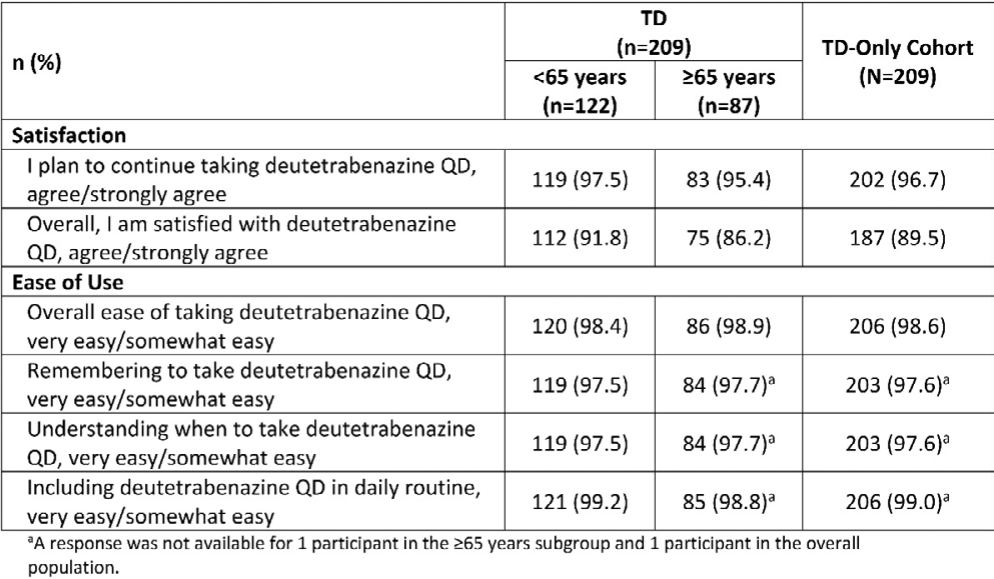
**TABLE 2** Participant‐reported satisfaction and ease of use.

**Conclusion:** Nearly all participants in both age subgroups reported overall satisfaction with deutetrabenazine QD, consistent with results in the overall population. Most participants reported improvements in their extra movements since starting deutetrabenazine QD, leading to positive impacts on several quality‐of‐life domains.

**Disclosure:** This study was funded by Teva Branded Pharmaceutical Products R&D, Inc.

## MS and related disorders 1

## EPR‐054

### Serum neurofilament light chain as a biomarker for treatment efficacy of extended interval dosing in multiple sclerosis

#### A. Konitsioti ^1^; F. Schweitzer^2^; W. Johannis^2^; M. Schroeter^1^; G. Fink^1^; C. Warnke^3^

##### ^1^Department of Neurology, University of Cologne, Faculty of Medicine and University Hospital Cologne, Cologne, Germany; ^2^Faculty of Medicine and University Hospital Cologne, Institute for Clinical Chemistry, Cologne, Germany; ^3^Department of Neurology, University Hospital Marburg, Germany

**Background and aims:** To evaluate serum neurofilament light chain (sNFL) levels as a biomarker for treatment efficacy in multiple sclerosis (MS) patients undergoing various disease‐modifying therapies (DMTs), focusing on extended interval dosing (EID) regimens.

**Methods:** We conducted a single‐center, cross‐sectional study involving 125 MS patients at the University Hospital Cologne from April to September 2024. Patients had been on therapy for over two years without relapses in the past three months. They were categorized by current DMT regimen: low‐efficacy DMTs (leDMTs; *N* = 8), natalizumab EID (every 8 weeks; *N* = 39), ofatumumab (*N* = 17), ocrelizumab standard interval dosing (SID; *N* = 20), ocrelizumab EID (every 9 months; *N* = 17), and a no‐DMT group (*N* = 19). sNFL levels were measured using an electrochemiluminescence assay.

**Results:** The cohort primarily consisted of females (59%), with a median age of 42.0 years, median therapy duration of 3.73 years, median sNFL of 1.300 pg/mL, median EDSS of 3.0 and median disease duration of 7.0 years. Ofatumumab and natalizumab EID were associated with the lowest sNFL levels. Significantly lower sNFL levels were observed in patients receiving natalizumab EID, ofatumumab, ocrelizumab SID and EID compared to the no‐DMT group, while no significant differences were noted among different DMT groups.

**Figure:**
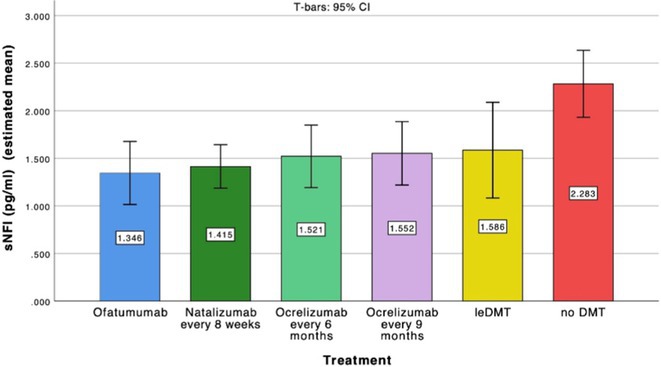
**FIGURE 1** Estimated mean of serum neurofilament light chain (sNFL) levels (in pg/ml) in multiple sclerosis (MS) patients receiving various disease‐modifying therapies (DMTs) with different dosing regimens, analyzed using Analysis of Covariance (ANCOVA).

**Conclusion:** In our cohort of MS patients receiving various monoclonal antibody therapies with different dosing regimens, sNFL levels showed no significant variation. Notably, EID regimens were not associated with elevated sNFL levels, indicating their potential to mitigate neuronal damage effectively. Further studies are warranted to investigate EID combined with sNFL monitoring as a strategy for de‐escalating long‐term immunotherapy in MS.

**Disclosure:** AK has received a study grant from Novartis. CW has received institutional support from Novartis, Alexion, Sanofi Genzyme, Biogen, Merck, Janssen, Bayer and Roche. He has received personal honoraria for teaching lectures from Biontech, Medpoint Medizinkommunikations, F&U confirm, Privatinstitut für Klinikmanagement,The Royal College Of Physicians, and for consulting from Wuesthoff+Wuesthoff and Bristows LLP. GRF, MS, WB and FS have nothing to disclose.

## EPR‐055

### Cognitive assessment of MOGAD and NMOSD patients: Preliminary results of a RIREMS group study

#### R. Orlandi^1^; V. Torri Clerici^2^; D. Ferraro^3^; R. Lanzillo^4^; M. Moccia^4^; A. Gallo^5^; V. Nociti^6^; S. Mariotto^1^; M. Calabrese^1^; P. Annovazzi^7^; P. Ragonese^8^; F. Buttari^9^; L. Lorefice^10^; C. Gasperini^11^; E. Cocco^10^; C. Solaro^12^; A. Gajofatto ^1^

##### ^1^Department of Neurosciences, Biomedicine and Movement Sciences, University of Verona, Verona, Italy; ^2^Neuroimmunology Unit, IRCCS Istituto Neurologico C. Besta, Milan, Italy; ^3^Neurology Unit, Department of Neurosciences, Azienda Ospedaliero‐Universitaria di Modena, Modena, Italy; ^4^Department of Neuroscience, Federico II University of Naples, Naples, Italy; ^5^Department of Advanced Medical and Surgical Sciences, University of Campania “L. Vanvitelli”, Naples, Italy; ^6^Multiple Sclerosis Center, Fondazione Policlinico Universitario “A. Gemelli” IRCCS, Rome, Italy; ^7^Neuroimmunology Unit‐Multiple Sclerosis Center, Gallarate Hospital, Gallarate, Italy; ^8^Neurology Unit, Department of Biomedicine, Neurosciences and Advanced Diagnostics, University of Palermo, Palermo Italy; ^9^Department of System Medicine, University of Tor Vergata, Rome, Italy; IRCCS Neuromed Institute, Pozzilli (IS), Italy, ^10^Multiple Sclerosis Center, Binaghi Hospital, Cagliari, Italy, ^11^MS Center, Department of Neuroscience, San Camillo Forlanini Hospital, Rome, Italy, ^12^Neurology Unit, Galliera Hospital, Genoa, Italy

**Background and aims:** Little is known about the impact of myelin oligodendrocyte glycoprotein antibody‐associated disease (MOGAD) and aquaporin‐4‐IgG‐seropositive neuromyelitis optica spectrum disorder (NMOSD‐AQP4+) on cognitive performance (CP). This ongoing multicenter longitudinal Italian study aimed to assess CP in adult MOGAD and NMOSD‐AQP4+ patients compared to relapsing remitting multiple sclerosis (RRMS) cases.

**Methods:** As of 15/01/2025, 21 MOGAD (7F, mean age 42y), 20 NMOSD‐AQP+ (18F, mean age 55y) and 19 RRMS patients (13F, mean age 37y) have been enrolled. CP is assessed using the Brief International Cognitive Assessment for Multiple Sclerosis (BICAMS) battery corrected for sex, age and education, including Symbol Digit Modalities Test (SDMT), California Verbal Learning Test (CVLT‐II) and Brief Visuospatial Memory Test‐Revised (BVMT‐R). Beck's Depression Inventory Scale (BDI‐II) and Fatigue Scale for Motor and Cognitive Functions (FSMC) are also administered. Assessment is performed at baseline and after 18+/‐6 months.

**Results:** At baseline, 5 (23.8%, CI:8.2‐47.2%) MOGAD, 3 (15.0%, CI:3.2‐37.9%) NMOSD and 3 (15.8%, CI:3.4‐39.6%) RRMS patients show impairment in one or more BICAMS tests. However, no significant differences in median values of any of the tests have been observed among the groups. Mean FSMC score is significantly higher in MS (55.4+/‐16.59) compared to MOGAD (38.3+/‐17.19) and NMOSD‐AQP4 patients (40.5+/‐22.07, *p* = 0.009). Follow‐up assessments are currently ongoing.

**Conclusion:** To our knowledge, this is the first study assessing CP in MOGAD and NMOSD using BICAMS battery. Our preliminary data suggest that patients with MOGAD and NMOSD could show impairment in cross‐sectional CP at similar rates of RRMS cases.

**Disclosure:** Massimiliano Calabrese received speaker honoraria from Biogen, Bristol Myers Squibb, Merck Serono, Novartis, and Roche and received research support from the Progressive MS Alliance and Italian Minister of Health and Biogen, Bristol Myers Squibb, Merck Serono, Novartis, and Roche. PA received honoraria for lecturing and participation in advisory boards, and/or travel expenses for attending congresses and meetings from Alexion, Almirall, Amgen, Biogen, BMS, Janssen, Lundbeck, Merck, Novartis, Roche, Sanofi‐Genzyme, Teva and Viatris.

## EPR‐056

### Placental and breastmilk transfer of ocrelizumab from women with multiple sclerosis to infants: MINORE and SOPRANINO

#### C. Oreja‐Guevara ^1^; S. Vukusic^2^; R. Bove^3^; A. Shah^4^; E. Graham^5^; T. McElrath^6^; C. Pietrasanta^7^; R. Dobson^8^; E. Maillart^9^; D. Jacobs^10^; H. Kletzl^11^; A. Kazlauskaite^11^; D. Zecevic^11^; C. Raposo^11^; L. Craveiro^11^; C. Lin^12^; N. Pasquarelli^11^; K. Hellwig^13^

##### ^1^Neurology, Hospital Clínico San Carlos, IdISSC, Madrid, Spain; ^2^Service de Neurologie et Sclérose en Plaques, Fondation Eugène Devic EDMUS contre la Sclérose en Plaques, Hôpital Neurologique Pierre Wertheimer, Lyon, France; ^3^Department of Neurology, UCSF Weill Institute for Neurosciences, University of California San Francisco, San Francisco, USA; ^4^Department of Neurology, Rocky Mountain MS Center, University of Colorado School of Medicine, Westminster, USA; ^5^Multiple Sclerosis and Neuroimmunology, Northwestern University, Chicago, USA; ^6^Division of Maternal‐Fetal Medicine, Brigham and Women's Hospital, Harvard Medical School, Boston, USA; ^7^Department of Clinical Sciences and Community Health, University of Milan, Milan, Italy; ^8^Centre for Preventive Neurology, Wolfson Institute of Population Health, Queen Mary University of London, London, UK; ^9^Neurology Department Hôpital de la Pitié Salpêtrière, Assistance Publique des Hôpitaux de Paris, Paris, France, ^10^Department of Neurology, Perelman School of Medicine at the Hospital of the University of Pennsylvania, Philadelphia, USA, ^11^F. Hoffmann‐La Roche Ltd, Basel, Switzerland, ^12^Roche Products Ltd, Welwyn Garden City, UK, ^13^Katholisches Klinikum Bochum, St. Josef Hospital, Universitätsklinikum, Bochum, Germany

**Background and aims:** Conventional multiple sclerosis (MS) pregnancy and breastfeeding management focuses on infant safety, but discontinuing disease‐modifying therapy increases the mother's risk of disease activity. Ocrelizumab (OCR) labeling advises contraception during treatment through 4 months post administration, but pregnancies may still occur. Prospective trials in women with MS assessed OCR transplacental (MINORE, NCT04998851) or breastmilk transfer (SOPRANINO, NCT04998851), and effects on infant B‐cell levels.

**Methods:** MINORE enrolled 35 pregnant women with MS (gestational week [W] ≤30) whose last OCR infusion occurred ≤6 months prior to the last menstrual period or during the first trimester. Primary endpoint was proportion of infants with B‐cell levels below lower limit of normal (LLN) at W6 of life. SOPRANINO enrolled 13 breastfeeding women receiving OCR (W2–24 at first postpartum infusion) and their infants. Coprimary endpoints were proportion of infants with B‐cell levels below LLN 30 days post‐infusion and OCR average daily infant dose over the 60 days post‐infusion.

**Results:** OCR was undetectable in most infant serum at birth in umbilical cord (33/35, 94.3%) and at W6 (32/33, 97.0%) (MINORE; Fig 1). OCR levels in breastmilk were negligible and undetectable in infant serum at 30 days post infusion (SOPRANINO). All infant B‐cell levels exceeded age‐specific LLN in MINORE (34/34) and SOPRANINO (10/10) (Fig 2). Maternal adverse events were typical of peripartum and the established OCR safety profile; infant adverse events were typical of infancy.

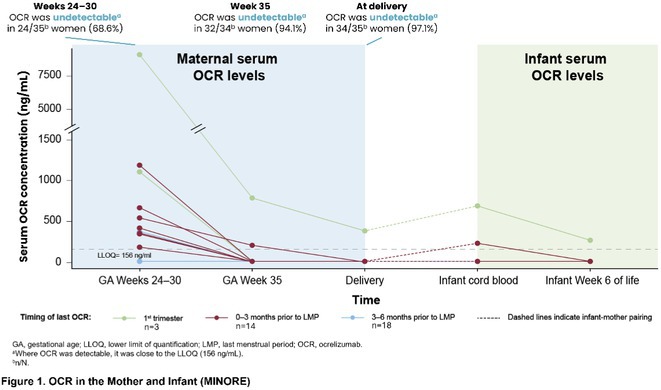

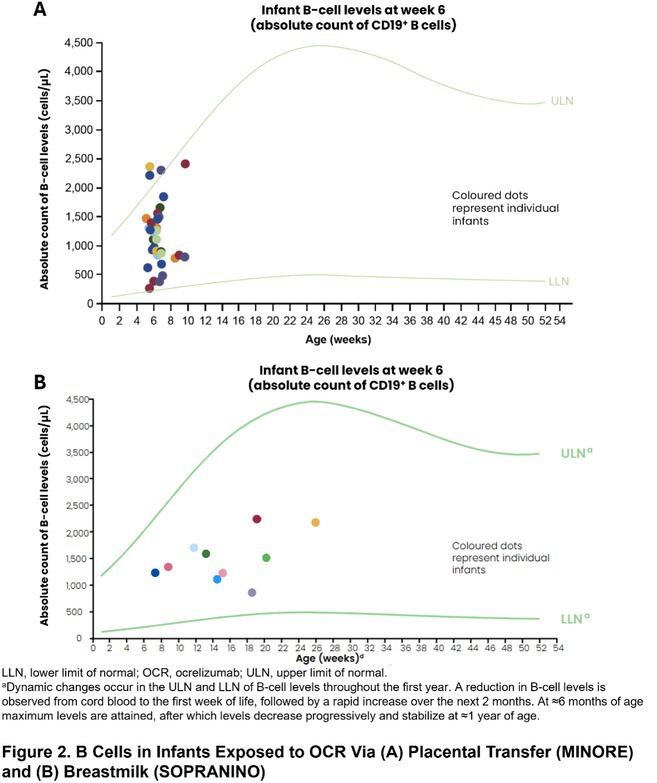

**Conclusion:** Pregnancy planning and breastfeeding are compatible with OCR treatment and provide an evidence basis for clinical management of women with MS.

**Disclosure:** Celia Oreja‐Guevara, Sandra Vukusic, Riley Bove, Anna Shah, Edith L. Graham, Thomas McElrath, Carlo Pietrasanta, Ruth Dobson, Heidemarie Kletzl, Agne Kazlauskaite, Dusanka Zecevic, Catarina Raposo, Licinio Craveiro, Chien‐Ju Lin, Noemi Pasquarelli and Kerstin Hellwig have disclosures. Elisabeth Maillart and Dina Jacobs have nothing to disclose. Full disclosures for each author will be provided at time of the presentation.

## EPR‐057

### Serum neurofilament light chain as a predictor of clinical and radiological outcomes: A post‐hoc analysis of MAGNIFY‐MS

#### Klaus Schmierer^1,2^; Heinz Wiendl^3^; Nicola De Stefano^4^; Patrick Vermersch^5^; Frederik Barkhof^6,7^; Xavier Montalban^8^; Anat Achiron^9,10^; Suzanne Hodgkinson^11^; Andrew Chan^12^; Alexandre Prat^13^; Letizia Leocani ^14–16^; Finn Sellebjerg^17,18^; Jens Kuhle^19^; Axel Nolting^20^; Anita Chudecka^21^; Lidia Gardner^22^; Tobias Derfuss^23^

##### ^1^The Blizard Institute, Centre for Neuroscience, Surgery and Trauma, Barts and The London School of Medicine & Dentistry, Queen Mary University of London, London, UK; ^2^Clinical Board Medicine (Neuroscience), The Royal London Hospital, Barts Health NHS Trust, London, UK; ^3^Department of Neurology with Institute of Translational Neurology, University of Münster, Münster, Germany; ^4^Department of Medicine, Surgery and Neuroscience, University of Siena, Siena, Italy; ^5^Univ. Lille, Inserm U1172 LilNCog, CHU Lille, FHU Precise, Lille, France; ^6^Department of Radiology and Nuclear Medicine, Amsterdam UMC, Vrije Universiteit, Amsterdam, the Netherlands; ^7^Queen Square Institute of Neurology and Centre for Medical Image Computing, University College London, London, UK; ^8^Department of Neurology‐Neuroimmunology, Centre d’Esclerosi Múltiple de Catalunya (Cemcat), Hospital Universitario Vall d’Hebron, Barcelona, Spain; ^9^Multiple Sclerosis Center, Sheba Academic Medical Center, Ramat Gan, Israel; ^10^Faculty of Medicine, Tel‐Aviv University, Israel; ^11^Ingham Institute for Applied Medical Research, University of New South Wales Medicine and Liverpool Hospital, Sydney, NSW, Australia; ^12^Department of Neurology, Inselspital, Bern University Hospital, University of Bern, Bern, Switzerland; ^13^Department of Neurosciences, Université de Montréal, Montréal, QC, Canada; ^14^University Vita‐Salute San Raffaele, Milan, Italy; ^15^Scientific Institute IRCCS San Raffaele, Milan, Italy; ^16^Department of Neurorehabilitation Science, Casa di Cura Igea, Milan, Italy; ^17^Danish MS Center, Department of Neurology, Copenhagen University Hospital ‐ Rigshospitalet, Glostrup, Denmark; ^18^Department of Clinical Medicine, University of Copenhagen, Copenhagen, Denmark; ^19^MS Centre and Research Center for Clinical Neuroimmunology and Neuroscience (RC2NB), Departments of Neurology, Biomedicine and Clinical Research, University Hospital and University of Basel, Basel, Switzerland; ^20^Merck Healthcare KGaA, Darmstadt, Germany; 21Cytel, Inc., Geneva, Switzerland; ^22^EMD Serono Research & Development Institute, Inc., Billerica, USA, an affiliate of Merck KGaA; ^23^Department of Neurology, University Hospital Basel, Basel, Switzerland

**Background and aims:** Serum neurofilament light chain (sNfL) is a biomarker reflecting neuroaxonal damage in multiple sclerosis (MS). We investigated associations of sNfL with radiological/clinical endpoints in cladribine tablets (CladT)‐treated participants with relapsing MS in 2‐year MAGNIFY‐MS study (NCT03364036).

**Methods:** sNfL percentiles and Z‐scores were derived based on a large reference dataset (Benkert et al., Lancet Neurol. 2022; 21:246–257). Changes in sNfL Z‐score at month (M)12 and M24 were calculated versus baseline (BL). Associations between magnetic resonance imaging (MRI) parameters and Z‐scores were explored using a multivariable linear regression model for repeated measures. Logistic regression was used for prediction analyses of no evidence of disease activity (NEDA‐3) and no evidence of progression or active disease (NEPAD). Subgroup analysis: BL sNfL Z‐score ≤1.5 and > 1.5.

**Results:** Of 270 participants, 180 (66.7%) were female, and 118 (43.7%) aged > 40 years. Mean sNfL Z‐scores decreased after first CladT dose in most participants and remained stable until study end (Table 1). sNfL Z‐score ≤1.5 subgroup: mean sNfL Z‐scores decreased close to 0; sNfL Z‐score > 1.5 subgroup: substantial decrease observed (Table 1). Higher BL sNfL Z‐scores predicted more pronounced decrease in future T1 gadolinium‐enhancing and active T2 lesion load between BL‐M12: estimated coefficient of change ‐0.40, *p* = 0.0005 and ‐4.84, *p* = 0.0002, respectively. BL sNfL Z‐scores could predict NEDA‐3/NEPAD at M12 (Table 2). M12 sNfL Z‐scores were not predictive for M24 results.

**Figure:**
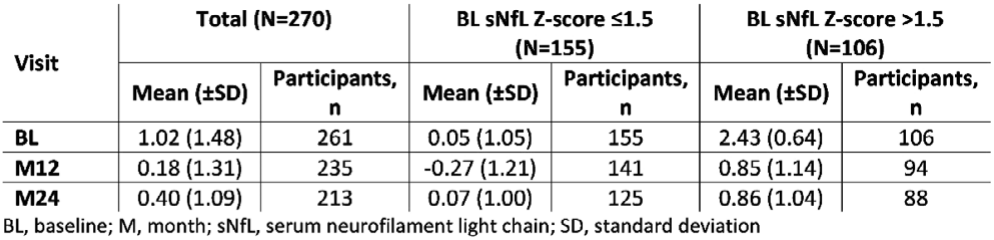
**TABLE 1** sNfL Z‐scores over the course of the study.

**Figure:**
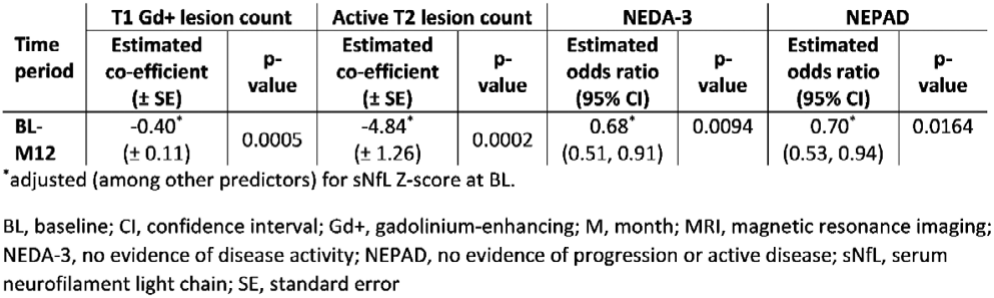
**TABLE 2** Effect of sNfL Z‐scores on MRI parameters and clinical outcomes.

**Conclusion:** CladT reduced sNfL levels in study participants, highlighting its neuroprotective effects. BL sNfL Z‐scores can predict MRI activity, NEDA‐3, and NEPAD in the first year of treatment.

**Disclosure:** **KS** has received research support, through Queen Mary University of London, from Biogen, Merck, Novartis, and Sandoz; speaking honoraria from, and/or served in an advisory role for, Biogen, EMD Serono Research & Development Institute, Inc., Billerica, MA, USA, an affiliate of Merck KGaA, Darmstadt, Germany, Merck, Neuraxpharm, Novartis, Roche, and Sanofi; and remuneration for teaching activities from AcadeMe and Medscape. **HW** is a member of scientific advisory boards/steering committees for Bayer, Biogen, Merck, Novartis, Roche, Sanofi, and Teva. He received speaker honoraria and travel support from Bayer, Biogen, CSL Behring, EMD Serono Research & Development Institute, Inc., Billerica, MA, USA, an affiliate of Merck KGaA, Fresenius Medical Care, Merck, Omniamed, Novartis, Sanofi, and Teva. He received compensation as a consultant from Biogen, Merck, Novartis, Omniamed, Roche, and Sanofi. He has received research support from Bayer, Biogen, Merck, Novartis, Sanofi, and Teva, as well as the German Ministry for Education and Research (BMBF), German Research Foundation (DFG), Else Kröner Fresenius Foundation, Fresenius Foundation, Hertie Foundation, NRW Ministry of Education and Research, Interdisciplinary Center for Clinical Studies (IZKF) Münster, and RE Children’s Foundation. **NDS** has received honoraria from Biogen, Celgene (Bristol Myers Squibb), Genzyme, Immunic, EMD Serono Research & Development Institute, Inc., Billerica, MA, USA, an affiliate of Merck KGaA, Novartis, Roche, and Teva for consulting services, speaking, and travel support. He serves on advisory boards for Biogen, Genzyme, Immunic, Merck, Novartis, and Roche, and has received research grant support from the Italian MS Society. **PV** has received honoraria or consulting fees from AB Science, Ad Scientiam, Biogen, Celgene (Bristol Myers Squibb), Imcyse, Janssen (J&J), Merck, Novartis, Roche, Sanofi, and Teva; and research support from Novartis, Roche, and Sanofi. **FB** is supported by the NIHR Biomedical Research Centre at UCLH and is a steering committee or Data Safety Monitoring Board member for ATRI/ACTC, Biogen, Merck, and Prothena. He is a consultant for Celltrion, Combinostics, IXICO, Janssen (J&J), Merck, Rewind Therapeutics, and Roche. Research agreements with Biogen, GE Healthcare, Merck, and Roche. Co‐founder and shareholder of Queen Square Analytics Ltd. **XM** has received compensation for lecture honoraria and travel expenses, participation in scientific meetings, clinical trial steering committee membership, or clinical advisory board participation in recent years from Abbvie, Actelion, Alexion, Bial PD, Biogen, Bristol‐Myers Squibb/Celgene, EMD Serono Research & Development Institute, Inc., Billerica, MA, USA, an affiliate of Merck KGaA, Genzyme, Hoffmann‐La Roche, Immunic Therapeutics, Janssen Pharmaceuticals, Medday, Medscape, Merck, Mylan, Nervgen, Neuraxpharm, Novartis, Peervoice, Samsung‐Biosys, Sandoz, Sanofi‐Genzyme, Teva Pharmaceutical, TG Therapeutics, Excemed, ECTRIMS, MSIF, and NMSS or any of their affiliates. **AA** has received over the last 5 years honoraria or consulting fees for participating in advisory boards related to clinical trial design, trial steering committees, and data and safety monitoring committees from Biogen, Bristol Myers Squibb, Merck, Novartis, Roche, and Sanofi; and research support for investigator‐initiated trials and MS patients’ benefits activities from Biogen, Bristol Myers Squibb, Merck, Novartis, Roche, and Sanofi. **SH** serves on advisory boards for Bayer, Biogen, Merck, Novartis, Roche, and Sanofi. She has received money for travel and speaker honoraria from Bayer, Biogen, Merck, Novartis, Roche, and Sanofi. **ACha** has received speakers’/board honoraria from Actelion (Janssen/J&J), Almirall, Bayer, Biogen, Celgene (Bristol Myers Squibb), Merck, Novartis, Roche, Sanofi, and Teva, all for hospital research funds. He received research support from Biogen, Sanofi, and UCB, the European Union, and the Swiss National Foundation. He serves as associate editor of the European Journal of Neurology, on the editorial board for Clinical and Translational Neuroscience, and as topic editor for the Journal of International Medical Research. **AP** has received speaking honoraria and travel expenses for participation in scientific meetings, has been a steering committee member of clinical trials or participated in advisory boards of clinical trials in the past years, and/or received operating grants from Alexion, Bayer, Biogen, Celgene (Bristol Myers Squibb), EMD Serono Research & Development Institute, Inc., Billerica, MA, USA, an affiliate of Merck KGaA, Novartis, Roche, Sanofi, and Teva. **LL** has received honoraria for consulting services or speaking activities from Bristol Myers Squibb, Janssen‐Cilag, Merck, Novartis, and Roche; and research support from Merck, and Novartis. **FS** has served on scientific advisory boards, been on the steering committees of clinical trials, served as a consultant, received support for congress participation, received speaker honoraria, or received research support for his laboratory from Biogen, Celgene (Bristol Myers Squibb), EMD Serono Research & Development Institute, Inc., Billerica, MA, USA, an affiliate of Merck KGaA, Merck, Novartis, Roche, Sanofi, and Teva. **JK** has received speaker fees, research support, travel support, and/or served on advisory boards by Swiss MS Society, Swiss National Research Foundation (320030_189140/1), University of Basel, Progressive MS Alliance, Bayer, Biogen, Celgene (Bristol Myers Squibb), Merck, Novartis, Octave Bioscience, Roche, and Sanofi. **AN** is an employee of Merck Healthcare KGaA, Darmstadt, Germany. **AChu** is an employee of Cytel, Inc., Geneva Branch, Switzerland, funded by Merck KGaA, Darmstadt, Germany to perform statistical analyses for this study.**LG** is an employee of EMD Serono Research & Development Institute, Inc., Billerica, MA, USA, an affiliate of Merck KGaA. **TD** serves on scientific advisory boards for Actelion (Janssen/J&J), Bayer, Biogen, Celgene (Bristol Myers Squibb), GeNeuro, MedDay, Merck, Mitsubishi Pharma, Novartis, Roche, and Sanofi; has received funding for travel and/or speaker honoraria from Biogen, Merck, Novartis, Roche, and Sanofi; and receives research support from Actelion, the European Union, Novartis, Roche, the Swiss MS Society, and the Swiss National Foundation.**Funding:** This study was sponsored by Merck (CrossRef Funder ID: 10.13039/100009945). Claire Snaith of inScience Communications, Springer Healthcare Ltd., UK, provided medical writing support, which was funded and supported by Merck, in accordance with the Good Publication Practice 2022 Guidelines.

## EPR‐058

### Peripartum disease activity in women with multiple sclerosis: Ocrelizumab clinical trials and a noninterventional study

#### M. Buttmann ^1^; T. Ziemssen^2^; R. Bove^3^; K. Hellwig^4^; A. Gondos^5^; A. Siadimas^5^; S. Walter^6^; C. Lin^7^; N. Pasquarelli^5^; S. Vukusic^8^

##### ^1^Caritas Hospital, Bad Mergentheim, Germany; ^2^Center of Clinical Neuroscience, Carl Gustav Carus University Clinic, Dresden, Germany; ^3^Department of Neurology, UCSF Weill Institute for Neurosciences, University of California San Francisco, San Francisco, USA; ^4^Katholisches Klinikum Bochum, St. Josef Hospital, Universitätsklinikum, Bochum, Germany; ^5^F. Hoffmann‐La Roche Ltd, Basel, Switzerland; ^6^Roche Pharma AG, Grenzach‐Wyhlen, Germany; ^7^Roche Products Ltd, Welwyn Garden City, UK; ^8^Service de Neurologie et Sclérose en Plaques, Fondation Eugène Devic EDMUS contre la Sclérose en Plaques, Hôpital Neurologique Pierre Wertheimer, Lyon, France

**Background and aims:** Pre‐pregnancy ocrelizumab (OCR) use in women with multiple sclerosis (MS) may help control disease activity during pregnancy and postpartum. Evidence on MS relapses around pregnancy from OCR interventional clinical trials (ICTs) can be complemented by real‐world data from CONFIDENCE (ML39632, EUPAS22951), a noninterventional, prospective, multicenter study assessing long‐term safety of OCR.

**Methods:** Women with MS across 13 OCR ICTs (as of Nov 2023) and CONFIDENCE (as of Nov 2024) who became pregnant (despite protocol requirements in ICTs) and had live births were included. Pre‐pregnancy (up to 1 year), pregnancy and postpartum (up to 1 year) periods were analyzed, using annualized relapse rate (ARR) as the primary measure of disease activity.

**Results:** A total of 178 women with live births were analyzed, including 103 from ICTs and 75 from CONFIDENCE. The CONFIDENCE cohort was older and more active prior to pregnancy, with a longer MS disease duration, lower proportions of treatment‐naive individuals and shorter duration of pre‐pregnancy OCR (Table 1). CONFIDENCE participants resumed OCR postpartum at higher rates than ICT participants (48% vs. 31%). ARRs (95% CI) before, during and after pregnancy were 0.06 (0.02–0.14), 0.03 (0.00–0.09) and 0.04 (0.01–0.14) for the ICT cohort and 0.16 (0.08–0.29), 0.02 (0.00–0.11) and 0.17 (0.08–0.33) for the CONFIDENCE cohort, respectively (Table 2).

**Figure:**
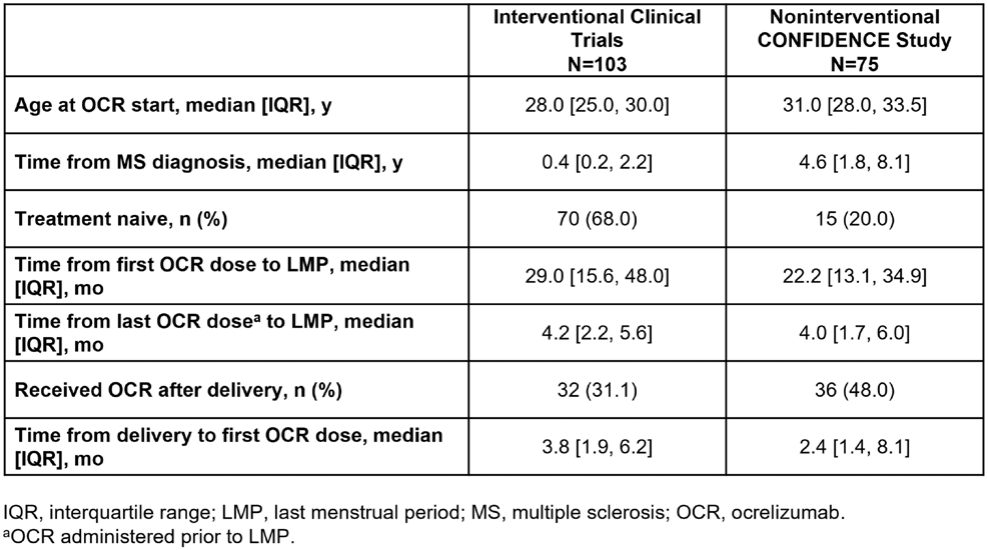
**TABLE 1** Participant Characteristics.

**Figure:**
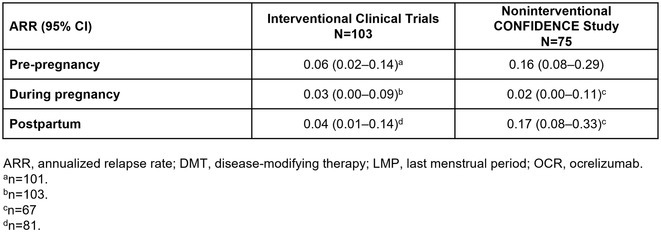
**TABLE 2** ARRs in Peripregnancy Periods.

**Conclusion:** Low peri‐pregnancy ARRs were observed across a broad and heterogenous population of childbearing women with MS. These data provide evidence on the pre‐pregnancy use of OCR to maintain disease control during pregnancy and postpartum.

**Disclosure:** Mathias Buttmann, Tjalf Ziemssen, Riley Bove, Kerstin Hellwig, Adam Gondos, Athanasios Siadimas, Saskia Walter, Chien‐Ju Lin, Noemi Pasquarelli and Sandra Vukusic have disclosures. Full disclosures for each author will be provided at the time of presentation.

## EPR‐059

### Ocrelizumab treatment in multiple sclerosis: 11‐Year efficacy and safety clinical trial data

#### M. Buttmann ^1^; M. Buttmann^2^; X. Montalban^3^; G. Giovannoni^4^; M. Filippi^5^; H. Schneble^6^; L. Craveiro^6^; N. Pasquarelli^6^; C. Chognot^6^; Q. Wang^6^; T. Derfuss^7^

##### ^1^Department of Neurology, Caritas Hospital, 97980 Bad Mergentheim, Germany; ^2^Medical Faculty, University of Würzburg, Würzburg, Germany; ^3^Department of Neurology and Centre d'Esclerosi Múltiple de Catalunya (Cemcat), Hospital Universitari Vall d'Hebron, Barcelona, Spain; ^4^Queen Mary University of London, London, UK; ^5^Neurology Unit, Neurorehabilitation Unit, and Neurophysiology Service, IRCCS San Raffaele Scientific Institute, and Vita‐Salute San Raffaele University, Milan, Italy; ^6^F. Hoffmann‐La Roche Ltd, Basel, Switzerland; ^7^Department of Neurology, University Hospital Basel, University of Basel, Basel, Switzerland

**Background and aims:** Ocrelizumab (OCR) is approved for treating adult patients with relapsing multiple sclerosis (pwRMS) and primary progressive MS (pwPPMS). This study evaluated the long‐term efficacy and safety of OCR up to 11 years through clinical trials (CTs) and their open‐label extension (OLE) periods (up to November 2023).

**Methods:** Efficacy and safety outcomes were reported for pwMS treated with OCR in ongoing or completed CTs and their OLE follow‐ups. Disability progression was assessed through 48‐week confirmed disability progression (48W‐CDP) on the Expanded Disability Status Scale (EDSS) and composite CDP. Rates per 100 patient years (PY) were recorded for adverse events (AEs), serious AEs, non‐serious and serious infections.

**Results:** After 11 years on OCR, 91.7% of pwRMS and 79.5% of pwPPMS did not require a walking aid or wheelchair, respectively; 17.8% of pwPPMS remained free from composite CDP. Early OCR treatment significantly reduced risks of disability milestones compared with delayed treatment. Over an > 11‐year follow‐up period in clinical trials (with > 60% of patients who received > = 8 doses of OCR), no new safety concerns were observed. Infections were mostly of urinary and respiratory nature. Most patients with at least one recurrent (non‐serious and serious) infection had one recurrency.

**Figure:**
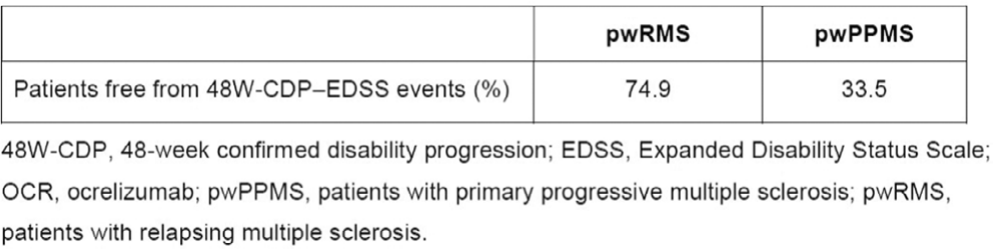
**TABLE 1** 48W‐CDP–EDSS over 11 years with OCR.

**Figure:**
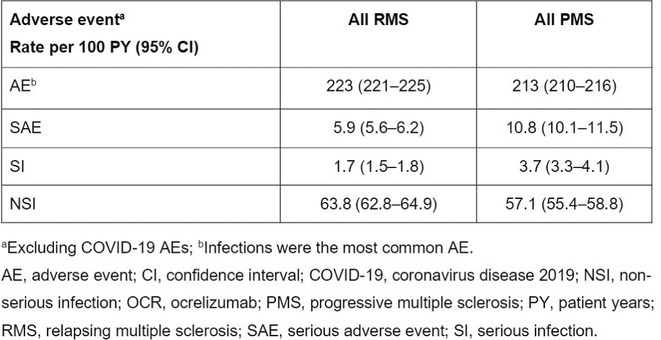
**TABLE 2** OCR overall safety profile.

**Conclusion:** Ocrelizumab demonstrated a stable and favorable long‐term benefit–risk profile over 11 years. Disability progression and rates of AEs remained stable, supporting its continued use in treating MS.

**Disclosure:** Sponsored by Roche; editorial assistance by Nucleus Global. MB: honoraria (Biogen, BMS, Das Fortbildungskolleg, Florian Schmitz Kommunikation, Janssen, Merck, Novartis, RG Ärztefortbildung, Roche, Sandoz, Sanofi, Teva, Viatris). XM: honoraria, consultancy (AbbVie, Actelion, Alexion, Biogen, BMS/Celgene, EMD Serono, Roche, Immunic, Janssen, MedDay, Merck, Mylan, NervGen, Novartis, Sandoz, Sanofi‐Genzyme, Teva, TG Therapeutics, Exemed, MSIF, NMSS). GG: consultancy (Roche, AbbVie, Aslan, Atara Biotherapeutics, Biogen, BMS/‐Celgene, GSK, GW Pharma, Janssen/J&J, Japanese Tobacco, Jazz Pharma, LifNano, Merck, Merck KGaA/EMD Serono, Moderna, Novartis, Sanofi‐Genzyme, Teva). MF: Editor (Journal of Neurology, Human Brain Mapping, Radiology, Neurological Sciences) consultancy (Alexion, Almirall, Bayer, Biogen, BMS, Celgene, Chiesi Italia SpA, Eli Lilly, Genzyme, Janssen, Merck, Merck‐Serono, Neopharmed Gentili, Novartis, Novo Nordisk, Roche, Sanofi, Sanofi‐Genzyme, Takeda, Teva) educ. events/research (Biogen, Biogen Idec., Celgene, BMS, Eli Lilly, Merck, Merck‐Serono, Novartis, Roche, Italian Ministry of Health, Fondazione Italiana Sclerosi Multipla, ARiSLA, Sanofi‐Genzyme). HMS, NP, CC, LC, QW: employees (Roche). TD: consultancy (Actelion, Alexion, Biogen, Celgene, Genzyme, GeNeuro, Merck, Mitsubishi Pharma, Novartis, Roche, Octapharma, MedDay) honoraria/research (Alexion, Biogen, Genzyme, Merck, Novartis, Roche, Merck‐Serono, the Swiss MS Society and National Foundation).

## EPR‐060

### Safety and efficacy of frexalimab in relapsing multiple sclerosis: 2‐Year results from phase 2 open‐label extension

#### P. Vermersch ^1^; C. Granziera^2^; Y. Mao‐Draayer^3^; G. Cutter^4^; O. Kalbus^5^; I. Staikov^6^; M. Dufek^7^; X. Montalban^8^; S. Krieger^9^; S. Saubadu^10^; X. Luo^11^; B. Smyth^11^; B. Djukic^12^; P. Truffinet^10^; E. Wallstroem^12^; G. Giovannoni^13^

##### ^1^University of Lille, Inserm U1172, Lille Neuroscience and Cognition, CHU Lille, FHU Precise, Lille, France; ^2^Translational Imaging in Neurology (ThINk) Basel, Department of Biomedical Engineering, Faculty of Medicine; Neurologic Clinic and Policlinic, MS Center and RC2NB, University Hospital Basel and University of Basel, Basel, Switzerland; ^3^Autoimmunity Center of Excellence, Oklahoma Medical Research Foundation, Oklahoma City, USA; ^4^Department of Biostatistics, UAB School of Public Health, Birmingham, USA; ^5^Department of Neurology, Dnipro State Medical University, Dnipro, Ukraine; ^6^Clinic of Neurology and Sleep Medicine, Acibadem City Clinic University Hospital Tokuda, Sofia, Bulgaria; ^7^First Department of Neurology, St. Anne's University Hospital, Faculty of Medicine, Masaryk University, Brno, Czechia; ^8^Multiple Sclerosis Centre of Catalonia, Department of Neurology, Vall d'Hebron University Hospital, Barcelona, Spain; ^9^Corinne Goldsmith Dickinson Center for Multiple Sclerosis, Icahn School of Medicine at Mount Sinai, New York, USA, ^10^Sanofi, Gentilly, France, ^11^Sanofi, Bridgewater, USA, ^12^Sanofi, Cambridge, USA, ^13^Queen Mary University of London, London, UK

**Background and aims:** Frexalimab, a second‐generation anti‐CD40L antibody, blocks the CD40/CD40L pathway, which is important in regulating adaptive and innate immunity. During the 12‐week (W) double‐blind‐period of the phase‐2 trial (NCT04879628) in participants with relapsing multiple sclerosis (pwRMS), frexalimab was well‐tolerated and efficacious, with the frexalimab‐1200mg/intravenous (IV) arm showing an 89% reduction in new gadolinium‐enhancing (Gd+) T1‐lesions versus placebo. Here, we report W96 results in open‐label extension (OLE).

**Figure:**
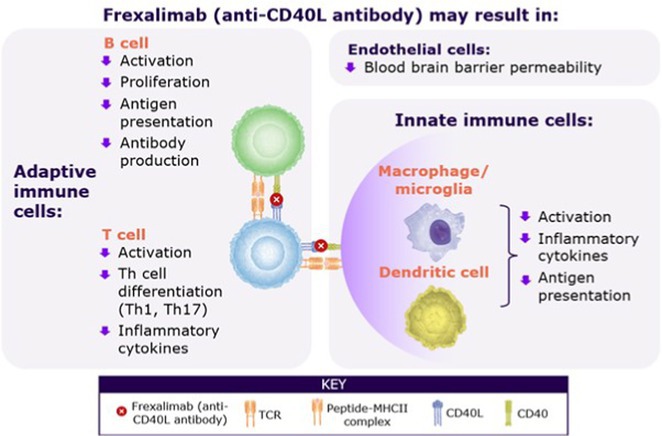
**FIGURE 1** Proposed mechanism of action of frexalimab (anti‐CD40L antibody) in multiple sclerosis

**Methods:** 129 participants were randomized (4:4:1:1) to frexalimab‐1200mg/IV every‐4‐weeks (q4w) or frexalimab‐300mg/subcutaneous (SC) q2w or matching placebo. At W12, participants receiving placebo switched to frexalimab. During OLE, SC dose was increased to 1800‐mg q4w, resulting in similar frexalimab exposure as with frexalimab‐1200mg/IV q4w dose; 36/57 participants in SC arms received this high‐dose prior to W96 MRI. Key endpoints include safety and efficacy (Gd+ T1‐lesions, new/enlarging T2‐lesions, annualized relapse rate [ARR]).

**Results:** 106 participants (82%) remained on treatment at W96. Total number of Gd+ T1‐lesions (mean [SD]) remained low in participants who continued receiving frexalimab and who switched to frexalimab at W12 (IV arms: frexalimab‐1200mg/IV: 0.1 [0.5], placebo‐IV/frexalimab‐1200mg/IV: 0.1 [0.3]; all SC arms: 0.4 or below). New/enlarging T2‐lesion monthly count remained low with frexalimab‐1200mg/IV through W96. ARR (baseline–W96) was low (0.08 [95% CI, 0.03–0.18]) in the frexalimab‐1200mg/IV arm; 92% of participants were relapse‐free. Most common adverse events were nasopharyngitis, headache, and COVID‐19. Lymphocyte counts were stable over W96.

**Conclusion:** Frexalimab continues to show favorable safety and sustained reduction in disease activity in pwRMS through W96, supporting its further development in phase‐3 trials as a potential high‐efficacy, non‐lymphocyte‐depleting therapy.

**Disclosure:** PV: Janssen, Biogen, Sanofi, Novartis (NVS), Teva, Merck, Roche, Imcyse, ABSci, BMS‐Celgene (Cel), AdScientiam. CG: Actelion, NVS, Sanofi, GeNeuro, Roche, Siemens, Biogen, Teva, Merck, Janssen. YMD: Acorda, Bayer, Biogen, BMS‐Cel, Chugai, EMDSerono(Ser), Genentech (Gene)‐Roche, Horizon/Amgen, Janssen, NVS, Sanofi, Teva, NIAID, NIH NINDS, PCORI. GC: DSMB: APLT, AI, AMO, AZ, Avexis, Biolinerx, BrainStormCell, BMS‐Cel, CSLBehring, Galmed, GreenValley, Horizon, Immunic, Karuna, Mapi, Merck, MitsubishiTanabe, Opko, Prothena, NVS, REGN, Sanofi, Reata, Teva, NHLBI, UTSouthwestern, UPenn, VTI; Alexion, Antisense, Biogen, ClinTrialSol, Entelexo, Sanofi, Gene, GW, Immunic, Immunosis, KleinBuendel, Merck/Ser, NVS, Perception, Protalix, REGN, Roche, SAB, UAB, Pythagoras. OK: Sanofi, Roche, Gene, Merck, NVS, GeNeuro, BMS‐Cel, Mapi, VielaBio, Teva. IS: Sanofi, Ewo‐Biogen, Shire, Gedeon‐Richter, Teva, BI, Pfizer, Bayer, Roche, Mylan, Polpharma, Penumbra, Adapt, Merck, GL, Medochemie, NVS, Viatris, Nobel. MD: Sanofi. XM: Abbvie, Actelion, Alexion, Bial PD, Biogen, BMS‐Cel, EMD Ser, Genzyme, Roche, Immunic, Janssen, MedDay, Medscape, Merck, Mylan, Nervgen, Neurax, NVS, Peervoice, Samsung‐Biosys, Sandoz, Sanofi, Teva, TG, Excemed, ECTRIMS, MSIF, NMSS. SK: Biogen, EMDSer, Gene, Genzyme, Mallinckrodt, MedDay, NVS, Octave, Teva, TG. SS, XL, BS, BD, PT, EW: Sanofi Emp. GG: AbbVie, Actelion, Atara, Biogen, Canbex, Cel, EMD Ser, Japan Tobacco, Sanofi, Gene, GSK, GW, Merck, NVS, Roche, Synthon, Teva.

## EPR‐061

### Frexalimab reduces plasma neurofilament light chain in relapsing multiple sclerosis: Week 72 data from phase 2 trial

#### P. Vermersch ^1^; J. Kuhle^2^; B. Djukic^3^; S. Geertsen^3^; A. Shafer^3^; P. Truffinet^4^; G. Giovannoni^5^

##### ^1^Univ. Lille, Inserm U1172, Lille Neuroscience and Cognition, CHU Lille, FHU Precise, Lille, France; ^2^University Hospital Basel, Basel, Switzerland; ^3^Sanofi, USA; ^4^Sanofi, France; ^5^Queen Mary University of London, London, UK

**Background and aims:** Frexalimab, a second‐generation anti‐CD40L antibody, inhibits the CD40/CD40L pathway that regulates adaptive and innate immunity. During the double‐blind period of a phase‐2 trial (NCT04879628) in participants with relapsing multiple sclerosis (pwRMS), frexalimab showed an 89% reduction in new gadolinium‐enhancing T1‐lesions in the 1200mg/intravenous (IV) every‐4‐weeks (q4w) arm versus placebo at week (W) 12. This was accompanied by a reduction in neurofilament light chain (NfL), a biomarker of neuroaxonal damage. Treatment effect has been sustained in the open‐label extension (OLE). Here, we describe changes in NfL through W72.

**Methods:** 129 pwRMS were randomized (4:4:1:1) to frexalimab‐1200mg/IV q4w, frexalimab‐300mg/subcutaneous (SC) q2w, or matching placebos. After W12, participants receiving placebo switched to respective frexalimab arms and entered the OLE (*n* = 125). Plasma samples were collected from baseline through W72. NfL levels were measured using Quanterix Simoa® NF‐LIGHT™ assay and summarized as geometric means.

**Results:** 111/125 participants remained on treatment at W72. Baseline NfL levels (*n* = 122; geometric mean [SD]) were similar across groups (F = 0.10, *p* = 0.96): 11.8 [2.0], frexalimab‐1200mg/IV; 12.6 [1.8], frexalimab‐300mg/SC; 12.4 [1.9], placebo‐IV/frexalimab‐1200mg/IV; and 12.1 [1.8], placebo‐SC/frexalimab‐300mg/SC. At W72 (*n* = 106), NfL levels reduced to 7.3 [1.6], 7.2 [1.9], 9.4 [1.7], and 7.1 [1.8] pg/mL, respectively. In participants with both baseline and W72 data (*n* = 104), there was a 40%, 45%, 25%, and 40% reduction in NfL geometric mean over time, respectively. Associations between the reduction in NfL and changes in MRI endpoints (baseline–W72) will be presented at the meeting.

**Conclusion:** The observed reduction in NfL through W72 indicates that frexalimab markedly reduces neuroaxonal damage in pwRMS.

**Disclosure:** Patrick Vermersch: Received honoraria and/or consulting fees (Janssen, Biogen, Sanofi, Novartis, Teva, Merck, Roche, Imcyse, AB Science, BMS‐Celgene, and Ad Scientiam) and research support (Novartis, Sanofi, and Roche). Jens Kuhle: Speaker fees, research support, travel support, and/or served on advisory boards of the Swiss MS Society, Swiss National Research Foundation (320030_189140/1), University of Basel, Progressive MS Alliance, Bayer, Biogen, Celgene, Merck, Novartis, Octave Bioscience, Roche, and Sanofi. Biljana Djukic, Svend S. Geertsen, Andrea T. Shafer, Philippe Truffinet: Employees of Sanofi (may hold shares and/or stock options in the company). Gavin Giovannoni: Received compensation over the last five years for receiving research support or serving as a consultant or speaker (AbbVie, Actelion, Atara Bio, Biogen, Canbex, Celgene, EMD Serono, Japanese Tobacco, Sanofi, Genentech, GlaxoSmithKline, GW Pharma, Merck, Novartis, Roche, Synthon BV, and Teva).

## EPR‐062

### Pediatric MS: Phenotypic characterization and pubertal influences on disease activity from the Danish MS Registry

#### S. Pilotto ^1^; L. Pontieri^2^; H. Hvilsted Nielsen^3^; P. Vestergaard Rasmussen^4^; K. Bacher Svendsen^4^; R. Marie Jensen^5^; M. Blinkenberg^5^; A. Thormann^5^; E. Cocco^6^; M. Pugliatti^7^; M. Magyari^8^

##### ^1^Department of Medical Sciences and Public Health, University of Cagliari, Cagliari, Italy; ^2^The Danish Multiple Sclerosis Registry, Department of Neurology, Copenhagen University Hospital – Rigshospitalet Glostrup, 2600 Glostrup, Denmark; ^3^Department of Neurology, Odense University Hospital, Odense, Denmark; ^4^Department of Neurology, Aarhus University Hospital, Aarhus, Denmark; ^5^Department of Neurology, Copenhagen University Hospital Danish Multiple Sclerosis Research Center, Glostrup, Denmark; ^6^Multiple Sclerosis Center, Department of Medical Sciences and Public Health, University of Cagliari, Cagliari, Italy; ^7^Department of Neuroscience and Rehabilitation, University of Ferrara, Ferrara, Italy; Interdepartmental Research Center for Multiple Sclerosis and Other Inflammatory and Degenerative Disorders of the Nervous System, University of Ferrara, Ferrara, Italy; ^8^The Danish Multiple Sclerosis Registry and the Danish Multiple Sclerosis Center, Department of Neurology, Copenhagen University Hospital – Rigshospitalet Glostrup, Denmark; Department of Clinical Medicine, University of Copenhagen, Copenhagen, Denmark

**Background and aims:** Pediatric‐onset multiple sclerosis (POMS) constitutes ~5% of MS cases and presents distinct clinical and diagnostic challenges. Puberty, characterized by significant hormonal changes, may influence disease presentation, relapse rates, and long‐term outcomes. This study aimed to investigate the impact of pubertal stages on clinical characteristics, relapse activity and disability progression in POMS using data from the Danish MS Registry (DMSR).

**Methods:** A nationwide cohort of 185 POMS patients included and s categorized into pre‐pubertal ( < 11 years), peri‐pubertal (11–14 years), and post‐pubertal ( > 14 years) onset groups. Demographics, presenting symptoms, MRI findings, relapse rates, and Expanded Disability Status Scale (EDSS) scores were compared. Patients transitioning between pubertal stages (*n* = 54) were analyzed longitudinally for relapse rate.

**Results:** Pre‐pubertal onset was associated with severe symptoms (cerebellar involvement, *p* = 0.042), greater lesion burden, higher 10‐year disability (EDSS median = 3.75, *p* = 0.039), and lower relapse rates (ARR = 0.200). Male sex reduced relapse rates (*p* = 0.013). Female‐to‐male ratio increased from 1:1 pre‐puberty to ~2:1 after puberty. Pre‐pubertal patients transitioning to peri‐ or post‐puberty showed increasing relapse rates, peaking during peri‐puberty (ARR = 0.302).

**Figure:**
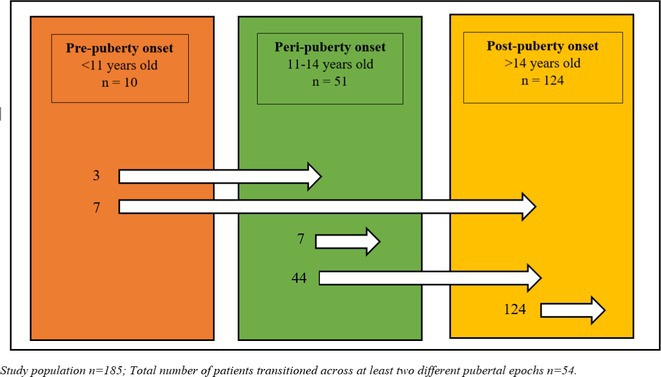
**FIGURE 1** Pre‐, Peri‐ and Post puberty epochs by age of disease onset

**Figure:**
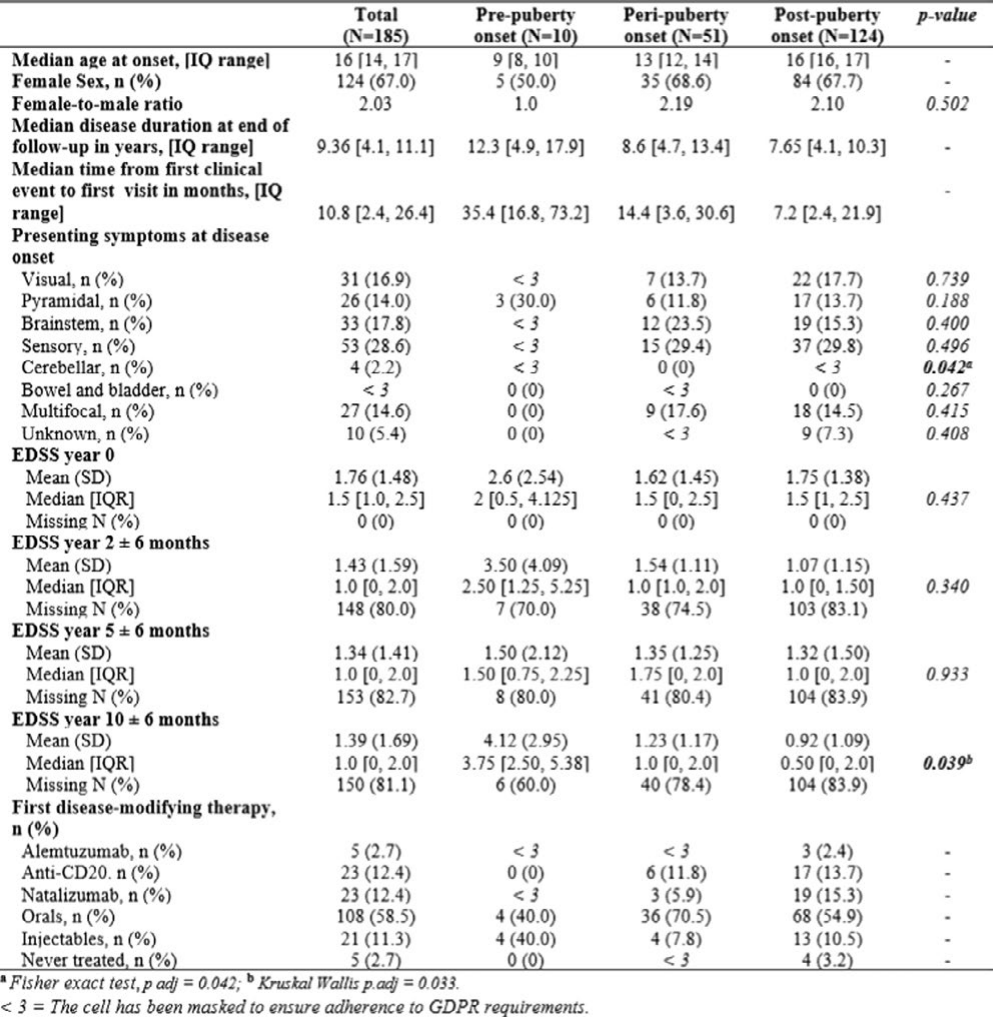
**TABLE 1** Relapse Risk and Key Determinants between Pubertal Groups compared to Post‐puberty epoch.

**Conclusion:** Puberty significantly modulates disease course in POMS, emphasizing the need for early, sex‐specific interventions, proactive monitoring, and further exploration of hormonal influences on disease progression and treatment response.

**Disclosure:** SP has received travel grants from Biogen, Teva, Bristol Myers Squibb and compensations for research activities from Fondazione Italiana Sclerosi Multipla LP Nothing to disclose HHN has received research support, travel grants, and/or teaching honoraria from Biogen, Merck, Novartis, Sanofi Genzyme, Teva, Roche PVR has served on scientific advisory boards for, served as consultant for, received support for congress participation or received speaker honoraria from Alexion, Biogen, Bristol Myers Squibb, Merck, Novartis, Roche, Sanofi RMJ has served on scientific advisory boards for Novartis, Merck, Sanofi and conference travel support from Biogen MB has served on scientific advisory boards, served as a consultant, received support for congress participation, or received speaker honoraria from the Danish MS Society, Biogen, Sanofi, Merck, Novartis, Roche. AT has received support for congress participation from Novartis, Merck KBS has served as consultant for Takeda Pharma A/S, and received travel grants from TEVA, Novartis, Biogen, Merck EC has received compensation for consulting and speaker fees from Alexion, Biogen, BMS, Janssen, Merck, Novartis, Roche, Sanofi MP has received compensation for consulting and speaker fees from Alexion, Biogen, BMS, Janssen, Merck, Novartis, Roche, Sanofi, Horizon MM has served on scientific advisory boards, served as a consultant, received support for congress participation, received speaker honoraria from Roche,Sanofi,Biogen,Merck,Novartis,BristolMyersSquibb,Medscape,Alexion,Moderna

## Muscle and neuromuscular junction disorder 1

## EPR‐063

### Novel RRM2B gene variants associated with mitochondrial DNA deletions syndrome

#### A. Alungulese ^1^; I. Catalina Alvarez^1^; A. Blazquez^2^; P. Serrano‐Lorenzo^2^; J. Suarez Gonzalez^3^; E. Trasobares Iglesias^4^; M. Esteban Rodríguez^5^; F. Arias^6^

##### ^1^Department of Neurology, Gregorio Marañón University Hospital, Madrid, Spain; ^2^Mitochondrial and Neuromuscular Research Group ‘12 de Octubre’, Hospital Research Institute, Madrid, Spain; ^3^Genomics Unit, Gregorio Marañón University Hospital, Madrid, Spain; ^4^Gregorio Marañón Health Research Institute, Madrid, Spain; ^5^Department of Neuropathology, La Paz University Hospital, Madrid, Spain; ^6^Department of Neuropathology, Gregorio Marañón University Hospital, Madrid, Spain

**Background and aims:** Mutations in the nuclear‐encoded mitochondrial maintenance gene RRM2B are an important cause of familial mitochondrial disease in both adults and children and represent the third most common cause of multiple mitochondrial DNA deletions in adults. We report a series of six patients (four symptomatic cases) with novel RRM2B gene variants associated with mitochondrial DNA deletions syndrome and stress the relevance of muscle biopsy to sustain the diagnosis of primary mitochondrial dysfunction and, in cases like ours, to provide material for biochemical and molecular studies required to support the pathogenicity of novel variants.

**Methods:** Investigations included clinical and routine laboratory analyses, electrodiagnostic studies, muscle biopsy and molecular genetics.

**Results:** Evaluation of the clinical features of the symptomatic patients harboring pathogenic RRM2B variants showed that PEO associated with ptosis were universal. Neuromuscular features included proximal muscle weakness and fatigue (3 patients). Needle electromyography revealed a myopathic pattern. CK was occasionally elevated up to 380U/L. Muscle biopsy finding showed subsarcolemmal mitochondrial accumulation (ragged‐red or ragged‐blue fibers) after Gomori trichrome staining or SDH enzyme histochemistry (Figure 1.A&B). A multigene panel detected previously unreported heterozygous change in RRM2B gene, c.941del, p.(Asn314Thrfs*4) in one case and c.1045G > A, p.(Ala349Thr) in 5 patients. All patients who underwent muscle biopsy had multiple mitochondrial DNA deletions detectable either by long‐range PCR assays or by Southern blot analysis (Figure 2).

**Figure:**
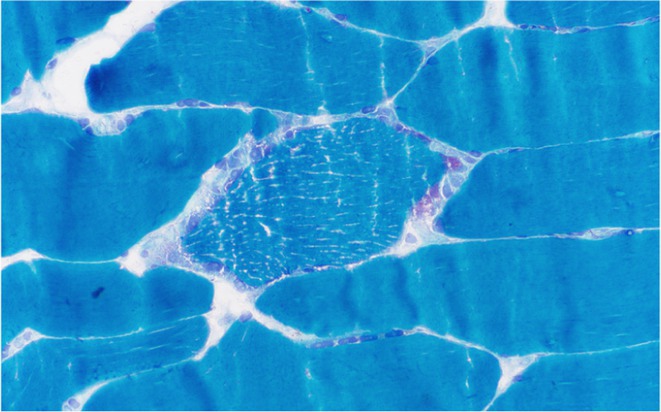
FIGURE 1A

**Figure:**
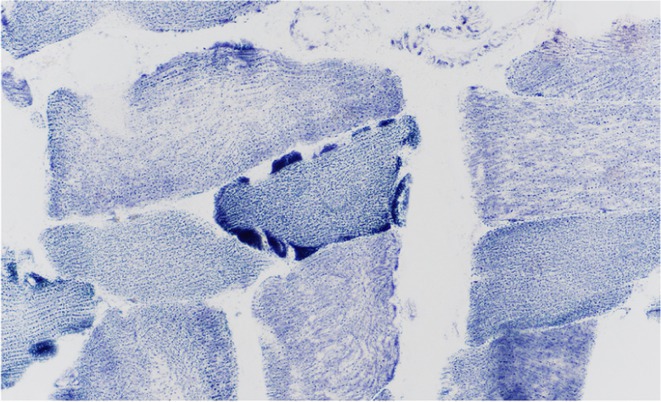
FIGURE 1B

**Figure:**
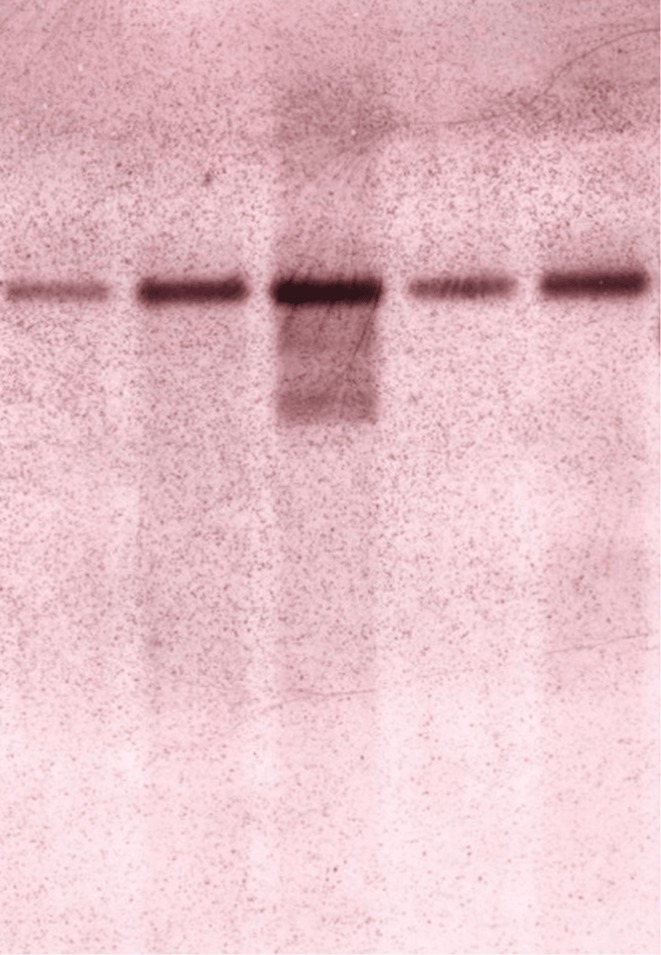
FIGURE 2

**Conclusion:** Our results support the pathogenicity of two novel RRM2B variants found in patients with multiple mtDNA deletions and highlights the utility of muscle biopsy in clarifying variants of unknown significance.

**Disclosure:** Nothing to disclose.

## EPR‐064

### Efgartigimod in pretreated patients with CIDP and progressive/relapsing or persistent disease activity

#### T. Skripuletz^1^; J. Allen^2^; S. Rinaldi^3^; G. Lauria^4^; L. Querol^5^; L. Magy^6^; A. De Roeck^7^; B. Van Hoorick ^7^; E. Hofman^7^; G. istas^7^; M. Stettner^8^; R. Lewis^9^; P. van Doorn^10^

##### ^1^Department of Neurology, Hannover Medical School, Hanover, Germany; ^2^Department of Neurology, University of Minnesota, Minneapolis, USA; ^3^Nuffield Department of Clinical Neurosciences, University of Oxford, Oxford, UK; ^4^IRCCS Foundation “Carlo Besta” Neurological Institute, Milan, Italy; Department of Medical Biotechnology and Translational Medicine, University of Milan, Milan, Italy; ^5^Department of Neurology, Neuromuscular Diseases Unit, Hospital de la Santa Creu i Sant Pau, Universitat Autònoma de Barcelona, Barcelona, Spain; Centro de Investigación Biomédica en Red de Enfermedades Raras (CIBERER), Madrid, Spain; ^6^Department of Neurology, CHU de Limoges, Hôpital Dupuytren, Limoges, France, ^7^argenx, Ghent, Belgium; ^8^Department of Neurology and Center for Translational Neuro‐ and Behavioral Sciences (C‐TNBS), University Medicine Essen, Essen, Germany; ^9^Department of Neurology, Cedars‐Sinai Medical Center, Los Angeles, USA, ^10^Department of Neurology, Erasmus University Medical Center, Rotterdam, The Netherlands

**Background and aims:** In the ADHERE trial, subcutaneous (SC) efgartigimod PH20 (co‐formulated with recombinant human hyaluronidase PH20) reduced relapse rate and was well tolerated in chronic inflammatory demyelinating polyneuropathy (CIDP). This post hoc analysis explores a pre‐specified subgroup of participants with prior CIDP treatment and progressive/relapsing or persistent disease activity (CIDP Disease Activity Status ≥4).

**Methods:** Participants withdrew CIDP treatments during a ≤12‐week run‐in. Participants with active disease entered stage A and received weekly efgartigimod PH20 SC 1000 mg. Those with evidence of clinical improvement entered stage B and were randomized (1:1) to weekly efgartigimod PH20 SC 1000 mg or placebo for ≤48 weeks (Figure). Secondary efficacy outcomes and safety are reported.

**Figure:**
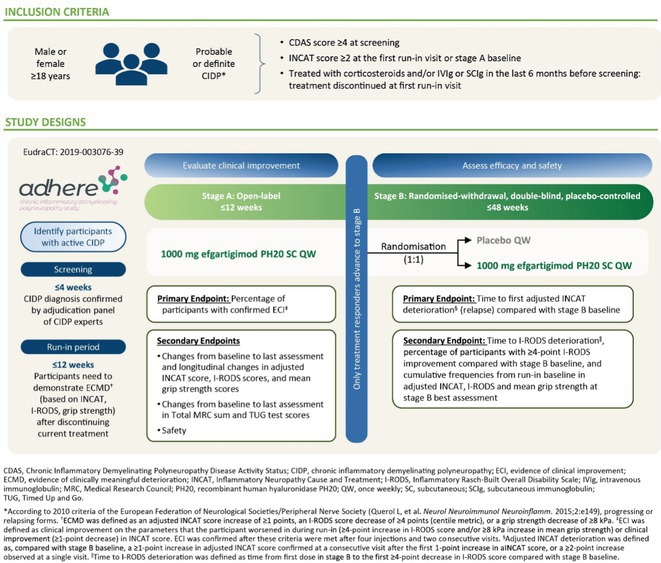
**FIGURE 1** ADHERE study design, with inclusion criteria for the pre‐specified subgroup of pretreated CIDP participants with persistent disease activity or progressive/relapsing active disease

**Results:** 139/322 participants met specified criteria and entered stage A; 95 were randomized and treated in stage B (efgartigimod PH20 SC: 48, placebo: 47). Mean Inflammatory Rasch‐built Overall Disability Scale (I‐RODS) score and mean grip strength (MGS) improved from stage (A and B) baseline to respective last assessment in efgartigimod‐treated participants (Table 1). Mean I‐RODS and MGS scores also improved from stage A‐baseline to stage B‐baseline. In efgartigimod‐treated participants, improvements were maintained up to stage B‐last assessment; scores deteriorated in placebo‐treated participants in stage B. In stage B, efgartigimod PH20 SC reduced the I‐RODS deterioration rate by 60% versus placebo (Table 1). Adverse events were mostly mild or moderate (Table 2). Results by CIDP treatment will be presented at the meeting.

**Figure:**
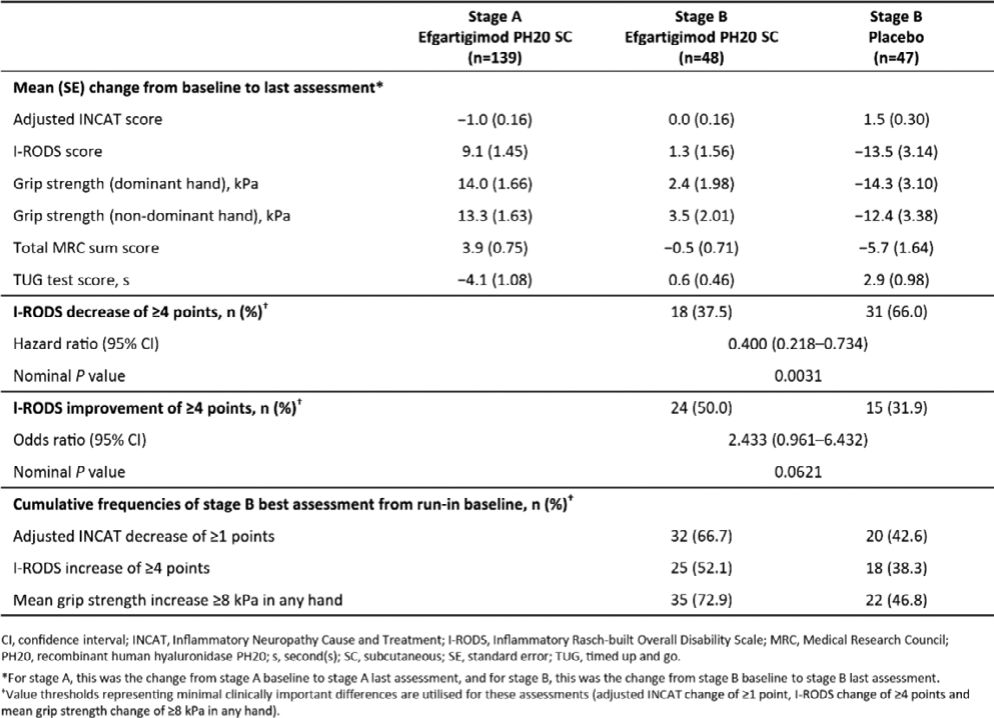
**TABLE 1** Secondary efficacy endpoints in the ADHERE trial in the pre‐specified subgroup of pretreated CIDP participants with persistent disease activity or progressive/relapsing active disease.

**Figure:**
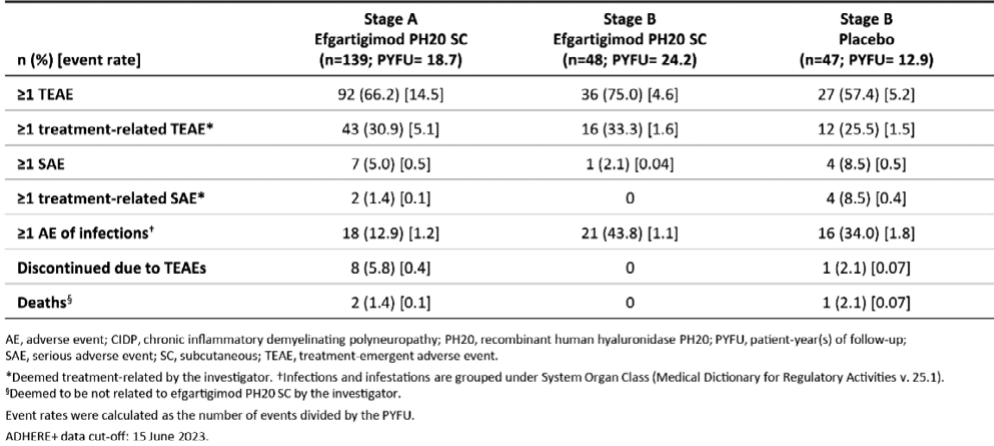
**TABLE 2** Incidence and event rates of adverse events in the pre‐specified subgroup of pretreated CIDP participants with persistent disease activity or progressive/relapsing active disease

**Conclusion:** Efgartigimod PH20 SC provided clinical improvement and was well tolerated in pretreated patients with CIDP and progressive/relapsing or persistent disease activity.

**Disclosure:** TS: Alexion, Alnylam, argenx, Bayer Vital, Biogen, Bristol Myers Squibb, Celgene, Centogene, CSL Behring, EUROIMMUN, Grifols, Hexal AG, Horizon, Janssen‐Cilag, Merck Serono, Novartis, Pfizer, Roche, Sanofi, Siemens, Swedish Orphan Biovitrum, Teva Pharmaceuticals, Viatris; JAA: Akcea Therapeutics, Alexion, Alnylam, Annexon Biosciences, argenx, CSL Behring, Grifols, Immunovant, ImmuPharma, J&J, Pfizer, Takeda; SR: Annexon Biosciences, argenx, CSL Behring, Dianthus Therapeutics, EXCEMED, Fresenius, Hansa Biopharma, Takeda, UCB; GL: Biogen, Chromocell, CSL Behring, Home Biosciences, Janssen, Lilly, Sangamo Therapeutics, Vertex Pharmaceuticals, Zambon; LQ: Alnylam, Annexon Biosciences, argenx, Avilar Therapeutics, Biogen, CSL Behring, Dianthus, Grifols, Janssen, LFB, Lundbeck, Merck, Novartis, Octapharma, Roche, Sanofi, UCB; LM: Alexion, Alnylam, argenx, Biogen, CSL Behring, LFB, Novartis, Pfizer, Roche, Sanofi; ADR, BVH, EH, GI: argenx; MS: argenx, Bayer, Biogen Idec, Biotest, CSL Behring, Genzyme, Grifols, Immunovant, Kedrion, Merck, Novartis, Octapharma, Roche, Sanofi‐Aventis, Teva Pharmaceuticals, UCB; RAL: Alexion, Annexon Biosciences, argenx, Avilar Therapeutics, BioCryst, Boehringer Ingelheim, CSL Behring, Dianthus Therapeutics, Grifols, Immunovant, Intellia Therapeutics, J&J, Nervosave Therapeutics, Novartis, Nuvig Therapeutics, Sanofi, Seismic Therapeutic, Takeda, TGTX; PAvD: Annexon Biosciences, argenx, Grifols, Hansa Biopharma, Octapharma, Roche, Sanofi, Sanquin, Takeda

## EPR‐065

### Myasthenic crises, exacerbations, and corticosteroid use in US patients receiving Ravulizumab or Efgartigimod

#### C. Scheiner ^1^; S. Macwan^2^; N. Streicher^3^; K. Yee^4^; J. Lee^4^; J. Yu^4^; M. Blackowicz^4^; E. Weiskopf^4^; N. Numapau^4^; D. Gentile^5^; J. Sharpe^5^; P. Pathak^5^; M. Pulley^6^

##### ^1^University of Tennessee Medical Center, Knoxville, USA; ^2^Eisenhower Health Center, Rancho Mirage, USA; ^3^MedStar Georgetown University Hospital, UT Neurology, Washington, USA; ^4^Alexion, AstraZeneca Rare Disease, Boston, USA; ^5^Cardinal Health, Dublin, OH, USA; ^6^University of Florida College of Medicine, Jacksonville, USA

**Background and aims:** Ravulizumab (terminal complement inhibitor) and efgartigimod (neonatal Fc receptor blocker) are approved to treat anti‐acetylcholine receptor antibody‐positive generalized myasthenia gravis (gMG). However, real‐world data comparing outcomes with these treatments remains limited. This study evaluated pre‐ and post‐treatment initiation hospitalizations due to crises or exacerbations and corticosteroid use among patients with gMG receiving ravulizumab or efgartigimod.

**Methods:** Physician‐abstracted medical records were included for adults with gMG in Cardinal Health's Neurology Provider Extended Network initiating first targeted immunotherapy on/after 01Dec2021. Hospitalizations due to crises or exacerbations were evaluated in the 6 months pre‐ and post‐treatment initiation; corticosteroid use was evaluated throughout treatment.

**Results:** In total, 45 patients received ravulizumab (female, 35.6%; mean ± SD age at initiation: 61.5 ± 13.6), and 107 received efgartigimod (54.2%; 57.0 ± 16.6). Pre‐initiation, 5 ravulizumab and 3 efgartigimod patients had crises‐related hospitaliszations; post‐initiation, zero hospitalizations for crises occurred in either group. Pre‐initiation, 9 patients in both groups experienced exacerbation‐related hospitalization; post‐initiation, zero ravulizumab patients and 2 efgartigimod patients experienced exacerbation‐related hospitalization. Mean exacerbation‐related hospitalizations per patient per month decreased from 0.17 to 0 among ravulizumab patients and increased from 0.19 to 0.25 among efgartigimod patients. Among patients taking oral corticosteroids (OCS) at initiation, 17/19 (89.5%) ravulizumab patients and 33/46 (71.7%) efgartigimod patients reduced/discontinued their OCS dose post‐initiation.

**Conclusion:** After initiation of either ravulizumab or efgartigimod, fewer patients were hospitalized due to crises or exacerbation, and both treatments were associated with OCS sparing. However, patients receiving ravulizumab trended toward fewer event‐related hospitalizations and a greater reduction in steroid use compared with those receiving efgartigimod.

**Disclosure:** CAS has received compensation for medical advisory board membership and/or serving as a speaker for Alexion, AstraZeneca Rare Disease, argenx, CSL Behring, and UCB. SPM has served as a consultant for Abbvie, Alexion, argenx, Catalyst, Grifols, KabaFusion, Supernus, and UCB. NS is a paid speaker for Alexion and Catalyst. KSY is an employee of Alexion, AstraZeneca Rare Disease, and holds stock or stock options in AstraZeneca and Takeda. JL, JCY, MB, EW, and NN are employees of Alexion, AstraZeneca Rare Disease and hold stock or stock options in AstraZeneca. DG, JS, and PP are employees of Cardinal Health, which received funding to conduct this research. MTP has received compensation for medical advisory board membership or regional advisory board participation from Alexion, AstraZeneca Rare Disease, Amgen, argenx, Catalyst, CSL Behring, Immunovant, and UCB.

## EPR‐066

### Comprehensive analysis of motor endplate pathology in AChR‐Ab‐positive myasthenia gravis

#### C. Preusse ^1^; A. Meisel^1^; P. Doksani^1^; M. Schuelke^2^; J. Rückert^4^; M. Pumberger^5^; F. Schömig^5^; W. Stenzel^3^; S. Hoffmann^1^

##### ^1^Charité – Universitätsmedizin Berlin, corporate member of Freie Universität Berlin, Humboldt‐Universität zu Berlin, and Berlin Institute of Health (BIH), Department of Neurology and Neuroscience Clinical Research Center, Germany; ^2^Charité ‐ Universitätsmedizin, corporate member of Freie Universität Berlin, Humboldt‐Universität zu Berlin and Berlin Institute of Health (BIH), Department of Neuropediatrics and NeuroCure Cluster of Excellence, Germany; ^3^Charité ‐ Universitätsmedizin, corporate member of Freie Universität Berlin, Humboldt‐Universität zu Berlin, and Berlin Institute of Health (BIH), Department of Neuropathology, Germany; ^4^Charité ‐ Universitätsmedizin, corporate member of Freie Universität Berlin, Humboldt‐Universität zu Berlin, and Berlin Institute of Health (BIH), Department of Thoracic Surgery, Germany; ^5^Charité ‐ Universitätsmedizin, corporate member of Freie Universität Berlin, Humboldt‐Universität zu Berlin, and Berlin Institute of Health (BIH), Center for Musculoskeletal Surgery, Germany

**Background and aims:** Since the 1970s, it has been established that complement deposition at the neuromuscular junction (NMJ) in AChR‐ab+ myasthenia gravis (MG), leads to motor endplate destruction. However, no in‐depth studies have been conducted since these early observations. We aimed to re‐examine the NMJ in AChR‐ab+ MG, motivated by the rapid effects of novel therapies which suggest that complete NMJ destruction is unlikely.

**Methods:** Intercostal muscle (ICM) biopsies from 30 AChR‐ab+ MG patients are analyzed by histology, electron microscopy (EM), transcriptomic and proteomic analysis, and compared to non‐myasthenic controls.

**Results:** Histological analysis revealed C5b‐9‐deposition at ~75% (interindividual range of 33‐100%) of all NMJs investigated. EM studies showed postsynaptic simplification and shortened clefts, though not all NMJs showed endplate destruction. There was notable inter‐ and intraindividual variability in endplate destruction despite overall high rates of complement deposition. Transcriptomic analysis revealed upregulation of immune cell markers with TNFA, STAT3 and IL6 showing the strongest expression. Proteomic analysis are pending. Additionally, we will investigate a potential correlation between NMJ destruction and disease severity.

**Conclusion:** Our findings demonstrate variability in complement deposition and NMJ destruction in AChR+ MG, indicating that not all NMJs are equally affected. Histologic and transcriptomic analyses suggest a critical role of macrophages, which could promote future therapeutic strategies.

**Disclosure:** Project supported by Janssen Pharmaceutica NV, a Johnson and Johnson company

## EPR‐067

### Real world study in Italian public hospital with Efgartigimod in patients affected by generalized myasthenia gravis

#### M. Sgarzi; P. Paone; G. Camera; E. Agazzi; D. Alimonti

##### ASST Papa Giovanni XXIII Hospital

**Background and aims:** Myasthenia gravis (MG) is an autoimmune neuromuscular disorder caused by IgG autoantibodies targeting the neuromuscular junction. Recycling of IgG is mediated by the neonatal Fc receptor (FcRn). Efgartigimod, an Fc fragment of human IgG1, has demonstrated efficacy in MG; however, the clinical characteristics of patients with the highest response remain unclear.

**Methods:** Twelve patients with AChR‐positive generalized MG were treated with two cycles of Efgartigimod over one year, and nine patients completed a third cycle. Clinical evaluation was conducted using MG‐ADL at four time points and QMG at the beginning and end of each cycle. MG‐ADL and QMG scores were further subdivided into ocular (O), bulbar (B), and generalized (G) symptom subdomains, and patients were classified as predominantly ocular (pO), bulbar (pB), or generalized (pG) based on symptom prevalence.

**Results:** Significant improvements were observed in MG‐ADL and QMG from baseline across all symptom subdomains. Baseline AChR antibody levels correlated with MG‐ADL improvement (*p* < .04). Thymectomized patients demonstrated superior outcomes, with MG‐ADL improving by 62% versus 22% (*p* < .01) and QMG by 45% versus 3.5% (*p* < .01) during the first two cycles. Patients with pO symptoms responded less to therapy, with generalized symptoms contributing most to the minor response.

**Figure:**
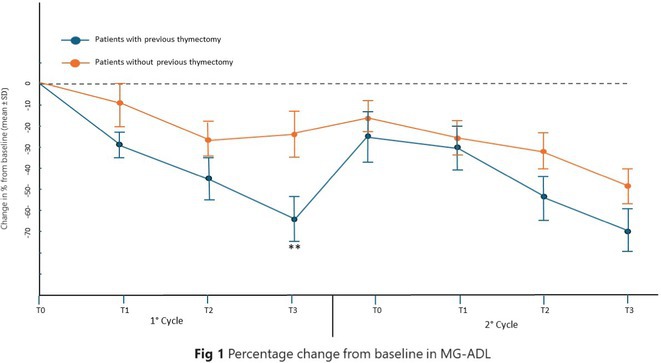
**FIGURE 1** Change of mean MG‐ADL (SD) from baseline (T0 of 1°cycle) of patients with previous thymectomy or not. ** *p* < .01 with *U*‐Mann–Whitney test

**Figure:**
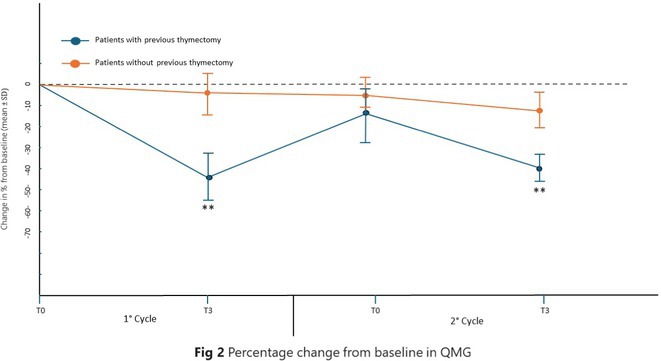
**FIGURE 2** Change of mean QMG (SD) from baseline (T0 of 1°cycle) of patients with previous thymectomy or not. ** *p* < .01 with *U*‐Mann–Whitney test.

**Conclusion:** Our findings suggest that patients with high baseline AChR antibody titers, previous thymectomy, and non‐ocular symptom predominance respond better to Efgartigimod. These results underscore the need for larger studies to validate these observations and optimize patient selection.

**Disclosure:** Nothing to disclose.

## EPR‐068

### Pulmonary manifestations in adult patients with Dermatomyositis (DM): Effect of intravenous immunoglobulins

#### J. Schessl ^1^; R. Aggarwal^2^; E. Clodi^3^; C. Charles‐Schoeman^4^

##### ^1^Friedrich‐Baur‐Institute, Department of Neurology, Ludwig‐Maximilians University of Munich, Munich, Germany; ^2^Division of Rheumatology and Clinical Immunology, University of Pittsburgh School of Medicine, Pittsburgh, USA; ^3^Octapharma PPG, Vienna, Austria; ^4^University of California, Los Angeles, USA

**Background and aims:** Pulmonary manifestations (e.g., interstitial lung disease) are a major cause of morbidity and mortality in patients with dermatomyositis (DM). The ProDERM trial was designed to evaluate the efficacy and safety of intravenous immunoglobulin (IVIg) in adult DM patients.

**Methods:** The ProDERM trial was a placebo‐controlled, double‐blind, Phase 3 study. We present a post‐hoc analysis of the ProDERM data on the effect of IVIg on pulmonary symptoms. Investigators assessed pulmonary symptoms at baseline and all subsequent visits by using the Myositis Disease Activity Assessment Tool (MDAAT). Visual analog scales (VAS) were scored over 10 cm to quantify overall pulmonary disease activity (higher score indicating worse disease). Physicians also reported presence/absence of dysphonia, dyspnea or cough from active reversible ILD, and dyspnea from respiratory muscle weakness on MDAAT.

**Results:** At baseline 59/95 patients (62.1%) had a pulmonary VAS ≤0.5 cm (Figure 1), indicating no myositis lung involvement. By Week 40, the number of patients with a VAS ≤0.5 cm increased to 68 of the 88 patients with available data (77.3%; *p* = 0.007). At week 16 (end of placebo‐controlled phase) data of patients with VAS > 0.5 cm showed a mean decrease in the IVIg group of 1.15 cm (*n* = 12; 37.7%; *p* = 0.001) versus 0.17 cm (6.5%) in patients on placebo (*n* = 21; *p* = 0.50). The percentage of patients with dysphonia decreased from 20% at baseline to 8% at Week 40 (*p* = 0.04).

**Figure:**
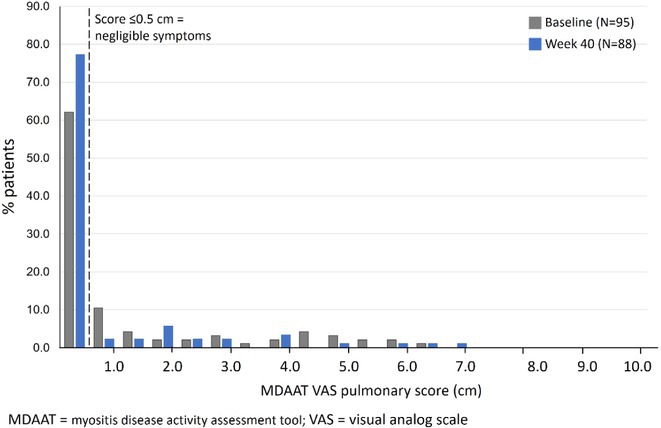
**FIGURE 1** MDAAT pulmonary VAS score at baseline and Week 40.

**Conclusion:** IVIg may have favorable effects on active pulmonary manifestations of DM. Future studies should include pulmonary outcomes.

**Disclosure:** J. Schessl: Pfizer, Octapharma C. Charles‐Schoeman: Pfizer, Octapharma, AbbVie, Bristol Myers Squibb, Boehringer Ingelheim, Recludix, Galapagos, Sana Biotechnology, Immunovant, Pfizer, Alexion, Priovant, CSL Behring, E. Clodi: Employee of Octapharma R. Aggarwal: Boehringer Ingelheim, Bristol Myers Squibb, EMD Serono, Janssen, Priovant, Pfizer, Alexion, ANI Pharmaceutical, Argenx, Artasome, AstraZeneca, CabalettaBio, Capella, Capstanx, Corbus, CSL BEhring, Glaapagos, Horizontal Therapeutics, I‐Cell, Immunovant, Kezar, Kyverna, Lilly, Manta Medicines, Novartis, Nuvig Therapeutic, Nakarta, Octapharma, Teva, Tourmaline Bio & Verismo Therapeutics

## EPR‐069

### Association of recent infection and subsequent myasthenia gravis: A nationwide case‐control study from 1985 to 2020

#### J. Jul Jarbæk Nielsen; L. Levison; H. Andersen

##### Neurology, Aarhus University Hospital, Aarhus, Denmark

**Background and aims:** Infections have been suggested to trigger myasthenia gravis (MG) onset. We determined the association between recent infections and the subsequent development of MG.

**Methods:** We used nationwide health registers from 1985 to 2020 to identify MG patients by linking individual‐level data across registers. Each patient was matched to 10 controls from the general population based on age, sex, and diagnostic index date. Using conditional logistic regression, we computed matched odds ratios (ORs) with 95% confidence intervals to assess the relative risk of developing MG following recent infection. Analyses were adjusted for baseline comorbidities.

**Results:** We identified 2,110 MG patients and 21,100 matched individuals from the general population, with 27.4% aged 50 years or younger. In the year preceding MG diagnosis (excluding the month before diagnosis), 4.9% of MG patients experienced a hospital‐diagnosed infection, compared to 2.8% in the general population, with an OR of 1.8 (95% CI: 1.5–2.2). The strongest associations were observed for lower respiratory tract and ear, nose, and throat infections. Comorbidity‐adjusted analyses did not alter the results (OR 1.7, 95% CI 1.4‐2.2).

**Conclusion:** MG patients have a 1.8‐fold higher infection risk in the year prior to diagnosis compared to the general population, likely due to unrecognized respiratory and bulbar dysfunction or infections triggering MG development. Clinical awareness of this association could enable early prevention and intervention strategies.

**Disclosure:** Nothing to disclose.

## EPR‐070

### Ulviprubart pharmacokinetics, pharmacodynamics (PK/PD), and safety: Inclusion body myositis (IBM) phase 1 study results

#### M. Needham ^1^; R. Henderson^2^; C. Liang^3^; D. Soler‐Ferran^4^; H. Wilkins^4^; S. Greenberg^5^

##### ^1^Perron Institute, QEII Medical Centre, Nedlands, Western Australia, Australia; University of Notre Dame, Fiona Stanley Hospital, and Murdoch University, Perth, Western Australia, Australia; ^2^Department of Neurology, Royal Brisbane & Women's Hospital and University of Queensland Centre for Clinical Research, University of Queensland, Brisbane, Queensland, Australia; ^3^Royal North Shore Hospital, St Leonards and Northern Clinical School, University of Sydney, New South Wales, Australia; ^4^Abcuro, Inc., Newton, MA, USA; ^5^Abcuro, Inc., Newton, MA, USA and Brigham and Women's Hospital, Harvard Medical School, Boston, MA, USA

**Background and aims:** IBM is a rare, progressive disease characterized by invasion of muscle by highly differentiated cytotoxic CD8+ T cells. Ulviprubart, a monoclonal antibody, selectively depletes cytotoxic CD8+ KLRG1+ T cells by targeting KLRG1 expressed on most IBM‐muscle–infiltrating T cells. Here, we describe PK/PD and safety of ulviprubart in patients with IBM.

**Methods:** In this phase 1, open‐label study (NCT04659031), initial patients received subcutaneous ulviprubart (0.1, 0.5, or 2.0 mg/kg) as a single dose prior to dosing every 8 weeks (Q8W) ~6−12 months later, while later patients received 2.0 mg/kg Q8W; patients received ulviprubart for up to 18 months. PK/PD and safety with ulviprubart were assessed.

**Results:** Nineteen patients (mean age, 66 years; 79% male) were enrolled (0.1 mg/kg: *n* = 3; 0.5 mg/kg: *n* = 3; 2.0 mg/kg: *n* = 13). Ulviprubart displayed a long absorption phase, slow clearance, and 21‐day half‐life. Peripheral CD8+ KLRG1+ and CD4+ KLRG1+ T‐cell depletion was achieved, with mean CD8+ KLRG1+ T‐cell maximum depletions of 69%, 97%, and 98% after single doses of 0.1, 0.5, and 2.0 mg/kg, respectively. Effector CD8+ T‐cell populations (T‐cell effector memory [TEM] and TEMs expressing CD45RA [TEMRA]) were depleted to the extent of their KLRG1 expression. Regulatory T cells and B cells were preserved. No serious adverse events (AEs) or discontinuations due to AEs were reported.

**Conclusion:** In patients with IBM, ulviprubart led to sustained selective depletion of peripheral blood CD8+ KLRG1+ T cells and had a favorable safety profile.

**Disclosure:** M Needham, RD Henderson, and C Liang: Abcuro, Inc. − Consultant D Soler‐Ferran and HJ Wilkins: Abcuro, Inc. – Employment S Greenberg: Abcuro, Inc. – Founder; Consultant

## EPR‐071

### Clinical outcome of myasthenic crisis: A multicenter prospective study

#### X. HUAN ^1^; R. Chen^1^; L. Jin^1^; J. Song^1^; H. Zhong^1^; Y. Wang^2^; Q. Jiang^3^; Z. Zou^4^; Y. Liu^5^; S. Tan^6^; S. Luo^1^; C. Zhao^1^

##### ^1^Huashan Rare Disease Center and Department of Neurology, Huashan Hospital, National Center for Neurological Disorders, Fudan University, Shangha, China.; ^2^Department of Blood Transfusion, Huashan Hospital Fudan University, Shanghai, China.; ^3^Department of Myopathy, The First Affiliated Hospital of Guangzhou University of Chinese Medicine, Guangzhou, China; ^4^Department of Neurology, Fujian Medical University Union Hospital, Fuzhou, China; ^5^Department of Neurology, Zhongnan Hospital of Wuhan University, Wuhan, China; ^6^Department of Neurology, Sichuan Provincial People's Hospital, University of Electronic Science and Technology of China, Chengdu, China

**Background and aims:** Myasthenic crisis (MC) affects 15‐20% of patients with MG (Myasthenia gravis) and remains a major clinical challenge, with high mortality and significant economic burden. With advances in disease monitoring and rescue therapies, the in‐hospital mortality for MC reduced significantly. However, studies regarding the long‐term outcomes post‐MC are limited.

**Methods:** This observational multicenter cohort study with prospective collected patients diagnosed with MC or impending MC between December 2018 and October 2024 (NCT04837625). Outcome measures included all‐cause mortality,1‐year MGFA postintervention status (PIS), and long‐term complications associated with MG.

**Figure:**
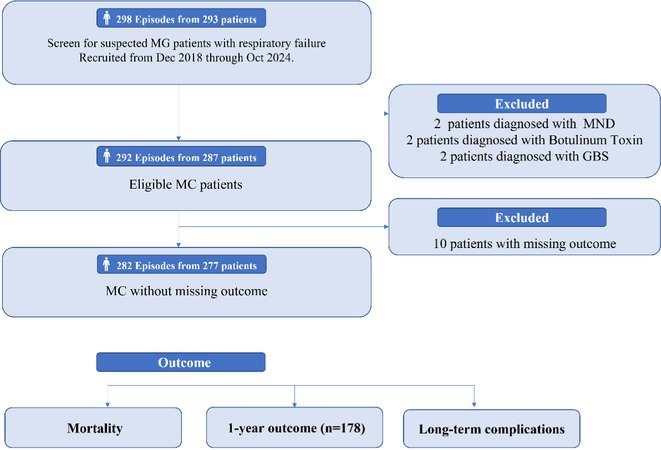
**FIGURE 1** Flowchart of the study cohort.

**Results:** In this cohort, we included 282 episodes of myasthenia gravis (MG) with an average follow‐up time of 26.13  ± 23.5 months and 923 clinical assessments. The all‐cause mortality rate was 14.9% (42/282), with an in‐hospital mortality rate of 4.96% (14/282). The primary causes of death were infectious shock (42.9%), multiple organ failure (16.2%), myocarditis (16.2%), and thymoma recurrence (8.1%). Kaplan–Meier survival curves indicated that patients with thymoma‐associated myasthenia gravis (TAMG) had a significantly lower survival rate compared to those with late‐onset (LOMG) and early‐onset myasthenia gravis (EOMG) throughout the observation period (*p* < 0.001). Among 178 patients with consecutive 1‐year follow‐up, 44.9% (80/178) achieved minimal manifestation (MM) status or better. Long‐term complications included recurrent infections (11.0%), gastrointestinal ulcers (8.2%), metabolic abnormalities (8.2%), osteoporosis (4.9%), and chronic pain (4.4%).

**Figure:**
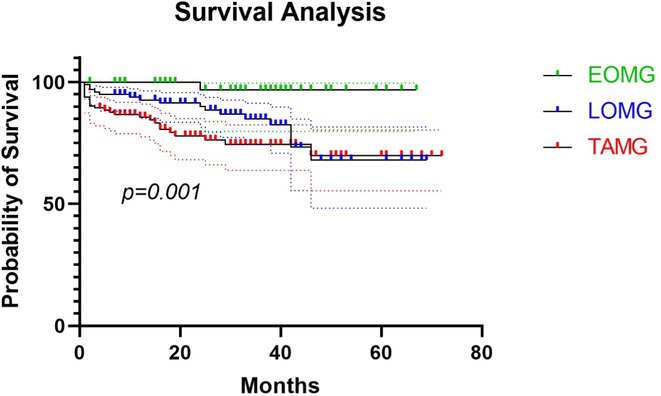
**FIGURE 2** Kaplan–Meier survival analysis of thymoma‐associated myasthenia gravis, late‐onset and early‐onset myasthenia Gravis

**Conclusion:** This is the first prospective cohort study to report the long‐term outcomes of MG patients post‐MC, highlighting the importance of disease monitoring and timely intervention in improving the life quality of MG.

**Disclosure:** Nothing to disclose.

## Neurogenetics

## EPR‐072

### Expanding the genotype‐phenotype spectrum of KCNA2‐related disorders

#### C. Ferrazzoli ^1^; L. Affronte^3^; M. De Wachter^4^; S. Gverdtsiteli^2^; R. Dahl^2^; U. Hedrich^5^; S. Syrbe^6^; S. Lauxmann^7^; I. Scheffer^8^; K. Study group^2^; H. Lerche^5^; R. S Møller^9^; G. Rubboli^10^

##### ^1^University of Rome “Tor Vergata”, Roma, Italy; ^2^Department of epilepsy genetics and personalized medicine, Danish Epilepsy Centre, Dianalund, Denmark; ^3^IRCCS Azienda Ospedaliero‐Universitaria di Bologna, Bologna, Italy; ^4^Antwerp University Hospital, University of Antwerp, Edegem, Belgium; ^5^Department of Neurology and Epileptology, Hertie Institute for Clinical Brain Research, University of Tübingen, Tübingen, Germany; ^6^Pediatric Epileptology, Center for Child and Adolescent Medicine, Medical Faculty, Heidelberg University, Heidelberg, Germany; ^7^Institute of Neurobiology, Faculty of Mathematics and Natural Sciences, University of Tübingen, Tübingen, Germany; ^8^Austin Health and Royal Children's Hospital, Florey and Murdoch Children's Research Institutes, University of Melbourne, Melbourne, VIC, Australia; ^9^University of Southern Denmark, Odense, Denmark, ^10^Institute of Clinical Medicine, University of Copenhagen, Copenhagen, Denmark

**Background and aims:** The aim of this study was to refine the genotype and phenotypic spectrum of KCNA2‐related disorders.

**Methods:** We collected phenotypic data of patients with electrophysiologically characterized KCNA2 variants through literature search, an international network of epilepsy and genetic centers.

**Results:** 122 patients (52 novel, 70 published) harboring KCNA2 pathogenic variants were collected. Inheritance was: de novo = 65, familial = 20, unknown = 37. Functional studies revealed: Loss‐Of‐Function (LOF) in 71/122, Gain‐Of‐Function (GOF) 38/122, GOF‐LOF 13/122. Mean age at epilepsy onset was 3.5 months in GOF‐LOF patients, around 10 months in both the LOF and GOF groups. Seizure types in the three functional groups are described in the table below. Febrile seizures occurred more frequently in LOF patients. Intellectual disability was mild in 23/54 LOF, 6/38 GOF, 1/13 GOF‐LOF, moderate in 10/54 LOF, 8/38 GOF, 2/13 GOF‐LOF, severe/profound in 8/54 LOF, 3/38 GOF, 6/13 GOF‐LOF. Most common neurological features were ataxia and tremor. 1/3rd of GOF‐LOF subjects had spasticity. MRI showed as the most common feature cerebellar atrophy, with predominance in GOF‐LOF.

**Figure:**
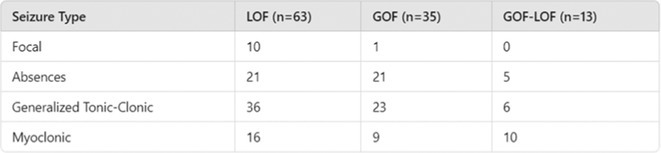
**TABLE 1** Seizure types in the three functional groups.

**Conclusion:** The largest cohort of patients with KCNA2‐related‐disorders to date is described in this study. Our data confirm genotype‐phenotype correlations with LOF variants featuring a higher proportion of milder phenotypes, whereas the most severe clinical presentations were observed in the GOF‐LOF group.

**Disclosure:** Nothing to disclose.

## EPR‐073

### Restoration of neurodegeneration in SCA3 via modulation of the insulin/IGF‐1 signaling pathway using astragaloside IV

#### C. Liu ^1^; Y. Lin^2^; W. Cheng^2^; T. Lin^2^

##### ^1^Department of Neurology, Changhua Christian Hospital, Changhua, Taiwan; ^2^Vascular and Genomic Research Center, iATP, Changhua Christian Hospital, Changhua, Taiwan

**Background and aims:** The pathogenesis of Spinocerebellar ataxia type 3 (SCA3) is associated with dysregulation of the Insulin/IGF‐1 signaling (IIS) pathway. Astragaloside IV (AST), a potent antioxidant, has demonstrated properties that enhance IIS activity.

**Methods:** This study investigated the therapeutic effects of AST (25 mg/kg and 50 mg/kg) on motor deficits, degradation of the SCA‐84Q mutant protein, IIS pathway modulation, mitochondrial function, and expression levels of phosphorylated IGF1R (p‐IGF1RTyr1135/1136) and IRS1 (p‐IRS1Ser307) in transgenic SCA‐15Q/84Q mouse models.

**Figure:**
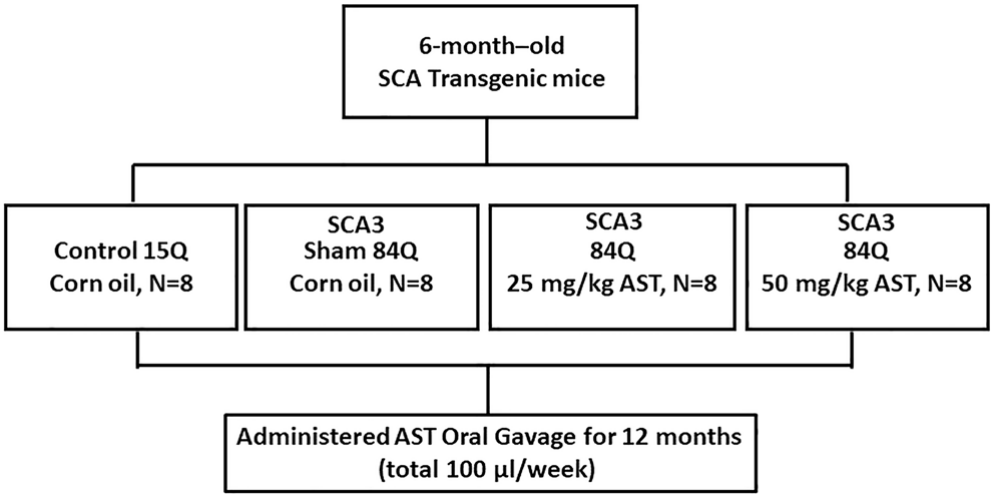
**FIGURE 1** Flow chart of Astragaloside IV(AST) therapy in SCA3 transgenic mice

**Results:** AST treatment improved motor performance, enhanced mitochondrial function in the cerebellum, and promoted Purkinje neuron survival. It also modulated the IIS pathway by upregulating Ins2 gene expression, increasing p‐IGF1RTyr1135/1136, and suppressing p‐IRS1Ser307 in the cerebellum of SCA‐84Q mice.

**Figure:**
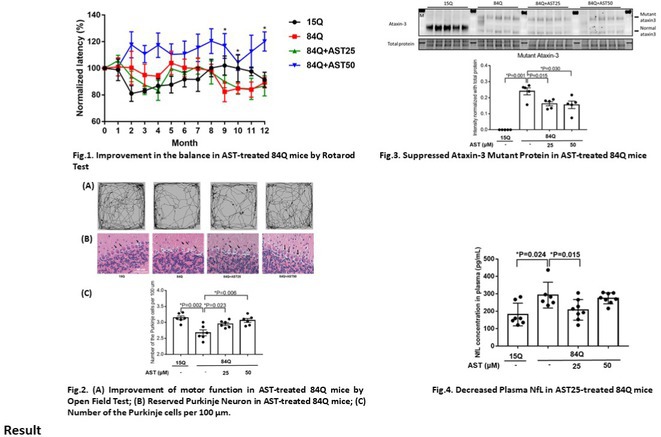
FIGURE 2

**Figure:**
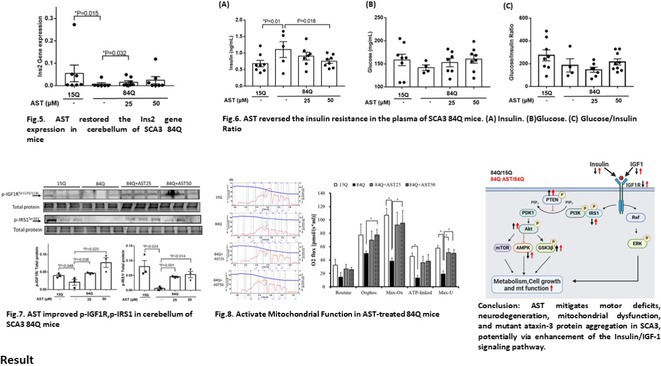
FIGURE 3

**Conclusion:** AST mitigates motor deficits, neurodegeneration, mitochondrial dysfunction, and mutant ataxin‐3 protein aggregation in SCA3, potentially via enhancement of the IIS pathway.

**Disclosure:** Nothing to disclose.

## EPR‐074

### Genetic findings through whole exome sequencing (WES) in dystonia patients

#### G. Bonato ^1^; M. Carecchio^1^; A. Antonini^1^; R. Erro^3^; F. Morgante^4^; F. Cavallieri^5^; V. Fioravanti^5^; G. Di Rauso^5^; F. Valzania^5^; G. Mostile^6^; M. Zappia^7^; F. Spagnolo^8^; P. Mandich^9^; E. Moro^10^; V. Rispoli^11^; P. Tocco^12^; D. Ottaviani^13^; M. Pellegrini^14^; M. Malaguti^14^; A. Di Fonzo^15^; E. Monfrini^15^

##### ^1^Parkinson and Movement Disorders Unit, Center for Rare Neurological Diseases (ERN‐RND), Department of Neuroscience, University of Padova, Padova, Italy; ^3^Neuroscience Section, Department of Medicine, Surgery and Dentistry “Scuola Medica Salernitana”, University of Salerno, Baronissi (Province of Salerno), Italy.; ^4^City St George's University of London, Neuroscience and Cell Biology Institute, London, UK; ^5^Neurology Unit, Neuromotor & Rehabilitation Department, Azienda USL‐IRCCS di Reggio Emilia, Reggio Emilia, Italy.; ^6^Department of Medical, Surgical Sciences and Advanced Technologies “GF Ingrassia”, University of Catania, Catania, Italy; Oasi Research Institute‐IRCCS, Troina, Italy; ^7^Department of Medical, Surgical Sciences and Advanced Technologies “GF Ingrassia”, University of Catania, Catania, Italy.; ^8^Neurological Department, A. Perrino's Hospital, Brindisi, Italy; ^9^IRCCS Ospedale Policlinico San Martino, UOC Genetica Medica, Genoa, Italy; Department of Neurosciences, Rehabilitation, Ophthalmology, Genetics, Maternal, and Child Health, University of Genoa, Genoa, Italy, ^10^Grenoble Alpes University, CHU of Grenoble, Division of Neurology, Grenoble, France, ^11^Neurology Unit, Department of Neuroscience, S. Agostino Estense Hospital, Azienda Ospedaliero‐Universitaria di Modena, Modena, Italy, ^12^Neurology and Stroke Unit, Pescara Hospital, Pescara, Italy, ^13^Department of Neurology, “Santa Maria del Carmine” Hospital, Azienda Provinciale per i Servizi Sanitari (APSS), Rovereto, Italy, ^14^Neurology Unit, Trento Hospital, Azienda Provinciale per i Servizi Sanitari (APSS) di Trento, Trento, Italy., ^15^Neurology Unit, Foundation IRCCS Ca'Granda Ospedale Maggiore Policlinico, Milan, Italy.

**Background and aims:** Dystonia is a hyperkinetic movement disorder characterized by abnormal repetitive movement and/or postures. Exome sequencing (ES) studies have shown a diagnostic yield of 20%, with higher rates in young onset, generalized and combined/complex dystonia, suggesting genetic testing only in selected patients. We evaluated diagnostic yield of genetic testing in an Italian dystonia cohort.

**Methods:** Cohort of 100 dystonic patients (45% males, mean age at evaluation 46 ± 19 years), ES, neurological examination, disease history collection, brain MRI.

**Results:** Childhood‐onset dystonia was documented in 31% of the cohort, adult‐onset in 51%; mean age at onset was 24.6 ± 19 years. Family history was positive in 53%. Overall, 38% had generalized dystonia, 26% multifocal, 19% focal, 17% segmental. 52% had isolated dystonia, 29% complex (mainly associated with intellectual disability), and 19% combined. Myoclonus was the most frequently associated movement disorder, followed by Parkinsonism. Genetic diagnosis was reached in 26% of cases, the most frequent genes being KMT2B (4 cases), VPS16 (3), GNAL (2) and GCH1 (2). Diagnostic yield was higher in complex phenotypes (41%), generalized dystonia (39%) and childhood onset (35%), although a genetic cause was found also in 15% of isolated dystonia, 21% of focal dystonia and 20% of adult‐onset dystonia. Unlike family history, brain MRI (iron deposition, basal ganglia alterations, cortical or cerebellar atrophy, leukopathy) provided helpful diagnostic clues in 38% of patients with a positive genetic diagnosis.

**Figure:**
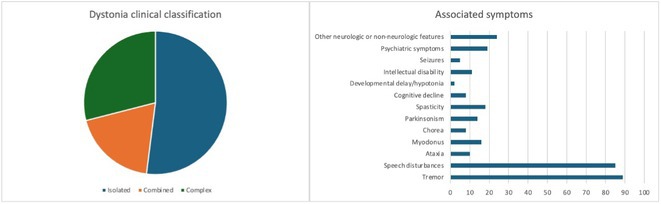
**FIGURE 1** Distonia classification and associated clinical features in the cohort.

**Conclusion:** ES is a powerful tool for genetic diagnosis in dystonia, with better results in complex phenotypes, young patients and suggestive alterations at brain MRI.

**Disclosure:** Nothing to disclose.

## EPR‐075

### Development of a gene panel for Parkinson's disease and repeat expansion disorders using long‐read adaptive sampling

#### J. Prietzsche ^1^; J. Laß^1^; C. Much^1^; S. Schaake^1^; T. Lüth^1^; C. Gabbert^1^; A. Fienemann^1^; A. Zimprich^2^; E. Stögmann^2^; T. König^2^; C. Ganos^3^; T. Gül‐Demirkale^4^; A. Başak^4^; R. Jamora^5^; R. Rosales^6^; G. Saranza^7^; C. Diesta^8^; P. Seibler^1^; M. Borsche^9^; N. Brüggemann^9^; C. Klein^1^; J. Trinh^1^

##### ^1^Institute of Neurogenetics, University of Lübeck, Lübeck, Germany; ^2^Department of Neurology, Medical University of Vienna, Vienna, Austria; ^3^Department of Neurology, Charité University Medicine, Berlin, Germany; ^4^Suna and İnan Kıraç Foundation, Neurodegeneration Research Laboratory (NDAL), KUTTAM, School of Medicine, Koç University, İstanbul, Turkey; ^5^Department of Neurosciences, College of Medicine‐Philippine General Hospital, University of the Philippines Manila, Manila, Philippines; ^6^Department of Neurology and Psychiatry, University of Santo Tomas and the CNS‐Metropolitan Medical Center, Manila, Philippines; ^7^Section of Neurology, Department of Internal Medicine, Chong Hua Hospital, Cebu, Philippines; ^8^Department of Neurosciences, Movement Disorders Clinic, Makati Medical Center, Makati City, Philippines; ^9^Department of Neurology, University of Lübeck and University Hospital Schleswig‐Holstein, Lübeck, Germany

**Background and aims:** Long‐read sequencing has improved the detection of structural variants and repeat expansions. We are now able to delineate complex structural rearrangements, repeat expansions, and low‐complexity regions with long‐reads. However, whole genome sequencing is still costly for genetic diagnostics. Herein, we created a neurodegenerative gene panel on the Oxford Nanopore Technologies (ONT) adaptive sampling platform that targets PD‐related and repeat expansion genes. We investigated the genetic diagnostic utility of this newly designed panel.

**Methods:** The long‐read gene panel consisted of 565 genes selected from genetic diagnostic panels for neurodegeneration (CeGat), MDS TaskForce genes, PD GWAS loci, and genes related to repeat expansion disorders. We tested the panel's diagnostic utility on patients with 1) known pathogenic variants in LRRK2, PRKN, SNCA, and RAB32 (*n* = 6); 2) idiopathic PD negative for known genetic causes (*n* = 3); 3) known repeat expansions in the genes ATXN1, ATXN2, ATXN3, C9orf72, DAB1, FGF14, HTT, RFC1, TAF1, and ZFHX3 (*n* = 12).

**Results:** A mean coverage of 25X and a mean Q‐score of 18.8 was obtained across all genes and individuals with an average read length of 10kb. Thus far, using an EPI2ME workflow and the software NCRF, we accurately detected four PRKN deletions, a missense variant in LRRK2, and repeat expansions in ATXN2, ATXN3, FGF14, and RFC1. However, an SNCA triplication was not detected.

**Conclusion:** This type of panel design has the potential to be a more efficient genetic diagnostic tool for structural variant detection. Further validation of the panel with a larger and more diverse patient cohort would strengthen its potential.

**Disclosure:** Nothing to disclose.

## EPR‐076

### Phenotypic‐genetic spectrum of STUB1‐related disorders: Insights into the severe end with neurodevelopmental disorder

#### K. Gunanayagam ^1^; R. Maroofian^1^; M. Abdel‐Hamid^2^; B. Stringer^3^; J. Alvi^4^; S. Efthymiou^1^; D. Calame^5,6^; A. Nezhad^7,8^; Z. Rezaei^9,10^; L. Habibi^8^; K. Zhelcheska^1^; A. Gezdirici^11^; T. Mitani^12^; D. Pehlivan^5,6,13^; A. Kocaaga^14^; J. Lupski^6,12,15^; M. Suri^16^; C. Alves^17^; J. Gleeson^18,19^; T. Sultan^4^; J. Giacomotto^20^; M. Zaki^21^; H. Houlden^1^

##### ^1^Department of Neuromuscular Diseases, UCL Queen Square Institute of Neurology, London WC1N 3BG, UK; ^2^Medical Molecular Genetics Department, Human Genetics and Genome Research Institute, National Research Centre, Cairo, Egypt; ^3^Griffith Institute for Drug Discovery, Centre for Cellular Phenomics, School of Environment and Science, Griffith University, Brisbane, Queensland, Australia; ^4^Department of Pediatric Neurology, the Children's Hospital and the University of Child Health Sciences, Lahore 54600, Pakistan; ^5^Section of Neurology and Developmental Neuroscience, Department of Pediatrics, Baylor College of Medicine, Houston, TX, 77030, USA; ^6^Texas Children's Hospital, Houston, Texas, 77030, USA; ^7^Maternal, Fetal and Neonatal Research Center, Family Health Research Institute, Tehran University of Medical Sciences, Tehran, Iran; ^8^Ronash Medical Laboratory, Tehran, Iran; ^9^Children's Medical Center, Paediatric Center of Excellence, Tehran university of Medical Sciences, Tehran, Iran, ^10^Paediatric and adolescent Multidisciplinary Epilepsy Research Center (PAMERC), Tehran University of Medical Sciences, Tehran Iran, ^11^Department of Medical Genetics, Basaksehir Cam and Sakura City Hospital, 34480, Istanbul, Turkey, ^12^Department of Molecular and Human Genetics, Baylor College of Medicine, Houston, TX, 77030, USA, ^13^Jan and Dan Duncan Neurological Research Institute at Texas Children's Hospital, Houston, TX, 77030, USA, ^14^Department of Medical Genetics, Eskisehir City Hospital, Eskişehir, Turkey, ^15^The Human Genome Sequencing Center, Baylor College of Medicine, Houston, TX, USA, ^16^Department of Clinical Genetics, Nottingham City Hospital, Nottingham, UK, ^17^Division of Neuroradiology, Department of Radiology, Boston Children's Hospital ‐ BCH Harvard Medical School, 300 Longwood Avenue, Boston, MA 02115, USA, ^18^Department of Neurosciences, University of California, San Diego, La Jolla 92093, USA, ^19^Rady Children's Institute for Genomic Medicine, San Diego 92123, USA, ^20^Queensland Brain Institute, The University of Queensland, Brisbane, Queensland, Australia, ^21^Clinical Genetics Department, Human Genetics and Genome Research Institute, National Research Centre, Cairo, Egypt

**Background and aims:** Pathogenic variants in STUB1 are associated with a broad spectrum of neurodegenerative disorders, including spinocerebellar ataxias‐16 and 48. Additionally, biallelic STUB1 variants have been reported in only four patients with neurodevelopmental disorders characterized by global developmental delay (GDD) and variable intellectual disability (ID). STUB1 encodes an E3 ubiquitin ligase that is crucial for maintaining protein homeostasis, yet the full phenotypic and allelic spectrum remains poorly understood.

**Methods:** Ten probands from eight consanguineous families with STUB1 variants were identified using exome sequencing and international data sharing platforms. Clinical phenotypes and brain magnetic resonance imaging were analyzed. Zebrafish STUB1 knockout embryos by CRISPR/Cas9 were generated and the role of STUB1 on brain morphology and motor function was characterized.

**Results:** We describe homozygous missense and predicted loss‐of‐function STUB1 variants in ten patients presenting with a neurodevelopmental phenotype characterized by GDD/ID, dysmorphic facial features, movement disorders, and ataxia. Brain imaging revealed two distinctive patterns: one of cerebellar atrophy; and the other of enlargement of the frontoparietal extra‐axial subarachnoid spaces, with compensatory lateral ventricle enlargement and thinning of the corpus callosum. Comparative studies in stub1 knockout zebrafish revealed early medulla size reduction, late‐onset cerebellar and dendritic defects, and motor abnormalities, without significant impact on overall brain structure. There was also slow growth.

**Conclusion:** This study not only consolidates the association of STUB1 defects with a neurodevelopmental phenotype but also expands the understanding of STUB1‐related disorders as part of an emerging group of conditions with a broad spectrum ranging from neurodevelopmental disorders to neurodegeneration.

**Disclosure:** Nothing to disclose.

## EPR‐077

### Resting‐state EEG analysis defines the signature of CACNA1A and GAA‐FGF14 related channelopathies

#### R. Angerbauer; I. Unterberger; W. Nachbauer; M. Amprosi; S. Boesch; M. Cesari; E. Indelicato

##### Department of Neurology, Medical University of Innsbruck, Innsbruck, Austria

**Background and aims:** CACNA1A variants and GAA‐FGF14 ataxia share overlapping neurological phenotypes, including chronic cerebellar signs and episodic ataxia. Sparse evidence linking CACNA1A mutations to altered resting‐state electroencephalogram (EEG) patterns exists, with no data for GAA‐FGF14. This study aimed to compare EEG metrics among these groups and healthy controls (HC).

**Methods:** Resting‐state EEGs were analyzed in 29 CACNA1A, 15 GAA‐FGF14 ataxia patients, and 30 HC. EEGs were recorded using the 10‐20 system, bandpass‐filtered (0.5–40 Hz), and segmented into 2‐second epochs. Artifacts were removed via independent component analysis. Relative bandpower was calculated using Welch's method, and functional connectivity was assessed via weighted Phase‐Lag Index (wPLI) and Minimum Spanning Tree (MST) metrics. Bayesian linear regression evaluated genotype effects, adjusting for age and sex differences. Priors were informed by separate healthy cohort data.

**Results:** Results are summarized in Table 1. Relative delta and theta power were increased in CACNA1A patients compared to both GAA‐FGF14 patients and HC (Figure 1a‐c). FGF14 patients consistently exhibited higher relative beta and gamma power than CACNA1A patients and HC (Figure 1d,e). Functional connectivity analysis revealed significantly increased overall wPLI in the theta and delta bands in CACNA1A patients compared to HC (Figure 2a). Additionally, MST Maximum Degree Centrality and in the delta band was increased in CACNA1A patients indicating higher degree of centralization (Figure 2b).

**Figure:**
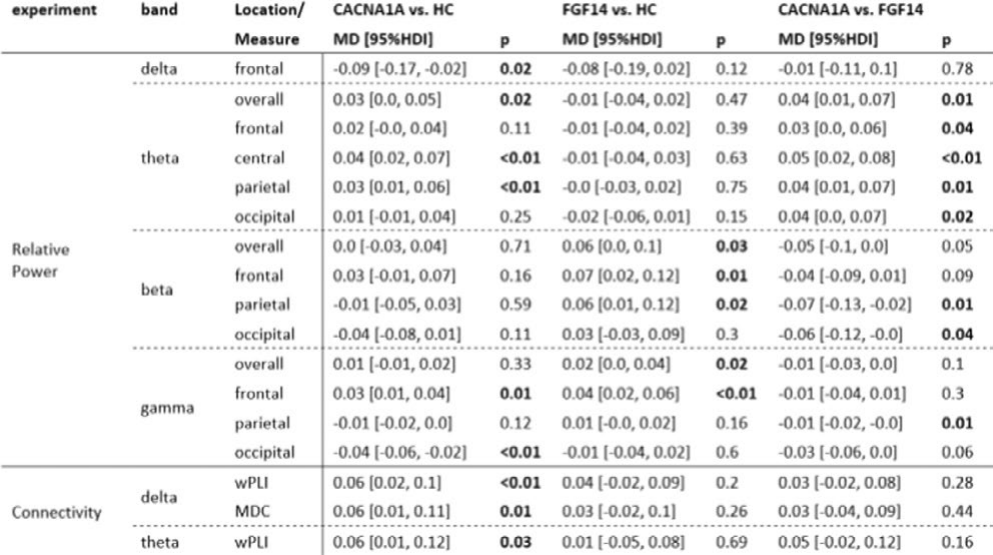
**TABLE 1** Summary of significant differences. Mean differences (MD) and the 95% Highest‐density intervals (95%HDI) are shown for each value. Significant *p*‐values (*p* < 0.05) are shown in bold.

**Figure:**
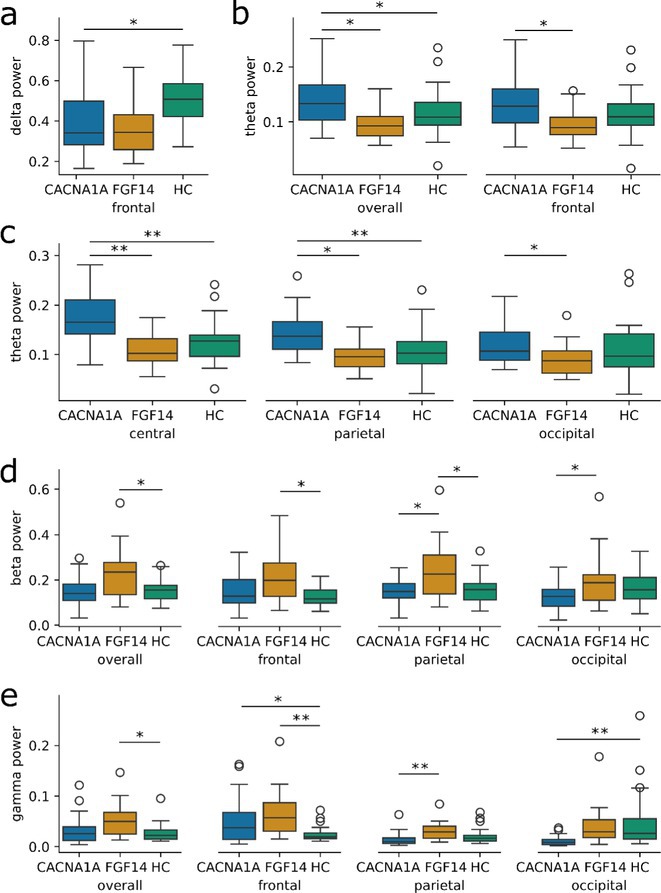
**FIGURE 1** Relative bandpower measures. The y‐axis represents the relative bandpower for each frequency band, while the x‐axis displays the groups, with the measurement locations indicated below. Panels show: (a) relative delta power, (b,c) relative theta.

**Figure:**
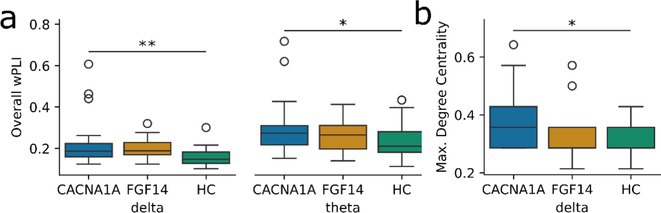
**FIGURE 2** Connectivity measures. The y‐axis represents the respective connectivity measure, while the x‐axis displays the groups, with the bands indicated below. Panels show: (a) Overall weighted Phase‐Lag Index (wPLI), and (b) Multiple Spanning Tree (MST).

**Conclusion:** Distinct resting‐state EEG alterations characterize CACNA1A and GAA‐FGF14 ataxias, reflecting unique network‐level effects of P/Q‐type calcium channel dysfunction. Advanced EEG analysis shows potential as a biomarker for calcium channelopathies.

**Disclosure:** This study was supported by the intramural funding program of the Medical University of Innsbruck for young scientists MUI‐START, Project 2022‐1‐3.

## EPR‐078

### Biallelic variants in alkaline ceramidase 3 cause infantile and early childhood onset neurodegeneration

#### R. Kaiyrzhanov ^1^; R. Maroofian^2^; A. ACER3‐related disease Study Group^3^

##### ^1^Department of Neurology, South Kazakhstan Medical Academy, Shymkent, Kazakhstan; ^2^Department of Neuromuscular Diseases, UCL Institute of Neurology, Queen Square, London, UK; ^3^Various international institutions

**Background and aims:** Biallelic variants in Alkaline Ceramidase 3 (ACER3) have recently been linked to early‐onset leukodystrophy in 3 case reports describing 5 families with 7 patents. Here we describe previously unreported 57 patients from 54 unrelated families with biallelic variants in ACER3 and cumulatively delineate the phenotypic spectrum of ACER3‐related.

**Methods:** Exome sequencing, data sharing, screening the genetic databases of several international genetic laboratories, and GeneMatcher were used to identify the patients here. ACER3 enzyme activity, lipidomics in patient fibroblasts, mutagenesis, functional assays, and protein modeling were performed.

**Results:** The cohort is composed of 64 patients from 59 families including 34 females and 30 males. 45 individuals are currently alive with a mean age of 6.6 ± 4.9 years (range 1.4‐18). 19 patients (30%) died between the ages of 3 and 19 years due to the rapidly progressive disease. The disease presents with predominantly infantile‐onset (86%), moderate (52%) and rapidly (39%) progressive neurological deficit manifesting with global developmental delay (71%) or developmental regression (98%)/stagnation (83%) commonly resulting in limbs spasticity (93%), limb dystonia (72%) and axial hypotonia (73%) with invariable posterior gradient white matter signal changes and diffuse cerebral volume loss (73%) on neuroimaging (Figure 1). ACER3 variants are presented in Figure 2. Functional studies reveal significantly reduced ceramide hydrolysis. Mutant analysis identifies functional hot spots, with in vitro assays showing a 2–40% decrease in ceramidase activity. Lipidomic analysis confirms a 50% increase in ceramide and sphingomyelin levels, alongside reduced sphingosine (Figure 3).

**Figure:**
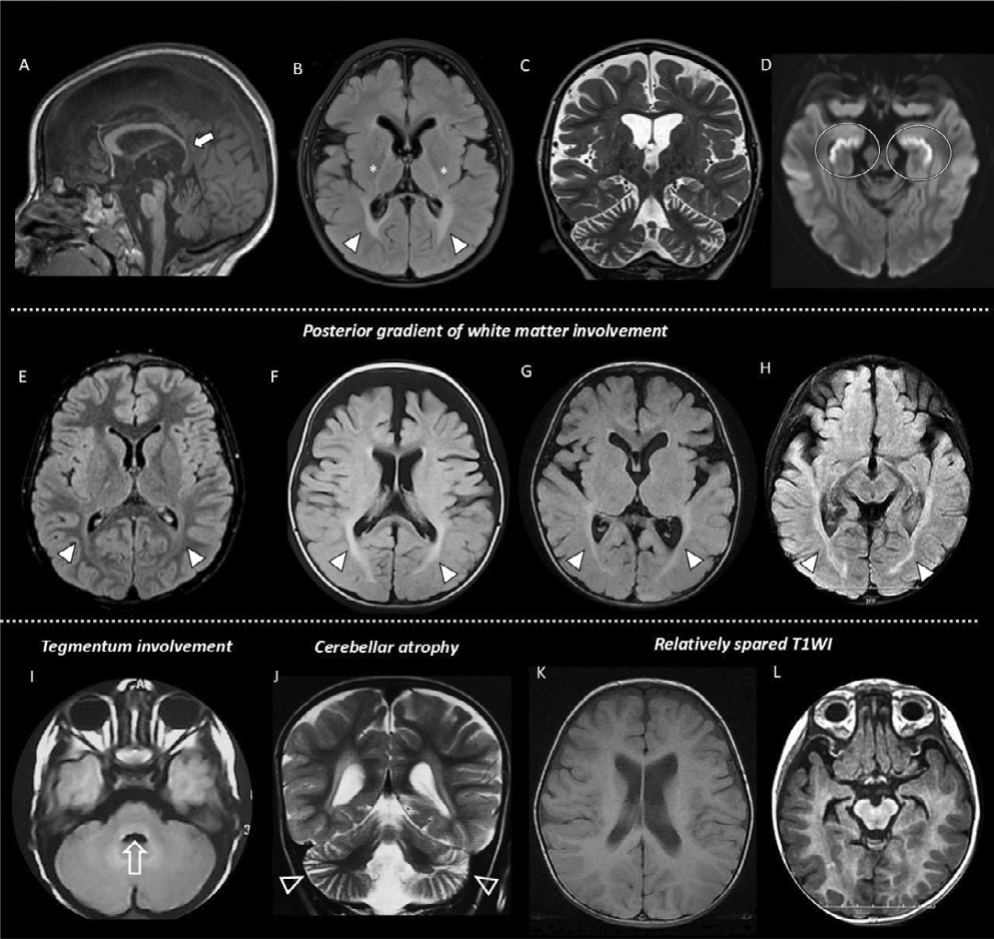
**FIGURE 1** Brain MRIs of the cohort showing white matter abnormalities.

**Figure:**
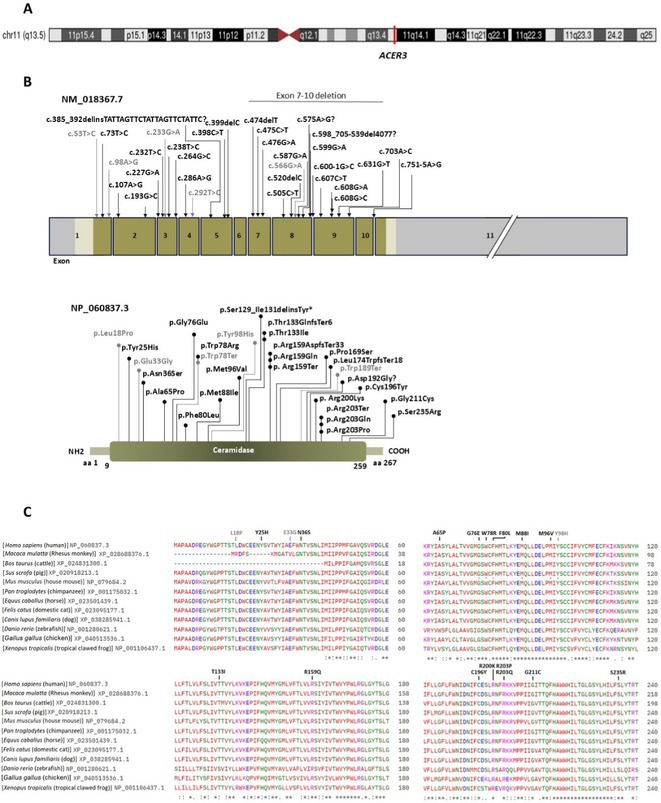
**FIGURE 2** Human chromosome 11 ideogram showing the locus of the ACER3 gene on Chr11q13.5. Schematic representation of ACER3 MAEN transcript (NM_018367.7) and protein. M (NP_060837.3) indicating the approximate positions of the variants identified in the families.

**Figure:**
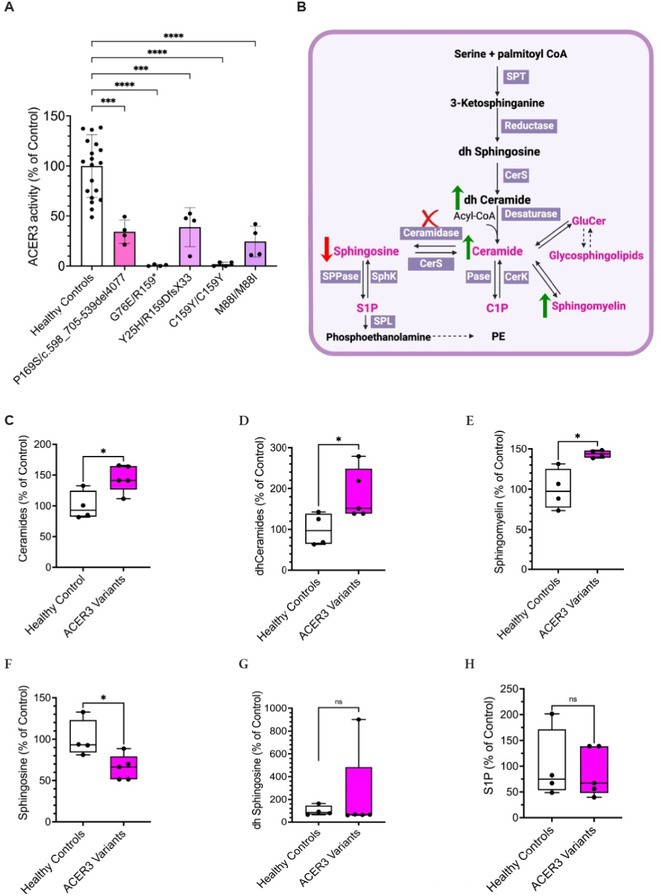
**FIGURE 3** ACER3 ceramidase enzyme activity was determined from skin fibroblasts from four healthy controls and five separate patient skin fibroblast cell lines

**Conclusion:** Biallelic variants in ACER3 are associated with infantile/childhood‐onset neurodegeneration with progressive leukodystrophy.

**Disclosure:** Nothing to disclose.

## EPR‐079

### NBIA diagnosis: Lessons from ten years experience in a French reference lab

#### V. Gioiosa ^1^; C. Angelini^1^; I. Coupry^2^; J. Deforges^3^; P. Fergelot‐Maurin^2^; J. Lasserre^2^; C. Plaisant^3^; G. Stevanin^2^; C. Goizet^1^

##### ^1^French reference center for rare diseases – Neurogenetics, Medical genetic department, Pellegrin Hospital, CHU Bordeaux, Bordeaux, France; ^2^University of Bordeaux, CNRS, INCIA, UMR 5287, NRGen Team, Bordeaux, France; ^3^Reference laboratory for NBIA diagnosis, molecular genetics laboratory, Pellegrin Hospital, CHU Bordeaux, Bordeaux, France

**Background and aims:** Neurodegeneration with Brain Iron Accumulation (NBIA) is a group of inherited disorders characterized by abnormal iron accumulation in brain's basal ganglia.

**Methods:** The DNA of 319 patients with clinical and MRI characteristics compatible with a diagnosis of NBIA was examined. A brain MRI was requested for each patient and clinical dataware was collected. A panel Next Generation Sequencing of 9 genes was performed, including: ATP13A2, CP, DCAF17, FA2H, FTL, C19orf12, PANK2, PLA2G6, and WDR45. For patients with negative NBIA panel, a trio exome or genome analysis has been requested.

**Results:** The molecular diagnosis of NBIA could be established for 72 (23 %). For each subtype, the age of onset of the first symptoms, the nature of these symptoms, and the main clinical manifestations were evaluated. Actually, 4 families presented with dominant MPAN, including mosaic carriers of the familial variant displayng late onset presentation in two of them. They are the only demonstrated mosaic cases reported to date in the C19ORF12 gene (3). Post‐zygotic mosaicism could also explain the presence in our cohort of two male monozygotic twins, where only one of them is symptomatic for a WDR45 variant. We have also identified an atypical form of Woodhouse Sakati syndrome, with a patient carrying composite heterozygous variants with the first description of a missense variant, in trans with a classical non sense variant, and an attenuated phenotype.

**Figure:**
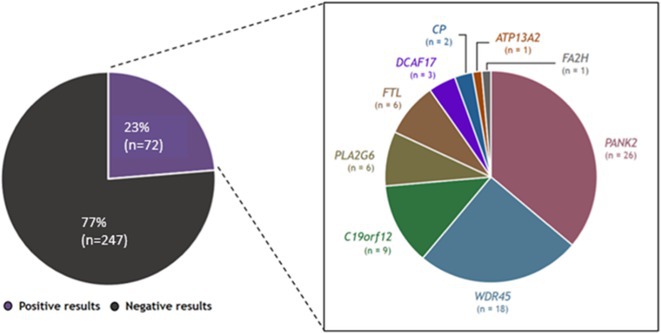
**FIGURE 1** Table

**Conclusion:** Our work aimed to provide a precise description of patients affected by NBIA, and to identify new genetic causes of iron accumulation.

**Disclosure:** Nothing to disclose.

## EPR‐080

### Trio‐WES in pediatric‐onset epilepsy: Assessing diagnostic yield and widening genotypic landscape

#### V. Yahya ^1^; E. Monfrini^2^; M. Molisso^3^; E. Zirone^3^; R. Del Bo^1^; A. Giacobbe^4^; E. Mauri^3^; L. Borellini^3^; A. Di Fonzo^2^; R. Dilena^3^

##### ^1^Dino Ferrari Center, Department of Pathophysiology and Transplantation, University of Milan, Milan, Italy; ^2^Neurology Unit, Fondazione IRCCS Ca’ Granda Ospedale Maggiore Policlinico, Milan, Italy; ^3^Neurophysiopathology Unit, Fondazione IRCCS Ca’ Granda Ospedale Maggiore Policlinico, Milan, Italy; ^4^Child and Adolescent Neuropsychiatry Service, Fondazione IRCCS Ca’ Granda Ospedale Maggiore Policlinico, Milan, Italy

**Background and aims:** Whole‐exome sequencing (WES) is a cost‐effective genetic test for epilepsy with an expected diagnostic yield of ~32%. We aimed to assess the diagnostic yield of trio‐based WES in pediatric‐onset epilepsy, identify novel variants associated with epilepsy, and evaluate the clinical impact of genetic diagnosis.

**Methods:** 30 patients with pediatric‐onset epilepsy were enrolled in EPIEXOME, a prospective monocentric study on genetic epilepsies, and underwent trio‐WES. The variants were classified according to ACMG criteria.

**Results:** The cohort included 11 patients with isolated epilepsy and 19 with cognitive and/or motor disturbances. Epilepsy was focal in 15/30 and generalized in 15/30 cases. Mean age at onset was 5.75 years [0‐16]. Trio‐WES resulted positive in 11/30 (37%, table 1), uncertain in 8/30, and negative in 11/30 cases. Among positive cases, 7 variants were de novo, one variant was inherited in autosomal dominant fashion by an affected father, 3 cases were recessive.

**Figure:**
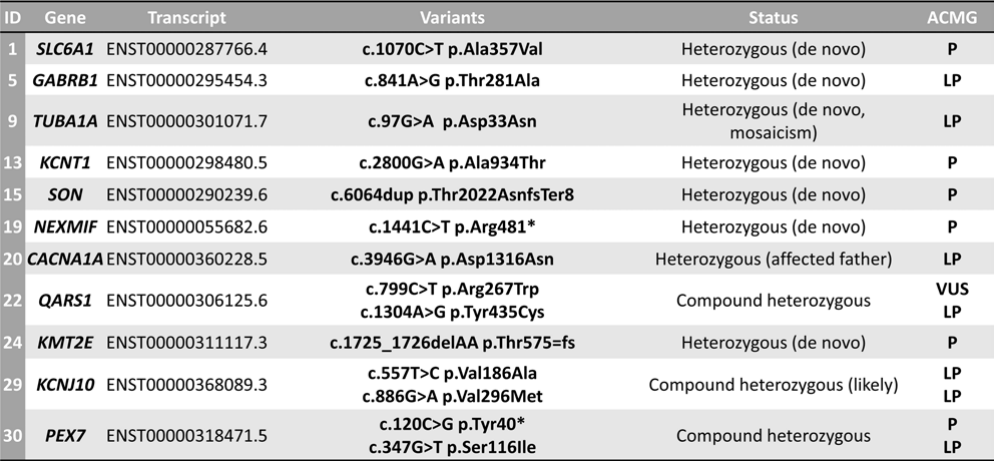
**TABLE 1** Variants identified in positive cases. Abbreviations: P = Pathogenic; LP = Likely pathogenic; VUS = variant of uncertain significance.

**Conclusion:** In a monocentric cohort, trio‐WES achieved a diagnostic result in 37% of the cases. The high representation of de novo variants stresses the relevance of trio analysis in epilepsy. We link GABRB1 gene with two novel epileptic phenotypes (EIMFS and Lennox‐Gastaut Syndrome) and report a case of steroid‐responsive para‐infectious acute encephalopathy in a patient with biallelic QARS1‐variants. The clinical impact of genetic diagnosis included personalized therapeutic possibilities (KCNT1, CACNA1A, QARS1), reverse phenotyping (SON), and adhesion to patient advocacy groups (SLC6A1).

**Disclosure:** Nothing to disclose.

## Neuroimmunology 1

## EPR‐081

### Single institution experience with efgartigimod in patients with GFAPA: Treatment response and adverse events

#### Z. Li

##### Department of Neurology, The Third Affiliated Hospital of Sun Yat‐sen University, Guangzhou, China

**Background and aims:** Autoimmune glial fibrillary acidic protein astrocytopathy (GFAPA) is a novel autoimmune central nervous system (CNS) disorder, some patients respond poorly to conventional immunotherapy. Our study revealed a real‐world experience with efgartigimod in treating GFAPA, which is expected to provide safety and effectiveness data for efgartigimod.

**Methods:** We conducted a retrospective analysis of 36 patients diagnosed with GFAPA from Jan 2021 to July 2024. Patients were divided into two groups: those who received efgartigimod treatment (*n* = 16) and those who did not (*n* = 20). Clinical outcomes were assessed via the modified Rankin Scale (mRS), Clinical Assessment Scale in Autoimmune Encephalitis (CASE), clinical symptoms, and Glasgow Coma Scale (GCS), along with analysis of treatment‐emergent adverse events (TEAEs), cerebrospinal fluid (CSF) total protein, leukocyte, and antibody titers, and blood serum IgG levels.

**Results:** Compared with the control, efgartigimod treatment group was associated with clinical improvement, as evidenced by significantly greater reductions in CASE scores at discharge (*p* < 0.05) and apparent decreases in CASE scores at follow‐up (*p* = 0.08), improvements in GCS scores at discharge and follow‐up. Additionally, patients receiving efgartigimod presented significant reductions in CSF total protein, leukocyte, and anti‐GFAP antibody titers and serum IgG levels. The most common TEAEs were mild to moderate infections, with no significant safety concerns identified.

**Conclusion:** Efgartigimod was generally safe for patients with GFAPA and appeared to accelerate the recovery of clinical symptoms and neurological function. However, further prospective randomized studies with larger patient cohorts are needed to confirm the safety and efficacy of efgartigimod.

**Disclosure:** Nothing to disclose.

## EPR‐082

### Super‐refractory Anti‐NMDAR encephalitis: Clinical features, risk factors and prognostic outcomes

#### I. Elosua‐Bayés ^1^; P. Dumez^2^; A. Farina^2^; M. Villagrán‐García^2^; E. Peter^2^; M. Benaiteau^2^; G. Picard^2^; N. Ciano‐Peterson^2^; L. Do^2^; V. Rogemond^2^; D. Gonçalves^3^; D. Psimaras^4^; J. Honnorat^2^; B. Joubert^2^

##### ^1^Neurology Department and Centre d'Esclerosi Múltiple de Catalunya (Cemcat), Hospital Universitari Vall d'Hebron, Universitat Autònoma de Barcelona, Barcelona, Spain; ^2^French Reference Center on Paraneoplastic Neurological Syndromes and Autoimmune Encephalitis, Hospices Civils de Lyon, Hôpital Neurologique, Bron, France.; ^3^Service d'Anatomie Pathologique, Hospices Civils de Lyon, Centre Hospitalier Lyon Sud, UMR CNRS Université de Lyon 1, Lyon, France; ^4^Service de Neurologie 2‐ Mazarin, Sorbonne Université, Inserm, CNRS, UMR S 1127, Institut du Cerveau, ICM, AP‐HP, Hôpitaux Universitaires La Pitié Salpêtrière ‐ Charles Foix, 75013, Paris, France.

**Background and aims:** Anti‐NMDAR encephalitis (NMDARE) is the most common autoimmune encephalitis, typically responding well to immunotherapy. Frequency, risk factors, and outcomes associated with severe clinical status after second‐line immunotherapy are unknown. This study aimed to define and characterize super‐refractory NMDARE.

**Methods:** Retrospective study including patients diagnosed with non‐herpetic NMDARE at the French national reference center between October 2007 and February 2022, with admission to ICU. Super‐refractory NMDARE was defined as ICU hospitalization lasting more than 90 days after second‐line immunotherapy, excluding those deceased within 90 days of treatment. Favorable outcome was defined as mRS < 2 at 24 months of onset. Clinical and paraclinical features were compared between super‐refractory and ICU‐admitted NMDARE.

**Results:** Of 219 NMDARE patients admitted to the ICU, 26 (11.45%) met criteria for super‐refractory (92% females, median age 24 years). Non‐Caucasian ethnicity was more prevalent (56% vs. 35%, *p* = 0.040) in super‐refractory patients, who presented more frequent intubation (62% vs. 100%, *p* < 0.001), seizures (87% vs. 100%, *p* = 0.051), movement disorders (82% vs. 100%, *p* = 0.018) and dysautonomia (62% vs. 85%, *p* = 0.023). Cerebrospinal fluid pleocytosis [cell‐count > 70 (46% vs. 21%, *p* = 0.008)], extreme delta brush pattern (21% vs. 7.1%, *p* = 0.041) and ovarian teratomas (62% vs. 22%, *p* < 0.001) were also more frequent. This group received earlier first and second‐line immunotherapy (*p* < 0.05) despite poorer outcomes (72% vs. 39%, *p* = 0.004) and higher mortality (19% vs. 6.7%, *p* = 0.046).

**Conclusion:** Super‐refractory NMDARE represents a distinct, high‐risk subgroup, marked by a severe clinical presentation, high prevalence of ovarian teratoma and poorer outcomes. Early identification and targeted management strategies are essential for improving its prognosis.

**Disclosure:** Nothing to disclose.

## EPR‐083

### Distinguishing primary angiitis of the central nervous system from its histopathological mimics

#### I. Elosua‐Bayés ^1^; A. Vilaseca^1^; A. Zabalza^1^; J. Camacho^2^; M. Roig^2^; J. Rio^1^; F. Martínez‐Valle^3^; J. Sastre‐Garriga^1^; M. Tintoré^1,4^; X. Montalban^1,4^; H. Ariño^1^; E. Martínez‐Saez^2^

##### ^1^Centre d’Esclerosis Múltiple de Catalunya, Departament of Neurology, Hospital Universitari Vall d’Hebron, Barcelona, Spain,^2^Pathological Anatomy Department, Hospital Universitari Vall d’Hebron, Barcelona, Spain,^3^Internal Medicine Department, Hospital Universitari Vall d’Hebron, Barcelona, Spain,^4^Universitat de Vic‐ Universitat Central de Catalunya (UVic‐UCC), Spain

**Background and aims:** Primary angiitis of the central nervous system (PACNS) has a challenging anatomopathological diagnosis due to its heterogeneity and secondary vascular inflammation present in other conditions. This study aimed to compare PACNS with histologically mimicking conditions, identifying features that may aid in differential diagnosis.

**Methods:** Retrospective case‐control study including 9 PACNS and 12 mimicking conditions (CNS infections, inflammatory diseases, or systemic affections). Using digital pathology (QPath), we analyzed CD3, CD20, and CD45 stains. Granulomas, fibrinoid necrosis, and microglial nodules were assessed. The five most inflamed medium‐sized vessels (excluding meningeal) were selected, creating three concentric zones: transmural, intermediate, and outer (fixed size). Lymphocyte densities were compared using *U*‐Mann–Whitney and mixed linear regression (accounting repeated measures). CD3 lymphocyte transmural density was analyzed with a ROC curve.

**Results:** PACNS presented granulomas more frequently (56.0% vs. 8.3%; *p* = 0.046), tending to exhibit higher transmural inflammation (lymphocytes/mm^2^) in CD3 staining [median: 5237 (IQR: 3958–6440) vs. 3410 (IQR: 3078–3559), *p* = 0.059], CD20 [median: 2215 (IQR: 1428–3618) vs. 473 (IQR: 0.00–2205), *p* = 0.117], and total lymphocytes [median: 7475 (IQR: 5892–10569) vs. 4183 (IQR: 3629–6115), *p* = 0.091]. Mixed analysis highlights higher transmural density respect other regions in PACNS (CD3: *p* = 0.0198; CD20: *p* = 0.1199; total: *p* = 0.0553). The ROC curve showed an AUC of 77.78%, with an optimal cutoff at 3509.48 CD3 transmural density (sensitivity: 0.88; specificity: 0.67) to discriminate PACNS from mimics.

**Conclusion:** Despite no significant differences in inflammation patterns between groups, granuloma presence, and transmural inflammation, predominantly CD3 lymphocytes, was greater in the PACNS, which may aid in histological diagnosis.

**Disclosure:** Nothing to disclose.

## EPR‐084

### Efgartigimod in the treatment of anti‐NMDAR encephalitis compared with IVIG and SPA‐IA during acute attacks

#### J. Liu ^1^; M. Li^1^; J. Liu^1^; D. Zheng^2^; Y. Zhou^3^; Y. Li^4^; X. Chen^5^; Y. Lin^6^; L. Yang^1^; X. Xu^1^; Y. Jiang^1^; F. Peng^1^

##### ^1^Department of Neurology, The Third Affiliated Hospital of Sun Yat‐sen University; ^2^Department of Neurology, The Affiliated Brain Hospital, Guangzhou Medical University; ^3^Department of Neurology, Shenzhen Second Peopleʼs Hospital; ^4^Department of Neurology, Sun Yat‐Sen Memorial Hospital of Sun Yat‐Sen University; ^5^Department of Neurology, Dong Guan Kang Hua Hospital; ^6^Department of Neurology, Yulin Frist People's Hospital

**Background and aims:** The purpose of this study was to evaluate the efficacy of Efgartigimod (EFG) in anti‐N‐methyl‐D‐aspartate receptor (anti‐NMDAR) encephalitis patients during acute attacks.

**Methods:** A case‐control study was designed to compare 26 anti‐NMDAR encephalitis patients who were treated with EFG, and 15 patients with intravenous immunoglobulin (IVIG), and 23 patients with immunoadsorption with staphylococcal protein A column (SPA‐IA) treatment.

**Results:** At baseline, no significant differences in mRS scores were observed among the EFG, IVIG, and SPA‐IA groups of anti‐NMDAR encephalitis patients. When compared with the IVIG group, patients treated with EFG had significantly decreased serum IgG levels (*p* = 0.002). Compared with the SPA‐IA group, EFG‐treated patients had lower CSF anti‐NMDAR antibody titers at admission (*p* = 0.039) and higher post‐treatment IgG levels (*p* = 0.002). In the EFG and SPA‐IA groups, there was a significant reduction in anti‐NMDAR antibody titers in both CSF and serum (*p* < 0.01), while no remarkable decrease was found in the IVIG group (*p* > 0.05). Additionally, serum IgG levels significantly decreased in both the EFG and SPA‐IA groups at baseline and during the 1‐month follow‐up. By the third month of follow‐up, IgG levels in the blood of both groups remained below the baseline levels.

**Conclusion:** EFG could be an elegant alternative to both IVIG and SPA‐IA therapies for anti‐NMDAR encephalitis during acute attacks. It has a better effect on reducing antibody titers than IVIG and is comparable to SPA‐IA therapy, and no serious adverse events were observed during infusion.

**Disclosure:** The authors declare that they have no competing interests.

## EPR‐085

### Safety and efficacy of riliprubart in chronic inflammatory demyelinating polyneuropathy: 76‐week phase 2 trial results

#### L. Querol^1^; R. A. Lewis^2^; H. ‐P. Hartung^3^; P. A. van Doorn^4^; J. Lin^5^; A. Dionne^6^; S. Attarian^7^; E. Wallstroem^8^; K. Auwarter^9^; Y. Lu^10^; M. Alonso Alonso ^8^; N. Atassi^8^; R. A. C. Hughes^11^; R. in CIDP Phase 2 Trial Group^12^

##### ^1^Neuromuscular Diseases Unit, Department of Neurology, Hospital de la Santa Creu i Sant Pau, Barcelona, Spain; Centro para la Investigación Biomédica en Red en Enfermedades Raras (CIBERER), Madrid, Spain; ^2^Department of Neurology, Cedars Sinai Medical Center, Los Angeles, CA, USA; ^3^Department of Neurology, Heinrich‐Heine‐University, Germany; Brain and Mind Center, University of Sydney, Australia; Department of Neurology, Medical University of Vienna, Austria; Department of Neurology, Palacky University Olomouc, The Czechia; ^4^Erasmus MC, University Medical Center, Rotterdam, The Netherlands; ^5^Department of Neurology and Rare Disease Center, Huashan Hospital, Fudan University, Shanghai, China; ^6^CHU de Quebec Universite Laval, Quebec, Canada; ^7^Neuromuscular Disease and ALS Reference Center, Timone University Hospital, Aix‐Marseille University, CHU Timone, Marseille Cedex 05, France; ^8^Sanofi R&D, Neurology Development, Cambridge, MA, USA; ^9^Sanofi, USA, ^10^Sanofi R&D, Biostatistics and Programming, Cambridge, MA, USA, ^11^UCL Queen Square Institute of Neurology, University College London, London, UK, ^12^Riliprubart Phase 2 PDY16744 Trial Group on behalf of participating investigational sites, International.

**Background and aims:** Riliprubart, a first‐in‐class humanized IgG4‐monoclonal antibody, selectively inhibits activated‐C1s in the classical‐complement pathway (Figure) and can be self‐administrated subcutaneously via an auto‐injector. Preliminary results from an ongoing Phase‐2 trial evaluating riliprubart in participants with chronic inflammatory demyelinating polyneuropathy (CIDP; NCT04658472) suggested encouraging clinical‐benefits and safety‐profile up to 48‐weeks. Here, we report efficacy and safety results of riliprubart at Week‐76.

**Figure:**
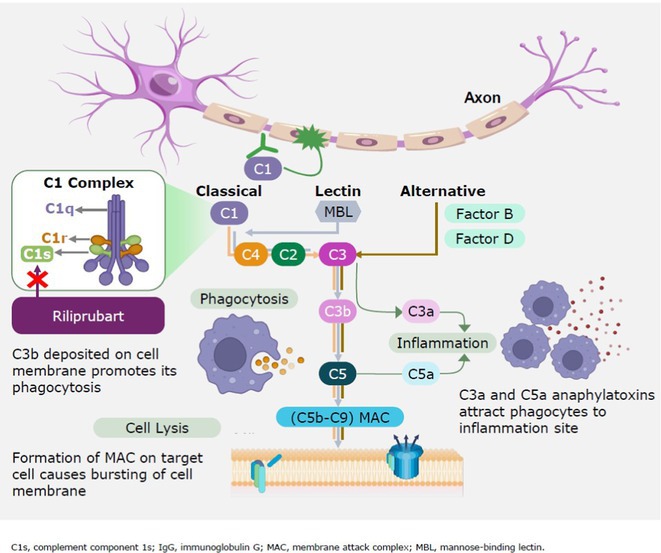
**FIGURE 1** Riliprubart targeting C1s and the classical complement pathway.

**Methods:** This open‐label, Phase‐2 trial evaluates riliprubart across three groups: Standard‐of‐care (SoC)‐Treated, SoC‐Refractory, and SoC‐Naïve. Participants undergo 24‐week treatment (Part‐A), followed by an optional treatment‐extension (Part‐B: 52‐weeks). Primary‐endpoint (Part‐A) is %‐participants relapsing (SoC‐Treated) or responding (SoC‐Refractory/Naïve), defined as ≥1‐point change in adjusted‐Inflammatory Neuropathy Cause and Treatment (INCAT) disability score. Part‐B evaluates safety and efficacy‐durability based on %‐relapse‐free participants (SoC‐Treated) or with sustained‐response (SoC‐Refractory/Naïve), defined as no‐increase in adjusted‐INCAT score ≥2‐points at Week‐76 relative to Week‐24. Exploratory‐endpoints include additional efficacy measures (INCAT, IRODS, MRC‐SS, grip‐strength), change in total‐complement, and plasma neurofilament‐light chain‐levels.

**Results:** As of August‐2024, in SoC‐Treated group, 81.3% (*N* = 39/48) participants entered Part‐B, including 47.9% (*N* = 23/48) completed Part‐B treatment‐period (ongoing:27.1%; discontinued:6.3%). In SoC‐Refractory and SoC‐Naïve groups, 72.2% (*N* = 13/18) and 50% (*N* = 6/12) participants, respectively, entered Part‐B, including 61.1% (*N* = 11/18) in SoC‐Refractory (ongoing:0%; discontinued:11.1%) and 16.7% (*N* = 2/12) in SoC‐Naïve group (ongoing:8.3%; discontinued:25%) completing Part‐B. Updated full Part‐B efficacy and safety data up to Week‐76 will be presented at meeting.

**Conclusion:** Mature Week‐76 results may suggest potential for riliprubart to demonstrate sustained clinical effect in participants who experience failure/inadequate response/residual disability despite SoC therapy, supporting its development in Phase‐3, and potentially offering new treatment option for CIDP.

**Disclosure:** LQ‐Research grants: Instituto de Salud Carlos III–Ministry of Economy and Innovation (Spain), CIBERER, Fundació La Marató, GBS‐CIDP Foundation International, UCB, Grifols. Received speaker/expert testimony honoraria: CSL Behring, Novartis, Sanofi, Merck, Annexon, Alnylam, Biogen, Janssen, Lundbeck, argenx, UCB, Dianthus, LFB, Avilar Therapeutics, Octapharma, Roche. Serves:Clinical Trial Steering Committee for Sanofi, Principal Investigator: UCB's CIDP01 trial. RAL‐Consultant: CSL Behring, BioCryst, Dianthus, Grifols, Nuvig, Pfizer, Sanofi (Steering Committee), Annexon, Alexion, Avilar, argenx, J&J, Takeda, Boehringer Ingelheim (DSMB), Intellia (DSMB), Nervosave, TGTX, Seismic and medical advisory board GBS‐CIDP Foundation International. Receives royalties: UptoDate; speaker: Medscape. HPH‐Consultant: Sanofi, Octapharma. Received fees:serving on Steering and Data Monitoring Committees from Biogen, BMS Celgene, GeNeuro, Merck, Novartis, Octapharma, Roche TG Therapeutics. PAvD‐Consultant: Annexon, argenx, Hansa Biopharma, Immunic, Octapharma, Roche, Sanofi (Institutional research fund received all honoraria), grants: Prinses Beatrix Spierfonds, Sanquin, Grifols. JL‐None. AD‐Received honoraria: argenx and Alexion for conference, ad board. SA‐Consultant:Alexion, argenx, UCB, Janssen, Hansa Biopharma, Roche, Sanofi, Amicus, LFB, Alnylam, Astrazeneca, Pfizer, Biogen. EW,KA,YL,MAA,NA‐Employees of Sanofi, may hold shares and/or stock options in company. RACH‐Consultant: Hansa Biopharma, Sanofi.

## EPR‐086

### Efficacy of Riliprubart in chronic inflammatory demyelinating polyneuropathy: Phase 2 subgroup analyses

#### R. A. Lewis^1^; L. Querol^2^; H. ‐P. Hartung^3^; P. A. van Doorn^4^; J. Lin^5^; A. Dionne^6^; S. Attarian^7^; E. Wallstroem^8^; Y. Lu^9^; M. Alonso Alonso ^8^; N. Atassi^8^; R. A. C. Hughes^10^; R. in CIDP Phase 2 Trial Group^11^

##### ^1^Department of Neurology, Cedars Sinai Medical Center, Los Angeles, CA, USA; ^2^Neuromuscular Diseases Unit, Department of Neurology, Hospital de la Santa Creu i Sant Pau, Barcelona, Spain; Centro para la Investigación Biomédica en Red en Enfermedades Raras (CIBERER), Madrid, Spain; ^3^Department of Neurology, Heinrich‐Heine‐University, Germany; Brain and Mind Center, University of Sydney, Australia; Department of Neurology, Medical University of Vienna, Austria; Department of Neurology, Palacky University Olomouc, Czechia; ^4^Erasmus MC, University Medical Center, Rotterdam, The Netherlands; ^5^Department of Neurology and Rare Disease Center, Huashan Hospital, Fudan University, Shanghai, China; ^6^CHU de Quebec Universite Laval, Quebec, Canada; ^7^Neuromuscular Disease and ALS Reference Center, Timone University Hospital, Aix‐Marseille University, CHU Timone, Marseille Cedex 05, France; ^8^Sanofi R&D, Neurology Development, Cambridge, MA, USA; ^9^Sanofi R&D, Biostatistics and Programming, Cambridge, MA, USA, ^10^UCL Queen Square Institute of Neurology, University College London, London, UK, ^11^Riliprubart Phase 2 PDY16744 Trial Group on behalf of participating investigational sites, International

**Background and aims:** Riliprubart, a first‐in‐class humanized IgG4‐monoclonal antibody, selectively inhibits activated‐C1s in the classical complement pathway (Figure) and can be self‐administrated subcutaneously, via an auto‐injector. In a Phase‐2 trial (NCT04658472), riliprubart treatment suggested encouraging clinical benefits and safety profile across a broad spectrum of participants with chronic inflammatory demyelinating polyneuropathy (CIDP). Here, we report subgroup efficacy analyses from this ongoing trial.

**Figure:**
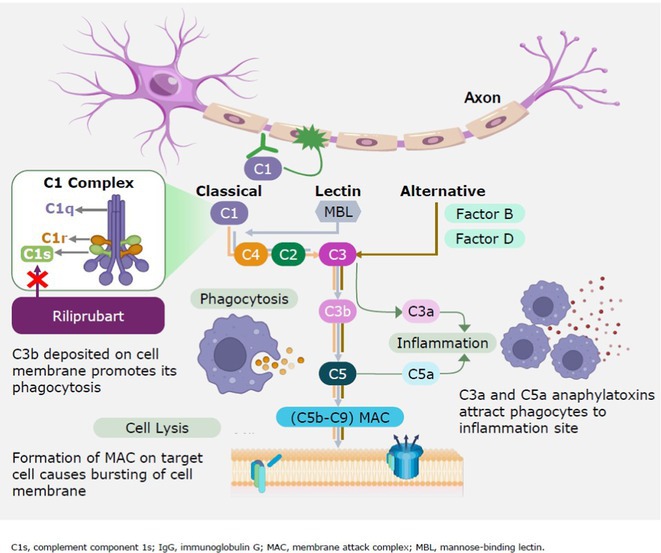
**FIGURE 1** Riliprubart targeting C1s and the classical complement pathway

**Methods:** This open‐label, Phase‐2 trial evaluates riliprubart across three groups: Standard‐of‐care (SoC)‐Treated, SoC‐Refractory, and SoC‐Naïve. Participants underwent 24‐week treatment (Part‐A), followed by an optional treatment extension (Part‐B: 52‐weeks). In Part‐A, post‐hoc analyses were performed in the standard‐of‐care (SoC)‐Treated and SoC‐Refractory groups, and all participants. Response rate for endpoints (INCAT, I‐RODS, MRC‐SS, grip strength) were evaluated in subgroups defined by standard demographics and clinical characteristics, possibly predictive for the overall outcome (age, sex, CIDP subtype, time since start of therapy/diagnosis, previous therapies, immunoglobulin [Ig] dose, baseline plasma neurofilament‐light chain [NfL], and INCAT score).

**Results:** As of April‐2024, preliminary interim data from 48 SoC‐Treated and 18 SoC‐Refractory participants (who completed or discontinued 24‐week treatment) were analyzed. In the overall population, the INCAT response rates were similar across all analyzed subgroups, indicating clinically meaningful benefits regardless of baseline characteristics. Consistent trends were observed across other efficacy measures. Available data of these results will be presented at the meeting.

**Conclusion:** The results suggest consistent clinical effects of riliprubart across a range of baseline characteristics and assessment scores for people living with CIDP who experience failure/inadequate response or residual disability despite SoC treatment.

**Disclosure:** RAL‐Consultant: CSL Behring, BioCryst, Dianthus, Grifols, Nuvig, Pfizer, Sanofi (Steering Committee), Annexon, Alexion, Avilar, argenx, J&J, Takeda, Boehringer Ingelheim (DSMB), Intellia (DSMB), Nervosave, TGTX, Seismic and medical advisory board GBS‐CIDP Foundation International. Receives royalties: UptoDate; speaker: Medscape. LQ‐Research grants: Instituto de Salud Carlos III–Ministry of Economy and Innovation (Spain), CIBERER, Fundació La Marató, GBS‐CIDP Foundation International, UCB, Grifols. Received speaker/expert testimony honoraria: CSL Behring, Novartis, Sanofi, Merck, Annexon, Alnylam, Biogen, Janssen, Lundbeck, argenx, UCB, Dianthus, LFB, Avilar Therapeutics, Octapharma, Roche. Serves: Clinical Trial Steering Committee for Sanofi, Principal Investigator: UCB's CIDP01 trial. HPH‐Consultant:Sanofi, Octapharma. Received fees:serving on Steering and Data Monitoring Committees from Biogen, BMS Celgene, GeNeuro, Merck, Novartis, Octapharma, Roche TG Therapeutics. PAvD‐Consultant: Annexon, argenx, Hansa Biopharma, Immunic, Octapharma, Roche, Sanofi (Institutional research fund received all honoraria), grants: Prinses Beatrix Spierfonds, Sanquin, Grifols. JL‐None. AD‐Received honoraria: argenx and Alexion for conference, ad board. SA‐Consultant: Alexion, argenx, UCB, Janssen, Hansa Biopharma, Roche, Sanofi, Amicus, LFB, Alnylam, Astrazeneca, Pfizer, Biogen. EW, YL, MAA, NA‐Employees of Sanofi, may hold shares and/or stock options in company. RACH‐Consultant: Hansa Biopharma, Sanofi.

## EPR‐087

### Healthcare resource utilization and costs associated with exacerbation or crisis in generalized myasthenia gravis

#### N. Silvestri^1^; K. Gandhi ^2^; M. Cloutier^3^; G. Coteur^4^; M. Zhdanava^3^; A. El Khoury^2^; P. Boonmak^3^; A. Tardif‐Samson^3^; M. Ait‐Thiyaty^5^; Y. Wang^3^; Z. Choudhry^6^; K. Grover^7^

##### ^1^University at Buffalo Jacobs School of Medicine & Biomedical Sciences, Buffalo, USA ^2^Johnson and Johnson, Raritan, USA; ^3^Analysis Group, Inc., Montreal, Canada, ^4^iPATH Solutions, BV, Wemmel, Belgium; ^5^Johnson and Johnson, Titusville, USA; ^6^Johnson and Johnson, Horsham, USA; ^7^Henry Ford Health System, Detroit, USA

**Background and aims:** Exacerbation and crisis are experienced by 38.5% and 2.1% of patients with generalized myasthenia gravis (gMG), respectively.1 While the overall burden of gMG is documented, the impact of exacerbation/crisis remains unclear, hindering efforts to address unmet healthcare needs.

**Methods:** Adults with incident gMG (MG diagnosis by a neurologist; 12‐month washout before the first MG diagnosis) and > = 1 clinical event (exacerbation: primary MG diagnosis in an inpatient or emergency setting or any exacerbation‐related diagnosis; or crisis: intubation) were identified from Komodo Research Database (01/2017‐09/2023). Index date was the first clinical event following gMG. Patients had > = 12 months of insurance coverage pre‐ and post‐index. Monthly per‐patient healthcare resource utilization (HRU) and costs were described 12 months pre‐ and post‐index.

**Results:** Among 2,657 patients (mean age: 61.1 years; female: 47.5%), the first clinical event (exacerbation: 98.2%; crisis: 1.8%) were reported predominantly in the outpatient setting (55.2%) and, on average, 3.8 months after gMG diagnosis. Mean monthly inpatient days (0.75 vs. 0.29) and outpatient visits (3.44 vs. 2.47) increased post‐index compared to pre‐index. Mean monthly total healthcare costs increased twofold ($7,985 vs. $2,939), driven by pharmacy ($3,711 vs. $723) and inpatient costs ($2,420 vs. $943). HRU and costs peaked during the first month post‐index; reaching $19,325, 3.21 inpatient days, and 4.88 outpatient visits and remained elevated compared to pre‐index.

**Figure:**
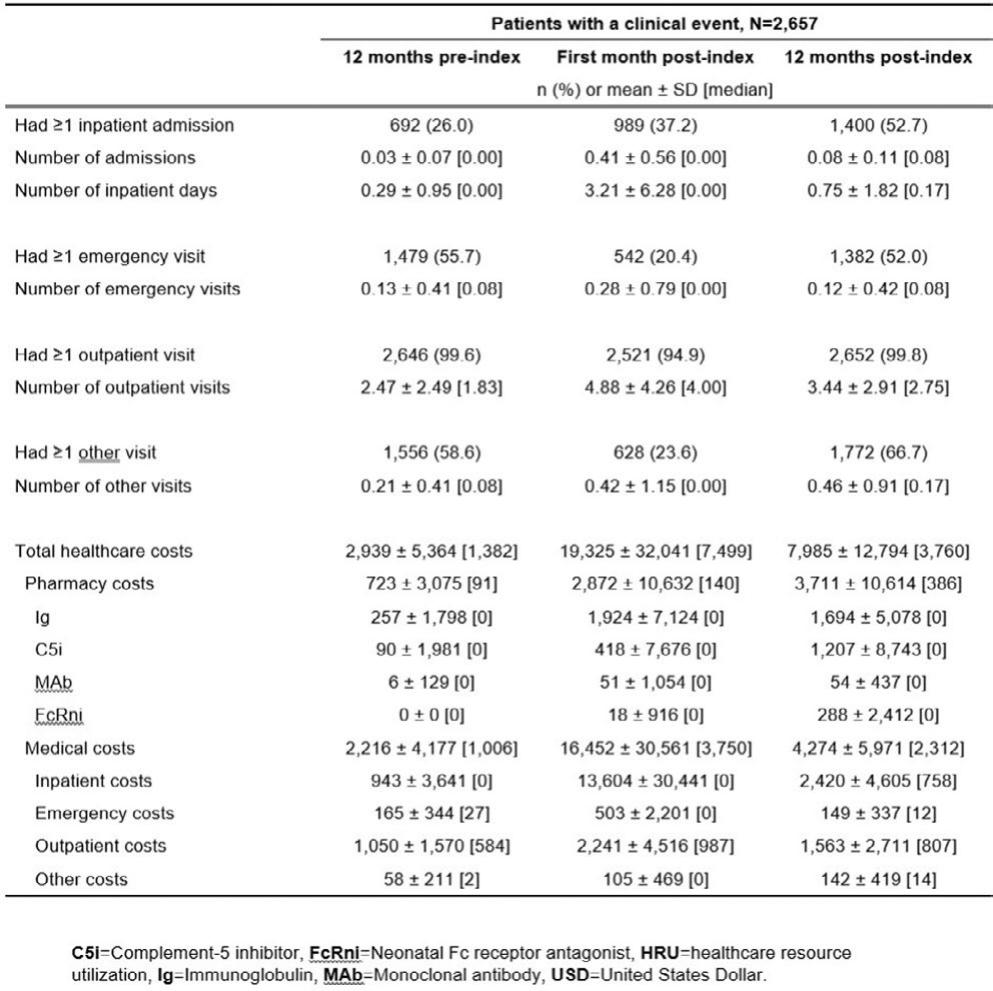
**TABLE 1** Monthly HRU and costs (2023 USD) pre‐ and post‐index clinical event.

**Figure:**
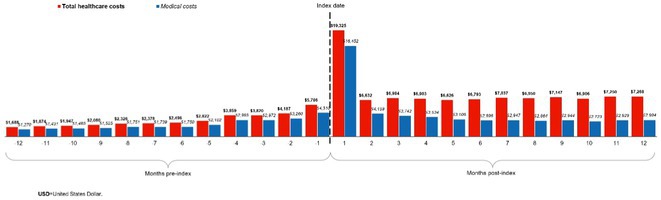
**FIGURE 1** Distribution of mean total healthcare and medical cost (2023 USD) pre‐ and post‐index.

**Conclusion:** Clinical events associated with gMG are resource‐intensive, with both acute and long‐term impacts. Findings underscore the importance of more effective and earlier treatments to achieve optimal disease control and reduce risk of crises/exacerbations.

**Disclosure:** This study was sponsored by Johnson & Johnson Innovative Medicine. Nicholas J. Silvestri and Kavita Grover have received personal compensation for serving as consultants for Johnson and Johnson. Kavita Gandhi, Antoine C El Khoury, Maria Ait‐Thiyaty and Zia Choudhry are employees of Johnson and Johnson and may hold stock/stock options of Johnson and Johnson. Martin Cloutier, Maryia Zhdanava, Porpong Boonmak, Anabelle Tardif‐Samson and Yuxi Wang are serving as employees of Analysis Group, Inc. Geoffroy Coteur is contractor for Johnson and Johnson.

## EPR‐088

### Clinical phenotypes of glycine receptor antibody encephalitis

#### S. Abu Hassan ^1^; J. Kerstens^1^; R. van Steenhoven^1^; M. Nagtzaam^1^; S. Veenbergen^2^; J. de Vries^1^; M. Titulaer^1^

##### ^1^Department of Neurology, Erasmus MC University Medical Center, Rotterdam, The Netherlands; ^2^Department of Immunology, Erasmus MC University Medical Center, Rotterdam, The Netherlands

**Background and aims:** Glycine receptor (GlyR) antibodies have initially been described in progressive encephalomyelitis with rigidity and myoclonus (PERM), and few reports have been published since of this rare entity with similar, but also different phenotypes. This study aimed to describe the clinical characteristics of patients with GlyR antibodies who were referred to our center.

**Methods:** Within our ERN‐accredited nationwide referral center, we included all patients who tested positive for GlyR antibodies in serum and/or cerebrospinal fluid (CSF), using an in‐house live cell‐based assay (serum 1:80, CSF 1:2). Patients were classified into the following phenotypes: PERM, rhombencephalitis, status epilepticus (SE), and chronic, focal epilepsy.

**Results:** We identified 20 patients with GlyR antibodies. One result was deemed clinically irrelevant (alternative diagnosis genetic leukodystrophy). Twelve patients (63%) were males, and median age was 56 (IQR 35, range 12‐83). PERM was the most common phenotype (*n* = 12, 63%), while two, two, and three patients had rhombencephalitis, SE, and epilepsy, respectively. Two patients had co‐existing phenotypes (the first being predominant): rhombencephalitis and PERM; PERM and SE. Brain imaging was often normal or non‐specific (14/17, 82%). Immunotherapy was effective in 9/13 assessable patients, five by first‐line immunotherapy (corticosteroids and intravenous immunoglobulins), and four after addition of rituximab. Two patients had underlying cancer, including mesothelioma treated with pembrolizumab, and sigmoid adenocarcinoma.

**Conclusion:** While patients with GlyR antibodies present largely with PERM, other phenotypes such as rhombencephalitis, status epilepticus, and chronic, focal epilepsy are also observed. Patients generally respond well to immunotherapy making an adequate and timely diagnosis essential.

**Disclosure:** S. Abu Hassan is an awardee of the Singhealth Health Manpower Development Plan by the Ministry of Health, Singapore that is funding her fellowship at Erasmus MC. The other authors have nothing to disclose.

## EPR‐089

### An open‐label trial of IVIg in autoimmune‐associated seizures: The AMICE study

#### Y. Crijnen^1^; S. Gefferie^2^; J. Kerstens^1^; S. Franken^1^; J. Brenner^1^; R. van Steenhoven^1^; S. Veenbergen^3^; P. Sillevis Smitt^1^; J. de Vries^1^; R. Thijs ^2^; M. Titulaer^1^

##### ^1^Department of Neurology, Erasmus University Medical Centre, Rotterdam, The Netherlands; ^2^Stichting Epilepsie Instellingen Nederland (SEIN), Heemstede, The Netherlands; ^3^Department of Immunology, Erasmus University Medical Centre, Rotterdam, The Netherlands

**Background and aims:** Autoimmune‐associated seizures (AAS) are most frequently refractory to anti‐epileptic drugs but often respond to immunotherapy. We assessed the efficacy of IVIg in AAS.

**Methods:** In this prospective open label trial with IVIg, we included patients with AAS ≥16 years, experiencing ≥1 seizure/week, and confirmed antibodies. All patients received two courses of IVIg (0.4 grams/kg/day day 1‐5 and 22‐26). The primary outcome measure was the proportion of patients with a seizure frequency reduction > 50% and the proportion achieving seizure freedom in Weeks 6–8. The secondary outcomes were clinical improvement, relapse rate and ≥50% seizure reduction at Weeks 16–18.

**Results:** Twenty‐six patients were included: 13 with antibodies against extracellular antigens (extracAE; *n* = 11 anti‐LGI1, *n* = 1 anti‐NMDAR, *n* = 1 anti‐Caspr2) and 13 with intracAE (all anti‐GAD, concentration > 10,000IU/ml). After IVIg, 14/26 (54%) had > 50% seizure frequency reduction, more frequent in extracAE patients (10 [77%] vs. 4 [31%], *p* = 0.024). Four extracAE patients achieved seizure freedom in Weeks 6–8, compared to zero intracAE patients (*p* = 0.048). ExtracAE patients had a 94% median reduction of seizure severity versus 4% in intracAE patients (*p* = 0.002). Still, 4/13 of the latter had > 50% seizure reduction after IVIg, while being refractory to anti‐seizure medication before. Recurrence of seizures was seen in 17/26 patients, necessitating renewed immunotherapy. At Week 18, 12 (92%) extracAE and 10 (77%) intracAE patients had > 50% seizure reduction.

**Figure:**
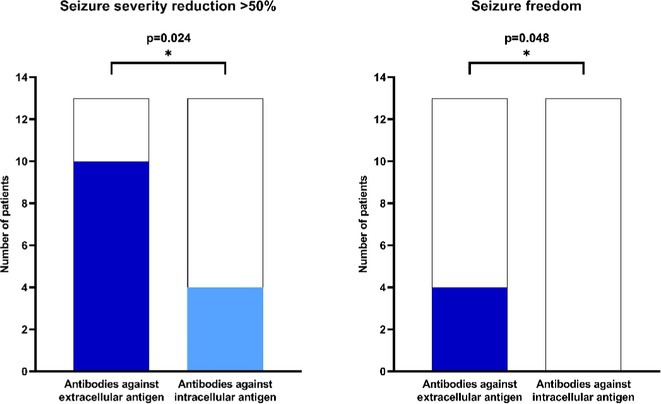
**FIGURE 1** Seizure freedom and > 50% seizure reduction in Weeks 6–8 compared to baseline. Number of patients with respectively > 50% seizure severity reduction and with seizure freedom in Weeks 6–8 (Days 36–56), compared to baseline.

**Figure:**
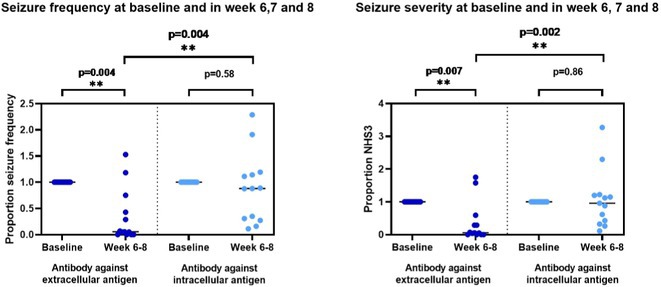
**FIGURE 2** Seizure frequency and severity at baseline and in Weeks 6–8. Respectively seizure frequency and seizure severity at baseline compared to Weeks 6–8, shown in proportions compared to baseline, and compared between patients with extra‐ and intracAE.

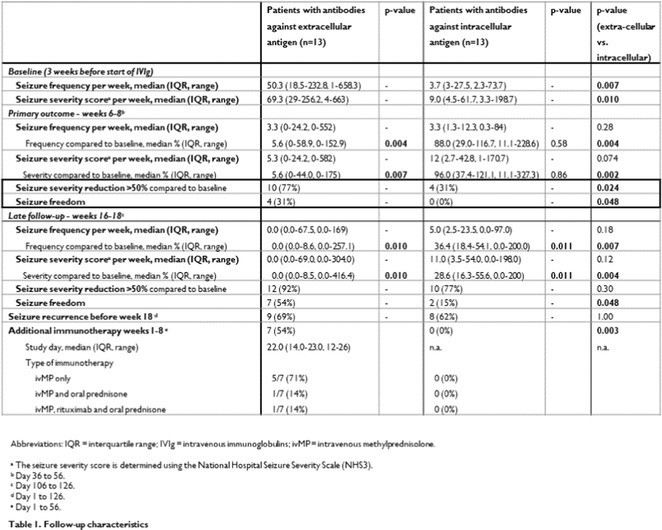

**Conclusion:** In patients with AAS, IVIg lead to reduction of seizures and seizure freedom, most pronounced in extracAE. Recurrence after waning effects of IVIg is common, supporting the need for longer‐lasting immunotherapies.

**Disclosure:** This study was funded by the Dutch Epilepsy foundation (EpilepsieNL, project 19‐08) and the Interlaken Leadership Award, an unrestricted research grant from CSL Behring.

## Neuropathies

## EPR‐090

### Nitrous oxide polyneuropathy: The new trend

#### A. Campos Villegas; C. Ortega Hiraldo; A. Gómez González; P. Hernández Vitorique; M. Vicente Domínguez; M. Mañez Sierra

##### Neurología, Hospital Universitario Virgen de la Victoria. Málaga, Spain

**Background and aims:** The prevalence of recreational use of nitrous oxide (N2O) is increasing among young people. NO2 is thought to alter neuronal membrane glutamate or gamma aminobutyric acid receptors, producing its effect within seconds of inhalation. N2O interferes with the metabolism of vitamin B12 and methionine, resulting in an increase in homocysteine concentration. Clinical manifestations include sensorimotor polyneuropathy, ataxia, myelopathy, and megaloblastic anemia, among others. Neurotoxicity is potentially reversible with vitamin B12 supplementation and N 2 O abstinence. Frequent findings on complementary tests include sensory/motor axonal involvement on electroneurography (ENG) or cervical spinal cord hyperintensity on MRI.

**Methods:** Description of three clinical cases (two males and one female) with a mean age of 48 years NO2 consumption.

**Results:** In this paper we discuss three clinical cases of three NO2‐consuming patients. Patient 1 reports frequent consumption, and 2 and 3 daily consumptions. All of them started with a similar clinical manifestation. On admission, laboratory tests were requested to evaluate vitamin deficiencies, as well as electroneurography tests, which revealed axonal polyneuropathy in all of them, either sensory or motor, or both. In patient 2, cervical MRI showed T2 enhancement at this level indicative of subacute combined spinal cord degeneration. The description of the case series is shown in table 1.

**Figure:**
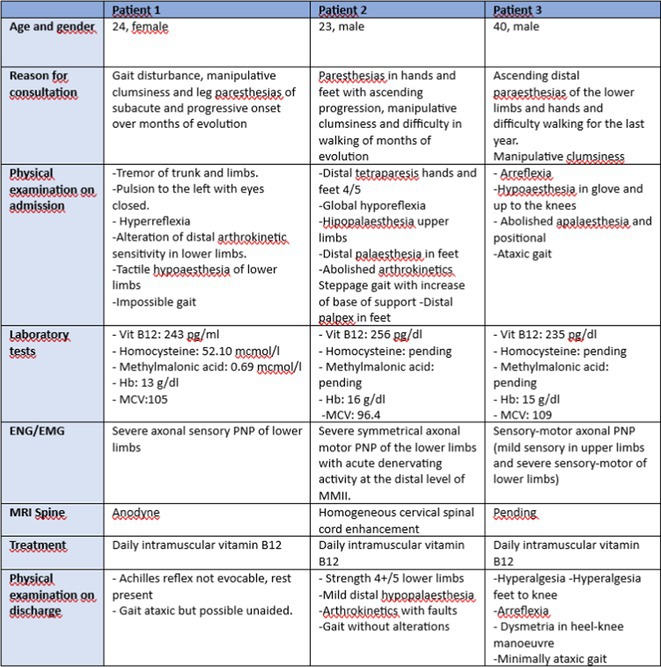
**TABLE 1** Case series description.

**Figure:**
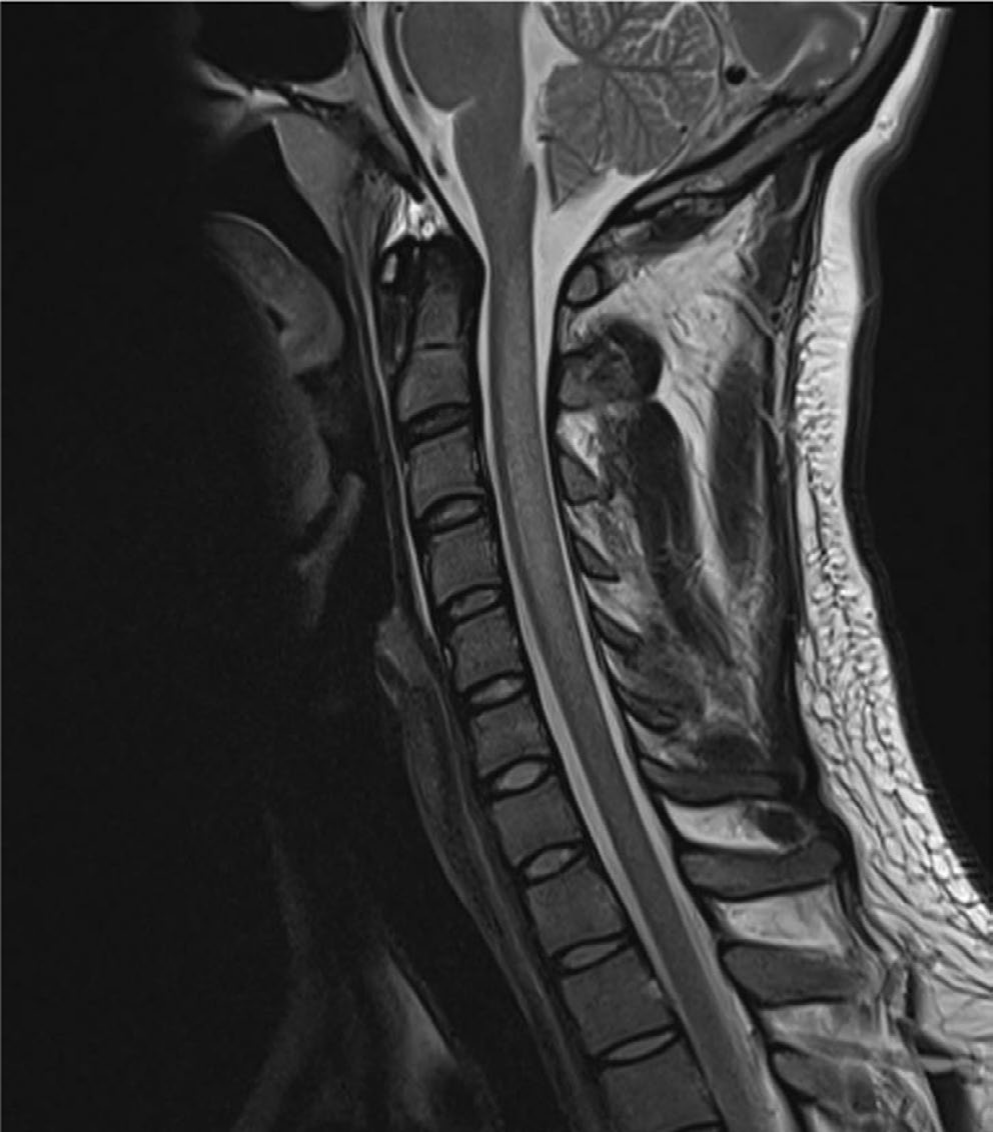
**FIGURE 1** Cervical MRI. T2 hyperintensity is observed at cervical level, corresponding to patient 2.

**Conclusion:** Clinical suspicion is essential in patients with these clinical manifestations as it has been shown that initiation of treatment and cessation of NO2 consumption improves patient recovery. This case report aimed to contribute to the scientific literature for the knowledge and early recognition of this entity.

**Disclosure:** Nothing to disclose.

## EPR‐091

### Minimally invasive resection of first rib for treatment of thoracic outlet syndrome: Technique and outcomes

#### A. Jenkins; C. Harvie; J. O'Donnell; R. Chung

##### Jenkins NeuroSpine, NY, USA

**Background and aims:** Thoracic outlet syndrome (TOS), first described by Peet et al. in 1956,1 refers to symptoms caused by the compression of the neurovascular bundle within the thoracic outlet which extends from the supraclavicular fossa to the axilla between the clavicle and first rib.2 Various approaches to a first rib resection, including the supraclavicular, infraclavicular, and trans‐axillary approach, offer distinct advantages and limitations.3,4 This report presents a minimally invasive infraclavicular approach to a first rib resection that improves visualization and access to the posterior first rib while allowing for neurovascular protection.3,5.

**Methods:** We retrospectively analyzed patient outcomes of TOS patients who received the minimally invasive infraclavicular approach to a first rib resection from 2020 through 2023. We assessed both intraoperative and postoperative metrics including blood loss, complications, fluids, pain, weakness, and numbness.

**Results:** Thirteen patients underwent the minimally invasive first rib resection procedure performed exclusively by the presenting surgeon. Intraoperative results found mean blood loss to be 59.1 cc, 1.3L of crystalloid fluid, and 3 minor complications. Postoperative outcomes showed that numbness improved in 85% (11 of 13 subjects) of patients. VAS neck and arm pain scores decreased 15% and 30%. Additionally, upper extremity weakness improved in 4 of the 5 with preoperative symptoms. A case illustration will be presented to highlight these significant improvements.

**Conclusion:** This novel minimally invasive infraclavicular approach to a first rib resection offers a clear and direct approach to the posterior first rib, enhancing neurovascular protection while also providing the patient with significant relief of their TOS symptoms.

**Disclosure:** Nothing to disclose.

## EPR‐092

### The impact of CIDP on patients’ employment: Results from a real‐world multinational survey

#### S. Paci^1^; C. Arvin‐Berod ^1^; F. Brackx^2^; L. Van de Veire^2^; J. Wright^3^; Y. Taylor^3^; R. Sahar^3^; S. Dewilde^2^

##### ^1^argenx BV, Zwijnaarde (Ghent), Belgium; ^2^Services in Health Economics (SHE), Brussels, Belgium; ^3^Adelphi Real World, Bollington, UK

**Background and aims:** The objective of this secondary analysis is to highlight the impact of Chronic Inflammatory Demyelinating Polyneuropathy (CIDP), an immune‐mediated rare disease that causes increasing weakness and sensory symptoms, on patients’ work situation.

**Methods:** As part Adelphi's CIDP Disease Specific Programme™, a cross‐sectional real‐world survey, matched physician‐ and patient‐reported data were collected in the US, the UK, France, Germany, Italy, Japan and Spain between September 2022 and April 2023. Neurologists submitted demographics through electronic record forms, while some patients voluntarily provided employment data and completed the Work Productivity & Activity Impairment (WPAI) questionnaire.

**Results:** Mean patient age was 54.4 (SD 12.6, *N* = 310), with 59% being male. Many patients were out of full‐time employment: 21% were retired, 10% unemployed, 8% working part‐time, and 6% on long term sick leave. In nearly half these cases, patients indicated CIDP was the reason for their current employment status. Out of 292 patients, 48% reported their disease had impacted their career (past or present): 15% had work responsibilities reduced, 14% stopped working, 12% needed flexible working hours, 11% had/needed unplanned time off work, and 9% experienced a reduction in income. Almost a third (32/118) of patients that were employed either full‐ or part‐time had missed time from work in the last 7 days because of CIDP (mean 6.8 hours, SD 7.0). The mean WPAI percentage for overall work impairment, encompassing absenteeism and presenteeism, was 33.6 (SD 25.4, *n* = 106).

**Conclusion:** CIDP has a substantial impact on patients’ employment, often hindering or completely preventing them from working.

**Disclosure:** SP and CA are employees of argenx and have stock in argenx, the sponsor of the study. JW, YT and RS are employees of Adelphi Real World which received honoraria from argenx for access to data from Adelphi's Disease Specific Programme. FB, LV and SD were commissioned by and received honoraria from argenx.

## EPR‐093

### Baseline characteristics of the first 200 study participants with multifocal motor neuropathy in the iMMersioN study

#### K. Claeys ^1^; C. Hewamadduma^2^; S. Peric^3^; L. Querol^4^; S. Altamimi^5^; I. Van de Walle^6^; E. Persson^6^; I. Van Hoomissen^6^; G. Szmyd^6^; M. Vujcic^6^; S. Cadour^6^; O. Van de Steen^6^; C. Arvin‐Bérod^6^; J. Allen^7^

##### ^1^Department of Neurology, University Hospitals Leuven, Leuven, Belgium; Laboratory for Muscle Diseases and Neuropathies, Department of Neurosciences, KU Leuven, and Leuven Brain Institute (LBI), Leuven, Belgium; ^2^Academic Neuromuscular Unit, Sheffield Teaching Hospitals Foundation NHS Trust, Sheffield, UK; Sheffield Institute for Translational Neuroscience (SITRAN), University of Sheffield, Sheffield, UK; ^3^University of Belgrade, Faculty of Medicine, Neurology Clinic, University Clinical Center of Serbia, Belgrade, Serbia; ^4^Department of Neurology, Neuromuscular Diseases Unit, Hospital de La Santa Creu I Sant Pau, Barcelona, Spain; Centro Para La Investigación Biomédica en Red en Enfermedades Raras (CIBERER), Madrid, Spain; ^5^The Neurology Group, Pomona, CA, USA, ^6^argenx, Ghent, Belgium; ^7^Department of Neurology, University of Minnesota, Minneapolis, MN, USA

**Background and aims:** Multifocal motor neuropathy (MMN) is a rare, peripheral, chronic neuropathy characterized by progressive and disabling asymmetric limb weakness without sensory loss, caused by immune‐mediated complement activation, motor nerve conduction block, and axonal degeneration. The global, prospective, longitudinal iMMersioN study (NCT0598 8073) will characterize disease course, management, and burden of MMN on adult patients with new/existing diagnoses, receiving standard of care treatments. Baseline characteristics of the first 200 participants are reported.

**Methods:** Outcome measures (MMN‐Rasch‐built Overall Disability Score [MMN‐RODS]; modified MRC‐10 [mMRC‐10]) and the impact on health‐related quality of life (Rasch‐Transformed Fatigue Severity Scale [RT‐FSS]; chronic acquired polyneuropathy patient‐reported index [CAP‐PRI]; Patient Global Impression of Severity [PGI‐S]) will be assessed for up to 24 months. Site visits coincide with existing treatment visits (approximately every 3 months), with an optional visit 7–14 days after study start.

**Results:** At enrolment closure, 413 participants have been enrolled. Of the first 200 participants, 76.9% had definite, 11.6% probable, and 11.6% possible MMN. Mean (SD) age was 55.4 (12.8) years; 37% were female, 68% white, and 76.5% European. 46.0% of participants had 3 or 4 limbs affected. Mean (SD) time since initial symptoms and diagnosis were 13.6 (10.0) and 9.6 (8.3) years, respectively. Mean (SD) baseline scores were 74.0 (16.0) for MMN‐RODS centile, 90.7 (10.9) for mMRC‐10, 11.0 (6.5) for RT‐FSS, and 10.7 (6.3) for CAP‐PRI. Most participants (*n* = 80) had a PGI‐S score of 4 (moderate disease).

**Conclusion:** iMMersioN is the first, global study to detail the impact of MMN and MMN treatment on patients in a real‐world setting.

**Disclosure:** KC: Alnylam, Amicus, argenx, Biogen, CSL Behring, Ipsen, Janssen, Lupin, Pfizer, Roche, Sanofi‐Genzyme, Vertex, UCB CH: argenx, Biogen, Lupin, Roche, UCB SP: ADOC, argenx, Berlin‐Chemie Menarini, Kedrion, Mylan, Octapharma, Pfizer, Roche, Salveo, Sanofi Genzyme, Teva Actavis, Wörwag LQ: Annexon, Alnylam, argenx, Avilar Therapeutics, Biogen, CIBERER, CSL Behring, Dianthus, Fundació La Marató, GBS‐CIDP Foundation International, Grifols, Instituto de Salud Carlos III – Ministry of Economy and Innovation (Spain), Janssen, LFB, Lundbeck, Merck, Novartis, Octapharma, Roche, Sanofi, UCB SA: Nothing to disclose IVdW, EP, IVH, MV, OVdS and CA‐B: Employees of argenx GS: Employee of PPD, part of Thermo Fisher Scientific, consultant for argenx SC: Employee of PPD, part of Thermo Fisher Scientific, consultant for argenx JAA: Akcea therapeutics, Alexion, Alnylam, Annexon, argenx, CSL Behring, Grifols, Immunovant, Immupharma, Johnson & Johnson, Pfizer, Takeda

## EPR‐094

### Hereditary neuropathies in Serbian population

#### M. Vukojevic; A. Marjanovic; V. Ivanovic; M. Jankovic; A. Kacar; I. Basta; S. Peric

##### Neurology clinic, University Clinical Center of Serbia, Belgrade, Serbia

**Background and aims:** Hereditary neuropathies encompass a genetically and phenotypically diverse group of disorders. This study aimed to determine final diagnoses in patients referred from a Serbian tertiary referral center under suspicion of hereditary neuropathy.

**Methods:** Patients referred for genetic testing from the Neurology Clinic, University Clinical Center of Serbia (2009–2023) were included. Among 778 suspected cases of hereditary neuropathy, 229 patients were either lost to follow‐up or presented with conditions mimicking neuropathy.

**Results:** Of 549 evaluated patients, 48 (8.7%) had hereditary neuropathy with liability to pressure palsies (HNPP) with PMP22 deletion, and 56 (10.2%) had non‐hereditary compressive neuropathies. Charcot‐Marie‐Tooth disease (CMT) subtype 1A (CMT1A) accounted for 91 (16.6%) cases (3 with point mutations). Other subtypes included X‐linked CMT (CMTX) (2.9%), HINT1 (2.7%), and 37 (6.7%) with rarer CMT forms. The most common distal motor neuropathy (dMN) subtype was HSPB1 (0.55%). Hereditary sensory autonomic neuropathy (HSAN) was identified in 2 cases. Polyneuropathy associated with other genetic syndromes was found in 29 (5.2%) patients, while 3.0% had inconclusive WES findings and 6.4% lacked mutations. Acquired neuropathy was diagnosed in 157 (28.6%) patients.

**Conclusion:** In line with other populations, CMT1A was the most common cause of hereditary neuropathy in Serbia. A notable presence of CMTX and HINT1 neuropathy is specific for Serbian population. These findings highlight the importance of targeted genetic analysis in diagnosing hereditary neuropathies in certain population.

**Disclosure:** Nothing to disclose.

## EPR‐095

### Healthcare resource utilization and caregiver burden in CIDP: Results from a real‐world multinational survey

#### S. Paci ^1^; C. Arvin‐Berod^1^; F. Brackx^2^; L. Van de Veire^2^; J. Wright^3^; Y. Taylor^3^; R. Sahar^3^; S. Dewilde^2^

##### ^1^argenx BV, Zwijnaarde (Ghent), Belgium; ^2^Services in Health Economics (SHE), Brussels, Belgium; ^3^Adelphi Real World, Bollington, UK

**Background and aims:** Chronic Inflammatory Demyelinating Polyneuropathy (CIDP) is a rare, immune‐mediated disease that causes progressive weakness and sensory symptoms. This analysis highlights medical and non‐medical resources required by CIDP patients.

**Methods:** CIDP‐treating neurologists completed surveys for Adelphi's Disease Specific Programme™ (September 2022‐April 2023), capturing demographics, healthcare resource utilization, caregiver support, and mobility aids usage across the UK, the US, France, Germany, Italy, Japan and Spain.

**Results:** Mean patient age was 54.8 (SD 12.6, *N* = 936) years; 63% were male and 47% used mobility aids, usually a cane/walking stick (36%). The mean number of healthcare professional types involved in disease management was 2.4 (SD 1.4); patients most often saw a neurologist (81%), followed by a physical therapist (41%) and family doctor/GP (38%). Patients attended a mean 7.8 (SD 13.4) consultations in the last 12 months (*n* = 914). Among 750 patients, 14% were hospitalized in the last 12 months; for their most recent hospitalization, 55% were admitted through the ER, and 9% spent time in the ICU. Out of 840 patients, 3% received professional caregiver support, while 25% relied on informal caregivers, usually a partner/spouse (84% out of 219 patients). Patients with informal caregiver(s) (*n* = 211) received a mean 28.7 (SD 33.3) hours of care per week. Consequently, caregivers (*n* = 157) reduced working hours (22%), stopped working (11%) or worked from home (10%). Participation in activities like social events (50%), physical activity/exercise (33%) and going on holiday (29%) was reduced or avoided altogether.

**Conclusion:** CIDP patients require many resources, including mobility aids, hospitalizations and assistance from healthcare professionals and caregivers.

**Disclosure:** SP and CA are employees of argenx and have stock in argenx, the sponsor of the study. JW, YT and RS are employees of Adelphi Real World which received honoraria from argenx for access to data from Adelphi's Disease Specific Programme. FB, LV and SD were commissioned by and received honoraria from argenx.

## EPR‐096

### CIDP patients’ journey to diagnosis: Results from a real‐world multinational survey

#### S. Paci ^1^; C. Arvin‐Berod^1^; F. Brackx^2^; L. Van de Veire^2^; J. Wright^3^; Y. Taylor^3^; R. Sahar^3^; S. Dewilde^2^

##### ^1^argenx, Zwijnaarde (Ghent), Belgium; ^2^Services in Health Economics (SHE), Brussels, Belgium; ^3^Adelphi Real World, Bollington, UK

**Background and aims:** This secondary analysis aimed to characterize the diagnostic journey of patients suffering from Chronic Inflammatory Demyelinating Polyneuropathy (CIDP), a rare and severe immune‐mediated disease associated with progressive limb weakness and sensory symptoms.

**Methods:** Adelphi's CIDP Disease Specific Programme™ collected point‐in‐time data through surveys of CIDP‐treating neurologists between September 2022 and April 2023. This real‐world, multinational study captured demographics and journey to diagnosis data for CIDP patients in the US, the UK, France, Germany, Italy, Japan and Spain.

**Results:** In the total sample (*N* = 936), 63% of patients were male, with the mean age being 54.8 (SD 12.6) years. The mean period of time between patients’ (*n* = 660) symptom onset and first consultation with a healthcare professional (*n* = 903), usually a general neurologist (56%) or family doctor/GP/PCP (27%), was about 7.0 (SD 15.7) months. Another 6.0 (SD 15.0) months would typically pass before patients were diagnosed with CIDP, predominantly by a general neurologist (76% of 934 patients). The mean number of diagnostic tests undergone by patients was 19.2 (SD 10.1). Neurological examination (98%), a review of the patient's medical history (88%) and an electromyogram (EMG) (85%) were most often used to aid diagnosis. Out of 740 patients, 34% were initially misdiagnosed with one or more condition(s). Patients were most frequently misdiagnosed with Guillain–Barré syndrome (33%), diabetic polyneuropathy (15%), and fibromyalgia (12%).

**Conclusion:** Patients suffering from CIDP experience a substantial burden securing their diagnosis. The journey to diagnosis is, on average, longer than a year, and over a third were initially misdiagnosed.

**Disclosure:** SP and CA are employees of argenx and have stock in argenx, the sponsor of the study. JW, YT, and RS are employees of Adelphi Real World which received honoraria from argenx for access to data from Adelphi's Disease Specific Programme. FB, LV and SD were commissioned by and received honoraria from argenx.

## EPR‐097

### A comparison of treatment satisfaction in CIDP across 5 countries: Results from a real‐world multinational survey

#### S. Paci ^1^; C. Arvin‐Berod^1^; F. Brackx^2^; L. Van de Veire^2^; J. Wright^3^; Y. Taylor^3^; R. Sahar^3^; S. Dewilde^2^

##### ^1^argenx BV, Zwijnaarde (Ghent), Belgium; ^2^Services in Health Economics (SHE), Brussels, Belgium; ^3^Adelphi Real World, Bollington, UK

**Background and aims:** Chronic Inflammatory Demyelinating Polyneuropathy (CIDP) is an immune‐mediated disorder affecting sensory function and strength. This study compares patient and physician satisfaction with treatment across five countries.

**Methods:** Point‐in‐time data were collected in the UK, France, Germany, Italy, and Spain via physician and patient surveys as part of Adelphi's CIDP Disease Specific Programme™ (September 2022‐April 2023). This real‐world study captured demographics, as well as treatment history and satisfaction data. Treatment satisfaction data were not collected for UK patients.

**Results:** In all countries, patients (*n* = 542) were predominantly male; mean (SD) age ranged between 51.9 (10.9) and 56.6 (13.8). At time of survey, the majority of patients were receiving treatment: the UK (*n* = 54) reported the largest proportion (94%), while Spain (*n* = 120) reported the lowest (80%). Despite treatment, around half of patients in most countries experienced moderate‐to‐severe symptoms; exceptions were Spain (38%) and the UK (78%). The proportion of patients less than satisfied with overall treatment were 21% (Germany, *n* = 81), 33% (Spain, *n* = 27), 35% (Italy, *n* = 26), and 45% (France, *n* = 18). Inversely, 4% (Italy), 10% (Germany), 11% (France), and 25% (Spain) of patients were very satisfied with treatment. For treatment efficacy and treatment convenience, Spanish patients again reported the highest rates of satisfaction, and German patients the lowest rates of dissatisfaction. Physicians of these patients were more positive: 33% (France, *n* = 18), 42% (Italy, *n* = 26), 48% (Spain, *n* = 27), and 58% (Germany, *n* = 81) were very satisfied with overall treatment.

**Conclusion:** Across countries, CIDP patients were less satisfied with treatment than their physicians; around half reported moderate‐to‐severe symptoms despite treatment.

**Disclosure:** SP and CA are employees of argenx and have stock in argenx, the sponsor of the study. JW, YT and RS are employees of Adelphi Real World which received honoraria from argenx for access to data from Adelphi's Disease Specific Programme. FB, LV and SD were commissioned by and received honoraria from argenx.

## EPR‐098

### Autonomic nervous system impairment in patients with chronic demyelinating polyneuropathies

#### T. Todorovic ^1^; I. Bozovic^2^; B. Bijelica^3^; A. Palibrk^2^; S. Peric^2^; I. Basta^1^

##### ^1^Faculty of Medicine, University of Belgrade, Serbia; ^2^Neurology Clinic, University Clinical Centre of Serbia; ^3^Neurology Clinic, Hannover Medical School, Germany

**Background and aims:** In chronic demyelinating polyneuropathies more attention is often paid to motor and sensory functions, and autonomic functions are usually overlooked. The aim of our study was to examine autonomic dysfunction in patients with different types of chronic demyelinating polyneuropathies, acquired and hereditary.

**Methods:** Following diagnoses were included: chronic inflammatory demyelinating polyneuropathy (CIDP, *n* = 98), polyneuropathy associated with monoclonal gammopathy of undetermined significance (MGUS‐PN, *n* = 51), Charcot‐Marie‐Tooth disease type 1A (CMT1A, *n* = 51), hereditary neuropathy with liability to pressure palsies (HNPP, *n* = 18) in comparison to healthy controls (HCs, *n* = 125), SCOPA‐AUT questionnaire was used, comprising questions related to the function of the cardiovascular, gastrointestinal and urinary systems, as well as thermoregulation and sexual functions.

**Results:** Mean SCOPA‐AUT score was 13.6 ± 11.1 in CIDP, 18.4 ± 8.4 in MGUS‐PN, 14.1 ± 9.5 in CMT1A, 11.7 ± 8.6 in HNPP versus 7.4 ± 7.3 in HCs. All patient groups, except for HNPP, differed in comparison to HCs (*p* < 0.01). Gastrointestinal, urinary and cardiovascular scores were worse in MGUS‐PN compared to HCs (3.3 ± 3.6 vs. 1.4 ± 1.7, 4.5 ± 3.0 vs. 2.3 ± 2.6 and 1.1 ± 1.4 vs. 0.5 ± 1.0, respectively, *p* < 0.05). Thermoregulation was worse in all patient groups compared to HCs (*p* < 0.05). Male sexual dysfunction was noticed in CIDP and MGUS‐PN, and female sexual dysfunction in CIDP, MGUS‐PN and CMT1A.

**Conclusion:** Among patients with chronic demyelinating polyneuropathies, autonomic dysfunction was most pronounced in patients with MGUS‐PN. Patients with HNPP presented the least autonomic impairment.

**Disclosure:** Nothing to disclose.

## Sleep‐wake disorders

## EPR‐099

### Obstructive sleep apnea in amyotrophic lateral sclerosis: Diagnostic challenges and predictive modeling

#### D. Bottignole ^1^; V. Malanchini^1^; A. Nuredini^1^; G. Balella^1^; F. Rausa^2^; L. Zinno^1^; M. Maggio^3^; L. Parrino^3^; C. Mutti^3^

##### ^1^Neurology Unit, Department of Medicine and Surgery, University of Parma, Parma, Italy; ^2^Sleep Disorders Center, Department of General and Specialized Medicine, University Hospital of Parma, Parma, Italy; ^3^Mario Giovanni Terzano Interdepartmental Center for Sleep Medicine, Department of Medicine and Surgery, University of Parma, Parma, Italy

**Background and aims:** Amyotrophic lateral sclerosis (ALS) is a neurodegenerative disorder characterized by motor and non‐motor symptoms, including sleep disturbances. Despite their clinical significance, sleep‐related issues remain underexplored in ALS clinical assessments. We aimed to investigate sleep disorders' prevalence, characteristics, and impact in ALS patients.

**Methods:** We conducted a prospective study enrolling ALS patients followed by the Neuromuscular Disorders Service of the University Hospital of Parma, Italy. Disease severity, cognitive performances, sleep quality, daytime sleepiness, and quality of life were specifically assessed. Full‐night cardiorespiratory monitoring (CRM) was performed to diagnose sleep‐related breathing disorders. Correlations between sleep disturbances, ALS severity, and patient outcomes were explored. Logistic regression was used to develop a predictive model for OSA.

**Results:** Among 22 ALS patients (70.57  ± 10.8 years), 86.4% reported sleep disturbances, including leg cramps (55%), insomnia (20%), and restless leg syndrome (15%). 57.1% of patients were affected by OSA: male patients were more frequently affected by OSA (72.7% vs. 40%) and generally presented more severe manifestations (*p* = 0.010). A novel predictive score incorporating BMI, PSQI, sex, and bulbar involvement showed 87.5% specificity and 80% accuracy for OSA detection (Fig. 1). Elevated apnea indices correlated with reduced 12‐month survival (67% vs. 100% for lower indices) (Fig. 2).

**Figure:**
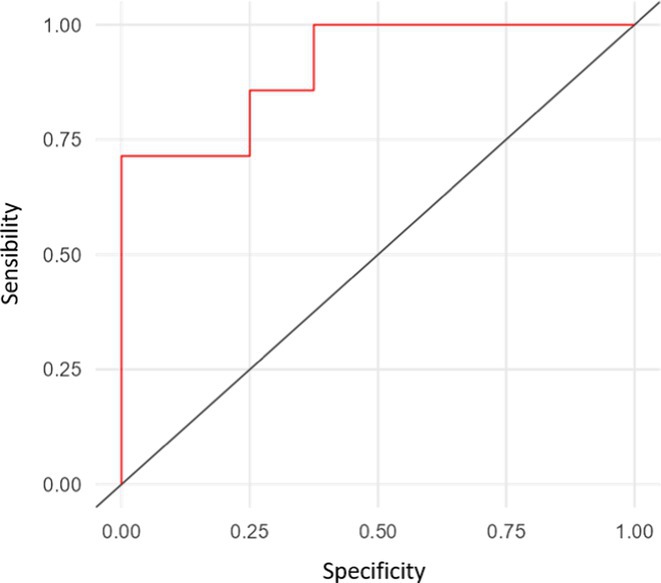
**FIGURE 1** ROC curve representing the reliability in predicting the presence of OSA in the study population using a model with four variables: Body Mass Index, Pittsburgh Sleep Quality Index total score, gender, and presence of pseudobulbar symptoms.

**Figure:**
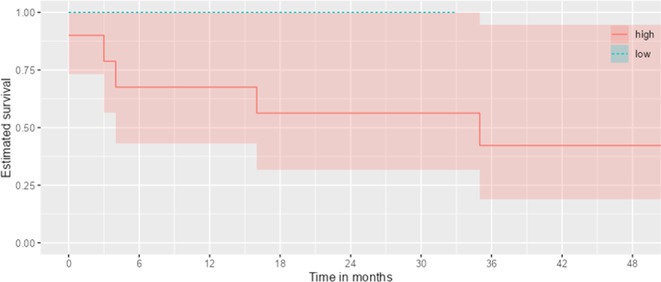
**FIGURE 2** Graphical representation (Kaplan–Meier method) of the estimated survival in relation to AHI values in the sample. The red line represents patients with an AHI > 3.8 events/h, the blue dotted line represents those with an AHI.

**Conclusion:** Our findings reveal a high prevalence of sleep disorders in ALS patients, emphasizing the limitations of standard OSA screening tools. The OSAPS model demonstrated superior accuracy for OSA prediction in this population. Comprehensive sleep assessments may improve patient management and outcomes. Further multicenter studies are warranted to validate these findings.

**Disclosure:** Each Author has no relevant financial or non‐financial interests to disclose. All authors have reviewed and approved the manuscript for submission and affirm that the work was conducted independently without external influence.

## EPR‐100

### The burden of insomnia and excessive daytime sleepiness: Switzerland's pilot study preliminary results

#### M. Tüzün ^1^; U. Kallweit^2^; S. Seidel^3^; O. Endrich^4^; S. Trelle^5^; M. Leone^6^; O. Bruni^7^; R. Dodel^8^; A. Fiorillo^9^; I. Holmerová^10^; J. Jaarsma^11^; M. Lolich^12^; M. Konti^12^; D. Ramankulov^12^; D. Pevernagie^13^; E. Pupillo^6^; W. Randerath^14^; L. Vignatelli^15^; C. Meyer‐Massetti^16^; M. Schmidt^1^; C. Bassetti^17^

##### ^1^Interdisciplinary Sleep‐Wake‐Epilepsy‐Center, Bern University Hospital (Inselspital) and University of Bern, Bern, Switzerland; ^2^University Witten/Herdecke, Faculty of Medicine, Professorship for Narcolepsy and Hypersomnolence Research, Witten, Germany; ^3^Rehabilitation Clinic Pirawarth, Bad Pirawarth, Austria; ^4^University Institute of Clinical Chemistry, University of Bern, Switzerland; ^5^Medical Directorate, Inselspital, Bern University Hospital, Switzerland; ^6^Department of Neurosciences, Istituto di Ricerche Farmacologiche “Mario Negri” IRCCS, Milano, Italy; ^7^Department of Developmental and Social Psychology, Sapienza University, Rome, Italy; ^8^Department of Geriatric Medicine, University Duisburg‐Essen, Essen, Germany; ^9^Department of Psychiatry, University of Campania “L. Vanvitelli”, Naples, Italy, ^10^Centre of Expertise in Longevity and Long‐term Care, Charles University, Prague, Czechia, ^11^European Alliance for Restless Legs Syndrome, Brussels, Belgium, ^12^European Academy of Neurology, Vienna, Austria, ^13^Department of Respiratory Diseases and Sleep Disorders Centre, AZ Delta, Roeselare, Belgium, ^14^Clinic for Pneumology and Allergology, Center of Sleep Medicine and Respiratory Care, Hospital Bethanien, Solingen, Germany, ^15^IRCCS Istituto delle Scienze Neurologiche di Bologna, Bologna, Italy, ^16^Clinical Pharmacology & Toxicology, Department of General Internal Medicine, Inselspital ‐ University Hospital Bern, Switzerland, ^17^Sleep‐Wake Epilepsy Center, NeuroTec, Department of Neurology, Inselspital, Bern University Hospital, University of Bern, Bern, Switzerland

**Background and aims:** Excessive daytime sleepiness (EDS) and insomnia (IN) significantly impact individuals and society, yet their specific needs and broader socioeconomic effects remain underexplored. To address this gap, a pilot trial for a multi‐stage, European‐wide, multicenter research study commenced in Switzerland in mid‐2023.

**Methods:** This prospective, national cohort observational study aimed to evaluate the burden and progression of EDS and IN over 12‐month post‐initial assessment. Recruitment is conducted by nine primary care providers, aiming for completion by June 2024. The primary goal is to assess study feasibility, with secondary goals of determining EDS/IN prevalence in primary care and their correlation with health‐related quality of life (QoL) using validated tools. Patients screened positive for EDS/IN are invited to join the online study, featuring standardized questionnaires.

**Results:** Of 632 screened subjects, 238 (44%) reported subjective EDS/IN, 135 expressed interest, and 92 participated in the online segment (64% female, mean age 47.4 years, BMI 24.8 kg/m^2^). Of these, 38% had EDS (ESS > 10) and 70% had IN symptoms (ISI > 7), though only 4.9% were diagnosed with a sleep/wake disorder at baseline. Over half reported distress in social, occupational, and educational areas. QoL assessment with SF‐12 showed reduced scores (normalized score: 0.66), primarily due to the mental component (0.57).

**Conclusion:** Excessive daytime sleepiness (EDS) and insomnia (IN) symptoms are highly prevalent in primary care settings and apparently underdiagnosed. Our findings highlight a significant decline in quality of life (QoL) among patients with these conditions.

**Disclosure:** This study was supported by the European Academy of Neurology.

## EPR‐101

### Discriminative value of psychomotor vigilance and sustained attention to response tests in identifying hypersomnolence

#### K. Zub^1^; J. van der Meer^1^; E. Wenz^1^; L. Fregolente^1^; J. Warncke^1^; O. Gnarra^2^; R. Morand^3^; A. Helmy^1^; R. Khatami^4^; S. von Manitius^5^; S. Miano^6^; M. Strub^7^; A. Datta^8^; S. Bürki^8^; U. Kallweit^9^; D. Bijlenga^10^; G. Lammers^10^; M. Tüzün ^1^; M. Schmidt^1^; C. Bassetti^1^

##### ^1^Sleep‐Wake Epilepsy Center, NeuroTec, Department of Neurology, Inselspital, Bern University Hospital, University of Bern, Bern, Switzerland; ^2^Sensory‐Motor System Lab, Department of Health Sciences and Technology, Institute of Robotics and Intelligent Systems, ETH Zurich, Switzerland; ^3^ARTORG Center for Biomedical Engineering Research, University of Bern, Bern, Switzerland; ^4^Clinic Barmelweid, Center for Sleep Medicine and Sleep Research, Barmelweid, Switzerland; ^5^Department of Neurology, Kantonsspital St. Gallen St, Gallen, Switzerland; ^6^Sleep and Epilepsy Center, Neurocenter of Southern Switzerland, Regional Hospital (EOC) of Lugano, Lugano, Switzerland; ^7^Centre for Sleep Medicine Basel, Basel, Switzerland; ^8^Neuropaediatrics, University Children's Hospital Basel, Basel, Switzerland; ^9^Center for Narcolepsy and Hypersomnias, Professorship for Narcolepsy and Hypersomnolence Research, Department of Medicine, University Witten/Herdecke, Witten, Germany, ^10^Sleep‐Wake Center, Stichting Epilepsie Instellingen Nederland (SEIN), ¨Heemstede, The Netherlands

**Background and aims:** Attention deficits are key symptoms of central disorders of hypersomnolence (CDH), such as narcolepsy. Sustained Attention to Response Test (SART) and Psychomotor Vigilance Test (PVT) are common tools to assess attention deficits, but their discriminative value for CDH remains unclear. This study aimed to identify the most discriminative outcome measures of SART and PVT and calculate their cut‐off values to distinguish CDH from healthy controls (HC).

**Methods:** This study is a subproject of the international Swiss Primary Hypersomnolence and Narcolepsy Cohort Study (iSPHYNCS). Participants (CDH patients and HC) completed SART (3 sessions: 11am, 1pm, 5 pm) and PVT (1 session: 3pm). Reaction time (RT), standard deviation of RT (SD), lapses (both tests), commission errors (SART), and fastest/slowest 10% of RT (F10 and S10, respectively) for PVT) were analyzed. ROC and PCA analyses were performed, and cut‐off values calculated.

**Results:** SART (CDH, *n* = 132; HC, *n* = 31) showed higher SD and lapses in patients (*p* < 0.05). For PVT (CDH, *n* = 119; HC, *n* = 31), all outcomes except F10 were worse in patients (*p* < 0.005). ROC analysis showed SART's lapses and SD achieved AUCs of 0.66 and 0.65, respectively, with cut‐offs of 0 lapses and 67 ms for SD of RT. PVT's SD and S10 yielded higher AUCs of 0.79 and 0.76, with cut‐offs of 47 ms and 410 ms, respectively. PCA‐based combination of parameters did not enhance accuracy.

**Figure:**
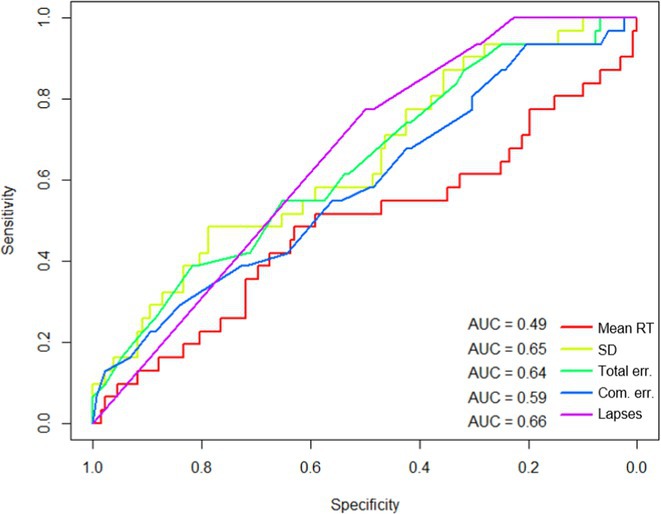
**FIGURE 1** ROC curves for SART outcomes distinguishing CDH patients from HC. “Com. err.” denotes commission errors, and “Total err.” represents the sum of commission errors and lapses.

**Figure:**
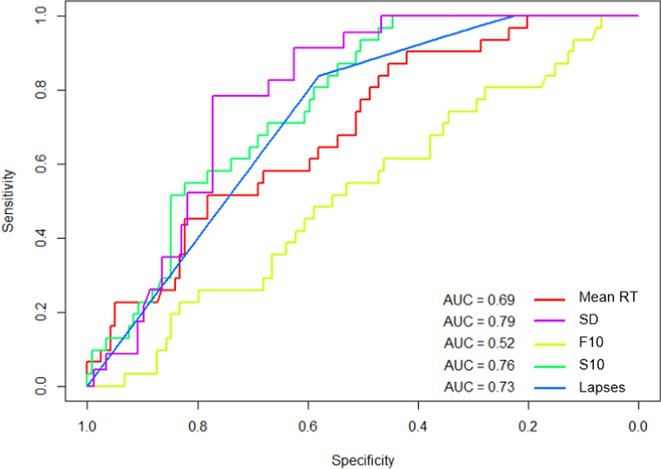
**FIGURE 2** ROC curves for the PVT outcomes to distinguish CDH patients from HC.

**Conclusion:** The PVT appears more accurate than the SART for identifying attention deficits in CDH. Reliable cut‐offs ensure proper test interpretation and support SART and PVT use for treatment monitoring.

**Disclosure:** The authors declare no conflict of interest. The study is supported by the Swiss National Science Foundation (SNF 320030_185362; SNF 32003B_215721).

## EPR‐102

### Glucagon‐like 1 analogs and sleep‐related eating disorder—A new hope?

#### M. de Oliveira Carvalho ^1^; A. Aldomiro^2^; S. Parreira^1^; A. Franco^1^; C. Bentes^1^; A. Peralta^1^

##### ^1^Neurology Department, Unidade Local de Saúde Santa Maria, Lisbon, Portugal; ^2^Neurology Department, Unidade Local de Saúde da Arrábida, Setúbal, Portugal

**Background and aims:** Sleep‐related eating disorder (SRED) is a syndrome characterized by periods of involuntary eating after arousal during sleep, with partial or complete loss of awareness. The current therapeutic options have limited efficacy. Glucagon‐like peptid‐1 (GLP‐1) analogs might be a possible treatment.

**Methods:** We report 3 cases of patients with SRED resolution after introducing GLP‐1 analogs.

**Results:** The first case is a 44‐year‐old woman who seeks medical attention due to initial insomnia and sleep fragmentation due to unaware arousals to consume high‐calorie and “bizarre” foods, causing a 40 kg weight gain in 20 years. SSRIs, topiramate, and zonisamide were tried without efficacy. After beginning liraglutide, complete and sustained remission was observed. The second case is a 47‐year‐old man evaluated for multiple night‐eating arousals since age 18. He was given ropinirole, topiramate and APAP, with partial improvement. SRED subsided with the introduction of semaglutide. The last case is a 56‐year‐old woman with SRED associated with zolpidem abuse. The patient reduced her zolpidem intake but still had SRED episodes. After starting semaglutide, complete control of symptoms was observed.

**Conclusion:** The effects of GLP‐1 analogs on appetite suppression have been extensively studied and may be due to effects on the digestive tract or central nervous system (such as on the infralimbic cortex). These have shown promise in treating addictions, such as smoking or drug use. These are the first reported SRED cases improved by GLP‐1 analogs, which may present a new hope for the treatment of this treatment‐resistant condition.

**Disclosure:** Nothing to disclose.

## EPR‐103

### Incidence and burden of narcolepsy in France using the Système National des Données de Santé (SNDS)

#### S. Crawford ^1^; S. Ghosh^1^; B. Podmore^2^; A. Azpeitia^2^; Y. Dauvilliers^3^

##### ^1^Takeda Development Center Americas, Inc., Cambridge, MA, USA; ^2^OXON Epidemiology, Madrid, Spain; ^3^Sleep‐Wake Disorders Center, Department of Neurology, Gui‐de‐Chauliac Hospital, CHU, Montpellier, France

**Background and aims:** Narcolepsy is a rare, chronic, central nervous system disorder of hypersomnolence characterized by excessive daytime sleepiness. The study objective was to estimate the incidence and burden of narcolepsy in France.

**Methods:** Retrospective population‐based cohort study using anonymized healthcare data among 66 million people from the SNDS database (2014‐2019). Incident narcolepsy cases (INCS) were matched to non‐narcolepsy controls.

**Results:** 1,650 INCS were identified, with annual incidence rates varying from 0.45 to 0.55 per 100,000 person years. INCS were young (50% < 24.5 years) and predominantly female (54.7%). Other sleep disorders (e.g., sleep apnea; 26.8% vs. 0.71%), psychiatric (e.g., depression, anxiety; 8.2% vs. 3.5%) and nervous system (e.g., migraine, epilepsy; 8.3% vs. 1.6%) comorbidities were more frequent in INCS versus controls. The frequency of outpatient and inpatient visits were significantly higher for INCS than controls, including general practitioners (incidence rate ratio [IRR] [95% CI] 1.77 [1.74‐1.79]) any specialists (2.71 [2.67‐2.75]), neurologists (29.96 [27.91‐32.16]), psychiatrists (5.23 [5.07‐5.40]), and all‐cause in‐patient admissions (3.87 [3.76‐3.98]). Approximately 80% of INCS received narcolepsy medication within 7 months of diagnosis: 73.0% first received modafinil monotherapy. Other treatments included: methylphenidate 32.4%, venlafaxine 19.5%, and pitolisant 17.6% at any time after the index date (date of narcolepsy diagnosis). Most patients (93%) started on monotherapy and up to 47.6% subsequently received combination therapy. Overall medication persistence of narcolepsy‐specific medications for INCS was 19‐28% at 6 months and 8‐15% at 12 months.

**Conclusion:** This study provides a robust estimation of narcolepsy incidence and presents evidence for high healthcare burden compared with controls.

**Disclosure:** SC and SG are employees of Takeda Development Center Americas, Inc. BP and AA are employees of OXON Epidemiology, who were contracted by Takeda for this work. YD received funds for seminars, board engagements and travel to congresses from Avadel, Bioprojet, Idorsia, Jazz, Orexia, and Takeda. Takeda Development Center Americas, Inc., provided funding to Excel Medical Affairs for support in writing this abstract.

## EPR‐104

### The relationship between glymphatic function and disease duration in isolated rapid eye movement sleep behavior disorder

#### V. Rottova ^1^; S. Marecek^1^; T. Krajca^2^; K. Sonka^1^; J. Keller^3^; P. Dusek^1^; J. Nepozitek^1^

##### ^1^Department of Neurology and Center of Clinical Neuroscience, First Faculty of Medicine, Charles University and General University Hospital, Prague, Czechia; ^2^Faculty of Biomedical Engineering, Czech Technical University in Prague, Kladno, Czechia; ^3^Radiodiagnostic Department, Na Homolce Hospital, Prague, Czechia

**Background and aims:** Isolated rapid eye movement sleep behavior disorder (iRBD) is commonly recognized as a prodromal stage of alpha‐synucleinopathies, such as Parkinson's disease, multiple system atrophy, and dementia with Lewy bodies. Dysfunction of the glymphatic system was observed in these neurodegenerative disorders using diffusion tensor imaging along the perivascular space (DTI‐ALPS) method. This study aimed to evaluate the association between the ALPS‐index and the duration of iRBD.

**Methods:** In this study, we included 51 patients (67.00  ± 7.0 years [mean age  ± SD]) with an iRBD diagnosis confirmed by polysomnography. The patients reported the onset of their dream enactment symptoms during interview. Glymphatic function was evaluated with the DTI‐ALPS method by obtaining diffusion parameters near the top of the lateral ventricles and calculating age‐adjusted ALPS‐indices. We performed a correlation analysis between the ALPS‐index and the duration of iRBD since the onset of the first symptoms.

**Results:** We identified a statistically significant positive correlation between the ALPS‐index and the duration of iRBD dream enactment (Spearman's rho = 0.357, *p* = 0.010). Regression analysis revealed statistical significance (*p* = 0.005), with the summary model showing R = 0.387, R^2 = 0.150.

**Conclusion:** Preserved function of the glymphatic system was associated with longer duration of iRBD. These findings suggest that the glymphatic system may serve as a protective factor preserving the prodromal stage of the disease and can potentially be used as a biomarker for delayed conversion into fully pronounced neurodegenerative disease.

**Disclosure:** Nothing to disclose.

## EPR‐105

### Sleep‐disordered breathing is associated with adverse short‐ and long‐term outcomes after acute ischemic stroke

#### X. Yang ^1^; J. Lippert^1^; S. Bauer‐Gambelli^1^; C. Horvath^2^; S. Baillieul^3^; T. Reichlin^4^; A. Brill^2^; C. Bernasconi^1^; M. Schmidt^1^; D. Seiffge^1^; M. Arnold^1^; C. Bassetti^1^

##### ^1^Department of Neurology, Inselspital, Bern University Hospital, University of Bern, Switzerland; ^2^Department of Pulmonary Medicine, Allergology and Clinical Immunology, Inselspital, Bern; ^3^Grenoble Alpes University, HP2‐Inserm U1300, CHU Grenoble Alpes, Grenoble, France; ^4^Department of Cardiology, Inselspital, Bern University Hospital, University of Bern, Switzerland

**Background and aims:** Sleep‐disordered breathing (SDB) is common after stroke and is associated with poor outcomes, but data from large cohort studies are sparse.

**Methods:** This retrospective cohort study, from the Bern Sleep – Stroke Registry, was conducted at the Inselspital Bern. Acute ischemic stroke/TIA patients underwent respiratory polygraphy within 72h of admission. SDB severity was categorized based on apnea‐hypopnea index (AHI), with thresholds defining mild (AHI≥5h), moderate (AHI≥15h), and severe (AHI≥30/h) cases. Evaluated outcomes including poor functional outcome (mRS≥3 at 3‐month), all‐cause mortality, and MACE over an 84‐month follow‐up period. Cox and logistic regression models, adjusted for age, sex, BMI, hypertension, diabetes, dyslipidemia, atrial fibrillation, and initial stroke severity (NIHSS), were employed.

**Results:** We included 1083 patients, (678 (63%) males, mean age 66.0  ± 14.5y, 982 (91%) ischemic stroke), 29), of whom 819 (76%) had SDB (AHI≥5/h), sub‐categorized as mild 323 (29.8%), moderate 248 (23%), and severe 248 (23%). Central sleep apnea was observed in 6.4%. The initial mean NIHSS was 4.4  ± 5.5. All‐cause mortality was associated with severe SDB (adjusted HR, 2.74 [95%CI, 1.19‐6.34]). MACE were significantly associated with mild (adjusted HR, 1.47 [95%CI, 1.06‐2.04]), moderate (adjusted HR, 2.01 [95%CI, 1.43‐2.82]) and severe SDB (adjusted HR, 1.53 [95%CI, 1.07‐2.18]). Poor functional outcome was associated with severe SDB (adjusted OR, 2.50 [95%, 1.45‐4.38]).

**Conclusion:** Results reveal a significant association between SDB and adverse short‐ and long‐term stroke outcomes, with the impact varying by SDB severity. Further investigation is needed to refine stroke risk profiles and optimize post‐stroke management strategies.

**Disclosure:** None

## EPR‐106

### The Bern Sleep–Wake Registry (BSWR): From raw polysomnographic data to clinical outcomes

#### J. van der Meer^1^; E. Villar Ortega^1^; V. Kälin^1^; A. Helmy^1^; U. Nwachukwu^1^; M. Pesce^1^; A. Tzovara^2^; E. Wenz^1^; L. Fregolente^1^; K. Zub^1^; M. Wulf^1^; A. Dietmann^1^; X. Yang ^3^; S. Carolin^1^; M. Schmidt^1^; C. Bassetti^1^

##### ^1^Sleep‐Wake Epilepsy Center, Department of Neurology, Inselspital, University Hospital Bern, University of Bern, Bern, Switzerland.; ^2^Institute of Computer Science, University of Bern, Bern, Switzerland.; ^3^Department of Pulmonary Medicine, Allergology and Clinical Immunology, Inselspital, Bern University Hospital, University of Bern, Bern 3010, Switzerland

**Background and aims:** The combined availability of clinical outcomes and polysomnography (PSG) raw data offers novel data driven approaches toward patient characterization and the identification of new digital biomarkers. With the Bern Sleep – Wake Registry (BSWR), we aimed to provide a continuously growing respective data collection.

**Methods:** Data including clinical records, electrophysiological measures and questionnaire scores from patients assessed at the sleep–wake center of the University Hospital Bern since 2000 are continuously transferred into a REDCap database. Median (IQR) values are reported.

**Figure:**
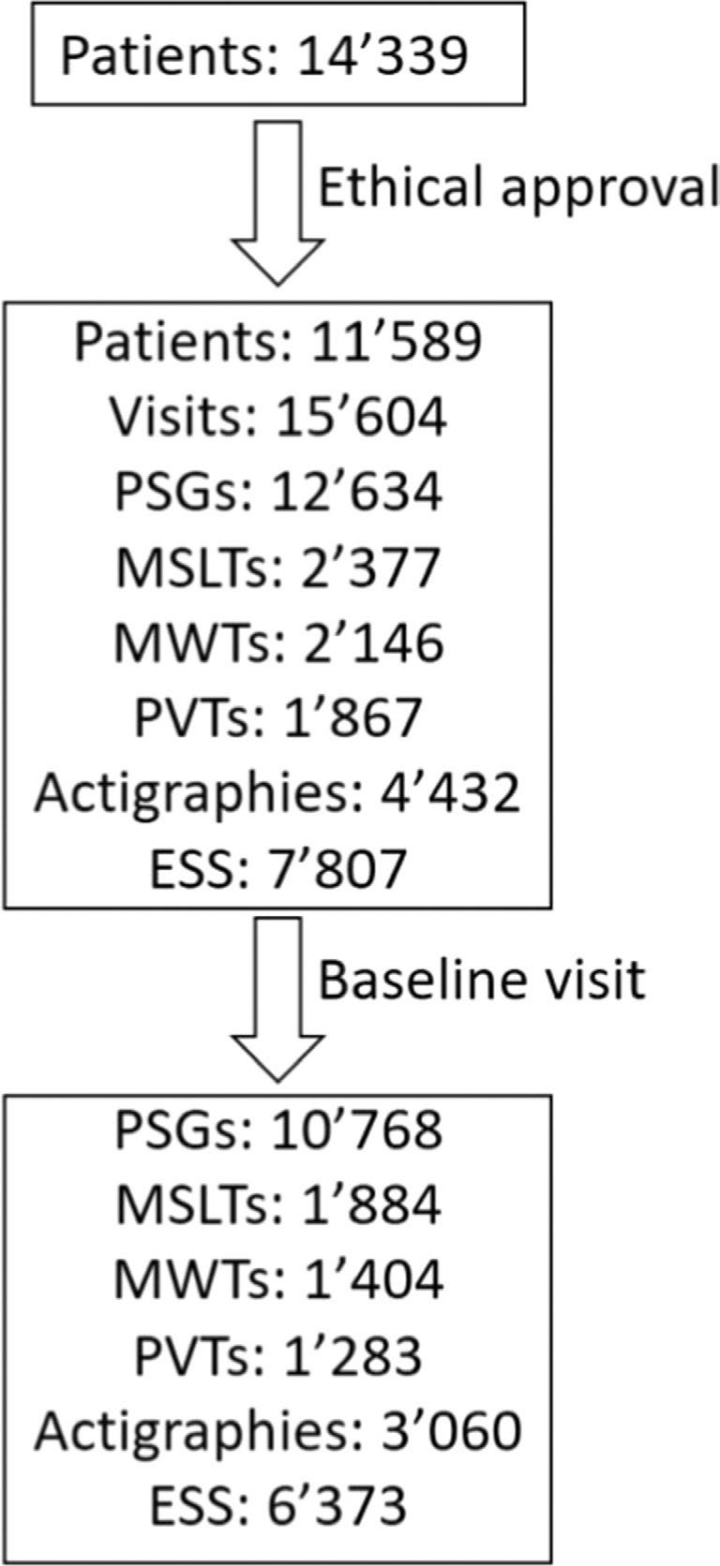
**FIGURE 1** Flowchart on availability of multi‐modal data.

**Results:** A total of 11,589 patients from the BSWR were included for scientific evaluation according to general consent based ethical approval (KEK‐ 2022‐00415). At first consultation, age was 50 (36, 61) years, with male over‐representation (65%), BMI was 26 (23, 30), Epworth sleepiness scale was 10 (6, 14), PSG‐derived sleep efficiency was 88% (77, 94), AHI was 12 (4, 28). Moderate to severe sleep apnea and periodic limb movements (AHI / PLMI > 15), were prevalent (44% and 23%, respectively). The most common diagnoses included sleep disordered breathing (62%), central disorders of hypersomnolence (12%) and insomnia (7%). We found marked differences between disorders, gender and age across multiple parameters in the multi‐modal data set, often with interdependent effects (e.g., an age‐dependent enhancement of the Epworth sleepiness scale in women as compared to men). From 2,162 patients, follow‐up visits are available.

**Figure:**
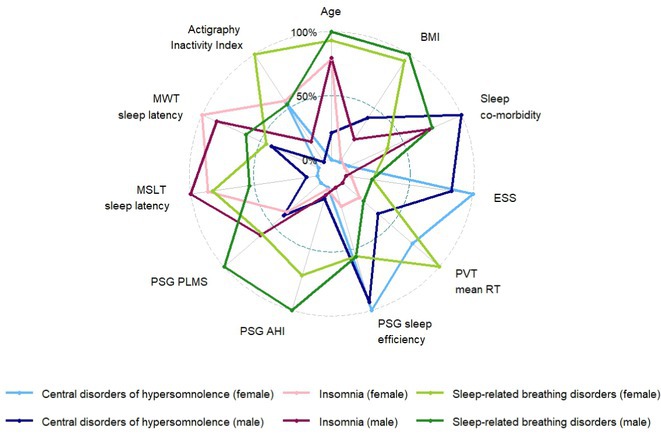
**FIGURE 2** Spider‐plot of multi‐modal data for most prevalent primary sleep disorders (baseline visit).

**Figure:**
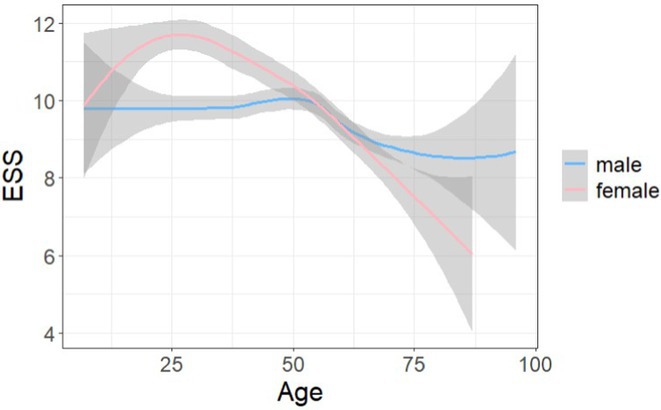
**FIGURE 3** Age‐dependent daytime sleepiness peak in women (baseline visit).

**Conclusion:** The BSWR is to date the largest patient registry covering the full sleep disorder spectrum, providing an invaluable source for validating digital biomarkers and diagnostic criteria.

**Disclosure:** The authors declare no conflicts of interest. This work was supported by the Inselspital MB‐Neuro Grant “A digital reference network platform for clinical and experimental neuroscience—deep phenotyping and data integration”.

## EPR‐107

### Effect of orexin agonist on wakefulness in NT1 during MWT and baseline comparison of microsleeps between NT1 and NT2

#### Y. Gong; B. Tracey; A. Cai; T. Olsson; D. Volfson

##### Takeda Development Center Americas, Inc., Cambridge, MA, USA

**Background and aims:** The Maintenance of Wakefulness Test (MWT) assesses excessive daytime sleepiness (EDS) in clinical trials, primarily using mean sleep latency (SL) as the endpoint. However, SL does not capture quality of wakefulness (QoW) before sleep. Microsleeps—brief sleep episodes (3‐15 seconds) occurring before sleep onset—may serve as a sensitive biomarker for EDS. This study compares baseline microsleep features between NT1 and NT2 participants in Phase 2 randomized trials NCT05687903 and NCT05687916. Additionally, we examined microsleep changes in NT1 patients receiving orexin receptor 2‐selective agonist TAK‐861 in NCT05687903.

**Methods:** Baseline MWT, ESS, and microsleep features were compared between 112 NT1 and 71 NT2 participants. In NCT05687903, NT1 participants received TAK‐861 (placebo: *n* = 22; 0.5mg/0.5mg: *n* = 23; 2mg/2mg: *n* = 21; 2mg/5mg: *n* = 23; 7mg: *n* = 23). Four 40‐minute MWT sessions were conducted at baseline and after 28 and 56 days of treatment. Microsleeps were manually scored from MWT data. Changes in microsleep rates and time‐to‐first microsleep were assessed using mixed‐effect models.

**Results:** At baseline, NT1 participants had significantly (*p* < 0.05) shorter mean SL (4.72 ± 6.46 vs. 9.75 ± 8.95 minutes), higher ESS scores (18.51 ± 2.99 vs. 17.14 ± 3.59), and higher microsleep rate (8.01 ± 7.26 vs. 2.82 ± 4.09) than NT2 participants. In NT1 participants, TAK‐861 reduced microsleep rates from > 6 (baseline) to < 2 (day 56) minutes per 10 minutes and delayed time‐to‐first microsleep for all treatment arms. Placebo‐treated NT1 participants showed minimal changes.

**Conclusion:** At baseline, QoW, measured by microsleep features, differed between NT1 and NT2 participants. TAK‐861 significantly reduced microsleep rates and delayed microsleep onset in participants with NT1. Future analyses will explore microsleep dynamics in NT2 participants.

**Disclosure:** All authors are employees of Takeda Development Center Americas, Inc. Takeda Development Center Americas, Inc., provided funding to Excel Medical Affairs for support in writing this abstract.

## Sunday, June 22, 2025

## Ageing and Dementia 2

## EPR‐108

### A cost‐efficient tool to screen patients for anti‐amyloid immunotherapies in memory clinics: The FRAPI score

#### A. Zilioli ^1^; B. Pancaldi^1^; G. Busi^1^; F. Misirocchi^1^; L. Corradi^1^; G. Messa^3^; L. Ruffini^1^; R. Mohanty^2^; E. Westman^2^; M. Spallazzi^1^

##### ^1^University‐Hospital of Parma; ^2^Division of Clinical Geriatrics; Center for Alzheimer Research; Department of Neurobiology, Care Sciences and Society, Karolinska Institutet, Huddinge, Stockholm, Sweden; ^3^AUSL Parma, Italy

**Background and aims:** Determining amyloid status is crucial for identifying patients eligible for anti‐amyloid therapies. This study aimed to develop a cost‐effective predictive tool that integrates neuropsychological tests and brain atrophy scales to assess amyloid status in a cohort potentially eligible for anti‐amyloid treatment.

**Methods:** We retrospectively analyzed 101 consecutive patients from the Dementia Unit at the University Hospital of Parma. Participants met the FDA eligibility criteria for Lecanemab and underwent amyloid‐PET assessment for suspected AD, complete cognitive tests, and MRI/CT scans. Binomial logistic regression evaluated the predictive accuracy of cognitive tests and visual atrophy scales individually and in combination.

**Results:** The Free and Cue Selective Reminding Test (FCSRT) Immediate Free Recall (IFR) subtest demonstrated the highest predictive accuracy (AUC = 0.68; 95% CI = 0.56–0.80), followed by the Recall of Rey's figure (AUC = 0.66; 95% CI = 0.54–0.78). Among visual rating scales, the antero‐posterior index (API) was the most accurate (AUC = 0.68; 95% CI = 0.59–0.80). Combining these measures into the FRAPI score (FCSRT, IFR ≤17 = 2 points; Recall of Rey's Figure: ≤13 = 1 point; API: ≥1 = 2 points) yielded the highest predictive accuracy (AUC = 0.77; 95% CI = 0.67–0.87), with notable performance in patients with > 8 years of education (AUC = 0.96; 95% CI = 0.91–1).

**Figure:**
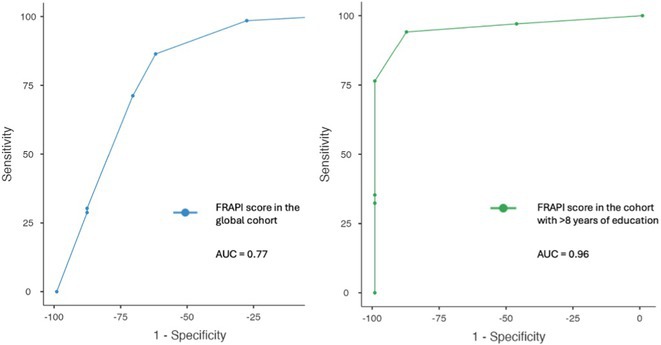
**FIGURE 1** ROC curves of the FRAPI (F: FCSRT; R: Recall of Rey's figure; API: Antero‐posterior index) score in the global cohort and among patients with education > 8 years.

**Figure:**
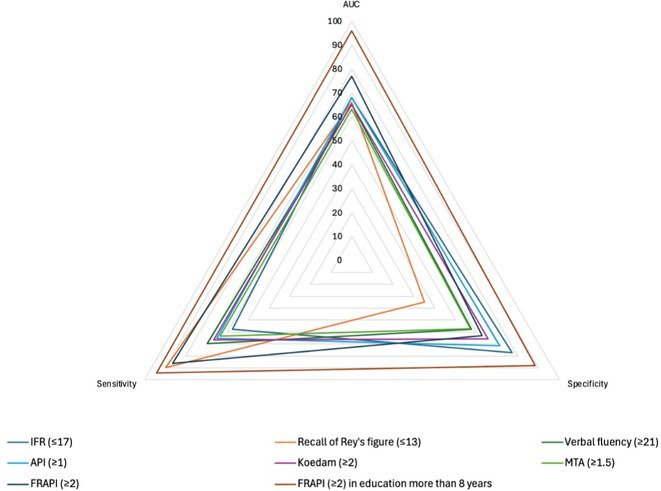
**FIGURE 2** Predictive performance of the main neuropsychological, visual rating scores, and the novel FRAPI (F: FCSRT; R: Recall of Rey's figure; API: Antero‐posterior index) score. The cut‐offs displayed are those with the highest Youden index.

**Figure:**
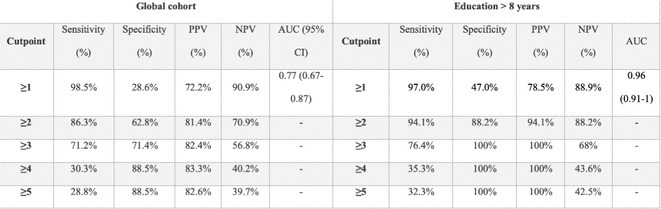
**TABLE 1** Predictive performance of the FRAPI (F: FCSRT; R: Recall of Rey's figure; API: Antero‐posterior index) score in the global cohort and in patients with education > 8 years.

**Conclusion:** The FRAPI score is a practical, cost‐effective tool for screening patients eligible for anti‐amyloid treatment, especially those with higher education, to obtain biological confirmation of AD in primary and secondary memory clinics.

**Disclosure:** Nothing to disclose.

## EPR‐109

### Water‐soluble QTY variant of the gamma‐secretase complex provides insights into amyloid precursor protein recognition

#### A. Karagöl

##### Istanbul Faculty of Medicine, Istanbul University, Istanbul, Turkey

**Background and aims:** The Amyloid Precursor Protein (APP), when processed by gamma‐secretase complex, is cleaved into various peptides, including the Amyloid Beta 42 fragments. These fragments are prone to oligomerization, and the resulting oligomers subsequently self‐assemble into amyloid plaques. This process is believed to initiate a cascade of events involved in Alzheimer's disease. Thus, targeting APP recognition could be a viable therapeutic and diagnostic approach.

**Methods:** We engineered a water‐soluble presenilin‐1 subunit of the gamma‐secretase complex utilizing the QTY‐code. QTY‐code is a method to generate water‐soluble variants of membrane proteins without the use of detergents. The APP fragment (PDB ID: 6IYC) complexes with the QTY‐variant were generated using AlphaFold3. All‐atom molecular dynamics simulations and binding free energy calculations were conducted to elucidate the interaction patterns with APP.

**Results:** Our findings reveal that the water‐soluble QTY‐variant of presenilin‐1 subunit demonstrates structural resemblance to presenilin‐1 (Figure 1). The QTY‐variant demonstrated water‐solubility (Figure 2), with its hydrophilic surface. Subsequently, simulations in aqueous solvent indicate that the system stabilizes after 30ns, with the MMGBSA binding free energy around ‐200 kcal/mol (Figure 3). The conserved binding interfaces suggest that APP is effectively captured by the water‐soluble variant, with key residues contributing to the interaction being uncovered.

**Figure:**
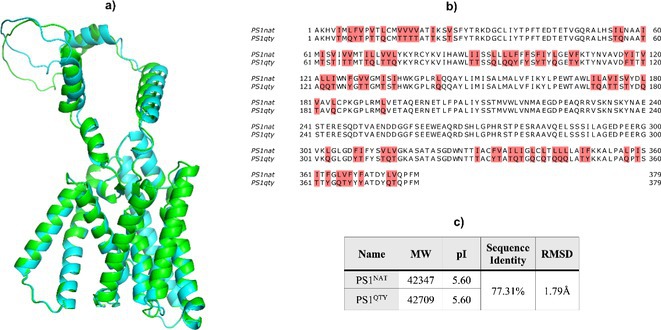
**FIGURE 1** Bioinformatics analysis of the QTY variant of presenilin‐1. a) Superposed structures of the QTY‐variant (cyan) and presenilin‐1 (green). Sequence alignment (b) with variations highlighted, and their sequence characteristics (c).

**Figure:**
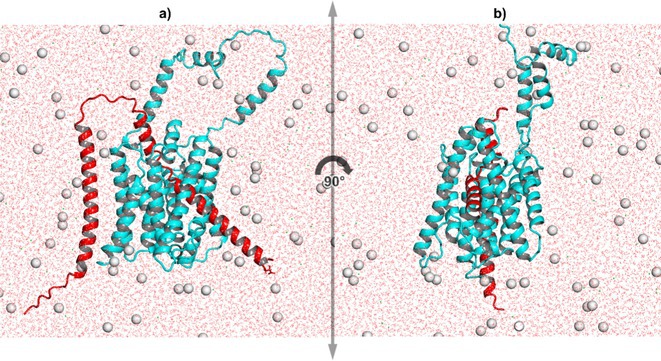
**FIGURE 2** The water‐soluble QTY‐variant of presenilin‐1 (cyan) complexed with APP fragment (red). The equilibrated complex is in solution with Monte‐Carlo placed K+ CL− ions (neutralizing, concentration = 0.15 M).

**Figure:**
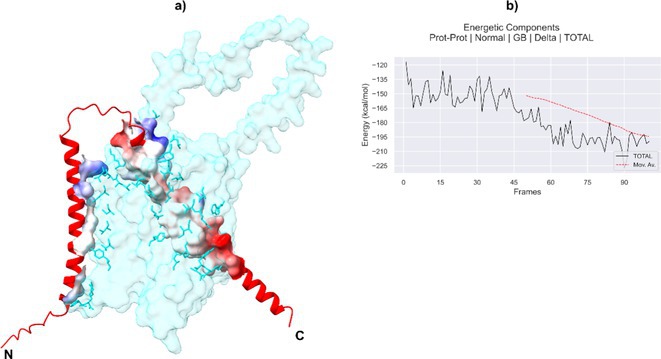
**FIGURE 3** Binding interface of the APP fragment and QTY‐variant complex. a) The QTY‐variant (cyan) and the APP (red) with the contributing residues mapped. b) MMGBSA binding energy calculations of the complex through 50ns equilibrated simulation.

**Conclusion:** These insights into APP binding reveal another aspect of the underlying interaction mechanism. Our findings establish a framework for studying the early molecular development of Alzheimer's disease. The water‐soluble QTY‐variant can be utilized as a diagnostic tool and for efficiently analyzing drug targets in an aqueous environment.

**Disclosure:** Nothing to disclose.

## EPR‐110

### Impact of the COVID‐19 pandemic on dementia diagnoses in Sweden: Trends from 2015 to 2023

#### B. Winblad ^1^; E. Aho^1^; S. Aye^1^; L. Jönsson^1^; S. Schedin‐Weiss^1^; L. Tjernberg^1^; A. Wimo^1^; X. Xia^1^; R. Ziyue Zhou^1^; . Axenhus^2^

##### ^1^Division of Neurogeriatrics, Department of Neurobiology, Care Sciences and Society, Karolinska Institutet, Stockholm, Sweden; ^2^Karolinska Institutet, Department of Clinical Sciences at Danderyd Hospital, Sweden

**Background and aims:** The COVID‐19 pandemic has influenced the diagnosis and management of dementia. This study explores trends in dementia diagnoses across Sweden from 2015 to 2023, examining regional, gender, and diagnostic differences. The analysis focuses on the pandemic's impact (2020–2022) and the post‐pandemic period (2023).

**Methods:** This is a retrospective cohort study. Age standardized diagnosis rates were collected from specialized in‐ and outpatient care obtained from the National Patient Registry. Diagnoses were vascular dementia, Alzheimer's disease, unspecified dementia, and other dementias. Diagnosis rates were averaged across pre‐pandemic (2015–2019), pandemic (2020–2022), and post‐pandemic (2023) periods. Logistic regression modeling was used to determine expected outcome without pandemic influence which was compared to actual outcome. Student t‐test was used to determine significant differences.

**Results:** Vascular dementia showed a 31.9% reduction (*p* < 0.01), unspecified dementia declined by 25.5% (*p* < 0.01), and Alzheimer's disease fell by 11.1% (*p* < 0.01). Post‐pandemic (2023) trends varied: Alzheimer's disease exhibited partial recovery, increasing by 6.0% from pandemic levels (*p* < 0.05), vascular and unspecified dementia rates declined by 2.8% (*p* < 0.05) and 3.4% (*p* < 0.05), respectively. Across all diagnoses, 2023 rates remained below pre‐pandemic levels by ‐33.8%.

**Conclusion:** The COVID‐19 pandemic significantly disrupted dementia diagnoses in Sweden. Vascular dementia appears more vulnerable than other diagnoses groups. Post‐pandemic recovery has been uneven, with some diagnoses, such as Alzheimer's disease, showing signs of improvement, while others continue to decline. Possible explanations could include higher diagnostic burden in primary care and less resources for diagnosis of complex dementia disorders.

**Disclosure:** No disclosures.

## EPR‐111

### Apraxic deficits predict general cognitive impairment in patients with biomarker‐verified Alzheimer's pathology

#### C. Schmidt ^1^; M. Bardakan^1^; E. Jaeger^2^; N. Richter^3^; G. Bischof^2^; K. Giehl^2^; O. Onur^3^; F. Jessen^4^; G. Fink^3^; A. Drzezga^2^; P. Weiss^1^

##### ^1^Cognitive Neuroscience, Institute of Neurosciences and Medicine (INM‐3), Forschungszentrum Jülich, Jülich, Germany; ^2^Department of Nuclear Medicine, Faculty of Medicine and University Hospital Cologne, University of Cologne, Cologne, Germany; ^3^Department of Neurology, Faculty of Medicine and University Hospital Cologne, University of Cologne, Cologne, Germany; ^4^Department of Psychiatry and Psychotherapy, Faculty of Medicine and University Hospital Cologne, University of Cologne, Cologne, Germany

**Background and aims:** Apraxia represents a core feature of Alzheimer's disease (AD), a neurodegenerative disorder characterized by the accumulation of β‐amyloid plaques and tau deposition. However, systematic descriptions of apraxic deficits in AD patients remain scarce. Here, we comprehensively investigate apraxia profiles and their link with cognitive impairment in patients with biomarker‐verified Alzheimer's pathology.

**Methods:** We characterized the frequency and patterns of apraxic deficits in patients with biomarker‐verified Alzheimer's pathology using a battery of standardized apraxia tests. Demographic variables and apraxia scores were related to patients’ general cognitive impairment using hierarchical regression analysis.

**Results:** Apraxic deficits were found in 68% of patients with biomarker‐verified Alzheimer's pathology (*n* = 66). AD patients were more impaired in imitating finger gestures (than hand gestures: 89.0% vs. 78.9%, *p* < 0.001) and imitating complex hand movements (than single hand movements: 97.4% vs. 77.9%, *p* < 0.001), even when controlling for general cognitive impairment. Apraxia assessments explained about 60% of the variance in dementia severity, with performance in the KAS subtest of pantomiming object use (beta coefficient: 0.44, *p* = 0.001) and the DATE subtest for limb apraxia (beta coefficient: 0.38, *p* = 0.002) constituting significant predictors of general cognitive impairment.

**Figure:**
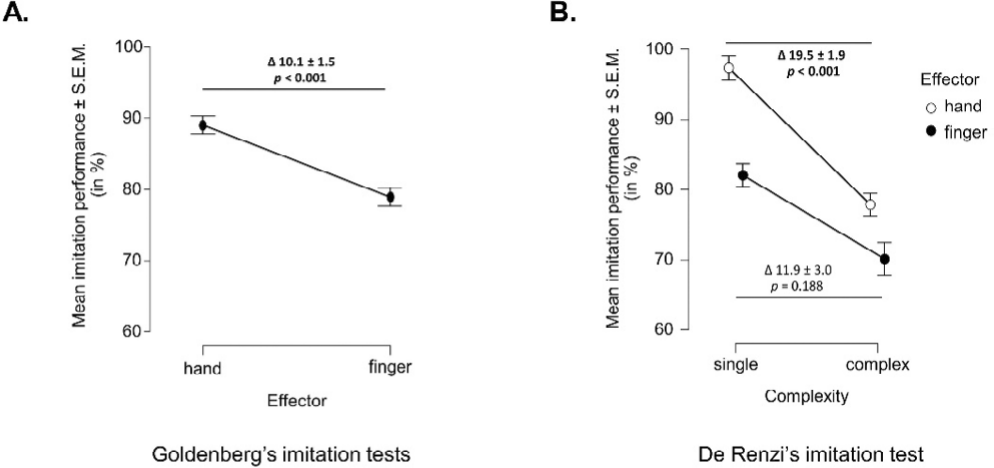
**FIGURE 1** Differential patterns of apraxic imitation deficits in the patients with biomarker‐verified Alzheimer's pathology

**Conclusion:** These findings emphasise the relevance of apraxia in patients with biomarker‐verified Alzheimer's pathology, revealing that praxis deficits predict general cognitive impairment in AD. Further research is warranted into the role of apraxia as a potential early diagnostic criterion in AD.

**Disclosure:** Nothing to disclose.

## EPR‐112

### Photophobia discriminates between Alzheimer's disease and dementia with Lewy bodies patients

#### F. Millet Barros ^1^; J. Durães^1^; M. Tábuas Pereira^1^; P. Faustino^1^; C. Bernardes^1^; R. Pancas^2^; M. Lima^3^; I. Baldeiras^4^; C. Cavadas^5^; I. Santana^1^

##### ^1^Neurology Department, Unidade Local de Saúde de Coimbra, Coimbra, Portugal; ^2^Internal Medicine Department, Unidade Local de Saúde de Coimbra, Coimbra, Portugal; ^3^Center for Research in Neuropsychology and Cognitive Behavioral Intervention, Faculty of Psychology and Educational Sciences, Universidade de Coimbra, Coimbra, Portugal; ^4^Faculty of Medicine, Universidade de Coimbra, Coimbra, Portugal; ^5^Center for Neuroscience and Cell Biology (CNC), Universidade de Coimbra, Coimbra, Portugal

**Background and aims:** Visual photosensitivity has recently been suggested as a common and possibly specific symptom in dementia with Lewy bodies (DLB), including in prodromal phases. It is, however, not an easily quantifiable symptom, as there are currently no validated scales for assessing photophobia in DLB. In this study, we aimed to assess the ability of the Visual Light Sensitivity Questionnaire‐8 (VLSQ‐8) to discriminate between DLB and Alzheimer's Disease (AD) patients.

**Methods:** We included 94 dementia patients ‐ 52 with DLB and 42 with AD ‐ diagnosed according to the most recent international criteria, who underwent CSF‐AD biomarker analysis, as well as cognitive (MMSE), dysautonomia (SCOPA‐AUT), cognitive fluctuations (DCFS‐R), hallucinations (NEVHI), and photophobia (VLSQ‐8) evaluations.

**Results:** Mean VLSQ8 was significantly higher in DLB patients (12.3 ± 5.9) than in AD (9.6 ± 3.1). VLSQ8 was correlated with dysautonomia (measured by SCOPA‐AUT, r = 0.38, *p* < 0.001), the presence of simple hallucinations (measured by NEVHI, r = 0.24, *p* = 0.020) and fluctuations (measured by DCFS‐R, r = 0.24, *p* = 0.019). In a binary logistic regression adjusted for age, age of onset, sex, education and MMSE, VLSQ8 was associated with the diagnosis of DLB (OR: 1.172, 95%CI = [1.048, 1.311], *p* = 0.005).

**Conclusion:** In our cohort of dementia patients, a quantitative assessment for photophobia using the VLSQ8 was able to discriminate between AD and DLB patients. It correlated with dysautonomia, fluctuations and visual hallucinations, suggesting common underlying mechanisms.

**Disclosure:** None.

## EPR‐113

### Dysautonomia and fluctuations in dementia with Lewy bodies

#### M. Coelho ^1^; J. Durães^1^; M. Tábuas‐Pereira^1^; C. Bernardes^1^; P. Faustino^1^; F. Millet‐Barros^1^; M. Ferreira^2^; M. Lima^3^; C. Cavadas^4^; I. Baldeiras^5^; I. Santana^1^

##### ^1^Neurology Department, Unidade Local de Saúde de Coimbra, Coimbra, Portugal; ^2^Neurology Department, Unidade Local de Saúde de Matosinhos, Porto, Portugal; ^3^Center for Research in Neuropsychology and Cognitive Behavioral Intervention, Faculty of Psychology and Educational Sciences, Universidade de Coimbra, Coimbra, Portugal; ^4^Center for Neuroscience and Cell Biology (CNC), Universidade de Coimbra, Coimbra, Portugal; ^5^Neurochemistry Laboratory, Center for Neuroscience and Cell Biology, Coimbra, Portugal

**Background and aims:** Cognitive fluctuations are a core clinical finding in Dementia with Lewy Bodies (DLB), yet their underlying causes remain poorly understood. Our aim was to evaluate whether dysautonomia is associated with cognitive fluctuations in DLB.

**Methods:** We included DLB patients with mild dementia from our memory clinic. Diagnosis was made according to the most widely accepted criteria and supported by extensive characterization, including DaTScan, Amyloid‐PET and/or CSF biomarkers. Cognitive fluctuations were assessed with the Mayo Fluctuations Scale (MFS) and dysautonomia with the SCOPA‐AUT questionnaire. Motor function was assessed with the Movement Disorder Society‐Unified Parkinson's Disease Rating Scale, Part III (MDS‐UPDRS III) and behavioral symptoms with the Neuropsychiatric Inventory (NPI). Patients with mild dementia (CDR = 1) were selected to warrant a homogeneous cohort with the core symptoms. Univariate and multivariable analyses were conducted to assess the relationship between cognitive fluctuations (MFS) and other variables.

**Results:** Our cohort included 61 patients, 37 of which (60,7%) were male. The mean age was 76.3( ± 5.5) years and the mean of disease duration was 4.1 ( ± 2.4) years. Average MMSE was 21.8( ± 3.8), mean MFS was 2.2( ± 1.2), mean MDS‐UPDRS III was 30.6( ± 23.7), mean NPI was 19.7( ± 13.7) and mean SCOPA‐AUT was 16.4( ± 9.0). On univariate analysis, MFS correlated with SCOPA‐AUT (r = 0.27, *p* = 0.038) and NPI (r = 0.48, *p* < 0.001). On multivariable analysis (table 1), MFS was only associated with SCOPA‐AUT (β = 0.039, 95%CI = [0.004, 0.074], *p* = 0.029).

**Conclusion:** Our study suggests an association between dysautonomia and cognitive fluctuations in DLB, highlighting potential novel therapeutic targets. Understanding this relationship may uncover new pharmacological and non‐pharmacological treatments to better manage DLB.

**Disclosure:** Nothing to disclose.

## EPR‐114

### Natural language processing distinguishes Italian individuals with nonfluent/agrammatic from logopenic variants of PPA

#### P. Battista ^1^; S. Aresta^1^; P. Santacesaria^1^; A. Benzini^1^; C. Palmirotta^1^; S. Tagliente^1^; R. Capozzo^2^; A. Introna^3^; S. Tagliente^4^; C. Lunetta^5^; C. Salvatore^6^; A. Benussi^7^

##### ^1^Laboratory of Neuropsychology, Clinical and Scientific Institutes Maugeri IRCCS, Bari, Italy; ^2^Neurology and Stroke Unit, Di Venere Hospital, Bari, Italy; ^3^Department of Translational Biomedicine and Neuroscience, University of Bari, Bari, Italy; ^4^Department of Neurology, “F. Miulli” General Hospital, Acquaviva delle Fonti, Italy; ^5^Neurorehabilitation Unit, Clinical and Scientific Institutes Maugeri IRCCS, Milan, Italy; ^6^Department of Science, Technology and Society, University School for Advanced Studies IUSS Pavia, Italy; ^7^Neurology Unit, Department of Medicine, Surgery and Health Sciences, University of Trieste, Trieste, Italy

**Background and aims:** Differentiating between logopenic (lvPPA) and nonfluent/agrammatic (nfvPPA) variants of Primary Progressive Aphasia relies on expert evaluations of speech and language production. An effortful production of sentences (including morphosyntactic and phonological errors) can be present in both variants for different reasons. Connected speech analysis greatly supports the phenotypical classification of PPA, targeting abnormal language production.

**Methods:** 19 Italian patients with PPA (nfvPPA = 9; lvPPA = 10) underwent an audio‐recorded picture description task from the SAND battery. The speech data were analyzed using computational methods with the CLAN software, allowing the extraction of linguistic features. Using a Mann‐Whitney non‐parametric test corrected for false discovery rate (FDR), we analyzed 45 linguistic features of four linguistic levels. A machine‐learning (ML) model was trained to classify nfvPPA versus lvPPA.

**Results:** We identified ten features belonging to 4 linguistic levels differentiating nfvPPA from lvPPA. These included, at the phonetic and phonological level: silent pause ratio; at the lexico‐semantic level: noun, adverb, article, and determiner ratios; at the morphosyntactic level: total number of utterances and utterance error ratio; and at the pragmatic/discourse level: total words, total morphemes, and idea density. The ML model reached a sensitivity and specificity > 90%.

**Conclusion:** We implemented natural language processing to perform a machine‐learning classification based on connected speech samples of Italian‐speaking subjects. This approach has been applied for the first time to Italian PPA and demonstrated that key linguistic markers can be identified and compared across the two variants.

**Disclosure:** Nothing to disclose.

## EPR‐115

### AI‐based staging, causal hypothesis and progression of subjects at risk of Alzheimer's disease: A multicenter study

#### S. Aresta ^1^; R. Nemni^2^; M. Zanardo^3^; G. Sirabian^2^; D. Capelli^2^; M. Alì^2^; P. Vitali^3^; E. Bertoldo^4^; V. Fiolo^4^; L. Bonanno^5^; G. Maresca^5^; P. Battista^6^; F. Sardanelli^3^; F. Pizzini^7^; I. Castiglioni^8^; C. Salvatore^9^

##### ^1^Department of Science, Technology and Society, University School for Advanced Studies IUSS Pavia, Pavia, Italy; ^2^Centro Diagnostico Italiano S.p.A., Milan, Italy; ^3^Unit of Radiology, IRCCS Policlinico San Donato, San Donato Milanese, Italy; ^4^Clinical Psychology Service, IRCCS Policlinico San Donato, San Donato Milanese, Italy; ^5^IRCCS Centro Neurolesi Bonino Pulejo, Messina, Italy; ^6^Laboratory of Neuropsychology, Istituti Clinici Scientifici Maugeri IRCCS, Institute of Bari, Bari, Italy; ^7^Department of Engineering for Innovation Medicine, University of Verona, Verona, Italy; ^8^Department of Physics “G. Occhialini”, Università degli Studi di Milano‐Bicocca, Milan, Italy; ^9^Deeptrace Technologies S.R.L., Milan, Italy

**Background and aims:** In 2024, eleven European scientific societies/organizations and one patient advocacy association have defined a biomarker‐based diagnostic workflow evaluating neurocognitive disorders. This study evaluated the clinical performance of an AI‐tool using neuropsychological assessment and neuroimaging for staging, diagnosis, and progression prediction in individuals at risk of Alzheimer's disease (AD).

**Methods:** This multicentric study enrolled 796 subjects: 705 from ADNI and 91 from three Italian centers. Participants were staged as healthy (HS), subjective cognitive impairment (SCI), mild cognitive impairment (MCI), or AD‐dementia at baseline and 24‐month follow‐up. Patients were clinically profiled based on cognitive and neuroimaging findings, with first‐line biomarkers measured. The AI‐tool TRACE4AD™ (DeepTrace Technologies S.R.L.) analyzed neuroimaging and neuropsychological data to perform staging, clinical profiling, and predicting AD‐dementia conversion. AI‐human staging agreement was assessed using Cohen's kappa, and AI performance was evaluated by sensitivity, specificity, positive predictive value (PPV), negative predictive value (NPV), area under curve (AUC), and accuracy.

**Results:** For the staging classification the inter‐rater AI‐humans agreement was substantial for both HS/SCI versus rest (Cohen's κ = 0.81) and MCI (κ = 0.70) classification, almost perfect for MSD versus rest (κ = 0.90) classification. For the causal hypothesis classification, the AI performance versus biomarker‐based diagnosis was: PPV 91%, NPV 100%, and accuracy 91%. For the binary classification of progression to AD‐dementia at 24‐month, the AI performance was: sensitivity 89%, specificity 82%, accuracy 85%, and AUC 83%.

**Conclusion:** The AI‐tool demonstrated its usefulness in supporting the clinical treatment of AD patients by assisting with staging, clinical profiling, and progression.

**Disclosure:** S.A., R.N., M.Z., G.S., D.C., M.A., P.V., E.G.B., V.F., L.B., G.M., P.B., F.S., F.B.P., declares no conflict of interest. I.C. and C.S. declare to own DeepTrace Technologies S.R.L shares. C.S. declares to be CEO of DeepTrace Technologies SRL. the Alzheimer's Disease Neuroimaging Initiative, Data used in preparation of this article were obtained from the Alzheimer's Disease Neuroimaging Initiative (ADNI) database (adni.loni.usc.edu). As such, the investigators within the ADNI contributed to the design and implementation of ADNI and/or provided data but did not participate in analysis or writing of this report. A complete listing of ADNI investigators can be found at: http://adni.loni.usc.edu/wpcontent/uploads/how_to_apply/ADNI_Acknowledgement_List.pdf

## EPR‐116

### CSF LDH is strongly associated with CSF Alzheimer's disease biomarkers: A real‐world cohort data

#### T. Oliveira ^1^; M. Coutinho^1^; M. Pereira^2^; J. Durães^2^; P. Faustino^2^; C. Bernardes^2^; M. Coelho^2^; S. Matos^2^; N. Ferreira^2^; C. Cunha^2^; M. Lima^2^; I. Baldeiras^2^; I. Santana^2^

##### ^1^ULS de São José, Lisboa, Portugal; ^2^ULS de Coimbra, Coimbra, Portugal

**Background and aims:** Disturbances in glucose transport mechanisms, oxidative stress, and impaired mitochondrial function are pathophysiological contributors to Alzheimer's disease (AD). We aimed to investigate the association of CSF LDH with established CSF AD biomarkers.

**Methods:** A retrospective single‐center observational study that included 265 patients with the diagnosis of AD at the stage of mild cognitive impairment and dementia, with confirmatory CSF biomarkers. We studied the relationship between CSF parameters (glucose, lactate dehydrogenase ‐ LDH, protein, chloride) and blood analytical parameters (glucose, proteins, albumin, C‐reactive protein) with AD CSF biomarkers levels.

**Results:** 265 patients were included, 56,6% were females. Mean age of onset was 63.77 ± 8.06 years. The mean MMSE score was 16.4 ± 7.54 points. Multiple regression, with AD CSF biomarkers levels, adjusted for age, MMSE, sex and education level showed that CSF LDH was positively correlated with CSF total tau (β = 0.66, *p*‐value < 000.1) and CSF p‐tau (β = 0.67, p‐value < 0.001) and negatively correlated with Aβ40/42 ratio (β = ‐0.58, p‐value < 0.001), with no correlation with Aβ42 level (β = 0.20, p‐value = 0.135). Multiple regression between CSF AD biomarkers and the rest of the analytical parameters didn’t show other significant associations.

**Conclusion:** LDH levels are increased in patients with acute brain injury and might reflect neuronal destruction. The positive correlation with the AD biomarkers for neurodegeneration might reflect impaired glucose metabolism, a key contributor to AD, but it can also be related to the loss of integrity of the neuronal membrane and more severe neurodegeneration. More studies are necessary to define the role and prognostic value of this biomarker in AD.

**Disclosure:** Nothing to disclose.

## Infectious diseases

## EPR‐117

### Meningoencephalitis caused by serratia marcescens in a 74‐year‐old patient with no significant medical history

#### A. Tsimpiktsioglou; M. Ioakeimidis; T. Doskas

##### Neurology Department, Athens Naval Hospital, Athens, Greece

**Background and aims:** We present a case of meningoecephalitis caused by Serratia marcescens and review the literature.

**Methods:** Case presentation

**Results:** A 74‐year‐old female patient with no significant medical history was hospitalized in the Neurology Department due to fever up to 40°C, confusion, and expressive aphasia. She also reported a few episodes of diarrhea within the previous 24 hours. The neurological examination revealed significant neck stiffness, a positive Kernig sign, and mild right hemiparesis. Based on these findings, a central nervous system (CNS) infection was suspected, and a lumbar puncture was performed. The analysis of the CSF revealed 5000 cells, with 96% neutrophills, elevated protein levels, and low glucose levels. Gram‐staining microscopy revealed a Gram‐negative bacillus. Cultures of the CSF grew Serratia marcescens. The patient was treated with 21 days of intravenous ceftazidime with excellent clinical and laboratory response.

**Conclusion:** Serratia marcescens is an extremely rare pathogen that causes acute CNS infection in adults. The majority of cases in the literature refer to patients who have recently undergone neurosurgical procedures. A few cases of this infection in intravenous drug users and neonates have also been reported. The is the first reported case of spontaneous CNS infection caused by Serratia marcescens in a patient with no significant medical history. As an enteropathogen, this case is considered an opportunistic infection with hematogenous spread, likely triggered by the intestinal inflammation that preceded the symptoms of the CNS infection.

**Disclosure:** Nothing to disclose.

## EPR‐118

### Fatal hemorrhagic leukoencephalitis following VZV infection in an immunocompetent host: A case report

#### G. Bonelli ^1^; G. Cutillo^1^; M. Rubin^1^; I. Gattuso^1^; M. Barbera^2^; L. Moiola^1^; G. Fanelli^1^; M. Filippi^1^

##### ^1^Neurology Unit, IRCCS S. Raffaele, Milan, Italy; ^2^Neuroloradiology Unit, IRCCS S. Raffaele, Milan, Italy

**Background and aims:** Varicella Zoster Virus (VZV) is a neurotropic virus primarily linked to neurological complications in immunocompromised individuals but rarely in immunocompetent hosts. This case report describes a fatal instance of hemorrhagic leukoencephalitis following VZV infection in an immunocompetent patient, emphasizing atypical neuroradiological features and negative cerebrospinal fluid (CSF) polymerase chain reaction (PCR) results.

**Methods:** Case description, literature review.

**Results:** A 76‐year‐old immunocompetent male with coronary artery disease on dual antiplatelet therapy (DAPT) and Parkinson's disease presented with transient loss of consciousness. Examination revealed mild extrapyramidal symptoms and right periocular crusting, consistent with recent Herpes Zoster treated two weeks earlier. Brain CT showed bilateral white matter hypodensities. Within hours, he deteriorated to coma. MRI revealed extensive T2/FLAIR hyperintensities in deep white matter, including the capsular, thalamic, and mesencephalic regions, rapidly extending with hemorrhagic transformation. Acyclovir, Methylprednisolone, and intravenous immunoglobulin were initiated. Delayed lumbar puncture showed elevated protein and pleocytosis, but VZV PCR was negative. Serum VZV PCR and serology were positive. Despite treatment, the patient succumbed.

**Figure:**
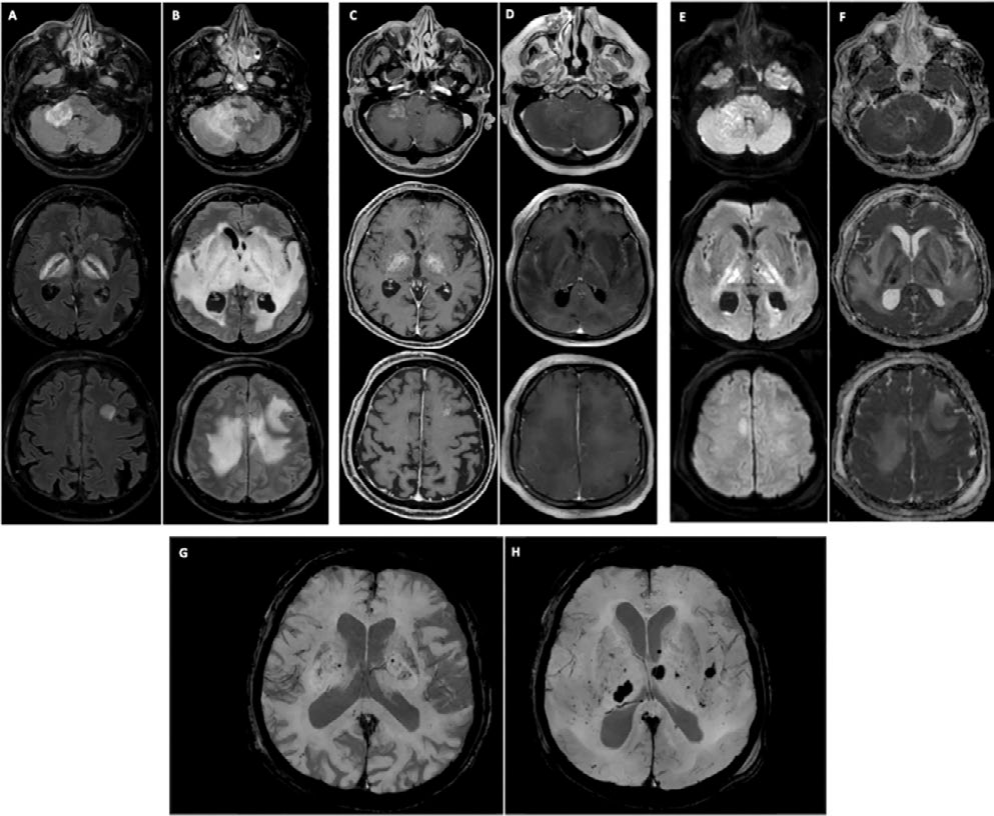
**FIGURE 1** Brain MRI of the patients at ER arrival and at follow‐up five days later.

**Conclusion:** This case highlights an atypical leukoencephalitic VZV presentation in an immunocompetent host, suggesting an immune‐mediated mechanism alongside direct viral effects. The symmetric white matter involvement with hemorrhagic transformation is reminiscent of acute hemorrhagic encephalomyelitis. Negative CSF PCR complicates diagnosis, underscoring the need for early recognition and combined immunomodulatory and antiviral therapy.

**Disclosure:** The authors have no disclosures related to the present work

## EPR‐119

### Clinical features and outcomes of neurocysticercosis patients in Thailand

#### C. Teekaput ^1^; K. Teekaput^2^; P. Kanjanahitanon^3^; P. Anakkanon^3^; C. Chimplee^3^; A. Pawabunsiriwong^3^; W. Teekaput^2^; R. Chaiwarith^4^

##### ^1^Division of Neurology, Department of Internal Medicine, Faculty of Medicine, Chiang Mai University, Chiang Mai, 50200, Thailand; ^2^Chiang Mai Municipality Hospital, Chiang Mai, 50000, Thailand; ^3^Faculty of Medicine, Chiang Mai University, Chiang Mai, 50200, Thailand; ^4^Division of Infectious Diseases and Tropical Infection, Department of Internal Medicine, Faculty of Medicine, Chiang Mai University, Chiang Mai, 50200, Thailand

**Background and aims:** Neurocysticercosis (NCC) is a life‐threatening disease with significant morbidity. This study evaluates clinical characteristics, risk factors, and outcomes among NCC patients in Northern Thailand.

**Methods:** A retrospective analysis was conducted on 106 NCC patients categorized into seizure (*n* = 74) and non‐seizure groups (*n* = 32). Demographics, clinical features, imaging findings, and treatment outcomes were compared using statistical analyses.

**Results:** The mean age was 48.3 ± 16.7 years, with seizure patients being younger than non‐seizure patients (45.4 ± 16.4 vs. 55.0 ± 15.8 years, p = 0.007). Males accounted for 64.2% of the cohort. Non‐seizure patients showed significantly higher rates of sensory deficits (28.1% vs. 10.8%, p = 0.04), intraventricular lesions (18.8% vs. 4.1%, p = 0.02), and hydrocephalus (34.4% vs. 5.4%, p < 0.001). Surgical intervention was more frequent in the non‐seizure group (21.9% vs. 6.8%, p = 0.04). Hospital stays tended to be longer in the non‐seizure group (median: 12 vs. 7 days, p = 0.07). Overall mortality was 0.9%, with no significant difference between groups.

**Conclusion:** Non‐seizure NCC patients exhibit distinct clinical characteristics, including higher rates of sensory deficits, intraventricular lesions, and hydrocephalus. These findings suggest a different disease phenotype, highlighting the need for tailored diagnostic and management strategies.

**Disclosure:** Nothing to disclose.

## EPR‐120

### Substantia Nigra encephalitis and post‐viral parkinsonism: case report and literature review

#### I. Pana ^1^; V. Tiu^1^; V. Tiu^2^; C. Ghita^2^; R. Urdea^2^; C. Panea^1^; C. Panea^2^

##### ^1^Carol Davila University of Medicine and Pharmacy, Bucharest, Romania; ^2^Elias Emergency University Hospital, Bucharest, Romania

**Background and aims:** Viral infections such as influenza, Coxsackie, Epstein‐Barr viruses, and Japanese encephalitis virus have been incriminated as rare causes of secondary parkinsonism due to their tropism for mesencephalon and diencephalon, leading to severe nigral cell loss and, consequently, to a broad spectrum of symptoms, from Parkinsonism to signs of widespread neuronal damage. We present a literature review of Substantia Nigra (SN) encephalitis starting from a case report.

**Methods:** We perform a literature review regarding potential causes of SN encephalitis. We present one such case admitted in our clinic.

**Results:** A 40‐year‐old female was admitted to our clinic with altered consciousness, GCS = 8 points(E2V2M4), flaccid tetraplegia and tonic‐clonic seizures. Lab results and vitals indicated septic shock, with pathogen screening returning positive blood culture for E.Coli. Extensive serologic and CSF screening detected the presence of Anti Cytomegalovirus IgG and IgM. Head MRI revealed bilateral, symmetrical T2/FLAIR hyperintensities involving the substantia nigra, suggesting a possible CMV SN encephalitis. The patient progressed to atypical post‐viral parkinsonism with a mild improvement under Levodopa/Carbidopa, before infectious complications led to her death. Cases of SN encephalitis have been documented since the encephalitis lethargica outbreak after the 1918 influenza epidemic, but they have been also linked to numerous other viral infections that had a tropism for SN due to numerous hypothesized mechanisms that were based on neurovirulence.

**Figure:**
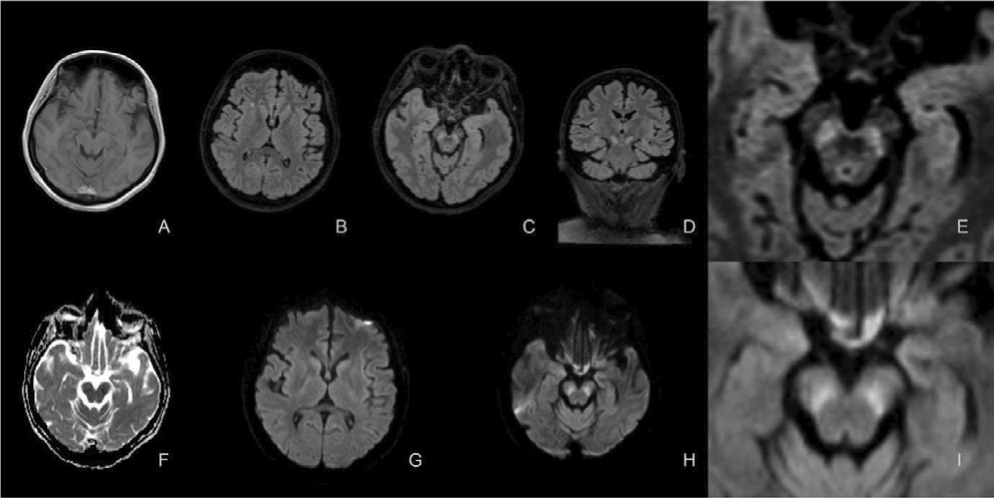
**FIGURE 1** Cerebral MRI showing bilateral extensive substantia nigra lesions, with hyperintense signal in T2/FLAIR and restriction of diffusion on DWI.

**Conclusion:** SN encephalitis is a rare complication of viral infections. This case highlights diagnostic challenges, the impact of viral infections upon SN inflammation and the severe outcome many of these patients face

**Disclosure:** Nothing to disclose.

## EPR‐121

### Clinical characteristics and treatment outcomes in herpes simplex type I encephalitis

#### J. Kim; J. Woo; G. Nam; H. Kim

##### Department of Neurology, Hallym University Dongtan Sacred Heart Hospital

**Background and aims:** Herpes simplex virus type 1 (HSV‐1) is leading cause of sporadic infectious encephalitis. The mortality rate of HSV‐1 encephalitis (HSV1E) has been reported below 20% but survivors still have a significant morbidity rate of 60~70%. We reviewed clinical characteristics and treatment outcomes of HSV1E in single center in South Korea.

**Methods:** We analyzed 12 patients of PCR positive HSV1E who were admitted to Department of Neurology at Hallym Medical Center (2017~2022).

**Results:** The mean age was 51.7 years. Presenting symptoms were headache (58%), fever (58%), seizure (50%), confusion (33%), and aphasia (33%). CSF study showed lymphocytic pleocytosis (185.7 ± 215.4 cells/μL) and mildly elevated protein (60.1 ± 25.9 mg/dL) but CSF glucose was not decreased. Brain MRI demonstrated typical unilateral or asymmetric edematous lesions in medial and anterior temporal areas (11 patients), also in insular cortex (7 patients), inferior frontal lobe (2 patients), and thalamus (3 patients). Brain MRI was negative in 1 patient. All patients were treated with intravenous acyclovir (10 mg/kg every 8 hours) at least for 14 days. Corticosteroid was given in 8 patients (66.7%). There was no significant difference in outcomes of patients with or without corticosteroid. Two patients developed antibody‐negative autoimmune encephalitis and the time of onset after HSV1E was 14 days and 180 days each. After 6 months, no one died but 5 patients (41.6%) had a moderate to severe disability.

**Conclusion:** The outcome of this case series is better than previous reports. Early suspicion and early empiric acyclovir therapy could reduce mortality and morbidity.

**Disclosure:** Nothing to disclose.

## EPR‐122

### Therapeutic approaches for ramsay hunt syndrome affecting multiple nerves: A systematic literature review

#### A. Reyes^1^; K. Rodríguez‐Manso^1^; M. Saint‐Felix^1^; D. Almánzar^1^; K. Yorro^1^; C. Lizardo^1^; A. Tejada^1^; M. Rodríguez ^2^

##### ^1^Universidad Iberoamericana, Mexico City, Mexico; ^2^Department of Neurology, Salvador Bienvenido Gautier University Hospital, Santo Domingo, Dominican Republic

**Background and aims:** Ramsay Hunt syndrome (RHS) involves reactivation of the varicella‐zoster virus in the geniculate ganglion, affecting the facial nerve (CN7) [1]. This leads to ipsilateral facial paralysis and may extend to nearby cranial nerves, causing symptoms like hearing loss, tinnitus, or vertigo [2]. Even with the unusualness of this, there´s no description of treatments and outcomes in these patients. This study aimed to highlight the interplay between cranial nerve dysfunction and posterior recovery, based on treatment regime.

**Methods:** We conducted a systematic literature review following PRISMA protocols, emphasizing abnormal presentations. PubMed database was searched until 2014 using keywords: “Ramsay‐Hunt Syndrome”, and “Nerve”. 45 studies found. We evaluated age, cranial nerve involvement, and outcome.

**Figure:**
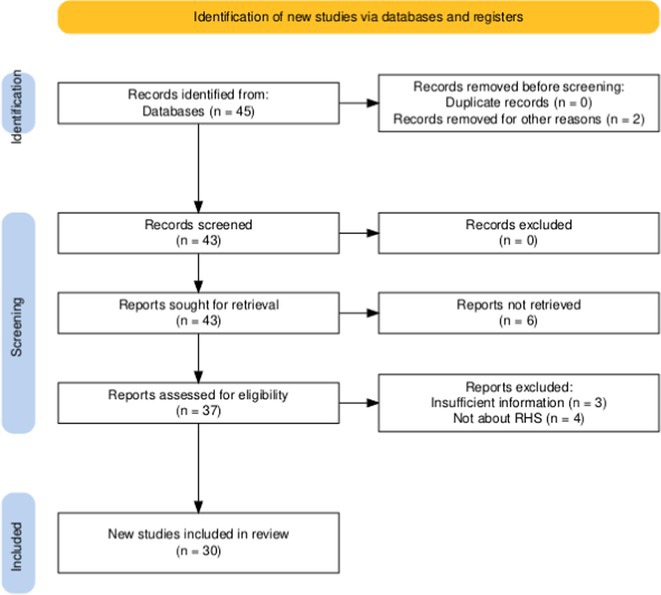
**FIGURE 1** PRISMA flowchart presenting the process of inclusion of studies for systematic review

**Results:** We included 30 studies, reporting 33 patients with a mean age of 56 years. Of which, 10 (30%) were female, and 23 (60%) male. 91% of patients presented CN7 involvement, 48.4% with vestibulocochlear, 36% trigeminal, and 27% vagus. 60% of patients treated with valacyclovir didn´t recover their functionality in nerves besides the CN7, compared to 47.6% of those treated with acyclovir. 43.7% of patients with vestibulocochlear involvement presented better prognosis using acyclovir, while valacyclovir obtained fewer results. Both treatments presented better recovery when administered with corticosteroids.

**Figure:**
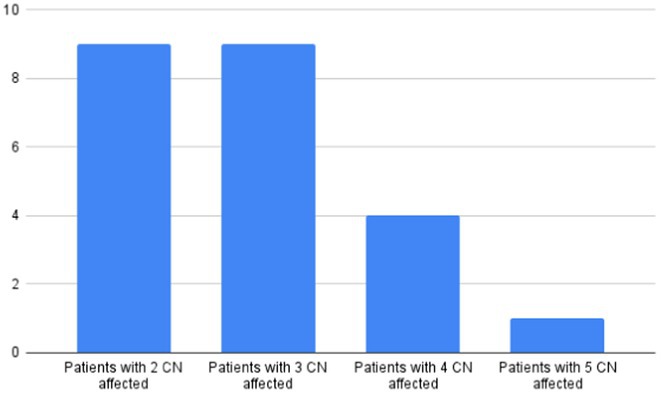
**FIGURE 2** Patients based on the number of structures affected

**Conclusion:** Although rare, cranial nerve involvement is a challenge in rehabilitation of RHS patients. This study provides a list of cases where the use of acyclovir showed a better prognosis when other cranial nerves are affected, contrasting with literature around CN7 involved alone, which might be inconclusive or opposite [3].

**Disclosure:** Nothing to disclose.

## EPR‐123

### Focal deficits mimicking stroke in post‐malaria neurological syndrome

#### S. Moreira; A. Câmara; A. Costa; J. Araújo; J. Alves; C. Ferreira; J. Pinto

##### Neurology, Unidade Local de Saúde de Braga, Braga, Portugal

**Background and aims:** Post‐malaria neurological syndrome (PMNS) is a rare but clinically significant complication emerging after resolution of acute malaria, particularly with Plasmodium falciparum, appearing days to weeks post‐treatment. Although only a few dozen cases are reported, actual incidence may be underrecognized.

**Methods:** Clinical case.

**Results:** A 60‐year‐old male, who had emigrated to Angola without malaria prophylaxis, was first admitted with persistent fever and altered consciousness requiring mechanical ventilation. He was diagnosed with severe Plasmodium falciparum malaria, presenting neurological, hematological, renal, and cardiovascular dysfunction. Following antimalarial therapy, he fully recovered. Several weeks later, he presented with sudden right hemispheric dysfunction, including left facial droop and hemiparesis. Brain imaging showed no large‐vessel occlusion, but CT‐perfusion revealed right hemispheric hypoperfusion. Tenecteplase was given. Subsequently, he developed fever and altered consciousness. Tests for malarial parasites and antigens were negative. Lumbar puncture revealed 47 cells (83% lymphocytes) with normal protein and negative meningitis/encephalitis panel. Electroencephalography showed focal slow theta activity in the right temporal region, without epileptiform discharges. Magnetic resonance imaging demonstrated multifocal lobar microhemorrhages. PMNS was presumed, and high‐dose steroids led to full recovery in 15 days.

**Conclusion:** Despite underdiagnosis in endemic regions, PMNS may appear in non‐endemic settings due to global travel. Recognizing this condition is crucial for timely diagnosis and management, as it responds to corticosteroids and may be self‐limiting. Investigation is warranted to clarify pathophysiological mechanisms, particularly in endemic areas, where exposure to Plasmodium might modulate immune responses and influence clinical outcomes.

**Disclosure:** Nothing to disclose.

## EPR‐124

### Balint's syndrome as a manifestation of post‐varicella acute disseminated encephalomyelitis in adults

#### Y. Naji; W. Hrouch; H. Lagtarna; N. Adali

##### Neurology department, University Hospital of Agadir, Agadir, Morocco. “N.I.C.E.” Research Team, “R.E.G.N.E.” Research laboratory, Faculty of Medicine and Pharmacy, Ibn Zohr University, Agadir, Morocco

**Background and aims:** Balint's syndrome (BS) is a rare neurological disorder characterized by a triad of simultanagnosia, optic ataxia, and oculomotor apraxia. It is typically associated with bilateral parieto‐occipital lesions and, in rare cases, can be an initial manifestation of acute disseminated encephalomyelitis (ADEM).

**Methods:** A 55‐year‐old man with a history of varicella 20 days before admission, presented with bilateral visual acuity decline, and speech impairment. On admission, the patient was disoriented in time and space, neurological examination revealed severe optic ataxia, simultaneous agnosia, oculomotor apraxia, and cerebellar syndrome. Ophthalmologic examination revealed a profound loss of visual acuity. Brain magnetic resonance imaging (MRI) revealed multiple patchy and nodular lesions in the bilateral parieto‐occipital regions, with ring enhancement after gadolinium injection. Laboratory results were unremarkable. The patient received intravenous methylprednisolone for five days, followed by oral corticosteroid therapy for three months, leading to progressive clinical improvement and complete resolution of the MRI lesions after eight months. A final diagnosis of BS associated with ADEM was confirmed.

**Figure:**
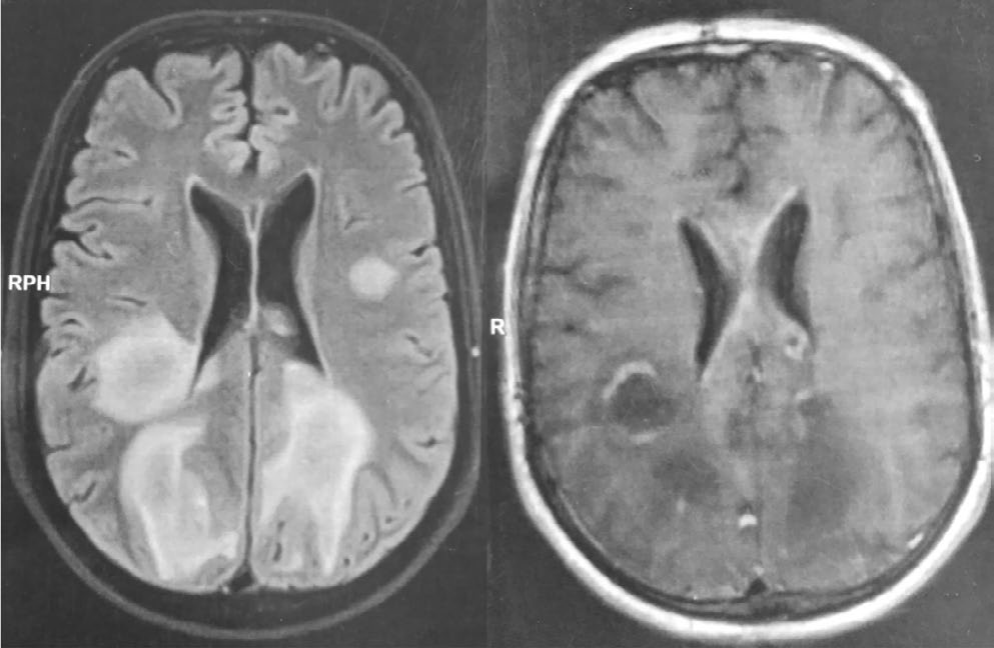
**FIGURE 1** Brain magnetic resonance imaging (T1 GADO and Flair) showed multiple patchy and nodular lesions in the bilateral parieto‐occipital regions, with ring enhancement after gadolinium injection

**Results:** ADEM is an acute demyelinating disease of the central nervous system that often occurs after a viral infection, particularly in children. It presents with diverse symptoms and atypical presentations such as BS. The precise mechanisms linking ADEM and BS remain unclear but suggest that extensive demyelination leads to a disconnection between posterior visual association areas and the motor regions of the prefrontal cortex.

**Conclusion:** The association between BS and ADEM is extremely rare. This case underscores the importance of recognizing complex visual disturbances in atypical ADEM presentations.

**Disclosure:** Nothing to disclose.

## Cerebrovascular diseases 2

## EPR‐125

### Capillary index score in mechanical thrombectomy: Predicting functional outcomes and identifying no‐reflow risk

#### A. Scartezzini ^1^; M. Plebani^2^; C. Zivelonghi^1^; N. Mandruzzato^2^; M. Cappellari^1^; B. Petralia^2^; B. Bonetti^1^; N. Micheletti^1^

##### ^1^Neurology Unit, University Hospital Verona, Italy; ^2^Neuroradiology Unit, University Hospital Verona, Italy

**Background and aims:** Despite advances in mechanical thrombectomy (MT), futile recanalization remains a major challenge, affecting nearly 50% of successfully recanalized patients. The no‐reflow phenomenon (NRP), a form of persistent microvascular dysfunction, is thought to contribute significantly. The Capillary Index Score (CIS), an angiographic marker based on procedural digital subtraction angiography (DSA), may help assess microvascular integrity, but its prognostic role in acute ischemic stroke (AIS) remains underexplored. We aimed to evaluate the predictive role of CIS before MT (pre‐CIS) and after MT (post‐CIS) for functional outcomes (mRS at 90 days) and hemorrhagic transformation (HT) in AIS patients treated with MT.

**Methods:** A single‐center, retrospective‐prospective study included 117 AIS patients with anterior circulation large vessel occlusions treated with MT. CIS was assessed pre‐ and post‐procedurally. Univariate and multivariate logistic regression identified predictors of unfavorable outcomes (mRS ≥3) and HT. ROC curves and ANOVA likelihood ratio tests evaluated model performance.

**Results:** A favorable pre‐CIS ( > 2) was an independent predictor of functional independence (OR 0.17, 95% CI 0.08–0.88, *p* = 0.03), confirming its role as an early marker of microvascular integrity. Pre‐CIS improved predictive model performance (*p* = 0.034). Post‐CIS reflected persistent microvascular dysfunction and showed a trend toward higher HT rates (*p* = 0.071), suggesting its role in identifying patients at risk of no‐reflow.

**Conclusion:** Pre‐CIS is confirmed as an early predictor of functional outcomes, while post‐CIS highlights persistent no‐reflow and its potential link to HT. CIS may enhance prognostic models, potentially serving as a tool for decision‐making, though further validation in larger cohorts is warranted.

**Disclosure:** Nothing to disclose.

## EPR‐126

### CD177null genotype on neutrophils predicts poor neurological outcome in ischemic stroke patients

#### C. Graser ^1^; J. Gronewold^1^; T. Tertel^2^; N. Hagemann^1^; M. Jung^3^; A. Giese^1^; A. Moenig^1^; M. Niklas^1^; M. Nanthagopal^1^; M. da Silva Rafael^1^; Y. Zhang^1^; B. Giebel^2^; M. Gunzer^3^; D. Hermann^1^

##### ^1^Department of Neurology, University Hospital Essen, University of Duisburg‐Essen, Essen, Germany; ^2^Institute for Transfusion Medicine, University Hospital Essen, University of Duisburg‐Essen, Essen, Germany; ^3^Institute for Experimental Immunology and Imaging, University Hospital Essen, University of Duisburg‐Essen, Essen, Germany

**Background and aims:** Neutrophils are important contributors to ischemic brain injury. In preclinical rodent studies, the lack of the neutrophilic surface molecule Ly6G was associated with exacerbated stroke damage. Ly6G is thought to represent an “eat me” signal on neutrophils that ensures neutrophil phagocytosis. Human neutrophils do not express Ly6G, but an analogue, CD177. CD177 is lacking in ~5% of the general population. The consequences of the CD177null genotype for stroke recovery were unknown.

**Methods:** In a prospective study, patients with first‐time ischemic strokes are recruited at the University Hospital Essen. Peripheral blood samples are taken. CD177 and various other immune cell markers are analyzed by flow cytometry. Clinical examinations and interviews are conducted to assess vascular risk factors and neurological impairments. Patients are examined on three time‐points: T1 = < 72h, T2 = ≥72h–144h, T3 = 3 months post‐stroke.

**Results:** From October 18th, 2022 to October 20th, 2024, 236 stroke patients were included. By flow cytometry, 10 patients were CD177null and 226 CD177WT genetopes. CD177null and CD177WT patients showed similar vascular risk factor profiles except that CD177null patients exhibited more often a history of TIA (20% vs. 0.4%, *p* = 0.073), carotid dissection (33.3% vs. 5.4%, *p* = 0.015) and regular sports activity (*p* = 0.024) (Figure 1). Compared with CD177WT patients, CD177null patients had higher neurological deficits in the National Institutes of Health Stroke Scale at all time‐points examined, most prominently at T2 and T3, and a higher disability in the modified Rankin Scale at T3 (Figures 2, 3).

**Figure:**
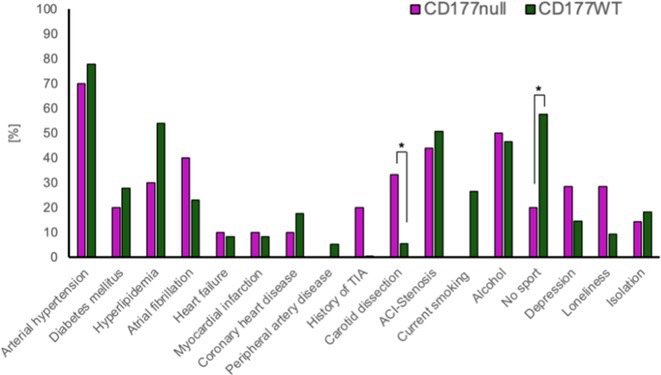
**FIGURE 1** Vascular risk profiles in CD177null and CD177WT stroke patients. Percentages of each vascular risk factor are shown.

**Figure:**
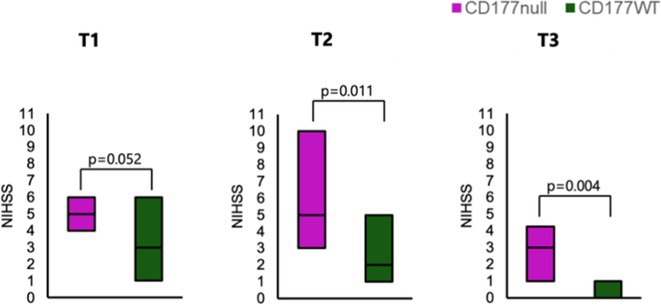
**FIGURE 2** National Institutes of Health Stroke Scale score in CD177null and CD177WT stroke patients. Median, Q1 and Q3 are shown.

**Figure:**
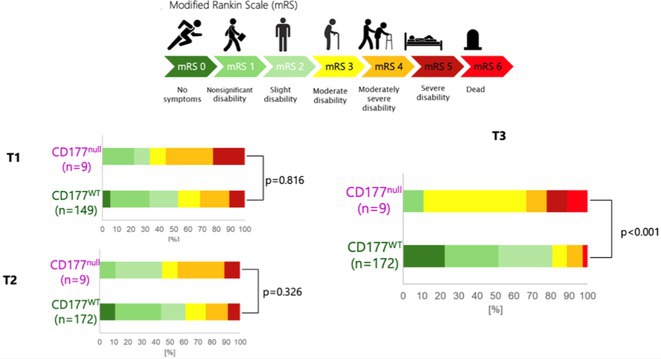
**FIGURE 3** Modified Rankin scale scores in CD177null and CD177WT stroke patients. Percentages of each score are shown.

**Conclusion:** CD177 deficiency is associated with poor neurological outcome in ischemic stroke patients.

**Disclosure:** Supported by DFG TRR332 (449437943).

## EPR‐127

### Predictors of intracranial non‐stenosing vulnerable atherosclerotic plaques in ESUS: A risk stratification model

#### F. Mazzacane ^1^; B. Del Bello^1^; C. Asteggiano^1^; E. Rognone^2^; F. Ferrari^1^; A. Persico^3^; A. Costa^1^; R. De Icco^1^; A. Morotti^4^; A. Pichiecchio^1^; A. Cavallini^3^

##### ^1^Department of Brain and Behavioral Sciences, University of Pavia, Pavia, Italy; ^2^Department of Neuroradiology, IRCCS Mondino Foundation, Pavia, Italy; ^3^Department of Stroke Unit and Emergency Neurology, IRCCS Mondino Foundation, Pavia, Italy; ^4^Department of Clinical and Experimental Sciences, Neurology Unit, University of Brescia, Brescia, Italy

**Background and aims:** Intracranial vulnerable non‐stenosing atherosclerotic plaques (vNSPs) are emerging as potential causes of Embolic Strokes of Undetermined Source (ESUS). While Vessel Wall MRI (VWMRI) is a valuable tool for detecting vNSPs, its limited availability and high technical complexity necessitate the identification of high‐risk patients for targeted imaging.

**Methods:** A prospective cohort of 80 single‐territory ESUS patients undergoing VWMRI was analyzed. A multivariable logistic regression model including the clinical predictors of vNSPs was developed. Model performance was evaluated using the area under the curve (AUC) of the receiver operating characteristic (ROC) curve, specificity, sensitivity, positive predictive value (PPV), and negative predictive value (NPV).

**Results:** Age (adjusted odds ratio [aOR] 1.12, 95% confidence interval [CI] 1.05–1.23, *p* = 0.04) and smoking exposure (aOR 8.48, 95% CI 2.32–40.6, *p* = 0.003) were independently associated with culprit vNSPs. The model, incorporating these two variables, demonstrated good discriminatory performance (AUC 0.84, 95% CI 0.76–0.92). Specificity, sensitivity, NPV, and PPV were 0.70, 0.91, 0.95, and 0.55, respectively.

**Figure:**
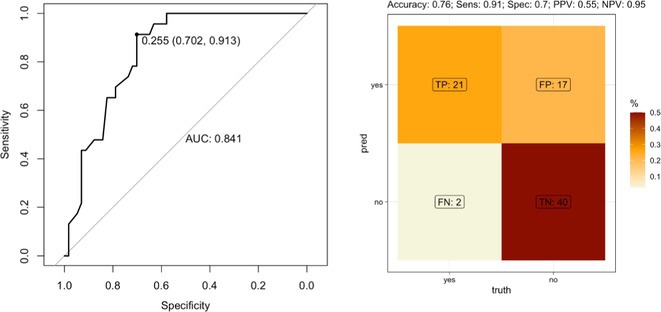
**FIGURE 1** Panel A: ROC curve for the predicted probability of the presence of a culprit vNSP. Panel B: confusion matrix. TP, true positives; FP, false positives; TN true negatives; FN, false negatives.

**Conclusion:** This simple clinical model shows promise in stratifying ESUS patients for VWMRI, with high NPV aiding in excluding low‐risk patients thus optimizing the diagnostic yield and resource utilization. However, the low PPV highlights the need for additional predictive factors, such as imaging and blood biomarkers, to refine risk stratification. In conclusion, a model based on simple clinical features demonstrated good performances in in identifying ESUS patients at risk for intracranial culprit vNSPs. Further studies and external validation are essential to confirm these results.

**Disclosure:** Funding: “Ricerca Corrente 2022‐2024” granted to IRCCS Mondino Foundation.

## EPR‐128

### Spatial transcriptomics of dopaminergic neuron degeneration in rat ventral tegmental area after motor‐cortical stroke

#### G. Kastner ^1^; M. Mühlbauer^1^; R. Stockfleit^2^; A. Jockers^2^; J. Zhang^3^; N. Neidert^3^; D. Heiland^4^; S. Frase^2^; J. Hosp^2^

##### ^1^DKTK, German Cancer Consortium partner site Freiburg, Freiburg, Germany; ^2^Department of Neurology, Medical Center ‐ University of Freiburg, Freiburg; ^3^Department of Neurosurgery, Medical Center ‐ University of Freiburg, Germany; ^4^Department of Neurosurgery, Medical Center ‐ University of Erlangen–Nuremberg, Erlangen, Germany

**Background and aims:** For motor learning, the integrity of dopaminergic (DAergic) pathways from the ventral tegmental area (VTA) to the cortex is essential. Secondary degeneration of DAergic VTA‐neurons after stroke, however, impedes re‐learning of lost motor skills. To study the time‐dependent impact of ischemic cortical stroke on the microenvironment of this complex brain region, we traced the cell type‐specific dynamics of DAergic degeneration in the VTA of adult rats, along with the response in the peri‐infarct cortex.

**Methods:** Adult Sprague‐Dawley rats (n = 2) received a photothrombotic stroke in the motor‐cortical area. Fresh‐frozen, OCT‐embedded samples from the midbrain and cortex were collected at post‐stroke days 3 and 7. Spatial transcriptomics (50 μm spots) was performed and integrated using deep learning and deconvolution techniques to profile the spatially resolved gene expression patterns at an inferred cellular level in the tissue. DAergic neuron degeneration was further validated via tyrosine hydroxylase‐positive (Th+) immunostaining.

**Results:** We analyzed the ventral midbrain including the substantia nigra and VTA for changes in cell type composition. Progressive degeneration of DAergic VTA‐neurons was observed, with the ipsilateral/contralateral ratio decreasing from 0.96 on day 3 to 0.76 on day 7 post‐stroke (*p* < 0.03). This reduction was independently validated through Th+ immunostaining (*p* < 0.0001). Additionally, reactive astrocytes marked by Gfap (*p* < 1e‐10) increased at day 3 in the ipsilateral midbrain hemisphere.

**Conclusion:** Our preliminary findings suggest spatiotemporal gene expression patterns of DAergic neurodegeneration in the midbrain, providing a robust model for exploring strategies to support post‐stroke recovery. Currently, we are complementing the data with additional time‐points and single‐nucleus sequencing.

**Disclosure:** Nothing to disclose.

## EPR‐129

### Early nutrition support enhances recovery after endovascular thrombectomy: A prospective study with historical control

#### . Maekawa ^1^; N. Ohara^1^; N. Higashibeppu^2^; M. Kawamoto^1^; T. Ohta^3^

##### ^1^Department of Neurology, Kobe City Medical Center General Hospital, Hyogo, Japan; ^2^Department of Anesthesiology, Kobe City Medical Center General Hospital, Hyogo, Japan; ^3^Department of Neurosurgery, Kobe City Medical Center General Hospital, Hyogo, Japan

**Background and aims:** We investigated the effectiveness of a comprehensive nutritional intervention provided by a multidisciplinary healthcare team on outcomes in acute stroke patients undergoing endovascular thrombectomy (EVT).

**Methods:** This prospective cohort study examined acute stroke patients undergoing EVT and achieved successful recanalization with comprehensive nutritional intervention, compared to a propensity‐matched historical control group. A total of 70 patients were prospectively enrolled between April 2022 and March 2024. The historical control group consisted of 427 consecutive patients treated between January 2015 and December 2020. After propensity matching, 42 pairs were included. The intervention protocol utilized a multidisciplinary healthcare team, including physicians, nurses, dietitians, speech‐language pathologists, and pharmacists to achieve targeted nutrition, aiming to initiate within two days and reach requirements (30 kcal/kg and 1.5 g/kg protein) within one week, with success defined as reaching ≥70% of targets. The primary outcome was the modified Rankin Scale (mRS) at 90 days.

**Figure:**
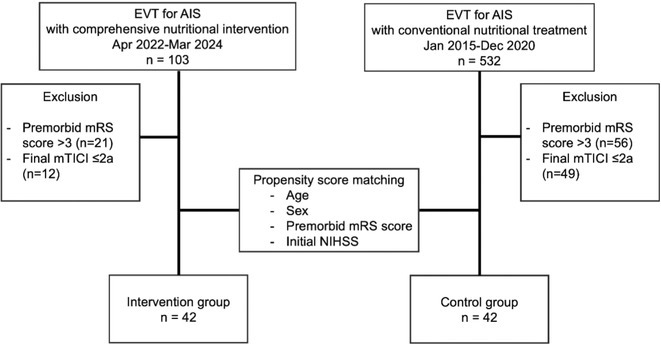
**FIGURE 1** Patient selection.

**Results:** The matched cohort (median age 78.0 years, 53.3% male) showed comparable baseline and EVT‐related characteristics. Nutritional therapy was initiated within two days in 92.8% and 78.5% of the intervention and control groups, respectively (*p* = 0.126). The intervention group showed favorable effects on 90‐day mRS scores (adjusted common odds ratio: 2.280, 95% CI 1.040‐5.080, *p* = 0.041). Target nutrient intake achievement was higher in the intervention group (95.2% vs. 76.1%, *p* = 0.025). No significant differences were observed in complications including aspiration pneumonia, diarrhea, and hyperglycemia.

**Figure:**
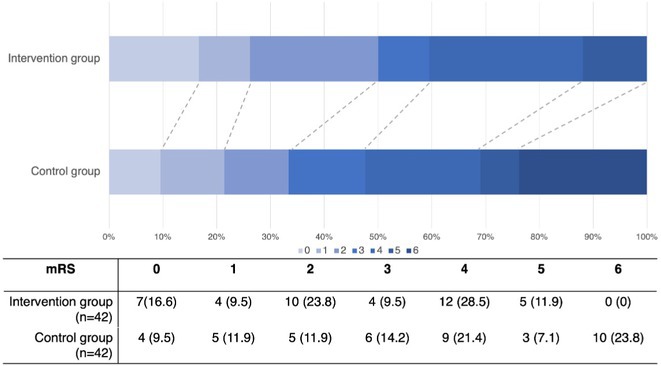
**FIGURE 2** Distribution of mRS scores at 90 days following EVT.

**Conclusion:** Comprehensive nutritional intervention in the acute phase improved functional outcomes in EVT patients, suggesting its potential benefit in post‐stroke care management.

**Disclosure:** Nothing to disclose.

## EPR‐130

### Low fibrinogen after intravenous thrombolysis: a possible predictor of hemorrhagic intracranial complications?

#### L. Simunek ^1^; V. Kunesova^2^; R. Mikulik^3^; R. Jura^4^; D. Vaclavik^5^; P. Geier^6^; M. Sramek^7^; J. Fiksa^8^; R. Herzig^1^

##### ^1^Department of Neurology, Comprehensive Stroke Center, Charles University, Faculty of Medicine and University Hospital in Hradec Kralove, Hradec Kralove, Czechia; ^2^Cerebrovascular Research Program, International Clinical Research Centre, St. Anne's University Hospital, Brno, Czechia; ^3^Department of Neurology, T. Bata Regional Hospital, Zlin, Czechia; ^4^Departmentof Neurology, Masaryk University, Faculty of Medicine and University Hospital, Brno, Czechia; ^5^Department of Neurology, Faculty of Medicine, University of Ostrava and University Hospital, Ostrava, Czechia; ^6^Department of Neurology, Faculty of Health Studies, Pardubice University and Pardubice Hospital, Pardubice, Czechia; ^7^Department of Neurology, Central Military Hospital in Prague, Prague, Czechia; ^8^Department of Neurology, Charles University, 1st Faculty of Medicine and General University Hospital, Prague, Czechia

**Background and aims:** Current Czech guidelines for treatment with intravenous thrombolysis (IVT) include the examination of coagulation parameters, including fibrinogen levels, before IVT, 6 hours and 24 hours after IVT, but there is no official recommendation on when to substitute fibrinogen, and whether to do so at all. Substitution is available in hospitals (Hemocomplettan i.v.). Fibrinogen substitution may be a way to prevent the development of bleeding complications during IVT treatment. The aim of this study was to evaluate whether a decrease in fibrinogen levels below the reference range of 1.8 g/l is associated with a higher likelihood of intracranial hemorrhage in patients with ischemic stroke treated with IVT.

**Methods:** This multicenter retrospective study was conducted in 7 Czech centers. The study population consists of 280 patients enrolled in the observational group (divided into subgroups according to the type of hemorrhagic complication after IVT) and the control group (without hemorrhagic complication) according to the model 1:1 principle (same NIHSS, age, sex).

**Results:** In all treatment groups (all observational subgroups as well as the control group), a decrease in fibrinogen levels was noted after IVT administration, with varying degrees of signification. A statistically significant difference (*p* = 0.0394) was found at 6 h after IVT administration in patients with the most clinically serious form of bleeding (parenchymal hematoma type 2), fibrinogen value 1.84 g/l versus 2.53 g/l.

**Figure:**
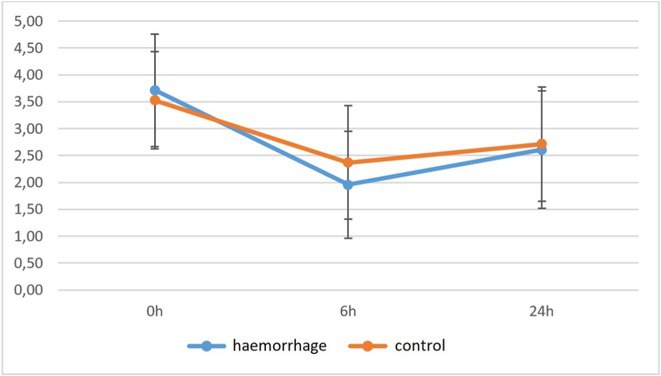
**FIGURE 1** Fibrinogen level in subgroup “parenchymal hematoma 2” and its control before IVT, 6 hours and 24 hours after IVT.

**Conclusion:** Patients with the clinically most severe form of hemorrhagic complication (parenchymal hematoma type 2) had significantly lower fibrinogen levels than those in the control group 6 hours after IVT administration.

**Disclosure:** Nothing to disclose.

## EPR‐131

### Outcomes of early vs. delayed anticoagulant Initiation in stroke patients + atrial fibrillation: Updated meta‐analysis

#### N. Nhan ^1^; H. Vinh^1^; N. Vy^2^; T. Nghi^1^; L. Oláh^3^

##### ^1^University of Debrecen Medical School, Debrecen, Hungary; ^2^The University of Melbourne, Melbourne, Australia; ^3^University of Debrecen Medical School, Department of Neurology, Debrecen, Hungary

**Background and aims:** Large randomized trials show direct oral anticoagulants (DOACs) reduce ischemic stroke risk in atrial fibrillation (AF) patients by ~2/3, with low intracranial hemorrhage risk. However, limited data exist on long‐term outcomes of early versus delayed NOAC/DOAC therapy, particularly within 90 days post‐stroke.

**Methods:** We conducted a systematic review and meta‐analysis by searching PubMed and the Cochrane database for randomized controlled trials (RCTs) and observational studies comparing early (≤4 days after stroke onset) versus delayed NOAC/DOAC therapy in ischemic stroke patients with AF. Outcomes included (1) recurrent ischemic stroke (IS), (2) intracranial bleeding (IB), and (3) all‐cause mortality (AM). Heterogeneity was assessed using the I^2^ statistic.

**Results:** Six studies (3 RCTs, 3 observational; n = 8,892) were included, with 4,076 patients receiving early NOACs. At 90 days, there were no significant differences in recurrent IS (RR 1.07; 95% CI 0.64–1.77; *p* = 0.80), IB (RR 0.90; 95% CI 0.47–1.70; *p* = 0.74), or AM (RR 0.79; 95% CI 0.52–1.20; *p* = 0.26). Subgroup analysis of RCTs alone showed consistent results, including the new OPTIMAS trial, published in November 2024. When hypothetically excluding OPTIMAS, early NOAC initiation significantly reduced recurrent IS risk (RR 0.63; 95% CI 0.41–0.98; *p* = 0.04).

**Figure:**
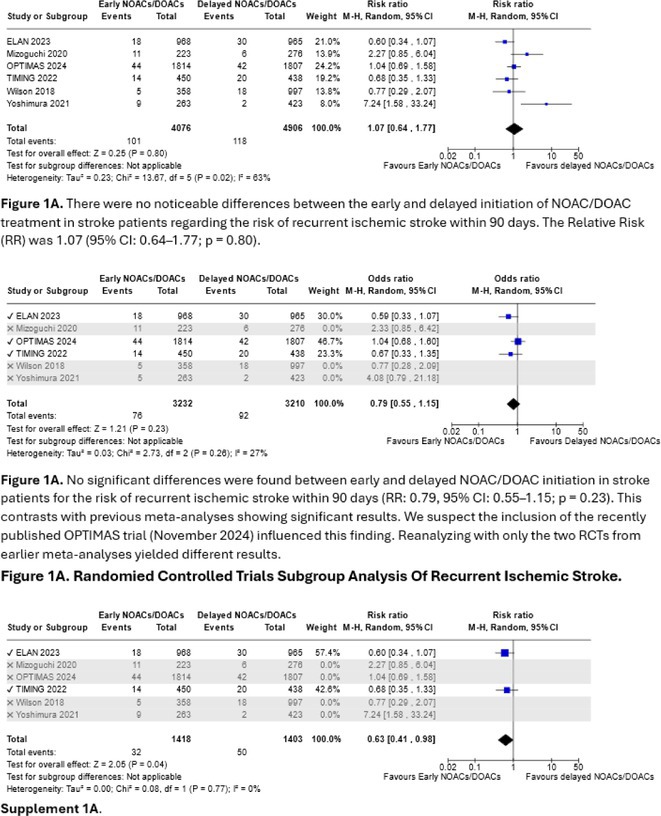
**Figure 1A**. Recurrent Ischemic Stroke Within 90 Days.

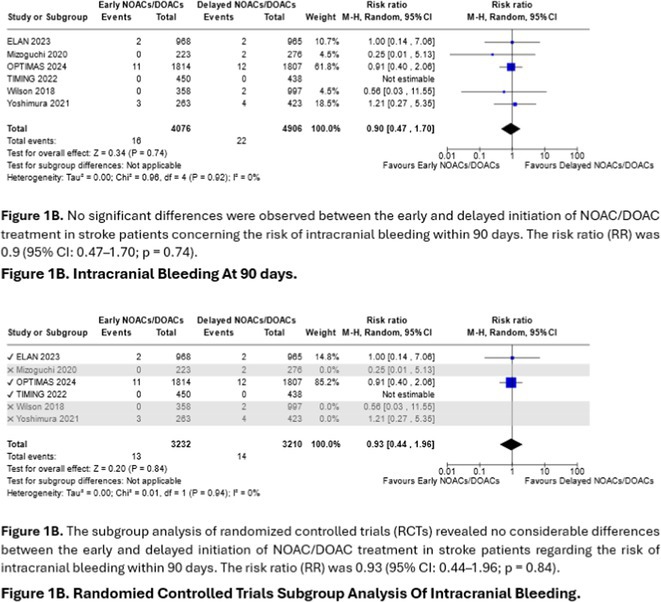

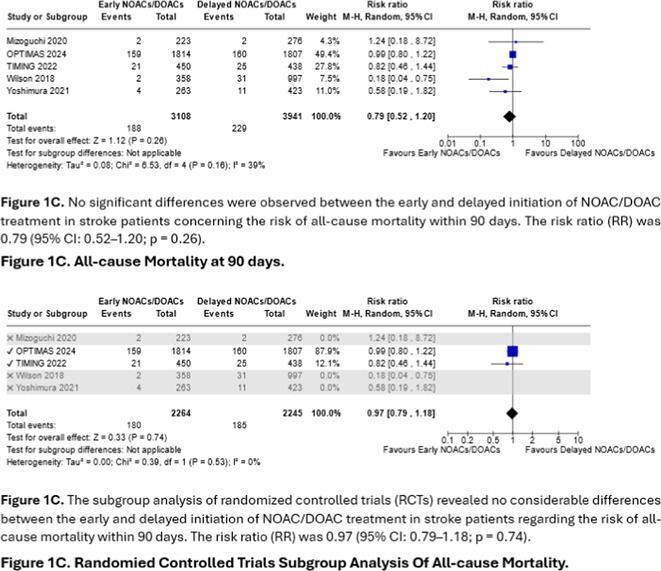

**Conclusion:** These findings suggest no significant differences in safety or efficacy between early and delayed NOAC/DOAC initiation within 90 days in stroke patients with AF. While current evidence supports the non‐inferiority of early therapy, further RCTs are needed to establish the long‐term superiority of early treatment.

**Disclosure:** Nothing to disclose.

## EPR‐132

### Safety and efficacy of CEA and IVT in acute stroke and occluded cervical internal carotid artery

#### R. Herzig ^1^; I. Guňka^2^; S. Ostrý^3^; J. Fiedler^4^; V. Přibáň^5^; M. Kovář^6^; P. Štádler^7^; O. Škoda^8^; J. Neumann^9^; V. Kunešová^10^

##### ^1^Department of Neurology, Charles University Faculty of Medicine and University Hospital Hradec Kralove, Hradec Kralove, Czechia; ^2^Department of Surgery, University Hospital Hradec Kralove, Hradec Kralove, Czechia; ^3^Department of Neurology, Comprehensive Stroke Centre, Ceske Budejovice Hospital, Ceske Budejovice, Czechia; ^4^Department of Neurosurgery, Comprehensive Stroke Center, Ceske Budejovice Hospital, Ceske Budejovice, Czechia; ^5^Department of Neurosurgery, University Hospital Pilsen, Faculty of Medicine in Pilsen, Charles University, Pilsen, Czechia; ^6^Department of Neurology, Comprehensive Stroke Center, Na Homolce Hospital, Prague, Czechia; ^7^Department of Vascular Surgery, Na Homolce Hospital, Prague, Czechia; ^8^Department of Neurology, Jihlava Hospital, Jihlava, Czechia; ^9^Department of Neurology, Krajska zdravotni – Hospital Chomutov, Chomutov, Czechia, ^10^Cerebrovascular Research Program, International Clinical Research Center, Brno, Czechia

**Background and aims:** In acute ischemic stroke (AIS) and acute cervical internal carotid artery (cICA) occlusion, intravenous thrombolysis (IVT) represents a standard treatment, and emergent carotid endarterectomy (CEA), used alone or in combination with IVT, is an experimental alternative. The aim was to assess the safety and efficacy of IVT, emergent CEA, and IVT+CEA in AIS patients with acute cICA occlusion.

**Methods:** In a retrospective, multicenter study, the IVT group consisted of 41 patients (26 males; median age 72 [60–79] years), the IVT+CEA group of 31 patients (26 males; median age 70 [63–77] years), and the CEA group of 61 patients (45 males; median age 68 [61–75] years). 3‐month outcomes were assessed using a modified Rankin Scale (mRS), with good outcomes defined as 0–3.

**Results:** The following results were observed in the IVT, IVT+CEA, and CEA groups: recanalization rate 17.1%, 77.4%, and 88.5%, resp.; good 3‐month clinical outcomes 56.1%, 74.2%, and 89.8%, resp.; 3‐month mortality 22.0%, 19.4%, and 6.8%, resp. (*p* < 0.05 in all cases for IVT vs. CEA comparison). The use of CEA alone was identified as an independent positive predictor of good 3‐month clinical outcomes (OR 5.185, 95% CI: 1.973–13.63; *p* = 0.0009), and an independent negative predictor of 3‐month mortality (OR 0.295, 95% CI: 0.07112–0.8754; *p* = 0.0329).

**Conclusion:** In this retrospective, multicenter study, using an emergent CEA alone in patients with AIS and acute cICA occlusion was associated with a higher recanalization rate and the achievement of good 3‐month clinical outcomes, and lower 3‐month mortality compared to IVT.

**Disclosure:** Disclosure: Roman Herzig: Supported by STROCZECH within the CZECRIN Large Research Infrastructure (No. LM2023049) funded by the state budget of the Czech Republic, Ministry of Health of the Czech Republic (Grant No. DRO—UHHK 00179906), and Charles University, Czech Republic (Cooperation Program, research area NEUR). Igor Guňka: Nothing to disclose. Svatopluk Ostrý: Nothing to disclose. Jiří Fiedler: Nothing to disclose. Vladimír Přibáň: Nothing to disclose. Martin Kovář: Nothing to disclose. Petr Štádler: Nothing to disclose. Ondřej Škoda: Nothing to disclose. Jiří Neumann: Nothing to disclose. Veronika Kunešová: Supported by STROCZECH within the CZECRIN Large Research Infrastructure (No. LM2023049) funded by the state budget of the Czech Republic.

## EPR‐133

### burden and distribution of cerebral microbleeds and stroke recurrence and prognosis in cerebral hemorrhage

#### R. Ma ^1^; J. Wang^2^; Y. Wei^3^; Q. Zhang^1^; J. Miao^4^; J. Niu^4^; X. Zheng^1^; X. Yang^1^; X. Wang^1^; X. Lv^1^; S. Wang^4^

##### ^1^Yan'an University, China; ^2^Guizhou Medical University, Guiyang, China; ^3^Baoji Central Hospital, China; ^4^Xianyang Hospital of Yan'an University, China

**Background and aims:** To investigate the relationship between the burden and distribution of cerebral microbleeds and the recurrence of ischemic and hemorrhagic stroke in survivors of spontaneous intracerebral hemorrhage (ICH) and their impact on prognosis.

**Methods:** Patients with first‐onset spontaneous ICH between January 1, 2019, and October 31, 2023, were consecutively enrolled from the stroke registry of Xianyang Hospital of Yan'an University. Patients were divided into a non‐severe CMBs group (< 10 microbleeds) and a severe CMBs group (≥10 microbleeds) based on magnetic susceptibility‐weighted imaging findings. Follow‐up assessments were conducted via telephone for a period of 12 months post‐discharge. The primary outcomes were recurrent stroke and type of recurrent stroke, while the secondary outcome was long‐term (1‐year) functional prognosis. Cox proportional hazards models were used to assess the risk of stroke recurrence. Logistic regression analysis was used to determine the independent association between CMBs and poor functional prognosis 1 year after cerebral hemorrhage.

**Results:** Among the 412 patients enrolled, 278 had detectable CMBs, including 80 in the severe CMBs and 198 in the non‐severe CMBS, with 22 cerebral hemorrhages and 35 cerebral infarctions occurring during a median follow‐up of 28.5 months. Severe CMBs burden was associated with recurrent hemorrhagic stroke (HR = 4.592, 95% Cl: 1.943–10.848) and was an independent risk factor for poor prognosis at 1 year (OR = 2.190, 95% Cl: 1.205–3.982) as well.

**Figure:**
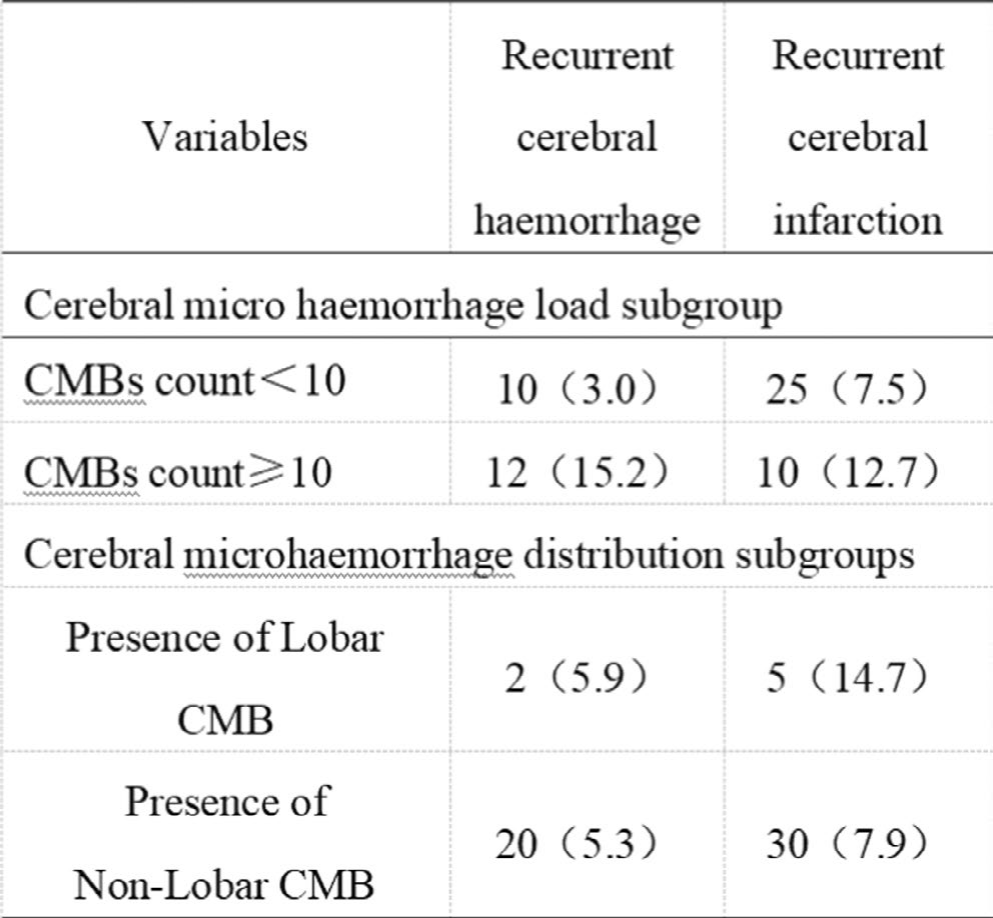
**TABLE 1** Endpoint events during follow‐up in subgroups with different burden and distribution of cerebral microhemorrhage.

**Figure:**
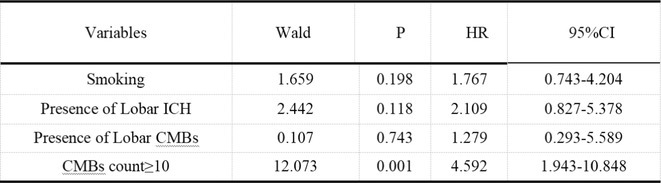
**TABLE 2** Multivariate cox proportional‐hazards model of hemorrhagic stroke recurrent

**Figure:**
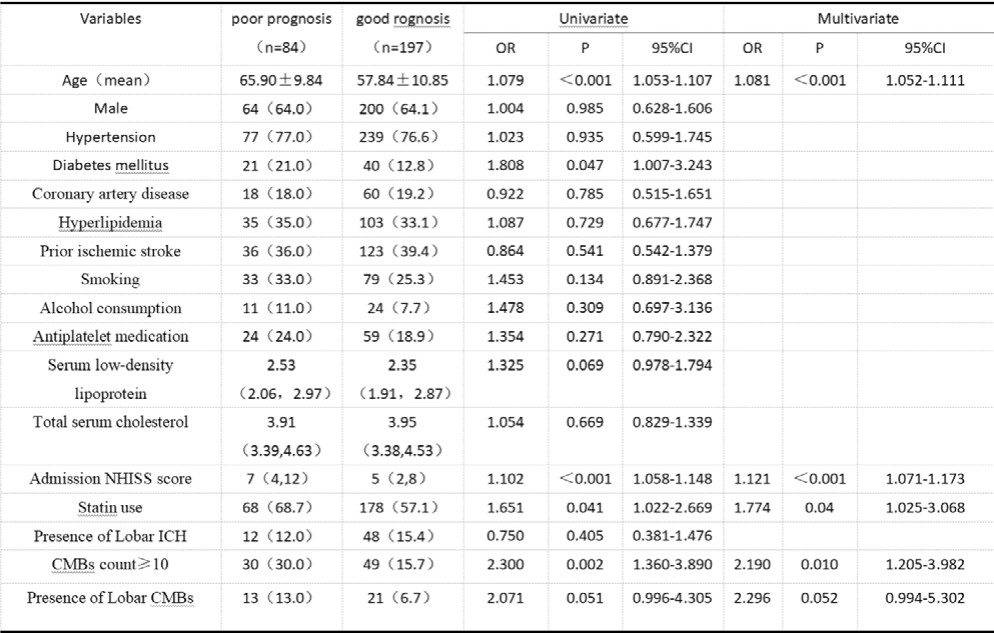
**TABLE 3** Univariate and multivariate logistic regression analysis affecting prognosis

**Conclusion:** Our findings indicate that patients with severe CMBs have a high risk of recurrent hemorrhagic stroke following ICH and a poor prognosis.

**Disclosure:** Nothing to disclose.

## Child neurology/developmental neurology

## EPR‐134

### Lentiviral hematopoietic stem cell gene therapy (atidarsagene autotemcel) for late juvenile metachromatic leukodystrophy

#### V. Calbi^1^; V. Gallo^2^; S. Mazza^1^; A. Zambon^3^; M. Bernardo^7^; S. Recupero^2^; B. Volpe^7^; S. Majidi‐Fard^7^; L. Del Giacco^7^; E. Inverso^7^; F. Ciotti^2^; M. Fraschini^2^; M. Sarzana^2^; C. Puricelli^2^; M. Sangalli^2^; A. Clerici^1^; F. Morena^4^; S. Martino^4^; J. Brooks^5^; P. Nutkins^5^; A. Shenker^6^; N. Gollop^5^; L. Campbell^5^; A. Aiuti^7^; F. Fumagalli ^1^

##### ^1^San Raffaele Telethon Institute for Gene Therapy (SR‐TIGET), IRCCS San Raffaele Scientific Institute, Milan, Italy; ^2^Pediatric Immunohematology Unit and BMT Program, IRCCS San Raffaele Scientific Institute, Milan, Italy; ^3^Neurology & Neurophysiology Unit, IRCCS San Raffaele Scientific Institute, Milan, Italy; ^4^Department of Chemistry, Biology and Biotechnologies, University of Perugia, Perugia, Italy; ^5^Orchard Therapeutics, London, UK; ^6^Clinical Consultant, Pennington, NJ, USA.; ^7^Vita‐Salute San Raffaele University, Milan, Italy

**Background and aims:** Metachromatic leukodystrophy (MLD) is a rare lysosomal disease due to arylsulfatase A (ARSA) deficiency. Atidarsagene autotemcel (arsa‐cel), an ex‐vivo hematopoietic stem cell gene therapy, has shown favorable outcomes in early‐onset MLD. A phase III trial (NCT04283227) evaluates arsa‐cel in pre‐symptomatic or early‐symptomatic Late Juvenile MLD (LJ‐MLD) patients. This report presents interim engraftment, pharmacodynamic and safety data.

**Methods:** Six patients (4 pre‐symptomatic, 2 early‐symptomatic) were treated. Median age at treatment was 10.4 years (range: 2.7‐15.5 years). Stem cell source was mobilized peripheral blood. The infused drug product median dose was 23.1×10^6 CD34+ cells/kg (range: 16.0‐28.9×10^6 CD34+ cells/kg), with a vector copy number (VCN)/cell range of 2‐5.

**Results:** Median follow‐up was 27.8 months (range: 12.6 to 34.3 months). All patients are alive. Five remained neurologically stable; one early‐symptomatic patient experienced disease progression, followed by stabilization up to 30 months post‐treatment. Short‐term adverse events were consistent with busulfan's safety profile. No malignancies, clonal expansion, or immune responses against ARSA occurred. All treated patients showed rapid engraftment of transduced cells. At 1‐year post‐treatment, five patients with available data showed median VCN in peripheral blood mononuclear cells (PBMCs) of 0.3/cell (range 0.2‐0.9 ) with a transduction rate in bone marrow progenitor cells ranging from 18.8% to 85.4%. ARSA activity reached supranormal levels in PBMCs and normal levels in cerebrospinal fluid at latest follow‐up.

**Conclusion:** Early results with arsa‐cel show safety and pharmacodynamics efficacy in LJ‐MLD, consistent with those seen in early‐onset MLD. Continued monitoring will assess clinical endpoints and long‐term safety.

**Disclosure:** Valeria Calbi, Francesca Fumagalli, Vera Gallo have occasionally received consultant fees and reimbursement for travel costs and participation fees from Orchard Therapeutics. JB, PN, NDG, and LC are current employees of Orchard Therapeutics. AS is a former employee of GSK and clinical consultant for Orchard Therapeutics. Atidarsagene autotemcel was licensed to GlaxoSmithKline (GSK) in 2014 and GSK became the clinical trial sponsor. In 2018 MLD development rights were transferred to Orchard Therapeutics (OTL) and OTL became the clinical trial sponsor

## EPR‐135

### Long‐term medical and social outcomes in children and adolescents with Lennox Gastaut syndrome

#### I. Ray Chaudhuri ^1^; V. KP^1^; V. Anand^1^; B. Raghavan^2^

##### ^1^Department of Paediatric Neurology, Amrita Institute of Medical Sciences, Kochi, India; ^2^Department of Clinical Psychology, Amrita Institute of Medical Sciences, Kochi, India

**Background and aims:** Lennox Gastaut syndrome (LGS) is a severe, chronic epilepsy that begins in childhood and continues into adulthood, causing persistent seizures and neurocognitive impairment. This study aimed to assess epilepsy, neurodevelopmental outcomes, and quality of life (QoL) in individuals with LGS and identify predictors of these outcomes.

**Methods:** A cross‐sectional evaluation was conducted at a tertiary‐care center in South India between June 2023 and March 2024. The cohort included 96 patients (aged 2‐25 years, diagnosed before 18), assessed for epilepsy severity, motor functional status, social quotient, behavioral comorbidities, quality of life, and caregiver burden using various standardized scales.

**Results:** The study involved 96 participants (63.5% boys) with at least 1‐year of regular follow‐up. Mean (+ S.D.) age of the cohort was 10.3 (5.8) years. 56.3% had a structural etiology. Long‐term outcomes showed ongoing epilepsy in 73 patients, unfavorable motor status in 77, moderate to severe socio‐adaptive deficits in 68, autism spectrum disorder in 47, ADHD in 39, impaired QoL in 78, and significant caregiver burden in 79. Generalized paroxysmal fast activity on inter‐ictal EEG was associated with ongoing epilepsy (*p* = 0.01). Better epilepsy control was linked to improved motor and cognitive outcomes.

**Conclusion:** The study revealed a predominance of structural etiology, with ongoing epilepsy and significant impacts on QoL and caregiver burden in more than two‐third of patients in last follow‐up. With improved survival rates in children and adolescents with LGS, better epilepsy control could lead to improved neurocognitive outcomes, enhanced QoL, and reduced caregiver burden.

**Disclosure:** Nothing to disclose.

## EPR‐136

### Long‐term 24‐month findings of N‐acetyl‐L‐leucine for Niemann‐Pick disease type C

#### M. Strupp ^1^; M. Patterson^2^; J. Raymond^2^; B. Zanrucha^2^; A. Hatcher^2^; T. Fields^2^; T. Bremova‐Ertl^3^; K. Martakis^4^

##### ^1^Department of Neurology, Hospital of the Ludwig Maximilians University, Munich, Germany; ^2^IntraBio; ^3^Department of Neurology and Center for Rare Diseases, University Hospital Inselspital Bern, Switzerland; ^4^Department of Neuropediatrics, Justus Liebig University, Giessen, Germany

**Background and aims:** The IB1001‐ 301 clinical trial was a Phase III, double‐blind, randomized, placebo‐controlled trial comparing N‐acetyl‐L‐leucine (NALL) with placebo for the treatment of neurological signs and symptoms in Niemann‐ Pick disease type C (NPC) after 12 weeks. The primary Scale for the Assessment and Rating of Ataxia (SARA) endpoint was reduced −1.97 points with NALL and −0.60 with placebo (*p* < 0.001). Extended follow‐up data were obtained in an open‐label Extension Phase (EP) to evaluate the long‐term, neuroprotective effects of NALL for NPC.

**Methods:** Patients received treatment with orally administered NALL 2–3 times per day (patients 4‐ 12 years receiving weight‐based doses (2 to 4 g per day), those ≥ 13 years 4 g per day). The primary endpoint was the modified 5‐domain NPC Clinical Severity Scale (5‐Domain NPC‐ CSS) (range 0–25 points; lower score representing better neurological status). Comparisons were made to the expected annual trajectory of disease decline established in published natural history studies. Exploratory endpoints included the 15‐domain NPC‐ CSS (excluding hearing) and SARA.

**Results:** 54 patients aged 5–67 years were treated in the EP. After 24 months, the mean ( ± SD) change from baseline on the 5‐domain NPC‐CSS was ‐0.24 ( ± 2.69) on NALL, compared to +3.0 ( ± 6.32) in the historical cohort: mean difference ‐3.24 (95% Confidence Interval (CI) ‐5.59 to ‐0.89; *p* = 0.009). The result of the 15‐domain NPC‐ CSS was supportive of the primary analysis and the improvements in neurological status demonstrated in the Parent Study's primary SARA endpoint were sustained over the 24‐month long‐term follow‐up.

**Conclusion:** Treatment with NALL after 24 months was associated with a statistically significant and clinically meaningful reduction in disease progression and consistent with a neuroprotective, disease‐modifying effect.

**Disclosure:** Marc Patterson, Janelle Raymond, Beth Zanrucha, and Asante Hatcher are all employees of IntraBio the study sponsor. Tatiana Bremova‐ Ertl received speaker's honoraria and consultancy fees from Actelion, Sanofi‐ Genzyme and Zevra as well as blinded video‐ rater fees from Intrabio.

## EPR‐137

### Quantitative sleep EEG biomarkers in Rett Syndrome: Sleep as a window to understand synaptic dysfunction

#### M. Cataldi ^1^; R. Cordani^1^; S. Sarasso^2^; M. Colombo^2^; L. Chiarella^3^; M. Veneruso^3^; L. Di Tullio^3^; S. Boeri^3^; G. Prato^1^; A. Barbieri^3^; R. Ferri^4^; L. Nobili^3^

##### ^1^Child Neuropsychiatry Unit, IRCCS Istituto Giannina Gaslini, Genoa, Italy; ^2^Department of Biomedical and Clinical Sciences, Università degli Studi di Milano, Milan, Italy.; ^3^Department of Neurosciences, Rehabilitation, Ophthalmology, Genetics, and Maternal and Child Health (DINOGMI), University of Genoa, Genoa, Italy; ^4^Sleep Research Center, Department of Neurology I.C, Oasi Research Institute ‐ IRCCS, Troina, Italy

**Background and aims:** Rett Syndrome (RTT) is a neurodevelopmental disorder caused by mutations in the MECP2 gene, with sleep disturbances affecting ~80% of individuals. Dysregulation of thalamocortical connectivity and synaptic dysfunction in RTT may contribute to abnormalities in sleep spindles and slow waves, as observed in other neurological disorders. This study investigates the role of quantitative sleep EEG analysis in uncovering neural circuit abnormalities in RTT, comparing findings with MECP2‐deficient animal models.

**Methods:** We enrolled 14 females with typical RTT and MECP2 mutations, alongside age‐matched controls. Using overnight polysomnography and quantitative EEG analysis, we assessed spindle density and slow‐wave parameters (power, slope, amplitude) via custom MATLAB scripts. Spearman's correlation analysis was applied for clinical insights, and Wilcoxon tests compared RTT patients with controls.

**Figure:**
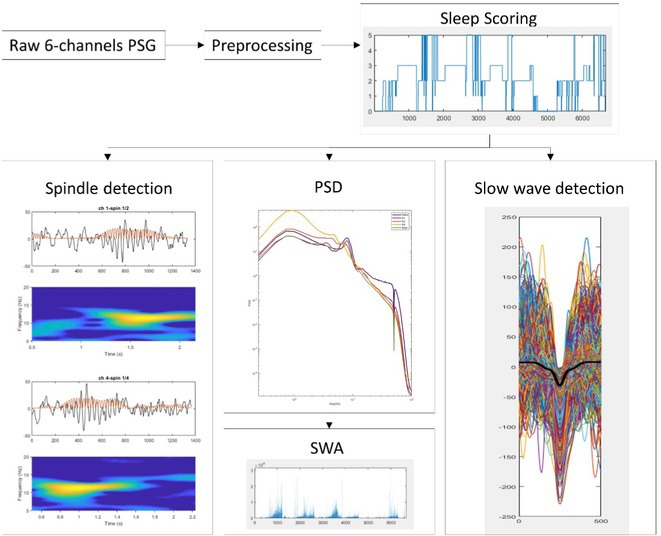
**FIGURE 1** Pipeline of analysis

**Results:** Our analysis identified two key findings. First, spindle density (spindles per minute) was significantly reduced in RTT patients compared to controls. Second, slow‐wave parameters exhibited a diminished nocturnal decrease in RTT patients, suggesting disrupted sleep‐dependent synaptic homeostasis and impaired cortical synaptic plasticity.

**Figure:**
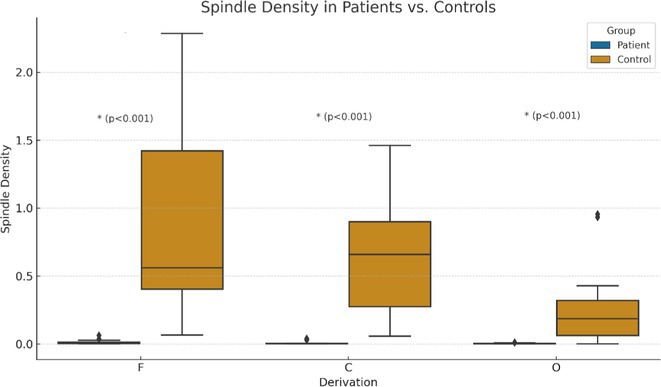
**FIGURE 2** Spindle density, patients versus controls

**Figure:**
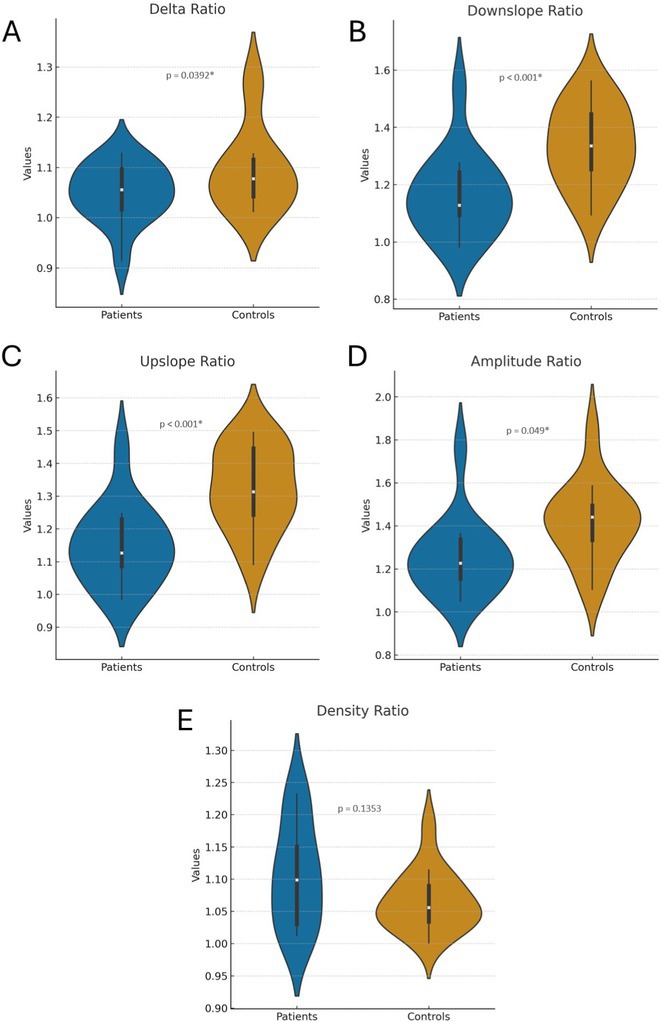
**FIGURE 3** Delta Power and Slow Waves parameters ratios between 1st and 2nd half of NREM

**Conclusion:** This study demonstrates decreased spindle density and altered slow‐wave parameters in RTT patients, consistent with findings in animal models. These electrophysiological biomarkers provide insights into sleep‐dependent synaptic dysfunctions and neural circuit abnormalities in RTT. Further exploration of quantitative sleep EEG biomarkers may offer valuable prognostic and therapeutic opportunities for RTT.

**Disclosure:** Nothing to disclose.

## EPR‐138

### Investigating the neuroprotective effects of MgSO4 in children at risk of preterm birth: Meta‐analysis of 20109 infants

#### O. Alomari^1^; M. Mokresh^1^; D. Aslan ^1^; C. Caliskan^1^; G. Ghahremanpour^1^; E. Ari^1^; M. Kammaz^1^; E. Kahraman^1^; N. Kömürcü^1^; E. Yazicilar^1^; H. Eyvazova^1^; G. Dizdarogulları^2^

##### ^1^Hamidiye International School of Medicine, University of Health Sciences, Istanbul Turkey; ^2^Department of Perinatology, University of Health Sciences Turkey, Kartal Dr. Lutfi Kirdar Training and Research Hospital, Istanbul, Turkey

**Background and aims:** Preterm birth, which is defined as the delivery of babies before 37 gestational weeks are completed, is still a serious global‐health issue. One of the major challenges among preterm birth infants is the prevalence of neurological disorders. In order to prevent the complications related to preterm birth, many interventions and agents have been utilized such as magnesium sulfate (MgSO4). The aim of this study was to investigate the neuroprotective effects of MgSO4 in children at risk of premature birth.

**Methods:** PubMed, WOS, Scopus, and Embase databases have been searched for relevant articles. The main outcomes assessed were cerebral palsy (CP), and any other available data. The random effects model was utilized to estimate odds ratio (OR).

**Results:** 55 studies involving more than 20,000 preterm infants were included. The analysis performed on the MgSO4 effectiveness on CP showed an OR suggesting a reduced risk of CP compared to the placebo group [OR = 0.74, 95% CI = (0.64; 0.86), I2 = 16%] (Fig. 1). The analysis performed on the effectiveness on preventing intraventricular hemorrhage showed an OR suggesting a reduced risk of CP compared to the placebo group [OR = 0.85, 95% CI = (0.76; 0.94), I2 = 66%] (Fig. 2). Side effects thought to be associated with MgSO4 such as necrotizing enterocolitis and patent ductus arteriosus didn’t show any significant difference between MgSO4 and placebo groups [OR = 1.08, 95% CI = (0.88; 1.32), I2 = 51%; OR = 1.11,95% CI = (0.83; 1.48), I2 = 5%; respectively] (Fig. 3).

**Figure:**
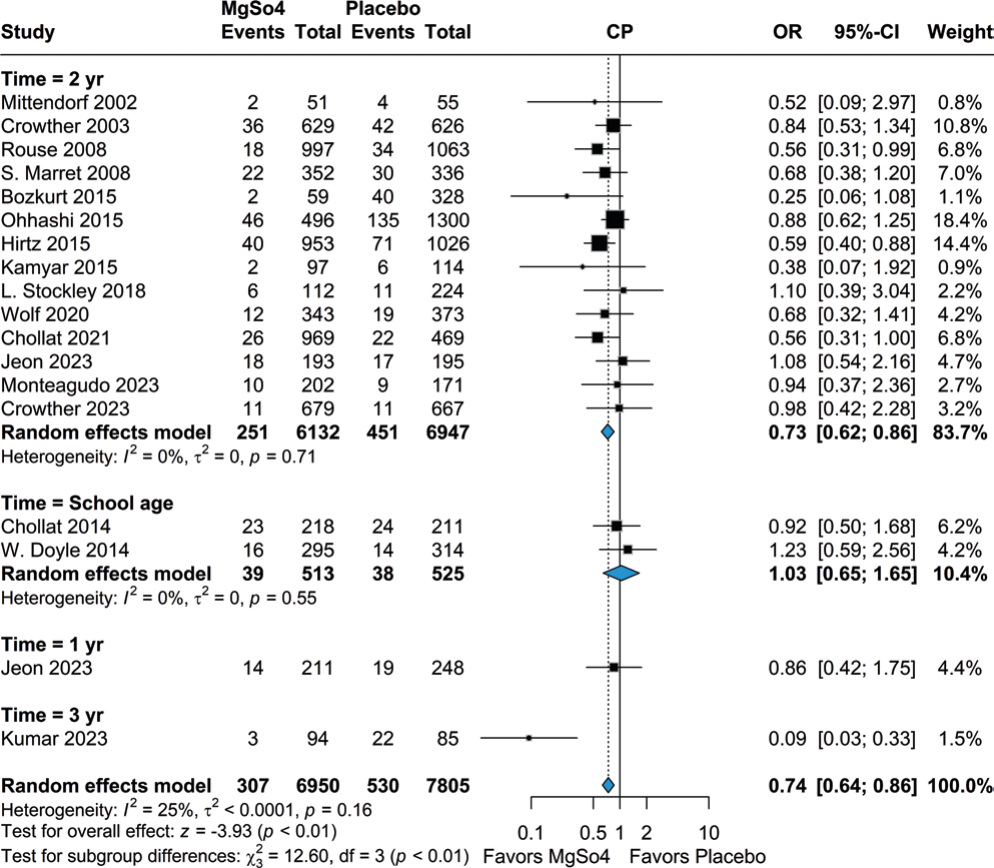
**FIGURE 1** Effectiveness of magnesium sulfate in reducing cerebral palsy risk in preterm infants.

**Figure:**
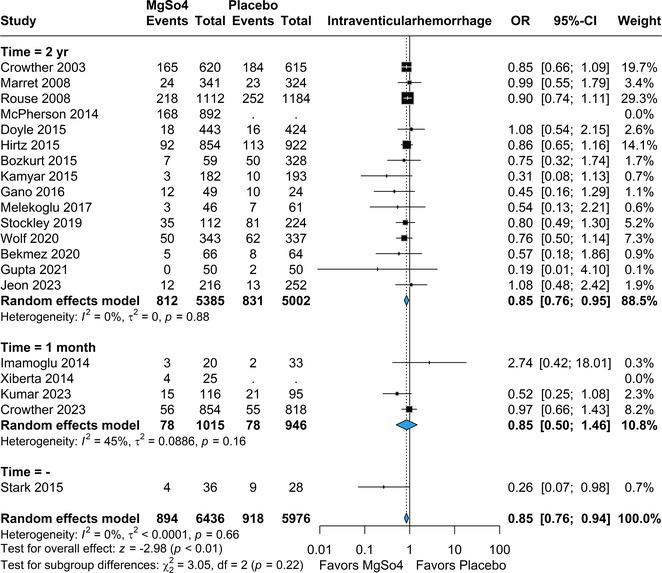
**FIGURE 2** Effectiveness of Magnesium Sulfate in Preventing Intraventricular Hemorrhage in Preterm Infants.

**Figure:**
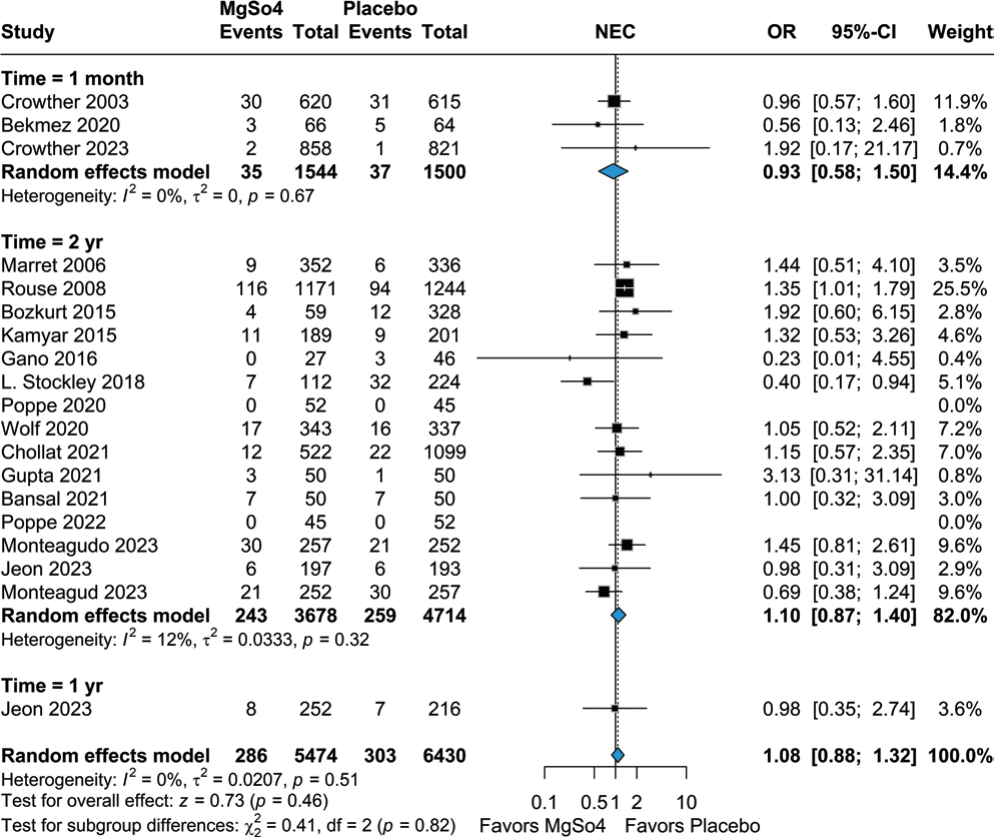
**FIGURE 3** Comparison of necrotizing enterocolitis (NEC) and patent ductus arteriosus (PDA) rates among included preterm infants with or without MgSO4 administration.

**Conclusion:** MgSO4 has a neuroprotective potential and is recommended to be used for preterm infants.

**Disclosure:** Nothing to disclose.

## EPR‐139

### Exploring the associations of lesion metrics and neurological functions on cognitive outcomes in pediatric stroke

#### S. Salzmann ^1^; L. Steiner^1^; T. Aprasidze^1^; A. Klein^1^; G. Oesch^1^; H. Arsany^2^; M. Steinlin^1^; R. Everts^1^

##### ^1^Division of Neuropediatrics, Development and Rehabilitation, Department of Pediatrics, Inselspital, Bern University Hospital, University of Bern, Bern, Switzerland; ^2^University Institute of diagnostic and interventional neuroradiology, Inselspital, Bern University Hospital, Bern, Switzerland

**Background and aims:** The prevalence of cognitive problems after pediatric stroke is high and various risk factors such as stroke‐related variables, demographic, neurological and cognitive factors may affect the cognitive long‐term outcome. Whether and how these risk factors are associated with cognitive outcome is still incompletely understood. This study investigated how lesion volume, lesion location and neurological functions at discharge, 6‐month and 2‐year after pediatric stroke contribute to long‐term cognitive outcome.

**Methods:** This observational study included patients after pediatric arterial ischemic stroke. Cognitive long‐term outcome (intelligence, processing speed, working memory) was assessed at least one year after stroke. Neurological functions were measured using the pediatric stroke outcome measure at discharge, at 6‐month and 2‐year follow‐up. Magnetic resonance imaging at stroke manifestation was applied to determine acute to subacute lesion metrics (volume & location).

**Results:** 43 patients aged 6 to 23 years were enrolled in the study. Cognitive functions were significantly associated with lesion volume and lesion location. Processing speed and working memory were worse if the left caudate nucleus was involved. Further, cognitive long‐term outcome correlated significantly with neurological functions at discharge, 6‐month and 2‐year follow‐up.

**Conclusion:** Cognitive long‐term outcome was associated with lesion volume, lesion location and neurological functions. Our study adds to the determination of risk factors for cognitive long‐term rehabilitation and highlights the need for the combined evaluation of neurological and cognitive performance.

**Disclosure:** Nothing to disclose.

## EPR‐140

### AI in pediatric TBI outcome prediction: A systematic review

#### S. Rajendra ^1^; A. Sharma^2^

##### ^1^The University of Buckingham, Buckingham, UK; ^2^The University of Buckingham, Buckingham, UK

**Background and aims:** Pediatric TBIs is a leading cause of mortality and long term disabilities in children. Approximately half a million children go into American hospitals every year due to a TBI, showing the extent to which this is an issue within the medical world. In order to prevent the more dangerous outcomes it is crucial that there is early intervention along with tailored care given how sensitive the system is. AI can provide an easier solution to this ‐ it can analyze the complex personalized datasets and provide accurate prognostic insights. This would reinforce or possibly show better ways to treat pediatric patients with TBIs and prevent harmful outcome.

**Methods:** A systematic search of PubMed, Embase, and Scopus databases was conducted for studies published in the last 10 years. Inclusion criteria were original peer‐reviewed research focusing on pediatric TBIs, utilizing AI for outcome predictions, and reporting performance metrics. Articles were screened, and data on AI model type, accuracy (e.g., AUC, sensitivity, specificity), and clinical applicability were extracted.

**Figure:**
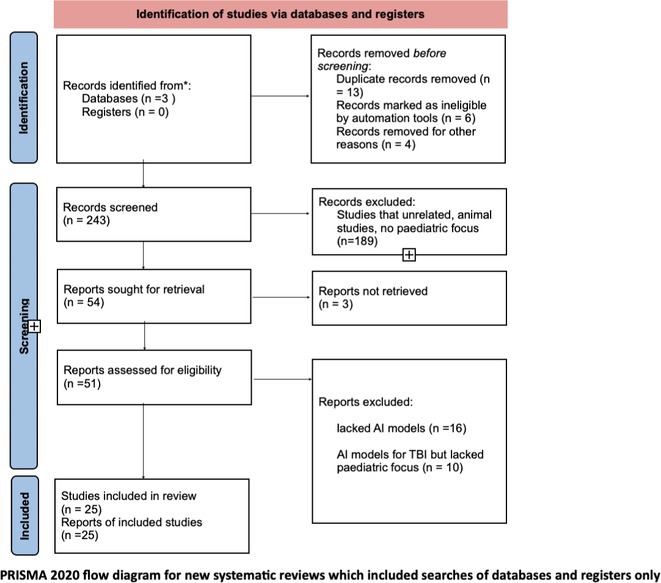
FIGURE 1

**Results:** Of 243 initial articles, 25 met the inclusion criteria. Most studies used machine learning algorithms, with several incorporating imaging and clinical data. Predictive accuracy for long‐term outcomes ranged from AUC 0.80 to 0.93. However, limitations included small, heterogeneous datasets and a lack of external validation. Few studies focused specifically on pediatric populations, limiting generalizability.

**Conclusion:** AI demonstrates potential in predicting outcomes in pediatric TBI but requires further validation. Developing standardized pediatric datasets and improving model transparency could enhance clinical adoption

**Disclosure:** Nothing to disclose.

## EPR‐141

### Neuroendoscopic lavage for the early management of posthemorrhagic hydrocephalus in neonates: A meta‐analysis

#### W. Fagundes^1^; L. Tamara Alves Carretta^2^; J. Kotochinsky^3^; R. Reis de Oliveira^4^; I. Simon Petry^5^; Y. Picanço Silva ^6^; P. Łajczak^7^; E. Sharma^8^; R. Fonseca Oliveira Suruagy Motta^9^; L. Silva Almeida^10^

##### ^1^Division of Neurosurgery, Federal University of Espírito Santo, Vitória, Espírito Santo, Brazil; ^2^Department of Medicine, Escola Superior de Ciências da Santa Casa de Misericórdia de Vitória (EMESCAM), Vitória, Brazil; ^3^Faculty of Medicine, National University of Cuyo, Argentina; ^4^Department of Medicine, Federal University of Pará, Belém, Pará, Brazil; ^5^Department of Medicine, University of Southern Santa Catarina, Palhoça, Santa Catarina, Brazil; ^6^Department of Internal Medicine, Healthcare Institution of South Iceland, Selfoss, Iceland; ^7^Faculty of Medical Sciences in Zabrze, Medical University of Silesia, Katowice, Poland; ^8^David Geffen School of Medicine at UCLA, Los Angeles, USA; ^9^Cesmac University Center, Maceió, Brazil, ^10^Department of Medicine, Catholic University of Brasília, Brasília, Brazil

**Background and aims:** Posthemorrhagic hydrocephalus (PHH) is a significant complication of neonatal intraventricular hemorrhage (IVH). Neuroendoscopic lavage (NEL) is an emerging treatment option, but its efficacy compared to traditional management remains unclear. We aimed to access the available evidence on the effectiveness and safety of NEL versus traditional management for the early treatment of PHH in neonates.

**Methods:** A comprehensive search of electronic databases (PubMed, Embase, Google Scholar, and Web of Science) was conducted to identify studies exploring the effectiveness of NEL. The analysis was performed using random‐effects models. The primary outcome was ventriculoperitoneal (VP) shunt dependency. Secondary outcomes included infection rates, multiloculated hydrocephalus, and mortality.

**Results:** Seven studies involving 237 neonates met the inclusion criteria. The pooled analysis revealed a shunt dependency rate of 50% (95% CI: 35.34‐65.02%) with significant heterogeneity (I^2^ = 83%). The infection rate was 11.12% (95% CI: 5.73‐16.52%), while the incidence of multiloculated hydrocephalus was 6% (95% CI: 0.52‐11.52%). Mortality showed a low rate of 4.38% (95% CI: 1.44‐7.32%). These findings suggest that while NEL is associated with promising safety outcomes and low VP shunt dependency, the variability found underscores the need for standardization and further research.

**Figure:**
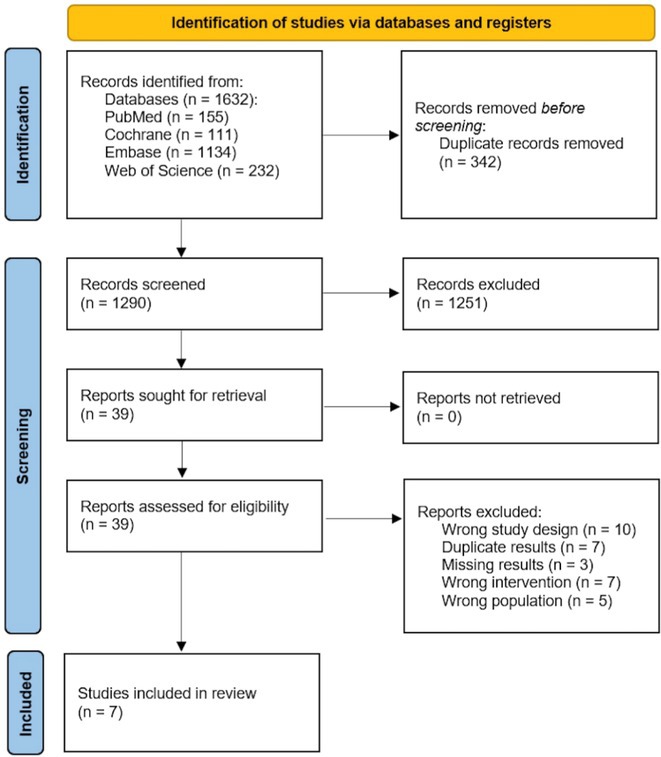
**FIGURE 1** Forest plots of results.

**Conclusion:** This meta‐analysis of NEL for neonatal PHH found low shunt dependency rates, moderate infection rates, and low mortality. Significant variability was observed across studies. Further research, including well‐designed randomized controlled trials, is needed to determine the true effectiveness and safety of NEL compared to traditional treatments.

**Disclosure:** Nothing to disclose.

## EPR‐142

### Institutional cohort characteristics of patients with AVMs

#### D. Zețu‐Buciușcan; O. Grosu; I. Preguza

##### Neurology Department. Diomid Gherman Institute of Neurology and Neurosurgery. Moldova, Chisinau.

**Background and aims:** Arteriovenous malformations (AVM) can develop within the brain or spine, with hemorrhage representing their most significant potential risk.

**Methods:** a retrospective evaluation of electronic medical records was conducted at the Diomid Gherman Institute of Neurology and Neurosurgery, from 2017 to 2024. Data were extracted using ICD‐10 codes, followed by manual review.

**Results:** The cohort included 76 patients, with a mean age of 43.7  ± 14.56 years; 51.3% were male. The most common clinical manifestations were headache (89.7%), neurological deficits (48.6%), convulsive seizures (35.5%), general weakness (34.2%), consciousness disturbances (32.8%), vertigo (32.8%), speech disorders (25%), visual disturbances (14.4%), cranial nerve palsies (13.1%), dizziness and nausea (12%), cerebellar syndrome (11.8%), and disease‐related mortality (5.2%). Of the AVMs, 95.5% were cerebral and 4.5% were spinal. Diagnostic methods utilized included digital subtraction angiography (91.2%), computed tomography angiography (75%), cerebral/spinal MRI (57.3%), and plain computed tomography (42.6%). Complications were 39.47% ‐ hemorrhagic stroke and 4.11% ischemic stroke. Surgical approaches were tailored individually, with a mean of 1.76  ± 1.48 interventions per patient (range: 0–7). Embolization emerged as the most frequently employed treatment following its implementation at the institution.

**Conclusion:** This cohort represents the largest series of AVM patients in the country. The clinical characteristics align with international findings, and patients were diagnosed and treated using state‐of‐the‐art techniques. These results underscore the importance of a multidisciplinary approach in managing AVM cases.

**Disclosure:** Institutional research grant.

## Headache 2

## EPR‐143

### Treatment pattern and reasons for discontinuing preventive treatment in cluster headache. A prospective cohort study

#### A. Petersen; M. Søborg; R. Jensen; N. Lund

##### Danish Headache Center, Department of Neurology, University of Copenhagen, Rigshospitalet‐Glostrup, Glostrup, Denmark

**Background and aims:** Pharmacological prevention of cluster headache aimed to reduce attack frequency and intensity, but its long‐term effectiveness and tolerability remain unknown. Despite recent innovations for mi‐graine, treatments for cluster headache have seen little progress since the 1990s. We aimed to quantify the discontinuation rates of verapamil and other prevention due to side effects and assess the proportion of verapamil responders who maintain effectiveness at follow‐up.

**Methods:** In total, 596 patients with cluster headache according to the ICHD‐criteria completed a semi‐structured interview between 2017 and 2023, with 430 being followed up for another interview after a median of 4.6 years (IQR: 2.8).

**Results:** Of the 457 patients (77%) who have tried prevention, 121 (26%) discontinued due to intolerable side effects. Discontinuation rate for the first‐line treatment, Verapamil, was 18%. Proportionally, second‐line treatments lithium and topiramate had higher discontinuation rates, with cessation due to tolerability in nearly half of the participants. The odds of discontinuing lithium or topiramate were four times higher than for verapamil (*p* < 0.0001). At follow‐up, only 33% were on a current prevention. Of the initial 137 verapamil responders, 76% continued use of verapamil or discontinued due to remission. Only half of these were 50%‐responders. 23% had discontinued verapamil due to side effects, with fatigue and dizziness most frequently reported.

**Figure:**
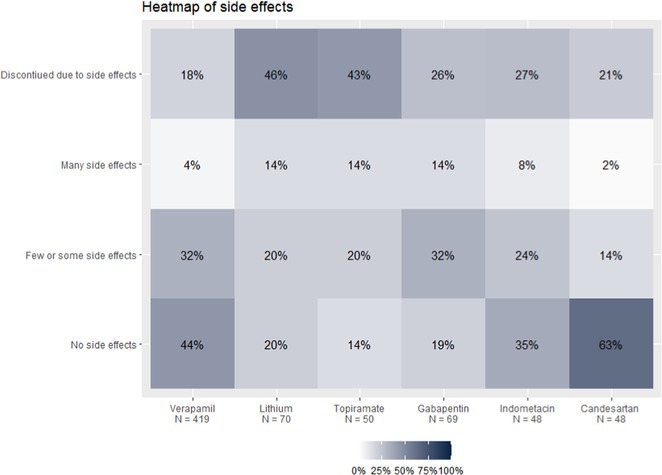
**FIGURE 1** Heatmap of discontinuation rates

**Conclusion:** Effective therapeutic prevention of cluster headache is hindered by intolerability and insufficient efficacy. Verapamil remains effective for most initial responders over time, but our findings under‐score the need for more tolerable and effective longterm preventive treatments for cluster headache.

**Disclosure:** This study was funded by H. Lundbeck A/S but had no influence on design or execution of the study nor drafting of the abstract.

## EPR‐144

### Impact of atogepant on achieving best possible quality of life among patient global impression of change responders

#### C. Tassorelli ^1^; P. Gandhi^2^; M. Duan^2^; K. Carr^2^; K. Umashankar^2^; J. Stokes^2^; G. Forde^3^; T. Bilchik^4^; R. Halker Singh^5^

##### ^1^University of Pavia, Pavia, Italy; ^2^AbbVie, North Chicago, IL, USA; ^3^North American Partners in Pain Management, North New Hyde Park, NY, USA; ^4^Department of Neurology, Yale School of Medicine, New Haven, CT, USA; ^5^Mayo Clinic, Phoenix, AZ, USA

**Background and aims:** We evaluated the impact of atogepant, an oral calcitonin gene‐related peptide receptor antagonist used for migraine prevention, on participants reporting best possible quality of life (QoL) based on Migraine‐Specific Quality of Life Questionnaire among responders and non‐responders to Patient Global Impression of Change (PGIC).

**Methods:** ADVANCE, ELEVATE, and PROGRESS were phase 3, multicenter, randomized, double‐blind, placebo‐controlled, 12‐week trials that included adults with episodic migraine (EM), EM with prior inadequate responses to 2‐4 oral preventive treatments, and chronic migraine (CM), respectively. This post hoc analysis evaluated the proportion of participants who achieved a score of 100 for all 3 MSQv2.1 domain scores at Week 12 based on their PGIC response (responders and non‐responders). PGIC is a 7‐point single question scale measuring participant impression of change in migraine symptoms since first dose of treatment. MSQv2.1 is a 3‐domain questionnaire composed of 14 items designed to assess how migraine limits social and work activities, prevents social and work activities, and what emotions are associated with migraine. A score of 100 indicates the best possible QoL, with less disruption from migraine.

**Results:** In PGIC responders, a higher proportion of EM participants treated with atogepant 60mg once daily achieved MSQv2.1 scores of 100 in all 3 domains compared with placebo [ADVANCE(*p* < = .05); ELEVATE(*p* < = .001)(Figure 1,2)], and numerically higher proportion in CM atogepant‐treated participants [PROGRESS(*p* > = .05)(Figure 3)].

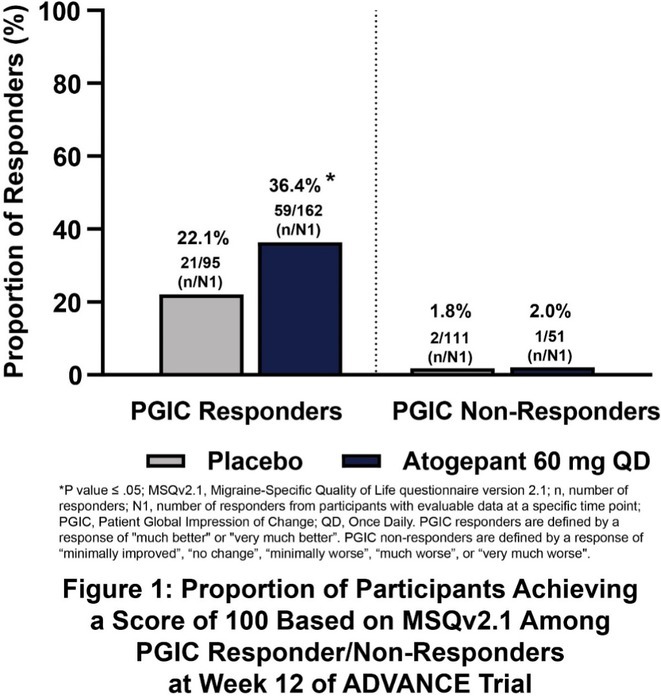

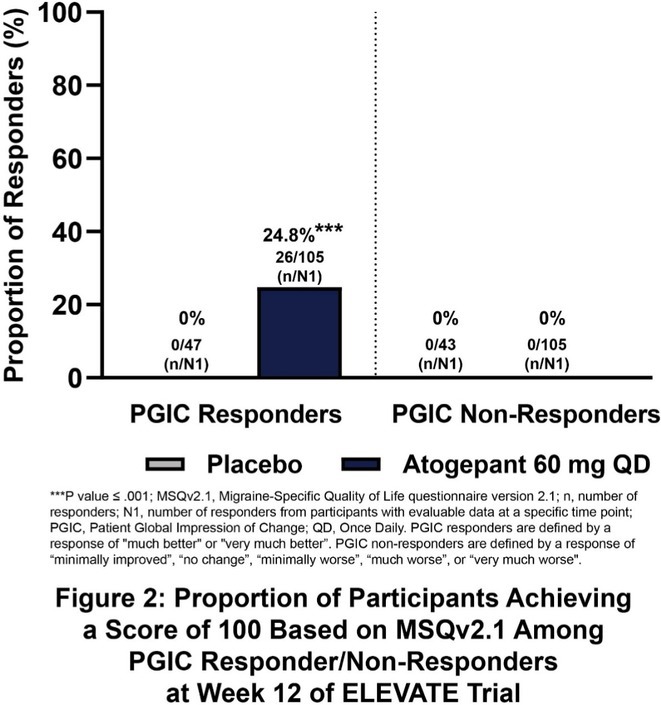

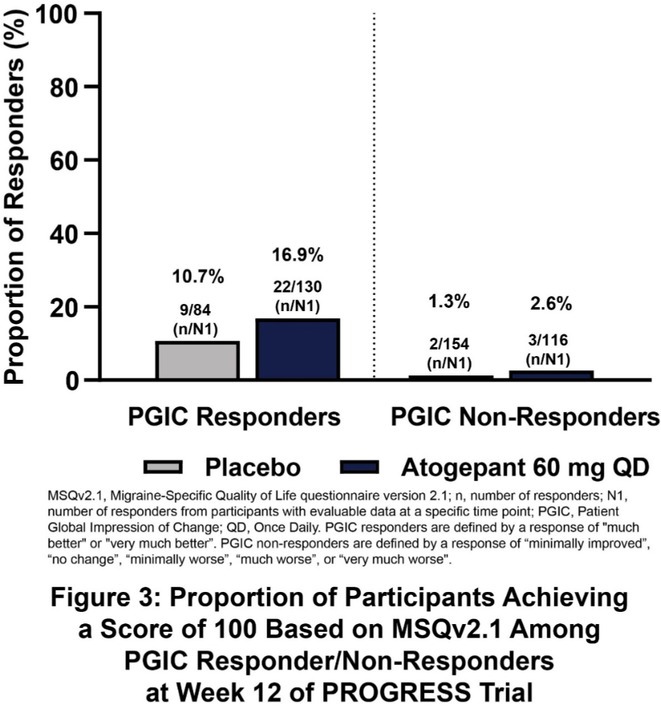

**Conclusion:** A greater proportion of atogepant‐treated EM and CM participants who are PGIC responders reported best possible QoL with lower disruption from migraine, compared with placebo.

**Disclosure:** Cristina Tassorelli has participated in advisory boards or has lectured for AbbVie, Dompé, Eli Lilly, Ipsen, Lundbeck, Medscape, Pfizer and Teva. She is principal investigator or collaborator in clinical trials sponsored by AbbVie, Eli Lilly, Ipsen, Lundbeck, Pfizer and Teva. She has received research grants from the European Commission, the Italian Ministry of Health, the Italian Ministry of University, the Migraine Research Foundation, and the Italian Multiple Sclerosis Foundation. Grace Forde has received personal compensation from AbbVie. Rashmi Halker Singh has served as a consultant for AbbVie, Impel, Pfizer, Supernus, and Teva. She has provided editorial services for Current Neurology and Neuroscience Reports, Headache, and Medscape, and has received grants for research support from Amgen, Eli Lilly, and Gore Pharmaceuticals. Tanya Bilchik has participated in an advisory board for AbbVie. She is a clinical trial investigator for AbbVie. PG, KC, MD, KU, and JS are AbbVie employees and may hold AbbVie stock.

## EPR‐145

### BIOmarkers of MIGraine response to erenumAb (BIOMIGA): Preliminary assessment of clinical and biomolecular markers

#### D. Martinelli ^1^; E. Caronna^2^; H. Basedau^3^; M. Pocora^4^; E. Ballante^1^; R. Greco^1^; V. Gallardo López^2^; L. Asskour^2^; E. Giné Ciprés^2^; A. Alpuente^2^; M. Torres‐ferrus^2^; A. Zanaboni^1^; J. Mehnert^3^; G. Castellazzi^1^; A. May^3^; P. Pozo‐Rosich^2^; C. Tasssorelli^4^

##### ^1^1IRCCS C. Mondino Foundation, Pavia, Italy; ^2^Headache and Neurological Pain Research Group, Vall d’Hebron Research Institute, Department of Neuroscience, Universitat Autònoma de Barcelona, Barcelona, Spain; Neurology Department, Hospital Universitari Vall d’Hebron, Barcelona, Spain; ^3^University Medical Center Hamburg‐Eppendorf,Institute of Systems Neuroscience, Hamburg, Germany; ^4^IRCCS C. Mondino Foundation, Pavia, Italy; University of Pavia, Brain and Behavioral Science Department, Pavia, Italy

**Background and aims:** The BIOMIGA project aimed to identify multimodal biomarkers predicting response to erenumab an anti‐CGRP monoclonal antibody in subjects with migraine. Here we present the preliminary analysis of the clinical and biomolecular data of the BIOMIGA population.

**Methods:** After preregistration (clinicaltrials: NCT04503083) data were collected in three European headache centers (Italy, Spain, Germany). Participants were clinically phenotyped at baseline. Plasma levels and gene expression in peripheral blood mononuclear cells (PBMCs) were assessed for α‐CGRP, IL‐1β, and TNF‐α levels and relative at baseline and after a 3‐month course of treatment with erenumab. Subjects were defined as responders if they achieved a ≥ 50% reduction in monthly headache/days(MHD) at month 3.

**Results:** Of the 164 participants enrolled, 137 (83.5%) were female, median age 42 years, 38% had a diagnosis of chronic migraine (CM), and 62% of highly frequent episodic migraine (HFEM). The median MHD at baseline was 13 [8‐25 95% nonparametric C.I.]. At month 3, 47.5% of subjects qualified as “responders”. CM was more prevalent among non‐responders (*p* = 0.024) and responders generally had fewer baseline MHDs. At baseline, responders had higher α‐CGRP plasma levels (433 vs. 361 pg/ml; *p* = 0.017), especially CM subjects (*p* = 0.039). IL1β expression was higher in responders (*p* = 0.033), notably in EM subjects (*p* = 0.029). At month 3, CM non‐responders exhibited a persistent higher CGRP expression in PBMCs (*p* = 0.018) compared to responders.

**Conclusion:** Clinical and molecular profiles could become treatment response biomarkers. Elevated baseline plasma CGRP and IL‐1β expression may help identify patients more likely to benefit from erenumab. Funding: This study was funded by ERANet Neuron.

**Disclosure:** DM declares honoraria from Lundbeck and AbbVie HB declares honoraria from Novartis, Teva, Lundbeck and Eli Lilly P.P.‐R. declares honoraria from AbbVie, Amgen, Dr Reddy's, Eli Lilly, Lundbeck, Medscape, Novartis, Organon, Pfizer and Teva Pharmaceuticals. Her research group has received research grants from AbbVie, AGAUR, EraNet Neuron, FEDER RIS3CAT, Instituto Investigación Carlos III, MICINN, Novartis, and Teva Pharmaceuticals, and has received funding for clinical trials from AbbVie, Amgen, Biohaven, Eli Lilly, Lundbeck, Novartis, Pfizer and Teva Pharmaceuticals. She is the Honorary Secretary of the International Headache Society, is on the editorial board of Revista de Neurologia, is an associate editor for Cephalalgia, Headache, Neurologia, Frontiers of Neurology, and is an advisor of the Scientific Committee of the Editorial Board of The Journal of Headache and Pain. CT has received, in the last 3 years, personal fees for the participation in advisory boards or for speaking at sponsored symposia from AbbVie, Eli Lilly, Ipsen, Lundbeck, Medscape, Pfizer and Teva. Her research group has received research grants from AbbVie, EraNet Neuron, Migraine Research Foundation and competitive grant from the Italian Ministry of Health. Her institution has received payments for clinical trials from AbbVie, Biohaven, Eli Lilly, Ipsen, Lundbeck, Pfizer and Teva. She is past‐President of the International Headache Society, Associate Editor of Cephalalgia.

## EPR‐146

### Patterns of referral to a headache unit: A gender perspective

#### E. Varas Martín; I. Ros González; Á. Sierra Mencía; A. Recio García; R. Areses Calderón; P. Mariluz; Á. Guerrero Peral

##### Headache Unit, Neurology Department, University Hospital, Valladolid, Spain

**Background and aims:** No major differences have been described regarding levels of care based on gender in migraine. Despite this, it has been proposed that women are more likely than men to receive treatment for migraine. We aimed to analyze differences regarding referral to a headache unit from a gender perspective.

**Methods:** In January 2008, an outpatient headache unit was set up in a tertiary hospital. Patients were referred mainly from general practitioners (GP) and, also, from general neurologists and other specialties. All patients were prospectively registered. Variables gathered in each patient were diagnosis, referral source, age at inclusion, time from onset of headache, gender, and previous therapies.

**Results:** In January 2025, 9940 patients had been included in the registry. Among them, 6220 patients (4956, 79.7% female) with migraine. Age at first consultation (39.93 ± 15.02 vs. 37.07 ± 15.52 years, *p*:0.004) and latency in years from onset of migraine to referral (18.11 ± 14.83 vs. 14.95 ± 14.19, *p* < 0.0001), were higher in female patients. Considering previous therapy, women had previously received more frequently triptans as symptomatic therapy (28.6% vs. 21.5%, *p* < 0.001) and at least one preventive (39.5% vs. 31.8%, *p* < 0.001) When considering only patients referred from GP, age at first consultation (39.32 ± 15.06 vs. 36.02 ± 15.28 years, *p* < 0.001) and latency from onset (17.73 ± 14.82 vs. 13.73 ± 13.28 years, *p* < 0.0001), were also higher among women, and they also previously received more frequently triptans (29.4% vs. 21.6%, *p* < 0.001) and preventive therapy (34.7% vs. 25.7%, *p* < 0.001).

**Conclusion:** Regardless of the referral source, women are referred to a headache unit later but having received more treatment.

**Disclosure:** Nothing to disclose.

## EPR‐147

### Optimization and validation of a new score in diagnosing spontaneous intracranial hypotension

#### K. Xie; Z. Wang; Q. Guo; T. Xia; F. Wang; H. Ye; C. Shen; Y. Li; J. Liu; Y. Dai; Y. An; Z. Wang; T. Liu; J. Wu; Z. Qi; L. Wu; J. Li

##### Xuanwu Hospital, Capital Medical University, Beijing, China

**Background and aims:** Diagnosis of spontaneous intracranial hypotension (SIH) relies on detecting cerebrospinal fluid (CSF) leak by myelography and opening pressure by lumbar puncture. However, these intrusive examinations limit the early diagnosis. We developed a new scoring system using magnetic resonance imaging (MRI) based on the Bern score to assist in diagnosing SIH.

**Methods:** This study was conducted from November 2018 to October 2022. SIH patients were diagnosed by ICHD‐3 and with CSF leak detected by myelography. Cerebral venous disease (CVD) patients and healthy controls were also included for differentiation. Six signs from the Bern score were evaluated. The new scoring system was developed from the items of the Bern score using binary logistic regression analysis. Its likelihood of the diagnosis was compared to that of the Bern score and the restricted Bern score.

**Figure:**
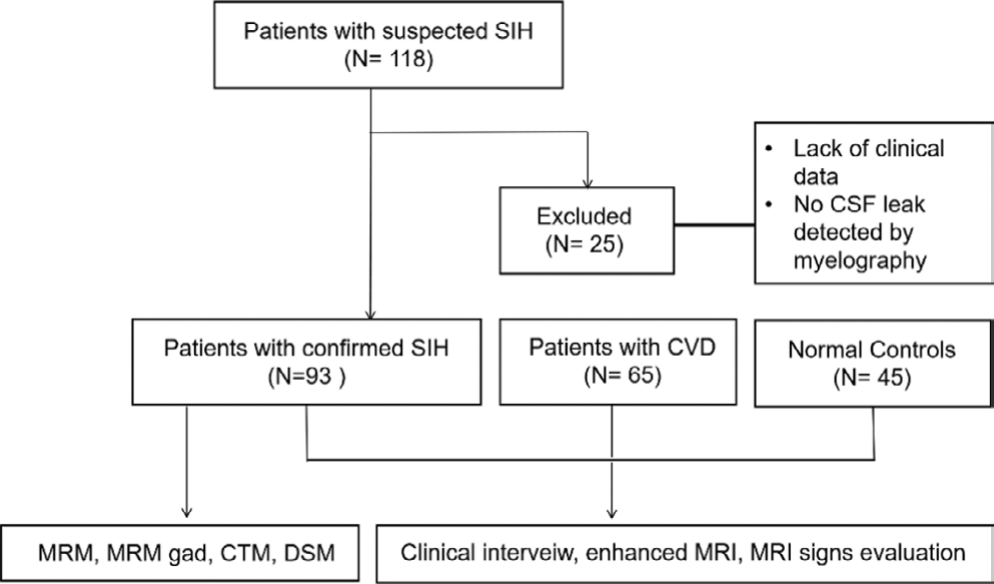
**FIGURE 1** Study flowchart of the patient inclusion and clinical evaluation

**Results:** A total of 93 SIH patients, 65 CVD patients and 45 healthy controls were studied. Three qualitative signs were selected. Pachymeningeal enhancement and subdural fluid were weighted major (2 points each), and venous sinus distention was weighted minor (1 point). The cut‐off value was 0.5 on a scale of 5 points. The new score showed better discriminatory value compared to the Bern score and restricted Bern score by the McNemar test (*p* < 0.001), sensitivity (93.5%), specificity(96.4%), and AUC(0.97). It also showed consistent diagnostic value as disease duration varied.

**Figure:**
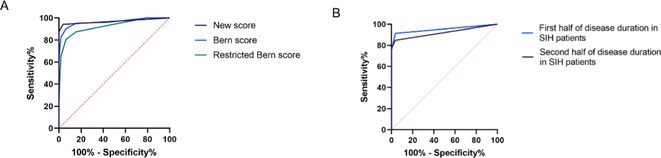
**FIGURE 2** Receiver operator characteristic (ROC) curve of the Bern score, restricted Bern score, the new score and the new score in different disease duration

**Figure:**
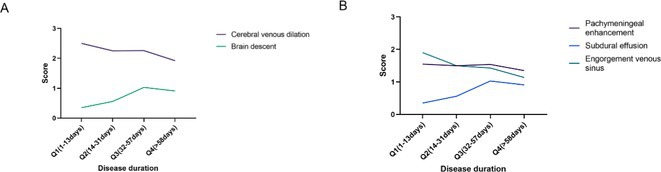
**FIGURE 3** Time‐dependent variation of MRI findings

**Conclusion:** The new MRI‐based scoring system effectively predicts SIH diagnosis using qualitative signs, making it more practical for clinical use and facilitating earlier identification and diagnosis of SIH.

**Disclosure:** Nothing to disclose.

## EPR‐148

### Inhibiting PAC1 receptor internalization and ERK activation may reduce hyperalgesia in a chronic migraine model

#### Z. Xiao

##### Department of Neurology, Renmin Hospital of Wuhan University, Wuhan, China.

**Background and aims:** Pituitary adenylate cyclase‐activating polypeptide (PACAP) and its PAC1 receptor are implicated in migraine, but their exact role in migraine pathogenesis remains unclear.

**Methods:** A chronic migraine (CM) rat model was induced by repeated nitroglycerin (NTG) injections. Mechanical and thermal pain thresholds were assessed using von Frey filaments and the hot plate test. c‐Fos, CGRP, PACAP, PAC1, PKA, and phosphorylated ERK levels were analyzed via western blot and immunofluorescence. PAC1 receptor internalization was visualized by fluorescence and confocal microscopy.

**Results:** NTG or PACAP administration increased c‐Fos and CGRP expression. Pitstop2 significantly reduced hyperalgesia in CM rats, whereas PACAP6‐38 had no effect. Pitstop2 blocked PAC1 receptor internalization and prevented PKA and ERK pathway activation, while PACAP6‐38 did not.

**Conclusion:** Inhibiting PAC1 receptor internalization alleviates allodynia in CM rats by suppressing ERK signaling. Modulating receptor internalization offers a novel approach for understanding PACAP signaling in the trigeminal vascular system and migraine pathogenesis.

**Disclosure:** Nothing to disclose.

## EPR‐149

### Neurovascular demand‐supply mismatch of visual network underpins aura phenomenon ignition in patients with migraine

#### L. Tartaglione ^1^; M. Silvestro^1^; F. Esposito^2^; A. De Rosa^2^; I. Orologio^1^; F. Trojsi^1^; P. Garcia‐Polo^3^; G. Tedeschi^1^; A. Tessitore^1^; M. Cirillo^2^; A. Russo^1^

##### ^1^Headache Centre, Department of Advanced Medical and Surgical Sciences, University of Campania “Luigi Vanvitelli”, Naples, Italy; ^2^Advanced MRI Neuroimaging Centre, Department of Advanced Medical and Surgical Sciences, University of Campania “Luigi Vanvitelli”, Naples, Italy; ^3^General Electric Healthcare, Spain

**Background and aims:** Although neuroimaging investigations have demonstrated that “hyperresponsive” and “hyperconnected” visual cortices may represent the functional substrate of cortical spreading depolarization in patients with migraine with aura (MWA), the mechanisms underpinning the brain "tendency" to ignite the aura phenomenon are still matter of debate. Since triggers able to induce aura increase brain increase energy demand, a vascular supply unable to satisfy the increased energy requirement could be hypothesized.

**Methods:** We recruited 23 patients with MWA, 25 patients with migraine without aura (MWoA) and 20 healthy controls (HC). All patients and HC underwent a 3‐Tesla MRI in order to obtain both cerebral blood flow and local functional connectivity maps. Finally, regional neurovascular coupling was estimated from the correlation coefficient between ReHo map and cerebral blood flow maps.

**Results:** A significantly higher regional cerebral blood flow across the visual cortex of both hemispheres was detected in MWA patients compared to patients with MWoA. Concomitantly, a reduced neurovascular coupling in the primary visual cortex parcel of the visual network was observed in the left hemisphere of MWA patients, compared to patients with MWoA and HC.

**Figure:**
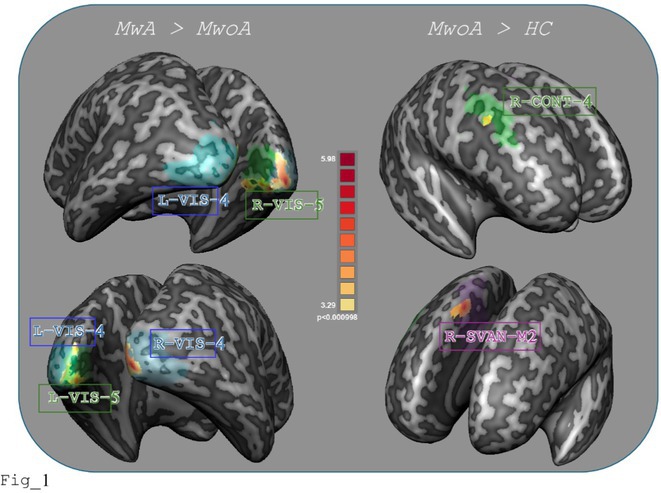
**FIGURE 1** Significant differences in voxel‐based CBF maps (*p* < 0.05 – TFCE corrected) between MwA and MwoA patients (left) and between MwoA patients and HCs (right).

**Figure:**
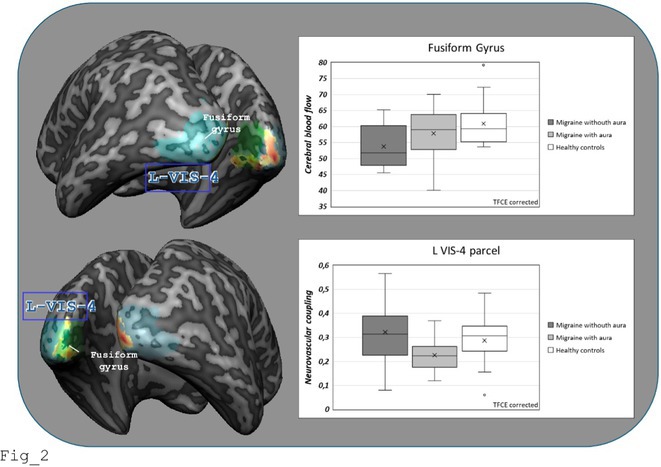
**FIGURE 2** Significant differences in visual areas CBF maps and VIS‐4 parcel neurovascular‐coupling according to Schaefer atlas (ref. 28) (TFCE corrected) showed on inflated brains and by box‐plots in patients with MwA, patients with MwoA and HC.

**Figure:**
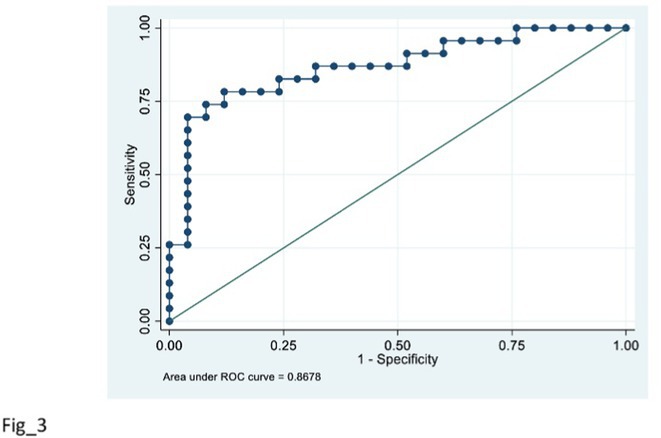
**FIGURE 3** ROC curve analysis of logistic regression model considering the rCBF of primary and secondary right visual cortices and neurovascular‐coupling of VIS‐4 parcel from Schaefer atlas (ref. 28).

**Conclusion:** Visual cortex neurovascular “decoupling” might represent the “link” between trigger exposure and aura phenomenon ignition. While vascular oversupply may compensate neurovascular demand‐supply at rest, it becomes inadequate in case of increased energy demand (as in response to aura triggers), paving the way to the aura phenomenon ignition in MWA. Whether preventive treatments may restore energy demands and cerebral blood flow trade‐off within the visual network should be further investigated.

**Disclosure:** Nothing to disclose.

## EPR‐150

### Identifying comorbidity profiles in migraine patients: results from the REFINE study

#### S. Avaltroni ^1^; R. Ornello^1^; V. Caponnetto^2^; A. Onofri^1^; C. Rosignoli^1^; M. Braschinsky^3^; O. Šved^3^; R. Gil‐Gouveia^4^; C. Lampl^5^; J. Paungarttner^5^; P. Martelletti^6^; W. Wells‐Gatnik^6^; I. Martins^7^; D. Mitsikostas^8^; L. Apostolakopoulou^8^; G. Nabaei^9^; A. Ozge^10^; D. Narin^10^; P. Pozo‐Rosich^11^; A. Muñoz‐Vendrell^11^; M. Prudenzano^12^; M. Gentile^12^; K. Ryliskiene^13^; J. Vainauskiene^13^; S. Sacco^1^

##### ^1^Department of Biotechnological and Applied Clinical Sciences, University of L’Aquila, L’Aquila, Italy; ^2^Department of Life, Health and Environmental Sciences, University of L'Aquila, L'Aquila, Italy; ^3^Department of Neurology and Neurosurgery, Institute, of Clinical Medicine, Faculty of Medicine, University of Tartu, Tartu, Estonia; ^4^Headache Center, Hospital da Luz, Lisbon, Portugal; ^5^Headache Medical Center, Seilerstaette Linz, Linz, Austria; ^6^Department of Clinical and Molecular Medicine, Sapienza University, Rome, Italy; ^7^Centro de Estudios Egas Moniz, Faculty of Medecine, University of Lisbon, Lisbon Portugal; ^8^First Department of Neurology, Aeginition Hospital, National and Kapodistrian University of Athens, Athens, Greece; ^9^Iranian Center of Neurological Research, Shariati Hospital, Tehran, University of medical sciences, Tehran, Iran, ^10^Department of Neurology, Mersin University Medical Faculty, Mersin, Turkey, ^11^Headache Unit, Neurology department, Vall d'Hebron university Hospital, Barcelona, Spain, ^12^Headache Center, Amaducci Neurological Clinic, Policlinico General Hospital, Bari, Italy, ^13^Center of Neurology, Vilnius University, Vilnius, Lithuania

**Background and aims:** Migraine is a leading cause of disability among young individuals worldwide. While preventive treatments improve outcomes for many, resistant and refractory migraine cases often fail to respond. This study aimed to identify specific comorbidity patterns in patients with resistant and refractory migraine.

**Methods:** The REFINE study included 689 patients classified as resistant, refractory, or non‐resistant/non‐refractory migraine according to European Headache Federation criteria. A Latent Class Analysis (LCA) was conducted using eight comorbidities associated with migraine—obesity, autoimmune/rheumatological disorders, psychiatric diseases, cardiovascular disorders, gastrointestinal conditions, chronic pain, allergic/respiratory conditions, and musculoskeletal disorders—and the EHF diagnosis as variables.

**Results:** The three‐class model provided the best fit (BIC = 6065.292). Class 1 comprised 30.99% of patients, with the highest prevalence of resistant (22.8%, vs. 13.9% in Class 2 and 14.6% in Class 3) and refractory migraine (33.2%, vs. 5.2% in Class 2 and 4.1% in Class 3). Class 1 also showed the highest prevalence of chronic pain (77.8%), psychiatric disorders (85.9%), and allergic/respiratory conditions (32.2%). Obesity was present in 100% of Class 1 and Class 3 but absent in Class 2. Autoimmune/rheumatological disorders affected 32.2% of Class 1 patients. Cardiovascular (27.9%) and gastrointestinal conditions (5.7%) were less frequent in Class 1.

**Conclusion:** LCA identified a class with a higher prevalence of obesity, psychiatric disorders, and chronic pain, significantly associated with resistant and refractory migraine, suggesting the need for tailored management strategies.

**Disclosure:** Nothing to disclose.

## EPR‐151

### Glymphatic dysfunction in migraine mice model

#### Z. Xiao; W. Huang; Y. Lei

##### Department of Neurology, Renmin Hospital of Wuhan University, Wuhan, China

**Background and aims:** The glymphatic system is crucial for waste removal in the central nervous system, facilitated by aquaporin‐4 (AQP4) at astrocytic ends. While it is involved in several neurological disorders, the link between the glymphatic system and migraine remains unclear.

**Methods:** Using a nitroglycerin‐induced migraine model in C57BL/6 mice, we examined glymphatic function by assessing cerebrospinal fluid (CSF) tracer influx. AQP4 expression and polarization were also measured. To investigate the role of glymphatic dysfunction, mice were treated with TGN‐020, an AQP4 blocker.

**Results:** In the migraine model, glymphatic CSF influx was reduced, and AQP4 expression and polarization were impaired, indicating glymphatic dysfunction. Further inhibition of glymphatic function with TGN‐020 worsened migraine‐related pathology in mice.

**Conclusion:** The findings suggest that glymphatic dysfunction may exacerbate migraine pathology. These results highlight the potential role of the glymphatic system in migraine, offering new targets for prevention and treatment.

**Disclosure:** Nothing to disclose.

## Movement disorders 2

## EPR‐152

### Characteristics of patients with Parkinson´s disease treated with a device‐aided therapy. A comparative analysis

#### D. Santos García ^1^; Á. Solleiro^1^; G. González‐Ortega^2^; P. Mir^3^; N. López‐Ariztegui^4^; R. García‐Ramos^5^; I. Legarda^6^; A. Planas‐Ballvé^7^; D. Alonso‐Modino^8^; P. Sánchez Alonso^9^; I. Cabo^10^; M. Blázquez Estrada^11^; f. Escamilla^12^; E. Cubo^13^; J. Hernández Vara^14^; Á. Sánchez Ferro^15^; S. DATs‐PD GETM Spanish Registry^16^

##### ^1^Neurology Department, CHUAC (Complejo Hospitalario Universitario de A Coruña), A Coruña, Spain; ^2^Hospital Universitario de Móstoles, Madrid, Spain; ^3^Hospital Universitario Virgen del Rocío, Sevilla, Spain; ^4^Hospital Universitario de Toledo, Toledo, Spain; ^5^Hospital Universitario Clínico San Carlos, Madrid, Spain; ^6^Hospital Universitario Son Espases, Palma de Mallorca, Spain; ^7^Consorci Sanitari Integral, Hospital Moisés Broggi, Sant Joan Despí, Barcelona, Spain; ^8^Hospital Universitario de la Candelaria, Santa Cruz de Tenerife, Spain; ^9^Hospital Universitario Puerta de Hierro, Madrid, Spain, ^10^Complejo Hospitalario Universitario de Pontevedra (CHOP), Pontevedra, Spain, ^11^Hospital Universitario Central de Asturias (HUCA), Oviedo, Spain, ^12^Hospital Universitario Virgen de las Nieves, Granada, Spain, ^13^Complejo Hospitalario Universitario de Burgos, Burgos, Spain, ^14^Hospital Universitario Vall d´Hebron, Barcelona, Spain, ^15^Hospital Universitario 12 de Octubre, Madrid, Spain, ^16^Fundación Degen ‐ COPPADIS Study Group

**Background and aims:** New device‐aided therapies (DATs) have emerged to treat people with Parkinson´s disease (PwP). Our aim was to know which DATs were indicated by the neurologist and to compare the characteristics of the patients treated in Spain under real clinical practice in 2024.

**Methods:** Data collected in the DATs‐PD GETM Spanish Registry were used (REDCap). This is a descriptive, observational, prospective, multicenter, clinical registry with progressive inclusion of PwP treated with a DAT in daily clinical practice conditions in more than 40 centers from Spain for 10 years. For this proposal, only data from visits V1 (indication of DAT) and V2 (initiation of DAT) were analyzed but no follow‐up data. All patients with information on DAT received until 31/DEC/2024 were included.

**Results:** A total of 313 PD patients (66.7  ± 9.6 years old at V2; 61.7% males) were treated with a DAT. The most frequent was subcutaneous foslevodopa/foscarbidopa (fLD/fCD) (47%) followed by deep brain stimulation (DBS) (20.1%) and continuous subcutaneous apomorphine infusion (CSAI) (19.8%) (Figure 1A). Up to 23.6% had received at least one previous DAT (Figure 1B) and 47% an on‐demand therapy (Figure 1C). Differences in age, time with fluctuations and other aspects (motor status, quality of life, activities of daily living, etc.) were observed between different DAT groups (Table 1 and Figure 2).

**Figure:**
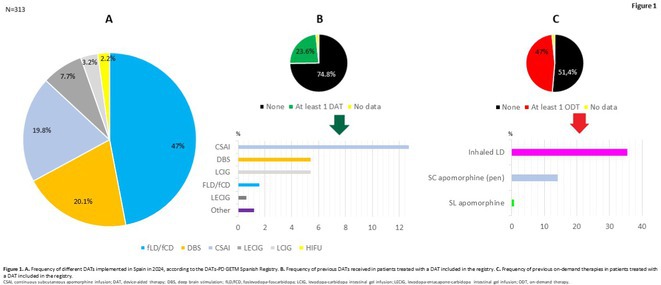
FIGURE 1

**Figure:**
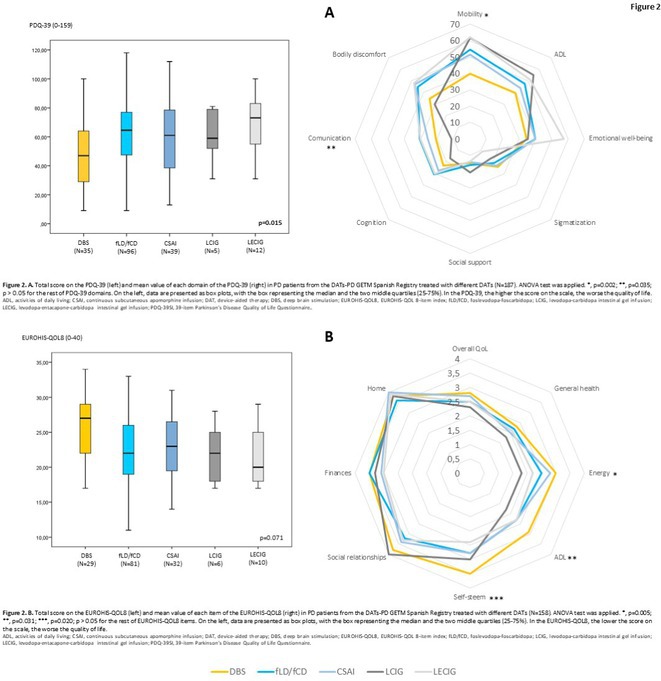
FIGURE 2

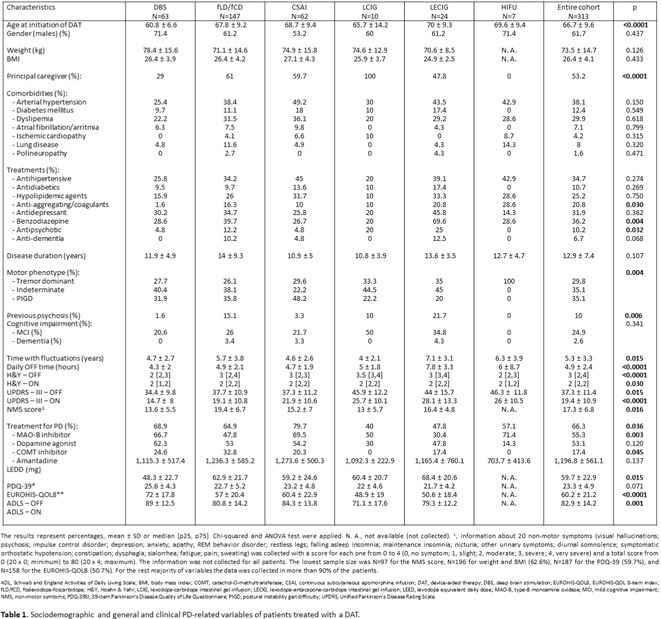

**Conclusion:** Subcutaneous therapies and DBS were the most frequent DATs (86.9%) implemented in Spain in 2024. Patients treated with DBS were younger and had less advanced disease, whereas those who received LECIG were the most affected.

**Disclosure:** The authors report no conflict of interest.

## EPR‐153

### Impairment of eye movements in early‐stage Parkinson's disease patients

#### Z. Popovic^3^; T. Gilman Kuric^3^; I. Rajkovaca Latic^4^; E. Strujic ^2^; S. Matosa^3^; L. Kusic^1^; A. de Gobbis^5^; A. Sadikov^5^; V. Groznik^5^; D. Georgiev^6^; S. Tomic^3^

##### ^1^Faculty of Medicine Osijek, Josip Juraj Strossmayer University of Osijek, Osijek, Croatia; ^2^Department of Neurology, University Hospital Center Osijek, Osijek, Croatia; ^3^Faculty of Medicine Osijek, Josip Juraj Strossmayer University of Osijek, Osijek, Croatia; Department of Neurology, University Hospital Center Osijek, Osijek, Croatia; ^4^Faculty of Medicine Osijek, Josip Juraj Strossmayer University of Osijek, Osijek, Croatia; Department of Gastroenterology and Endocrinology, Dr. Josip Bencevic General Hospital, Slavonski Brod, Croatia; ^5^Faculty of Computer and Information Science, University of Ljubljana, Ljubljana, Slovenia; ^6^Faculty of Computer and Information Science, University of Ljubljana, Ljubljana, Slovenia; Department of Neurology, University Medical Centre Ljubljana, Ljubljana Slovenia

**Background and aims:** One of the most prevalent motor symptoms in Parkinson's disease (PD) is eye movement impairment, presenting in 75% of PD patients, which have fragmented and hypometric smooth pursuit movements with prolonged latency, and slow and hypometric saccades with impaired precision. We aimed to investigate differences in smooth pursuit, memory saccades and reading between patients with early‐stage PD and healthy controls.

**Methods:** We conducted a cross‐sectional study with idiopathic PD patients in early stage of PD (Hoehn and Yahr stage 0, 1 and 2) and healthy controls. The impairment of smooth pursuit, saccades, antisaccades, and memory‐guided saccades was evaluated with eye‐tracker analysis using a battery of tests.

**Results:** Forty two subjects with early‐stage idiopathic PD and 50 healthy controls participated in the study. There were no statistically significant differences in age, gender, years of education, or cognition between the groups. Average duration of disease in PD patients was 4 (2‐7) years. Early‐stage PD patients showed impairment in velocity, phase, and range of motion of smooth pursuit eye movements (Table 1), as well as impaired precision and recollection performing visually‐guided memory saccades (Table 2). There is also a reading dysfunction, with slower reading speed and longer duration of eye fixations (Table 3).

**Figure:**
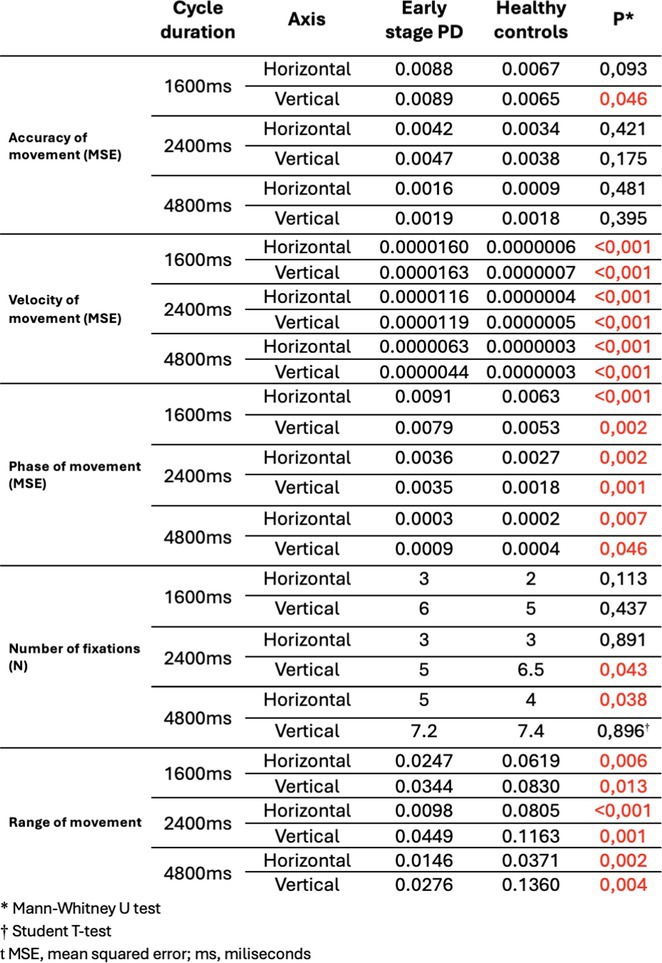
**TABLE 1** Comparison of smooth pursuit parameters in all observed cycles between patients with early‐stage PD and healthy controls.

**Figure:**
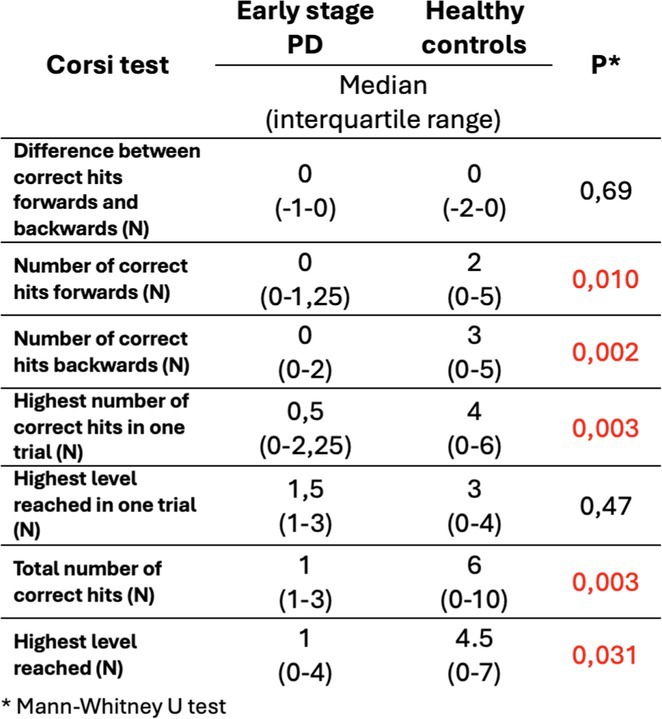
**TABLE 2** Comparison of visually guided memory saccades parameters between patients with early‐stage PD and healthy controls.

**Figure:**
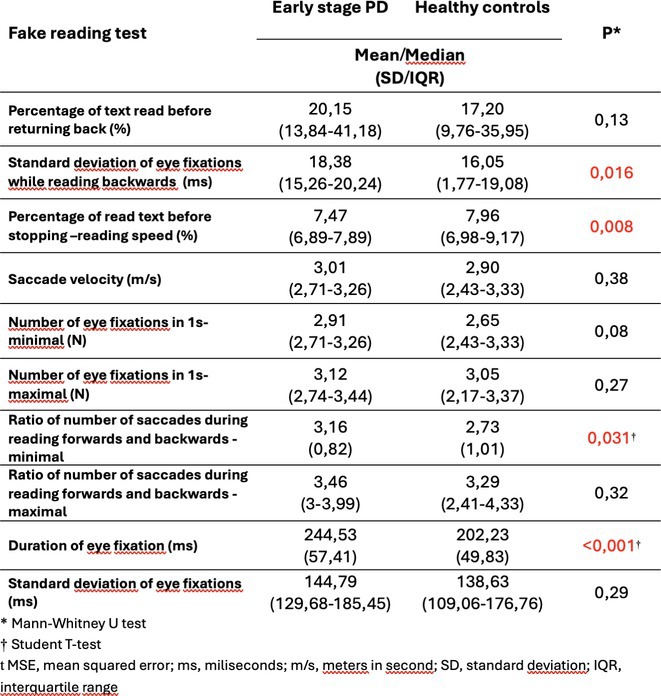
**TABLE 3** Comparison of fake reading test parameters between patients with early‐stage PD and healthy controls.

**Conclusion:** Results suggest that impaired smooth pursuit movements, visually‐guided reflexive saccades and reading functions are present in early‐stage PD, even without other expressed motor symptoms. These findings could potentially contribute to the development of new and non‐invasive diagnostic biomarkers in PD.

**Disclosure:** Nothing to disclose.

## EPR‐154

### Retinal structural changes in Parkinson's disease (PD): Comparison between GBA1‐PD and non‐mutated PD

#### G. Di Rauso ^1^; M. Giacomelli^2^; G. Portato^1^; S. Grisanti^3^; V. Fioravanti^1^; L. Caprari^1^; G. Toschi^1^; G. Argenziano^1^; R. Sabadini^1^; V. Ferrari^1^; A. Groppi^1^; A. Melpignano^4^; A. Scaglioni^5^; B. Canuti^2^; M. Paoli^2^; M. Romano^2^; V. Bottazzi^2^; S. Annovi^2^; G. De Ninno^2^; R. Surano^2^; E. Monfrini^6^; A. Di Fonzo^6^; M. Vecchi^2^; F. Cavallieri^1^; F. Valzania^1^

##### ^1^Neurology Unit, Neuromotor and Rehabilitation Department, Azienda USL‐IRCCS di Reggio Emilia, Reggio Emilia, Italy; ^2^Ophthalmology Unit, Azienda USL‐IRCCS di Reggio Emilia, Reggio Emilia, Italy; ^3^Clinical and Experimental Medicine PhD Program, University of Modena and Reggio Emilia, Modena, Italy; ^4^Presidio Ospedaliero Fidenza AUSL Parma, Fidenza, Italy; ^5^Centro S. Maria ai Servi, Fondazione Don Gnocchi, Parma, Italy; ^6^Foundation IRCCS Ca' Granda Ospedale Maggiore Policlinico, Neurology Unit, Milan, Italy

**Background and aims:** Retinal thinning has been observed in Parkinson's disease (PD) patients compared to healthy controls, and few studies correlated these changes with disease severity and cognitive impairment although data are still controversial. To date, there are no data about retinal structural changes in GBA1‐related PD (GBA1‐PD) patients. The aim of the study was to assess differences in retinal thickness between GBA1‐PD and non‐mutated PD (NM‐PD) and to explore correlations between clinical and retinal parameters.

**Methods:** A consecutive cohort of GBA1‐PD patients was matched for age, sex, disease duration, H&Y stage with a cohort of NM‐PD patients. All patients underwent a clinical assessment (MDS‐UPDRS and MoCA) and optical coherence tomography (OCT) to evaluate retinal nerve fiber layer (RNFL) thickness with three circular scans (diameters: 3.5‐,4.1‐ and 4.7‐mm).

**Results:** A total of 52 PD patients (104 eyes from 26 GBA1‐PD and 26 NM‐PD) were included. The two groups did not show statistically significant differences in clinical variables. Temporal RNFL thickness was significantly reduced in NM‐PD compared to GBA1‐PD at 3.5‐, 4.1‐ and 4.7 mm (*p* = 0.021, *p* = 0.006, *p* = 0.005 respectively). In the total PD cohort, the average temporal RNFL thickness (4.1 mm scan) inversely correlated with MDS‐UPDRS part I total score (*p* = 0.04).

**Conclusion:** This study highlights a reduced retinal thickness in NM‐PD compared to GBA1‐PD, showing a greater involvement of papillo‐macular bundle in NM‐PD than in GBA1‐PD. This may suggest differential involvement of retinal cells, potentially related to distinct pathophysiological mechanisms. Interestingly, in the total PD cohort temporal RNFL thickness was inversely correlated with non‐motor symptoms burden.

**Disclosure:** The authors have no conflicts of interest to declare that are relevant to the content of this abstract. Patients included in the study are part of the FIN‐RER study.

## EPR‐155

### Patient‐reported outcomes of long‐term deutetrabenazine use in the RIM‐td open‐label extension study European cohort

#### R. Hauser^1^; K. Duma ^2^; N. Chaijale^3^; S. Factor^4^; J. Joohi.Jimenez‐shahed@mountsinai.org^5^; N. Gross^3^; L. Marinelli^3^; M. Gordon^3^; K. Anderson^6^

##### ^1^University of South Florida Parkinson's Disease and Movement Disorders Center, Tampa, FL, USA; ^2^Teva Pharmaceuticals Europe B.V., Amsterdam, The Netherlands; ^3^Teva Branded Pharmaceutical Products R&D, Inc., West Chester, PA, USA; ^4^Jean and Paul Amos Parkinson's Disease and Movement Disorder Program, Emory University, Atlanta, GA, USA; ^5^Icahn School of Medicine at Mount Sinai, New York, NY, USA; ^6^Georgetown University, Department of Psychiatry & Department of Neurology, Washington, DC, USA

**Background and aims:** Deutetrabenazine is approved by the United States Food and Drug Administration for the treatment of tardive dyskinesia (TD) in adults. This analysis assessed outcomes self‐reported by patients treated with deutetrabenazine in a European cohort of the RIM‐TD study.

**Methods:** Patients completing the phase 3 ARM‐TD or AIM‐TD study could enter RIM‐TD, a 3‐year, single‐arm open‐label extension study (NCT02198794), where they received deutetrabenazine for ≤145 weeks. This post hoc subgroup analysis focused on participants from European countries. Assessments included Abnormal Involuntary Movement Scale (AIMS), Patient Global Impression of Change (PGIC), a modified Craniocervical Dystonia Questionnaire (mCDQ‐24) scale, and adverse event (AE) and discontinuation rates.

**Results:** Among the 142 participants enrolled in Europe (Table 1), mean ± SE total deutetrabenazine dose at Week 145 was 39.5 ± 1.13 mg/day. At Week 145, symptom improvement was observed based on reductions in total motor AIMS scores (mean −6.4; items 1–7, total score range 0–28) and on patient‐reported awareness of abnormal movements or resulting incapacitation (Table 2). Most patients (56%) scored much/very much improved on the PGIC. At Week 106, improvements in quality of life were observed on the mCDQ‐24 scale total score (mean −4.0) and 4 subdomain scores; the largest change was in the stigma subdomain (mean −8.2). AEs and treatment‐related AEs were reported for 77.5% and 45.8% of patients, respectively; 66.2% of patients reached Week 145 (Table 3).

**Figure:**
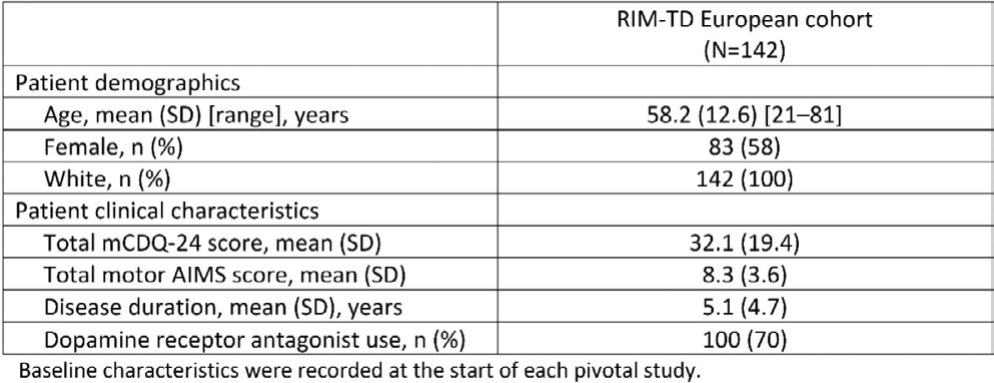
**Table 1** Baseline characteristics and patient demographics.

**Figure:**
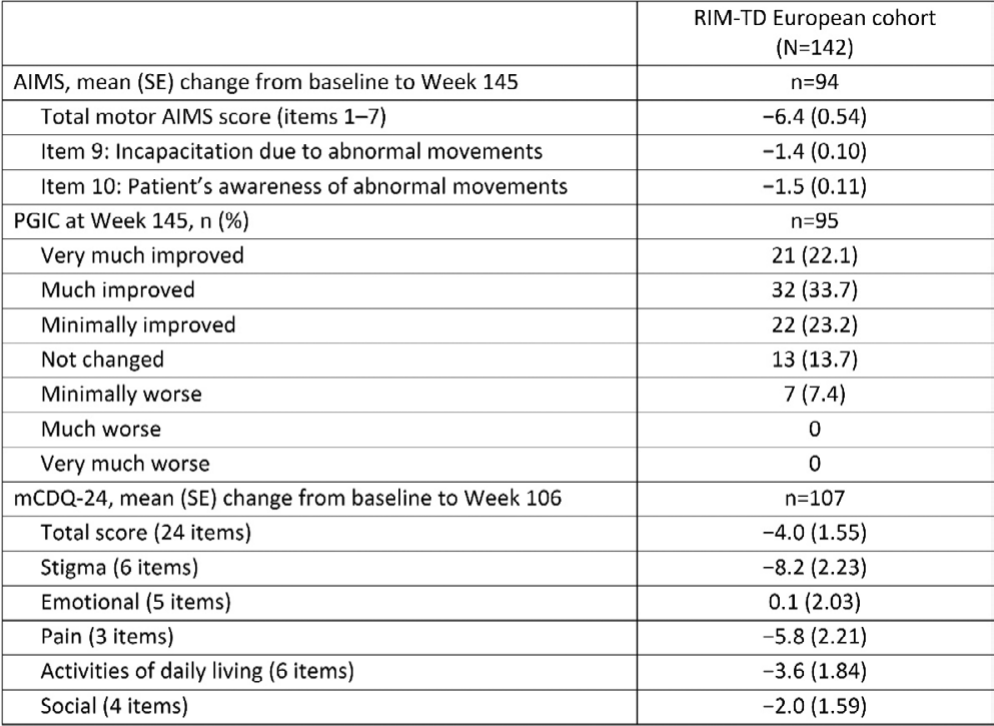
**Table 2** AIMS, PGIC, and mCDQ‐24.

**Figure:**
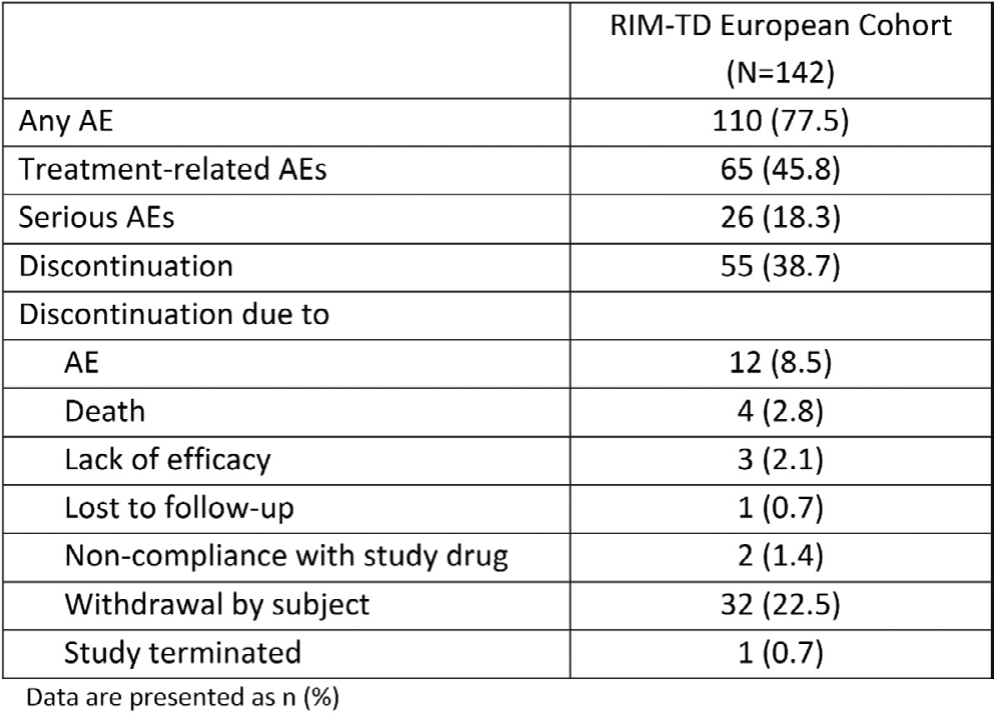
**Table 3** AEs and study discontinuation.

**Conclusion:** Long‐term deutetrabenazine treatment was associated with improvements in patient‐reported measures of treatment success and was well tolerated in patients with TD treated at European sites.

**Disclosure:** This study was funded by Teva Branded Pharmaceutical Products R&D, Inc.

## EPR‐156

### Abstract withdrawn

## EPR‐157

### Multimodal deep learning approaches to predict motor outcomes in Parkinson's disease patients

#### M. Malaguti ^1^; M. Moroni^2^; F. Ragni^2^; S. Bovo^2^; W. Endrizzi^2^; C. Longo^1^; M. Chierici^2^; L. Gios^3^; D. Ottaviani^4^; R. Di Giacopo^4^; L. Avanzino^5^; R. Marchese^5^; F. Di Biasio^5^; M. Marenco^5^; A. Uccelli^5^; B. Giometto^1^; V. Osmani^2^; G. Jurman^2^

##### ^1^Azienda Provinciale per i Servizi Sanitari (APSS) di Trento, Trento, Italy; ^2^Fondazione Bruno Kessler, Trento, Italy; ^3^TrentinoSalute4.0 – Competence Center for Digital Health of the Province of Trento, Trento, Italy; ^4^Department of Neurology, Santa Maria del Carmine Hospital, Azienda Provinciale per i Servizi Sanitari (APSS), Rovereto, Italy; ^5^IRCCS Ospedale Policlinico San Martino di Genova, Italy

**Background and aims:** Parkinson's disease (PD) trajectories are highly variable and the identification of prognostic features represents a challenge toward personalized approaches. Multicenter studies collecting data from multiple modalities (imaging, electronic health records – EHRs) allow to investigate the prognostic role of single data types and of their combination. This study investigates the ability of Deep Learning (DL) in predicting motor functions both from single and multimodal information.

**Methods:** Imaging (DaTSCAN) and EHRs data (including demographic, motor, clinical and pharmacological data) from *n* = 339 patients from the Parkinson's Progression Markers Initiative (PPMI) were used to predict motor impairments at three years follow‐up assessed through the unified Parkinson's disease rating scale part III (UPDRS < 30). Prediction was done using a modified PDNet applied to imaging volumes alone or combined with radiomic features (unimodal), while the multimodal approach combined DaTSCAN with EHRs data. Models’ performance was measured through Matthews Correlation Coefficient (MCC) and Balanced Accuracy (Acc) on a 20% holdout PPMI validation set and on an external cohort of *n* = 64 PD patients collected for the project NeuroArtP3 (NET‐2018‐12366666).

**Results:** DaTSCAN imaging was informative of motor outcomes (Table 1. PPMI Acc = 0.67; NeuroArtP3 Acc = 0.73). Adding radiomic features improved the accuracy on the PPMI cohort (Acc = 0.76) but reduced generalization to the NeuroArtP3 group (Acc = 0.59). Multimodal approach increased prediction performance to 0.71 and 0.76 on the PPMI and NeuroArtP3 cohort, respectively.

**Figure:**
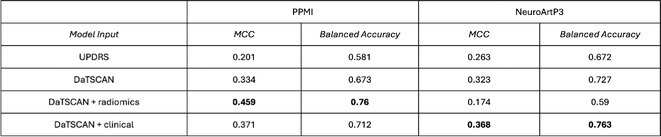
**TABLE 1** Performances of unimodal and multimodal PDNet on the PPMI and NeuroArtP3 validation cohort.

**Conclusion:** Combining imaging and EHRs with a multimodal approach lead to an improvement in the prognostic accuracy of DL models, which is robust to the generalization to external cohorts.

**Disclosure:** Nothing to disclose.

## EPR‐158

### Microstructural MRI and glymphatic flow alterations in patients with isolated REM sleep behavior disorder

#### S. Basaia ^1^; E. Sarasso^2^; A. Gardoni^1^; A. Grassi^1^; A. Brivio^3^; S. Marelli^4^; R. Balestrino^5^; L. Zenere^1^; A. Castelnuovo^4^; M. Malcangi^5^; E. Canu^1^; L. Ferini‐Strambi^4^; M. Filippi^6^; F. Agosta^7^

##### ^1^Neuroimaging Research Unit, Division of Neuroscience, IRCCS San Raffaele Scientific Institute, Milan, Italy; ^2^Neuroimaging Research Unit, Division of Neuroscience, and Department of Rehabilitation and Functional Recovery, IRCCS San Raffaele Scientific Institute, Milan, Italy; and DINOGMI, University of Genoa, Genoa, Italy; ^3^Vita‐Salute San Raffaele University, Milan, Italy; ^4^Sleep Disorders Center, Division of Neuroscience, IRCCS San Raffaele Scientific Institute, and Vita‐Salute San Raffaele University, Milan, Italy; ^5^Neurology Unit, IRCCS San Raffaele Scientific Institute, Milan, Italy; ^6^Neurology Unit, Neurorehabilitation Unit, Neurophysiology Service, and Neuroimaging Research Unit, Division of Neuroscience, IRCCS San Raffaele Scientific Institute, and Vita‐Salute San Raffaele University, Milan, Italy; ^7^Neuroimaging Research Unit, Division of Neuroscience, and Neurology Unit, IRCCS San Raffaele Scientific Institute, and Vita‐Salute San Raffaele University, Milan, Italy

**Background and aims:** To assess neurological, gait analysis, microstructural MRI, glymphatic flow alterations features in isolated‐REM sleep behavior disorder (iRBD) subjects relative to controls; to study the correlations between clinical and MRI features; and to compare sub‐groups of iRBD patients.

**Methods:** Forty‐four iRBD subjects and 52 controls underwent motor, non‐motor and cognitive assessments, gait analysis and MRI evaluations. Brain microstructural alterations were studied using Tract‐Based Spatial Statistic (TBSS) and Gray‐matter‐Based Spatial Statistics (GBSS). Diffusion‐Tensor Image Along the Perivascular Space (DTI‐ALPS) index was obtained for the evaluation of glymphatic flow functionality. Cluster analysis was applied to divide iRBD patients in sub‐groups. ANOVA models were used to compare clinical, and MRI data. Correlations between clinical and MRI data were assessed.

**Results:** IRBD subjects showed worse sleep quality, a reduced manual dexterity and spatio‐temporal gait parameters alterations relative to controls. iRBD showed microstructural alterations in the gray‐matter of frontal and parietal lobes, and in the white‐matter of brainstem and frontal lobe. iRBD showed lower DTI‐ALPS index relative to controls. Correlation analyses in the iRBD group showed that worse gray matter microstructural alterations correlated with worse performance in the Nine‐Hole‐Peg‐Test, lower peak turning velocity during Timed Up and Go test with a cognitive dual‐task and worse sleep quality. Cluster analysis resulted in two clusters, with one showing generally worse clinical, neuropsychological and gait performances together with a worse DTI‐ALPS index.

**Conclusion:** Clinical, gait analysis and MRI data collected longitudinally could be useful in the creation of predictive models for the conversion from iRBD to parkinsonisms.

**Disclosure:** Funding from the Italian Ministero della Salute (grant number RF‐2018‐12366746). Disclosures: SB, ES, SM, RB received grants from the Italian Ministry of Health. AEB, AGa, AGr, LZ, MM, AC nothing to disclose. LF‐S received speaker honoraria from Biprojet, Idorsia, Italfarmaco, and Takeda, and received research support from the Italian Ministry of Health and the Italian Ministry of University and Research. MF received compensation for consulting services or speaking activities from Alexion, Almirall, Bayer, Biogen, Celgene, Chiesi Italia SpA, Eli Lilly, Genzyme, Janssen, Merck‐Serono, Neopharmed Gentili, Novartis, Novo Nordisk, Roche, Sanofi Takeda, and TEVA; Advisory Boards for Alexion, Biogen, Bristol‐Myers Squibb, Merck, Novartis, Roche, Sanofi, Sanofi‐Aventis, Sanofi‐Genzyme, Takeda; scientific direction of educational events for Biogen, Merck, Roche, Celgene, Bristol‐Myers Squibb, Lilly, Novartis, Sanofi‐Genzyme; he receives research support from Biogen Idec, Merck‐Serono, Novartis, Roche, the Italian Ministry of Health, the Italian Ministry of University and Research, and FISM. FA received speaker honoraria from Biogen Idec, Roche, Eli Lilly and GE Healthcare; and grants from the Italian Ministry of Health, the Italian Ministry of University and Research, AriSLA, the European Research Council, the EU Joint Programme—Neurodegenerative Disease Research (JPND), and the Foundation Research on Alzheimer Disease (France).

## EPR‐159

### Evaluating beta‐sensing for the optimization of deep brain stimulation in Parkinson's disease

#### S. Malaspina ^1^; F. Valentino^2^; G. Belluscio^1^; S. Moraru^1^; M. Avenali^1^; R. Zangaglia^2^

##### ^1^Department of Brain and Behavioural Sciences, University of Pavia, Pavia, Italy; ^2^Parkinson's Disease and Movement Disorders Unit, IRCCS Mondino Foundation, Pavia, Italy.

**Background and aims:** Deep brain stimulation (DBS) is a well‐established treatment for advanced Parkinson's disease (PD). We aimed to compare beta‐sensing with clinical monopolar review and 3D‐radiological reconstructions to optimize DBS parameters in PD patients.

**Methods:** We included PD patients implanted with beta‐band sensing DBS devices. Baseline motor performances were evaluated using the Unified Parkinson's Disease Rating Scale (MDS‐UPDRS) part III in the OFF‐stimulation/OFF‐medication state. Non‐motor symptoms, motor fluctuations, and quality of life were assessed using MDS‐UPDRS parts I, II, and IV, and the Parkinson's Disease Questionnaire (PDQ‐39). DBS was activated using parameters pre‐established through monopolar review and 3D‐reconstructions. Three hours later, patients were clinically examined using MDS‐UPDRS‐III in an OFF‐med state, and new parameters were set based on beta‐sensing. At follow‐up visits, we reassessed the patients and examined beta‐band sensing recording. We also calculated Total Electrical Energy Delivered (TEED) at every timepoint.

**Results:** Eight PD patients (5 males, 3 females, mean age at onset 53.5 ± 5.5 years) underwent DBS surgery at a mean age of 63.4 ± 4.1 years. Seven received STN‐DBS and one GPi‐DBS. Baseline mean MDS‐UPDRS part III scores were 35.3 ± 7.0 (OFF‐med/OFF‐stim) and 23.3 ± 5.4 (OFF‐med/ON‐stim). Scores for parts I, II, IV, and PDQ‐39 were 8.0 ± 5.1, 7.3 ± 3.7, 3.9 ± 3.3, and 29.6 ± 27.9, respectively. Beta‐sensing suggested new parameters in six patients. Three attended the first follow‐up visit, showing trends toward improved MDS‐UPDRS and PDQ‐39 scores, though differences were not significant, with reduced TEED.

**Conclusion:** Beta‐sensing appears as effective as traditional methods for short‐term DBS optimization. We are collecting long‐term follow‐up data in a larger cohort to establish long‐term efficacy.

**Disclosure:** Nothing to disclose.

## EPR‐160

### Development of diagnostic, prognostic biomarkers and rehabilitation strategies in functional motor disorders

#### S. Cuoco ^1^; M. Gandolfi^2^; I. Di Vico^3^; P. Barone^1^; M. Pellecchia^1^; R. Erro^1^; A. Sandri^3^; I. Carotenuto^1^; M. Russo^1^; M. Tinazzi^2^

##### ^1^Neurological Clinic, AOU San Giovanni di Dio e Ruggi d', Aragona, Salerno, Italy; ^2^Department of Neurosciences, Biomedicine and Movement Sciences, University of Verona, Verona, Italy; ^3^Azienda Ospedaliera Universitaria Integrata Verona, Verona, Italy

**Background and aims:** Functional motor disorders (FMD) present delayed diagnoses and inadequate treatments underscoring the need for diagnostic and prognostic biomarkers [2,3].This research, funded by the European Union – Next Generation EU (NRRP M6C2 – Investment 2.1, PNRR‐MAD‐2022‐12376826), aimed to address this gap. To develop diagnostic and prognostic disease‐specific biomarker algorithms in motor, exteroceptive, and interoceptive domains through advanced behavioral, neurophysiological, and MRI assessments supported by explainable artificial intelligence.

**Methods:** FMD patients, healthy controls (HC), and patients with “organic” motor disorders underwent behavioral, neurophysiological, and MRI assessments targeting motor, exteroceptive, interoceptive, and cerebral domains.

**Results:** Preliminary analyses comparing 115 HC (mean age 39.62 ± 11.5 years) and 41 FMD patients (mean age 45.5 ± 13.3 years) revealed alexithymia, anxiety, depression, fatigue, pain and reduced quality of life in FMD patients (*p* < 0.05). Age‐matched subgroup analysis showed significant motor impairment in FMD, including reduced stride length and slower walking speed (*p* < 0.001, for all), with improved gait metrics in dual‐task visual conditions. Balance challenges were evident in Romberg index performance under single‐task conditions (*p* < 0.003) but not during dual‐task conditions (Figure 1). Blink reflex R2 response modulation between baseline and prepulse conditions differed significantly in FMD (*p* < 0.021), partially aligning with HC patterns (Table 1). Exteroceptive assessments showed altered laser‐evoked potentials in FMD, with N2P2 amplitude reductions during Diffuse Noxious Inhibitory Controls (DNIC) returning to baseline post‐DNIC (*p* < 0.05), mirroring HC patterns (Table 2). Interoceptive and MRI findings remain under analysis.

**Figure:**
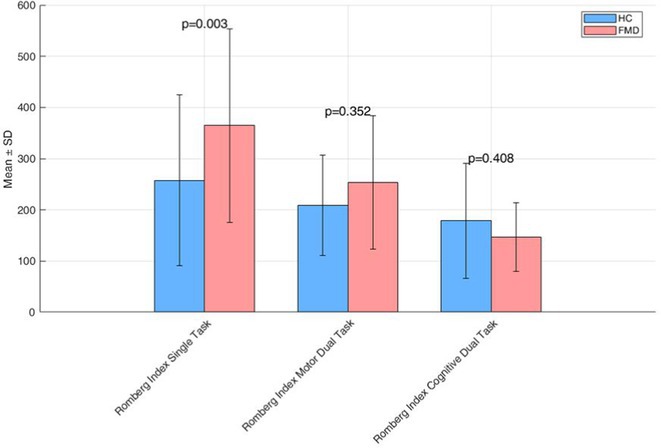
**FIGURE 1** Comparison of Romberg Index between Healthy Control (HC) and Functional Movement Disorders (FMD) subjects across single and dual‐task. Significant statistical *p*‐value set at 0.05.

**Figure:**
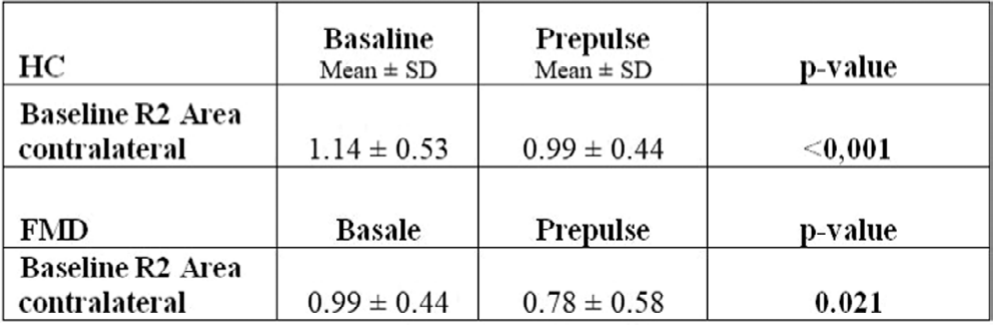
**TABLE 1** Comparison analysis on Blink reflex across Baseline and Prepulse condition both Healthy Control (HC) and Functional Movement Disorders (FMD) subjects. Mean  ± SD with corresponding *p*‐values. Significant results are shown in bold.

**Figure:**
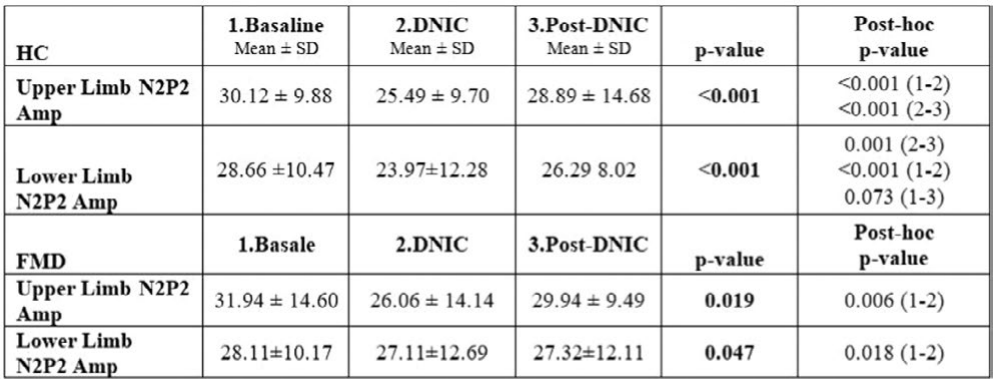
**TABLE 2** Comparison analysis on laser‐evoked potentials across Baseline (1), during Diffuse Noxious Inhibitory Controls (DNIC) (2), and post‐DNIC (3) both Healthy Control (HC) and Functional Movement Disorders (FMD) subjects. Mean  ± SD with corresponding.

**Conclusion:** Preliminary results reveal distinct motor and exteroceptive biomarkers in FMD, offering insights into mechanisms and diagnostic tools. Findings support multimodal assessments to improve FMD diagnosis.

**Disclosure:** This research funded by the European Union – Next Generation EU (NRRP M6C2 – Investment 2.1, PNRR‐MAD‐2022‐12376826; title of scientific research “A window into the mind‐brain‐body interplay: development of diagnostic, prognostic biomarkers and rehabilitation strategies in functional motor disorders”). Collaborators: S. Mariotto, S. Tamburin, M. Fiorio, C. Vinciguerra, A. Botto, L. Zenere, E. Canu, E. Sibilla, M. Filippi, E. Sarasso, C. Geroin, M. Fasoli, A. Marotta, E. Pizzolla, F. Salaorni, I. Lozzi, F. Bombieri, G.M.Squintani, G. De Biasi, G. Piscosquito, M. Amboni, G., Mansueto, M. Barillari, F.B. Pizzini, M.F. Lauriola, M.C. Tozzi, F. Rusciano, A. Paolicelli, G. Pedrotti, F. Agosta.

## MS and related disorders 2

## EPR‐161

### Anti‐BCMA CAR T cell therapy in patients with multiple sclerosis

#### C. Qin ^1^; M. Dong^1^; L. Zhou^1^; Shang^1^; J. Xiao^1^; L. Zhu^2^; H. Ye^3^; S. Cai^3^; D. Wang^2^; B. Bu^1^; G. zu Hörste^4^; C. li^2^; D. Tian^1^; W. Wang^1^

##### ^1^Department of Neurology, Tongji Hospital, Tongji Medical College, Huazhong University of Science and Technology, Wuhan, China; ^2^Department of Hematology, Tongji Hospital, Tongji Medical College, Huazhong University of Science and Technology, Wuhan, China; ^3^Nanjing IASO Biotherapeutics Ltd, Nanjing, China; ^4^Department of Neurology with Institute of Translational Neurology, University Hospital Münster, Münster, Germany

**Background and aims:** B cells and neuroinflammation within the central nervous system (CNS) contribute to disease progression in multiple sclerosis (MS) (Correale et al., 2017). Eque‐cel is a fully human B‐cell maturation antigen (BCMA) targeted chimeric antigen receptor (CAR) T‐cell therapy. This study investigates the potential of anti‐BCMA CAR T cell therapy in treating three patients with MS.

**Methods:** Autologous T cells were collected and enriched from patients with MS. Eque‐cel was generated by transducing these T cells with a lentiviral vector encoding a fully human anti‐BCMA CAR. As of December 31, 2024, three progressive MS patients received a three‐day consecutive lymphodepletion regimen and 1.0×10^6 total CAR T cells/kg. All MS related medications were discontinued the day before lymphodepletion therapy.

**Results:** Baseline patient characteristics are summarized in Table 1. CAR T cells rapidly reached peak expansion by day 10, with flow cytometry showing significant B cell depletion post infusion (Figure 1). All patients experienced transient grade 1 cytokine release syndrome (CRS). No ICANS or other neurologic toxicities. Functional improvement were noted, including better Expanded Disability Status Scale (EDSS) scores, reduced times on the Nine‐Hole Peg Test and Timed 25‐Foot Walk test, and resolution of oligoclonal bands (OCBs) in cerebrospinal fluid (CSF) (Figure 2).

**Figure:**
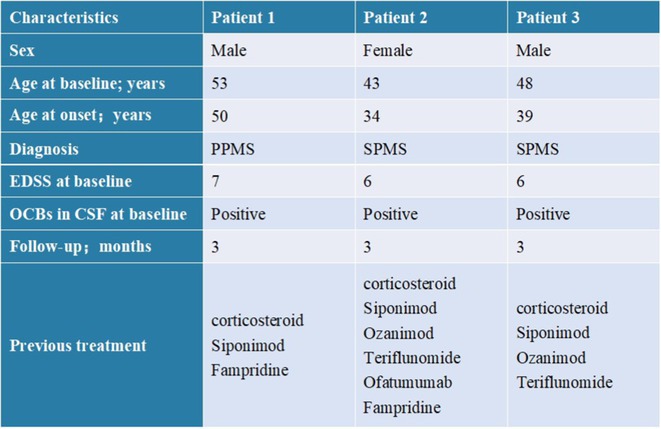
**TABLE 1** Demographic and clinical characteristics. PPMS: primary progressive multiple sclerosis. SPMS: Secondary progressive multiple sclerosis. EDSS: expanded disability status scale. OCBs: Oligoclonal bands.

**Figure:**
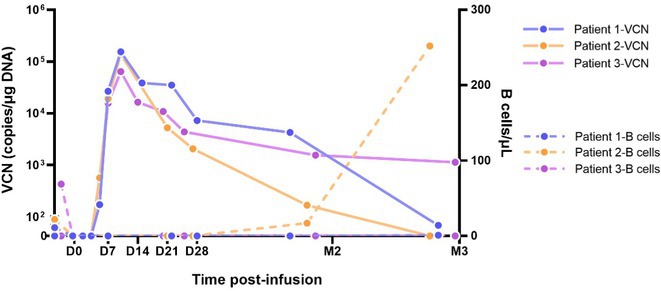
**FIGURE 1** Vector copy numbers (VCN) and B cells following CAR‐T cell therapy.

**Figure:**
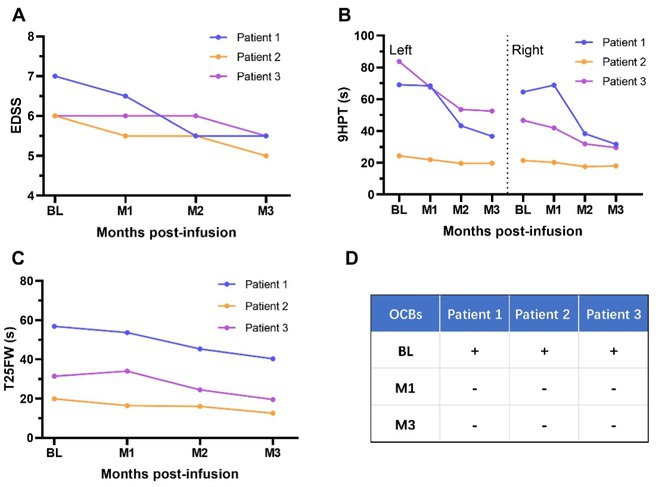
**FIGURE 2** Clinical evaluation and Oligoclonal bands. (A) EDSS scores of the patient before and after CAR‐T cell therapy. (B) Results of Nine‐Hole Peg Test (9HPT) evaluation. (C) Results of Timed 25‐Foot Walk (T25FW) evaluation. (D) Oligoclonal bands (OCB).

**Conclusion:** Anti‐BCMA CAR T cells are well tolerated and show high efficacy in treating progressive MS, as demonstrated by improved physical function and resolution of OCBs in CSF.

**Disclosure:** Huan Ye and Song‐Bai Cai are employees of Nanjing IASO Biotechnology Co., Ltd.

## EPR‐162

### Ocrelizumab subcutaneous: Updated analyses at week 72 and PATIENT PREferences from the OCARINA II study

#### D. Centonze ^1^; D. Centonze^2^; L. Goldstick^3^; K. Selmaj^4^; K. Selmaj^5^; E. Krzystanek^6^; D. Zecevic^7^; C. Figueiredo^7^; S. Clinch^8^; C. Giacobino^7^; J. Azmi^7^; S. Newsome^9^

##### ^1^Department of Systems Medicine, Tor Vergata University, Rome, Italy; ^2^Unit of Neurology, IRCCS Neuromed, Pozzilli (IS), Italy; ^3^Department of Neurology and Rehabilitation Waddell Center for Multiple Sclerosis, University of Cincinnati, College of Medicine, Cincinnati, OH, USA; ^4^Centrum Neurologii, Łódź, Poland; ^5^Department of Neurology, University of Warmia & Mazury, Olsztyn, Poland; ^6^Department of Neurology, School of Health Sciences in Katowice, Medical University of Silesia, Katowice, Poland; ^7^F. Hoffmann‐La Roche Ltd, Basel, Switzerland; ^8^Roche Products Ltd, Welwyn Garden City, UK; ^9^Johns Hopkins University School of Medicine, Baltimore, MD, USA

**Background and aims:** OCARINA II (NCT05232825) showed ocrelizumab (OCR) subcutaneous (SC) 920 mg (co‐formulated with recombinant human hyaluronidase PH20 [rHuPH20]) has a similar benefit–risk profile to OCR intravenous (IV) 600 mg in people with relapsing and primary progressive multiple sclerosis (PwRMS/PwPPMS). Updated safety and clinical data, and new patient‐reported outcomes (PROs) are presented at clinical cut‐off date (CCOD: up to September 2024).

**Methods:** OCR‐naive PwRMS/PwPPMS (18–65 years; Expanded Disability Status Scale [EDSS] score: 0–6.5) were randomized 1:1 to OCR IV 600 mg (OCR IV/SC) or OCR SC 920 mg (OCR SC/SC). At Week (W)24, all patients received OCR SC up to W96. Endpoints reported include EDSS, safety and PROs (Multiple Sclerosis Treatment Preference Questionnaire [MSTPQ] and Patient Preference Questionnaire [PPQ]).

**Results:** Mean (SD) changes in EDSS scores from baseline were –0.14 (0.77) and –0.06 (0.80) in OCR IV/SC and SC/SC, respectively. Safety data from patients who received ≥1 OCR SC dose are shown in Table 1. Injection reactions (IRs) were the most common adverse event. All IRs were non‐serious and mild/moderate in intensity; 99.4% resolved. At the CCOD, no treatment‐emergent antidrug antibodies to OCR were reported; one patient experienced treatment‐emergent anti‐rHuPH20 antibodies. At W48, the MSTPQ showed that 98.2% were satisfied/very satisfied with OCR SC; among 139 patients who previously received other disease‐modifying therapies, 82.0% preferred OCR. Per the PPQ, 80.4% of patients who received both OCR IV and SC preferred SC administration.

**Conclusion:** Ocrelizumab SC continues to show similar clinical and safety profiles to ocrelizumab IV. Patients showed stronger preferences for ocrelizumab SC.

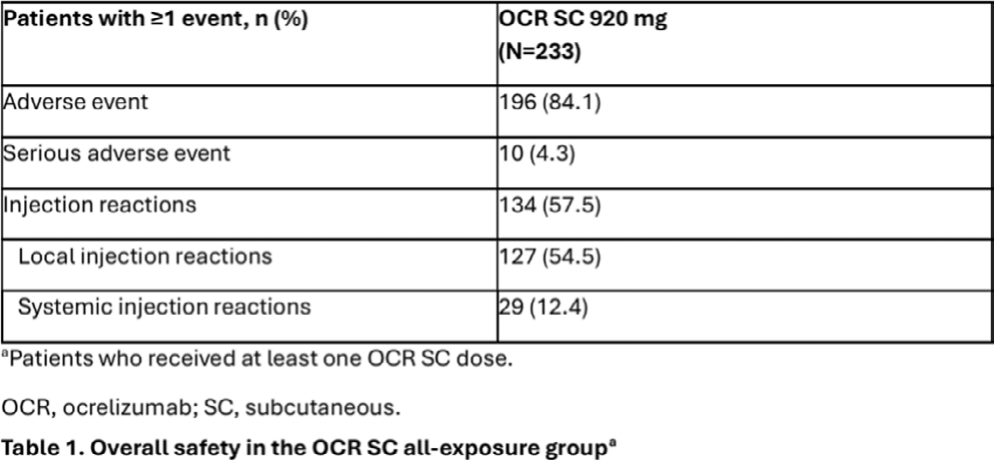

**Disclosure:** Sponsored by Roche; editorial assistance by Nucleus Global. DC ad board, honoraria/consulting (Alexion, Almirall, Amicus, Bayer, Biogen, BMS, Celgene, Chiesi, GW Pharmaceuticals, Horizon, Janssen, Lundbeck, Merck‐Serono, Novartis, Roche, Sandoz, Sanofi‐Genzyme, Viatris, Teva); study lead PI (Biogen, BMS, Merck‐Serono, Mitsubishi, Novartis, Roche, Sanofi‐Genzyme, Actelion); grant (Bayer Schering, BMS, Biogen, Celgene, Lundbeck, Merck‐Serono, Novartis, Roche, Sanofi‐Genzyme Teva). LG consulting (EMD Serono, BMS, Biogen, Sanofi‐Genzyme Roche/Genentech); consulting, compensation (TG Therapeutics); funding (Biogen, Roche/Genentech, Sanofi‐Genzyme). KS honoraria, consulting, ad board (Merck, Novartis, Roche, Biogen, Celgene, BMS, TG Therapeutics). EK consulting, ad board (Biogen, Merck‐Serono, Bayer, Roche, Novartis, Polish MS Society); study lead PI (Roche, TG Therapeutics, Merck, Biogen, Lundbeck, Janssen clinical trial programs); compensation (Biogen, Bayer, Novartis, UCB, Roche, Merck‐Serono, Teva, Lundbeck, Pfizer, Sandoz, Sanofi‐Genzyme). DZ, CF, SC, CG, JA employees & shareholders (Roche). SDN consulting, ad board (Biogen, Genentech, Inc., BMS, Novartis TG Therapeutics); study lead PI (Roche); funding (Biogen, Lundbeck, Roche, Genentech, Inc., Sanofi, National MS Society, The Stiff Person Syndrome Research Foundation, Department of Defense, Patient‐Centered Outcomes Research Institute).

## EPR‐163

### Clinico‐demographic and radiological features associated with sleep disorders and multiple sclerosis

#### G. Corsini ^1^; A. Spiezia^1^; G. Pontillo^4^; F. Falco^1^; M. Eliano^1^; F. Lamagna^1^; A. Esposito^1^; C. Di Monaco^1^; V. Nicolella^1^; F. Novarella^1^; M. Moccia^1^; S. Cocozza^2^; A. Brunetti^2^; M. Petracca^3^; V. Brescia Morra^1^; R. Lanzillo^1^; A. Carotenuto^1^

##### ^1^Multiple Sclerosis Unit, Federico II University Hospital of Naples, Naples, Italy; ^2^Department of Advanced Biomedical Sciences, Federico II University Hospital of Naples, Naples, Italy; ^3^Department of Human Neurosciences, Sapienza University, Rome, Italy; ^4^MS Center Amsterdam, Radiology and Nuclear Medicine, Vrije Universiteit Amsterdam. Amsterdam Neuroscience, Amsterdam UMC location VUmc, Amsterdam, The Netherlands

**Background and aims:** People with Multiple Sclerosis (MS) often experience sleep disorders, usually resulting from a combination of physical and psychological factors as well as from cerebral damages. In this study we aimed to analyze the correlation between sleep impairment and clinical, neuropsychological and radiological findings.

**Methods:** This cross‐sectional study included 400 MS patients. Neuropsychological assessments included Brief International Cognitive Assessment for MS (BICAMS), Beck Depression Inventory (BDI‐II) and Modified Fatigue Impact Scale (MFIS) and BAI. Sleep Impairment was assessed through the PSQI (score > 5 indicating impairment). A forward stepwise multivariable linear regression model evaluated the association between PSQI scores and clinical features and between PSQI scores and radiological features using age, gender, BDI‐II, BAI and MFIS as correcting factors.

**Results:** Among the study population 181 MS patients showed cognitive impairment, and 90 MS patients showed sleep impairment. Sleep impairment was linked to greater depressive symptoms (OR = 1.10, *p* < 0.001) and higher EDSS (OR = 1.30, *p* = 0.02). Furthermore, PSQI scores correlated with increased bilateral hippocampal and nucleus pallidus volume (corr. coeff. = 2.04, *p* < 0.001 and corr. coeff. = 3.15, *p* = 0.02, respectively), higher T2 Lesion Volume (corr. coeff. = 0.26, *p* < 0.001) and reduced bilateral amygdala and caudate nucleus (corr. coeff. = ‐2.34, *p* = 0.03; corr. coeff. = ‐1.49, *p* = 0.05).

**Conclusion:** Motor and depressive symptoms in MS patients may affect sleep through different manifestations such as muscle spasms, cramps and increased anxiety. At the same time sleep impairment may result from imbalanced structural alterations in subcortical gray matter, suggesting disrupted connectivity between subcortical structures.

**Disclosure:** A.E. has received honoraria from Novartis. M.M. has received research grants from ECTRIMS‐MAGNIMS, the UK MS Society, and Merck, and honoraria from Biogen, BMS Celgene, Ipsen, Janssen, Merck, Novartis, Roche, and Sanofi‐Genzyme. M.P. has received research grants from the Italian MS Foundation and Baroni Foundation, honoraria from Health & Life and Biogen, and sponsorship for travel/meeting expenses from Novartis, Roche, and Merck. S.C. received personal fees from Shire and Genzyme, outside the submitted work. R.L. has received honoraria from Biogen, Merck, Novartis, Roche, and Teva. V.B.M. has received research grants from the Italian MS Society and Roche, and honoraria from Bayer, Biogen, Merck, Mylan, Novartis, Roche, Sanofi‐Genzyme, and Teva. A.C. has received research grants from Almirall, research grants from ECTRIMS‐MAGNIMS, and honoraria from Almirall, Biogen, Roche, Sanofi‐Genzyme, Merck, Ipsen, and Novartis. None of the other authors has any conflict of interest to disclose.

## EPR‐164

### Predicting disability level in multiple sclerosis using MSCopilot®: A real‐world post‐market surveillance study

#### L. Carment ^1^; P. Drouin^1^; N. Sellami^1^; L. Pillet^1^; S. Zinaï^1^; A. Tourbah^2^

##### ^1^Ad Scientiam, Paris, France; ^2^Service de Neurologie, Hôpital Raymond‐Poincaré AP‐HP, Garches, Université Versailles‐Saint‐Quentin‐en‐Yvelines, Inserm U1195, Université Paris‐Saclay, France

**Background and aims:** This real‐world post‐market surveillance study evaluates the ability of MSCopilot®, a clinically validated software‐as‐a‐medical‐device, to differentiate disability levels, as measured by the Expanded Disability Status Scale (EDSS), in a French cohort of patients with multiple sclerosis (PwMS).

**Methods:** Digital biomarkers, self‐reported socio‐demographic and clinical parameters, were collected using MSCopilot® in a subgroup of adult PwMS who provided informed consent (October 2017 to September 2023). MSCopilot® was used at home without supervision to assess walking capacity, low‐contrast visual acuity, cognitive processing, and dexterity. Disability levels were categorized as low to mild (EDSS < 4) or moderate to severe (EDSS ≥4).

**Results:** Among 590 PwMS included, the majority (71%, n = 421) reported an EDSS < 4. In this group, participants were younger (mean age = 40  ± 11 years) and had shorter disease duration (mean duration since diagnosis = 7  ± 8 years) compared to the EDSS ≥4 group (mean age = 48  ± 12, disease duration = 14  ± 10; *p* < 0.001, for both). Significant differences were observed with decreased performance in walking capacity, cognitive function, and dexterity in the EDSS ≥4 group (all *p*‐values < 0.01). The digital walking test demonstrated strong discriminatory ability between EDSS groups, with an AUC of 0.8 (sensitivity = 0.86, specificity = 0.71) in univariate logistic regression. Multivariate regression improved sensitivity but did not enhance discriminatory power.

**Conclusion:** This pioneering real‐world study demonstrates the feasibility of using MSCopilot® to support clinicians in making informed decisions about their patients' MS‐related disability, between in‐person routine visits, using reliable real‐world data.

**Disclosure:** L. Carment, P. Drouin, N. Sellami, L‐E. Pillet, S. Zinaï are employees of Ad Scientiam, A. Tourbah is a member of Ad Scientiam scientific committee and received honoraria for lectures, travel grants and research support from Biocara, Hikma, Novartis, Roche.

## EPR‐165

### Clinical integration of brain and cord MRI features improves differential diagnosis of multiple sclerosis

#### M. Rocca ^1^; S. Ratzinger^2^; P. Preziosa^1^; A. Meani^3^; M. Gueye^2^; P. Vezzulli^4^; E. Pagani^3^; F. Esposito^5^; A. Giordano^6^; B. Colombo^5^; A. Falini^7^; M. Filippi^8^

##### ^1^Neuroimaging Research Unit, Division of Neuroscience, and Neurology Unit, IRCCS San Raffaele Scientific Institute, and Vita‐Salute San Raffaele University, Milan, Italy; ^2^Neuroimaging Research Unit, Division of Neuroscience, and Neurology Unit, IRCCS San Raffaele Scientific Institute, Milan, Italy; ^3^Neuroimaging Research Unit, Division of Neuroscience, IRCCS San Raffaele Scientific Institute, Milan, Italy; ^4^Neuroradiology Unit and High Field MRI Center, IRCCS San Raffaele Scientific Institute, Milan, Italy; ^5^Neurology Unit, Division of Neuroscience, IRCCS San Raffaele Scientific Institute, Milan, Italy; ^6^Neurology Unit, Division of Neuroscience, IRCCS San Raffaele Scientific Institute, and Vita‐Salute San Raffaele University, Milan, Italy; ^7^Neuroradiology Unit and High Field MRI Center, IRCCS San Raffaele Scientific Institute, and Vita‐Salute San Raffaele University, Milan, Italy; ^8^Neurology Unit, Neurorehabilitation Unit, Neurophysiology Service, and Neuroimaging Research Unit, Division of Neuroscience, IRCCS San Raffaele Scientific Institute, and Vita‐Salute San Raffaele University, Milan, Italy

**Background and aims:** To explore the role of brain and spinal cord MRI features in differentiating patients with suspected central nervous system (CNS) inflammatory diseases.

**Methods:** Prospective data from 125 patients undergoing diagnostic evaluation, including 1.5T brain and spinal cord MRI scans from February 2021 and March 2024 were analyzed. The cohort comprised 91 patients with multiple sclerosis (MS), 15 with other inflammatory neurological diseases (OIND), and 19 with non‐inflammatory neurological diseases (NIND). Brain and spinal cord lesion topographies and morphological features were evaluated to identify MRI features discriminating MS from OIND and NIND.

**Results:** Random forest analysis identified key MRI features supporting MS diagnosis over OIND: absence of longitudinally extensive transverse myelitis (relative importance [RI] = 100%), presence of > = 1 Dawson's finger (RI = 55.3%), > = 1 cortical lesion (RI = 42.6%), and > = 1 brain T2‐hyperintense white matter (WM) lesion (RI = 36.4%). After excluding the presence of > = 1 brain T2‐hyperintense WM lesion, fulfilling > = 2 of the 3 selected criteria distinguished MS from OIND patients with a sensitivity of 0.59 and a specificity of 0.80. For distinguishing MS from NIND, relevant MRI features included > = 1 T2‐hyperintense spinal cord lesion (RI = 100.0%), > = 1 Dawson's finger (RI = 84.3%), > = 1 cortical lesion (RI = 61.4%), > = 1 cerebellar peduncle lesion (RI = 52.2%) and > = 3 central vein sign‐positive lesions (RI = 27.8%). Fulfilling > = 2 of the 5 selected criteria identified MS patients with a sensitivity of 0.64 and a specificity of 0.84.

**Conclusion:** Integrating novel MRI features in the diagnostic work‐up of patients with suspected CNS inflammatory disease improves differentiation between MS, OIND, and NIND, reducing the risk of misdiagnosis.

**Disclosure:** MAR consulting fees from Biogen, Bristol Myers Squibb, Eli Lilly, Janssen, Roche, and speaker honoraria from AstraZaneca, Biogen, Bristol Myers Squibb, Bromatech, Celgene, Genzyme, Horizon Therapeutics Italy, Merck Serono SpA, Novartis, Roche, Sanofi and Teva. SR, AM, MG,PV, EP, AG, AF, FE nothing. PP speaker honoraria from Roche, Biogen, Novartis, Merck, Bristol Myers Squibb, Genzyme, Horizon, Sanofi. BC travel grants or honoraria for advisory boards, speaker panels, or investigation studies from Novartis, Teva, Eli Lilly, Pfizer, and Lusofarmaco. MF consulting or speaking fees from Alexion, Almirall, Bayer, Biogen, Celgene, Chiesi Italia SpA, Eli Lilly, Genzyme, Janssen, Merck‐Serono, Neopharmed Gentili, Novartis, Novo Nordisk, Roche, Sanofi Takeda, and TEVA; Advisory Boards for Alexion, Biogen, Bristol‐Myers Squibb, Merck, Novartis, Roche, Sanofi, Sanofi‐Aventis, Sanofi‐Genzyme, Takeda; scientific direction of educational events for Biogen, Merck, Roche, Celgene, Bristol‐Myers Squibb, Lilly, Novartis, Sanofi‐Genzyme; research support from Biogen Idec, Merck‐Serono, Novartis, Roche.

## EPR‐166

### The impact of national policies on reimbursement of DMT for MS on disease outcomes: Slovenia versus Croatia

#### M. Krbot Skoric^1^; G. Brecl Jakob^2^; I. Adamec^1^; U. Rot^2^; T. Gabelić^1^; A. Horvat Ledinek^2^; M. Fatur Triller^2^; B. Barun^1^; S. Gomezelj^2^; R. Bernik^2^; M. Habek ^1^

##### ^1^University Hospital Center Zagreb, Department of Neurology, Referral Center for Autonomic Nervous System Disorders, Zagreb, Croatia; ^2^Department of Neurology, University Medical Centre Ljubljana, Ljubljana, Slovenia

**Background and aims:** The aim of this research was to determine the impact of national policies on reimbursement of disease‐modifying therapies (DMT) for multiple sclerosis (MS) on disease outcomes.

**Methods:** The population included the consecutive Slovenian and Croatian people with MS (pwMS) cohort entered in the MSBase registry database. We calculated the following DMT variables from the above information: time to first ever DMT, total DMT duration, and ever used DMT. We applied 1:1 propensity score matching to match pwMS from Slovenia and Croatia to mitigate baseline differences between the groups.

**Results:** Out of 894 pwMS from Slovenia and 1363 from Croatia, 565 pwMS from both countries were enrolled after propensity score matching. pwMS living in Croatia had statistically significantly higher levels of disability measured with EDSS compared to pwMS living in Slovenia (2.0 vs. 1.5 respectively, *p* = 0.004) (Table 1). The time to first‐ever DMT was longer in the Croatian cohort (2.76; 1.20‐6.92) than in the Slovenian cohort (1.29; 0.48‐4.60), *p* < 0.001. In a multivariable logistic regression, pwMS who lived in Croatia had an increased probability of EDSS ≥3 by 52.8% (Exp(B) 1.528, 95% C.I. 1.182‐1.974, *p* = 0.001). Furthermore, the time to first‐ever DMT increased the probability of EDSS ≥3 by 10.6% (Exp(B) 1.106, 95% C.I. 1.083‐1.129, *p* < 0.001).

**Figure:**
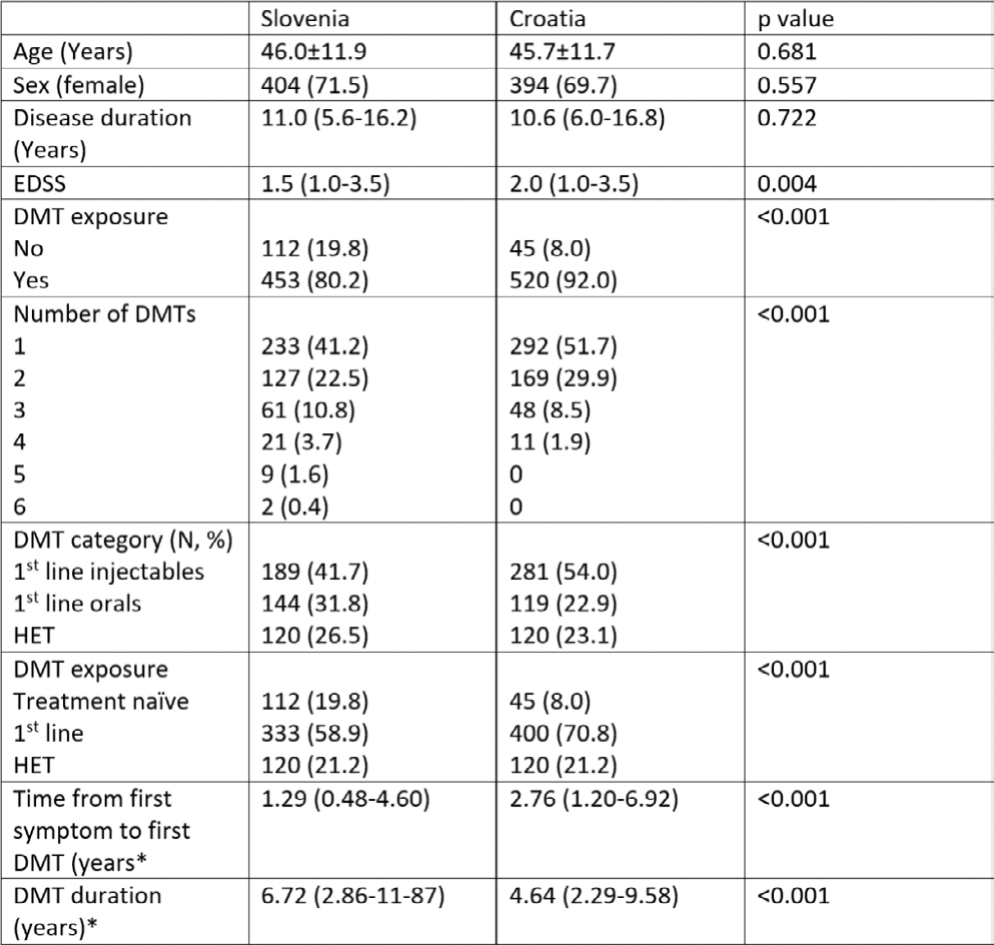
**TABLE 1** Baseline characteristics of the matched study population.

**Figure:**
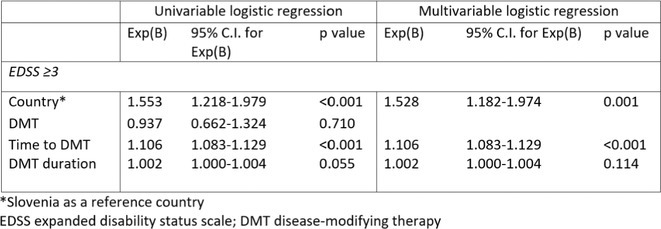
**TABLE 2** Results of the univariable and multivariable logistic regression analysis.

**Conclusion:** This study provides evidence for the impact of national policies on the reimbursement of DMTs on disability outcomes in pwMS. pwMS living in a country with less stringent reimbursement policies have a higher probability of having lower levels of disability.

**Disclosure:** GBJ: Clinical investigator and/or received consultation and/or speaker fees from: Amgen, AstraZeneca, Biogen, Janssen, Lek, Merck, Novartis, Pliva/Teva, Roche, Sanofi Genzyme, Swixx, Viatris. MKS, IA, TG, BB, MH: Clinical investigator and/or received consultation and/or speaker fees from: Biogen, Sanofi Genzyme, Merck, Novartis, Pliva/Teva, Roche, Zentiva, Actelion, Alexion Pharmaceuticals, TG Pharmaceuticals. UR: has received consultation fees/Honoraria from: Bayer, Biogen, Janssen, Lek, Merck, Novartis, Roche, Sanofi‐Genzyme, Teva and Grants/Research support from Biogen and Novartis. AHL: Clinical investigator and/or received consultation and/or speaker fees from: AstraZeneca, Biogen, Janssen, Lek, Merck, Novartis, Pliva/Teva, Roche, Sanofi Genzyme. MFT: has received speaker fees from: Biogen, Novartis, Janssen. SG: has received speaker fees from: Merck, Novartis. RB: Nothing to disclose.

## EPR‐167

### Social determinants of health and multiple sclerosis in Italy: The SocialMS study

#### M. Ponzano ^1^; I. Schiavetti^1^; A. Signori^1^; A. Bellavia^2^; D. Landi^3^; M. Sormani^1^

##### ^1^Department of Health Sciences, University of Genoa, Genoa, Italy; ^2^Harvard T.H. Chan School of Public Health, Department of Environmental Health, Boston, USA; ^3^Tor Vergata University, Multiple Sclerosis Clinical and Research Unit, Department of Systems Medicine, Rome, Italy

**Background and aims:** SocialMS is an observational cross‐sectional study on social determinants of health (SDoH) in Italian patients with multiple sclerosis (MS). Data were collected based on self‐administered surveys and started in March 2024.

**Methods:** Patients who completed sections on demographics, MS and SDoH by August 17 were included in this interim analysis. The SDoH section covered: sex, gender and sexuality, origin, education, employment, socioeconomic status, abuse, health‐care access, food, air pollution and social support. Disability was assessed by the Patient Determined Disease Steps (PDDS) scale.

**Results:** 1090 patients were included (37% North, 29% Centre, and 34% South and Islands). Median(interquartile range) PDDS was 1(0‐3); PDDS was greater for patients with financial difficulties (*p* < 0.001) and with less education (*p* = 0.001); education was related to economic and employment status (*p* < 0.001). Lower income was associated with longer months between MS symptoms and diagnosis (*p* = 0.020) and diagnostic delay was associated with disability (*p* < 0.001). Long waiting lists was a common issue for care‐access; patients who could afford private healthcare (59%) often had greater income, higher education and medical insurance (*p* < 0.001). MS itself had a negative impact on SDoH: educational (40%) and professional (45%) goals; job quit/change due to MS diagnosis (12%) or disability (16%); financial resources (33%); abuse (3%); social life (44%), relationship with partner (31%) and friends (23%). The impact of MS was stronger for more disabled patients.

**Figure:**
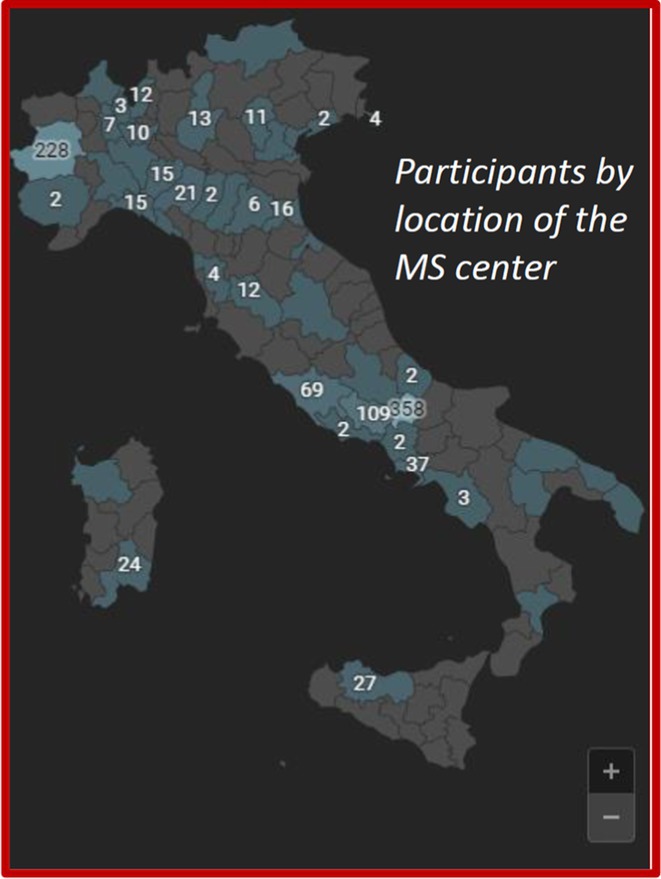
**FIGURE 1** Number of participants by location of the MS center

**Figure:**
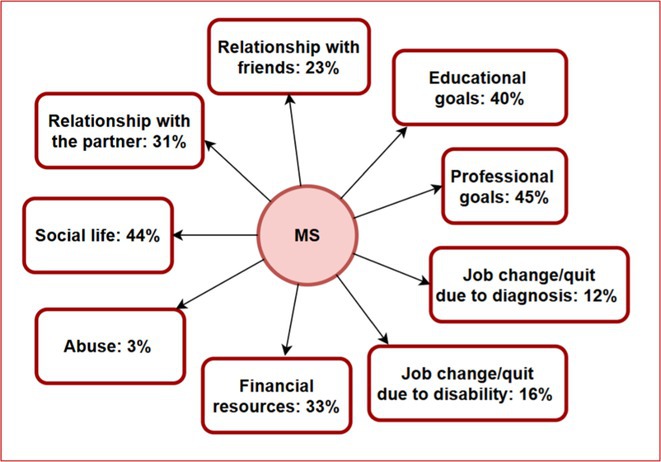
**FIGURE 2** Impact of MS

**Conclusion:** In Italy, more efforts are needed to address SDoH that drive inequities and to support more effectively MS patients.

**Disclosure:** Nothing to disclose.

## EPR‐168

### Aerobic and cognitive training effects on insular functional connectivity in progressive multiple sclerosis: CogEx trial

#### M. Albergoni ^1^; M. Rocca^2^; F. Romanò^1^; P. Valsasina^1^; R. Motl^3^; M. Amato^4^; G. Brichetto^5^; D. Boccia^6^; J. Chataway^7^; N. Chiaravalloti^8^; G. Cutter^9^; U. Dalgas^10^; J. DeLuca^8^; R. Farrell^7^; P. Feys^11^; J. Freeman^12^; M. Inglese^6^; C. Meza^13^; A. Salter^14^; B. Sandroff^8^; A. Feinstein^13^; M. Filippi^15^

##### ^1^Neuroimaging Research Unit, Division of Neuroscience, IRCCS San Raffaele Scientific Institute, Milan, Italy; ^2^Neurology Unit, and Neuroimaging Research Unit, Division of Neuroscience, IRCCS San Raffaele Scientific Institute, and Vita‐Salute San Raffaele University, Milan, Italy; ^3^Department of Kinesiology and Nutrition, University of Illinois Chicago, Chicago, IL, USA; ^4^Department NEUROFARBA, Section Neurosciences, University of Florence, and IRCCS Fondazione Don Carlo Gnocchi, Florence, Italy; ^5^Scientific Research Area, Italian Multiple Sclerosis Foundation (FISM), Genoa, Italy; ^6^Department of Neuroscience, Rehabilitation, Ophthalmology, Genetics, Maternal and Child Health, and Center of Excellence for Biomedical Research, University of Genoa, Genoa, Italy; ^7^Queen Square Multiple Sclerosis Centre, Department of Neuroinflammation, Queen Square Institute of Neurology, University College London, London, UK; ^8^Kessler Foundation, West Orange, NJ, USA; ^9^Department of Biostatistics, University of Alabama at Birmingham, Birmingham, AL, USA, ^10^Exercise Biology, Department of Public Health, Aarhus University, Aarhus, Denmark, ^11^REVAL, Faculty of Rehabilitation Sciences, Hasselt University, Diepenbeek, Belgium, ^12^Faculty of Health, School of Health Professions, University of Plymouth, Devon, UK, ^13^Department of Psychiatry, University of Toronto and Sunnybrook Health Sciences Centre, Toronto, Canada, ^14^Department of Neurology, Section on Statistical Planning and Analysis, UT Southwestern Medical Center, Dallas, TX, USA, ^15^Neurology Unit, Neurorehabilitation Unit, Neurophysiology Service, and Neuroimaging Research Unit, Division of Neuroscience, IRCCS San Raffaele Scientific Institute, and Vita‐Salute San Raffaele University, Milan, Italy

**Background and aims:** The anterior insula (AI) is crucial for cognitive attentional processes, while the posterior insula (PI) is related to somatosensory properties. CogEx trial (NCT03679468) investigated the effects of aerobic exercise (EX) and cognitive rehabilitation (CR) on cognitive impairment in progressive multiple sclerosis (MS). The aim of this study was to assess the effects of rehabilitation on resting state functional connectivity (RSFC) of AI and PI exploiting CogEx data.

**Methods:** CogEx participants were randomized to: ‘CR+EX’, ‘CR+sham EX (EX‐S)’, ‘EX+sham CR (CR‐S)’ and ‘CR‐S+EX‐S’. We selected all subjects (*n* = 87) who underwent the 12‐week intervention period and completed baseline and 12‐week physical/cognitive and RS‐fMRI assessments. RSFC of AI and PI was assessed using a seed‐based approach.

**Results:** At week‐12 compared to baseline, groups performing CR were both characterized by increased RSFC between AI and the left temporal pole, while groups performing EX were both characterized by increased RSFC between AI and the left hippocampus. Conversely, ‘CR‐S+EX‐S’ patients were characterized by decreased RSFC of AI/PI with cingulate cortex and frontoparietal regions. In the 'EX+CR‐S' group, increased RS FC between AI and left hippocampus tended to be associated with concomitant increase in California Verbal Learning Test score (*p* = 0.063). In contrast, in 'CR‐S+EX‐S' group, over time modifications of insular RSFC with cingulate and parieto‐temporal regions were associated with concomitant worsening of visuospatial memory performance (*p* < 0.047).

**Conclusion:** EX and CR modulated RSFC of anterior and posterior insular regions in patients with progressive MS. Adaptive compensatory mechanisms occurring in insular RSFC seem to support cognitive mnemonic function.

**Disclosure:** Funding. Funded by MS Society of Canada (EGID3185). Ancillary funding: CMSC, Danish MS Society, US MS Society.

## EPR‐169

### Comparison of different age‐derived cut‐offs for plasma neurofilament light chain in multiple sclerosis

#### V. Nicolella ^1^; M. Varelli^2^; S. Fasano^2^; R. Sirica^3^; C. Polito^3^; A. Fasano^3^; M. Fiorenza^3^; F. Novarella^1^; D. Ranucci^1^; A. Carotenuto^1^; M. Petracca^4^; R. Lanzillo^1^; V. Brescia Morra^1^; G. Castaldo^5^; D. Terracciano^3^; M. Moccia^5^

##### ^1^Department of Neuroscience, Reproductive Science and Odontostomatology, Federico II University of Naples, Naples, Italy; ^2^Istituto Diagnostico Varelli, Naples, Italy; ^3^Department of Translational Medical Sciences, Federico II University of Naples, Naples, Italy; ^4^Department of Human Neuroscience, Sapienza University of Rome, Rome, Italy; ^5^Department of Molecular Medicine and Medical Biotechnology, Federico II University of Naples, Italy

**Background and aims:** Clinical use of blood neurofilament light chain (NfL) requires cut‐off values that reflect disease status independently of confounding factors, such as age, hemodilution and cardiovascular risk factors. We compared the performance of different previously‐suggested cut‐offs in separating MS cases and controls, and in identifying different MS clinical features, across age groups.

**Methods:** In this cross‐sectional study, we included people with MS (*n* = 312) and age, sex and eGFR‐matched controls (*n* = 236). For MS cases, we collected descriptors of disease progression (relapsing or progressive), EDSS, and evidence of disease activity in the previous year (including relapses, active MRI, and EDSS progression). Plasma NfL (pNfL) was evaluated using Lumipulse™ fully automated chemiluminescent enzyme immunoassay. We classified MS cases and controls based on three suggested cut‐offs derived from pNfl and age.

**Results:** In individuals aged 18‐50 years, pNfL was able to discriminate MS cases and controls (AUC = 0.73; 95%CI = 0.67, 0.78; *p* = 0.028) with high specificity (> 85%): In the MS population pNfL was able to discriminate relapsing and progressive cases (AUC = 0.70; 95%CI = 0.63, 0.77; *p* = 0.034), patients with EDSS≥4.0 and EDSS < 4.0 (AUC = 0.69; 95%CI = 0.63, 0.76; *p* = 0.032), and patients with EDSS≥6.0 and EDSS < 6.0 (AUC = 0.70; 95%CI = 0.62, 0.78; *p* = 0.040), with high sensitivity (> 75%). Different cut‐offs provided similar sensitivity and specificity, but the accuracy decreased in older age groups.

**Conclusion:** Previously‐validated cut‐offs provided similar sensitivity, specificity and accuracy in separating MS cases and controls and in identifying MS clinical features across different age groups, with the best performance before 50 years.

**Disclosure:** Valerio Nicolella discloses travel/meeting expenses from Alexion. Antonio Carotenuto has received research grants from Almirall and ECTRIMS‐ MAGNIMS and honoraria from Almirall, BMS Celgene, Biogen, Roche, Sanofi‐Genzyme, Merck, Ipsen and Novartis. Maria Petracca discloses travel/meeting expenses from Novartis, Janssen, Roche, Merck and Alexion; speaking honoraria from HEALTH&LIFE S.r.l., AIM Education S.r.l., Biogen, Novartis and FARECOMUNICAZIONE E20; honoraria for consulting services and advisory board participation from Biogen; research grants from Baroni Foundation and the Italian Ministry of University and Research (PRIN 2022LP5X2E). Vincenzo Brescia Morra and Roberta Lanzillo received research grants from the Italian MS Society, and Roche, and honoraria from Bayer, Biogen, BMS Celgene, Merck, Mylan, Novartis, Roche, Sanofi‐Genzyme, and Teva Marcello Moccia is editorial board member of Neurology (AAN, MN, US), and the Multiple Sclerosis Journal (Sage, UK); has received research grants from MUR PNRR Extended Partnership (MNESYS no. PE00000006, DHEAL‐COM no. PNC‐E3‐2022‐23683267), ECTRIMS‐MAGNIMS, UK MS Society, and Merck; and has received honoraria from Abbvie, Biogen, BMS Celgene, Ipsen, Janssen, Merck, Novartis, Roche, Sanofi‐Genzyme.

## Muscle and neuromuscular junction disorder 2

## EPR‐170

### Differential effect of Eculizumab and Efgartigimod on subscores of the MG‐ADL and QMG in generalized Myasthenia Gravis

#### A. Sarnataro ^1^; A. Bonfini Rendina^1^; A. Marsili^1^; G. Puorro^1^; F. Saccò^2^

##### ^1^Neurscience, Reproductive and Odontostomatological Sciences (NSRO) Department, Federico II University, Naples, Italy; ^2^GENESIS Department, Federico II University, Naples, Italy

**Background and aims:** Eculizumab and Efgartigimod are both approved for the treatment of generalized Myasthenia Gravis. Objective of our study is to describe the differential response of both treatments on subscores of the MG‐ADL, and QMG scale in a real‐life setting.

**Methods:** We included patients receiving either ECU or EFGA and retrospectively collected data on the MG‐ADL and QMG. We limited the observation of MG‐ADL to weekly scores for the first 8 weeks, and of QMG at baseline and every 12 weeks.

**Results:** We enrolled 38 patients, 22 treated with ECU and 16 with EFGA. We found a higher response to Eculizumab at MG‐ADL, with significant difference at week 7 and for QMG with significant difference at week 24, 36 and 48. We then compared the response to both treatments at subscores of MG‐ADL and QMG and found no difference for the ocular and limb subscores. We found a significant treatment difference at the bulbar subscores both at the MG‐ADL and QMG, with a higher drop for Eculizumab treated patients at MG‐ADL at week 6 and 7, and for Eculizumab at QMG at week 12 and 36. Mean QMG score for the forced vital capacity (FVC) decreased more with Eculizumab throughout the entire observation period.

**Figure:**
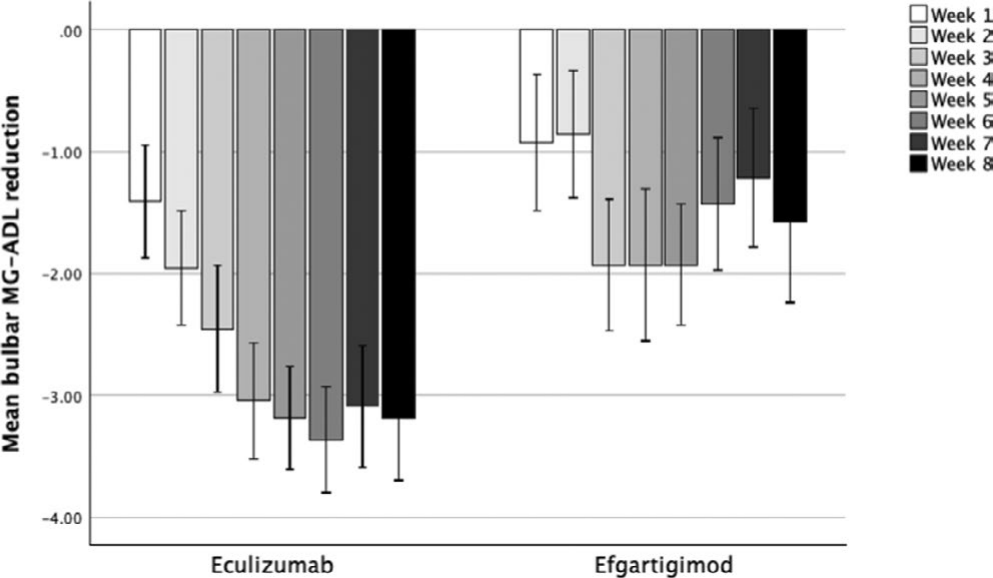
**FIGURE 1** MG‐ADL bulbar subscore

**Figure:**
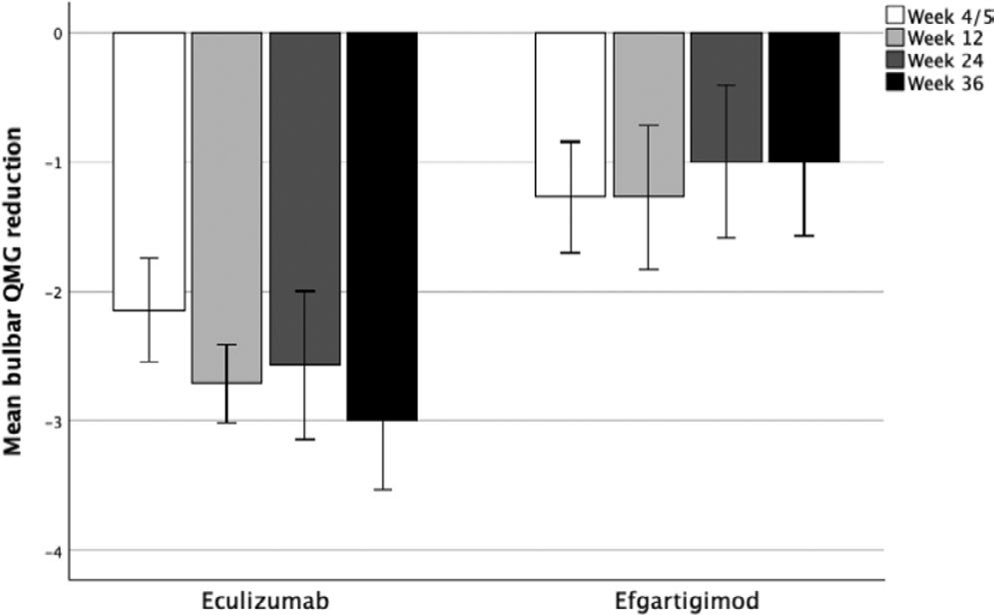
**FIGURE 2** QMG bulbar subscore

**Figure:**
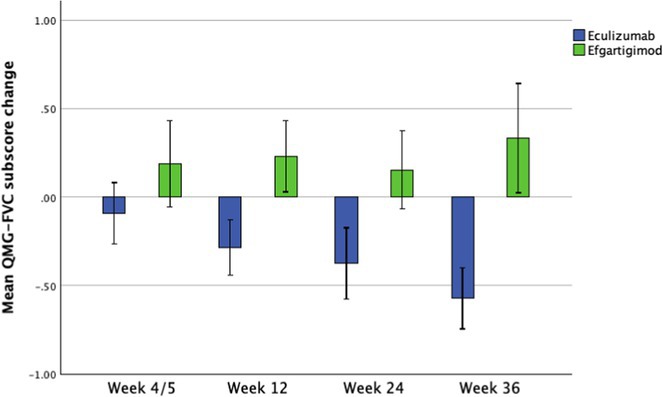
**FIGURE 3** FVC comparison

**Conclusion:** Our study shows a differential effect of Eculizumab and Efgartigimod on the MG‐ADL and QMG with a deeper effect of Eculizumab on bulbar scores. This differential effect should be considered when treating patients with high bulbar scores and ventilatory insufficiency as they may benefit more from Eculizumab.

**Disclosure:** Nothing to disclose.

## EPR‐171

### Observed efficacy of efgartigimod in generalized myasthenia gravis across patient subgroups in the ADAPT‐SC+ study

#### A. Meisel ^1^; J. De Bleecker^2^; A. Kostera‐Pruszczyk^3^; S. Muppidi^4^; T. Vu^5^; K. Heerlein^6^; R. Kerstens^6^; K. Utsugisawa^7^

##### ^1^Department of Neurology and Neuroscience Clinical Research Center, Charité – Universitätsmedizin Berlin, Berlin, Germany; ^2^Ghent University Hospital, Ghent, Belgium; ^3^Department of Neurology, Medical University of Warsaw, Warsaw, Poland; ^4^Stanford Health Care, Palo Alto, California, USA; ^5^Department of Neurology, University of South Florida Morsani College of Medicine, Tampa, Florida, USA, ^6^argenx, Ghent, Belgium; ^7^Department of Neurology, Hanamaki General Hospital, Hanamaki, Japan

**Background and aims:** Efgartigimod is a human immunoglobulin G1 (IgG1) antibody Fc fragment that reduces total IgG levels (including pathogenic autoantibodies) through neonatal Fc receptor blockade. In the ADAPT‐SC study, subcutaneous (SC) efgartigimod PH20 (coformulated with recombinant human hyaluronidase PH20) showed noninferior total IgG reduction compared with intravenous efgartigimod in participants with generalized myasthenia gravis (gMG). The objective of the current analysis was to assess efficacy of efgartigimod PH20 SC in several subgroups of participants with acetylcholine receptor antibody–positive (AChR‐Ab+) gMG (*n* = 130) during the first cycle of the open‐label extension (OLE), ADAPT‐SC+.

**Methods:** Efgartigimod PH20 SC 1000 mg was administered in cycles of 4 once‐weekly injections. Myasthenia Gravis Activities of Daily Living (MG‐ADL) scores evaluated clinical efficacy.

**Results:** The largest improvements in MG‐ADL total score (mean change [SE] from OLE baseline) were observed at week 4 of cycle 1, 1 week after last administration, (−4.1 [0.27]). Improvements were observed in multiple subgroups, including: disease duration < 3 years (−3.4 [0.62]), 3‐ < 6 years (−4.6 [0.48]), and > = 6 years (−4.1 [0.39]); aged 18‐64 years (−4.5 [0.32]) and > = 65 years (−2.5 [0.43]); MG‐ADL baseline total score 0‐4 (−1.3 [0.35]), 5‐8 (−3.2 [0.28]), and > = 9 (−6.3 [0.44]); thymectomized (−4.5 [0.40]) and nonthymectomized (−3.8 [0.37]); receiving only concomitant acetylcholinesterase inhibitors (−5.5 [0.77]), any nonsteroidal immunosuppressive treatments (−3.8 [0.38]), or any steroids (−3.8 [0.31]). Efgartigimod PH20 SC was well tolerated; no new safety signals observed.

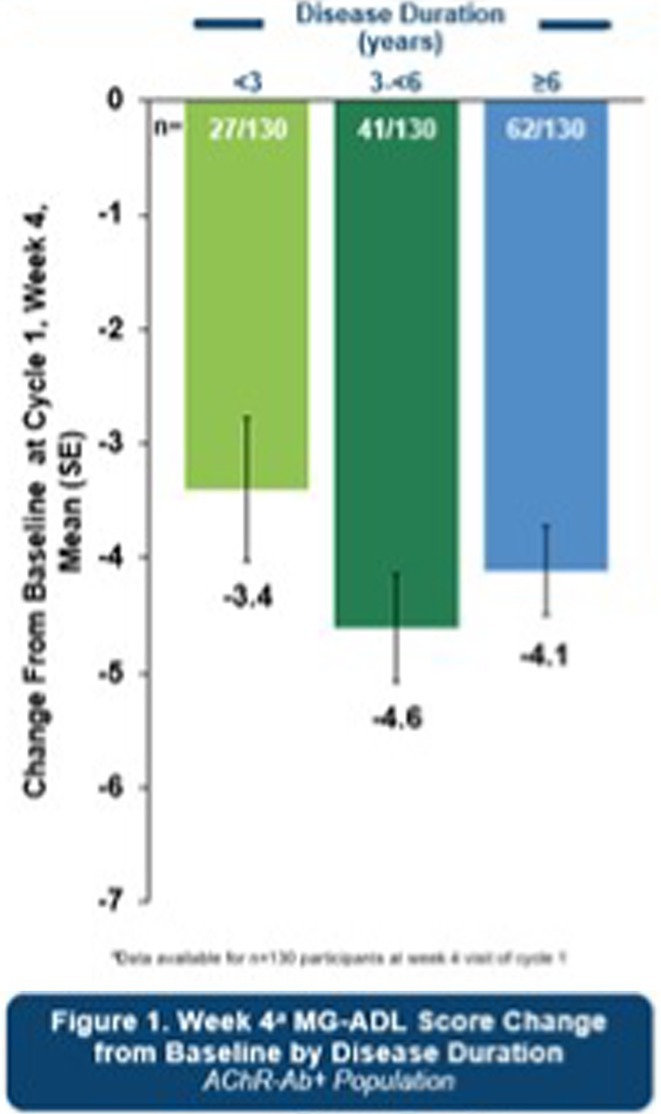

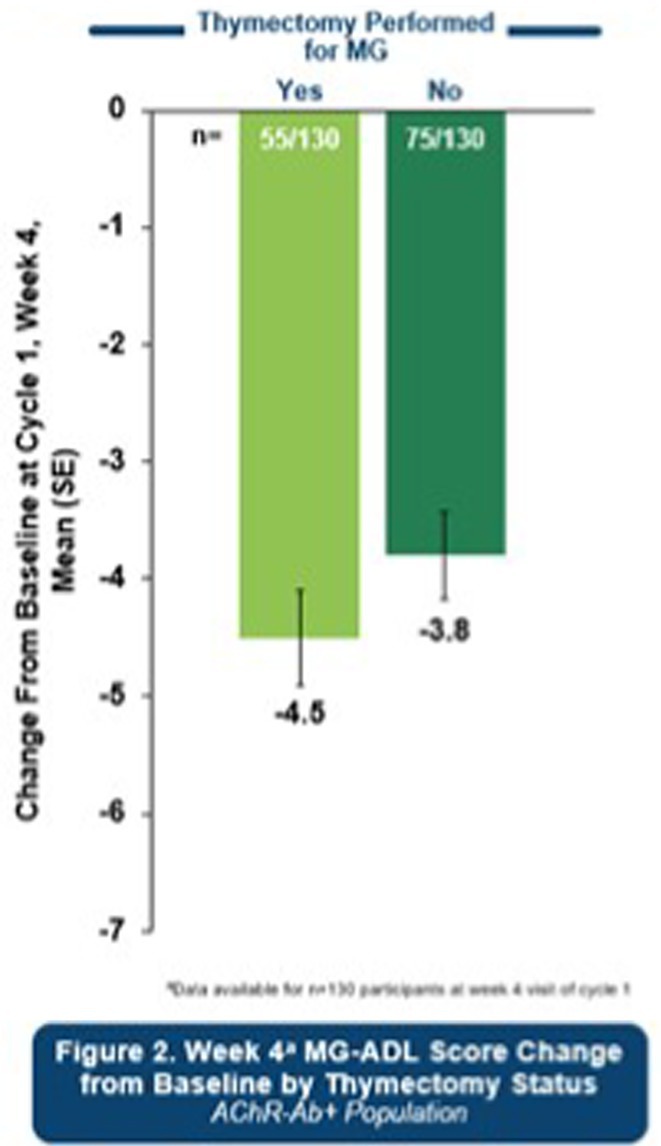

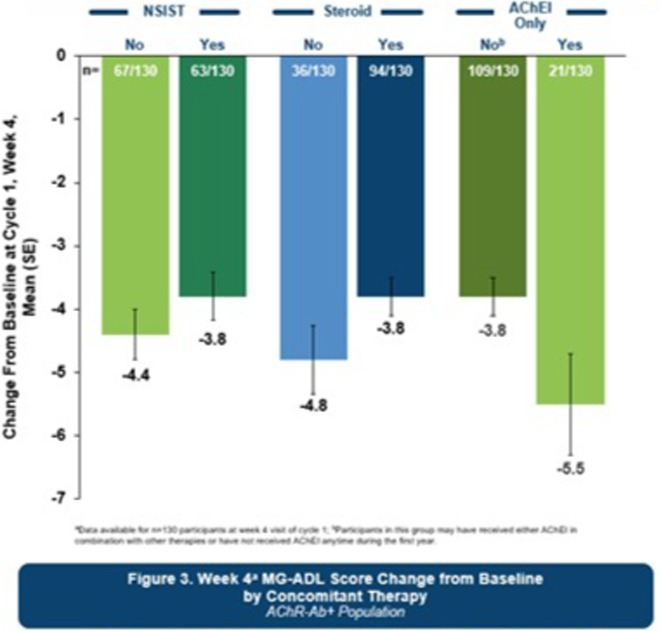

**Conclusion:** Efgartigimod PH20 SC resulted in consistent clinical improvements in AChR‐Ab+ participants across various subgroups, reinforcing the efficacy of efgartigimod PH20 SC across a broad gMG population.

**Disclosure:** This study was sponsored by argenx; EB and RK are employees of argenx; SM, TV, KU, and AM have reported financial/nonfinancial relationships with argenx at the time of submission.

## EPR‐172

### Treatment of very‐late‐onset myasthenia gravis with Efgartigimod: A retrospect cohort study

#### C. Ma; J. Shen; Y. Zhu; R. Zhu

##### Department of Neurology, The First Affiliated Hospital of China Medical University, Shenyang, China

**Background and aims:** Given the potential for very‐late‐onset myasthenia gravis (VLOMG) patients to manifest with more severe symptoms and poor response to treatment, hence, our evaluation was conducted to ascertain the safety and efficacy of efgartigimod in the VLOMG population.

**Methods:** This was a retrospective single‐center cohort study of 42 patients with VLOMG (onset ≥65 years), MG‐ADL score, QMG score, prednisone dose, laboratory data, and adverse events were assessed at each follow‐up.

**Results:** The MG‐ADL responder (≥2 MG‐ADL scores improvement sustained for ≥4 weeks) exhibited 97.6% (41/42), and 83% demonstrated sustained response for 12 weeks follow‐up. As the mean baseline MG‐ADL scores was 9.88 ± 3.48, after 1 cycle of efgartigimod, the change of MG‐ADL reached 8.23 ± 3.62. With a mean QMG score of 13 ± 4.24 at the time of enrollment, 97% achieved clinical meaningful improvement (CMI, ≥3 decreased QMG scores) and the mean duration was 6.37 ± 5.46 days. Minimal symptom expression (MSE, MG‐ADL score of 0 or 1) was achieved in 45% patients at week 4, rising to 60% at week 8 and remaining at up to 45% at 12 weeks follow‐up. All patients were able to reduce their daily dose of steroids. None of the patients experienced serious adverse events or worsening of pre‐existing comorbidities during the study period.

**Conclusion:** With significant efficacy and clinical durability, efgartigimod showed a more pronounced response without impact on comorbidities in VLOMG patients in our study. An early escalation of therapy could achieve rapidly control of disease activity and appears to be beneficial for sustained long‐term prognosis of VLOMG.

**Disclosure:** Nothing to disclose.

## EPR‐173

### Unraveling myasthenia‐myositis overlap: Clinical insights into immune checkpoint inhibitor‐related autoimmunity

#### D. Marando ^1^; E. Rossini^1^; S. Morino^2^; L. Leonardi^1^; L. Fionda^1^; A. Lauletta^1^; M. Salvetti^3^; G. Antonini^1^; M. Garibaldi^1^

##### ^1^Neuromuscular Disease Centre, Department of Neuroscience, Mental Health and Sensory Organs (NESMOS), Sant’Andrea Hospital, Sapienza University of Rome, Rome, Italy; ^2^Neuromuscular and Rare Disease Centre, Neurology Unit, Sant’Andrea Hospital, Rome, Italy; ^3^Department of Neuroscience, Mental Health and Sensory Organs (NESMOS), Faculty of Medicine and Psychology., SAPIENZA University of Rome, Via Grottarossa 1035‐1039, 00189, Rome, Italy.

**Background and aims:** Myasthenia gravis (MG) and inflammatory myopathies (IM) are autoimmune disorders affecting the neuromuscular junction and skeletal muscle fibers, respectively. The coexistence of MG and IM can pose significant diagnostic and therapeutic challenges, particularly in patients treated with immune checkpoint inhibitors (ICIs). This study aimed to compare clinical, serological, and pathological characteristics, treatment strategies, and outcomes of sporadic MG‐IM (s‐MG‐IM) and ICI‐associated MG‐IM (ICIs‐MG‐IM).

**Methods:** A retrospective, multicenter study was conducted, enrolling patients with s‐MG‐IM and ICIs‐MG‐IM. Inclusion criteria required clinical, neurophysiological, and serological confirmation of both MG and IM. Data were collected from medical records across participating centers. Clinical characteristics, laboratory findings, treatment strategies, and outcomes were statistically analyzed.

**Results:** A total of 28 patients were included: 15 with s‐MG‐IM and 13 with ICIs‐MG‐IM. ICIs‐MG‐IM patients were predominantly older males with more severe clinical manifestations, including higher rates of respiratory involvement (76.9%) and myocarditis (76.9%). Acute‐phase mortality was significantly higher in ICIs‐MG‐IM (30.8%). s‐MG‐IM patients had longer diagnostic delays and frequent relapses. Treatment outcomes varied, with s‐MG‐IM requiring prolonged corticosteroids and immunosuppressants, while ICIs‐MG‐IM patients demonstrated a more stable long‐term course after acute management.

**Conclusion:** The findings highlight distinct clinical and pathological differences between s‐MG‐IM and ICIs‐MG‐IM. Early recognition and tailored treatment strategies are critical, particularly in ICIs‐MG‐IM, where severe complications may arise. Survivors of the acute phase generally achieve stable remission without the need for chronic immunosuppressive therapy.

**Disclosure:** Nothing to disclose.

## EPR‐174

### Mortality in Myasthenia Gravis in Denmark from 1985 to 2020: A population‐based cohort study

#### J. Jul Jarbæk Nielsen; L. Levison; H. Andersen

##### Neurology, Aarhus University Hospital, Aarhus, Denmark

**Background and aims:** Myasthenia gravis (MG) is generally associated with a favorable prognosis due to progressive treatment but remains linked to elevated mortality. We determined the short‐term (< 1 year) and long‐term (1‐5 years) MG mortality compared to the general population.

**Methods:** Through nationwide health registers from 1985 to 2020, we identified MG patients and matched each patient with 10 individuals from the general population by age, sex, and diagnostic index date. We used Cox regression analysis to compute matched hazard ratios (HRs) for short‐ and long‐term mortality, stratified by sex, age, period, and baseline comorbidities. We adjusted for baseline comorbidities to assess their possible impact on MG mortality.

**Results:** Our cohort included 2,110 MG patients (1,059 females) and 21,100 general population members, with 77.3% under 75 years at index date. The short‐term cumulative mortality was 4.8% for MG patients compared to 2.6% in the general population, with an associated HR of 1.8 (95% CI 1.5‐2.3). Long‐term mortality decreased to a HR of 1.3 (95% CI 1.1‐1.5). While short‐term mortality declined from 1985 to 2014, it rose again from 2015 until 2020, particularly in patients over 75 years (HR 2.8, 95% CI 1.8‐3.6). The comorbidity‐adjusted analyses reduced short‐term mortality to a HR of 1.6 (95% CI 1.3‐2.0) and long‐term mortality became comparable to the general population.

**Conclusion:** MG remains associated with increased mortality, especially in patients over 75 years within the first year of diagnosis. Early intervention and close monitoring are indispensable to improve patient outcomes.

**Disclosure:** Nothing to disclose.

## EPR‐175

### A real‐world experience with Efgartigimod in AChR+ generalized myasthenia gravis

#### M. Baruca Grad; L. Leonardis; E. Žitnik; B. Koritnik

##### Institute of Clinical Neurophysiology, University Medical Centre Ljubljana, Slovenia

**Background and aims:** Efgartigimod(EFG) was recently approved for the treatment of acetylcholine receptor antibody positive(AChR+) generalized myasthenia gravis(gMG). Real‐world evidence is needed to fully evaluate the drug. We aimed to assess efficacy and safety of EFG in a single‐center real‐world clinical setting.

**Methods:** This was a retrospective chart review of 21 patients with anti‐AChR gMG treated with EFG at the Institute of Clinical Neurophysiology, UMC Ljubljana. The patients were followed‐up for a median time of 40 weeks and received a median of 4 cycles of EFG. Efficacy was assessed by MG‐activity of daily living(ADL), MG‐composite(MG‐C) and MG‐quality of life 15r(MG‐QoL15r) scale change, and steroid dose reduction. Data on adverse events were collected.

**Results:** EFG was selected mainly for patients who were treatment refractory, had side effects to other treatments, and/or required quick improvement in their symptoms. All patients had been previously treated with at least one medication for MG and had a median baseline MG‐ADL score of 6 points. Upon treatment, the patients’ MG‐ADL, MG‐C and MG‐QoL15r score improved by a median of 6, 5, and 10 points at 12 weeks (*p* < .001) and 4, 5 and 9 points by 24 weeks (*p* < .001). Forty‐five percent of patients achieved minimal symptom expression and 57% of patients were able to reduce the steroid daily dose ≦4mg. Adverse events were reported in 67% of patients on EFG, the most common being mild urinary tract infection.

**Conclusion:** The present study provides real‐world evidence supporting the use of EFG for the treatment of patients with refractory anti‐AChR gMG.

**Disclosure:** Mateja Baruca Grad received public speaking honoraria and consultation fees from Alexion, Astra Zeneca, Argenx and Medison Pharma.

## EPR‐176

### Quantitative muscle magnetic resonance imaging as a possible biomarker in late onset Pompe disease

#### M. Croce ^1^; L. Barzaghi^2,3^; M. Paoletti^3^; C. Bonizzoni^3^; N. Bergsland^4,5^; X. Deligianni^6,7^; F. Santini^6,7^; M. Filosto^8,9^; B. Risi^9^; L. Poli^10^; T. Mongini^11^; L. Vercelli^11^; L. Maggi^12^; S. Gasperini^13^; M. Sciacco^14^; A. Sechi^15^; M. Grandis^16,17^; M. Sacchini^18^; S. Ravaglia^19^; A. Pichiecchio^1,3^

##### ^1^Department of Brain and Behavioural Sciences, University of Pavia, Pavia, Italy; ^2^Department of Mathematics, University of Pavia, Pavia, Italy; ^3^Advanced Imaging and Artificial Intelligence Center, Neuroradiology Department, IRCCS Mondino Foundation, Pavia, Italy; ^4^Department Of Neurology, Jacobs School of Medicine and Biomedical Sciences, Buffalo Neuroimaging Analysis Center, University of Buffalo, The State University of New York, Buffalo, New York, United State; ^5^IRCCS, Fondazione Don Carlo Gnocchi Onlus, Milan, Italy ^6^Department of Radiology, University Hospital Basel, Basel, Switzerland; ^7^Basel Muscle MRI, Department of Biomedical Engineering, University of Basel, Basel, Switzerland; ^8^Department of Clinical and Experimental Sciences, University of Brescia, Brescia, Italy; ^9^NeMO‐Brescia Clinical Center for Neuromuscular Diseases, Brescia, Italy, ^10^Unit of Neurology, ASST Spedali Civili, Brescia, Italy, ^11^Neuromuscular Unit, Department of Neurosciences, University of Turin, Turin, Italy, ^12^Neuroimmunology and Neuromuscular Diseases Unit, Fondazione IRCCS Istituto Neurologico “Carlo Besta”, Milan, Italy, ^13^Department of Pediatrics, Università Degli Studi Milano‐Bicocca, Fondazione MBBM, San Gerardo Hospital, Monza, Italy, ^14^IRCCS Fondazione Ca' Granda Ospedale Maggiore Policlinico, Neuromuscular and Rare Disease Unit, Milan, Italy, ^15^Regional Coordinator Center for Rare Diseases, Academic Hospital of Udine, Udine, Italy, ^16^Università Di Genova, Genoa, Italy, ^17^IRCCS Ospedale Policlinico San Martino, Genoa, Italy, ^18^Unit of Hereditary Metabolic and Muscular Disorders, Meyer Children's University Hospital, Firenze, Italy, ^19^IRCCS C. Mondino Foundation, Pavia, Italy

**Background and aims:** Identifying biomarkers for early muscle changes and monitoring progression is critical in late onset Pompe disease (LOPD).

**Methods:** This study included 33 LOPD patients (16 asymptomatic or paucisymptomatic, Walton score ≤ 1; 17 symptomatic, Walton score ≥ 2) and 34 age and sex matched healthy controls. Thigh magnetic resonance imaging was performed using T1‐weighted and STIR sequences. Fat fraction (FF) was calculated by selecting three slices of the 6‐point Dixon images from proximal/medial/distal thigh levels to be automatically segmented into eleven regions of interest, then computed using the Fatty Riot algorithm. Clinical assessments included manual muscle testing, six minutes walking test, functional scales (Quick Motor Function Test; Gait, Stairs, Gowers, Chair Test), and patient‐reported outcomes.

**Results:** Symptomatic patients showed significantly higher thigh FF compared to asymptomatic patients and controls, with differences evident in most individual thigh muscles. While asymptomatic patients showed no overall differences in thigh FF compared to controls, the adductor magnus (AM) exhibited significantly higher FF. In fact, even in asymptomatic patients, AM was the first muscle to show fatty replacement. No clinical differences were observed between asymptomatic patients with at least one muscle exceeding 10% FF and those with none. A significant correlation was found between average thigh FF and most clinical scales.

**Conclusion:** These results highlight quantitative MRI with the Dixon method as a sensitive, objective, and non‐invasive biomarker for detecting early fat replacement in LOPD.

**Disclosure:** This project was funded with the support of the Italian Ministry of Health (RC 22‐24).

## EPR‐177

### Coexisting autoantibodies in idiopathic inflammatory myopathies: Role of antinuclear antibodies and line blot testing

#### S. Kim; Y. Choi; H. Park

##### Department of Neurology, Gangnam Severance Hospital, Yonsei University College of Medicine, Seoul, Republic of Korea

**Background and aims:** Idiopathic inflammatory myopathies (IIMs) are autoimmune disorders with diverse presentations, making diagnosis challenging. Line blot assays detect myositis‐specific antibodies (MSAs) and myositis‐associated antibodies (MAAs) but have limitations, including false results. Antinuclear antibody (ANA) testing complements these assays, improving diagnostic precision.

**Methods:** We studied 228 patients with suspected IIM, recruited from Gangnam Severance Hospital (2003–2024). Line blot assays tested 16 MSAs, and enzyme‐linked immunosorbent assays (ELISA) quantified anti‐HMGCR and anti‐NT5c1A antibodies. ANA indirect immunofluorescence (ANA‐IIF) classified nuclear speckled and cytoplasmic patterns.

**Results:** Initial diagnoses included polymyositis (169), dermatomyositis (40), and inclusion body myositis (19). Line blot assays identified ≥2 MSAs/MAAs in 59 patients (25.9%), a single MSA/MAA in 103 (45.2%), and no autoantibodies in 66 (28.9%). ANA‐IIF confirmed ≥2 MSAs/MAAs in 24 patients; anti‐Ro52 was present in 23 (95.8%), coexisting with anti‐SRP in 12 (52.2%). Combining ANA with line blot assays improved sensitivity and negative predictive value, reclassifying 80 patients as immune‐mediated necrotizing myopathy, 38 as dermatomyositis, 19 as inclusion body myositis, 12 as anti‐synthetase syndrome, and 79 as polymyositis.

**Conclusion:** Combining ANA testing with line blot assays significantly improves diagnostic accuracy in IIM, reducing false results and supporting better patient care.

**Disclosure:** Nothing to disclose.

## EPR‐178

### Muscle fiber response to mitochondrial dysfunction in mitochondrial myopathies

#### T. Bernardino Gomes ^1^; V. Di Leo^2^; C. Warren^2^; A. Khan^4^; I. Barrow^5^; D. Turnbull^3^; G. Gorman^3^; C. Lawless^3^; A. Vincent^2^

##### ^1^NHS Highly Specialised Service for Rare Mitochondrial Disorders of Adults and Children, Directorate of Neurosciences, Newcastle upon Tyne Hospitals NHS Foundation Trust, UK; ^2^Mitochondrial Research Group, Translational and Clinical Research Institute, Newcastle University, UK; ^3^Wellcome Centre for Mitochondrial Research, Translational and Clinical Research Institute, Faculty of Medical Sciences, Newcastle University, Newcastle upon Tyne NE2 4HH, UK; ^4^Centre for Doctoral Training in Cloud Computing and Big Data, Newcastle University, UK; ^5^Newcastle NIHR Biomedical Research Centre, Newcastle Upon Tyne Hospitals NHS Foundation Trust, Newcastle Upon Tyne, UK

**Background and aims:** Mitochondrial diseases affect 1 in 8,000 adults in north‐east England, often causing disabling weakness, fatigue, and exercise intolerance. The unfolded protein response (UPR) and metabolic remodeling have been associated with mitochondrial dysfunction, but their relationship remain poorly characterized in mitochondrial myopathies. We investigated protein markers across pathways of mitochondrial biology and disease to understand how muscle fibers respond to mitochondrial dysfunction at the cellular level.

**Methods:** Muscle biopsies from 19 patients with mitochondrial myopathy associated with mitochondrial DNA deletions, were studied using Imaging Mass Cytometry for 25 protein markers of UPR, oxidative phosphorylation (OXPHOS), and mitochondrial metabolism. MT‐ND4, MT‐CYB, MT‐CO1 and MT‐ATP8 were used to classify fibers as deficient or normal for OXPHOS complexes I, III‐V. Fiber classes were compared for all proteins using Generalized Linear Mixed Models (GLMM).

**Results:** GLMM analyzed 48,472 fibers, revealing significant protein fold‐change increases in OXPHOS‐deficient fibers, highest in MT‐ATP8‐deficient fibers, including TFAM (1.41), DLAT (1.65), HSPD1 (2.10), and HSPA9 (2.16); whereas C1QBP (1.31) was highest in MT‐CYB‐deficient fibers. MT‐ND4, MT‐CO1, and MT‐ATP8 deficiencies co‐occurred in 41.5% of fibers, whereas MT‐CYB deficiency was rarer, co‐occurring with others in 8%.

**Figure:**
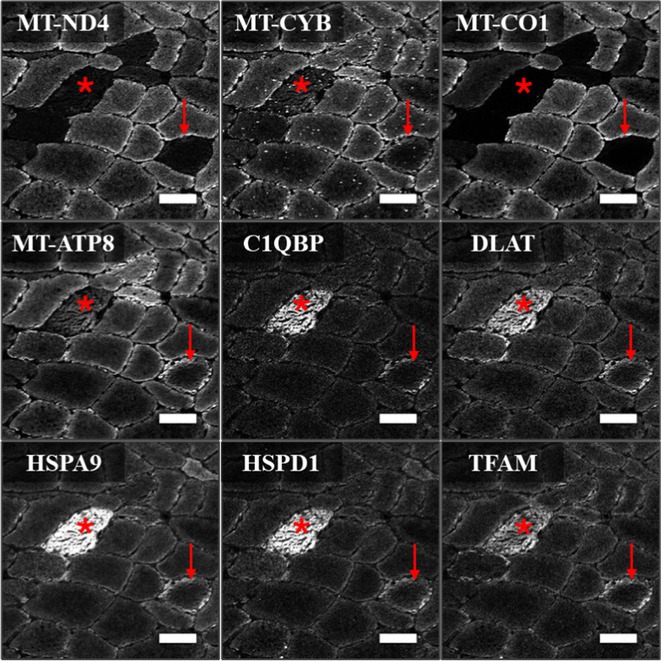
**FIGURE 1** Fibers stained for OXPHOS, DLAT, C1QBP, HSPA9, HSPD1, and TFAM. *OXPHOS deficient fiber with increased non‐OXPHOS proteins. Arrows show the same but only at fiber's periphery, suggesting peripheral origin of mitochondrial pathology. Scale bar 100 μm.

**Conclusion:** These results suggest that accumulation of OXPHOS dysfunction is associated with increased markers of mitochondrial biogenesis (TFAM), metabolic remodeling (DLAT), and mitochondrial proteostasis and UPR (C1QBP, HSPA9, and HSPD1). This study represents the most comprehensive analysis of these markers in human tissues at the single‐fiber level, helping to address the knowledge gap and informing the development of targeted, disease‐specific treatments for mitochondrial myopathy.

**Disclosure:** Nothing to disclose. This work was supported by the Wellcome Centre for Mitochondrial Research (203105/Z/16/Z), the National Institute for Health and Care Research Newcastle Biomedical Research Centre (BRC‐1215‐20005), the Newcastle upon Tyne Hospitals NHS Foundation Trust, and a Sir Henry Wellcome Fellowship (Dr A E Vincent, 215888/Z/19/Z). Additionally, the MRC Mitochondrial Disease Patient Cohort: A Natural History Study Patient Registry (REC Ref: 13/NE/0326) provide some of the phenotypical data, and the Newcastle Mitochondrial Research Biobank (REC Ref: 16/NE/0267) contributed with some of the tissues samples used in this work.

## Neurorehabilitation

## EPR‐179

### Upper limb rehabilitation using virtual reality vs. a real‐setting training in people with Parkinson's disease

#### A. Gardoni^1^; E. Sarasso ^2^; L. Zenere^3^; A. Grassi^3^; R. Balestrino^4^; S. Basaia^3^; E. Canu^3^; E. Sibilla^3^; V. Castelnovo^3^; F. Freri^3^; C. Tripodi^3^; D. Emedoli^5^; M. Malcangi^4^; M. Volontè^6^; D. Corbetta^5^; M. Filippi^7^; F. Agosta^8^

##### ^1^Neuroimaging Research Unit, Division of Neuroscience, IRCCS San Raffaele Scientific Institute, and Vita‐Salute San Raffaele University, Milan, Italy; ^2^Neuroimaging Research Unit, Division of Neuroscience, IRCCS San Raffaele Scientific Institute, Vita‐Salute San Raffaele University, Milan, Italy; and DINOGMI, University of Genoa, Genoa, Italy; ^3^Neuroimaging Research Unit, Division of Neuroscience, IRCCS San Raffaele Scientific Institute, Milan, Italy; ^4^Neurology Unit, and Neurorehabilitation Unit, IRCCS San Raffaele Scientific Institute, Milan, Italy; ^5^Department of Rehabilitation and Functional Recovery, IRCCS San Raffaele Scientific Institute, Milan, Italy; ^6^Neurology Unit, IRCCS San Raffaele Scientific Institute, Milan, Italy; ^7^Neurology Unit, Neurorehabilitation Unit, Neurophysiology Service, and Neuroimaging Research Unit, Division of Neuroscience, IRCCS San Raffaele Scientific Institute, and Vita‐Salute San Raffaele University, Milan, Italy; ^8^Neuroimaging Research Unit, Division of Neuroscience, and Neurology Unit, IRCCS San Raffaele Scientific Institute, and Vita‐Salute San Raffaele University, Milan, Italy

**Background and aims:** Bradykinesia affects upper‐limb functions in Parkinson's disease (PD). This study assessed the efficacy of physiotherapy on upper‐limb motor function and brain activity in patients with PD (pwPD) comparing virtual‐reality and real‐setting training.

**Methods:** Forty pwPD and 30 age‐/sex‐matched healthy controls (HC) were included. We obtained kinematic data on touchscreen gestures and handwriting through customized tests on tablet/smartphone. Subjects performed a fMRI hand‐tapping task. PwPD were randomized into two groups performing 8‐week rehabilitation program stimulating speed/amplitude of handwriting and tap/swipe/slide movements in a real‐setting (RS‐training‐group) or using technological devices/virtual‐reality (VR‐training‐group).

**Results:** Relative to HC, pwPD showed reduced manual dexterity and movement speed/amplitude, and reduced fMRI activity of motor‐related brain areas. After rehabilitation, VR‐training‐group showed greater improvement in manual dexterity. Both patient groups showed improved speed/amplitude during handwriting; however, VR‐training‐group showed greater improvement in handwriting on tablet. Both patient groups improved in tap/swipe tasks on smartphone, particularly RS‐training‐group. Both patient groups showed reduced sequence effect on amplitude during finger‐tapping task, with RS‐training‐group showing greater improvement. After training, RS‐training‐group showed increased activity of sensorimotor areas and reduced activity of extra‐motor areas; VR‐training‐group showed increased activity of thalamus and parietal areas and reduced activity of caudate and temporal areas.

**Conclusion:** Physiotherapy can promote improvements in upper‐limb function and brain functional reorganization in pwPD. Real‐setting or virtual‐reality trainings have some specific effects that might help tailoring the therapeutic approach, but one cannot be considered superior. Imaging results could be interpreted as a pattern of recovery in RS‐training‐group and of mixed compensatory/recovery mechanisms in VR‐training‐group.

**Disclosure:** Funding. Italian Ministry of Health (GR‐2018‐12366005). Disclosures. AG, LZ, AGr, RB, DE, ES, VC, FF, CT, MM, MAV nothing to disclose. ES, SB, EC, DC grants form the Italian Ministry of Health. MF received compensation for consulting services or speaking activities from Alexion, Almirall, Bayer, Biogen, Celgene, Chiesi Italia SpA, Eli Lilly, Genzyme, Janssen, Merck‐Serono, Neopharmed Gentili, Novartis, Novo Nordisk, Roche, Sanofi Takeda, and TEVA; Advisory Boards for Alexion, Biogen, Bristol‐Myers Squibb, Merck, Novartis, Roche, Sanofi, Sanofi‐Aventis, Sanofi‐Genzyme, Takeda; scientific direction of educational events for Biogen, Merck, Roche, Celgene, Bristol‐Myers Squibb, Lilly, Novartis, Sanofi‐Genzyme; he receives research support from Biogen Idec, Merck‐Serono, Novartis, Roche, the Italian Ministry of Health, the Italian Ministry of University and Research, and FISM. FA received speaker honoraria from Biogen Idec, Roche, Eli Lilly and GE Healthcare; and grants from Italian Ministry of Health, Italian Ministry of University and Research, AriSLA, European Research Council, EU Joint Programme—Neurodegenerative Disease Research, and Foundation Research on Alzheimer Disease (France).

## EPR‐180

### Efficacy on disphagia of swallowing therapy plus neuromuscular electrostimulation vs. tst plus sham‐nmes in MS

#### c. Solaro ^2^; R. Beccari^1^; D. Cattaneo^5^; i. Colombini^2^; A. Cuccaro^1^; S. De Santi^2^; R. Di Giovanni^2^; G. Fusari^5^; C. Giudice^4^; K. Inglese^2^; D. Martinelli^4^; E. Monti^4^; M. Monti Bragandin^3^; F. Masserano Zoli^3^; G. Musto^1^; A. Servetto^4^; M. Simonelli^1^; A. Toscano^3^; A. Tufaro^3^; C. Vitali^5^; G. Presicce^1^; G. Bricchetto^3^; M. Rovaris^5^; C. Tassorelli^4^; M. Grasso^1^

##### ^1^I.R.R.C.S. “FONDAZIONE S. LUCA” Via Ardeatina, 306 ‐ Rome, Italy; ^2^C.R.R.F. “Mons. L. Novarese Moncrivello”; ^3^Italian MS Foundation; ^4^IRCCS C. Mondino Foundation and University of Pavia; ^5^IRCCS Fondazione Don Carlo Gnocchi, Via Capecelatro, 66, 20148 Milan, Italy

**Background and aims:** Dysphagia is a disabling symptom in MS. The effectiveness of neuromuscular electrical stimulation (NMES) for dysphagia has limited research in pwMS. The aim of the study was to determine whether NMES benefit to a standard rehabilitative therapy (SRT) in dysphagic pwMS.

**Methods:** This multicenter, double‐blind, randomized clinical trial compared SRT‐NMES versus SRT with sham NMES (SRT‐S). Patients underwent 16 therapy sessions and assessments at baseline (T0), after 16 sessions (T2) and 12 weeks post‐treatment (T3). Main outcomes was ASHA scale, Visual VAS, DYMUS, and DOSS scores on FEES.

**Results:** 101 patients (mean age: 56.2 ± 10.9 years; 55% female). Both interventions led to significant improvements in ASHA, DYMUS, VAS and DOSS. In ASHA scores in the SRT‐NMES improved from 4.3 ± 1.3 at T0 to 5.2 ± 0.8 at T2 (*p* < 0.001), in the SRT‐S group from 4.5 ± 1.0 at T0 to 5.0 ± 0.8 at T2 (*p* < 0.001). DYMUS in the SRT‐NMES group from 4.3 ± 2.2 at T0 to 2.8 ± 2.2 at T2 (*p* < 0.001) and in the SRT‐S group from 5.1 ± 2.8 at T0 to 3.9 ± 2.4 at T2 (*p* < 0.001). DOSS scores improved in SRT‐NMES group from 4.4 ± 1.1 at T0 to 5.3 ± 0.8 at T2 (*p* < 0.001) and the SRT‐S group from 4.6 ± 1.1 at T0 to 5.1 ± 0.9 at T2 (*p* = 0.001).

**Conclusion:** Both SRT‐NMES and SRT‐S therapies significantly improved swallowing function in pwMS. The inter‐group analysis showed a trend in favor of SRT‐NMES, Future research should explore the long‐term benefits of these therapies to enhance rehabilitation strategies.

**Disclosure:** Nothing to disclose.

## EPR‐181

### Impact of experimentally induced fatigability on motor and cognitive functions in MS: a cross‐sectional study

#### C. Solaro

##### Neurology Unit Galliera Hospital Genova, Genoa, Italy

**Background and aims:** Fatigability is a major symptom in People with Multiple Sclerosis (PwMS), affecting walking, balance, and cognitive function, and reducing quality of life. This study aimed to assess the acute effects of induced fatigability on gait, balance, and cognitive functions in PwMS using objective, instrumented assessments before (Pre), immediately after (Post), and after 30‐minute (Rest) an overground Fatiguing Walking Test (FWT).

**Methods:** 96 PwMS (age: 54.1 ± 10.2 EDSS: 3.1 ± 1.2 points, 52% women) and 22 HC were assessed at three Italian MS centers. Participants completed a 30‐minute or until exhaustion (Rate of Perceived Exertion; RPE; > 17 points) FWT, followed by balance tests (firm/foam surface, eyes open/closed) with three Inertial Measurement Units and cognitive assessment using the Brief International Cognitive Assessment for Multiple Sclerosis (BICAMS) at Pre, Post, and Rest.

**Results:** All HS completed the FWT with a RPE of 10.5 ± 2.6 points, while 49% of PwMS completed the FWT (RPE: 14.5 ± 3.7 points), and 51% walked for 15.6 ± 6.3 minutes (RPE: 19.1 ± 1.1 points, p Time * Group < 0.001). (Figure 1) Static balance tests showed significant Time* Group interactions for trunk Medio‐Lateral (ML) sway amplitude (*p* = 0.011) and sample entropy (*p* = 0.043 ). (Figure 2) Dynamic balance showed increased ML instability in PwMS versus a decrease in HS ( *p* = 0.007). No changes were observed in BICAMS (*p* > 0.05

**Conclusion:** Data show an acute effect of motor fatigability on balance but on cognition. Next step will be to apply an ad‐hoc rehabilitation protocol in order to interferee with faticability

**Disclosure:** This work was supported by the Italian Multiple Sclerosis Foundation [FISM Grant 2022/R‐Multi/005] Nothing to disclose.

## EPR‐182

### Rehabilitation after stroke: The role of educational programs for family members

#### S. Khudoyorov; D. Rasulova; M. Rasulova; I. Yusupova

##### Tashkent Medical Academy, Tashkent, Uzbekistan

**Background and aims:** Stroke is considered as one of the major causes of permanent disability across the globe contributing to increased functional impairment and reduced quality of life. Rehabilitation is largely dependent on family support, but the role of trained caregivers on recovery outcomes needs to be studied further.

**Methods:** A randomized controlled trial was conducted with 60 post‐stroke patients, with an intervention group (*n* = 30) whose family members were taken through an education program, while the control group (*n* = 30) received the standard care Functional recovery was assessed using the Fugl‐Meyer Assessment (FMA) at 1, 3, and 6 months. Preliminary evaluations were done using Modified Rankin Scale (MRS), the National Institutes of Health Stroke Scale (NIHSS) and the Rivermead Mobility Index (RMI). At six months follow up, quality of life was measured using Stroke‐Specific Quality of Life Scale (SS‐QOL).

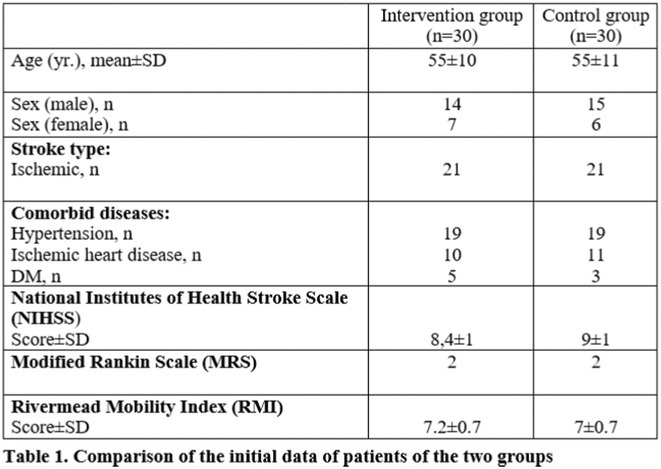

**Results:** The intervention group proved to have improved functional recovery, with FMA scores increasing from 59 ± 11 at 1 month to 92.7 ± 12 at 6 months, unlike the control group (51 ± 11 to 67 ± 14) with significant improvements (*p* < 0.01). Quality of life was notably good in the intervention group which SS‐QOL scored them at 162.2 ± 11 compared to the control group with 106.9 ± 17 with (*p* < 0.01). The program prepared family members to enhance their support in rehabilitation activities leading to better recovery trajectories.

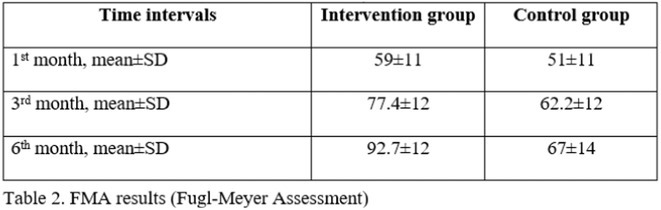

**Conclusion:** Structured educational programs for relatives significantly improve the functional recovery and quality of life of patients after stroke. Further studies with larger samples are recommended to confirm the findings and optimize educational programs.

**Disclosure:** Nothing to disclose.

## EPR‐183

### Functional neural correlates of improvements after aerobic training in people with multiple sclerosis

#### F. Romanò^1^; P. Valsasina^1^; M. Albergoni^1^; T. Morozumi ^2^; N. Tedone^2^; P. Preziosa^3^; M. Rocca^4^; M. Filippi^3^

##### ^1^Neuroimaging Research Unit, Division of Neuroscience, IRCCS San Raffaele Scientific Institute, Milan, Italy; ^2^Neuroimaging Research Unit, Division of Neuroscience, IRCCS San Raffaele Scientific Institute, and Vita‐Salute San Raffaele University, Milan, Italy; ^3^Neuroimaging Research Unit, Division of Neuroscience, and Neurology Unit, IRCCS San Raffaele Scientific Institute, and Vita‐Salute San Raffaele University, Milan, Italy; ^4^Neurology Unit, Neurorehabilitation Unit, Neurophysiology Service, and Neuroimaging Research Unit, Division of Neuroscience, IRCCS San Raffaele Scientific Institute, and Vita‐Salute San Raffaele University, Milan, Italy

**Background and aims:** Aerobic training (AT) has shown promising results for the improvement of motor and cognitive functions in people with multiple sclerosis (pwMS). The aim of this study was to investigate the neural correlates of such changes using functional magnetic resonance imaging (fMRI).

**Methods:** Fifty‐seven pwMS and 39 healthy controls (HC) were randomized to receive either AT or a control intervention (CT) for 8 weeks. Treatment was performed 3 times/week in 30‐minute sessions. AT consisted of treadmill walking at progressively increasing intensities, while CT included mobilization, stretching, and balance exercises. The assessments, performed before and after treatment, evaluated cardiorespiratory fitness (V02peak), gait, mobility, cognitive functions (Brief Repeatable Battery of Neuropsychological tests), and brain activations during the Stroop test, which evaluates the ability to inhibit cognitive interference.

**Results:** After training, both MS groups improved in mobility (*p* < = 0.24) and verbal learning (*p* < = 0.01). MS‐AT improved in V02peak, gait endurance, and cognitive interference inhibition (*p* < = 0.02), while MS‐CT improved in verbal memory, visuospatial memory, and semantic fluency (*p* < = 0.03). All MS and HC groups improved in information processing speed (*p* < = 0.45). Stroop fMRI activity increased in the pre/postcentral gyri, thalamus, and cerebellum in MS‐AT, while it decreased in the thalamus, insula, and occipital cortex in MS‐CT (*p* < 0.001, uncorrected). No fMRI changes were found in HC. There were significant correlations between functional improvements and changes in fMRI activity in both MS groups.

**Conclusion:** AT is a valid strategy for the management of motor and cognitive dysfunctions in pwMS. Functional neuroplasticity represents one of the possible mechanisms underlying improvements after treatment.

**Disclosure:** Funding. Partially supported by Italian Ministry of Health (GR‐2019‐12369599). Disclosures. FR, PV, MA, TM, NT have nothing to disclose. PP received speaker honoraria from Roche, Biogen, Novartis, Merck, Bristol Myers Squibb, Genzyme, Horizon and Sanofi. He received research support from Italian Ministry of Health and Fondazione Italiana Sclerosi Multipla (FISM). MAR received consulting fees from Biogen, Bristol Myers Squibb, Eli Lilly, Janssen, Roche, and speaker honoraria from AstraZaneca, Biogen, Bristol Myers Squibb, Bromatech, Celgene, Genzyme, Horizon Therapeutics Italy, Merck Serono SpA, Novartis, Roche, Sanofi and Teva, she receives research support from the MS Society of Canada, the Italian Ministry of Health, the Italian Ministry of University and Research, and FISM. MF received compensation for consulting services or speaking activities from Alexion, Almirall, Bayer, Biogen, Celgene, Chiesi Italia SpA, Eli Lilly, Genzyme, Janssen, Merck‐Serono, Neopharmed Gentili, Novartis, Novo Nordisk, Roche, Sanofi Takeda, and TEVA; Advisory Boards for Alexion, Biogen, Bristol‐Myers Squibb, Merck, Novartis, Roche, Sanofi, Sanofi‐Aventis, Sanofi‐Genzyme, Takeda; scientific direction of educational events for Biogen, Merck, Roche, Celgene, Bristol‐Myers Squibb, Lilly, Novartis, Sanofi‐Genzyme; he receives research support from Biogen Idec, Merck‐Serono, Novartis, Roche, the Italian Ministry of Health, the Italian Ministry of University and Research, and FISM.

## EPR‐184

### Challenges in neurorehabilitation in a war zone

#### T. Voloshyn

##### KIRC, Truskavets, Ukraine

**Background and aims:** In war zones, neurorehabilitation faces unique challenges that impact the delivery and effectiveness of care for individuals with neurological diseases. Scarcity of medical supplies, equipment, and trained personnel hampers the ability to provide rehabilitation. Ongoing conflict poses risks to both patients and healthcare providers, making it difficult to access facilities&maintain consistent care. The trauma associated with war exacerbates psychological conditions, complicating rehabilitation efforts. Many individuals are displaced due to conflict, affecting their access to rehabilitation services and continuity of care.

**Methods:** Mixed‐methods approach, combining quantitative data from rehabilitation centers in conflict‐affected areas with qualitative interviews from healthcare providers/patients. Surveys assessed resource availability, treatment outcomes, and patient satisfaction, while interviews explored personal challenges faced during rehabilitation.

**Results:** Critical resource shortages, with 70% of centers reporting inadequate access to essential medical supplies and rehabilitation equipment. Security issues were cited by 85% of healthcare providers as a major barrier, resulting in interrupted services and limited patient access. Psychosocial challenges were prevalent, with 80% of patients exhibiting increased anxiety, depression, which negatively impacted their rehabilitation progress. Coordination issues among healthcare providers led to fragmented care, as reported by 75% of surveyed professionals.

**Conclusion:** Addressing these challenges requires innovative strategies, collaboration with local communities, and adaptable rehabilitation models that consider the complexities of providing care in a war‐torn environment. There's urgent need for targeted interventions to address these challenges. Strategies that enhance resource allocation, ensure safety, integrate mental health support, foster cultural competence are essential for improving rehabilitation outcomes in the war zone environments.

**Disclosure:** Nothing to disclose.

## EPR‐185

### Predictors of response to physical exercise in patients with multiple sclerosis: A structural and functional MRI study

#### T. Morozumi ^1^; P. Preziosa^2^; A. Meani^3^; E. Pagani^3^; P. Valsasina^3^; C. Arezzo^3^; M. Filippi^4^; M. Rocca^2^

##### ^1^Neuroimaging Research Unit, Division of Neuroscience, IRCCS San Raffaele Scientific Institute, and Vita‐Salute San Raffaele University, Milan, Italy; ^2^Neuroimaging Research Unit, Division of Neuroscience, and Neurology Unit, IRCCS San Raffaele Scientific Institute, and Vita‐Salute San Raffaele University, Milan, Italy; ^3^Neuroimaging Research Unit, Division of Neuroscience, IRCCS San Raffaele Scientific Institute, Milan, Italy; ^4^Neurology Unit, Neurorehabilitation Unit, Neurophysiology Service, and Neuroimaging Research Unit, Division of Neuroscience, IRCCS San Raffaele Scientific Institute, and Vita‐Salute San Raffaele University, Milan, Italy

**Background and aims:** Aerobic exercise is a promising rehabilitation strategy for multiple sclerosis (MS). However, a certain heterogeneity in individual response may be observed. This study aimed to assess how baseline demographic, clinical, and MRI features of MS patients influence their response to aerobic and non‐aerobic motor training.

**Methods:** Eighty‐eight MS patients and 70 healthy controls underwent structural and functional MRI acquisition to evaluate lesional, volumetric, cortical thickness, diffusivity, and resting‐state functional connectivity measures. Clinical evaluation included expanded disability status scale, modified fatigue impact scale, timed 25‐foot walk test, peak oxygen consumption and 6‐minute walking test (6MWT). Patients were divided into two groups following aerobic or non‐aerobic exercise programs and classified as “responders” or “non‐responders” based on a 6MWT post‐treatment improvement of at least 21.6 meters. Predictive factors for improvement were assessed among demographic, clinical, and MRI measures using random forest analyses.

**Results:** Independent predictors of treatment response were corticospinal tract (CST) fractional anisotropy (FA) and middle cerebellar peduncle (MCP) FA in the aerobic group (out‐of‐bag [OOB]‐area under the curve [AUC] = 0.682), superior cerebellar peduncle (SCP) mean diffusivity (MD) in the non‐aerobic group (OOB‐AUC = 0.713), and SCP MD, MCP MD, CST MD, CST FA, and MCP FA in all MS patients (OOB‐AUC = 0.734).

**Conclusion:** Response to physical exercise in MS patients is associated with specific markers of microstructural damage in key white matter tracts related to motor function for both the treatment strategies proposed. These findings highlight the potential role of neuroimaging in identifying MS patients who most likely benefit from motor rehabilitation.

**Disclosure:** Funding. Grants from Italian Ministry of Health (GR‐2019‐12369599) and MS Society of Canada (EGID3185). Disclosures. TM, AM, EP, PV, CA nothing to disclose. PP received speaker honoraria from Roche, Biogen, Novartis, Merck, Bristol Myers Squibb, Genzyme, Horizon and Sanofi. He received research support from Italian Ministry of Health and Fondazione Italiana Sclerosi Multipla (FISM). MAR received consulting fees from Biogen, Bristol Myers Squibb, Eli Lilly, Janssen, Roche, and speaker honoraria from AstraZaneca, Biogen, Bristol Myers Squibb, Bromatech, Celgene, Genzyme, Horizon Therapeutics Italy, Merck Serono SpA, Novartis, Roche, Sanofi and Teva, she receives research support from the MS Society of Canada, the Italian Ministry of Health, the Italian Ministry of University and Research, and FISM. MF received compensation for consulting services or speaking activities from Alexion, Almirall, Bayer, Biogen, Celgene, Chiesi Italia SpA, Eli Lilly, Genzyme, Janssen, Merck‐Serono, Neopharmed Gentili, Novartis, Novo Nordisk, Roche, Sanofi Takeda, and TEVA; Advisory Boards for Alexion, Biogen, Bristol‐Myers Squibb, Merck, Novartis, Roche, Sanofi, Sanofi‐Aventis, Sanofi‐Genzyme, Takeda; scientific direction of educational events for Biogen, Merck, Roche, Celgene, Bristol‐Myers Squibb, Lilly, Novartis, Sanofi‐Genzyme; he receives research support from Biogen Idec, Merck‐Serono, Novartis, Roche, the Italian Ministry of Health, the Italian Ministry of University and Research, and FISM.

## EPR‐186

### Bimanual motor skill learning in acute stroke patients: robotic assessment and neural substrates

#### Y. Vandermeeren ^1^; N. Delinte^2^; L. Dricot^3^; B. Herman^4^; T. Lejeune^5^; B. Bihin^6^; N. Mulquin^7^; B. de Coene^7^; C. Hoffelt^1^; L. Lefebvre^1^; C. van Ravestyn^1^

##### ^1^Stroke Unit / Motor Learning Lab, Department Neurology, CHU UCL Namur (Godinne) / UCLouvain, Yvoir, Belgium.; ^2^Louvain Bionics, UCLouvain, Louvain‐la‐Neuve, Belgium; ^3^NEUR division, Institute of NeuroScience (IoNS), UCLouvain, Brussels, Belgium; ^4^UCLouvain, Institute of Mechanics, Materials and Civil Engineering, Louvain‐la‐Neuve, Belgium.; ^5^Physical Medicine and Rehabilitation Department, Saint‐Luc University Clinics / UCLouvain, Brussels, Belgium; ^6^Scientific Support Unit (USS), CHU UCL Namur (Godinne) / UCLouvain, Yvoir, Belgium.; ^7^Radiology Department, CHU UCL Namur (Godinne) / UCLouvain, Yvoir, Belgium

**Background and aims:** Activities of daily life are mostly bimanual, learned, and may be impaired by stroke. While rehabilitation of bimanual coordination and skills is crucial, it is currently unknow whether bimanual motor skill learning (bim‐MSkL) is enhanced or impaired during (sub)acute stroke. We aimed to quantify bim‐MSkL in acute patients and unveil the neural structures related to impaired and successful bim‐MSkL.

**Methods:** 74 (sub)acute stroke patients and 62 age‐matched healthy individuals (HI) trained during three consecutive days with a bim‐MSkL cooperation task on the REA2plan® robot (AXINESIS, Belgium). Using the REA2plan's handles, they had to complete as many laps as possible on a complex circuit over 1‐minute blocks. One hand controlled the common cursor's lateral displacements, the other its sagittal displacements; which hand controlled which axis was randomized. Bim‐MSkL was quantified with a bimanual speed/accuracy trade‐off (biSAT) and coordination with a bimanual coordination factor of hands speeds (BCF). The patients underwent a comprehensive evaluation and a multimodal MRI.

**Results:** Overall, the patients improved bim‐MSkL and bimanual coordination over the three days, but less than the HI (log(biSAT): ‐0.43, *p* < 0.001; BCF: ‐0.08, *p* < 0.001), likely because more patients (42%) were poor learners (biSAT improvement < 25%) than HI (16%). Clusters of injured voxels associated with reduced motor function at baseline and reduced ability to improve coordination were located along the corticospinal tract and in the corpus callosum.

**Figure:**
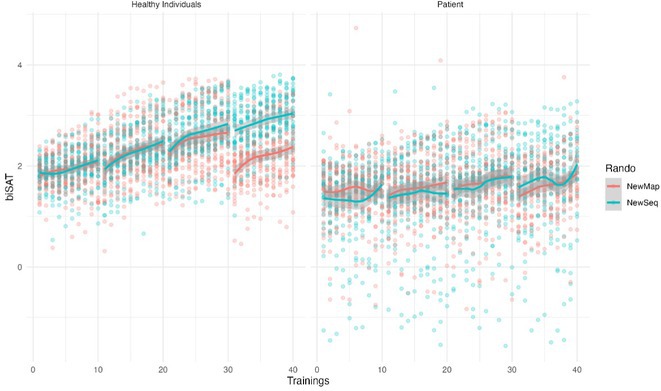
**FIGURE 1** log(biSAT) in HI and patients (10 training/day). For generalization on Day 3, either the sequence (CIRCUIT NewSeq) or the hands mapping (NewMap) changed.

**Figure:**
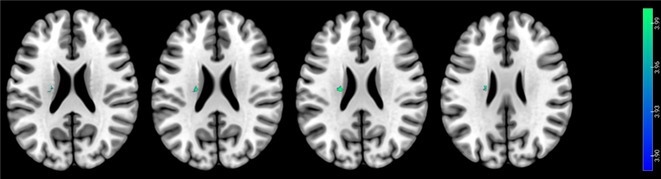
**FIGURE 2** Voxel‐based Lesion Symptom Mapping (*n* = 50). Acute stroke involving the corticospinal tract impaired the improvement of bimanual coordination over 3 days.

**Conclusion:** Shortly after the stroke, the patients showed a reduction in bim‐MSkL, which correlated with damage to the corticospinal tract and corpus callosum.

**Disclosure:** Nothing to disclose.

## Neuroimmunology 2

## EPR‐187

### HLA Class I and II alleles in myasthenic patients of Romanian descent: considerations regarding genotype and phenotype

#### C. Croitoru ^1^; M. Pavel‐Tanasa^2^; D. Hodorog^3^; D. Cuciureanu^3^; P. Cianga^2^

##### ^1^Neurology Clinic I, “Prof. Dr . Nicolae Oblu” Emergency Clinical Hospital, Iași, Romania; ^2^Department of Immunology, “Grigore T. Popa” University of Medicine and Pharmacy, Iași, Romania; ^3^Medical Department III‐Neurology, “Grigore T. Popa” University of Medicine and Pharmacy, Iași, Romania

**Background and aims:** In Eastern European populations data regarding positive and negative associations between certain Human Leukocyte Antigen (HLA) alleles and myasthenia gravis (MG) subtypes are scarce. The main aim of this study was to analyze the associations of HLA Class I and II alleles with MG in patients of Romanian descent.

**Methods:** We consecutively enrolled adult Romanian unrelated myasthenic patients. After genotyping them by next‐generation sequencing for six primary loci (HLA‐A, ‐B, ‐C, ‐DRB1, ‐DQB1 and ‐DPB1) we compared their allelic frequencies with the controls’. The latter were represented by three separate groups of random normal subjects, two descent‐matched and one of Italian descent, collected from the Allele Frequency Net Database.

**Figure:**
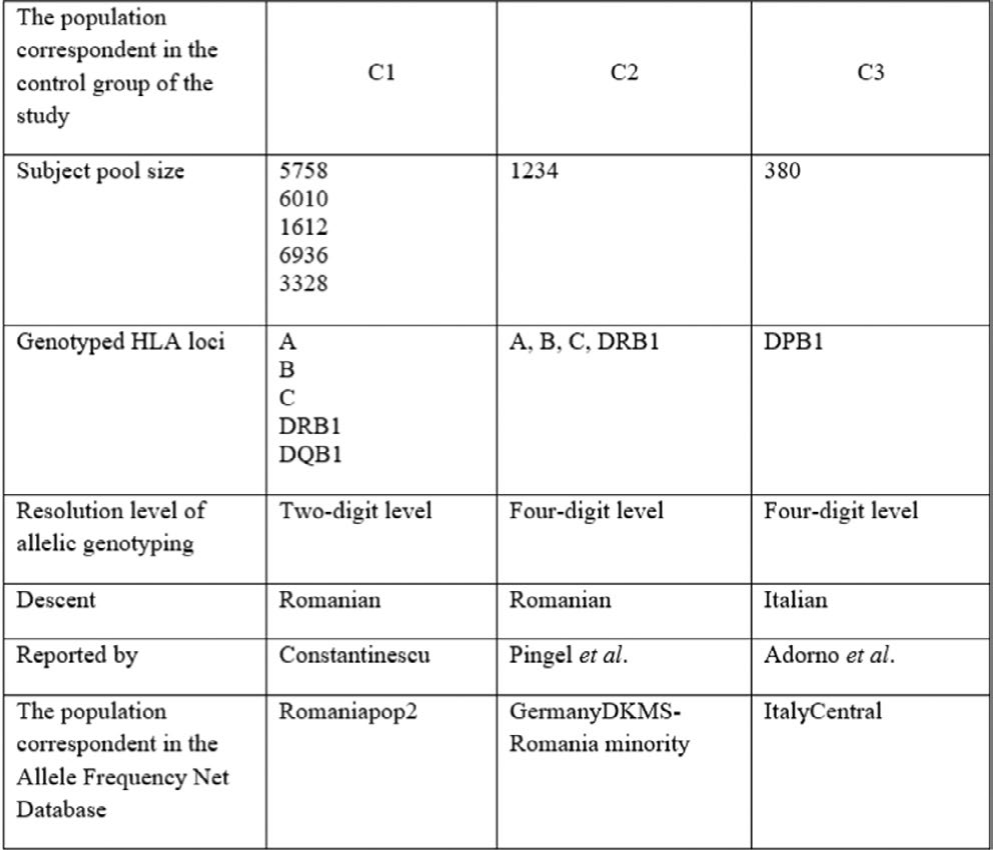
**TABLE 1** The control lot.

**Results:** We included 40 heterogenous patients (women: 80%; early‐onset MG: 57.50%; juvenile MG: 7.50%; thymomatous MG: 22.50%; anti‐acetylcholine receptor autoantibodies positive MG: 75%) and identified a total of 109 alleles. We obtained four previously acknowledged allelic associations in Western‐European and North‐American Caucasian subjects: negative for DRB1*13 and positive for HLA‐B*08, DRB1*14:54 and DRB1*16:01. Surprisingly, we discovered six potential novel significant positive allelic associations with MG (HLA‐A*02:36, B*47, B*73, B*44:27, B*57:02 and HLA‐ DPB1*51:01). Further, we analyzed the HLA allelic profile of specific MG subtypes and discussed potential immunological mechanisms involved.

**Conclusion:** Our work pioneers allelic association studies in Romanian myasthenic patients. The results suggest that the geographical and ethnical disparities of the MG‐associated HLA alleles are perhaps overrated. Consequently, one can deduce that at least some of the immunopathogenic mechanisms of MG might also be common in patients of different descent.

**Disclosure:** This research was co‐funded by the Romanian Operational Program for Competitiveness 2014–2020, Axis 1, under POC/448/1/1 Research Infrastructure Projects for Public R&D Institu‐tions/Universities “Multidisciplinary Medical Research‐Development Platform in the NE re‐gion ”CENEMED”, mySMIS code: 127606.

## EPR‐188

### Immune checkpoint inhibitor‐related myositis‐myasthenia: A retrospective cohort study in Spain

#### E. Martinez‐Hernandez ^1^; J. Cabrera‐Maqueda^1^; E. Fonseca^1^; C. Marco^2^; J. Chico^3^; J. Gomez‐Fernandez^4^; A. Llaurado^5^; J. Cabello^6^; L. del Pino^7^; C. Izquierdo^8^; E. Cortés^9^; J. Gállego^10^; M. Ros^11^; R. Caldú^12^; A. Pelayo^13^; C. Sanchez‐Vizcaino^14^; H. Hernández^15^; J. Álvarez^16^; M. Simó^2^; J. Dalmau^1^; J. Bruna^2^; R. Velasco^2^

##### ^1^Department of Neurology. Hospital Clinic Barcelona FRCB‐IDIBAPS. Barcelona. Spain; ^2^Department of Neurology. Hospital de Bellvitge, Catalan Institute of Oncology. L'Hospitalet de Llobregat. Spain; ^3^Department of Neurology. Hospital Ramon y Cajal. Madrid. Spain; ^4^Department of Neurology. Hospital Virgen del Rocío. Sevilla. Spain; ^5^Department of Neurology. Hospital Vall d'Hebron. Barcelona. Spain; ^6^Department of Neurology. Hospital La Fe. Valencia. Spain; ^7^Department of Neurology. Hospital Gregorio Marañón. Madrid. Spain; ^8^Department of Neurology. Hospital German Tries i Pujol. Badalona. Spain; ^9^Deaprtment of Neurology. Hospital de Sant Pau. Barcelona. Spain, ^10^Department of Neurology. Clínica Universitaria de Navarra. Pamplona. Spain, ^11^Deaprtment of Neurology. Hospital Parc Taulí. Sabadell. Spain, ^12^Department of Neurology. Hospital Clínico Lozano Blesa. Zaragoza. Spain, ^13^Department of Neurology. Hospital Marqués de Valdecilla. Santander. Spain, ^14^Department of Neurology. Hospital Santa Lucía. Cartagena. Spain, ^15^Department of Neurology. Hospital Nuestra Señora de Candelaria. Tenerife. Spain, ^16^Department of Internal Medicine. Hospital La Paz. Madrid. Spain

**Background and aims:** The combination myositis‐myasthenia with or without myocarditis, is a rare but life‐threatening complication of immune checkpoint inhibitors (ICI). Diagnostic and treatment approaches vary across hospitals. We evaluated the clinical spectrum, treatments, and outcomes of patients with this ICI‐related complication.

**Methods:** This retrospective observational multicenter study in Spain collected clinical information on patients diagnosed with ICI‐related myositis‐myasthenia with/without myocarditis over the past 7 years using a structured questionnaire. Syndromes were classified as definite, probable, or possible based on the 2021 consensus disease definitions.

**Results:** We included 133 patients from 33 hospitals, with a median age of 72 years [IQR 34‐89], 67% male. Symptoms began 30 days [IQR 18‐68] after ICI initiation, including limb weakness, ptosis, diplopia, dysphagia, dysphonia, dyspnea, myalgia and axial weakness. Mean time from onset to hospital admission was 11 days [IQR 6‐27]. Initial diagnoses were myositis in 88% (isolated 22%, combined with myasthenia 15%); myocarditis in 54% (with myositis 27%), and myasthenia in 9% (with myositis and myocarditis 24%). According to consensus definitions, 74% of myositis cases were definite, 61% of myasthenia cases probable, and 49% of myocarditis cases possible. During the acute phase, 74% had mRS > 2, 26% required intensive care, 96% received steroids, and 60% received immunoglobulins. At 30 days, 16% had died, 9% worsened, 3% stabilized, and 72% improved. At last follow‐up (175 days [IQR 47‐579]), ICI‐related mortality was 23%. Predictors of poor outcomes will be presented.

**Figure:**
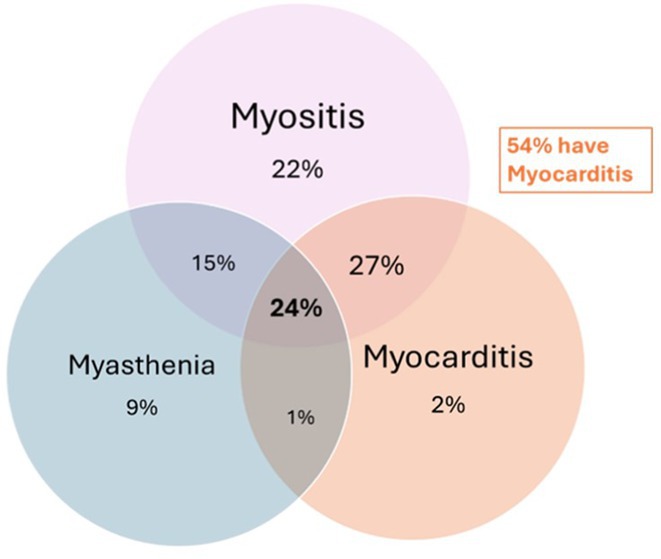
**FIGURE 1** Frequncy and combinations of myositis, myasthenia and myocarditis

**Conclusion:** ICI‐related myositis‐myasthenia develops early after ICI initiation, progresses with severe symptoms, often includes myocarditis, and has a high first‐month mortality rate.

**Disclosure:** This study is supported, in part, by a grant from the Neuroepidemiology Study Group of the Spanish Society of Neurology.

## EPR‐189

### The rs10191329 risk allele is associated with retinal layer thinning in multiple sclerosis

#### G. Bsteh ^1^; A. Schmidt^3^; N. Krajnc^1^; F. Föttinger^1^; T. König^2^; M. Krenn^1^; M. Ponleitner^1^; H. Hegen^4^; B. Pemp^5^; T. Berger^1^

##### ^1^Department of Neurology, Medical University of Vienna, Austria; ^2^Comprehensive Center for Clinical Neurosciences and Mental Health, Medical University of Vienna, Austria; ^3^Department of Neurology, Medical University of Bonn, Bonn, Germany; ^4^Department of Neurology, Medical University of Innsbruck, Innsbruck, Austria; ^5^Department of Ophthalmology, Medical University of Vienna, Vienna, Austria

**Background and aims:** The minor allele of the genetic variant rs10191329 in the DYSF–ZNF638 locus, associated with CNS resilience rather than immune‐mediated pathology, has been linked to faster disability progression in MS. Retinal layer thinning measured by OCT is a biomarker of neuroaxonal damage. We aimed to investigate whether rs10191329 is associated with retinal layer thinning in relapsing MS.

**Methods:** We included relapsing MS patients with ≥2 OCT scans from a prospective observational study, excluding eyes affected by optic neuritis. DNA was genotyped using the Illumina Infinium Global Screening Array‐24 and imputed to the Haplotype Reference Consortium panel using Minimac4 (V1.0.2). A multivariate linear regression model evaluated mean annualized rates of retinal layer thinning (%/year) in the peripapillary retinal nerve fiber layer (aLpRNFL) and ganglion cell‐inner plexiform layer (aLGCIPL) using rs10191329*A allele count as the independent variable, adjusted for age, sex, and ancestry components.

**Results:** We included 208 patients (mean age 30.5 years [SD 7.9], 74.1% female, median EDSS 2.0, median follow‐up 25 months, median OCT scans 3). The rs10191329 risk allele was associated with faster retinal thinning (estimate 0.119 [SE 0.056], *p* < 0.001). Each rs10191329*A allele increased aLpRNFL thinning by 0.10%/year (95% CI 0.05–0.19) and aLGCIPL thinning by 0.11%/year (95% CI 0.07–0.19), contributing 26.4% and 27.2% of the mean atrophy rates, respectively.

**Conclusion:** The minor rs10191329 allele may predispose carriers to MS‐related neuroaxonal damage. Genotypic stratification in clinical trials may be warranted.

**Disclosure:** Gabriel Bsteh: has participated in meetings sponsored by, received speaker honoraria or travel funding from Biogen, Celgene/BMS, Lilly, Merck, Novartis, Roche, Sanofi‐Genzyme and Teva, and received honoraria for consulting Biogen, Celgene/BMS, Novartis, Roche, Sanofi‐Genzyme and Teva. He has received unrestricted research grants from Celgene/BMS and Novartis.

## EPR‐190

### Efgartigimod combined with glucocorticoid in the treatment of severe generalized myasthenia gravis

#### S. Gu; Y. Li; H. Dong; H. Yang; M. Ma; M. Li; C. Yan; P. Jia; Y. Wang; G. Qi

##### Center of Treatment of Myasthenia Gravis, People's Hospital of Shijiazhuang, Shijiazhuang, China

**Background and aims:** High‐dose glucocorticoid pulse (IVMP) is effective in treating generalized myasthenia gravis (gMG), but early treatment may cause transient aggravation, with some patients experiencing MG crisis. This study investigates the safety and efficacy of efgartigimod combined with different doses of IVMP in gMG patients.

**Methods:** Myasthenia gravis patients treated at Shijiazhuang People's Hospital from December 2023 to May 2024 were retrospectively analyzed. Patients received efgartigimod (D1, D8) combined with IVMP or IVMP alone. Efficacy was assessed using the Quantitative Myasthenia Gravis Score (QMG) at baseline, 2 and 12 weeks.

**Results:** A total of 57 patients were included: 20 in the 1000 mg IVMP group (A), and 7, 10, and 20 in the 1000 mg (B), 500 mg (C), and 250 mg (D) IVMP combined with efgartigimod groups, respectively. QMG scores for groups A, C, and D gradually decreased over 12 weeks. Group B showed an increase in QMG score at week 12 compared to week 2 but remained below baseline (Figure 1). There was a significant difference between group A and group B at week 2 in QMG score reduction (Figure 2). Group A had a significantly higher incidence of transient exacerbations than groups C and D (Table 1). The incidence of adverse reactions was higher in group A than in the other three groups, especially in the development of osteoporosis and electrolyte disturbances (Table 2).

**Figure:**
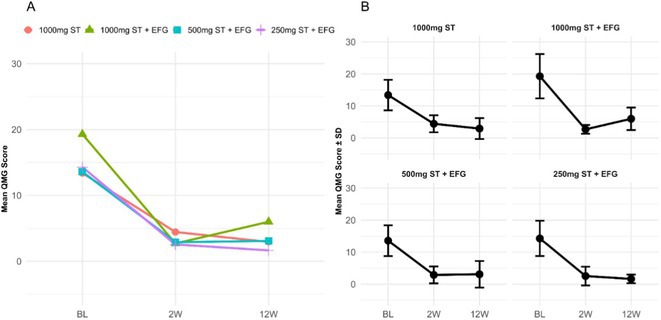
**FIGURE 1** Changes in QMG score during the observation period.

**Figure:**
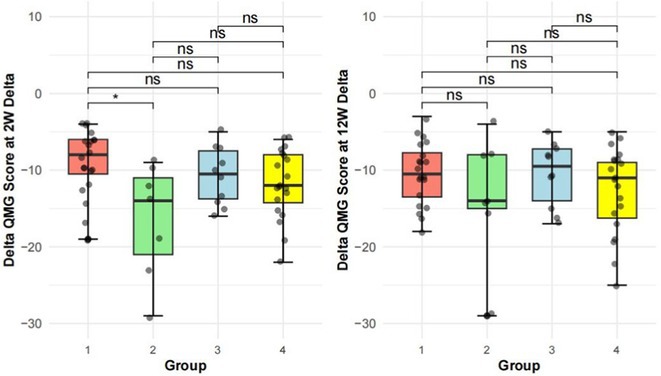
**FIGURE 2** QMG score reductions in various groups.

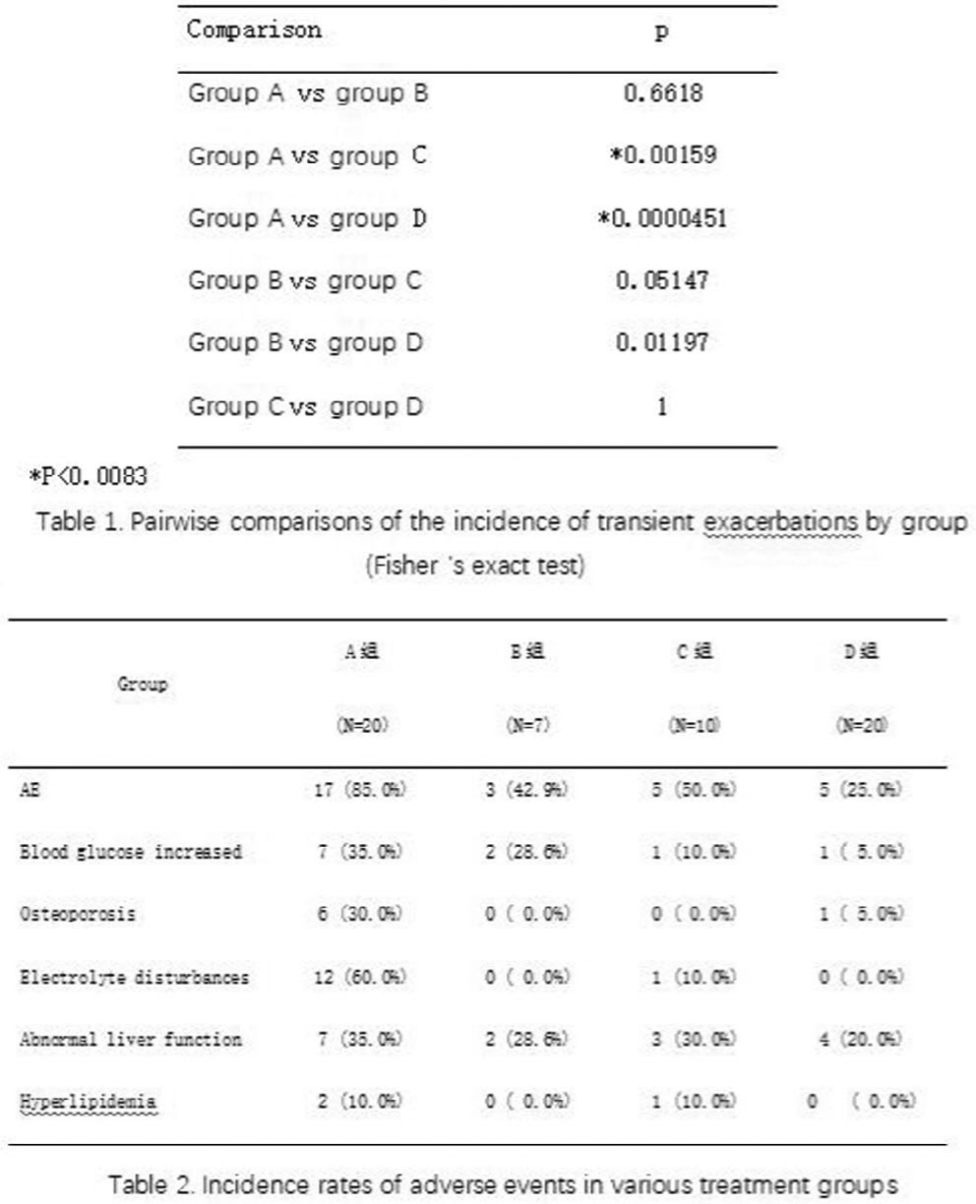

**Conclusion:** Efgartigimod combined with low‐dose IVMP pulse therapy rapidly improved myasthenic symptoms and reduced the transient exacerbations and adverse reactions in severe gMG patients.

**Disclosure:** This work was supported by the National Natural Science Foundation of China (No. 82274582), Central guidance for local scientific and technological development funding projects (No. 246Z7706G), Key Laboratory Construction Subsidy Fund Project (No. 236790017H) S&T Program of Hebei (231201013D).

## EPR‐191

### Do people with Multiple Sclerosis age differently? Reference values for natural killer cell aging and study outline

#### J. Nolte ^1^; H. Vietzen^3^; T. Zrzavy^1^; F. Föttinger^1^; N. Krajnc^2^; E. Puchhammer‐Stöckl^3^; T. Berger^1^; P. Rommer^1^; G. Bsteh^1^

##### ^1^Department of Neurology, Medical University of Vienna, Vienna, Austria; ^2^Comprehensive Center for Clinical Neurosciences and Mental Health, Medical University of Vienna, Vienna, Austria; ^3^Center for Virology, Medical University of Vienna, Vienna, Austria

**Background and aims:** Chronological age is the most consistent factor influencing the observed disease course of people with Multiple Sclerosis (pwMS). We aim to explore differences in biological aging and identify covariates linked to accelerated aging in pwMS. The expression of the marker CD57 on CD56dim natural killer (NK) cells and its proportion of the whole NK population is a well‐established indicator for matured NK cells and may therefore serve as a surrogate marker for biological age.

**Methods:** Peripheral blood mononuclear cells (PBMCs) from healthy blood donors in Switzerland, Austria, and Germany were analyzed using flow cytometry, gating for CD3, CD56, and CD57. Age‐adjusted z‐scores for CD57+CD56dim NK‐cell proportions were estimated. In this ongoing study, PBMCs from 100 pwMS will be collected for comparisons of NK‐cell aging. Potential covariates, including disease duration, treatment duration, and disease‐modifying treatment type, will be evaluated in 80 pwMS across 16 subgroups.

**Results:** Data from 10,437 HCs aged 18‐70 years (mean age: 45.9 years [SD 13.3], 33.6% female) were analyzed. The mean proportion of CD57+CD56dim cells was 38.4% [SD 15.4], and it increased fourfold from individuals younger than 21 to those older than 64 years. On average, the proportion of CD57+CD56dim NK cells increased by 3.9% per year. Adding sex as a covariate did not improve the model fit.

**Conclusion:** CD57+CD56dim NK cells are a robust marker of NK‐cell aging and may indicate biological age, regardless of sex. Based on these findings, age‐adjusted z‐scores can be predicted for pwMS to assess whether biological and chronological age differ.

**Disclosure:** Nothing to disclose.

## EPR‐192

### Regression of pre‐existing white matter hyperintensities after CD19 CAR T‐cell induced ICANS: A case series.

#### M. Borrell‐Pichot ^1^; A. Lozano^2^; E. Granell^2^; A. Caballero^3^; J. Briones‐Mejide^3^; M. Caballero‐Avila^1^; A. Vidal‐Jordana^1^; L. Querol^1^; L. Martín‐Aguilar^1^

##### ^1^Neurology department, Hospital de la Santa Creu i Sant Pau, Barcelona, Spain; ^2^Neuroradiology department, Hospital de la Santa Creu i Sant Pau, Barcelona, Spain; ^3^Haematology department, Hospital de la Santa Creu i Sant Pau, Barcelona

**Background and aims:** Chimeric antigen receptor (CAR T‐cell) therapy has revolutionized the treatment of hematological malignancies, but its use is often limited by toxicity, such as in immune effector cell‐associated neurotoxicity syndrome (ICANS). The mechanisms behind ICANS are still poorly understood, but evidence suggests that cytokines increase blood‐brain barrier (BBB) permeability and a potential off‐target effect of CD19 CAR T‐cells on pericytes. White matter hyperintensities (WMHs) are part of the spectrum of small vessel disease and are often considered irreversible. We present the regression of pre‐existing WMHs in three patients treated with CAR T‐cell therapy who experienced ICANS.

**Methods:** All patients who receive CAR T therapy in our center undergo a baseline brain magnetic resonance imaging (MRI) before infusion as part of our CAR T interdisciplinary protocol. We included three patients with diffuse large B‐cell lymphoma (DLBCL), treated with anti‐CD19 CAR T‐cell therapy, that developed ICANS and in which longitudinal MRI scans were available.

**Results:** We report the regression of WMHs present in the baseline MRI in three patients upon ICANS resolution. The severity of ICANS was heterogeneous: speech impairment appeared in the three patients and encephalopathy in two of them. All patients received levetiracetam and thiamine, two received corticosteroids and one received anakinra. All patients progressed favorably.

**Figure:**
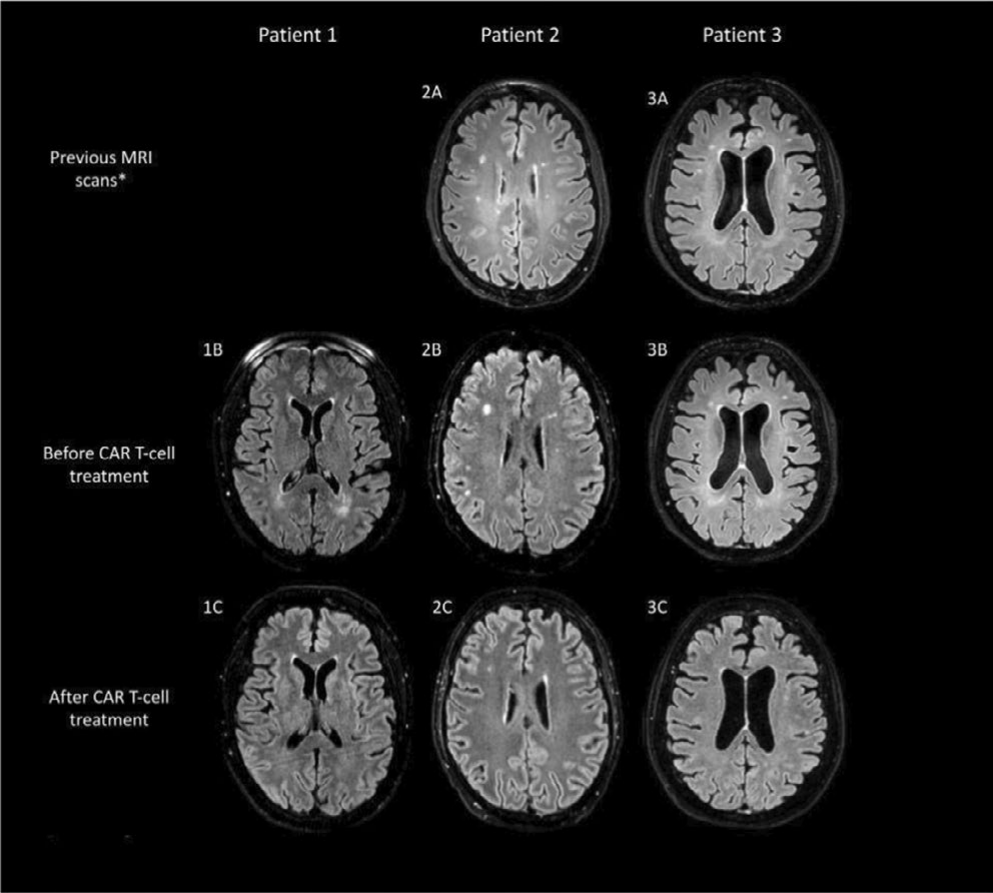
**FIGURE 1** Axial MRI FLAIR of patients 1 to 3. “A” and “B” show subcortical white matter hyperintensities (WMH) in scans prior to CAR T‐cell treatment. “C” shows the disappeareance or decrease in size of the same WMHs, after CAR T‐cell infusion.

**Figure:**
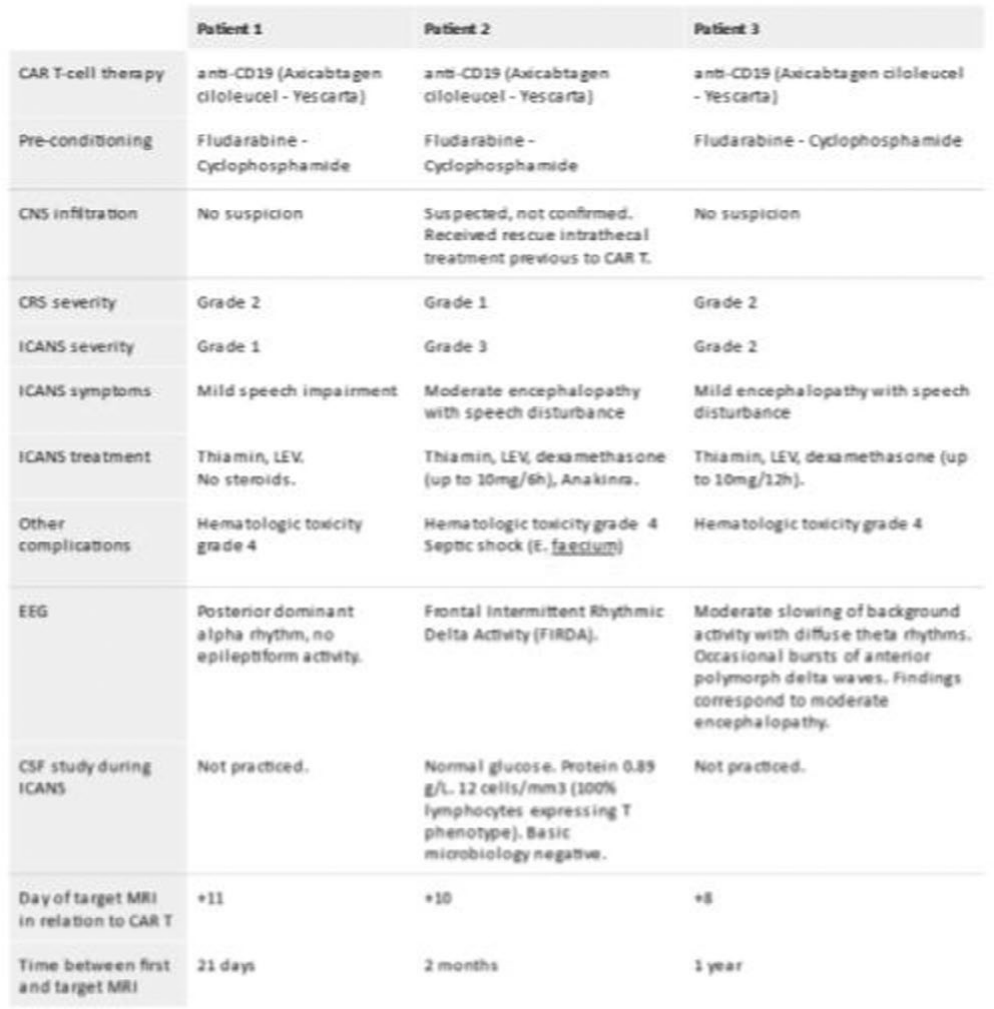
**TABLE 1** Main characteristics of the three patients. CAR, Chimeric Antigen Receptor; CRS, Cytokine Release Syndrome; ICANS, Immune effector Cell Associated Neurotoxicity Syndrome; LEV, levetiracetam.

**Conclusion:** To our knowledge, this is the first report of WMHs disappearance in DLBCL patients after CAR T‐cell therapy. Further research is needed to understand the nature of WMHs in patients with DLBCL and to gain more insight into the mechanisms underlying CAR T‐cell neurotoxicity.

**Disclosure:** Nothing to disclose.

## EPR‐193

### Immunological signature to guide treatment in anti‐NMDAR encephalitis

#### T. Bienfait ^1^; M. Paunovic^1^; S. Ahn^5^; N. Nagtzaam^2^; S. Yee Mon^5^; Y. Jang^5^; M. van Duijn^1^; M. Nagtzaam^1^; D. Bastiaansen^1^; J. Brenner^1^; P. van de Spek^3^; F. Leypoldt^4^; P. Sillevis Smitt^1^; S. Veenbergen^2^; S. Lee^5^; M. Titulaer^1^

##### ^1^Department of Neurology, Erasmus MC University Medical Centre, Rotterdam, The Netherlands; ^2^Department of Immunology, Laboratory Medical Immunology, Erasmus MC University Medical Centre, Rotterdam, The Netherlands; ^3^Department of Clinical Bioinformatics, Erasmus MC University Medical Centre, Rotterdam, The Netherlands; ^4^Department of Neurology, Kiel University, Kiel, Germany; ^5^Department of Neurology, Seoul National University Hospital, Republic of Korea

**Background and aims:** Anti‐N‐methyl‐D‐aspartate receptor (NMDAR) encephalitis is a severe autoimmune disorder with a broad spectrum of clinical manifestations and disease severity. Although many patients significantly improve after first line immunotherapy, others experience refractory disease, highlighting variability in treatment response. This heterogeneity underscores the need to better understand the underlying pathophysiology, and to identify reliable biomarkers to guide therapeutic response.

**Methods:** Using the SOMAscan proteome platform, we identified potential relevant proteins from the CSF of 29 untreated anti‐NMDARE patients and compared these to 15 healthy controls. The results were confirmed using Luminex, initially for two cytokines. Obtaining promising pilot data, STRING database was used to create a 21‐cytokine pane, linked by immunological pathways to the initial two. The 21‐cytokine panel is tested in 75 untreated CSF samples from Dutch and South Korean anti‐NMDAR encephalitis patients.

**Results:** TNFsR‐ll and sL‐Selectin were upregulated in anti‐NMDARE using SOMAscan (median 57,032 vs. 17,487RFU and 107,862 vs. 46,636RFU, respectively, both *p* < 0.0001)), and TNFsR‐II more so in patients with poor prognosis (median 96,913 poor vs. 46,303 good). These results were confirmed by Luminex (median concentration 1,037 vs. 135pg/ml and 11,600 vs. 4,113pg/ml, respectively, *p* < 0.0001), TNFsR‐ll was more outspoken in poor prognosis patients (1,438pg/ml poor vs. 546 good). Full cohort data are currently being analyzed (*n* = 75 anti‐NMDARE patients [54 good, 21 poor outcome], 22 controls)

**Conclusion:** Preliminary data suggest that distinct immune pathways contribute to outcomes in anti‐NMDAR encephalitis. Measuring these signatures in CSF from untreated anti‐NMDAR encephalitis patients might provide the opportunity to apply targeted therapeutic strategies.

**Disclosure:** Nothing to disclose.

## EPR‐194

### Genetic insights into multiple sclerosis, myasthenia gravis and immune‐mediated disorders

#### V. Fominykh; A. Shadrin; P. Jahołkowski; J. Fuhrer; O. Andreassen

##### Institute of Clinical Medicine, University of Oslo, Oslo, Norway

**Background and aims:** Immune‐mediated diseases can be classified as a continuum from pure autoimmune to autoinflammatory with mixed diseases in between [McGonagle, 2006, 2021]. Here we investigate the polygenic continuum of immune‐linked disorders using statistical genetics methods and define genetic architecture focusing on the place of multiple sclerosis (MS) and myasthenia gravis (MG) in the continuum.

**Methods:** We analyzed genome‐wide association studies for MS (*n* cases 14802), MG (*n* cases 1873) and 13 immune‐linked disorders (SLE, SS, SjS, PSC, PBC, AITD, UC, CD, CeD, PS, RA, JIA, T1D) using statistical genetics methods (LDSC, MiXeR, LAVA and genomicSEM) for characterization of overlap and genetic architecture. FUMA and MAGMA were used for functional annotation.

**Results:** GenomicSEM suggested a continuum structure with four underlying factors from autoimmune diseases at one end to inflammatory on the opposite end (Fig. 1). Genetically MG was located between cluster 2 (together with coeliac disease) and cluster 3 disorders. MS shows high correlation and overlap with UC, CD, PSC and PBC (Fig. 2), and these findings show that genetically MS has common genetic background not only with autoimmune but also with inflammatory diseases. We observed a balanced mixture of negative and positive local correlations within the MHC region, while outside this region they were predominantly positive. MAGMA analysis shows genes associated with monogenic immune diseases in diseases with prominent autoimmune and inflammatory components.

**Figure:**
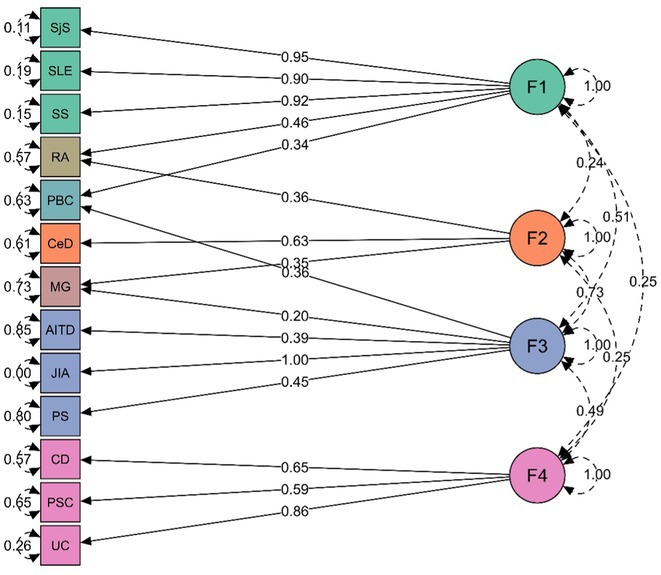
**FIGURE 1** Four groups of immune‐mediated diseases identified in genomic SEM analysis.

**Figure:**
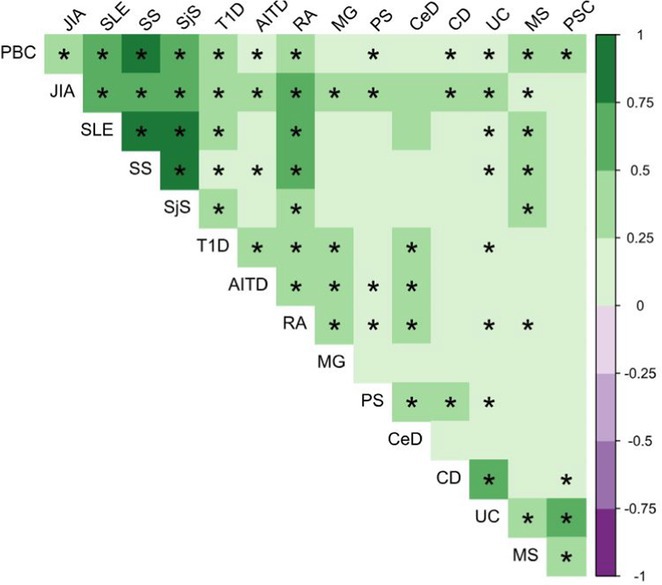
**FIGURE 2** Heatmap of genome‐wide genetic correlations across 15 immune‐mediated diseases.

**Conclusion:** This study explores the genetic aspects of the autoimmune‐autoinflammatory continuum, highlighting the position of MS and MG. The findings provide insights that can inform future research in this field.

**Disclosure:** Disclosures: No special disclosures. This work was supported by an RCN grant 324252.

## Neurotraumatology

## EPR‐195

### Efficacy of prokinetics in preventing pneumonia in post‐stroke patients with nasogastric tubes: A meta‐analysis

#### L. Carretta^1^; P. Teixeira^1^; L. Faria^1^; N. Oliveira^1^; E. Freitas^1^; F. Pinto ^2^; L. Santos^3^; S. Souza^4^; C. Fukunaga^5^; L. Mendes^6^; C. Moura^7^; I. Bezerra^8^; F. Oliveira^8^

##### ^1^Department of Medicine, Escola Superior de Ciências da Santa Casa de Misericórdia de Vitória, Vitória, Brazil; ^2^Department of Medicine, Estácio de Sá University, Rio de Janeiro, Brazil; ^3^Department of Medicine, University Institute of Health Sciences, La Rioja, Argentina; ^4^Department of Medicine, University of São Paulo, São Paulo, São Paulo, Brazil; ^5^Department of Medicine, FMABC University Center, Santo André, Brazil; ^6^Faculty of Medicine of Taubaté, Taubaté, São Paulo, Brazil; ^7^Department of Neurology, Federal Fluminense University, Niterói, Brazil; ^8^Postgraduate Department, Escola Superior de Ciências da Santa Casa de Misericórdia de Vitória, Vitória, Brazil

**Background and aims:** Post‐stroke patients using nasogastric tubes are at increased risk of developing aspiration pneumonia. Prokinetics may help prevent pneumonia by enhancing gastric emptying and reducing gastroesophageal reflux, both of which are risk factors for aspiration. However, studies are controversy regarding the prevention of pneumonia in such patients. Our aim was to evaluate the efficacy of prokinetics in preventing pneumonia in post‐stroke patients using nasogastric tubes.

**Methods:** We systematically searched the literature for randomized controlled trials on Cochrane, PubMed, Embase, Web of Science and Scopus databases from inception to November 2024. The primary efficacy outcome assessed was incidence of pneumonia. Risk of bias was assessed using the RoB 2 and the ROBINS‐1 tools.

**Results:** Of 326 articles screened, 3 randomized controlled trials were included, with a total of 600 patients. The intervention group comprised 291 patients, and the control group comprised 309 patients. The overall risk ratio (RR) for pneumonia in the intervention group compared to the control group was 0.80 (95% CI: 0.50–1.27, *p* > 0.05), indicating no statistically significant reduction in pneumonia risk with the intervention. High heterogeneity was observed among studies (I^2^ = 87%, *p* < 0.001), reflecting substantial variability in outcomes.

**Conclusion:** Current data does not indicate any correlation between the use of prokinetics and the incidence of pneumonia in patients in use of nasogastric tubes. More trials would help with more robust evidence. Currently, there are no formal guidelines or standardized recommendations for their use in this context.

**Disclosure:** All authors report no relationships that could be construed as a conflict of interest. All authors take responsibility for all aspects of the reliability and freedom from bias of the data presented and their discussed interpretation.

## EPR‐196

### MSC‐Exos improve cognitive function in mTBI by inhibiting ferroptosis via the PI3K/AKT/mTOR pathway

#### h. hu

##### Department of Neurology, the First Medical Center, Chinese PLA General Hospital, Beijing, China

**Background and aims:** Mild traumatic brain injury (mTBI) often leads to cognitive dysfunction, yet effective treatments remain scarce. Ferroptosis, a form of regulated cell death, plays a key role in mTBI. This study explores the potential of MSC‐Exos to inhibit ferroptosis and improve cognitive function.

**Methods:** mTBI was induced in rats using a weight‐drop model. MSC‐Exos (50 μg, 100 μg, 200 μg) were administered. Cognitive function was assessed using NOR and MWM tests. Lipid peroxidation (MDA, 4‐HNE) and Gpx4 expression were measured to evaluate ferroptosis.

**Results:** MSC‐Exos improved cognitive function in mTBI rats, with the 200 μg group showing the best results. MSC‐Exos reduced lipid peroxidation (MDA, 4‐HNE) and restored Gpx4 expression, inhibiting ferroptosis and enhancing cognitive performance.

**Figure:**
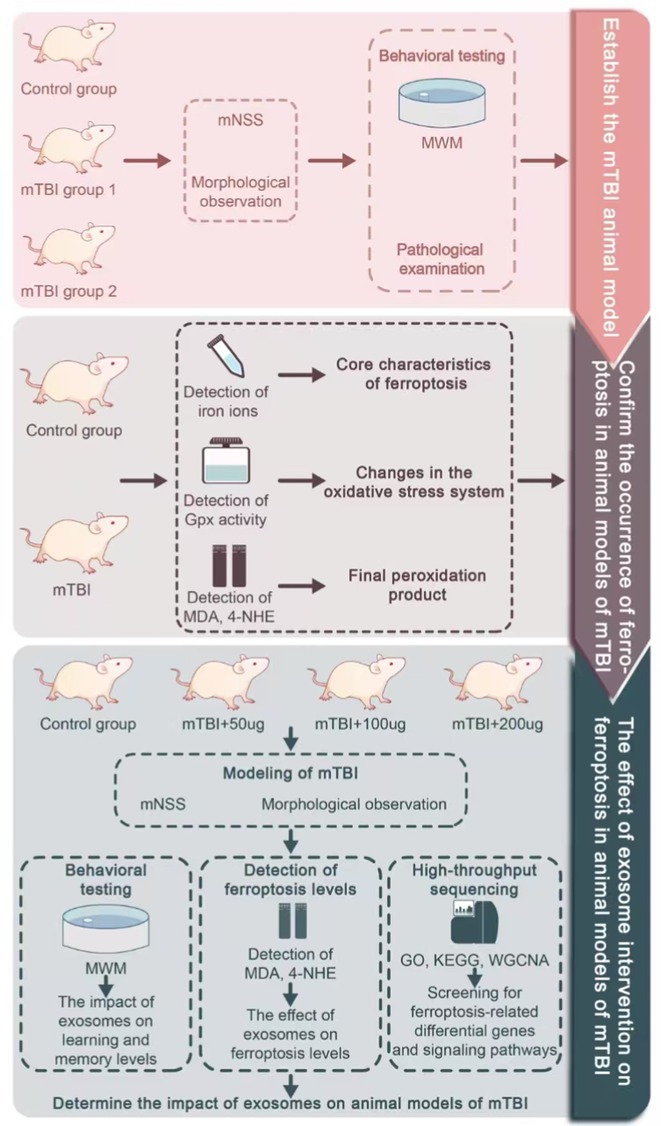
**FIGURE 1** Behavior test

**Figure:**
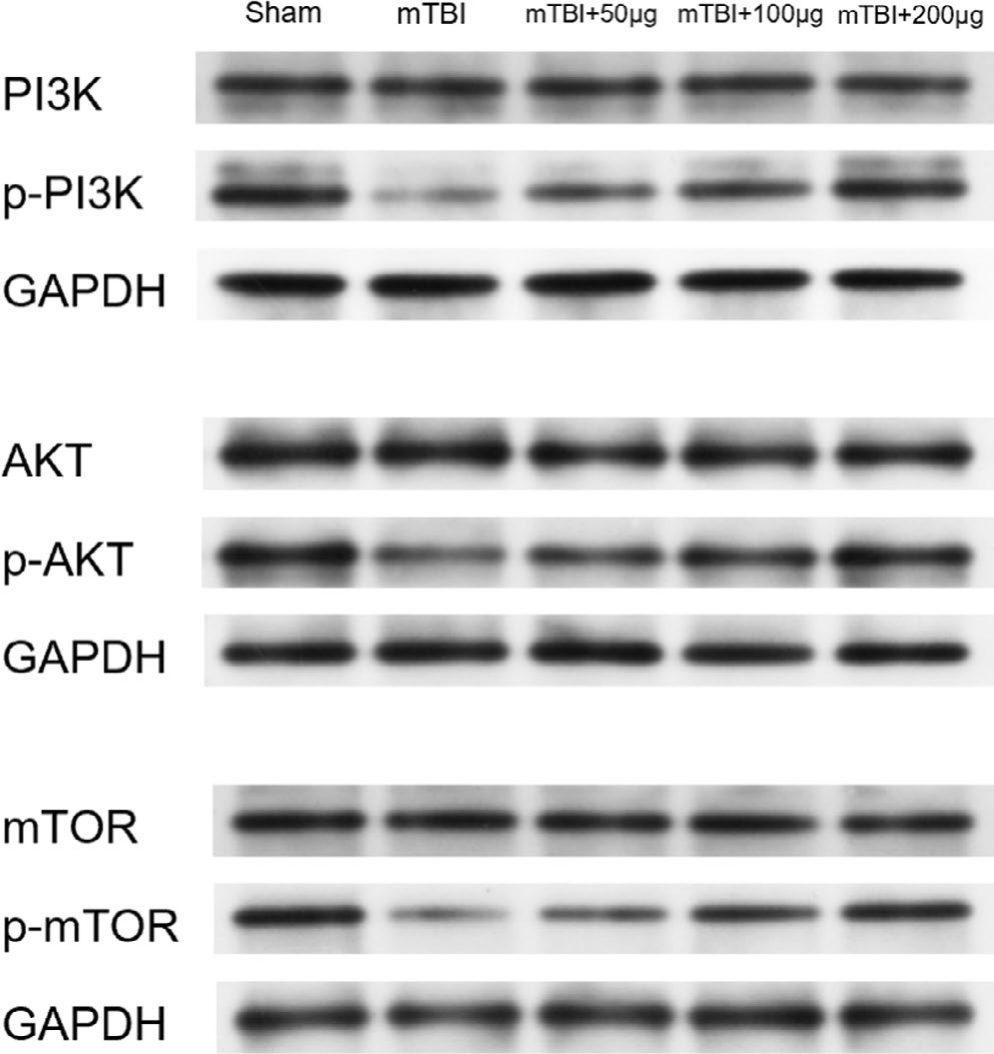
**FIGURE 2** Western Blot Test

**Conclusion:** MSC‐Exos inhibit ferroptosis and improve cognitive function in mTBI by activating the PI3K/AKT/mTOR pathway, offering a promising therapeutic approach for mTBI.

**Figure:**
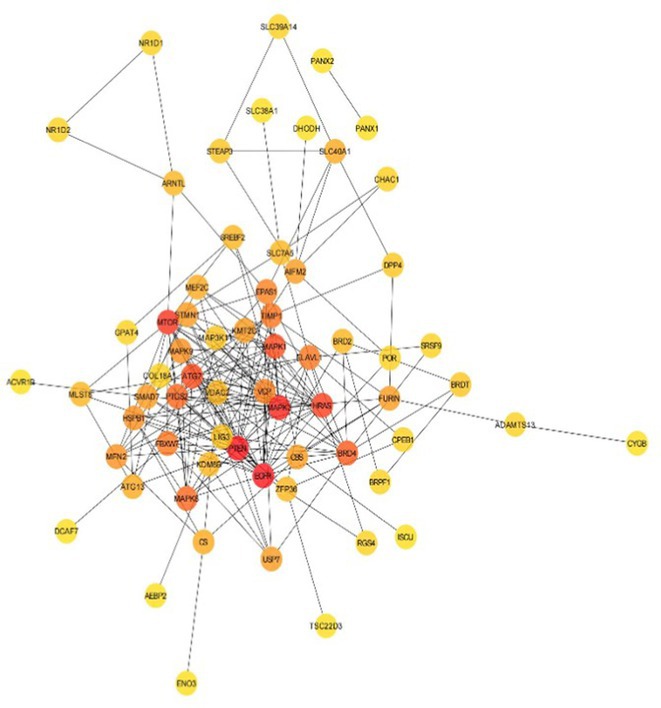
**FIGURE 3** PPI network

**Disclosure:** The authors declare no conflict of interest.

## EPR‐197

### Safety and efficacy of ALMB‐0166 in patients with acute spine cord injury (SCI): A randomized, phase I/II study

#### J. Liang ^1^; C. Li^2^; M. Jin^2^; S. Ni^2^; Y. Yang^2^; Y. Zhang^2^; Q. Wang^2^; G. Qiu^1^

##### ^1^Department of Orthopedics, Peking Union Medical College Hospital, Chinese Academy of Medical Sciences & Peking Union Medical College; ^2^CSPC Pharmaceutical Group, Ltd

**Background and aims:** ALMB‐0166 is a first‐in‐class humanized monoclonal antibody that block connexin‐43 hemichannels in spinal cord astrocytes, which have the potential to prevent further injuries following SCI and promote neurological recovery.

**Methods:** Eligible patients had an acute SCI with American Spinal Injury Association (ASIA) levels at C3 and below and ASIA impairment scale (AIS) B or C, and were within 72 hours after injury. Patients were randomized to receive a single intravenous infusion of ALMB‐0166 200, 600 (2:1) and 1200, 2400, 4800 mg (3:1) or placebo with best supportive care. Primary outcome was safety.

**Results:** 24 patients with C3‐C7 SCI received treatment (17 with ALMB‐0166, 7 with placebo). Treatment emergent adverse events (TEAEs) occurred in 94.1% (16/17) and 100% (7/7) of ALMB‐0166 and placebo treated patients. ≥Grade 3 TEAEs were 17.6% (3/17) with ALMB‐0166 and 42.9% (3/7) with placebo, commonest being hypokalemia, pulmonary inflammation, respiratory failure, and deep venous thrombosis (*n* = 1) with ALMB‐0166 and hypokalemia (*n* = 2), hyponatremia (*n* = 1) with placebo. At day 56, patients treated with ALMB‐0166 had improvement compared with those given placebo in motor function (scores increased from baseline by 66.0 for 2400 mg and 45.6 for placebo), sensatory function (scores increased by 77.5, 62.7 for 600, 1200 mg and 52.3 for placebo), AIS (2 patients recovered to grade E for ALMB‐0166 and 0 patient for placebo), and pain (VAS scores decreased by 44.3, 23.7 for 1200, 2400 mg and 20.9 for placebo).

**Conclusion:** ALMB‐0166 demonstrated great safety profile and improved neurologic recovery in patients with acute SCI.

**Disclosure:** Chao Li, Miao Jin, Shaonan Ni, Yumei Yang, and Qingxi Wang are employees of CSPC Pharmaceutical Group, Ltd. Yanfeng Zhang is an employee of AlaMab Therapeutics Inc. Other authors have nothing to disclose.

## EPR‐198

### Liberal vs. restrictive transfusion strategy in patients with acute brain injury: A systematic review and meta‐analysis

#### A. Menegaz de Almeida^1^; F. Alves de Paiva^2^; F. Alves Kelly^3^; J. Martinez‐Lemus^4^; C. Rocha Dantas^5^; A. Boris de Mesquita^5^; L. Violeta Rodrigues de Matos^6^; K. Violeta Rodrigues de Matos^7^; A. Machado Silva^8^; H. Roberto Moreira de Oliveira Carriço^9^; J. Patino^10^; V. Vyas^10^; L. Fernandes Torres^11^; M. Carolina Chaves do Vale ^12^

##### ^1^Medicine, Federal University of Mato Grosso, Sinop, Brazil; ^2^Medicine, Federal University of Amazonas, Manaus Brazil; ^3^Cardiology, Dante Pazzanese Institute of Cardiology, São Paulo, Brazil; ^4^Department of Neurology, UTHealth Houston McGovern Medical School, Houston, Texas, USA; ^5^Medicine, Universidad de Buenos Aires, Buenos Aires, Argentina; ^6^Medicine, Federal University of Amazonas, Manaus, Brazil; ^7^Medcine, Metropolitan University of Manaus, Manaus, Brazil; ^8^Medicine, University City of São Paulo, Brazil; ^9^Medicine, University of Southern Santa Catarina, Tubarão, Brazil, ^10^Neurology, UTHealth Houston McGovern Medical School, Houston, Texas, USA, ^11^Vivian L. Smith Department of Neurosurgery, University of Texas Health Science Center at Houston ‐ McGovern Medical School, Houston, Texas, USA, ^12^Medicine, Catholic University of Pernambuco, Recife, Brazil

**Background and aims:** Brain injury is the leading cause of death and disability worldwide. Anemia is common among brain injury patients, with transfusion of red blood cells (RBC) often being required. Evidence is lacking to determine the preferred hemoglobin (Hb) threshold for transfusion, whether a restrictive (< 7 g/dL) strategy or a liberal approach (< 10 g/dL).

**Methods:** MEDLINE, Web of Science, SCOPUS, and Cochrane databases were searched for randomized clinical trials (RCTs) comparing the restrictive transfusion method with a liberal threshold. RCTs published up to December 9, 2024 were eligible. Differences between groups were estimated using the Mantel‐Haenszel method; heterogeneity was assessed using I2 statistics.

**Results:** 6 RCTs with data from 2599 patients were included. Statistically significantly better rates of neurological recovery were found in the liberal transfusion group [GOS‐5; 21.1% vs. 16.3%; OR, 1.39; 95% CI, 1.13–1.72; *p* = 0.001; I^2^ = 0%]. The restrictive group was found to have higher rates of remaining in a vegetative state [GOS‐2; 1.7% vs. 3.2%; RR, 0.54; 95% CI, 0.29–1.00; *p* = .050; I^2^ = 0%] and higher rates of sepsis/septic shock [6.4% vs. 9%; RR, 0.73; 95% CI, 0.56–0.95; *p* = .020; I^2^ = 0%]. Higher Hb levels at enrollment and more units of RBC units used per patient were associated with a lower rate of GOS‐3.

**Figure:**
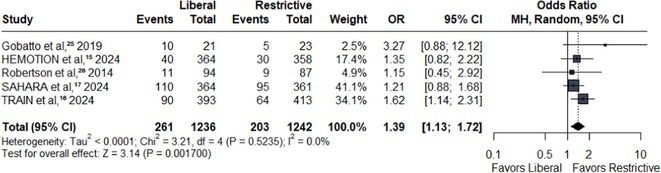
**FIGURE 1** Glasgow Outcome Scale 5 (GOS), reporting good neurologic recovery in six months

**Figure:**
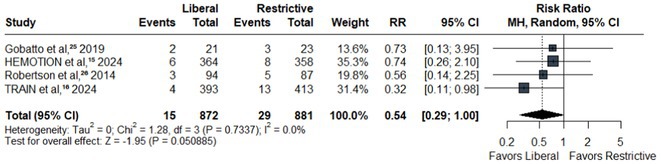
**FIGURE 2** Glasgow Outcome Scale 2 (GOS), reporting patients remaining in a vegetative state in six months

**Figure:**
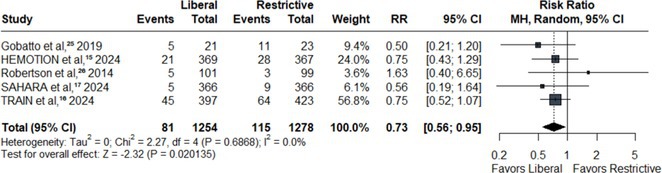
**FIGURE 3** Sepsis or septic shock occurrence

**Conclusion:** This meta‐analysis establishes the liberal strategy as having a better rate of neurological recovery and safety compared to a restrictive approach for managing acute brain injury patients.

**Disclosure:** Nothing to disclose.

## EPR‐201

### Evaluating stroke recognition and time metrics in Emergency Dispatch: Comparing mimics versus true strokes/TIA

#### N. Leto ^1^; E. Farbu^2^; P. Barach^3^; C. Bjørshol^1^; M. Kurz^3^; A. Fromm^4^; C. Bjerkvig^5^; T. Lindner^1^

##### ^1^The Regional Centre for Emergency Medical Research and Development in Western Norway (RAKOS), Stavanger, Norway; ^2^Department of Neurology, Neuroscience Research Group, Stavanger University Hospital, Stavanger, Norway; ^3^School of Medicine, Thomas Jefferson University, Philadelphia, USA; ^4^Department of Neurology, Haukeland University Hospital, Bergen, Norway; ^5^Department of Emergency Care, Haukeland University Hospital, Bergen, Norway

**Background and aims:** Recognition of acute stroke by the Emergency Medical Dispatch Centre (EMDC) can lead to efficient management, yet many alerts are false due to stroke mimics. We aimed to quantify EMDC over‐triage by comparing stroke mimics and confirmed stroke cases.

**Methods:** This retrospective study included patients with a suspected stroke admitted via the EMDC in Western Norway from January 1, 2021, to December 31, 2022. Data on EMDC dispatch criteria, prehospital time metrics, and discharge diagnoses were collected. The EMDC used the Norwegian Medical Priority Dispatch System (MPDS) algorithm with eight stroke suspicion criteria.

**Results:** Of 8259 patients, 7080 (86%) were stroke mimics and 1181 (14%) were confirmed stroke cases: 15 (1%) transient ischemic attack (TIA), 998 (85%) acute ischemic stroke (AIS), and 168 (14%) intracerebral hemorrhage (ICH). Five EMDC criteria had stroke mimic rates > 90%, including breathing problems (96%), acute dizziness (94%), acute hemianopsia (91%), acute ataxia/confusion (94%), and acute headache (95%). The Face‐Arm‐Speech (FAST) criteria had higher confirmed stroke rates compared to the five other EMDC criteria (OR 2.82, 95% CI 1.69‐4.67). Median EMS response times were similar for mimics and confirmed strokes (10 vs. 9 min; *p* = 0.47), but on‐scene times were longer for mimics (16 vs. 10 min; *p* < 0.001).

**Conclusion:** EMDC stroke dispatch criteria have low positive predictive value. EMDC FAST dispatch criteria were associated with higher specificity in stroke recognition. Confirmed stroke diagnoses correlate with shorter EMS on‐scene times. Our findings indicate the need for new EMDC stroke assessment tools, although requiring validation in prospective studies.

**Disclosure:** Nothing to disclose.

## EPR‐202

### Laboratory diagnostics of rhinoliquorrhea – Re‐evaluation of cut‐off values of beta‐trace protein

#### M. Haindl^1^; V. Vershinin^1^; A. Ferstl‐Rohrbacher^1^; J. Archelos^1^; T. Keck^2^; M. Gugatschka^3^; S. Wolfsberger^4^; M. Khalil^1^; C. Enzinger^1^; S. Hochmeister ^1^

##### ^1^Department of Neurology, Medical University of Graz, Austria; ^2^Döbling Private Hospital Vienna, Outpatient center for Otorhinolaryngology and Sleep medicine, Vienna, Austria; ^3^Department of Otorhinolaryngology, Medical University Graz, Austria; ^4^Department of Neurosurgery, Medical University Graz, Austria

**Background and aims:** Cerebrospinal fluid rhinorrhea (CSFR) occurs when cerebrospinal fluid (CSF) leaks through a bony defect into the nasal cavity. This condition poses a significant risk of bacterial meningitis, necessitating timely and accurate diagnosis. Beta‐trace protein (BTP) is the most widely used biomarker for detecting CSF contamination in nasal mucus, but diagnostic reliability is hindered by inconsistent cut‐off values, ranging from 0.24 to 6.00 mg/L in the literature. This study re‐evaluates these cut‐offs to improve diagnostic precision.

**Methods:** We analyzed BTP levels using nephelometry in pure ventricular and lumbar CSF, serum, and nasal mucus samples from 265 patients with clinically suspected CSFR. Data were further assessed through experimental setups, including pure sample measurement, laboratory modeling of CSF contamination, and retrospective analysis of clinical cases. Receiver operating characteristic (ROC) analyses were performed to determine optimal cut‐off values.

**Results:** BTP concentrations were significantly higher in lumbar CSF (25.10 mg/L) than ventricular CSF (6.70 mg/L, *p* < 0.001), and higher in serum (0.46 mg/L) compared to nasal mucus (0.29 mg/L, *p* < 0.001). ROC analysis identified 1.40 mg/L as the most feasible cut‐off for nasal mucus BTP, achieving 100% sensitivity and 94% specificity. Incorporating serum BTP levels did not enhance diagnostic accuracy.

**Conclusion:** A cut‐off of 1.40 mg/L for nasal mucus BTP provides high diagnostic reliability. However, multi‐center studies are needed to validate these findings across various laboratory systems. Interim recommendations include tailoring cut‐offs to specific laboratory protocols to optimize diagnostic validity.

**Disclosure:** The study was approved by the ethics commission of the Medical University of Graz (EK 32‐149 ex 19/20). MTH declares no conflict of interest. VV declares no conflict of interest. AFR declares no conflict of interest. JJA declares no conflict of interest. TK declares no conflict of interest. CE declares no conflict of interest. SW declares no conflict of interest. MG declares no conflict of interest. SH declares no conflict of interest. MK has received travel funding and speaker honoraria from Bayer, Biogen, Novartis, Merck, Sanofi and Teva and serves on scientific advisory boards for Biogen, Bristol‐Myers Squibb, Gilead, Merck, Neuraxpharm, Novartis, Alexion, Amgen and Roche. He received research grants from Biogen, Novartis and Teva.

## Movement disorders 3

## EPR‐203

### Post‐hoc analysis of the GUT‐PARFECT trial for fecal microbiota transplantation in Parkinson's disease

#### A. Bruggeman ^1^; H. Hamerlinck^2^; C. Vandendriessche^3^; D. De Looze^4^; J. Raes^5^; B. Verhasselt^2^; D. Laukens^6^; R. Vandenbroucke^3^; P. Santens^1^

##### ^1^Department of Neurology, University Hospital Ghent, Ghent, Belgium; ^2^Department of Laboratory Medicine, Ghent University Hospital, Ghent, Belgium; ^3^VIB‐UGent Center for Inflammation Research, VIB, Ghent, Belgium; ^4^Department of Gastroenterology, University Hospital Ghent, Ghent, Belgium; ^5^VIB‐KU Leuven Center for Microbiology, VIB, Leuven, Belgium; ^6^Faculty of Medicine and Health Sciences, Ghent University, Ghent, Belgium

**Background and aims:** Dysregulation of the gut microbiome has been implicated in Parkinson's disease (PD). The recently published GUT‐PARFECT trial evaluated the clinical effects and safety of a single fecal microbiota transplantation (FMT) in patients with early‐ stage PD. Mild, but long‐lasting beneficial effects on motor symptoms and colon transit time were reported. This exploratory post hoc analysis aimed to investigate which factors might predict the magnitude of response to FMT.

**Methods:** Univariate and multivariate analyses were conducted to determine possible predictors of motor response to FMT. Responders were defined by an improvement of more than 3.25 on the Movement Disorders Society‐Unified Parkinson's Disease Rating Scale (MDS‐UPDRS) part 3 motor score, which is considered a clinically relevant difference. Potential treatment by subgroup interactions for changes in the major outcomes were evaluated among post hoc subgroups defined by age, age at diagnosis, disease duration, sex, motor phenotype, and presence of certain non‐motor symptoms (e.g., constipation, REM sleep behavior disorder). Gut microbiota analysis was performed through 16S rRNA sequencing.

**Results:** FMT responders were more likely to have shorter colon transit time, less REM sleep behavior disorder, less motor fluctuations, and less severe non‐motor symptoms at baseline. Greater alpha diversity of gut microbiota (measured by the Shannon index) at baseline was associated to a more beneficial response to FMT. Individual healthy donors that provided the stool samples for treatment could not predict response in their respective acceptors.

**Conclusion:** The results of this exploratory post hoc analysis can guide future planned clinical trials of FMT in PD.

**Disclosure:** Nothing to disclose.

## EPR‐204

### Patient‐reported outcomes, psychosocial health and healthcare utilization in patients with friedreich ataxia

#### M. Grobe‐Einsler^1^; S. Borel^2^; M. Buchholz^3^; S. Sayah^2^; R. Hilab^2^; A. Iskandar^3^; B. Humphries^4^; K. Schirduan^5^; A. Durr^2^; T. Klockgether^1^; F. Xie^4^; B. Michalowsky ^3^

##### ^1^German Center for Neurodegenerative Diseases, Bonn, Germany; ^2^Paris Brain Institute (ICM ‐ Institut du Cerveau), INSERM, CNRS, Assistance Publique‐Hôpitaux de Paris (AP‐HP), Sorbonne University, Paris, France; ^3^Patient‐reported Outcomes & Health Economics Research, German Center for Neurodegenerative Diseases, Site Rostock/Greifswald, Greifswald, Germany; ^4^Department of Health Research Methods, Evidence and Impact, McMaster University, Hamilton, Ontario, Canada; ^5^Biogen GmbH, Munich, Germany

**Background and aims:** Friedreich's ataxia (FA) is a rare neurodegenerative disorder characterized by worsening movement and speech impairments. This study aimed to assess FAs impact on patient‐reported, psychosocial, and health service outcomes.

**Methods:** Within the PROFA study, we assessed 102 FA patients in France, Germany, and Austria, including a baseline study center and remote mobile assessment, capturing disease severity (SARA), daily living deficits (FARS‐ADL), cognitive and affective impairments (CCAS), health‐related quality of life (HRQoL: PROM‐ATAX, EQ‐5D‐5L), mental well‐being (WEMWEBS), hearing (HearWHO) and communication disabilities (COMATAX) and healthcare service and informal care utilization. Spearman correlations and multivariate regressions were used to evaluate associations between outcomes and between outcomes and disease severity, respectively.

**Results:** 33% of patients received formal and 61% informal care (on average, 12.6 hours/week). 32%, especially females and younger patients with higher daily living deficits and lower disease severity, Omaveloxolone, the first approved FA treatment. Daily living deficits (rs = 0.707), communication disabilities (rs = 0.630), HRQoL (rs = 0.569) correlated strongly, and cognitive impairment (rs = ‐0.317), informal care provision (rs = 347), and hearing problems (rs = 0.346) moderately with disease severity. Further associations were found for daily living deficits with HRQoL (rs = 0.6518) and communication disabilities (rs = 0.567). Regression analyses showed a significant worsening of communication (b = 0.58), HRQOL (b = 0.57), mental well‐being (b = ‐0.30), daily living activities (b = 0.75), cognitive abilities (b = ‐0.41), informal care dependency (b = 0.38), and hearing (b = 0.16) with increasing disease severity.

**Conclusion:** Results emphasize FA's multidimensional burden and the need for comprehensive care addressing physical and mental health. The reliance on informal care underscores FA's societal and economic burden.

**Disclosure:** Ksenija Schirduan is an employee and may hold stock in Biogen.

## EPR‐205

### Global approaches to device‐aided therapies in Parkinson's disease

#### D. Ledingham ^1^; C. Stewart^1^; S. Sathyanarayana^1^; V. Foster^1^; R. Iredale^1^; D. Galley^1^; M. Baker^2^; N. Pavese^1^

##### ^1^Clinical Ageing Research Unit, Newcastle University, Campus for Ageing and Vitality, Newcastle Upon Tyne, UK; ^2^Translational and Clinical Research Institute, The Medical School, Newcastle University, Newcastle Upon Tyne, UK

**Background and aims:** Patients with Parkinson's Disease (PwPD) who develop medication‐refractory motor complications and/or tremor may undergo treatment with Device‐Aided Therapies (DAT). These therapies include surgical options such as Deep Brain Stimulation (DBS), MRI‐guided Focused Ultrasound (MRgFUS), and infusion therapies. There is limited information on the factors influencing the timing and global preference for these treatments during the disease course.

**Methods:** We analyzed clinical, demographic, and genetic data from a large cohort of PwPD in the Parkinson's Progression Markers Initiative (PPMI) study.

**Results:** At the time of analysis, a total of 1291 PwPD had participated in the PPMI study. PwPD had been followed up for up to 14 years. During follow‐up, 8.1% of PwPD underwent treatment with DAT, with the majority using DBS. This cohort includes patients from 52 different sites internationally. Genetics, dominant clinical symptoms, and site preference appear to influence the selection timing for DAT.

**Conclusion:** DBS was the most common form of DAT used in this cohort of patients. Understanding the current global use of DAT and the factors that influence the selection for early DAT has important health‐economic significance and can help optimize treatment strategies for PwPD.

**Disclosure:** Nothing to disclose.

## EPR‐206

### Long‐term effectiveness of foslevodopa/foscarbidopa vs. real‐world standard of care in advanced Parkinson's disease

#### D. Standaert^1^; F. Ory‐Magne^2^; D. Kern^3^; O. de Fabregues^4^; L. Defebvre^5^; T. Oeda^6^; D. Safarpour^7^; A. Parab^8^; C. Yan^8^; S. Wang^8^; K. Onuk ^8^; P. Kukreja^8^; L. Bergmann^8^; V. Fung^9^

##### ^1^Center for Neurodegeneration and Experimental Therapeutics, University of Alabama at Birmingham, Birmingham, USA; ^2^Department of Neurology, University Hospital of Toulouse, Toulouse, France; ^3^University of Colorado School of Medicine, Departments of Neurology and Neurosurgery, Aurora, USA; ^4^Department of Neurology, Vall d’Hebron Hospital Universitari, Universitat Autònoma de Barcelona, and Neurodegenerative Diseases Laboratory, Vall d’Hebron Institut de Recerca (VHIR), Vall d'Hebron Barcelona Hospital Campus, Barcelona, Spain; ^5^Lille Neurosciences & Cognition, Movement Disorders Department, CHU Lille, Lille University, Lille, France; ^6^Clinical Research Centre and Department of Neurology, National Hospital Organization Utano National Hospital, Kyoto, Japan; ^7^Department Neurology, Oregon Health Science University Portland, Oregon, USA; ^8^AbbVie Inc, North Chicago, USA; ^9^Westmead Hospital, Westmead, Australia and Sydney Medical School, University of Sydney, Sydney, Australia

**Background and aims:** People with advancing Parkinson's disease(PwaP) experience motor fluctuations despite optimized oral therapies(standard of care;SoC). Foslevodopa/foscarbidopa(LDp/CDp) showed greater motor symptom improvements than oral immediate‐release levodopa/carbidopa in a 3month(M) randomized controlled trial. This study evaluated long‐term comparative effectiveness of LDp/CDp versus real‐world SoC in PwaP over 24M.

**Methods:** PwaP receiving LDp/CDp in a 12M open‐label study(NCT03781167) plus open‐label extension (NCT04379050) were indirectly compared to PwaP receiving SoC in a 24M prospective observational study(PROSPECT). Analyses included completers and discontinuers to maximize statistical power and reduce selection bias. Imputed and non‐imputed analyses were conducted. Change from baseline and achievement of minimum clinically important differences(MCID) between treatment cohorts were evaluated for motor symptoms(PD diary), QoL(PDQ‐39,EQ‐5D‐5L), daily living(MDS‐UPDRS II), and sleep disturbances(PDSS‐2) at 6M intervals. Comparative measures were adjusted for baseline characteristics and outcomes.

**Results:** Analyses included 129 LDp/CDp‐ and 185 SoC‐treated patients (Table 1). LDp/CDp showed significantly greater improvements for OFF and good ON time over 24M and PDSS‐2 over 12M, compared with SoC (Table 2). At all points, ≥66% of LDp/CDp and ≤49% of SoC achieved MCID for these outcomes (Table 3). LDp/CDp showed greater improvements in MDS‐UPDRS II, PDQ‐39, and EQ‐5D‐5L than SoC; effects decreased over time in both cohorts, with SoC worsening (Table 2). Overall, 42%‐61% of LDp/CDp versus 13%‐22% of SoC achieved MCID for these outcomes (Table 3). Results were similar between imputed and non‐imputed analyses.

**Figure:**
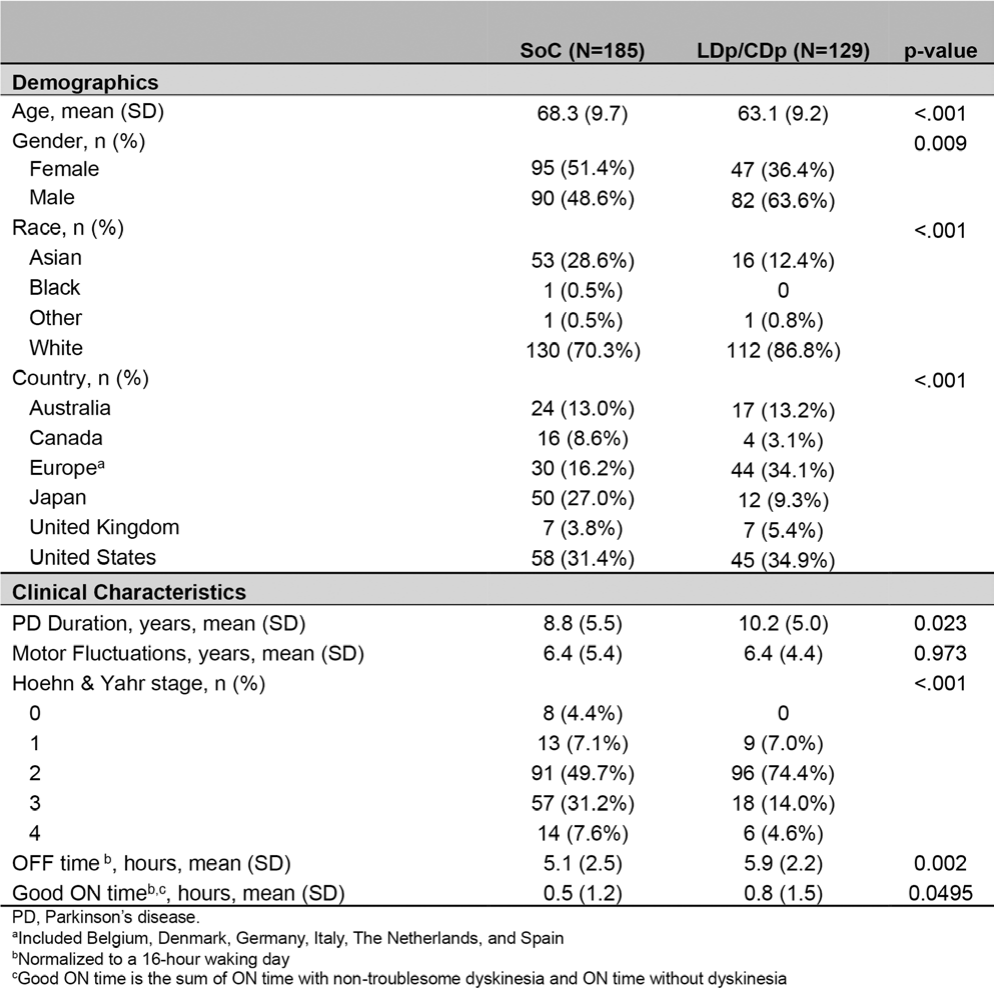
**TABLE 1** Baseline demographics and clinical characteristics.

**Figure:**
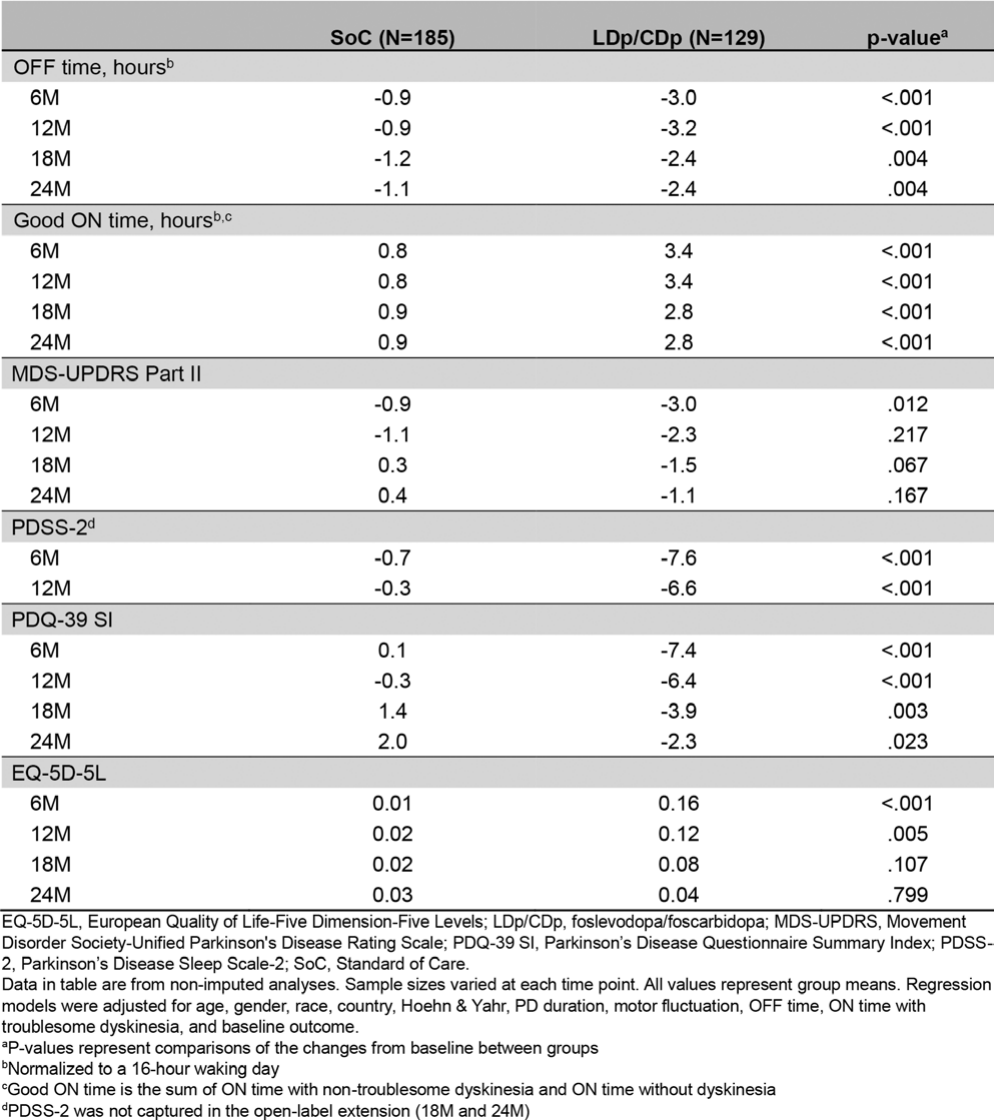
**TABLE 2** Mean change from baseline in clinical outcomes.

**Figure:**
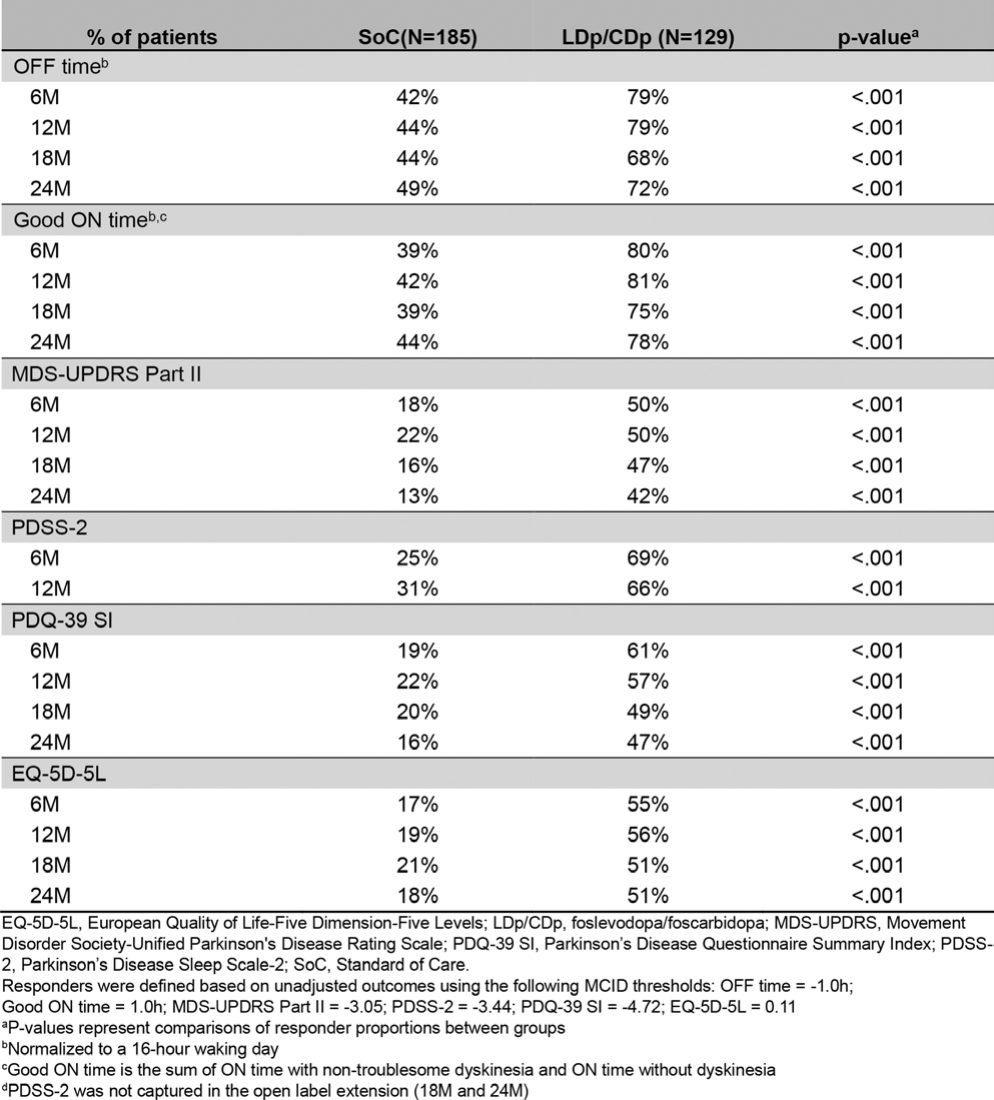
**TABLE 3** Proportion of Responders Achieving MCID for Clinical Outcomes

**Conclusion:** LDp/CDp showed greater and sustained improvements in motor symptoms, sleep, and QoL than SoC in PwaP. Results suggest meaningful advantages of LDp/CDp as PD advances where SoC impact is diminished.

**Disclosure:** DS: University of Alabama at Birmingham, University of Alabama Health Services Foundation, American Parkinson Disease Association, Michael J. Fox Foundation, National Parkinson Foundation, Alabama Department of Commerce, Alabama Innovation Fund, Genetech, Department of Defense, NIH, International Parkinson and Movement Disorders Society, AbbVie, Alnylam Pharmaceutics, Appello, Biohaven Pharmaceuticals, BlueRock Therapeutics, Coave Therapeutics Curium Pharma, F. Hoffman‐La Roche, Eli Lilly USA, Sanofi‐Aventis, Theravance, McGraw‐Hill. FOM: AbbVie, Aguettant, Actelion, Univar, Orkyn, France‐Parkinson Association, Ministère des solidarités et de la santé, LVL Medical, Medtronic, NHC France. DK: AbbVie, Abbott, Boston Scientific, Colorado Clinical and Translational Sciences Institute Data Safety Monitoring Board, Medtronic, Parkinson's Foundation, University of Colorado Department of Neurology. OdF: AbbVie, Alexion, Orphalan, Esteve, Italfarmaco. LD: AbbVie, Aguettant, Orkyn. TO: AbbVie, Takeda, Eisai, Ono, Kyowakirin, FP Pharma, Japan Blood Products Organization. DS: Medtronic; Boston Scientific; AbbVie. AP, CY, SW, KO, PK, LB: employees of AbbVie. VSCF: NSW Health, Michael J. Fox Foundation, AbbVie, Merz, Health Press Ltd, Taylor&Francis Group. Commercial support: study funded by AbbVie who participated in the study design; study research; collection, analysis, interpretation of data; and writing, reviewing, approving this abstract.

## EPR‐207

### Deep brain stimulation in neurological features of Lesch‐Nyhan syndrome: A critical review of literature

#### G. Cabral

##### Neurology Department, Hospital de Egas Moniz, Unidade Lisboa Local de Saúde, Lisbon, Portugal.

**Background and aims:** Lesch‐Nyhan syndrome (LNS) is a rare X‐linked metabolic disorder characterized by hyperuricemia, motor dysfunction (primarily dystonia), and self‐injurious behavior (SIB), often resistant to medical treatment. Deep brain stimulation (DBS) has emerged as an alternative for managing refractory dystonia and SIB in LNS patients.

**Methods:** A comprehensive analysis of reported LNS cases treated with DBS was conducted, focusing on demographics, motor and behavioral outcomes, adverse events, and follow‐up durations.

**Results:** Twenty‐eight cases of DBS targeting the Globus Pallidus Internus (GPi) were identified 53.6% with 2 electrodes, and 46.4% with 4 electrodes). Patients (all male) had a mean age of 12 years (SD 6, range 5–28) at surgery, with a median follow‐up of 3.75 years (range 0.5–12). Dystonia was the most common motor feature, and all but one patient exhibited SIB. DBS led to motor improvements in 64% of cases, with reductions in dystonia ranging from 16% to 75%, based on the Burke‐Fahn Marsden Dystonia Rating Scale or caregiver assessment, especially with posterolateral GPi targeting. Behavioral outcomes included reduced SIB, improved impulsivity, and better caregiver‐reported comfort. Complications occurred in 57% of cases, including infections, hardware failures, and electrode displacement, but behavioral improvements often persisted.

**Conclusion:** DBS targeting GPi shows promise in treating severe motor and behavioral symptoms in LNS. Long‐term follow‐up and standardized assessments are critical to optimize patient outcomes while mitigating adverse events.

**Disclosure:** Nothing to disclose.

## EPR‐208

### Sleep‐related complaints and early morning dystonia in Parkinson's patients receiving opicapone: Results from OASIS

#### J. Ferreira^1^; M. Gago^2^; R. Costa^3^; M. Fonseca^4^; H. Brigas ^3^; J. Holenz^3^; C. Trenkwalder^5^

##### ^1^IMM ‐ Instituto de Medicina Molecular João Lobo Antunes, Faculdade de Medicina, Universidade Lisboa, Lisbon, Portugal; ^2^Department of Neurology, ULS Alto Ave, Hospital da Senhora da Oliveira, Guimarães, Portugal; ^3^BIAL – Portela & Ca S.A., Coronado, Portugal; ^4^BIAL ‐ R&D Investments, S.A. Coronado, Portugal; ^5^Paracelsus‐Elena Klinik, Kassel, Germany

**Background and aims:** Sleep disturbances are common and challenging to manage in Parkinson's disease (PD). By optimizing levodopa, the catechol‐O‐methyl transferase inhibitor opicapone (OPC) may alleviate specific PD‐related sleep issues in patients with motor fluctuations. This study assessed the effect of OPC on different sleep issues in PD patients with sleep disturbances.

**Methods:** The 6‐week, open‐label, single‐arm OpicApone in Sleep dISorder (OASIS) study evaluated the efficacy of OPC 50 mg in treating sleep disturbances as levodopa add‐on therapy. The primary endpoint was changes from baseline to Week 6 in PD Sleep Scale‐2 (PDSS‐2) total score. This post‐hoc analysis evaluated changes in specific PDSS‐2 items and in the number of patients reporting early morning dystonia at Week 6.

**Results:** Of the 16 patients included in the OASIS, 15 completed treatment. At Week 6, patients experienced improvements in several sleep issues as indicated by the mean (standard error) reductions in the scores for poor sleep quality in the previous week (‐1.1 [0.3]; ‐42%), sleep latency (‐0.9 [0.4]; ‐50%), sleep fragmentation (‐1.3 [0.4]; ‐39%), and restorative sleep (‐1 [0.3]; ‐41%) (Figure 1A). Patients also reported significantly less difficulty moving or turning in bed (‐0.9 [0.3]; ‐35%) and significantly less tremor upon waking (‐0.7 [0.3], ‐39%) (Figure 1A). Among patients reporting early morning dystonia at baseline (25%), half transitioned to no dystonia (Figure 1B).

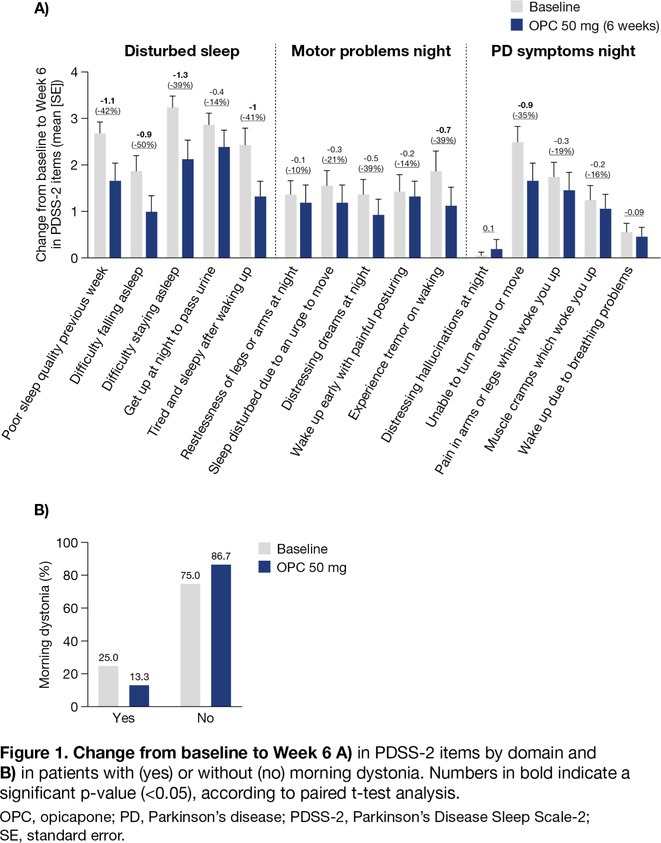

**Conclusion:** OPC adjunct to levodopa therapy improved sleep‐related symptoms by > 30%, including insomnia and restorative sleep and also nighttime and morning motor symptoms, offering dual benefit for PD patients with wearing‐off and sleep‐related disturbances.

**Disclosure:** JJF has received grants from GlaxoSmithKline, Grunenthal, Fundação MSD (Portugal), TEVA, MSD, Allergan, Novartis and Medtronic. JFF also received consultancy and speaker fees, and participated in advisory boards for GlaxoSmithKline, Novartis, TEVA, Lundbeck, Solvay, BIAL, Merck‐Serono, Merz, Ipsen, Biogen, Acadia, Allergan, Abbvie, Sunovion Pharmaceuticals, Zambon, Affiris and Angelini. MFG has received payment/honoraria for lectures from Zambon, Bial Portugal, Takeda and Amicus Therapeutics, and payment/honoraria for advisory boards from Abbvie and Bial Portugal. MMF, RC, HB and JH are employees of Bial. CT has received consulting/independent contractor fees from AbbVie, UCB, Roche, Bial, Ono, Boehringer and Convatec, and speakers honoraria from AbbVie, STADA, Bial and Esteve. CT also receives royalties from Thieme Publisher, License fee: PDSS‐2; and grant and contracted research support from The Michale J. Fox Foundation, EU: Era‐Net program, BRAVA Project; and is an employee (full or part‐time) of Paracelsus‐Elena Hospital, Kassel. Study supported by Bial.

## EPR‐209

### Long‐term effect of opicapone in Parkinson's patients without motor complications: 1.5‐Year EPSILON study findings

#### J. Ferreira ^1^; O. Rascol^2^; F. Stocchi^3^; A. Antonini^4^; G. Castilla‐Fernández^5^; H. Brigas^6^; J. Moreira^6^; J. Rocha^6^; M. Fonseca^5^; J. Holenz^6^; W. Poewe^7^

##### ^1^IMM ‐ Instituto de Medicina Molecular João Lobo Antunes, Faculdade de Medicina, Universidade Lisboa, Lisbon, Portugal; ^2^University of Toulouse, University Hospital of Toulouse, INSERM, Clinical Investigation Center CIC1436 Departments of Neurosciences and Clinical Pharmacology and NS‐Park/FCRIN network, Toulouse, France; ^3^Department of Neurology, IRCCS San Raffaele Pisana, Rome, Italy; ^4^Department of Neurosciences, University of Padova, Padova, Italy; ^5^BIAL R&D Investments, S.A., Portugal; ^6^BIAL – Portela & Ca S.A., Coronado, Portugal; ^7^Department of Neurology, Medical University of Innsbruck, Innsbruck, Austria

**Background and aims:** The EPSILON study assessed the clinical efficacy of adjunctive opicapone (OPC) in levodopa‐treated Parkinson's Disease (PD) patients without motor complications. Long‐term effects of OPC exposure are reported.

**Methods:** EPSILON consisted of a double‐blind (DB) and a 1‐year open‐label‐extension (OLE) phase. In the DB phase, levodopa‐treated PD patients without motor complications received OPC 50 mg or placebo: primary endpoint was change in Movement Disorder Society‐Unified PD Rating Scale‐Part III (MDS‐UPDRS‐III) score from baseline to Week 24. In the OLE, patients completing the DB phase received OPC 50 mg; key endpoint was mean change from OLE baseline to Week 52 in MDS‐UPDRS‐IV scores. Other endpoints included: safety/tolerability, Clinician's and Patient's Global Impression of Improvement (CGI‐I, PGI‐I), non‐motor symptoms and quality of life (QoL).

**Results:** At Week 24, significantly greater improvements in MDS‐UPDRS‐III score and lower proportions of patients with motor complications were reported for OPC versus placebo (Figure 1). At study end, patients treated with OPC in both phases (N = 151) maintained reductions in MDS‐UPDRS‐III scores (mean [standard error] change of ‐7.2 ± 0.8 from DB baseline), with minimal changes in daily levodopa dosage and lower rates of patients with motor complications than those who received placebo in the DB (Figure 1). After 1.5 years, minimal changes in MDS‐UPDRS‐IV scores, improvements on PGI‐C and CGI‐I, and no changes in non‐motor symptoms and QoL were reported (Table 1). Opicapone was well‐tolerated.

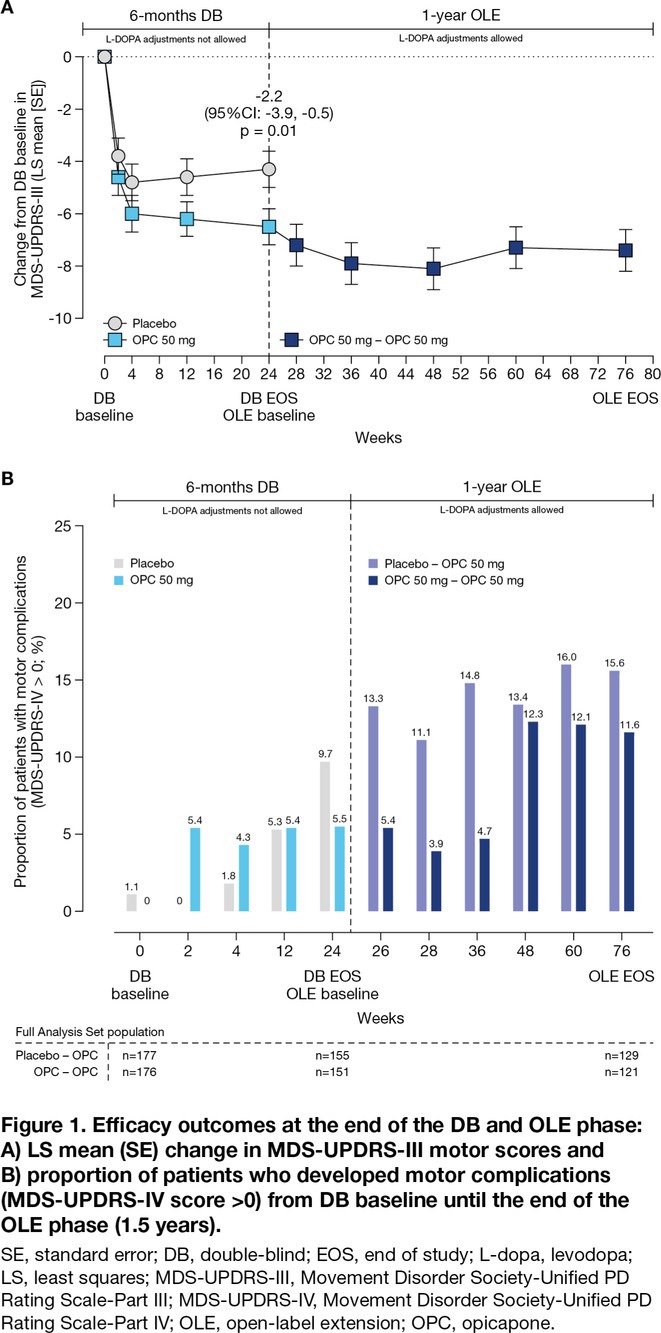

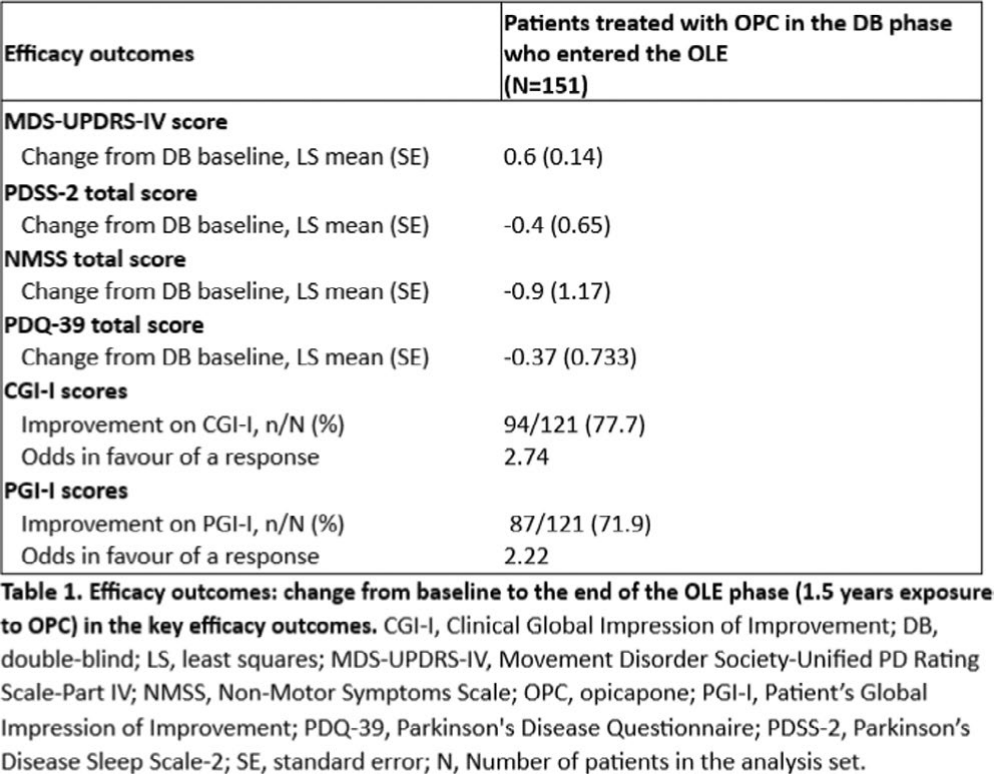

**Conclusion:** OPC provided sustained motor benefits over 1.5 years with few patients developing motor complications, supporting its long‐term use in PD patients without motor fluctuations.

**Disclosure:** JJF received grants/compensation from GlaxoSmithKline, Grunenthal, Fundação MSD, TEVA, MSD, Allergan, Novartis, Medtronic, GlaxoSmithKline, Novartis, TEVA, Lundbeck, Solvay, BIAL, Merck, Merz, Ipsen, Biogen, Acadia, Allergan, Abbvie, Sunovion Pharma, Zambon, Affiris and Angelini. OR has received compensation from AbbVie, Adamas, Acorda, Addex, AlzProtect, ApoPharma, AstraZeneca, Axovant, BIAL, Biogen, Britannia, Buckwang, CereSpir, Clevexel, Denali, INC Research, IPMDS, Lundbeck, Lupin, Merck, MundiPharma, NeurATRIS, NeuroDerm, Novartis, ONO Pharma, Osmotica, Parexel, Pfizer, Prexton Therapeutics, Quintiles, Roche, Sanofi, Servier, Sunovion, Theranexus, Takeda, Teva, UCB, Vectura, Watermark Research, XenoPort and Zambon; grants from CHU, France‐Parkinson, INSERM, Michael J. Fox Foundation and Cure Parkinson UK. FS received honoraria from Lundbeck, UCB, Chiesi, Zambon, Britannia, Cynapsus, Sunovion, Kyowa, Abbvie, Neuroderm, Biogen and BIAL. AA received compensation/support from UCB, Boehringer Ingelheim, Britannia, AbbVie, Zambon, BIAL, NeuroDerm, Theravance Biopharma and Roche, Chiesi Pharma, Lundbeck and Horizon 2020. WP received fees/honoraria from Alterity, AbbVie, Affiris, AstraZeneca, Axovant, BIAL, Biogen, Britannia, Lilly, Lundbeck, NeuroDerm, Neurocrine, Denali Pharma, Orion Pharma, Roche, Stada, Sunovion, Takeda, UCB and Zambon; grant from Michael J. Fox Foundation, FP7 and Horizon 2020. GCF, HB, JM, FR, MMF and JH are employees of Bial. Study supported by Bial.

## EPR‐210

### Phenotypic and genotypic spectrum of late‐onset dystonia

#### L. Kunc ^1^; P. Havránková^1^; J. Roth^1^; J. Rajmonová^1^; J. Necpál^2^; M. Škorvánek^3^; T. Serranová^1^; O. Ulmanová^1^; M. Baláž^4^; E. Tsoma^5^; M. Zech^6^; R. Jech^1^

##### ^1^Department of Neurology and Center of Clinical Neuroscience, First Faculty of Medicine, Charles University and General University Hospital in Prague, Czechia; ^2^Department of Neurology, Zvolen Hospital, Slovak Republic; ^3^Department of Neurology, Faculty of Medicine, Safarik University and University Hospital of L. Pasteur, Kosice, Slovak Republic; ^4^First Department of Neurology, Medical Faculty of Masaryk University, Movement Disorders Center, Masaryk University School of Medicine, St. Anne's Hospital, Brno, Czechia ^5^Regional Clinical Center of Neurosurgery and Neurology, Department of Family Medicine and Outpatient Care, Uzhhorod National University, Uzhhorod, Ukraine; ^6^Institute of Neurogenomics, Helmholtz Zentrum München, Munich, Germany

**Background and aims:** Dystonia is a dyskinetic movement disorder with significant phenotypic and genotypic variability. Recent advances in genetic testing have shown that genetically determined dystonic syndromes are typically associated with early onset, generalized forms, and/or complex neurological symptoms. Although genetically determined late‐onset dystonia (LOD) has also been described, its genotypic and phenotypic characteristics remain systematically unexplored. This study aimed to analyze the phenotypic and genotypic spectrum of LOD (onset > = 21 years).

**Methods:** All 728 patients with dystonia included in a whole‐exome sequencing project were categorized by age of onset, phenotype, and genotype, that is, pathogenic or likely pathogenic variants, variants of uncertain significance (VUS), and no identified mutations.

**Results:** A total of 160 patients with LOD were identified. Pathogenic or likely pathogenic variants were detected in 21 patients, and VUS in 13, together representing 21.2% of identified variants. Complex neurological symptoms were observed in 71% of patients with pathogenic or likely pathogenic mutations, compared to 17% in the mutation‐negative group. Among those with complex symptoms and pathogenic or likely pathogenic mutations, cerebellar phenotype occurred in 67%, compared to 32% in the mutation‐negative group. Similarly, cognitive impairment was observed in 60% of patients with pathogenic or likely pathogenic mutations versus 36% in the mutation‐negative group.

**Conclusion:** In conclusion, our findings suggest that patients with LOD, particularly those with complex dystonia involving cognitive and/or cerebellar impairments, may have a substantial genetic background. These symptoms could serve as markers, increasing the likelihood of mutation identification and highlighting the need for a tailored approach to genetic testing.

**Disclosure:** The study was supported by the grant: AZV Czech Republic: NW24‐04‐00067 and EU program EXCELES: LX22NPO5107.

## EPR‐211

### Outcomes of MVD surgery in hemifacial spasm: A single‐center experience

#### M. Segura‐Lozano ^1^; C. Castillo‐Rangel^2^; O. Carranza‐Rentería^1^; A. Munguía Rodríguez^1^

##### ^1^Neurología Segura. Hospital Angeles Morelia, Morelia, México; ^2^Neurocirugía, Hospital 1° de Octubre, ISSSTE, Ciudad de México, México

**Background and aims:** Hemifacial spasm (HFS) is characterized by involuntary tonic and clonic contractions of the facial muscles, commonly caused by vascular compression at the root entry/exit zone of the facial nerve (CN VII). This study aimed to evaluate the clinical outcomes of microvascular decompression (MVD) for HFS in our patient series.

**Methods:** We conducted a retrospective analysis of 58 patients who underwent MVD for HFS at our institution between January 2014 and December 2023. Data collected included demographics, symptom duration, intraoperative findings, and postoperative outcomes. Follow‐up ranged from 12 months to 10 years.

**Results:** Among 58 patients, 28 (48.3%) were women (mean age: 47.8 years) and 30 (51.7%) were men (mean age: 46.6 years). Immediate postoperative outcomes were excellent or good in 93.1% of cases. Long‐term success was achieved in 84.5%, with a mean follow‐up of 4.1 years. Recurrence occurred in 10.3% at a mean of 8.5 months post‐surgery. Transient complications included facial paralysis (37.9%) and hearing loss (27.6%), while permanent complications included hearing loss (13.8%) and facial paralysis (5.2%). No mortality was reported. In 98.3% of cases, the vascular culprit was identified, with the most common offender being the anterior inferior cerebellar artery (48.3%).

**Conclusion:** MVD is highly effective for the treatment of HFS, with excellent immediate and long‐term outcomes. Early surgical intervention is recommended to enhance prognosis, as delayed treatment may increase the risk of complications and recurrence.

**Disclosure:** Nothing to disclose.

## Monday, June 23 2025

## Clinical neurophysiology

## EPR‐212

### Temporal interference stimulation of the hippocampus suppresses epileptic biomarkers in patients with epilepsy

#### A. Williamson; M. Brázdil; F. Missey; J. Trajlinek; O. Studnička; M. de Araújo e Silva; V. Všianský; I. Dolezalova; M. Pail

##### St. Anne's University Hospital, Brno, Czechia

**Background and aims:** Medication‐refractory focal epilepsy presents a significant clinical challenge, with approximately 30% of patients ineligible for surgery due to the involvement of eloquent cortex in the epileptogenic network. For such patients with limited surgical options, electrical neuromodulation represents a promising alternative therapy. In this study, we assess the potential of non‐invasive temporal interference (TI) electrical stimulation to reduce epileptic biomarkers in patients with epilepsy by comparing intracerebral recordings obtained before, during, and after TI stimulation to those recorded during low and high kHz frequency (HF) sham stimulation (ClinicalTrials.gov: NCT06716866).

**Figure:**
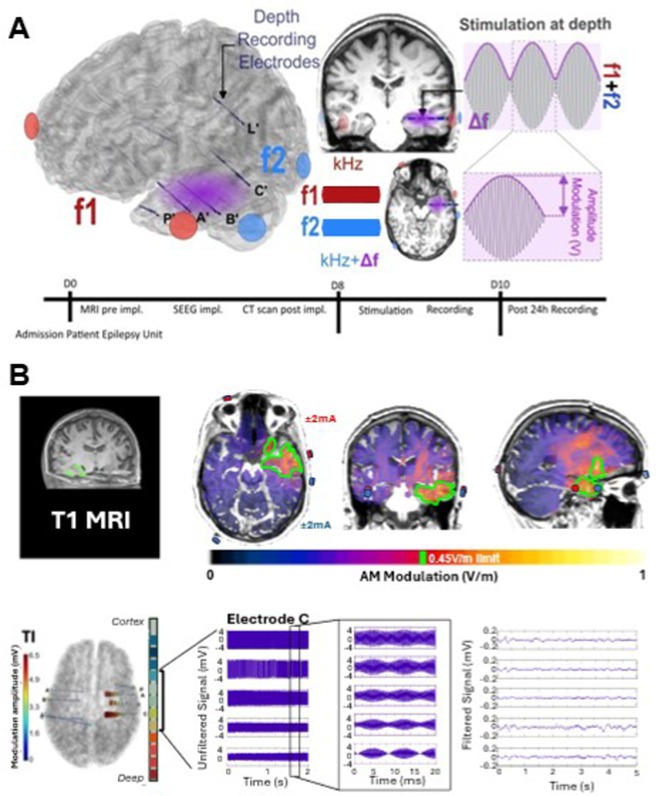
**FIGURE 1** Temporal interference protocol in sEEG‐implanted patients with epilepsy.

**Methods:** Thirteen patients diagnosed with mesiotemporal epilepsy (MTLE) and implanted with stereoelectroencephalography (sEEG) depth electrodes received TI stimulation with an amplitude modulation (AM) frequency of 130Hz (Δf). AM was applied using low‐frequency carriers (1kHz + 1.13kHz) or high‐frequency carriers (9kHz + 9.13kHz), targeting the hippocampus. Epileptic biomarkers, including interictal epileptiform discharges (IEDs) and pathological high‐frequency oscillations (HFOs), were evaluated across conditions.

**Results:** TI stimulation significantly reduced IEDs and fast‐ripple (FR) HFOs in the hippocampal focus, with suppression propagating brain‐wide. HF sham stimulation at 1kHz impacted IEDs in the cortex but failed to reach the hippocampus, and its effects diminished with increasing frequency. Unlike HF sham, TI demonstrated a frequency‐independent effect and exhibited a strong carry‐over suppression post‐stimulation.

**Figure:**
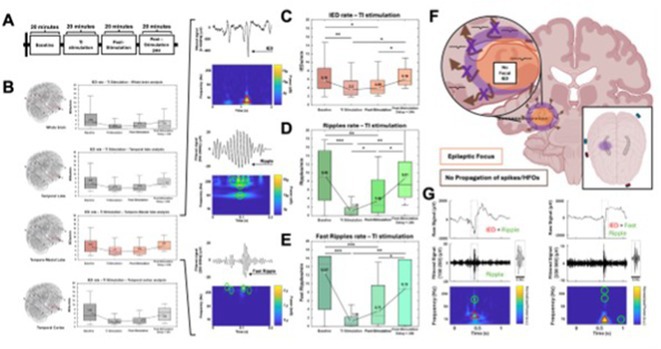
**FIGURE 2** Epileptic biomarkers are suppressed during TI stimulation, and a post‐stimulation carrier‐over effect is observed.

**Conclusion:** These findings support TI as a promising non‐invasive neuromodulation approach for epilepsy, providing a potential pre‐screening tool before deep brain stimulation (DBS) or responsive neurostimulation (RNS). TI's distinct biophysical properties highlight its advantages over HF conduction block.

**Disclosure:** Nothing to disclose.

## EPR‐213

### Prediction of abnormal responses to single‐pulse electrical stimulation in drug‐resistant epilepsy using adaptive AI

#### S. Shirani^1^; B. Abdi‐Sargezeh^2^; I. Stavropoulos ^1^; G. Alarcon^4^; A. Valentin^1^

##### ^1^Institute of Psychiatry, Psychology & Neuroscience, Department of Basic & Clinical Neuroscience, King's College London, UK; ^2^Nuffield Department of Clinical Neurosciences, University of Oxford, Oxford, UK; ^4^Department of Clinical Neurophysiology, Royal Manchester Children's Hospital, Manchester, UK

**Background and aims:** Delayed responses (DRs) to Single pulse electrical stimulation (SPES) during intracranial EEG (iEEG) recordings observed 100ms‐1s after the stimulation, are associated with epileptogenicity and are useful for identifying the seizure onset zone (SOZ). We employed discriminative underlying features of iEEG signals in a custom adaptive attention‐based deep neural network to predict the DRs to SPES.

**Methods:** We analyzed iEEG data from 60 patients. DRs were annotated and the iEEG was fed to a peak‐detection algorithm to identify SPES artifact and select 1.5‐second segments prior to this. The segments were labeled as normal (no visible DRs) and abnormal (with visible DRs) and fed to a features‐extraction algorithm. A sequential floating forward selection (SFFS) algorithm was used to identify the most discriminative subset of features between normal and abnormal channels for each case in an iterative fashion. We input these features to a custom adaptive deep‐neural network pipeline that employs the attention‐layer mechanism in its architecture to prioritize the features based on importance.

**Figure:**
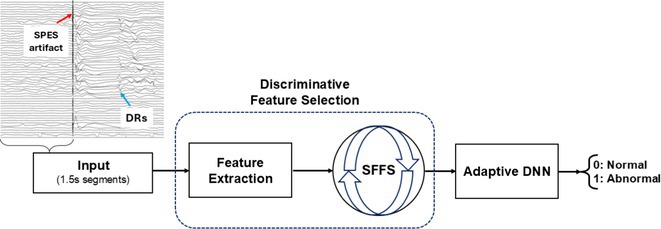
**FIGURE 1** The overall pipeline used in this study for prediction of SPES DRs. The red arrow points to the SPES artifact and the blue array shows examples of annotated visible DRs.

**Results:** The most discriminative subset of features identified by the SFFS algorithm were spectral centroid, wavelet entropy, peak frequency, spectral flatness, and beta band power. The adaptive attention‐based deep neural network achieved an area under the ROC curve (AUC) of 0.87, indicating strong predictive capability. Sensitivity and specificity were above 80%.

**Conclusion:** The findings demonstrate a direct relationship between pre‐stimulation iEEG patterns and the SPES response, suggesting that pre‐stimulation iEEG features can serve as predictive factors for SPES DRs, and abnormal areas can be better identified by analyzing the background activity.

**Disclosure:** Part of this work was supported by a European Union's Horizon 2020 grant, the Multidisciplinary Expert System for the Assessment and Management of Complex Brain Disorders (MES‐CoBraD) [grant agreement No 965422].

## EPR‐214

### Decremental response in patients with amyotrophic lateral sclerosis

#### J. Zhang

##### Beijing 301 Hospital, China

**Background and aims:** Neuromuscular junction (NMJ) denervation plays a critical role in amyotrophic lateral sclerosis (ALS). Lately, repetitive nerve stimulation (RNS) has been applied to assess the efficacy of drugs targeting the NMJ. However, the sensitivity and stability of parameters during RNS, and factors contributing to the decremental response, have yet to be fully elucidated.

**Methods:** A total of 626 patients who were diagnosed with ALS and underwent 3Hz RNS test from June 2016 to August 2023 were enrolled. Data on their clinical, biochemical and electrophysiological indicators were divided into a training set and a test set. Stepwise regression was used in model building.

**Results:** Forty‐two percent of patients had a decrement larger than 10% and 24% had a decrement larger than 15%. Area decrement had a higher rate of abnormal result and a lower coefficient of variation than amplitude decrement. No significant difference in the rate of abnormal decrement was found when the first compound muscle action potential was compared with either the fourth or fifth one. High‐density lipoprotein cholesterol, serum uric acid, forced vital capacity, onset site, sex, and motor unit potential duration were independent factors contributing to the decremental response.

**Conclusion:** In patients with ALS, NMJ safety factor is reduced during re‐innervation. During RNS test, assessing area decrement significantly enhances our ability to detect the impairment of neuromuscular transmission in patients with ALS. Independent factors contributing to decremental response need to be considered in clinical trials targeting NMJ in patients with ALS.

**Disclosure:** Nothing to disclose.

## EPR‐215

### Unraveling Guillain‐Barré Syndrome: Predictors, patterns and outcomes from a decade of care

#### M. Pereira ^1^; C. Guedes Vaz^1^; D. Pereira^1^; M. Cardoso^2^; A. Sousa^2^

##### ^1^Neurology Department, ULSSA, Porto, Portugal; ^2^Neurophysiology Department, ULSSA, Porto, Portugal

**Background and aims:** We aimed to analyze Guillain‐Barré Syndrome (GBS) patients over the last 10 years, correlating clinical, analytical, and electrophysiological findings with outcomes.

**Methods:** Retrospective analysis of clinical and additional diagnostic data of all patients admitted to a tertiary center between January 2013‐December 2023.

**Results:** Ninety‐nine patients (52.5% women), with a mean age at presentation of 45.5 ± 26.3 years, were included. Fifty‐nine (59.6%) presented with the acute inflammatory demyelinating polyneuropathy subtype, with a median of 4 days (IQR 5) to first evaluation. Among 27 pediatric patients, limb muscle pain was more common at presentation compared to adults (37%, *p* < 0.001). Upper airway infections(44.4%) was the most frequent trigger, followed by gastroenteritis (19.2%), which was associated with the acute motor axonal neuropathy subtype (52.9%,*p* = 0.013). Decreased motor amplitude on electromyography (EMG) correlated with higher disability at discharge (median modified Ranking score (mRS)≥3, IQR 2, *p* = 0.001), though not at 1‐year. Spontaneous activity on EMG (27.8%,*p* 0.048), cranial nerve involvement (27%, *p* < 0.001), and respiratory failure (41.2%, *p* < 0.001) were linked to need of rescue treatment. A GBS disability score≥4 was associated with a lower MRC sum score (28 vs. 63, *p* < 0.001) and invasive ventilation (30.8%, *p* < 0.001). Hyponatremia at admission predicted worse 1‐year outcomes (mRS≥3, 40%, *p* = 0.004).

**Conclusion:** EMG findings (decreased motor amplitude and spontaneous activity) and hyponatremia at admission were predictors of higher initial disability and worse long‐term outcomes, respectively. Despite the severity of cases requiring ventilation, long‐term outcomes were unaffected. This study highlights key features of GBS in a diverse cohort and the need of tailored management of risk factors.

**Disclosure:** Nothing to disclose.

## EPR‐216

### Personalized acoustic‐based music neuromodulation in epilepsy

#### O. Strýček ^1^; J. Mekyska^3^; Š. Miklánek^3^; M. Fusek^4^; K. Štillová^1^; M. Mazánek^5^; I. Rektor^2^

##### ^1^Brno Epilepsy Centre, First Department of Neurology, St. Anne's University Hospital and Faculty of Medicine, Masaryk University, Member of ERN‐EpiCARE, Brno, Czechia. asaryk University, Brno, Czechia; ^2^Central European Institute of Technology (CEITEC), Masaryk University, Brno, Czechia; ^3^Department of Telecommunications, Faculty of Electrical Engineering and Communication, Brno University of Technology, Brno, Czechia; ^4^Department of Mathematics, Faculty of Electrical Engineering and Communication, Brno University of Technology, Brno, Czechia; ^5^Masaryk University Symphony Orchestra, Department of Musicology, Faculty of Arts, Masaryk University, Brno, Czechia

**Background and aims:** Music‐based neuromodulation has emerged as a promising therapeutic approach for drug‐resistant epilepsy. This study builds on previous research by investigating how distinct musical features affect interictal epileptiform discharges (IEDs) in intracerebral EEG (iEEG).

**Methods:** Twenty‐five patients with drug‐resistant epilepsy undergoing presurgical iEEG evaluation participated in a two‐day study. Patients listened to a variety of musical compositions with defined acoustic properties. EEG recordings were taken before and after each listening session to assess changes in IEDs.

**Results:** The study revealed individualized patterns of IED reduction, with certain acoustic properties showing consistent effects across musical genres. Mozart's Piano Concerto No. 27 (K 595c) reduced IEDs by 28% during music listening (*p* = 0.0191) and by 19% in the post‐music resting state (*p* = 0.0111). In contrast, relaxation music increased IEDs by 55% (*p* = 0.0197). Individualized acoustic analyses identified compositions that significantly reduced IEDs, with reductions ranging from 32% to 44% (*p* = 0.0001). Compositions with contrasting acoustic properties did not show significant effects, highlighting the influence of acoustic features rather than musical genre.

**Conclusion:** Specific acoustic properties can reproducibly modulate brain activity at the individual level, reducing IEDs based on personalized testing and selection across musical genres. These findings support the potential of music‐based neuromodulation as a tailored therapeutic approach for epilepsy management. Further research is needed to explore individual variability in music‐based interventions for epilepsy treatment.

**Disclosure:** Nothing to disclose.

## EPR‐217

### Synkineses as a marker for neuroplasticity in nerve transfer surgeries

#### Š. Brušáková ^1^; I. Humhej^2^; J. Ceé^1^; I. Holečková^3^

##### ^1^1Department of Neurology, Masaryk Hospital Krajská Zdravotní a.s., Sociální Péče 3316/12A, Ústí nad Labem, Czechia, ^2^2Neurosurgical Department, Faculty of Health Studies J. E. Purkynje University, Masaryk Hospital Krajská Zdravotní a.s., Sociální Péče 3316/12A, Ústí nad Labem, Czechia, ^3^3Neurosurgical Department, Faculty of Medicine in Pilsen, Charles University, Husova 3, Pilsen, Czechia

**Background and aims:** Synkineses occure after neurotization operations on peripheral nerves, when an anatomically close nerve or its fascicle is used to reconstruct an destroyed, unreconstructible nerve (impossibility of suture or grafts use). This study explores its clinical implications in patients with brachial plexus injuries treated with nerve transfers.

**Methods:** A total of 34 nerve transfers in 21 patients were evaluated at a minimum of three years post‐surgery. Synkineses were verified by a two‐channel needle EMG in the muscle originally innervated by the donor nerve and in the target muscle (recipient). We monitor the voluntary activity in both muscles during the voluntary contraction of the original and target muscles. The follow‐up period is set to 3 years.

**Results:** Synkineses were observed in 70.6% (24/34) of cases. Patients without synkineses achieved significantly higher voluntary activation scores (*p* < 0.05). Early reinnervation correlated positively with reduced synkineses and improved outcomes (Rs = 0.528). Time from injury to surgery inversely impacted neuroplastic adaptation (Rs = ‐0.500), highlighting the importance of early intervention.

**Conclusion:** Synkinesis reflects both adaptive and maladaptive neuroplasticity in nerve transfer patients. Monitoring the dynamics of synkinesis donor ‐ recipient serves as an indirect evidence of reorganization of the motor cortex. Future research should focus on optimizing protocols to leverage neuroplasticity while minimizing synkinetic interference.

**Disclosure:** Nothing to disclose.

## EPR‐218

### Treatment of the spastic hemiparesis with selective dorsal rhizotomy in adults

#### Y. Rushkevich; G. Zabrodzets; E. Vasilevich; M. Talabaev; Y. Piashko; K. Malhina

##### Republican Research and Clinical Center of Neurology and Neurosurgery, Minsk, Belarus

**Background and aims:** Spasticity is a common cause of disability. Selective dorsal rhizotomy (SDR) successfully reduces lower limb spasticity (LLS) in children but its efficacy in adult spasticity is being investigated. Aim: evaluate the efficacy of SDR for LLS in adults with spastic hemiparesis (SH).

**Methods:** Five male patients with SH were included (aged 18‐45 years): 2 ‐ traumatic brain injury, 1 had cerebral hemorrhage, 2 had cerebral palsy. The GMFCS, modified Ashworth score (MAS), EMG, range of motion (ROM) of the involved joints were controlled. In all patients single‐level SDR with intraoperative triggered EMG were done.

**Results:** Preoperatively, 4 patients had GMFCS II, one had I. The target of SDR was the key muscles (KM) with MAS 2‐3. Intraoperative ipsilateral L3‐S1 sensory nerve roots were divided into 3‐5 portions that were EMG tested. Portions with pathological KM pattern were dissected up to 75%. The spastic EMG pattern was different from the normal KM innervation. One week after surgery, EMG analysis showed that KM spasticity was significantly reduced. The H‐reflex, foot clonus completely disappeared in all patients. 3 patients with GMFCS II improved to I. There were statistically significant differences in the KM MAS, the ROM of the ankle and knee, 25‐Foot Walk Test between preoperatively and postoperatively (*p* < 0.05). All patients had ipsilateral L5‐S1 hypesthesia which significantly decreased after 1 month. No urinary/bowel dysfunction was observed. After a month, all patients considered that they had benefited from SDR.

**Conclusion:** SDR is effective in SH for adults. Intraoperative EMG monitoring is necessary for the SDR.

**Disclosure:** Nothing to disclose.

## Epilepsy

## EPR‐219

### Genetic variability in inflammatory genes and susceptibility to mesial temporal lobe epilepsy and drug resistance

#### B. Guerra Leal ^1^; M. Costa^2^; C. Carvalho^2^; S. Brás^2^; R. Samões^3^; C. Lemos^5^; C. Teixeira^4^; J. Freitas^4^; P. Pinho e Costa^1^; J. Chaves^3^

##### ^1^Unit for Multidisciplinary Research in Biomedicine (UMIB, ICBAS‐UPorto); Immunogenetics Laboratory, ICBAS‐UPorto; Laboratory for Integrative and Translational Research in Population Health (ITR), Porto, Portugal; ^2^Immunogenetics Laboratory, Department of Molecular Pathology and Immunology, Instituto de Ciências Biomédicas Abel Salazar Universidade do Porto (ICBAS‐UPorto); ^3^Unit for Multidisciplinary Research in Biomedicine (UMIB, ICBAS‐UPorto); Hospital de Santo António ‐ Unidade Local de Saúde de Santo António, Porto, Portugal; ^4^Hospital de Santo António ‐ Unidade Local de Saúde de Santo António, Porto, Portugal; ^5^Unit for Multidisciplinary Research in Biomedicine (UMIB, ICBAS‐UPorto), Porto, Portugal

**Background and aims:** Genetic Variability in Inflammatory Genes and Susceptibility to Mesial Temporal Lobe Epilepsy and Drug Resistance

**Methods:** A total of 218 MTLE‐HS patients (101 males, 116 with febrile seizure antecedents, and 173 with drug‐resistant epilepsy [DRE]) and 177 healthy controls (82 males) were genotyped for the selected SNPs using TaqMan Real‐Time PCR.

**Results:** The rs4612666 TT genotype, which enhances NLRP3 activity, was more frequent in MTLE‐HS patients compared to controls, but this difference was not statistically significant (6.7% vs. 3.4%; OR [95% CI] = 2.18 [0.79–6.04]; *p* = 0.13). The rs3751143 AC genotype was more frequent in DRE patients compared to non‐DRE patients (33.8% vs. 16.2%; OR [95% CI] = 2.56 [0.99–6.62]; *p* = 0.05). No other significant associations were observed.

**Conclusion:** The rs3751143 polymorphism impairs the function of the purinergic receptor P2X7, affecting the inflammatory response and seizure‐induced damage repair. This could potentially contribute to a poor anti‐seizure treatment response. Although no direct association between the studied polymorphisms and MTLE‐HS susceptibility was found, our results highlight the possible role of inflammatory genes, especially P2X7R, in the clinical presentation of MTLE‐HS, emphasizing the need for further research.

**Disclosure:** Work partially funded by a Tecnifar Grant.

## EPR‐220

### Effects of NMDAR and LGI1 antibodies on absence seizures: Insights from genetic and pentylenetetrazol‐induced models

#### N. Çarçak Yılmaz^1^; E. Tuzun ^2^; Ş. Akat^1^; C. Küçükali^2^; F. Onat^3^

##### ^1^Department of Pharmacology, Faculty of Pharmacy, Istanbul University, Istanbul, Turkey; ^2^Department of Neuroscience, Aziz Sancar Institute of Experimental Medicine, Institute of Health Sciences, Istanbul University, Istanbul, Turkey; ^3^Department of Medical Pharmacology, Acibadem Mehmet Ali Aydinlar University Faculty of Medicine, Istanbul, Turkey

**Background and aims:** Leucine‐rich glioma‐inactivated protein 1 (LGI1) and N‐methyl‐D‐aspartate receptor (NMDAR) are key proteins involved in regulating neuronal excitability within the central nervous system. In conditions like anti‐LGI1 encephalitis and anti‐NMDAR encephalitis, autoantibodies target and disrupt these proteins, leading to epileptic seizures. However, the roles of LGI1 and NMDAR dysfunction in the pathophysiology of absence seizures remain unclear.

**Methods:** IgG purified from the peripheral blood of patients with anti‐NMDAR (*n* = 3) encephalitis, anti‐LGI1 encephalitis (*n* = 4) and healthy controls (HC) (*n* = 4) was administered chronically every other day for 11 days via stereotaxic injection into the lateral ventricle of genetic absence epilepsy rat model (GAERS) and Wistar rats. Before and after antibody administration, electroencephalography (EEG) recordings were taken for 2 hours to analyze the duration and number of spontaneous spike‐and‐wave discharges (SWDs) in GAERS rats and 120 minutes after 35 mg/kg pentylenetetrazol (PTZ)‐administration in Wistar rats.

**Results:** In GAERS rats, NMDAR IgG infusion significantly increased the duration and number of SWDs compared to the healthy control group, highlighting its acute effect on SWD activity. In contrast, LGI‐1 IgG did not cause significant changes in SWD parameters. Similarly, NMDAR‐ but not LGI1‐IgG‐infused Wistar rats exhibited increased susceptibility to PTZ‐induced absence seizures compared to healthy control group.

**Conclusion:** Our findings demonstrate differential roles of NMDAR and LGI‐1 antibodies in modulating absence seizure activity. NMDAR IgG infusion significantly enhances SWD duration and number in GAERS rats and PTZ‐induced absence seizures in Wistar rats, highlighting the role of NMDAR in absence seizure induction.

**Disclosure:** Nothing to disclose.

## EPR‐221

### Describing patients with prolonged seizures: European subgroup results from a global real‐world point‐in‐time study

#### E. Trinka ^1^; M. Walker^2^; R. Kalviainen^3^; S. Haut^4^; J. Stern^5^; L. Hirsch^6^; A. Gillespie^7^; L. LeBrocq^7^; E. Smith^7^; C. Laloyaux^8^; O. Radunz^8^; J. Wilson^8^

##### ^1^Department of Neurology, Neurocritical Care, and Neurorehabilitation, Member of European Reference Network EpiCARE, Center for Cognitive Neuroscience, Christian Doppler University Hospital, Paracelsus Medical University, Salzburg, Austria; ^2^UCL Queen Square Institute of Neurology, Department of Clinical & Experimental Epilepsy, QCL, UK; ^3^Department of Neurology, School of Medicine, University of Eastern Finland and Kuopio Epilepsy Center, Kuopio, Finland; ^4^Montefiore‐Einstein Comprehensive Epilepsy Center, New York, USA; ^5^Department of Neurology, University of California, Los Angeles, USA; ^6^Comprehensive Epilepsy Center, Department of Neurology, Yale University, New Haven, USA; ^7^Adelphi Real World, Bollington, UK; ^8^UCB, Brussels, Belgium

**Background and aims:** People with epilepsy (PwE) can experience prolonged seizures (PS), which may progress to Status epilepticus (SE; seizure(s) lasting ≥5min). However, the definition, prevalence, and patient population of PS are not well characterized.

**Methods:** European data were drawn from Adelphi PS Disease Specific Programme™ (real‐world, point‐in‐time survey) conducted in France/Germany/Italy/Spain/United Kingdom/United States/Japan/China, from March/2023‐February/2024. Neurologists/epileptologists/internal medicine specialists (IMs) completed record forms for PwE on stable antiseizure medication (including rescue medications [RM]) regimen who had experienced ≥1 PS (≥2min and/or longer than “normal”/“non‐PS”) in prior 12‐months. Outcomes of PS and non‐PS are presented.

**Results:** 132/28/9 neurologists/epileptologists/IMs completed records for 1,411 PwE experiencing PS. Median [Q1‐Q3] patient age 34.0 [22.0‐50.0] years, 58% male, 41% required caregiver(s). 49% of sample had had SE, 27% seizure clusters (SC). During prior 12‐months, proportion of sample experiencing events (related to PS vs. non‐PS): SE (34%), SC (PS: 15%; non‐PS: 11%), aura (PS: 45%; non‐PS 38%), injuries (PS: 27%; non‐PS 23%), required emergency services (PS: 27%; non‐PS: 22%) – necessitating emergency‐room (ER) admission (PS: 78%; non‐PS: 41%), intensive‐care unit (ICU) (PS: 14%; non‐PS: 5%) – and hospitalization (PS: 25%; non‐PS: 14%). 64% of sample were prescribed RM (often including oral benzodiazepines), 57% had seizure action plans. PwE self‐reported seizure worry via 0‐10 scale where 10 = “worry all the time”: PS (median [Q1‐Q3]) 5.0/10 [2.0‐7.0]; non‐PS 4.0/10 [1.3‐6.0].

**Conclusion:** PwE experiencing PS regularly encounter progression to SE and/or SC, leading to emergency care, and hospital/ER/ICU admissions, despite best practice. Rapid and early seizure termination is essential to avoid harmful outcomes. UCB‐sponsored

**Disclosure:** RK reports personal fees from Angelini Pharma, Lundbeck, Marinus, Orion Pharma, Eisai, UCB, OmaMedical, Takeda, Jazz Pharmaceuticals; her institution received grants from European Union, Academy of Finland, Finnish Government Research Funding, Saastamoinen Foundation, Vaajasalo Foundation, Jane and Aatos Erkko Foundation. ET has received personal fees from Arvelle Therapeutics, Inc., Argenx, Alexion, Bial, Biogen, Biocodex, Böhringer Ingelheim, Eisai, Epilog, Everpharma, GlaxoSmithKline, GW Pharma, Jazz Pharmaceuticals, LivaNova PLC, Marinus Pharmaceuticals, Inc., Medtronic, NewBridge Pharmaceuticals, Novartis, Sandoz, Sanofi, Sunovion Pharmaceuticals, Inc., Takeda, UCB, Xenon; grants from Austrian Science Fund (FWF), Bayer, Biogen, Eisai, European Union, GlaxoSmithKline, Novartis, Österreichische Nationalbank, Red Bull, UCB; he is CEO of NeuroConsult GmbH. MCW has received honoraria/consulting fees from Bioquest, Eisai, UCB, Angelini, Seer, EpilepsyGtx; has shares in EpilpesyGtx, an epilepsy gene therapy company. SH is a consultant for UCB, Neurelis, Ventus; and a DSMB member for AbbVie. JS is an advisor for Ceribell, Jazz, Neurelis, SK Life Science, UCB, Xenon; holds stocks in Ceribell; is a speaker for Ceribell, Jazz, LivaNova, Neurelis, SK life Science, UCB; has royalties for Cambridge University Press, MedScape, Springer, Wolters Kluwer. LH declares consultation fees for advising from Accure, Ceribell, Gilead, Marinus, Natus, Neurelis, Neuropace, Rapport Therapeutics, UCB; royalties from Wolters‐Kluwer for authoring chapters for UpToDate‐Neurology, from Wiley for co‐authoring the book “Atlas of EEG in Critical Care”, 1st/2nd editions; honoraria for speaking from Neuropace, Natus, UCB. AG, LL, and ES are employees of Adelphi Real World. UCB contracted Adelphi Real World to perform this secondary analysis. CL, OR, and JCW are salaried employees of UCB and receive stocks or stock options from their employment.

## EPR‐222

### First in human study in drug resistant epilepsy: Safety and performance of a novel optoelectronic vagus nerve stimulator

#### D. El Tahry^1^; D. Mertens^2^; D. Hogeveen^2^; D. Rooijakkers^1^; D. Dewaele^2^; D. Ferrao Santos^1^; S. Gadeyne^2^; I. Cakiroglu^1^; D. Schulze‐Bonhage^3^; D. Coenen^3^; C. Léonard^4^; D. Levy^5^; D. Vonck ^2^

##### ^1^Neurologie, Centre de référence pour l'épilepsie réfractaire Cliniques Universitaires St.‐Luc Av. Hippocrate 10, Brussels Belgium; ^2^Department of Neurology UZ GENT (Universitair Ziekenhuis Gent), Gent Belgium; ^3^Freiburg Epilepsy Center Universitätsklinikum Freiburg, Germany; ^4^Synergia Medical, Mont‐saint‐guibert, Belgium; ^5^Marcus Neuroscience Institute, Boca Raton, Florida, USA

**Background and aims:** Drug‐resistant epilepsy (DRE) affects nearly 30% of patients. For those ineligible for resective surgery, Vagus Nerve Stimulation (VNS) is an alternative treatment. However, current VNS systems face limitations, particularly regarding MRI safety and device longevity. A novel optoelectronic VNS system was developed to address these challenges, providing unconditional MRI access and extended device lifespan. This first‐in‐human study evaluates its safety, performance, and usability in DRE patients.

**Methods:** Patients were recruited from three clinical sites: Cliniques Universitaires Saint‐Luc (CUSL), Ghent University Hospital (UZ Gent), and Universitätsklinikum Freiburg. Eligible participants were adults with DRE and candidates for VNS therapy. The primary endpoint assessed procedure‐ and device‐related adverse events within three months. Secondary endpoints included long‐term safety, seizure outcomes, quality of life, and mood over 24 months.

**Results:** The first five patients were successfully implanted at CUSL and UZ Gent. Postoperative recovery was smooth, and stimulation therapy began 2 weeks post‐implantation. Over the next 10 weeks, the step by step increase of the stimulation delivered was well tolerated. Laryngeal Muscle‐Evoked Potential (LMEP) were successfully recorded in all patients, providing indirect confirmation of vagus nerve activation. By 3 months, the primary safety endpoint was met, with no serious adverse events and other safety events within expected VNS response ranges. No failures were reported with the implanted device, as confirmed by device self‐checks. Initial therapy efficacy was observed.

**Conclusion:** Initial results demonstrate the system's safety and reliability, and the potential for the optoelectronic VNS system to transform the treatment landscape for DRE.

**Disclosure:** Study is supported by Synergia Medical.

## EPR‐223

### Graph neural networks for enhanced epileptogenic zone localization in epilepsy surgery: An innovative method

#### M. Pail ^1^; P. Nejedly^2^; V. Hrtonova^2^; J. Cimbalnik^3^; P. Daniel^1^; V. Travnicek^2^; I. Dolezalova^1^; P. Jurak^2^; P. Klimes^2^; M. Brazdil^1^

##### ^1^Brno Epilepsy Center, Department of Neurology, Member of ERN‐EpiCARE, St. Anne's University Hospital, Faculty of Medicine, Masaryk University, Brno, Czechia; ^2^Institute of Scientific Instruments, The Czech Academy of Sciences, Brno, Czechia; ^3^International Clinical Research Center, St. Anne's University Hospital, Brno, Czechia

**Background and aims:** Epilepsy surgery often fails due to inaccurate localization of the epileptogenic zone (EZ). This study introduces a new method using Graph Neural Networks (GNNs) to analyze interictal biomarkers like epileptiform discharges (IED) and high‐frequency oscillations (HFO). By mapping these features onto a graph that reflects each patient's unique electrode implantation topology, the method aimed to more accurately depict the dynamics of epileptic networks.

**Methods:** A GNN model was developed to pinpoint the EZ, identified as contacts removed during effective epilepsy surgery. The model underwent training and validation through leave‐one‐patient‐out cross‐validation involving 31seizure‐free patients. Interictal biomarkers were detected across 30 minutes of NREM sleep SEEG, then encoded into a graph structure for each patient as node features. In the graph structure, electrode contacts within 8 mm were connected by edges weighted by the Euclidean distance. Benchmark models, LR (Logictic Regression) and SVM (Support Vector Machines), analyzed the same features without considering implantation topology.

**Results:** The GNN model outperformed LR and SVM in terms of median Area Under the Receiver Operating Characteristic (AUROC) and Area Under the Precision‐Recall Curve (AUPRC). Specifically, GNN achieved an AUROC of 0.93 and an AUPRC of 0.69, whereas LR and SVM posted lower scores, nevertheless there were no statistically significant differences between GNN and LR.

**Conclusion:** The GNN model outperformed traditional methods like SVM in modeling SEEG data as graphs, incorporating electrode implantation topology. This approach suggests that acknowledging spatial relationships between electrode contacts, typically overlooked by conventional methods, can significantly enhance localization precision.

**Disclosure:** This work was supported by the Czech Science Foundation, project number 22‐28784S, and by project nr. LX22NPO5107 (MEYS): Financed by European Union – Next Generation EU.

## EPR‐224

### Effect of cenobamate on sudden unexpected death in epilepsy (SUDEP) risk in a Spanish cohort of a phase 3 clinical trial

#### V. Villanueva^1^; J. Leach^2^; K. Thangavelu^3^; P. Pérez‐Domper ^4^; E. Alvarez‐Barón^4^

##### ^1^Hospital Universitari i Politècnic La Fe, Valencia, Spain; ^2^Angelini Pharma S.p.A., Rome, Italy; ^3^MeDaStats LLC, Tampa, FL, USA; ^4^Angelini Pharma España, Madrid, Spain

**Background and aims:** SUDEP accounts for 2‐17% of deaths in patients with epilepsy (Ficker 2000, Epilepsia 41 Suppl 2:S7‐S12). SUDEP‐3 (score 0‐4) and SUDEP‐7 (score 0‐10) scales assess the potential risk of SUDEP. For each point reduction in SUDEP‐3, SUDEP odds decrease by 64%; for SUDEP‐7, per‐point odds decrease is 29% (Rasekhi 2021, Epilepsia 62(7):1536‐1545).

**Methods:** NCT02535091 (C021, *N* = 1340) was a global, multicenter, phase 3, open‐label safety study of cenobamate as adjunctive treatment in adults with uncontrolled focal‐onset seizures (FOS). Efficacy data pre‐ and post‐cenobamate treatment were collected in in a multicenter retrospective observational study of the C021 Spanish cohort (*n* = 127). SUDEP‐3 and SUDEP‐7 risk scores (RS) were calculated for patients in that cohort before and after cenobamate treatment.

**Results:** At baseline, 76% and 24% patients had SUDEP‐3 RS of 2 and 3. After 2 years of cenobamate treatment, 6% had a RS point reduction of 3, 11% of 2, and 14% of 1 point; 69% remained stable (Figure 1). Using the SUDEP‐7 inventory, at baseline, 51% of patients had a RS of 1‐3 and 49% had a RS of 4‐8. After 2 years of cenobamate treatment, 1% had a RS point reduction of 4, 9% of 3, 9% of 2, and 30% of 1 point. 44% remained stable, and 7.5% increased SUDEP risk (Figure 2).

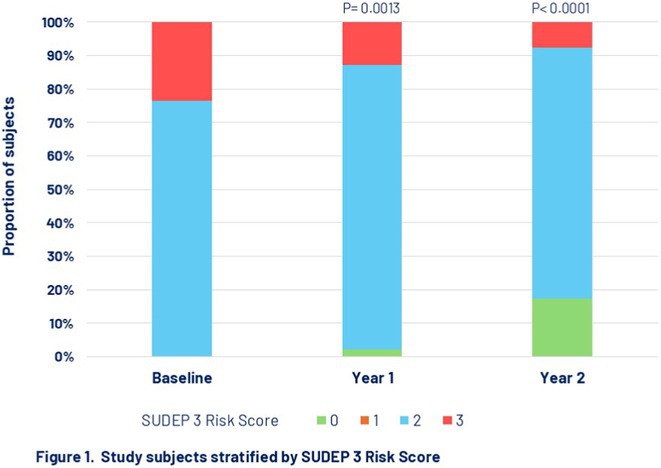

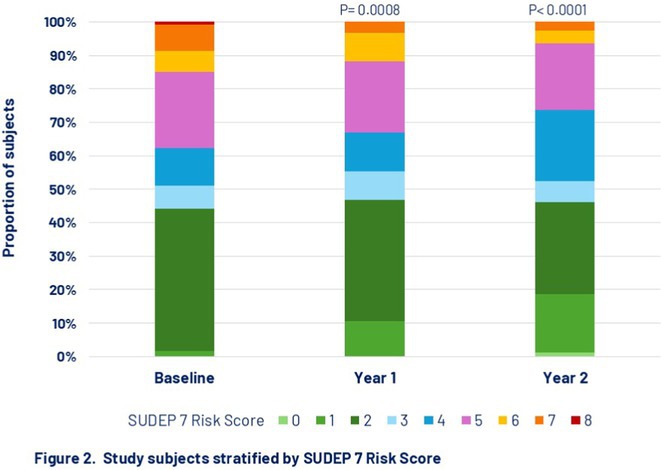

**Conclusion:** Cenobamate treatment significantly reduced SUDEP risk in some Spanish cohort patients as measured by two SUDEP risk scales. The potential for reducing SUDEP risk should be considered when initiating/changing treatment in patients with uncontrolled FOS.

**Disclosure:** The original study C021 (NCT02535091) was supported by SK Life Science, Inc (Paramus, NJ, USA), the Spanish cohort study was supported by Angelini Pharma Spain, and these analyses were supported by Angelini Pharma S.p.A. (Rome, Italy). VV: Consultant/advisor: Angelini Pharma, BIAL, Eisai, Esteve, GlaxoSmithKline, Jazz, Novartis, Sandoz, Takeda, UCB Pharma, Xenon; Speaker: Angelini Pharma, BIAL, Cevomed, Eisai, Esteve, Jazz, Newbridge, Paladin, UCB Pharma; Research support: Angelini Pharma, BIAL, Eisai, Jazz, UCB Pharma. JPL, PPD, EAB: Employees, Angelini Pharma. KT: Consultant: Angelini Pharma.

## EPR‐225

### Peri‐ictal MRI abnormalities in status epilepticus, seizure clusters, and single seizures: A prospective study

#### R. Cutellè ^1^; A. Pascarella^1^; D. Tedeschi^1^; L. Manzo^2^; O. Marsico^1^; C. Mummolo^1^; E. Africa^3^; A. Coglitore^3^; D. Abelardo^1^; C. Prestandrea^2^; C. Paleologo^2^; V. Cianci^2^; A. Armentano^3^; U. Aguglia^1^; G. Kuchukhidze^4^; E. Trinka^4^; E. Ferlazzo^1^

##### ^1^Department of Medical and Surgical Sciences, Magna Graecia University of Catanzaro, Italy; ^2^Neurology Unit, Great Metropolitan “Bianchi‐Melacrino‐Morelli” Hospital, Reggio Calabria, Italy; ^3^Neuroradiology Unit, Great Metropolitan “Bianchi‐Melacrino‐Morelli” Hospital, Reggio Calabria, Italy; ^4^Department of Neurology, Christian Doppler University Hospital, Member of the European Reference Network EpiCARE and Centre for Cognitive Neuroscience, Paracelsus Medical University of Salzburg, Austria

**Background and aims:** Brain MRI frequently identifies peri‐ictal abnormalities (PMA) during or after seizures, especially in status epilepticus (SE), but also in single seizures (SiS) and seizure clusters (CS). The prevalence, features, and natural course of PMA remain poorly understood. This study prospectively evaluated PMA prevalence, clinical correlations, and progression in SE, CS, and SiS.

**Methods:** Patients with SE, CS, and SiS were prospectively enrolled and underwent brain MRI within 120 hours of the ictal event. Demographic, clinical, EEG, and MRI data were collected. For patients with PMA, follow‐up MRI was conducted until resolution. The study assessed the incidence, clinical associations, and progression of PMA across the three groups.

**Results:** Among 76 patients (30 with SE, 22 with CS, and 24 with SiS), PMA were identified in 31 individuals (41%), with significant differences between groups (*p* = 0.011). PMA were less frequent in SiS (17%) compared to SE (57%) and CS (45%). Acute symptomatic SE or seizures were linked to a higher likelihood of PMA (*p* = 0.045), while a history of epilepsy was associated with a lower incidence (*p* = 0.011). The temporal cortex and hippocampus were most frequently affected. Follow‐up MRI in 16 patients showed PMA resolution in 75% of cases, with a median recovery time of 24 days (IQR: 8–39).

**Figure:**
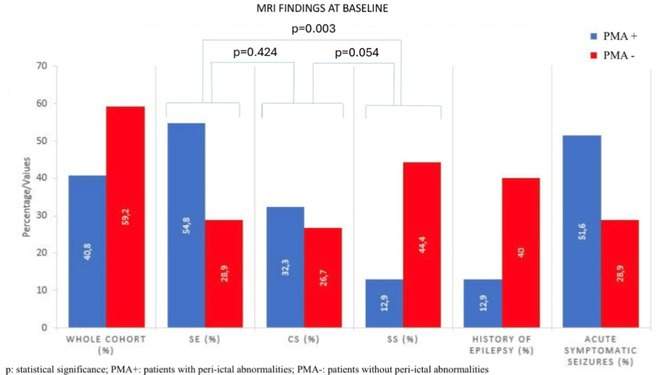
**FIGURE 1** *p*: statistical significance: PMA+: patients with peri‐ictal abnormalities: PMA‐: patients without peri‐ictal abnormalities.

**Conclusion:** PMAs were more common in SE and CS than in SiS, with acute pathology as a key predictor. While ictal duration may contribute to PMA development, it was not the main factor. Most PMAs resolved spontaneously, especially in SiS. Further studies are required to understand their significance.

**Disclosure:** Nothing to disclose.

## Cerebrovascular diseases 3

## EPR‐226

### Intra‐arterial thrombolysis after mechanical thrombectomy in acute ischemic stroke: A meta‐analysis

#### T. Hashmi^1^; H. Ashraf^1^; M. Ahmed^1^; G. Imbianozor^2^; A. Alareed ^3^; R. Ahmed^4^

##### ^1^Rawalpindi Medical University, Rawalpindi, Pakistan; ^2^Royal Wolverhampton NHS Trust. New Cross Hospital, Wolverhampton, UK; ^3^University Hospital Southampton NHS Foundation Trust, UK; ^4^Imperial College London, UK

**Background and aims:** Mechanical thrombectomy (MT) is the standard treatment for acute ischemic stroke (AIS) caused by large vessel occlusions. The potential role of adjunctive intra‐arterial thrombolysis (IAT) in improving outcomes following MT remains unclear. This meta‐analysis evaluates the efficacy and safety of IAT after MT compared to MT alone in AIS patients.

**Methods:** A systematic search was conducted across PubMed, Cochrane Library, and EMBASE to identify RCTs comparing IAT following MT versus MT alone in patients with acute ischemic stroke. Statistical analysis was performed using a random‐effects model in R.

**Results:** Four RCTs encompassing 1,392 patients (MT and IAT: 700; MT alone: 692) met the inclusion criteria. The pooled analysis demonstrated significantly improved excellent functional outcome (mRS 0‐1 at 90d: OR = 1.31, 95% CI = 1.06 to 1.63) in patients receiving IAT after MT compared to MT alone. No statistically significant difference was observed for good functional outcome (mRS 0‐2 at 90d: OR = 1.07, 95% CI = 0.86 to 1.32), symptomatic intracranial hemorrhage (OR = 1.31, 95% CI = 0.74 to 2.34), any intracranial hemorrhage (OR 1.30, 95% CI 0.96 to 1.96) and all‐cause death (OR 0.92, 95% CI 0.70 to 1.21).

**Figure:**
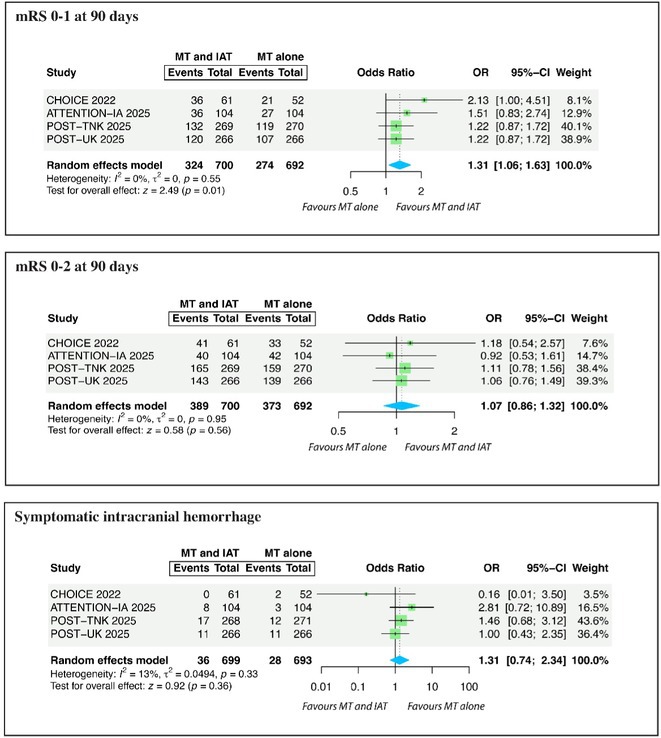
**FIGURE 1** Forest plots for (A) Excellent functional outcome, (B) Good functional outcome, and (C) Symptomatic intracranial hemorrhage.

**Figure:**
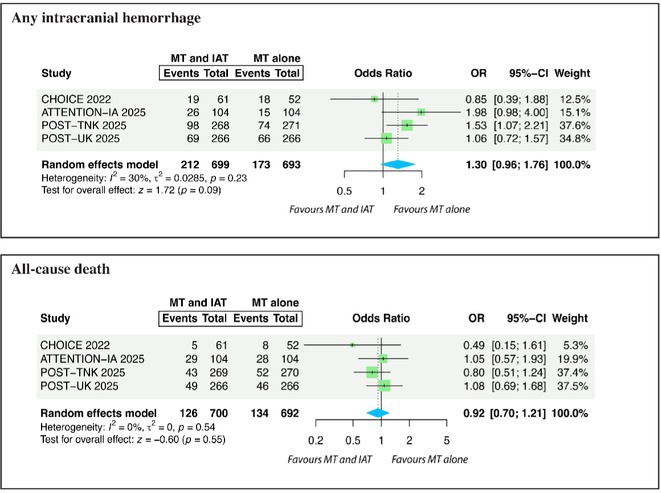
**FIGURE 2** Forest plots for (A) Any intracranial hemorrhage, and (B) All‐cause death.

**Conclusion:** Intra‐arterial thrombolysis after mechanical thrombectomy improves excellent functional outcomes at 90 days in acute ischemic stroke patients, with similar safety profiles to mechanical thrombectomy alone.

**Disclosure:** NA

## EPR‐227

### Association between cardiac morphological changes and cerebrovascular lesion load in patients with atrial fibrillation

#### C. Barbato ^1^; B. Formelli^1^; E. Barucci^1^; M. Berteotti^2^; A. Ginestroni^3^; A. Gori^2^; F. Cesari^2^; B. Giusti^2^; F. Pescini^1^; F. Arba^1^; C. Sarti^1^; E. Salvadori^4^; E. Fainardi^3^; A. Di Carlo^5^; R. Marcucci^2^; A. Poggesi^1^

##### ^1^NEUROFARBA, University of Florence, Italy; ^2^Atherothrombotic Diseases Center ‐ Azienda ospedaliera universitaria Careggi – Florence, Italy; ^3^Department of Neuroradiology ‐ Azienda ospedaliera universitaria Careggi ‐ Florence, Italy; ^4^Depatment of Clinical and Biomedical Science, Milan, Italy; ^5^Italian National Research Council, Florence, Italy

**Background and aims:** Neurological complications of atrial fibrillation(AF) extended beyond cardioembolic stroke and neuroimaging examination of patients with AF have revealed unexpected cerebrovascular features. However, the link between AF and neurological and neuroimaging manifestations remains unclear. To elucidate this relation, we aimed to evaluate the association between cardiac structural changes and cerebrovascular load (either microangiopathic or non‐microangiopathic) in patients with AF.

**Methods:** This is a preliminary cross‐sectional analysis of data derived from the observational, prospective, single center Strat‐AF2study (Stratification of cerebral bleeding risk in AF), enrolling older AF‐patients on oral anticoagulants. All patients underwent a comprehensive clinical assessment, including neuroimaging (CT or MRI) and cardiological evaluations (echocardiography).

**Results:** Among 179 patients (mean age:78.6 years) high prevalence of cerebral small vessels disease markers (cSVD‐m) (Figure 1) and left atria (LA) cardiomyopathy were found. Structural changes in left cardiac chambers, particularly LA‐dilatation and ventricle hypertrophy, were significantly associated with higher burden of cSVD‐m (mainly cerebral atrophy and lacunes), even after adjusting for major cardiovascular risk factors (CRFs). However, in multivariate logistic regression analysis, LA‐dimension was not a key predictor of non‐microangiopathic lesions, whereas CRFs and AF duration remains significant determinants. Main results are presented in Figure 2.

**Figure:**
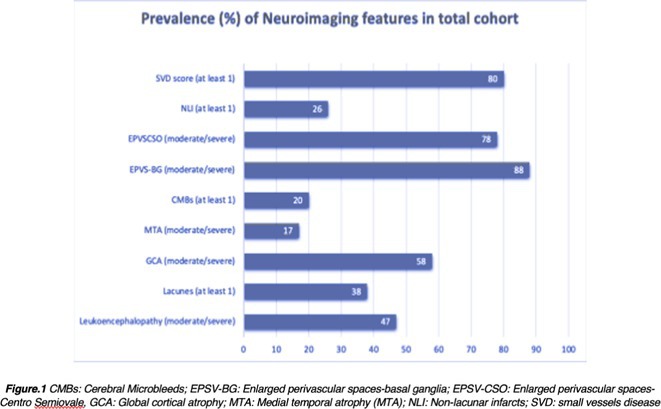
**FIGURE 1** Prevalence of neuroimaging features in the total cohort.

**Figure:**
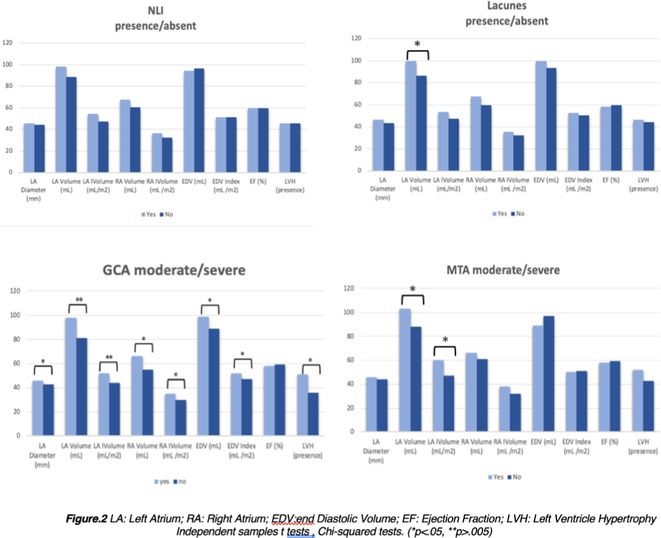
**FIGURE 2** Association between cardiac structural changes and neuroimaging features.

**Conclusion:** This is the first study to examine the link between cardiac parameters and the overall burden of cerebrovascular lesions in AF‐patients. According to our results, we speculate that cSVD‐m may be associated with chronic and systemic effects of left heart changes in AF‐patients. Despite limitations, our findings provide preliminary insight into complex association between cardiac morphological changes and cSVD in AF‐patients.

**Disclosure:** Nothing to disclose.

## EPR‐228

### c‐Jun N‐terminal kinase 3 serum levels in ischemic stroke: Preliminary findings for a potential biomarker

#### F. Ferrari ^1^; F. Mazzacane^1^; B. Del Bello^1^; A. Giani^2^; S. Turchetti^3^; F. Colpo^2^; N. Perta^3^; S. Scaranzin^4^; C. Morandi^4^; A. Persico^5^; M. Gastaldi^4^; A. Cavallini^5^; T. Borsello^2^

##### ^1^Department of Brain and Behavioral Sciences, University of Pavia, Pavia, Italy; ^2^Neuronal death and neuroprotection laboratory Department of Pharmacological and Biomolecular Sciences, via Balzaretti 9, Milan University, Italy.; ^3^Department of Life and Environmental Sciences, Polytechnic University of Marche, Ancona, Italy; ^4^Neuroimmunology Unit, IRCCS Neurological Institute Foundation Mondino, Pavia, Italy; ^5^Cerebrovascular Disease & Stroke Unit, IRCCS Neurological Institute Foundation Mondino, Pavia, Italy

**Background and aims:** Reliable biomarkers for stroke are essential for identifying therapeutic targets. Brain‐specific JNK3, implicated to synaptic dysfunction in experimental stroke models, has yet to be explored in clinical settings. This observational prospective study aimed to investigate serum JNK3 concentrations in stroke patients and their correlation with neuronal damage marker Neurofilament‐Light‐Chain (NfL).

**Methods:** We included 18‐80‐year‐old patients with stroke at neuroimaging, onset < 24h, NIHSS > 1, pre‐stroke mRS < 2, without: previous stroke/TBI/other neurological disease, chronic immunosuppression, pregnancy, eGFR < 30mL/min. As comparison, subjects hospitalized for acute neurologic symptoms meeting enrollment criteria except for ischemic lesion at neuroimaging (NL) were considered. Sera were collected within 24h (T0) from patients and NL, after 3‐5d (T1) and 7 ± 2d (T2) in patients. JNK3 levels were measured by commercial ELISA kit, NfL by Ella Automated‐Immunoassay‐System.

**Results:** At present, 96 patients have been enrolled (49M, mean age 64.5y ± 1.3). At onset, 51% had minor (NIHSS:1‐4), 40.6% moderate (NIHSS:5‐15), 8.3% severe stroke (NIHSS > 16). NL are 12 (8M, mean age 63.2y ± 4.5). At T0, JNK3 was higher in patients (2.84[IQR 0.28]) vs. NL (2.48[IQR 0.19]; *p* < 0.001). Across the considered time‐points, JNK3 was not different, while NfL was lower at T0 vs. T1, and T0 vs. T2 (*p* < 0.001) (Fig. 1). No correlations were found between JNK3 and NfL concentrations.

**Figure:**
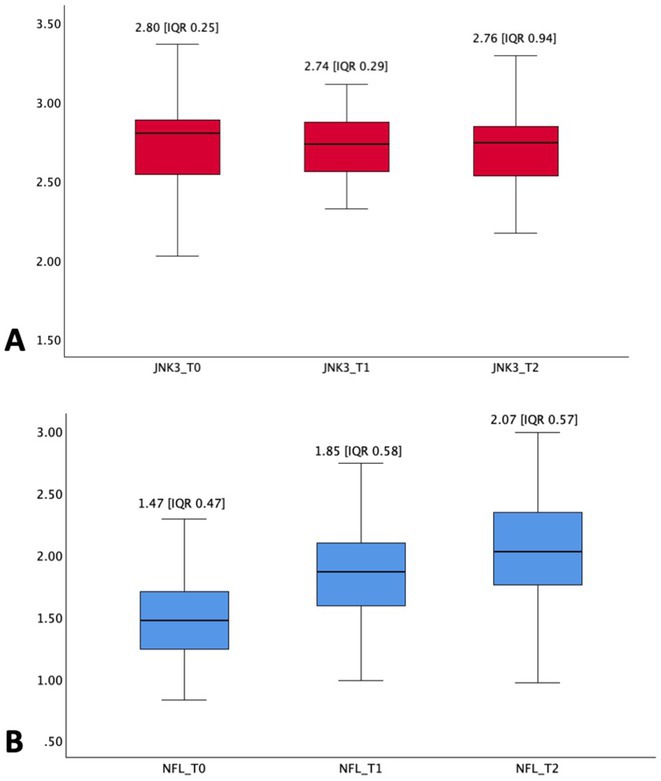
**FIGURE 1** JNK3 (A) and NfL (B) serum concentrations in stroke patients at T0 (24h), T1(3‐5d), T2(7 ± 2d), expressed as Log10(pg/mL). Indicated are median values with interquartile range (IQR).

**Conclusion:** Serum JNK3 levels were measurable in humans and were elevated when an ischemic lesion was present, indicating JNK3 release after stroke. No significant temporal differences were observed at the considered time‐points, suggesting slower kinetics or distinct release mechanisms versus NfL, with which no correlations were documented. Further research to explore JNK3 in stroke is needed.

**Disclosure:** Funding: “Ricerca Corrente 2022‐2024” granted by Italian “Ministero della Salute” (IRCCS Mondino Foundation); SEED for Innovation Patent 2.0 (SEED4IP 2.0) granted by Italian “Ministero dello Sviluppo Economico”, now renamed “Ministero delle imprese e del Made in Italy ‐MIMIT”. Double affiliations: FF: Cerebrovascular Disease & Stroke Unit, IRCCS Neurological Institute Foundation Mondino, Pavia, Italy; TB, AG, ST, NP: Mario Negri Institute for Pharmacolgical Research‐IRCCS, Department of Neuroscience, Via Mario Negri 2, 20156 Milano, Italy.

## EPR‐229

### Tenecteplase versus alteplase in patients with acute ischemic stroke: An updated meta‐analysis

#### M. Sheraz^1^; N. Asif^2^; A. Khan^3^; M. Khan^4^; M. Rehan^5^; M. Ch^6^; A. Khalid^7^; C. Alfieri^8^; E. Bouyarden^9^; M. Ghenai^9^; M. Ahmed ^10^; M. Ehsan^2^; R. Ahmed^11^; G. Imbianozor^12^; A. Alareed^13^

##### ^1^Department of Medicine, Continental Medical College, Lahore, Pakistan; ^2^Department of Medicine, King Edward Medical University, Lahore, Pakistan; ^3^Department of Medicine, Services Institute of Medical Sciences, Lahore, Pakistan; ^4^Department of Radiology, Khyber Teaching Hospital, Peshawar, Pakistan; ^5^Department of Medicine, Faisalabad Medical University, Pakistan; ^6^Department of Medicine, Rashid Latif Medical College, Lahore, Pakistan; ^7^Department of Medicine, Faisalabad Medical University, Faisalabad, Pakistan; ^8^Department of Medicine, The Royal College of Physicians in Ireland, Dublin, Ireland; ^9^Faculty of Medecine, Université de Montreal, Canada, ^10^Department of Medicine, Rawalpindi Medical University, Rawalpindi, Pakistan, ^11^Royal Brompton Hospital, London, UK, ^12^Royal Wolverhampton NHS Trust. New Cross Hospital, Wolverhampton, ^13^University Hospital Southampton NHS Foundation Trust, UK

**Background and aims:** Acute ischemic stroke (AIS) is a leading cause of morbidity and mortality worldwide. While alteplase has been widely used for acute management, recent clinical trials suggest that tenecteplase (TNK) may offer improved clinical outcomes. This study aimed to compare the efficacy and safety of TNK compared with alteplase.

**Methods:** A comprehensive literature search was conducted using PubMed, Embase and Cochrane Library from inception to October 2024 to identify randomized controlled trials that compared TNK at 0.25 mg/kg dosage with alteplase. Data about clinical outcomes was extracted from both groups and assessed by generating forest plots using the random‐effects model and pooling odds ratios (ORs).

**Results:** A total of 11 RCTs with 7,546 patients were included in the analysis. TNK showed statistically significant improvement in excellent functional outcome (mRS 0‐1) compared with alteplase (OR = 1.14, 95% CI = 1.03‐1.25). No statistically significant difference was observed for good functional outcome (mRS 0‐2) (OR = 1.11, 95% CI = 0.9‐1.25), early neurological improvement (OR = 1.08, 95% CI = 0.93‐1.26), all‐cause death (OR = 0.99, 95% CI = 0.08‐1.22), symptomatic intracranial hemorrhage (OR = 1.16, 95% CI = 0.84‐1.59) and poor functional outcome (mRS = 4‐6) (OR = 0.95, 95% CI = 0.79‐1.14).

**Figure:**
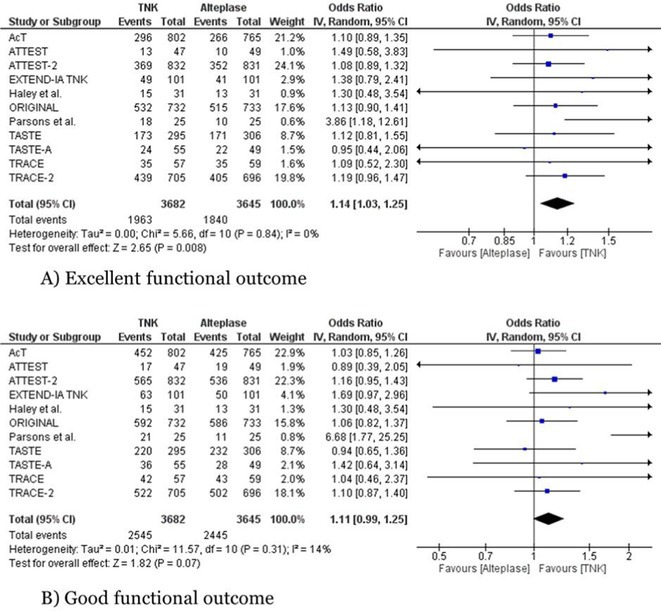
**FIGURE 1** Forest plots for (A) Excellent functional outcome (B) Good functional outcome.

**Figure:**
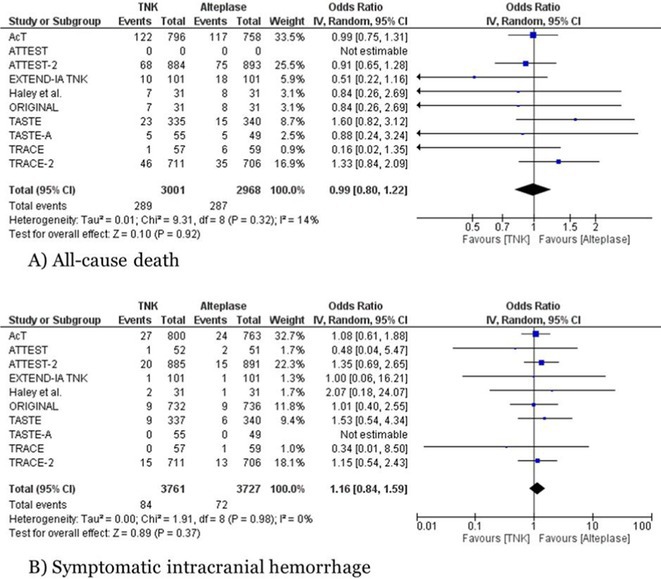
**FIGURE 2** Forest plots for (A) All‐cause death (B) symptomatic intracranial hemorrhage.

**Figure:**
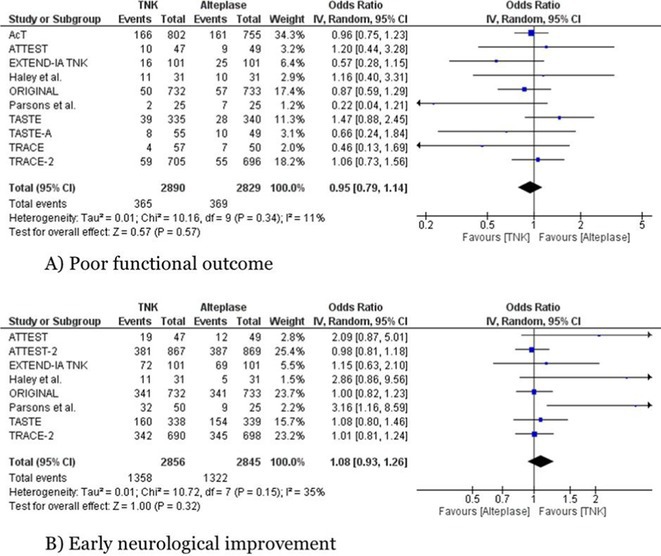
**FIGURE 3** Forest plots for (A) Poor functional outcome (B) Early neurological improvement.

**Conclusion:** In patients with acute ischemic stroke, TNK demonstrated a significant advantage over alteplase in achieving excellent functional outcomes. The incidence of early neurological improvement, symptomatic intracranial hemorrhage, all‐cause death, and poor functional outcome remained comparable across the two groups.

**Disclosure:** NA

## EPR‐230

### Patent foramen ovale closure in patients older than 60 years. 6‐year experience of a comprehensive stroke center

#### Ó. Camejo ^1^; J. Rodríguez Pardo^1^; P. López Grueiro^1^; R. Rigual^1^; G. Ruiz Ares^1^; G. Galeote^2^; C. Hervás Testal^1^; E. De Celis^1^; L. Casado^1^; M. Alonso de Leciñana^1^; A. Martínez Roca^2^; B. Fuentes^1^

##### ^1^Neurology Department and Stroke Center. Hospital La Paz Institute for Health Research – IdiPAZ (La Paz University Hospital – Universidad Autónoma de Madrid), Madrid, Spain; ^2^Department of Cardiology. La Paz University Hospital. IdiPAZ, Madrid, Spain

**Background and aims:** Patent foramen ovale (PFO) is a well‐known cause of stroke in the young, but the benefit of PFO closure in older patients remains unclear. We aimed to evaluate the differences in outcomes of patients with PFO‐attributed stroke according to age.

**Methods:** Retrospective observational study of patients with PFO‐attributed stroke from 2018 to 2024. We compared clinical characteristics, procedural complications, atrial fibrillation (AF) and stroke recurrence between younger (≤60 years) and older (> 60 years) groups.

**Results:** We included 102 patients. Thirty (29%) were > 60 year old. Older patients had higher rates of hypertension (50% vs. 4%, *p* < .001) and cortical infarction (63% vs. 38%, *p* = .017), higher NIHSS score on admission (median [IQR] 3 [0‐12] vs. 2 [0‐5], *p* = 0.006), lower RoPE scores (median [IQR] 4 [2‐6] vs. 7 [4‐10]), and underwent percutaneous closure less frequently (77% vs. 97%, *p* = .001) than younger patients. There were no differences in PFO size or frequency of procedural complications (7/70 in the younger and 2/23 in the older group, all minor complications). Patients with minor complications had longer PFO tunnels (median [IQR] 21 mm [11‐31] vs. 10 mm [8‐13], *p* = .032). AF was observed in three (4.3%) treated patients from the younger cohort (all in the first two months after procedure), and in none of the older cohort. No stroke recurrence was observed in either group over a median follow‐up of 3 [1‐4] years.

**Figure:**
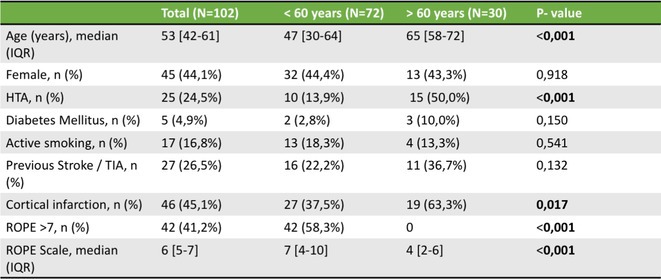
**TABLE 1** Demographic data.

**Figure:**
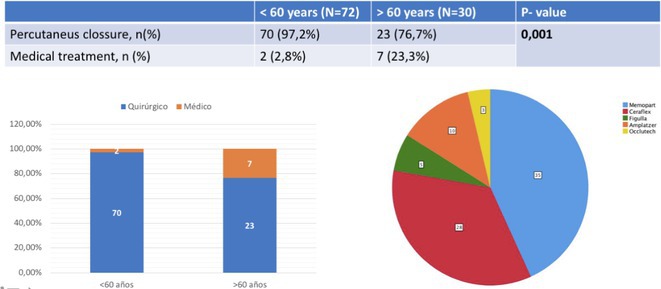
**FIGURE 1** Treatment of PFO

**Figure:**
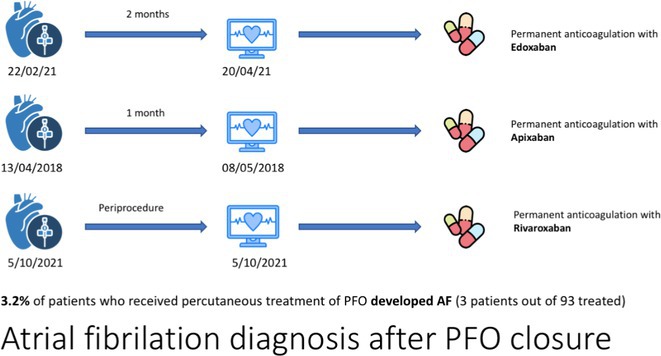
**FIGURE 2** AF after closure

**Conclusion:** Procedural complications and early recurrences of percutaneous PFO closure seem as uncommon in > 60 years as in younger patients with PFO‐attributed stroke.

**Disclosure:** Nothing to disclose.

## EPR‐231

### Carotid strain predicts 3‐year stroke and coronary risk in metabolic syndrome: A new diagnostic insight

#### S. Arnautu ^1^; D. Arnautu^1^; D. Jianu^2^

##### ^1^Internal Medicine Department, Victor Babes University of Medicine and Pharmacy, Timisoara, Romania; ^2^Neurology Department, Victor Babeș University of Medicine and Pharmacy, Timișoara, Romania

**Background and aims:** This study aimed to determine if carotid strain and strain rate might predict major cardiovascular events (MACE) in individuals with metabolic syndrome (MS) during a three‐year period.

**Methods:** This prospective observational study included 220 adult MS patients (60.7 ± 7.5 years old, 53% male). CS and CSR were measured using bilateral 2D common carotid artery (CCA) speckle‐tracking ultrasonography. Clinical outcomes were monitored for three years.

**Results:** After a 3‐year follow‐up, 14 (7%) had MACE: Four (2%) experienced acute coronary syndrome, eight (4%) had atherothrombotic ischemic stroke, and two (1%) were hospitalized for heart failure. Univariate regression analysis of MS patients' clinical and echocardiographic parameters indicated that age, systemic hypertension, diabetes, and CCA circumferential strain and strain rate were substantially linked with MACE risk. Using multivariate logistic regression, two independent predictors of MACE in MS patients were identified: CCA‐related CS (%) and CSR (1/s), *p* < 0.01. The ROC curve assessments of these independent MACE predictors showed suitable sensitivities and specificities. CS: AUC = 0.806 (sensitivity = 82.6%, specificity = 79.2%, *p* < 0.0001); CSR: AUC = 0.779 (sensitivity = 82.6%, specificity = 72.4%, *p* < 0.0001). The cut‐off values were ≤2.9% for carotid CS and ≤0.35 s–1 for CSR. According to Kaplan‐Meier survival curves, MS patients with lower carotid CS and CSR had significantly decreased MACE, ischemic stroke, and ACS‐free survival (*p* < 0.0001).

**Figure:**
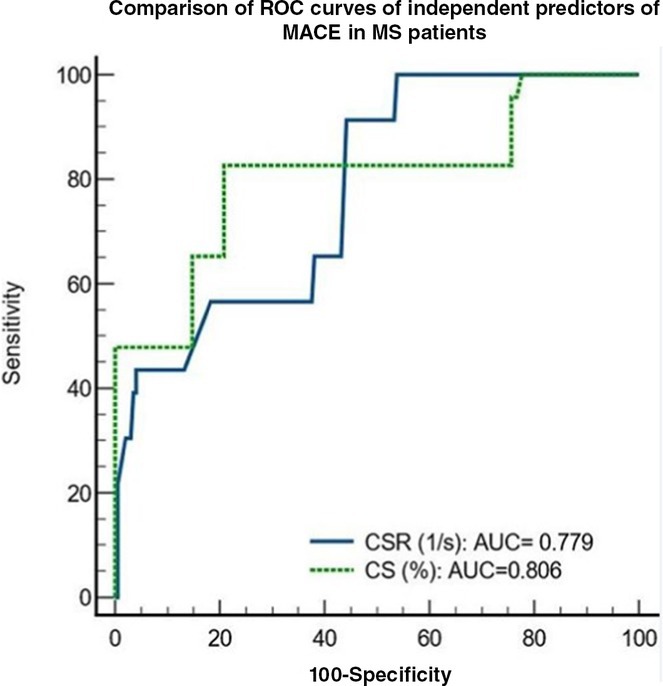
**FIGURE 1** Comparison of ROC curves

**Figure:**
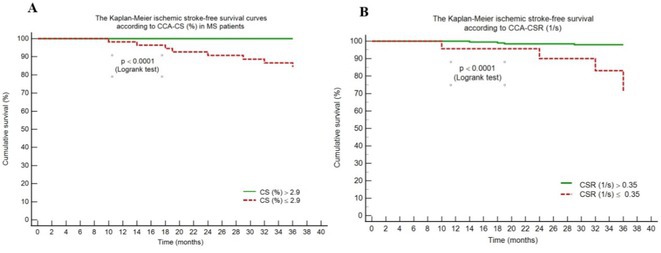
**FIGURE 2** The Kaplan‐Meier MACE‐free survival curves in metabolic syndrome patients according to common carotid artery circumferential strain and strain rate rate.

**Conclusion:** Carotid CS and CSR independently predicted major cardiovascular and cerebrovascular events in prospectively studied MS patients without cardiovascular disease. In this demographic, carotid distortion may indicate cardiovascular risk early on.

**Disclosure:** Nothing to disclose.

## EPR‐232

### Safety and efficacy outcomes of LAAO in patients with prior ICH or cerebral amyloid angiopathy: A meta‐analysis.

#### T. Mavridis ^1^; P. Archontakis‐Barakakis^2^; D. Chlorogiannis^3^; A. Charidimou^4^

##### ^1^Department of Neurology, Tallaght University Hospital (TUH), Dublin, Ireland; ^2^Redington‐Fairview General Hospital, Skowhegan, Maine, USA; ^3^Department of Radiology, Brigham and Women's Hospital, Harvard Medical School, Boston, Massachusetts, USA; ^4^Department of Neurology, Boston University Medical Center, Boston University School of Medicine, Massachusetts, USA

**Background and aims:** Patients under oral anticoagulation for atrial fibrillation (AF) with a history of intracerebral hemorrhage (ICH) or cerebral amyloid angiopathy (CAA), have and increased risk of recurrent ICH. In those high‐bleeding risk patients, left atrial appendage occlusion (LAAO) provided a promising alternative. Limited data from observational studies have shown that LAAO is safe and feasible with antiplatelet therapy or short‐term OAC post‐procedure. This meta‐analysis aimed to provide data regarding the safety and efficacy of LAAO in patients with prior ICH or CAA.

**Methods:** PubMed/MEDLINE and EMBASE were systematically reviewed for randomized control trials, observational studies, and case series reporting stroke (ischemic and hemorrhagic) events in patients with AF undergoing LAAO who had a history of previous ICH and/or CAA. Pooled incidence rates (IRs) with corresponding 95% confidence intervals (CIs) were calculated for primary (post‐procedural stroke and recurrent ICH) and secondary outcomes.

**Results:** Fourteen studies were included in the final analysis including 1,235. The pooled IRs for recurrent ICH, ischemic stroke, and all‐cause mortality were 0.02% (95%CI: 0.004%–0.03%), 0.02% (95%CI: 0.01%–0.03%), and 0.04% (95%CI: 0.01%–0.08%), respectively (Figures 1‐3). Subgroup analyses of patients with intraparenchymal hemorrhage and/or CAA reported pooled IRs of 0.04% (95% CI: 0.004–0.1%) for recurrent ICH, 0.04% (95% CI: 0.01–0.08%) for ischemic stroke, and 0.09% (95% CI: 0.01–0.22%) for all‐cause mortality (Figures 1‐3).

**Figure:**
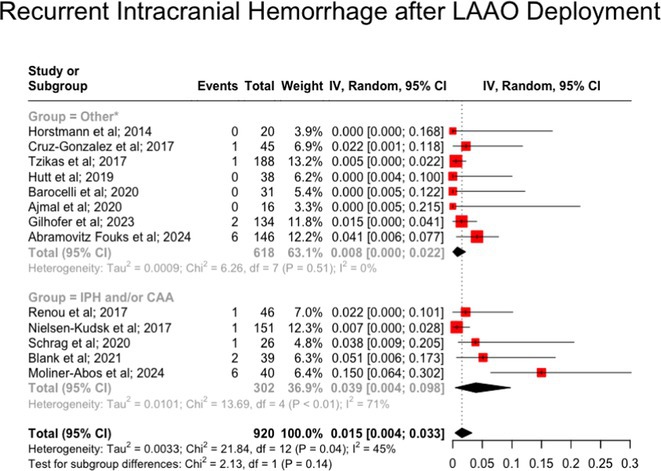
FIGURE 1

**Figure:**
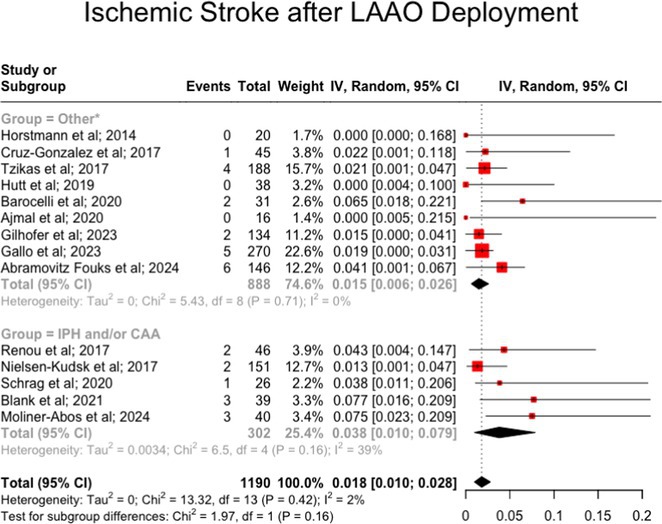
FIGURE 2

**Figure:**
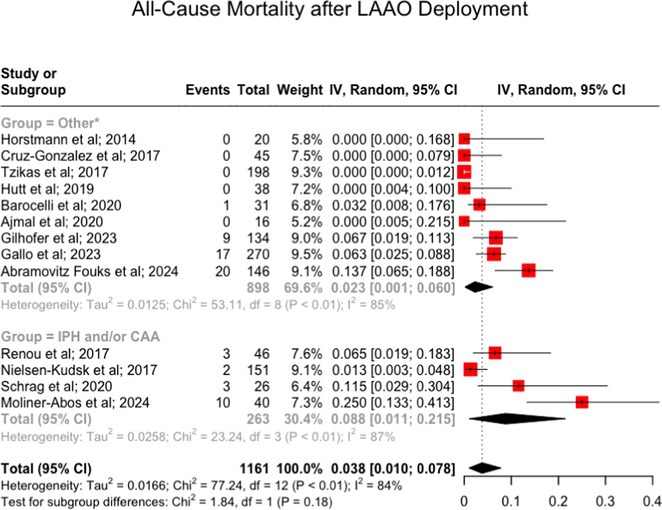
FIGURE 3

**Conclusion:** For selected AF patients with a history of ICH and/or CAA, LAAO is considered safe and effective, exhibiting low rates of major cerebrovascular events.

**Disclosure:** Nothing to disclose.

## EPR‐234

### Protective effects of bavachin in a rat middle cerebral artery occlusion stroke model

#### Y. Ma; T. Lam; R. Cheung

##### Department of Medicine, School of Clinical Medicine, Li Ka Shing Faculty of Medicine, The University of Hong Kong, Hong Kong, China

**Background and aims:** Bavachin is a flavonoid isolated from the seeds of Psoralea corylifolia. Recent studies have shown that bavachin inhibits neuroinflammation and protects against lipopolysaccharide‐induced oxidative stress in mice. However, its protective effects in ischemic stroke remain unclear. In this study, we investigated the therapeutic potentials of bavachin and its underlying mechanisms in a rat middle cerebral artery occlusion (MCAO) stroke model.

**Methods:** Adult male Sprague Dawley (SD) rats underwent right‐sided MCAO for 90 minutes with intraperitoneal injection of with bavachin at 40 mg/kg or its vehicle for 3 consecutive days. The first dose was given 3 hours before MCAO onset, and the remaining doses were given on days 1 and 2 after MCAO. Body weight and a modified Neurological Severity Score (mNSS) were assessed daily before and after MCAO until sacrifice on day 3 when infarct volume, cerebral edema index, brain weight, spleen, and thymus index were determined.

**Results:** Compared with the sham group, the thymus index was significantly decreased after MCAO; this was reversed by bavachin treatment. Bavachin also significantly reduced the infarct volume and mNSS but not the spleen index on day 3. Furthermore, the cerebral edema index was significantly increased after MCAO; this was not affected by bavachin treatment. Finally, bavachin did not affect the body weight.

**Conclusion:** The present results indicate that bavachin has some neuroprotective effects in a rat MCAO stroke model. Bavachin may be a potential treatment for ischemic stroke. However, the underlying mechanisms need to be further investigated.

**Disclosure:** Nothing to disclose.

## Motor neurone diseases

## EPR‐235

### Connecting SBMA international registries/databases: A retrospective study

#### A. Bertini ^1^; S. Fenu^1^; C. Grunseich^2^; C. Koniari3; L Zampedri^4^; C. Rinaldi^5^; L. Blasi6; A Cakar^7^; G. Querin^8^; P. Pradat^8^; J. Vissing^9^; E. Cavalca1; S Bernsen^10^; P. Weydt10; S Yamada^11^; K. Schellenberg^12^; G. Pfeffer^13^; Y. Parman^7^; G. Karadima^3^; G. Koutsis^3^; J. Palmio^14^; M. Jokela^14^; A. Conte^15^; M. Sabatelli^15^; P. Fratta^4^; M. Katsuno^11^; J. Park^16^; G. Sorarù^6^; D. Pareyson^1^

##### ^1^Unità di Malattie Neurologiche Rare, Dipartimento di Neuroscienze Cliniche, Fondazione IRCCS Istituto Neurologico Carlo Besta, Milan, Italy; ^2^Neurogenetics Branch, National Institute of Neurological Disorders and Stroke, National Institutes of Health, Bethesda, Maryland, USA; ^3^Neurogenetics Unit, 1st Department of Neurology, National and Kapodistrian University of Athens, Eginitio Hospital, Athens, Greece; ^4^University College of London, UK; ^5^Institute of Developmental and Regenerative Medicine (IDRM), University of Oxford, UK; ^6^Neuromuscular Unit, Neurology Department, Istanbul Faculty of Medicine, Istanbul University, Istanbul, Turkey; ^7^Département de Neurologie, Hôpital Pitié‐Salpêtrière, Centre référent SLA, AP–HP, Paris, France; ^8^Copenhagen Neuromuscular Center, Department of Neurology, Rigshospitalet, University of Copenhagen, Copenhagen, Denmark; ^9^Department of Neuromuscular Diseases, Center for Neurology, University Hospital Bonn, Bonn, Germany, ^10^Department of Neurology, Nagoya University Graduate School of Medicine, Nagoya, Aichi, Japan, ^11^Division of Neurology, Department of Medicine, University of Saskatchewan, Saskatoon, Saskatchewan, Canada, ^12^Department of Clinical Neurosciences, University of Calgary, Calgary, Alberta, Canada, ^13^Neuromuscular Research Center, Department of Neurology, Tampere University and University Hospital, Tampere, Finland, ^14^Fondazione Policlinico Universitario Agostino Gemelli IRCCS., Rome, Italy, ^15^Fondazione Policlinico Universitario Agostino Gemelli IRCCS, ^16^Department of Neurology, School of Medicine, Kyungpook National University, Kyungpook National University Chilgok Hospital, Daegu, Republic of Korea

**Background and aims:** Over the years, several groups have collected retrospective data on different national series of Spinal‐Bulbar Muscular Atrophy (SBMA) subjects in national databases/registries. We propose a retrospective study collating together data across different populations into a large international database, in order to better understand clinical/laboratory features, compare disease across different countries, and detect disease evolution on a global scale.

**Methods:** Centers from following countries accepted the invitation to join Italy in this project and already shared data: USA, Germany, South Korea, Finland, Greece, Denmark, Turkey. Data are due to be sent by UK, France, Japan. In Canada, the majority of patients have Indigenous ancestry, and participation is pending a community engagement process. We collected data with greatest overlap across the different databases, including symptoms at onset, symptoms progression, medications, co‐morbidities, MRC of 6 muscle pairs, bulbar involvement, cardiologic/respiratory parameters (ECG, spirometry), outcome measures/scales (SBMA‐FRS/ALS‐FRS, AMAT, IPSS, IIEF scales, and two/six minutes walking test), extensive lab tests, follow‐up visits, and outcome.

**Results:** To date, we have collected data on 335 patients: 139 from Italy, 87 from Finland, 29 from Denmark, 25 from USA, 21 from Greece, 20 from South Korea, 8 from Turkey, 6 from Germany. We estimate that data from ~320 more patients will be integrated from UK, Canada, France, Japan.

**Conclusion:** Through an international joint effort, we created an international SBMA dataset that potentially encompasses up to ~650 subjects, with the aim of better characterize disease course, and explore regional differences within the disease. Funded by KDA‐Kennedy Disease Association

**Disclosure:** AB acknowledges support of EAN fellowship.

## EPR‐236

### Iron metabolism in als patients: A cohort study

#### B. Risi ^1^; I. Bianchi^2^; F. Caria^3^; B. Labella^4^; E. Bertella^3^; L. Poli^5^; L. Ferullo^4^; E. Olivieri^4^; A. Padovani^4^; I. Zanella^6^; M. Filosto^7^

##### ^1^NeMO‐Brescia Clinical Center for Neuromuscular Diseases, Brescia, Italy; Department of Molecular and Translational Medicine, University of Brescia, Brescia, Italy; ^2^Medical Genetics Laboratory, ASST “Spedali Civili”, Brescia, Italy; ^3^NeMO‐Brescia Clinical Center for Neuromuscular Diseases, Brescia, Italy; ^4^Unit of Neurology, ASST “Spedali Civili”, Brescia, Italy; Department of Clinical and Experimental Sciences, University of Brescia, Brescia, Italy; ^5^Unit of Neurology, ASST “Spedali Civili”, Brescia, Italy; ^6^Medical Genetics Laboratory, ASST “Spedali Civili”, Brescia, Italy; Department of Molecular and Translational Medicine, University of Brescia, Brescia, Italy; ^7^NeMO‐Brescia Clinical Center for Neuromuscular Diseases, Brescia, Italy; Department of Clinical and Experimental Sciences, University of Brescia, Brescia, Italy

**Background and aims:** Given the evidence for altered iron metabolism in ALS, we aimed to investigate the relationship between iron homeostasis, genetic background and clinical variables in a larger cohort.

**Methods:** 77 ALS patients were tested for ferritin, iron and transferrin. A subset of 46 patients were also tested for common variants in iron‐related genes, including ERFE, HFE, IL6, IL6R, SLC11A1. ANCOVA, adjusted correlation analysis and multiple linear regression were performed to test significant associations with clinical variables. Ferritin levels were normalized to the mean values in the men and female groups.

**Results:** Ferritin levels were abnormally elevated in 50% of men and 59.3% of women and correlated positively with the neutrophil to lymphocytes ratio (NLR) (*p* = 0.021) and negatively with the ALSFRS‐R (*p* = 0.001). Iron levels were lower than normal in 7.8% of the patients and correlated negatively with the NLR (*p* = 0.012) and positively with the ALSFRS‐R (*p* = 0.006). Transferrin levels were lower than normal in 44.7% and correlated negatively with the NLR (*p* = 0.049). The ALSFRS‐R was lower in SLC11A1 carriers (*p* = 0.047), whereas the disease progression rate (DPR) and the NLR were both higher in ERFE carriers (*p* = 0.003 and *p* = 0.020, respectively). IL6R carriers had lower ferritin levels than non‐carriers (*p* = 0.003). IL6R status was the only predictor of ferritin levels (*p* = 0.003) among sex, site of onset, disease duration and DPR.

**Conclusion:** Altered iron homeostasis in ALS is associated with systemic inflammation and high disease burden. This study also suggests a possible protective role of the IL6R D358A variant against higher ferritin levels, highlighting the importance of genetic characterization for disease prognosis.

**Disclosure:** Nothing to disclose.

## EPR‐237

### The CCL2‐CCR2 axis drives neuromuscular denervation in ALS

#### B. Nógrádi ^1^; K. Molnár^2^; R. Kristóf^2^; Y. Huang^3^; Z. Ridgway^3^; A. Elicegui^4^; S. Alonso‐Martin^4^; G. Szebeni^5^; N. Gémes^5^; A. Ramadan^3^; H. Smith^3^; O. Horváth^1^; I. Krizbai^2^; R. Patai^2^; L. Siklós^2^; P. Klivényi^1^; H. Chaytow^3^; T. Gillingwater^3^

##### ^1^Department of Neurology, Albert Szent‐Györgyi Health Centre, University of Szeged; Szeged, Hungary; ^2^Department of Biophysics, HUN‐REN Biological Research Centre; Szeged, Hungary; ^3^Edinburgh Medical School: Biomedical Sciences, University of Edinburgh; Edinburgh, UK; ^4^Stem Cells and Aging Group, Biogipuzkoa Health Research Institute; San Sebastian, Spain; ^5^Laboratory of Functional Genomics, Core Facility, HUN‐REN Biological Research Centre; Szeged, Hungary

**Background and aims:** Systemic immune changes have been implicated in the pathogenesis of amyotrophic lateral sclerosis (ALS), but the precise mechanisms and cellular targets remain unknown. Here we aimed to characterize the inflammatory response around neuromuscular junctions (NMJs) in skeletal muscle and evaluate the potential therapeutic effects of modulating these immune pathways.

**Methods:** Confocal microscopy and proteomic analysis were used to characterize the immune alterations in skeletal muscle of ALS patients and the hTDP‐43 mouse model of ALS. Local antibody‐based treatment was administered to examine the effects of CCL2 suppression in hTDP‐43 mice. Treatment efficiency was evaluated via immune cell quantification and NMJ analysis.

**Results:** We observed a marked leukocyte infiltration in the skeletal muscle of ALS patients. Major leukocyte and macrophage infiltration was recapitulated in hTDP‐43 mice, most notably in close proximity to NMJs and in muscles severely affected by denervation, such as gastrocnemius. Proteomic analysis revealed elevated levels of chemokines CCL2, 3, 4 and 5 in gastrocnemius, but not in less affected muscles. Increased numbers of CCL2+ cells were located near NMJs in hTDP‐43 mice, alongside CCR2 receptor‐expressing immune cells, present from pre‐symptomatic stages of disease. Increased numbers of CCR2+ cells were also observed in skeletal muscle from ALS patients. Local immunomodulatory treatment with either CCL2‐neutralising antibodies or normal IgG antibodies in hTDP‐43 mice reduced leukocyte infiltration and robustly ameliorated NMJ denervation.

**Conclusion:** These results demonstrate that the CCL2‐CCR2 axis drives immune cell infiltration targeting NMJs in ALS and suggest that these immune pathways can be therapeutically modulated to protect NMJs from denervation.

**Disclosure:** THG has provided advisory services for Roche and Novartis. SA‐M is a named inventor on a patent related to neurological disorders. SA‐M also have ownership in Miaker Developments S.L., a startup related with a pipeline on Neurodegenerative and Neuromuscular Diseases.

## EPR‐238

### Safety and efficacy data from the phase 2 ARDA study of empasiprubart in multifocal motor neuropathy

#### L. Querol ^1^; T. Harbo^2^; S. Peric^3^; Y. Hussain^4^; S. Cadour^5^; I. Van de Walle^5^; E. Persson^5^; M. Vujcic^5^; O. Van de Steen^5^; J. Allen^6^; E. Nobile‐Orazio^7^; S. Attarian^8^; C. Karam^9^; H. Katzberg^10^; M. Stettner^11^; S. Rinaldi^12^; W. van der Pol^13^

##### ^1^Department of Neurology, Neuromuscular Diseases Unit, Hospital de La Santa Creu I Sant Pau, Universitat Autònoma de Barcelona, Barcelona, Spain; Centro De Investigación Biomédica en Red en Enfermedades Raras (CIBERER), Madrid, Spain; ^2^Department of Neurology, Aarhus University, Aarhus, Denmark; ^3^University of Belgrade, Faculty of Medicine, Neurology Clinic, University Clinical Center of Serbia, Belgrade, Serbia; ^4^Austin Neuromuscular Center, Austin, TX, USA, ^5^argenx, Ghent, Belgium; ^6^Department of Neurology, University of Minnesota, Minneapolis, MN, USA; ^7^Neuromuscular and Neuroimmunology Unit, IRCCS Humanitas Research Hospital, Department of Medical Biotechnology and Translational Medicine, University of Milan, Milan, Italy; ^8^Referral Centre for Neuromuscular Diseases and ALS, Hôpital La Timone, Marseille, France; ^9^Department of Neurology, University of Pennsylvania, Philadelphia, PA, USA, ^10^Ellen & Martin Prosserman Centre for Neuromuscular Diseases, Toronto General Hospital, University Health Network, University of Toronto, Toronto, ON, Canada, ^11^Department of Neurology, Essen University Hospital, University Duisburg‐Essen, Essen, Germany, ^12^Nuffield Department of Clinical Neurosciences, University of Oxford, Oxford, UK, ^13^Department of Neurology and Neurosurgery, University Medical Center Utrecht, Utrecht, The Netherlands

**Background and aims:** Multifocal motor neuropathy (MMN) is a rare, immune‐mediated, chronic neuropathy leading to axonal degeneration and progressive, disabling, asymmetric limb weakness. Empasiprubart binds C2, blocking classical and lectin complement pathways involved in MMN pathophysiology. In a neuropathy ex vivo mouse model, blocking C2 prevented neurofilament light protein (NfL) loss, maintaining axonal integrity. The phase 2 ARDA (NCT05225675) study assessed the safety and efficacy of empasiprubart in adults with MMN.

**Methods:** Enrolled participants had probable/definite MMN (2010 EFNS/PNS guidelines) and proven intravenous immunoglobulin (IVIg) dependency and were on a stable IVIg regimen before randomization. Participants were assigned to 1 of 2 dosing cohorts, each randomized 2:1 to empasiprubart or placebo. Efficacy endpoints included IVIg retreatment rate, Patient Global Impression of Change scale score, change from baseline in grip strength (most affected hand), and serum NfL concentration in the double‐blind treatment period.

**Results:** 27 participants (empasiprubart, *n* = 18; placebo, *n* = 9) were randomized per cohort. Cohort 2 participants were older, with longer duration of disease and time since first IVIg treatment. Empasiprubart was well tolerated overall. Most adverse events were mild/moderate. Empasiprubart was associated with reduced IVIg retreatment risk versus placebo in both cohorts (Figure). Improvements in patients’ self‐assessment (Table 1) and grip strength (Table 2) were greater with empasiprubart versus placebo in both cohorts. NfL levels were low and decreased with empasiprubart treatment.

**Figure:**
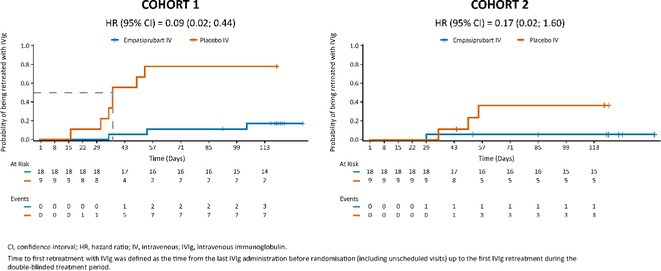
**FIGURE 1** Time to first retreatment with IVIg in cohorts 1 and 2

**Figure:**
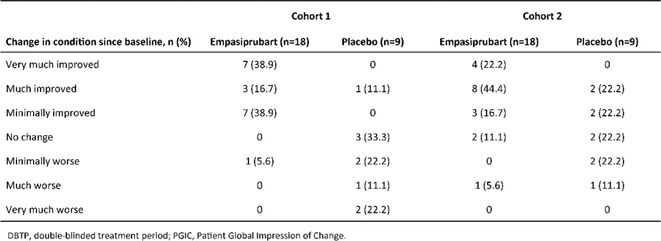
**TABLE 1** Summary of PGIC at last assessment during DBTP.

**Figure:**
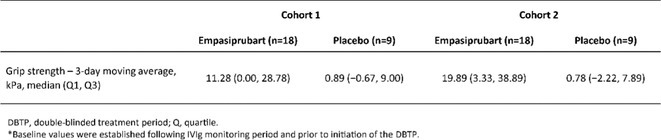
**TABLE 2** Change from baseline* in grip strength by treatment group at last assessment during DBTP.

**Conclusion:** Efficacy and safety results from the ARDA trial support proof of concept of empasiprubart in MMN and pave the way for a phase 3 trial.

**Disclosure:** LQ: Annexon, Alnylam, argenx, Avilar, Biogen, CIBERER, Fundació La Marató, CSL Behring, Dianthus, Grifols, Janssen, LFB, Lundbeck, Merck, Novartis, Octapharma, Roche, Sanofi, UCB TH: Annexon, argenx, Dianthus, Immunovant, Janssen, Nuvig, Sanofi, Takeda SP: ADOC, argenx, Berlin‐Chemie Menarini, Kedrion, Mylan, Octapharma, Pfizer, Roche, Salveo, Sanofi Genzyme, Teva Actavis, Wörwag YMH: Nothing to disclose SC: PPD, part of Thermo Fisher Scientific, argenx IVdW, EKP, MV, OVdS: employees of argenx JAA: Akcea, Alexion, Alnylam, Annexon, argenx, CSL Behring, Grifols, Immunovant, ImmuPharma, Johnson & Johnson, Pfizer, Takeda EN‐O: argenx, CSL Behring, Kedrion, LFB, Roche, Sanofi, Takeda SA: Alexion, argenx, Biogen, Janssen, LFB, Pfizer, Sanofi, UCB CK: Alexion, Alnylam, Alpine, Annexon, argenx, AstraZeneca, Biogen, Corino, CSL Behring, Genentech, Ionis, Neuroderm, Novo Nordisk, Pfizer, Sanofi, UCB, Takeda, Zai Lab HK: Alexion, argenx, CSL Behring, Grifols, Octapharma, Takeda, UCB MS: argenx, Bayer, Biogen Idec, Biotest, CSL Behring, Genzyme, Grifols, Immunovant, Kedrion, Merck, Novartis, Octapharma, PPTA, Roche, Sanofi‐Aventis, Teva, UCB SR: Annexon, argenx, CSL Behring, Dianthus, EXCEMED, Fresenius, Hansa Biopharma, Takeda, UCB WLvdP: argenx, Biogen, Biohaven, NMD Pharma, Novartis, Roche, Scholar Rock, Takeda.

## EPR‐239

### Pridopidine for the treatment of ALS: Benefits in definite, probable and early subjects in the Healey ALS platform trial

#### M. Geva^1^; J. Shefner^2^; B. Oskarsson^3^; R. Lichtenstein^4^; A. Cruz‐Herranz^1^; M. Meltzer^1^; Y. Cohen^1^; K. Chen^1^; M. Leitner^5^; S. Paganoni^6^; M. Cudkowicz^6^; M. Hayden^1^; N. Gershoni Emek ^1^

##### ^1^Prilenia Therapeutics B.V., Naarden, The Netherlands; ^2^Barrow Neurological Institute, Phoenix, USA; ^3^Department of Neurology, Mayo Clinic, Jacksonville, USA; ^4^Avram and Stella Goldstein‐Goren Department of Biotechnology Engineering, Ben‐Gurion University of the Negev, Beersheba, Israel; ^5^Accelerating NeuroVentures, LLC, Needham, USA; ^6^Sean M. Healey and AMG Center for ALS and the Neurological Clinical Research Institute, Massachusetts General Hospital, Harvard Medical School, Boston, USA

**Background and aims:** Pridopidine, a S1R agonist, demonstrates neuroprotective effects by improving cellular pathways impaired in ALS including ER stress. Pridopidine (1μM) reduced parameters of ER stress including expression of BiP (72% reduction, *p* < 0.0001) and CHOP (50% reduction, *p* < 0.0001) and increased cell viability by 69% (*p* < 0.0001). Rare mutations in S1R cause ALS. Pridopidine 45mg (bid) was evaluated in the Ph2 HEALEY ALS Platform Trial.

**Methods:** A post hoc analysis of El Escorial definite+probable ALS and early (symptom onset < 18mo) subjects in the Ph2 HEALEY ALS Platform Trial was performed. Endpoints include change from baseline to 24 weeks in ALSFRS‐R, and measures of respiration, bulbar, speech, and quality‐of‐life (QoL).

**Results:** Pridopidine was well tolerated, consistent with its prior safety profile. Pridopidine (*n* = 37) shows 32% slowing of decline versus placebo (*n* = 35) in ALSFRS‐R (wk24 Δ2.9, *p* = 0.03). Benefits are observed in ALSFRS‐R respiratory (wk24 Δ1.20, *p* = 0.03), and bulbar (wk24 Δ0.93, *p* = 0.06) domains. Pridopidine shows no worsening in dyspnea (wk24 Δ0.62, *p* = 0.04). Benefits in speaking rate (Δ0.39, *p* = 0.005) and articulation rate (Δ0.40, *p* = 0.002) are observed. A Kaplan‐Meier survival analysis shows a prolongation of median survival time (~300 to 600 days) compared to the delayed‐start (168 days) placebo‐to‐pridopidine participants (*n* = 12)(log rank *p* = 0.069). The Cox Proportional Hazard Ratio (HR), adjusted for baseline characteristics is 0.429 (*p* = 0.052).

**Conclusion:** Pridopidine demonstrated beneficial effects across multiple clinical measures of ALS, including survival benefits in definite+probable ALS and early participants. These encouraging observations support and inform planning for a Ph3 study.

**Disclosure:** Prilenia Therapeutics supported this study.

## EPR‐240

### Managing multifocal motor neuropathy: A survey of neurologists from Japan, Canada, and six European countries

#### L. Querol^1^; R. Lewis^2^; C. Sommer^3^; M. Bromberg^4^; S. Barrera‐Sierra ^5^; C. Arvin‐Berod^5^; P. Nisbet^6^; D. Gelinas^5^; J. Guptill^7^; J. Wood^5^

##### ^1^Department of Neurology, Neuromuscular Diseases Unit, Hospital de La Santa Creu I Sant Pau, Universitat Autònoma de Barcelona, Barcelona, Spain; Centro De Investigación Biomédica en Red en Enfermedades Raras (CIBERER), Madrid, Spain; ^2^Department of Neurology, Cedars‐Sinai Medical Center, Los Angeles, CA, USA; ^3^Department of Neurology, University Hospital Würzburg, Würzburg, Germany; ^4^Department of Neurology, University of Utah, Salt Lake City, UT, USA, ^5^argenx, Ghent, Belgium; ^6^One Research LLC, Mt. Pleasant, SC, USA, ^7^argenx, Ghent, Belgium; Department of Neurology, School of Medicine, Duke University, Durham, NC, USA

**Background and aims:** Multifocal motor neuropathy (MMN) is a rare, immune‐mediated, chronic neuropathy characterized by progressive, disabling, asymmetric limb weakness without sensory loss. We report results from a survey of neurologists treating MMN, aimed at understanding diagnostic and treatment patterns, perceived patient illness burden, and future treatment directions.

**Methods:** Neurologists in Japan, Canada, Denmark, Germany, Italy, the Netherlands, Spain, and the UK ≥2 years since residency who consult/treat ≥2 patients with MMN per year completed a 47‐item online survey.

**Results:** 150 neurologists completed the survey: 53% reported practicing at an academic/referral center, 24% in the community/outpatient setting, and 23% in both settings. Neurologists considered 23% of the patients they treat to have severe MMN, 43% to have moderate MMN, and 33% to have mild MMN. Use of established diagnostic guidelines was variable (Table). 82% of neurologists reported using intravenous immunoglobulin as first‐line therapy. In the previous year, neurologists switched 20% of patients to second‐line therapies, primarily due to a lack of significant improvement (39%) or disease progression (36%). The most commonly used second‐line therapies were rituximab (35%) and plasmapheresis (27%). Corticosteroids were used second‐line by 17%, despite not being recommended by guidelines. 57% of neurologists were satisfied with current treatments’ ability to improve symptoms. 45% of neurologists considered “better efficacy that improves daily functioning to perform tasks” an important attribute of potential new treatments.

**Figure:**
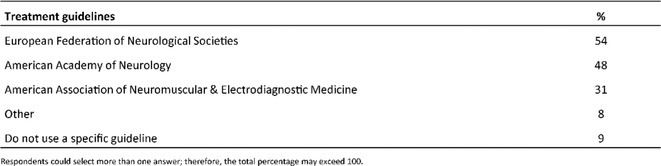
**TABLE**. Treatment guidelines and the percentage of neurologists who consider them the most useful.

**Conclusion:** This survey highlights the unmet need for greater awareness of diagnostic and treatment guidelines in MMN.

**Disclosure:** LQ: Alnylam Pharmaceuticals, Annexon Biosciences, argenx, Avilar Therapeutics, Biogen, CIBERER, CSL Behring, Dianthus Therapeutics, Fundació La Marató, GBS/CIDP Foundation International, Grifols, Instituto de Salud Carlos III – Ministry of Economy and Innovation (Spain), Janssen, LFB, Lundbeck, Merck, Novartis, Octapharma, Roche, Sanofi, UCB RAL: Alexion, Annexon Biosciences, argenx, Avilar Therapeutics, BioCryst, Boehringer Ingelheim, CSL Behring, Dianthus Therapeutics, Grifols, GBS/CIDP Foundation International, Immunovant, Intellia Therapeutics, Johnson & Johnson, Medscape, MGFA, Novartis, Nervosave Therapeutics, Nuvig Therapeutics, Sanofi, Seismic Therapeutic, Takeda, TGTX, UpToDate CS: AKIGAI, Algiax Pharmaceuticals, Alnylam Pharmaceuticals, Annexon Biosciences, argenx, CSL Behring, Grifols, GBS/CIDP Foundation International, Kedrion, Nevro, Novartis, Pfizer, Takeda, Teva Pharmaceuticals MBB: Accordant Health Care, argenx, Cambridge University Press, Oxford University Press, Takeda, UpToDate PN has nothing to disclose SB‐S, CA‐B, DG, JTG, JW are employees of argenx

## EPR‐241

### Low‐intensity pulsed ultrasound modulates disease progression in a mouse model of amyotrophic lateral sclerosis

#### X. Zhang ^1^; Z. Liu^2^; H. Zhang^2^

##### ^1^Department of Neurology, Shanghai Jiao Tong University School of Medicine Affiliated Sixth People's Hospital, Shanghai, 200233, China; ^2^Department of Ultrasonography, Shanghai Jiao Tong University School of Medicine Affiliated Sixth People's Hospital, Shanghai, China

**Background and aims:** Amyotrophic lateral sclerosis (ALS) is a devastating neurodegenerative disease characterized by the progressive loss of motor neurons in the brain and spinal cord, and there are no effective drug treatments. Low‐intensity pulsed ultrasound (LIPUS) has garnered attention as a promising noninvasive neuromodulation method. In this study, we investigate its effects on the motor cortex and underlying mechanisms using the SOD1G93A mouse model of ALS.

**Methods:** The Rotarod test and CatWalk test were used to assess the disease onset and measure gait function in different groups of mice. 9.4T MRI was used to detect cerebral blood flow in the motor cortex. In vivo multiphoton images were used to measure microvascular density in the motor cortex of mice. The change in mRNA expression after LIPUS treatment was detected by transcriptome sequencing analysis.

**Results:** Our results show that LIPUS treatment delays disease onset and prolongs lifespan in ALS mice. LIPUS significantly increases cerebral blood flow in the motor cortex by preserving vascular endothelial cell integrity and increasing microvascular density, which may be mediated via the ion channel TRPV4. RNA sequencing analysis reveals that LIPUS substantially reduces the expression of genes associated with neuroinflammation.

**Conclusion:** We demonstrate that LIPUS on the motor cortex leads to delayed disease progression in a mouse model of ALS. Enhanced cerebral blood flow, which is achieved by maintaining vascular endothelial cell integrity and increasing microvascular density, is crucial for the protective effects of LIPUS in ALS.

**Figure:**
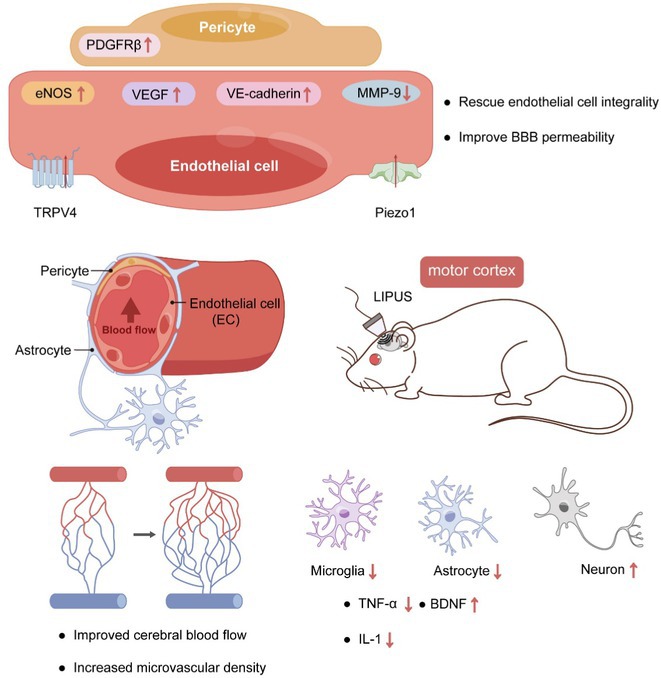
**FIGURE 1** Graphical abstract

**Disclosure:** The authors declare no competing interests.

## EPR‐242

### Lower creatinine‐to‐cystatin C ratio associated with increased risk of incident ALS in the UK Biobank Cohort

#### Z. Wang ^1^; W. Cao^1^; B. Deng^2^; D. Fan^1^

##### ^1^Department of Neurology, Peking University Third Hospital, Beijing, China; ^2^Department of Neurology, First Affiliated Hospital of Wenzhou Medical University, China.

**Background and aims:** Reduced muscle mass has been associated with the progression and prognosis of amyotrophic lateral sclerosis (ALS). However, it remains unclear whether decreased muscle mass is a risk factor for ALS or a consequence of motor neuron degeneration. Recently, serum creatinine‐to‐cystatin C ratio (CCR) have emerged as promising biomarkers for assessing muscle mass. We aimed to explore the association between CCR and the incidence of ALS using data from the UK Biobank.

**Methods:** Between 2006 and 2010, 446,945 participants were included in the baseline. CCR was calculated as the ratio of serum creatinine to cystatin C. Cox regression models were used to analyze the relationship between CCR and ALS incidence. Furthermore, subgroup analyses were conducted to investigate potential covariates in these relationships.

**Figure:**
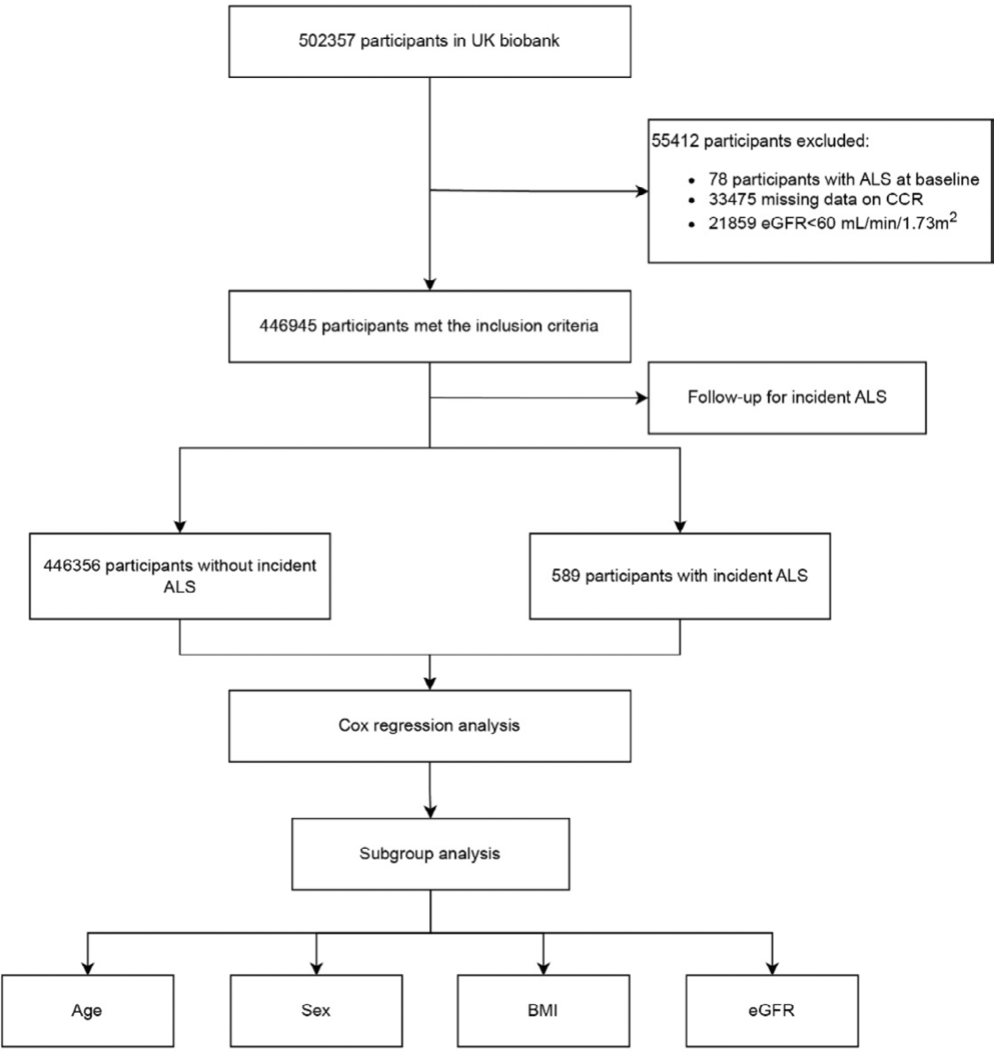
**FIGURE 1** Flowchart of the study.

**Results:** After adjusting for all covariates, the multivariate Cox regression analysis revealed a significant association between decreased CCR and an increased risk of ALS (hazard ratio (HR) = 0.990, 95% confidence interval (CI): 0.982‐0.999, *p* = 0.026). Participants were stratified into groups based on CCR tertiles. Compared with participants in the highest tertiles of CCR, those in the lowest (HR = 1.388, 95% CI: 1.032‐1.866, *p* = 0.030) and medium tertiles (HR = 1.348, 95% CI: 1.045‐1.739, *p* = 0.021) had an increased risk of ALS incidence. Subgroup analysis showed that the relationship between CCR and ALS incidence was particularly significant among participants aged < 65 years.

**Figure:**
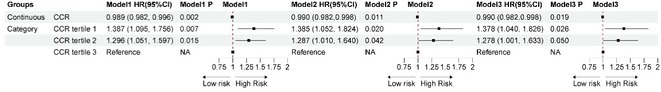
**FIGURE 2** Forest plot of the CCR on ALS incidence.

**Conclusion:** The present results demonstrate that lower CCR is significantly associated with a higher risk of ALS.

**Disclosure:** Nothing to disclose.

## Movement disorders 4

## EPR‐243

### Handwriting, touchscreen dexterity and bradykinesia measures in Parkinson's disease: A feature selection study

#### A. Gardoni^1^; E. Sarasso ^2^; S. Basaia^3^; D. Corbetta^4^; L. Zenere^3^; A. Grassi^3^; R. Balestrino^5^; E. Canu^6^; V. Castelnovo^6^; E. Sibilla^3^; D. Emedoli^4^; M. Malcangi^5^; M. Volontè^7^; F. Agosta^8^; M. Filippi^9^

##### ^1^Neuroimaging Research Unit, Division of Neuroscience, IRCCS San Raffaele Scientific Institute, and Vita‐Salute San Raffaele University, Milan, Italy; ^2^Neuroimaging Research Unit, Division of Neuroscience, IRCCS San Raffaele Scientific Institute, Vita‐Salute San Raffaele University, Milan, Italy; and DINOGMI, University of Genoa, Genoa, Italy; ^3^Neuroimaging Research Unit, Division of Neuroscience, IRCCS San Raffaele Scientific Institute, Milan, Italy; ^4^Department of Rehabilitation and Functional Recovery, IRCCS San Raffaele Scientific Institute, Milan, Italy; ^5^Neurology Unit, and Neurorehabilitation Unit, IRCCS San Raffaele Scientific Institute, Milan, Italy; ^6^Neuroimaging Research Unit, Division of Neuroscience, and Neurology Unit, IRCCS San Raffaele Scientific Institute, Milan, Italy; ^7^Neurology Unit, IRCCS San Raffaele Scientific Institute, Milan, Italy; ^8^Neuroimaging Research Unit, Division of Neuroscience, and Neurology Unit, IRCCS San Raffaele Scientific Institute, and Vita‐Salute San Raffaele University, Milan, Italy; ^9^Neurology Unit, Neurorehabilitation Unit, Neurophysiology Service, and Neuroimaging Research Unit, Division of Neuroscience, IRCCS San Raffaele Scientific Institute, and Vita‐Salute San Raffaele University, Milan, Italy

**Background and aims:** Bradykinesia affects handwriting and smartphone usage in Parkinson's disease patients (pwPD). This study aimed at assessing handwriting, hand dexterity, smartphone usage and bradykinesia in pwPD, and at identifying the features that best describe upper‐limb alterations in pwPD.

**Methods:** Forty pwPD and 30 age/sex‐matched healthy controls were included. We used standard handwriting/dexterity tests: Manual‐Ability‐Measure‐36, Purdue‐Pegboard‐Test (PPT) and copy of a text on paper. Spatiotemporal handwriting parameters were assessed using tests on a tablet: copy of text and pre‐writing tasks. To obtain objective data on movement speed and amplitude on the smartphone, we developed tests involving the most commonly used gestures (tap, swipe, slide). Bradykinesia during a finger tapping task was evaluated using electromagnetic sensors. Sequential feature selection models were used to identify the parameters best distinguishing pwPD and healthy controls.

**Results:** PwPD relative to healthy controls showed reduced manual ability and dexterity. They showed reduced movement amplitude and speed in smartphone tests and signs of micrographia during handwriting tests. Moreover, kinematic parameters correlated with both PPT and Movement Disorder Society Unified Parkinson's Disease Rating Scale III. Each feature selection model demonstrated a good accuracy, particularly when including standard handwriting/dexterity tests (R2 = 0.90), tests on smartphone (R2 = 0.94) and all the features together (R2 = 0.97). The best features were self‐reported manual abilities, PPT, tap and swipe speed/amplitude on smartphone.

**Conclusion:** This study showed that technological devices with customized software can provide quantitative measures of handwriting, dexterity and bradykinesia that will be useful to assess PD progression and the effects of interventions in pwPD.

**Disclosure:** Funding: Italian Ministry of Health, grant number GR‐2018‐12366005. Disclosures. A.G. L.Z., D.E., A.Gr., V.C., E.S., M.M., R.B. and M.A.V. Nothing to disclose. E.S., S.B., D.C., and E.C. grants form the Italian Ministry of Health. MF received compensation for consulting services or speaking activities from Alexion, Almirall, Bayer, Biogen, Celgene, Chiesi Italia SpA, Eli Lilly, Genzyme, Janssen, Merck‐Serono, Neopharmed Gentili, Novartis, Novo Nordisk, Roche, Sanofi Takeda, and TEVA; Advisory Boards for Alexion, Biogen, Bristol‐Myers Squibb, Merck, Novartis, Roche, Sanofi, Sanofi‐Aventis, Sanofi‐Genzyme, Takeda; scientific direction of educational events for Biogen, Merck, Roche, Celgene, Bristol‐Myers Squibb, Lilly, Novartis, Sanofi‐Genzyme; he receives research support from Biogen Idec, Merck‐Serono, Novartis, Roche, the Italian Ministry of Health, the Italian Ministry of University and Research, and FISM. F.A. is Associate Editor of NeuroImage: Clinical, has received speaker honoraria from Biogen Idec, Italfarmaco, Roche, Zambon and Eli Lilly, and receives or has received research supports from the Italian Ministry of Health, the Italian Ministry of University and Research, AriSLA (Fondazione Italiana di Ricerca per la SLA), the European Research Council, the EU Joint Programme—Neurodegenerative Disease Research (JPND) and Foundation Research on Alzheimer Disease (France).

## EPR‐244

### Diabetes impact on nigrostriatal vulnerability in Parkinson's Disease

#### A. Pilotto ^1^; A. Galli^1^; C. Zatti^1^; S. Caminiti^2^; E. Premi^3^; F. Bertaga^1^; D. Perani^4^; A. Padovani^1^

##### ^1^University of Brescia, Italy; ^2^University of Pavia, Italy, ^3^stroke unit asst spedali civili of Brescia, Italy; ^4^San Raffaele University, Milan, Italy

**Background and aims:** Less is known about mechanisms underlying the role of diabetes mellitus (DM) as modulator of clinical severity in Parkinson's Disease (PD).

**Methods:** Patients with and without diabetes were first contrasted and secondarily matched for age, sex, MDS‐UPDRS‐I and III. Regional differences in 123I‐FP‐CIT binding within nigrostriatal pathways in PD with and without diabetes were addressed via ROI‐based and voxel‐wise univariate analyses. Alterations in molecular connectivity between the groups were assessed via correlation analysis.

**Results:** 269 drug‐naïve PD patients were enrolled (*n* = 174 patients from PPMI; *n* = 95 patients from DNA). In both cohorts, patients with diabetes (PD‐DM) were older, exhibited a higher male prevalence, and exhibited worse non‐motor and cognitive symptoms than PD without (PD‐n). After the severity‐matching procedure, PD‐DM exhibited higher dopamine binding in left putamen compared to PD in both independent cohorts. PD‐DM showed significant dopaminergic connectivity alterations within nigrostriatal nodes (20%), primarily due to a loss of connectivity (98%). PD‐n showed a higher percentage of connectivity alterations within nigrostriatal nodes (33%), characterized by both loss (84%) and gained connections (16%).

**Conclusion:** This is the first study demonstrating the impact of diabetes on striatal dopaminergic motor reserve, amplifying the effect of dopaminergic loss on motor symptoms.

**Disclosure:** none

## EPR‐245

### ADL impairments in prodromal Parkinson's: Impact of RBD, hyposmia, and combined phenotypes

#### C. Stewart ^1^; S. Sathyanarayana^1^; V. Foster^1^; R. Iredale^1^; D. Galley^1^; J. Pasquini^2^; K. Anderson^3^; N. Pavese^1^; D. Ledingham^1^

##### ^1^Clinical Ageing Research Unit, Newcastle University, Campus for Ageing and Vitality, Newcastle upon Tyne, UK; ^2^Department of Clinical and Experimental Medicine, University of Pisa, Pisa, Italy; ^3^Regional Sleep Service, Newcastle upon Tyne, NHS Foundation Trust, Newcastle upon Tyne, UK

**Background and aims:** Activities of daily living (ADL) impairments are important markers of Parkinson's disease (PD) progression. However, limited research exists on ADL impairments in individuals with prodromal symptoms, such as rapid eye movement sleep behavior disorder (RBD) and hyposmia, who have not been diagnosed with PD. This study aimed to evaluate ADL impairments in prodromal subgroups—RBD, hyposmia, and combined RBD and hyposmia (RBD+H) to assess whether these phenotypes are associated with worse ADL function.

**Methods:** Data from 245 healthy controls (HC), 74 RBD, 491 hyposmia, and 476 RBD+H participants in the Parkinson's Progression Markers Initiative (PPMI) were analyzed. RBD was confirmed via polysomnography, while hyposmia was defined as an UPSIT score < = 15th percentile without RBD symptoms. ADL impairments were assessed using the MDS‐UPDRS Parts I and II and Schwab and England scales. Statistical tests included Kruskal‐Wallis and generalized linear modeling (GLM), adjusted for age and sex, with Bonferroni‐corrected post‐hoc analyses.

**Results:** Subgroup membership significantly predicted ADL impairments (Chi‐square = 240.519, *p* < 0.001). Estimated means indicated higher ADL impairments in pure RBD (10.38) and RBD+H (7.88), compared to hyposmia (4.87) and HCs (3.04). RBD participants reported higher impairments due to anxiety (*p* < 0.001), and RBD+H participants had more impairments related to constipation compared to hyposmia (*p* = 0.002).

**Conclusion:** Subgroup membership is a strong predictor of ADL impairments, emphasizing the need to address these challenges in prodromal PD, for early intervention and improved disease staging. Furthermore, this research highlights the importance of further investigating prodromal phenotypes to better understand PD progression.

**Disclosure:** No specific funding was received for this study. All authors are involved in the recruitment and/or conduct of the PPMI study at the Newcastle UK study site.

## EPR‐246

### Treatment stability comparison among different ADD‐ON therapies in fluctuating Parkinson's disease patients

#### D. Rinaldi ^1^; S. Galli^1^; G. Imbalzano^2^; S. Budicin^2^; E. Bianchini^1^; L. De Carolis^1^; M. Alborghetti^1^; M. Zibetti^2^; L. Lopiano^2^; F. Pontieri^1^; C. Artusi^2^

##### ^1^Dipartimento di Neuroscienze, Salute Mentale e Organi di Senso, Sapienza Università di Roma, Via di Grottarossa, 1035‐00189, Roma, Italy; ^2^Department of Neuroscience “Rita Levi Montalcini”, University of Turin, Turin, Italy

**Background and aims:** Motor fluctuations are a major challenge in advanced Parkinson's disease (PD), often managed using add‐on therapies such as catechol‐O‐methyl transferase (COMT) or monoamine oxidase‐B (MAO‐B) inhibitors. However, real‐life comparisons between these therapies are limited. This study investigates the efficacy and tolerability of selegiline (SL), rasagiline (RS), safinamide (SF), and opicapone (OP) in fluctuating PD patients, focusing on treatment stability and demographic influences.

**Methods:** This retrospective longitudinal study included 160 fluctuating PD patients treated at two Italian tertiary centers (2012–2023). Inclusion criteria required motor fluctuations (WOQ‐19 ≥2) and at least 12 months of follow‐up. Patients were grouped by add‐on therapy (SL, RS, SF, OP). The primary outcome was the stability of antiparkinsonian therapy, defined as months without significant modifications in treatment or adverse events (AEs). Demographic and clinical factors were analyzed using Cox regression.

**Results:** The OP group had the longest disease duration (9.8 ± 4.6 years, *p* = 0.003) and the highest baseline LEDD (*p* = 0.022). Stability did not differ significantly between groups (*p* = 0.167). However, females exhibited higher therapy modification rates (*p* = 0.013). AEs occurred in 15% of patients, predominantly dyskinesia (6.9%) and hallucinations (5%). OP was more frequently prescribed to younger patients (64.3 ± 7 years).

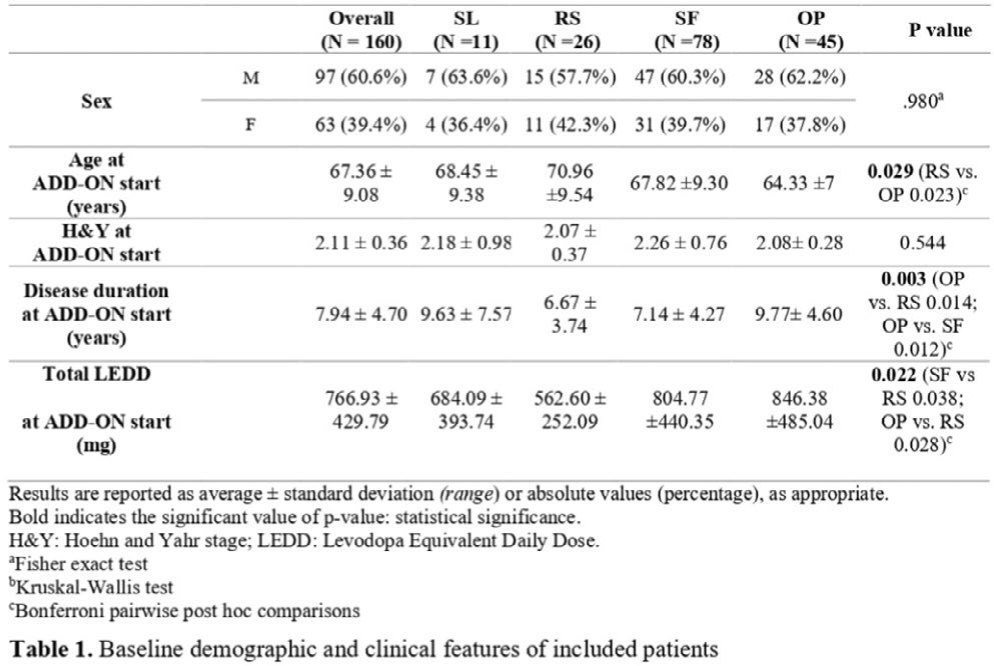

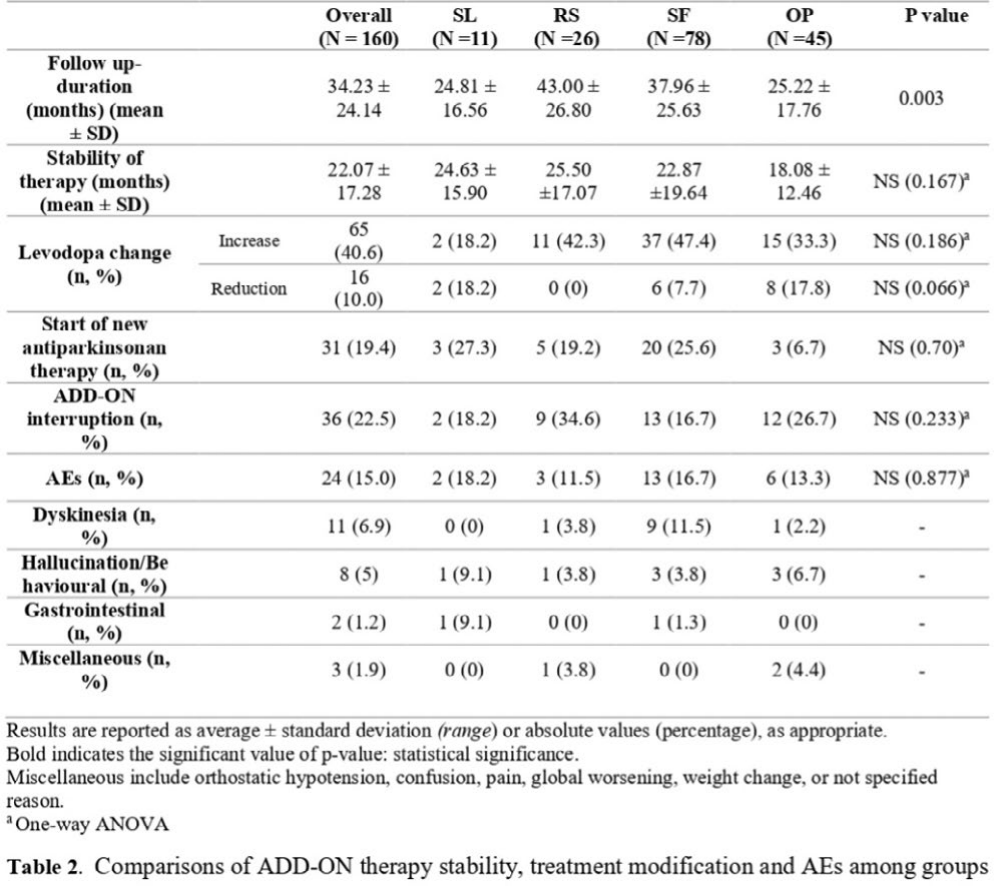

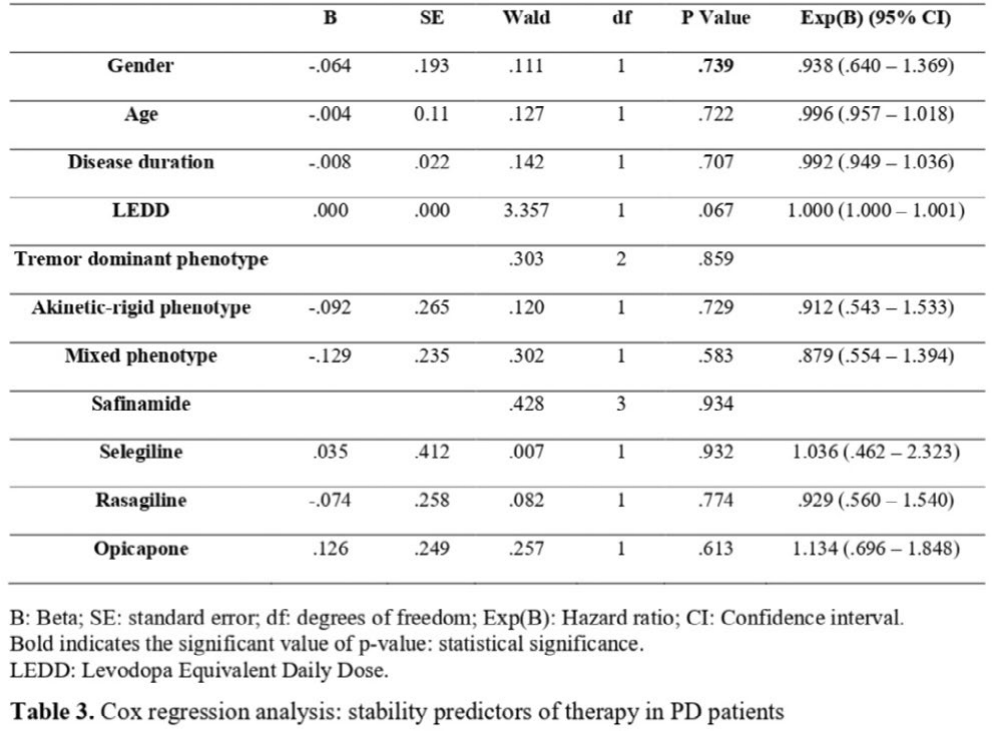

**Conclusion:** No single add‐on therapy demonstrated superior stability, highlighting comparable efficacy across options. Sex significantly influenced therapy adjustments, with females requiring more frequent modifications. These findings underscore the importance of personalized treatment strategies in fluctuating PD management.

**Disclosure:** Nothing to declare.

## EPR‐247

### Theta‐gamma frequency subthalamic stimulation for verbal fluency in Parkinson's Disease: A randomized, crossover trial

#### G. Imbalzano ^1^; E. Montanaro^2^; C. Ledda^1^; F. Donetto^3^; F. D'Angelo^4^; C. Campisi^1^; C. Artusi^1^; A. Romagnolo^1^; M. Rizzone^1^; M. Bozzali^1^; L. Lopiano^1^; M. Zibetti^1^

##### ^1^Department of Neuroscience “Rita Levi Montalcini”, University of Torino, Torino, Italy; ^2^Clinical Psychology Unit, AOU Città della Salute e della Scienza, Torino, Italy; ^3^SC Neurologia 2U, AOU Città della Salute e della Scienza, Torino, Italy; ^4^Department of Psychology, University of Torino, Torino, Italy

**Background and aims:** High‐frequency Deep Brain Stimulation (DBS) of the subthalamic nucleus (STN) improves motor symptoms in Parkinson's disease (PD), but may affect cognition, especially verbal fluency (VF). Low‐frequency stimulation (theta, 4‐10 Hz) showed potential cognitive benefits but can worsen motor symptoms in PD. This randomized, double‐blind, cross‐over study evaluated the safety and efficacy of combined theta‐gamma frequency stimulation on VF in PD patients with STN‐DBS.

**Methods:** Patients were randomly assigned 1:1 to start with either standard or theta‐gamma STN stimulation, followed by the reverse. VF was assessed at baseline, one hour, and one month after each stimulation change. Secondary endpoints included adverse events (AEs), motor and non‐motor symptoms (including mood and impulsivity) and their complications. Data were analyzed using a linear mixed‐effects model, considering fixed effects for visit time, stimulation setting, and their interaction.

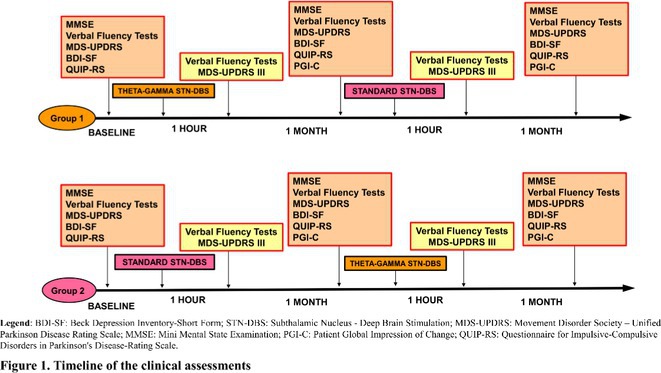

**Results:** Twelve patients completed the study. No significant effects of the stimulation settings were observed one hour after stimulation change. After one month, theta‐gamma stimulation significantly improved non‐episodic category VF (*p* = 0.037) and episodic category VF (*p* = 0.034) over standard stimulation. No significant differences were found in phonemic fluency or category switching. Motor and non‐motor outcomes were not significantly affected by the stimulation setting. AEs were mild and evenly distributed between conditions.

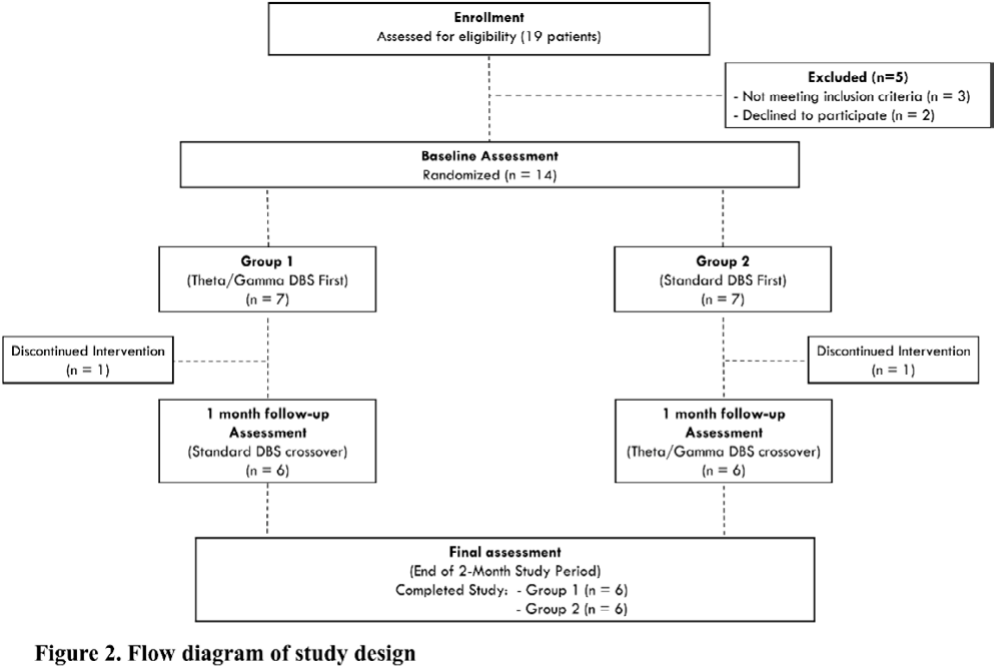

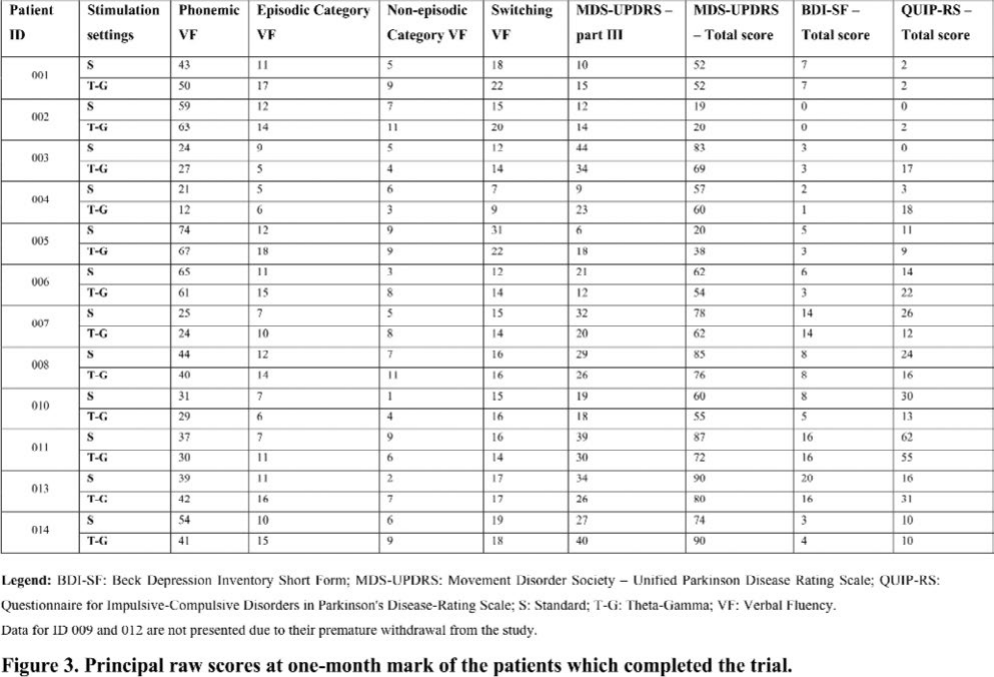

**Conclusion:** Combined theta‐gamma frequency stimulation may improve VF in PD patients treated with STN‐DBS safely and without worsening motor symptoms. This approach could be integrated into existing stimulation paradigms, though further studies are needed to confirm these findings and explore broader clinical implications.

**Disclosure:** Nothing to disclose.

## EPR‐248

### GLP‐1 receptor agonist: a new disease‐modifying therapy in Parkinson's disease? A systematic review and meta‐analysis

#### G. Siconolfi

##### Department of Neuroscience, Catholic University of the Sacred Heart, Rome, Italy

**Background and aims:** Glucagon‐like peptide‐1 receptor (GLP‐1R) agonists, primarily used for treating diabetes, have recently demonstrated neuroprotective properties in a mouse model for Parkinson's disease (PD). Despite this, their efficacy as a potential disease‐modifying therapy for PD remains controversial. Therefore, we conducted a meta‐analysis to evaluate the impact of GLP‐1R agonists on slowing the progression of motor and non‐motor symptoms in PD patients.

**Methods:** We performed a systematic review and meta‐analysis of randomized clinical trials (RCTs) comparing GLP‐1R agonists to placebo or best medical therapy (BMT) in patients with mild to moderate PD. We searched in Scopus, Cochraine and PubMed. The primary goal was to assess the efficacy of GLP‐1R agonists in reducing motor disability progression valuing the change in MDS‐UPDRS part III scores after a washout period from PD therapy

**Results:** Four RCTs with a total of 515 patients were included. Among these, 300 (58%) received GLP‐1R agonists, and 215 (42%) received a placebo or BMT. GLP‐1R agonists were associated with significantly slower progression of motor symptoms, as indicated by changes from baseline in the MDS‐UPDRS part III score (MD ‐2.08, 95% CI ‐3,87 to ‐0,28, *p* = 0.02, I^2 = 30%

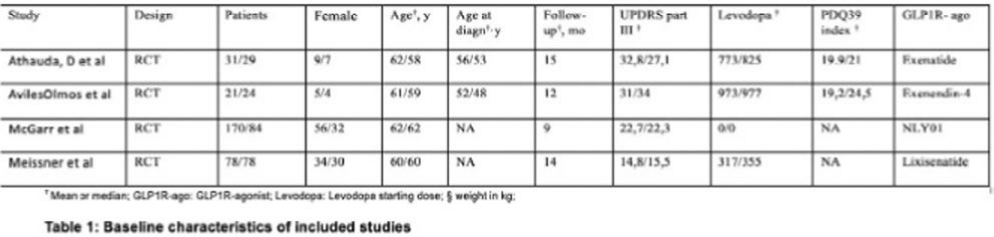

**Figure:**
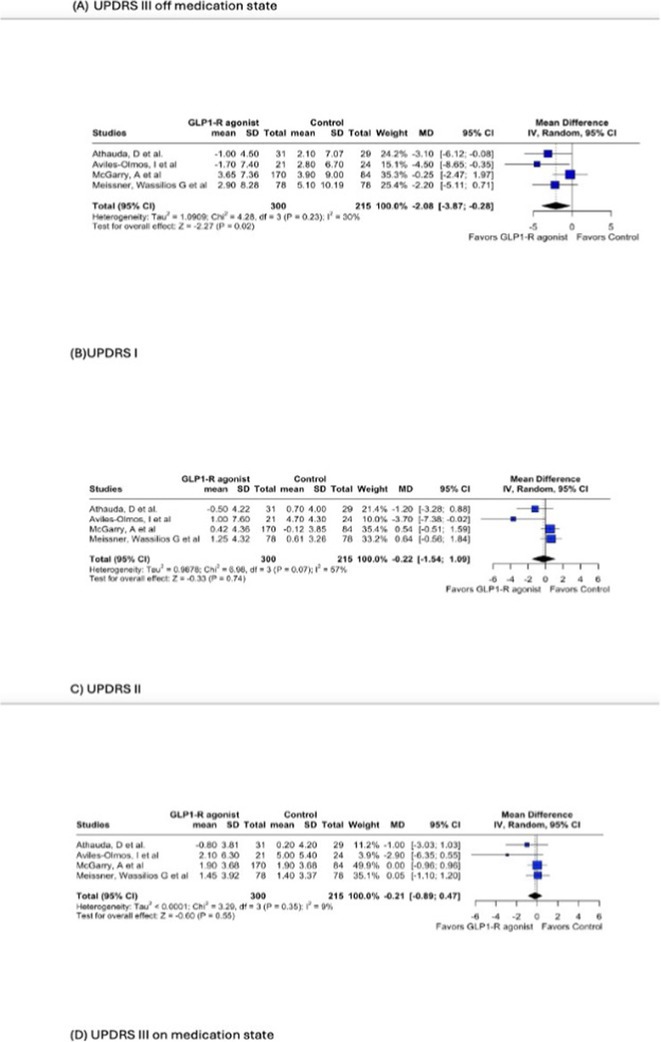
**FIGURE 1** Meta‐analysis (A) UPDRS III off medication state (B) UPDRS I on medication state (C) UPDRS II

**Figure:**
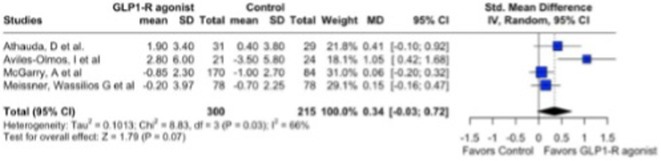
**FIGURE 2** Cognitive functions assessed by standardized mean difference of Moca and MDRS‐2 from baseline

**Conclusion:** This meta‐analysis, encompassing 515 patients from four RCTs, demonstrated that GLP‐1R agonists are associated with a slower deterioration of motor symptoms and cognitive functions in patients with mild to moderate Parkinson's disease.

**Disclosure:** Nothing to disclose.

## EPR‐249

### Advancing Deep Brain Stimulation programming in Parkinson's disease: Automated algorithms for electrode selection

#### I. D'Ascanio ^1^; I. Cani^2^; L. Baldelli^2^; A. Conti^2^; G. Lopane^3^; P. Mantovani^4^; M. Pegoli^5^; P. Cortelli^2^; L. Chiari^1^; C. Giovanna^2^; L. Palmerini^1^; G. Giannini^2^

##### ^1^Department of Electrical, Electronic, and Information Engineering, Alma Mater Studiorum – University of Bologna, Bologna, Italy; ^2^UOC Clinica Neurologica Rete Metropolitana NEUROMET, IRCCS Istituto delle Scienze Neurologiche di Bologna, Bologna, Italy; ^3^Unit of Rehabilitation Medicine, IRCCS Istituto delle Scienze Neurologiche di Bologna, Bologna, Italy; ^4^Unit of Neurosurgery, IRCCS Istituto delle Scienze Neurologiche di Bologna, Bologna, Italy; ^5^Anesthesia and Neurointensive Care Unit, IRCCS Istituto delle Scienze Neurologiche di Bologna, Bologna, Italy

**Background and aims:** The programming of stimulation settings is a critical aspect in the postoperative management of individuals with Parkinson's disease undergoing deep brain stimulation [1]. However, the process of identifying effective stimulation contacts and optimizing stimulation parameters is not standardized yet, resulting in a time‐consuming and resource‐intensive task. This study aimed to i) evaluate state‐of‐the‐art methods for electrode selection and ii) to validate a novel automated

**Methods:** This retrospective analysis includes 16 individuals who were consecutively entered in the One Hospital ClinicalService Brain Modulation Project and underwent implantation of a PerceptTM PC (Medtronic) neurostimulator. A comparative analysis was conducted on three methods for beta peak selection and stimulation electrode suggestion. The methods differ in their definition of the beta band, the cut‐offs considered for peak amplitude, and the criteria chosen for electrode selection. Two Medtronic algorithms (the device algorithm and a novel one) and an existing method [2] were analyzed. The electrode detected by the algorithms was then compared with the final programming decision made by clinicians.

**Results:** Data from both hemispheres were available in 29 out of 36 recording sessions (81%). The device current and novel algorithm match the final programming decision on the stimulation electrode in 63.2% and 67.9% of cases, respectively. In contrast, the algorithm developed by Strelow et al. [2] matches in only 2 cases (7.1%).

**Conclusion:** This study highlights the potential of automated algorithms in supporting the selection of stimulation electrodes, thereby reducing the complexity of DBS programming and enhancing clinical efficiency, while ensuring concordance with clinician‐guided programming decisions.

**Disclosure:** Nothing to disclose.

## EPR‐250

### CSF tau protein levels as a predictor of motor fluctuation risk in de novo Parkinson's disease patients

#### J. Bissacco ^1^; R. Bovenzi^1^; M. Conti^1^; C. Simonetta^1^; D. Mascioli^1^; M. Mancini^1^; V. Buttarazzi^1^; R. Cerroni^1^; M. Pierantozzi^1^; A. Stefani^2^; N. Mercuri^1^; T. Schirinzi^1^

##### ^1^Department of Systems Medicine, University of Rome “Tor Vergata”, Rome, Italy.; ^2^UOSD Parkinson Centre, Tor Vergata University Hospital, Rome, Italy.

**Background and aims:** Motor fluctuations (MF) are a disabling complication of Parkinson's disease (PD). While multiple factors contribute to MF onset, the role of brain co‐pathology, including tauopathy and amyloidopathy, remains unclear. The objective of this study was, therefore, to explore the association between CSF co‐pathology profiles and MF development in a longitudinal de novo (DN) PD cohort.

**Methods:** We conducted a single‐center retrospective study with 108 DN PD patients, assessed by the MDS‐UPDRS and MoCA scores, and the measurement of CSF total alpha‐synuclein (alpha‐syn), total and phosphorylated‐181 tau (t‐tau, p‐tau), amyloid‐beta42 and amyloid‐beta40 (ABeta42, ABeta40) levels, p‐tau/t‐tau, ABeta42/ABeta40, and p‐tau/ABeta42 ratios. Patients were classified as “fluctuator” (FLUCT) or “no‐fluctuator” (NoFLUCT) based on MF development (MDS‐UPDRS part IV > = 1) during follow‐up. Baseline variables were compared; ROC and Cox regression analyses were run to estimate their predictive values.

**Results:** The DN PD cohort was followed for 5 ( ± 1.45) years, with 32 (29.6%) patients developing MF. At baseline, patients showed lower CSF alpha‐syn and t‐tau levels than controls, while FLUCT had higher p‐tau, p‐tau/t‐tau, and p‐tau/ABeta42 ratios. The p‐tau/t‐tau ratio best predicted MF development; above the cutoff of 0.135, MF were 5 times more likely with 87.1% sensitivity and 63.5% specificity (AUC = 0.81).

**Conclusion:** Elevated CSF p‐tau/t‐tau ratios in DN PD patients indicate a higher risk of MF. These findings suggest that tau and, to a lesser extent, amyloid‐beta, co‐pathology might be involved in the mechanisms leading to MF onset, supporting lines of evidence that show their contribution to the degeneration of motor circuits in PD.

**Disclosure:** The research leading to these results has received funding from the European Union (NextGenerationEU) through the Italian Ministry of University and Research under PNRR–M4C2‐I1.3 Project PE_00000019 “Heal Italia” to T.S. (CUP E83C22004670001). The views and opinions expressed are those of the authors only and do not necessarily reflect those of the European Union or the European Commission. The authors have no conflicts of interest to declare.

## EPR‐251

### Mobility in people with Parkinson's disease with and without nocturnal hypokinesia: A propensity score‐matched study

#### P. Pacilio ^1^; E. Bianchini^1^; S. Galli^1^; L. De Carolis^1^; C. Hansen^2^; M. Alborghetti^1^; T. Milane^3^; F. Pontieri^1^; D. Rinaldi^1^; N. Vuillerme^3^

##### ^1^Department of Neuroscience, Mental Health and Sensory Organs (NESMOS), Sapienza University of Rome, Rome, Italy; ^2^Department of Neurology, Kiel University, Kiel, Germany; ^3^Univ. Grenoble Alpes, AGEIS, Grenoble, France.

**Background and aims:** Mobility impairment and nocturnal hypokinesia (NH) are common in people with Parkinson's disease (PwPD) and significantly impact quality of life. Reduced hip muscle strength/torque could contribute to NH and link it to mobility problems. However, no studies investigated the relationship between these two aspects in PwPD.

**Methods:** Data from 30 PwPD with NH [females: 11 (37%); age: 69.8 ± 7.8; disease duration: 6.4 ± 3.3] and 60 without NH, propensity score‐matched for age, sex and disease duration were included. NH was defined as MDS‐UPDRS‐II‐2.9 ≥1. Participants performed three supervised mobility tasks with a lower‐back‐mounted inertial sensor (BTS G‐WALK): a 25‐meter forward walking at self‐selected speed (FW), a Timed‐Up‐and‐Go (TUG) and 3‐meter backward walking test (3MBWT). Spatiotemporal gait parameters, TUG duration, mean (MAV) and peak angular velocity (PAV) of TUG 180° turning and 3MBWT speed were measured. Participants wore a Garmin Vivosmart 4 smartwatch for 5 consecutive days on the least affected wrist and average daily steps (avDS) were calculated.

**Results:** PwPD with NH showed a lower gait speed (*p* = 0.024), normalized stride length (*p* = 0.036), TUG duration (*p* = 0.033), MAV (*p* = 0.011), PAV (*p* = 0.009), 3MBWT speed (*p* = 0.022) and avDS (*p* = 0.011). Moreover, PwPD with NH showed a higher prevalence of falls (*p* = 0.001)

**Conclusion:** NH was associated with worse supervised and real‐world mobility as well as a higher prevalence of falls. This could suggest a shared mechanism between NH and mobility impairments as part of a more severe disease phenotype. Our results highlight the need to investigate NH and develop therapeutical strategies for this symptom.

**Disclosure:** Nothing to disclose.

## Movement disorders 5

## EPR‐252

### Plasma pTau217 detection for Alzheimer's Disease co‐pathology in Parkinson's Disease and Parkinsonism

#### E. Fiorenzato^1^; G. Musso^3^; F. Vianello^1^; S. Cauzzo^1^; R. Biundo^4^; V. Misenti^1^; G. Bonato^1^; M. Campagnolo^1^; M. Carecchio^1^; A. Guerra^1^; V. D'Onofrio^1^; A. Cagnin^2^; M. Montagnana^3^; A. Antonini ^1^

##### ^1^Parkinson and Movement Disorders Unit, Study Center for Neurodegeneration (CESNE), Department of Neuroscience, University of Padova, Padova, Italy.; ^2^Padova Neuroscience Center (PNC), University of Padova, Padova, Italy.; ^3^Department of Medicine – DIMED, University of Padova, Italy.; ^4^Complex Operative Unit (UOC) of the Psychology, Neurology Hospital division, Padova University Hospital, Padova, Italy; Department of General Psychology, University of Padova, Padova, Italy.

**Background and aims:** Alzheimer's disease (AD) co‐pathology is common in Parkinson's spectrum disorders and independently contributes to dementia. Plasma pTau217 has emerged as a promising blood‐based biomarker for detecting AD pathology, correlating with amyloid and tau PET, and may help to identify AD co‐pathology in movement disorders. We evaluated plasma pTau217 for detecting AD co‐pathology in PD and parkinsonism and its association with cognitive impairment.

**Methods:** We included 170 participants from PADUA‐CESNE cohort: 57 PD, 4 dementia with Lewy bodies (DLB), 28 Progressive Supranuclear Palsy (PSP), 4 corticobasal syndrome (CBS), 51 healthy aged controls (HC), 26 mild cognitive impairment (MCI). All participants underwent an extensive cognitive assessment, including MoCA and MMSE, and were classified accordingly (PD‐cognitive spectrum: PD‐NC normal cognition, PD‐MCI, PDD dementia). Plasma pTau217 was measured through Lumipulse G1200; amyloid positivity was defined as pTau217 > 0.22 ng/L

**Results:** Among PD spectrum, PD‐NC showed lower levels of pTau217 than PD‐MCI (*p* = 0.042), PDD/DLB (*p* = 0.049), and PSP/CBS (*p* = 0.002). No pTau217‐positive cases were observed in PD‐NC. Amyloid positivity was highest in the MCI group (32.14%), followed by CBS/PSP (28.13%), PDD/DLB (27.27%), and PD‐MCI (15.63%), with the lowest in HC (7.84%). Negative correlations were found between pTau217 and MoCA (r^2^ = –0.38, *p* = 0.004) and MMSE (r^2^ = –0.37, *p* = 0.006).

**Conclusion:** pTau217 is elevated in PD patients with cognitive impairment, particularly PDD/DLB, and in patients with atypical parkinsonism such as PSP and CBS, but not in cognitively normal PD. It may serve as a reliable marker of AD co‐pathology and cognitive involvement, warranting further validation with PET imaging.

**Disclosure:** Nothing to disclose.

## EPR‐253

### Nocturnal heart rate variability in iRBD

#### I. Filchenko; L. Serrano Lopes; J. van der Meer; C. Bassetti; C. Schäfer

##### Department of Neurology, University Hospital, Inselspital, Bern, Switzerland

**Background and aims:** Idiopathic REM sleep behavior disorder (iRBD) is a prodromal marker of neurodegenerative diseases, often preceding their clinical diagnosis by years. Autonomic dysfunction, a hallmark of early neurodegeneration, is a clinical feature of iRBD. We investigated the autonomic function during sleep in iRBD by analyzing nocturnal heart rate variability (HRV).

**Methods:** In this case‐control study, patients with iRBD were matched 1:6 for age, sex, and apnea‐hypopnea index with non‐iRBD controls without known brain damage (e.g., stroke) from the Bern Sleep‐Wake Registry (*n*≈11000). All participants underwent clinical polysomnography. iRBD patients were followed for the development of an overt alpha‐synucleinopathy such as Parkinson's Disease (PD). Twenty nocturnal HRV parameters, including time‐domain, frequency‐domain, and nonlinear measures, were derived from routine clinical polysomnography. Group differences in nocturnal HRV were analyzed using the Wilcoxon rank‐sum test.

**Results:** Seventeen patients with iRBD and 102 controls were included in the analysis. Patients with iRBD showed low HRV, with low absolute power in both low‐ and high‐frequency components, indicating impaired autonomic modulation during sleep. Three out of seventeen iRBD patients subsequently developed PD (follow‐up: 132 person‐years). In an exploratory analysis, the patients, who progressed to PD, had higher nocturnal heart rate, lower HRV, and higher sympathetic dominance compared to those, who did not.

**Figure:**
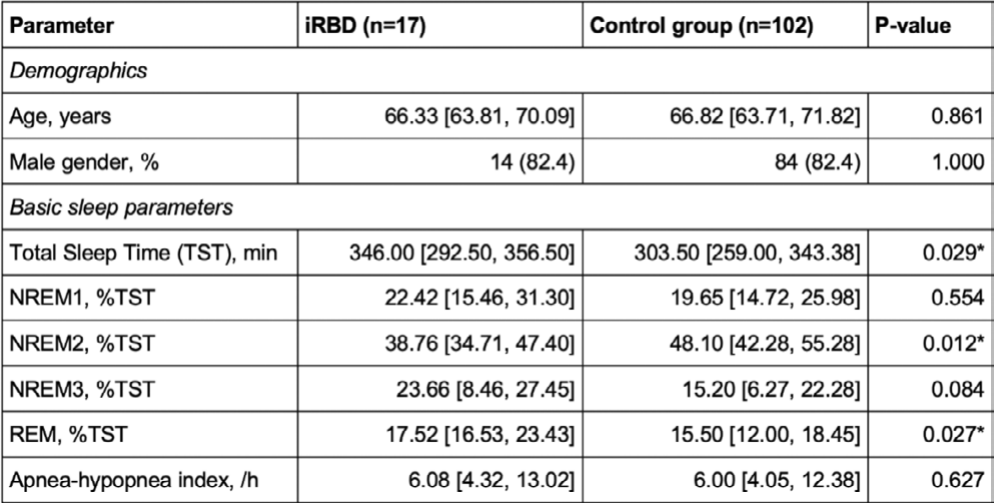
**TABLE 1** Study population: iRBD versus control group.

**Figure:**
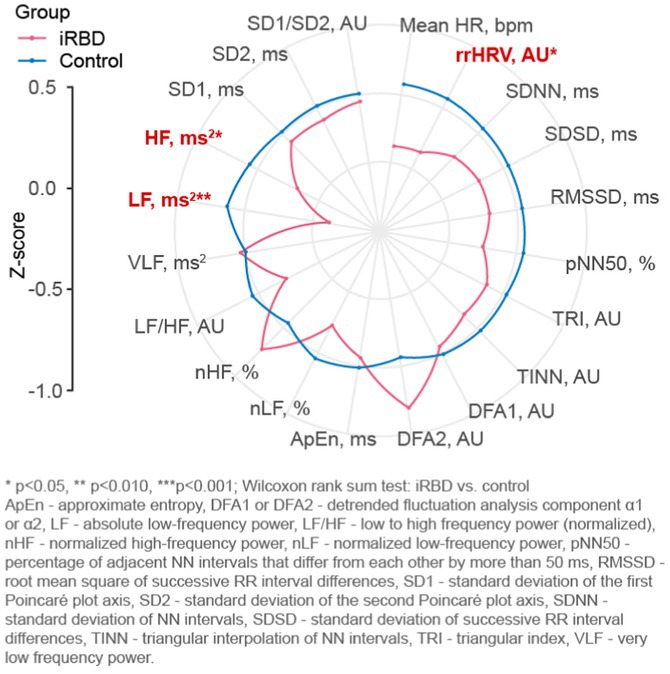
**FIGURE 1** Circular plot of the differences in nocturnal HRV between iRBD patients and the control group.

**Figure:**
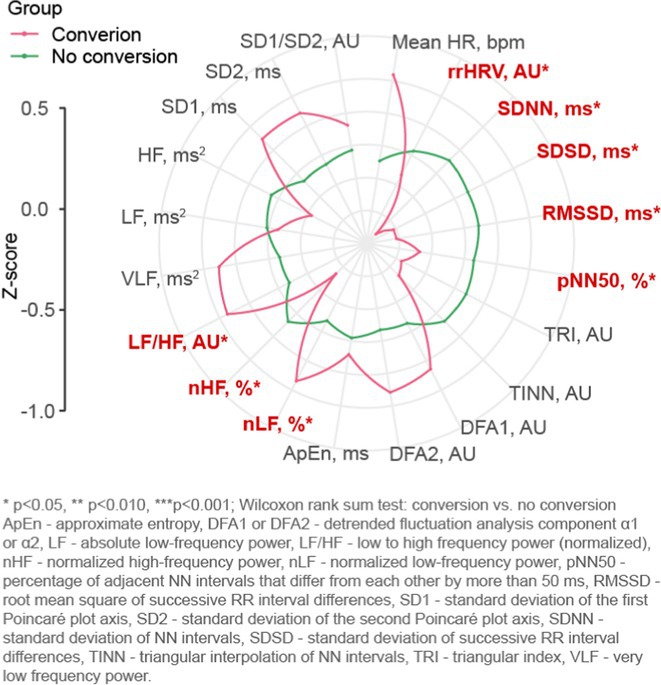
**FIGURE 2** Circular plot of the differences in nocturnal HRV between iRBD patients depending on the conversion to Parkinson's disease.

**Conclusion:** Low nocturnal HRV is a potential marker of autonomic dysfunction in iRBD. Sympathetic hyperactivation may signal the approaching conversion of iRBD to PD and might serve as a marker for neurodegenerative progression in iRBD.

**Disclosure:** Nothing to disclose.

## EPR‐254

### Biomarkers in early‐stage Huntington's disease

#### C. Cerejo ^1^; E. Mandler^1^; F. Carbone^1^; G. Bsteh^2^; B. Teuchner^3^; K. Schwarzová^1^; M. Peball^1^; A. Djamshidian^1^; K. Seppi^4^; B. Heim^1^

##### ^1^Department of Neurology, Medical University of Innsbruck, Innsbruck, Austria; ^2^Department of Neurology, Medical University of Vienna, Vienna, Austria and Comprehensive Center for Clinical Neurosciences and Mental Health, Medical University of Vienna, Vienna, Austria; ^3^Department of Ophthalmology and Optometry, Medical University of Innsbruck, Austria; ^4^Department of Neurology, Medical University of Innsbruck, Innsbruck, Austria and Department of Neurology, Hospital Kufstein, Austria

**Background and aims:** Huntington's disease (HD) is a neurodegenerative disorder characterized by motor, cognitive, and behavioral abnormalities. Optical Coherence Tomography (OCT) and olfactory testing offers a non‐invasive method to measure retinal changes and olfactory dysfunction, respectively, that reflect neurodegenerative processes.

**Methods:** This cross‐sectional study compared spectral domain OCT data, and olfactory testing using the Sniffin’ Sticks battery for identification and discrimination in HD patients and healthy controls (HC). HD patients were classified into Stage1 and Stage2 based on motor symptoms and functional capacity.

**Results:** We recruited a total of 68 participants including 39HD patients (22 stage1, 17 stage2) and 29 age‐matched HC. There were no significant differences in age and gender between the groups. Stage2 HD patients showed worse motor function (UHDRS‐TMS 28.44 ± 18.13 vs. 13.74 ± 8.78, *p* = 0.002), functional capacity (UHDRS‐TFC 8.13 ± 2.03 vs. 12.44 ± 0.99, *p* < 0.001), and lower scores on MMSE (27.36 ± 1.64 vs. 28.73 ± 1.74, *p* = 0.005 vs. 29.45 ± 0.91, *p* < 0.001) compared to stage1 HD patients and HC, respectively. Both stage1 and stage2 HD groups displayed significantly reduced macular retinal nerve fiber layer thickness (mRNFL) (33.45 ± 4.70, 31.90 ± 3.47 vs. 38.45 ± 5.00; *p* < 0.01) and ganglion cell‐inner plexiform layer thickness (GCIPL) (71.63 ± 6.38, 60.42 ± 4.67 vs. 77.03 ± 8.40; *p* < 0.01) as compared to HC. Odor identification and discrimination were significantly reduced in both stage1(10.87 ± 3.11, 9.62 ± 2.99 respectively; *p* < 0.001) and stage2(9.88 ± 2.90, 7.38 ± 3.89 respectively; *p* < 0.001) HD patients as compared to HC.

**Conclusion:** In this study, HD patients showed significantly thinner GCIPL and mRNFL, and olfactory dysfunction compared to healthy controls, even in early disease stages. These findings suggest that OCT and olfactory testing may serve as a valuable biomarker especially in the early stages of HD.

**Disclosure:** Nothing to disclose.

## EPR‐255

### Region‐specific treatment preferences in advanced Parkinson's disease: A discrete choice experiment

#### I. A. Malaty^1^; J. Domingos^2^; R. Pahwa^3^; K. Ray Chaudhuri^4^; A. Antonini^5^; F. De Renzis^6^; P. Arija^7^; M. Heisen^8^; H. Penton^7^; C. H. Yan ^9^; E. Shirneshan^9^; M. Shah^9^; P. Kukreja^9^; J. Carlos Parra^9^; M. Boeri^10^

##### ^1^University of Florida, Fixel Institute for Neurological Diseases, Gainesville, FL, USA; ^2^Parkinson's Europe, Orpington, UK; Egas Moniz School of Health & Science, Almada, Portugal; ^3^University of Kansas Medical Center, Kansas City, KS, USA; ^4^King's College and Parkinson Foundation Centre of Excellence, Kings College Hospital London and Kings College, London, UK; ^5^Parkinson and Movement Disorders Unit, Study Centre for Neurodegeneration, Department of Neuroscience, University of Padova, Padova, Italy; ^6^Parkinson's Europe, Orpington, UK; ^7^Patient Centered Outcomes, OPEN Health, Rotterdam, The Netherlands; ^8^Heisen Health, Utrecht, The Netherlands; ^9^AbbVie Inc., North Chicago, IL, USA, ^10^Preference Research and Scientific Lead for Patient Centered Outcomes, OPEN Health, UK

**Background and aims:** Understanding preferences for advanced Parkinson's Disease (aPD) treatments is essential, particularly in the context of varying cultural, healthcare system, and economic factors that shape individual priorities across regions. This study explores treatment preferences and benefit/risk trade‐offs among people with aPD (PwaP) in two regions.

**Methods:** A discrete‐choice experiment was conducted with 304 PwaP and care partners based on 7 attributes (Table 1). Random‐parameter logit estimates were used to determine Attribute Relative importance (RI) and benefit/risk trade‐offs, comparing the United States (US; *n* = 148), with the United Kingdom (UK; *n* = 46) and Germany combined (DE; *n* = 110).

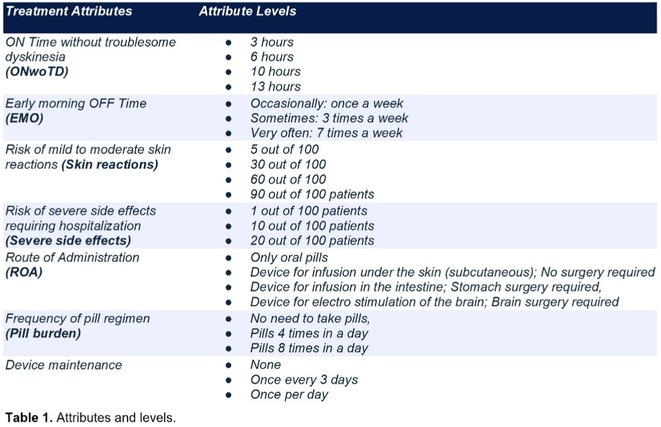

**Results:** For US and UK/DE participants, average age was 66.2 (SD = 8.6) and 56.3 (SD = 9.3), PD duration was 9.8 years (SD = 4.3) and 10.5 (SD = 4.7), and self‐reported OFF time was 4.0 hours/day (SD = 2.6) and 4.4 (SD = 1.9), respectively. The most important attributes were ON time without troublesome dyskinesia (ONwoTD) and route of administration (ROA); ONwoTD was statistically more important in the UK/DE group than in US (RI = 31.4 vs. 19.9). In US, ROA was viewed as twice as important as ONwoTD (RI = 37.2 vs. 19.9). US respondents also valued the risk of skin reactions more than their UK/DE counterparts (RI = 15.5 vs. 8.5). Both subsamples showed willingness to accept any risks of skin reactions for switching to a less invasive ROA. Both groups would consider switching from oral pills to subcutaneous ROA for a gain of 2 additional ONwoTD hours.

**Conclusion:** This study highlights regional differences in aPD treatment preferences, emphasizing need for tailored approaches across geographic locations to enhance personalized care.

**Disclosure:** JD represents Parkinson's Europe. FDR is employed by Parkinson's Europe. KRC has received fees, honoraria, and/or educational funds from AbbVie, Bial, Britannia, Britannia Bial, US Worldmeds, Otsuka, Medtronic, Zambon, Sunovion, Scion, and UCB. RP has received fees, honoraria, and/or grants from AbbVie, ACADIA, Avid, Acorda, Adamas, Biotie, Civitas, Cynapses, Global Kinetics, Kyowa, Lundbeck, National Parkinson Foundation, Neurocrine, NIH/NINDS, Parkinson Study Group, Pfizer, Sage, Sunovion, Teva Neuroscience, and US World Meds. AA has received fees, honoraria, and/or grants from AbbVie, Bayer, Biopharma, Bial, Britannia, Ever Pharma, Horizon 2020, Italian Ministry of University and Research, Italian Ministry of Health, Jazz, Medscape, Next Generation EU ‐ National Center for Gene Therapy and Drugs, and Investment PE8 – Project Age‐It: “Ageing Well in an Ageing Society”, Roche, Theravance, UCB, and Zambon. IM has received fees, honoraria, royalties, and/or grants from the Parkinson Foundation, Dystonia Coalition, AbbVie, Emalex, Medscape, Neuroderm, Praxis, Revance, Sage, Tourette Association of America, and Robert Rose Publishers. PA, MB, and HP are employees of OPEN Health. OPEN Health received funding from AbbVie for the conduct of this study. CHY, ES, MS, PK, and JCP are employees of AbbVie and may own stocks/shares in the company. MH was employed by OPEN Health at the time of study conduct. OPEN Health received funding from AbbVie for the conduct of this study.

## EPR‐256

### Identification of a Novel KIF5A mutation in a Romani family with autosomal‐dominant dystonia

#### J. Dulski ^1^; D. Pant^2^; D. Hoffman‐Zacharska^3^; M. Kwasniak‐Butowska^4^; Z. Wszolek^5^; J. Slawek^4^

##### ^1^Division of Neurological and Psychiatric Nursing, Faculty of Health Sciences, Medical University of Gdansk, Gdansk, Poland; Neurology Department, St Adalbert Hospital, Copernicus PL Ltd., Gdansk, Poland; Department of Neurology, Mayo Clinic, Jacksonville, USA; ^2^Department of Cell Biology, Emory University, Atlanta, USA; ^3^Department of Medical Genetics, Institute of Mother and Child, Warsaw, Poland; ^4^Division of Neurological and Psychiatric Nursing, Faculty of Health Sciences, Medical University of Gdansk, Gdansk, Poland; Neurology Department, St Adalbert Hospital, Copernicus PL Ltd., Gdansk, Poland; ^5^Department of Neurology, Mayo Clinic, Jacksonville, USA

**Background and aims:** Mutations in the KIF5A gene have been linked to several neurological disorders, including amyotrophic lateral sclerosis, hereditary spastic paraplegia type 10, Charcot–Marie–Tooth disease type 2, and neonatal intractable myoclonus. To date, no association between KIF5A mutations and dystonia has been reported. This study reports the first family with autosomal‐dominant dystonia exhibiting incomplete penetrance, associated with a newly identified KIF5A mutation.

**Methods:** Between 2017 and 2024, seven members of the same Roma family underwent clinical evaluations, including detailed medical histories and neurological assessments. Genetic testing included Sanger sequencing, MLPA screening of SGCE, PCR‐RFLP/BseRI testing for the common TOR1A (c.907‐909del) dystonia mutation, and whole‐exome sequencing of the proband and one affected family member. The remaining individuals were screened using Sanger sequencing.

**Figure:**
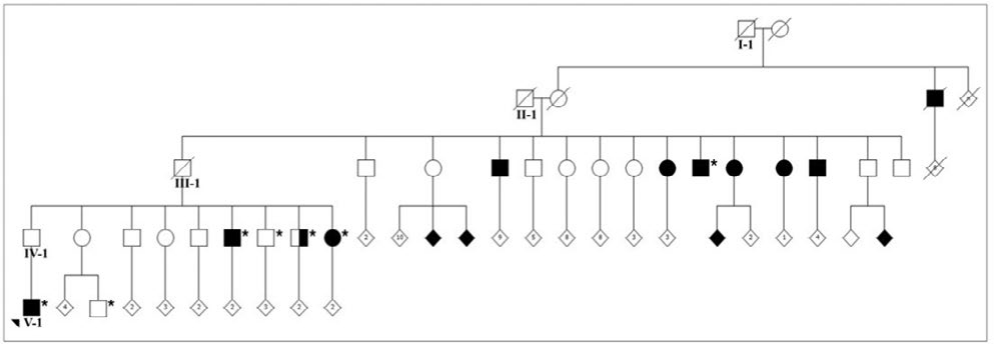
**FIGURE 1** Family pedigree

**Results:** A missense mutation in the KIF5A (c.118G > A, p.Val40Ile ) was found in four individuals with dystonia and one asymptomatic carrier, while it was absent in two unaffected relatives. This mutation is rare in the general population (frequency of 0.00001 in gnomAD 4.0) and affects a conserved amino acid residue. Computational models (M‐CAP) predicted its pathogenicity, and it was classified as likely pathogenic based on the ACMG criteria (PM1, PM2, PP2, PP3).

**Figure:**
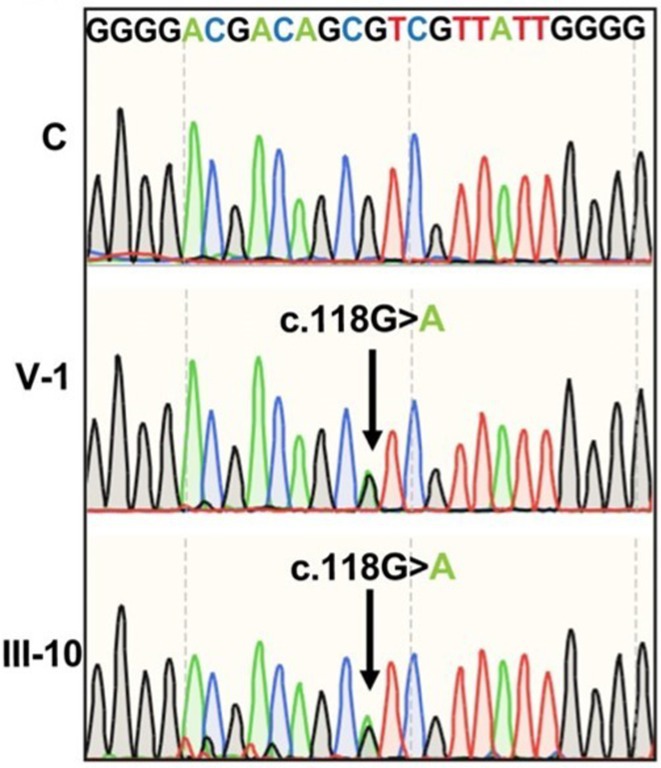
**FIGURE 2** Sanger sequencing confirmed KIF5A c.118G > A variant in both the proband (V‐1) and his relative (III‐10), while it was absent in the control (C).

**Conclusion:** This study identifies KIF5A as a potential dystonia‐related gene and underscores the importance of its inclusion in genetic screening. Integrating historically underrepresented populations into genetic research is essential, as it not only benefits these groups directly but also enhances our understanding of disorders across diverse populations, driving progress in precision medicine and targeted therapies.

**Disclosure:** J.Dulski: grants from Polish Minister of Science (Scholarship for Outstanding Young Scientists), Haworth Family Professorship in Neurodegenerative Diseases fund; editorial boards: Neurologia i Neurochirurgia Polska; speakers’ bureau: VM Media Ltd., Radosław Lipiński 90 Consulting, Ipsen; intellectual property rights: “Application of Hydrogen Peroxide and 17β ‐Estradiol and its Metabolites as Biomarkers in a Method of Diagnosing Neurodegenerative Diseases In Vitro”. D.C. Pant: grants from MDA DG and LLLF CDA; J. Slawek: co‐editor‐in‐chief of Neurologia i Neurochirurgia Polska; consultancies: Allergan, Abbvie, Ipsen, Everpharma, Merz, Novartis, Biogen, Roche, TEVA; speakers’ bureau: Allergan, Abbvie, Ipsen, Everpharma, Merz, Novartis, Biogen, Roche, TEVA; intellectual property rights: “Application of Hydrogen Peroxide and 17β ‐Estradiol and its Metabolites as Biomarkers in a Method of Diagnosing Neurodegenerative Diseases In Vitro”; Z. Wszolek: grants from NIH/NIA, NIH/NINDS, Haworth Family Professorship in Neurodegenerative Diseases fund, Albertson Parkinson's Research Foundation, PPND Family Foundation, Wilchek Family; PI or Co‐PI: Biohaven Pharmaceuticals, Vigil Neuroscience, ONO‐2808‐03, and Amylyx AMX0035‐009, Mayo Clinic APDA Center for Advanced Research; external advisory boards: Vigil Neuroscience, Inc.; consultant: Eli Lilli & Company, NovoGlia.

## EPR‐257

### Differential effect of dopaminergic treatment on bradykinesia features and limb‐kinetic apraxia in Parkinson's disease

#### M. De Riggi ^1^; G. Paparella^1^; L. Angelini^1^; A. Cannavacciuolo^1^; D. Costa^2^; D. Birreci^1^; M. Bologna^1^

##### ^1^Department of Human Neurosciences, Sapienza, University of Rome, Rome, Italy; ^2^IRCCS Neuromed, Pozzilli, IS, Italy

**Background and aims:** Bradykinesia is a primary motor symptom in Parkinson's disease (PD), but other cognitive‐motor disorders, such as limb‐kinetic apraxia, can also contribute to motor dysfunction and influence treatment responses. This study aimed to investigate the differential effects of dopaminergic therapy on bradykinesia and limb‐kinetic apraxia in PD patients using kinematic analysis. Additionally, transcranial magnetic stimulation (TMS) was utilized to examine the neural pathways involved.

**Methods:** Twenty‐five PD patients and 24 age‐ and gender‐matched healthy controls (HC) were assessed in both OFF‐ and ON‐medication states. Kinematic analysis evaluated bradykinesia using a finger‐tapping task and limb‐kinetic apraxia using a 10‐second coin rotation task. Corticospinal excitability was assessed through TMS, measuring resting motor thresholds, motor‐evoked potential input/output curves, short‐interval intracortical inhibition, and interhemispheric inhibition.

**Results:** In the OFF‐medication state, PD patients showed slower velocity, reduced amplitude (sequence effect), and decreased regularity in finger‐tapping movements compared to healthy controls (HC). Similar findings were observed in the coin rotation task. Dopaminergic therapy improved finger‐tapping velocity but had no significant effect on other parameters or the coin rotation task, indicating a differential impact on the two motor tasks. Increased M1 excitability was associated with impaired motor performance in both tasks, but no such correlations were observed in the ON state. Additionally, no correlations were found between changes in kinematic parameters and TMS measures from the OFF to the ON state.

**Conclusion:** The differential treatment effects on bradykinesia and limb‐kinetic apraxia in PD suggest distinct pathophysiological mechanisms, potentially involving cortical and subcortical systems with different sensitivities to dopaminergic therapy.

**Disclosure:** Nothing to disclose.

## EPR‐258

### The allosteric activator of glucocerebrosidase VQ‐101 shows sustained activation of lysosomal GCase in humans

#### M. Facheris ^1^; D. Ysselstein^1^; J. Van der Valk^2^; E. Thijssen^2^; L. Pagan^2^; G. Valstar^2^; P. Kremer^2^; M. Hagey^1^; J. Cedarbaum^3^; J. Sullivan^1^; K. Hunt^1^; O. Siddiqui^1^

##### ^1^Vanqua Bio, Chicago, USA; ^2^Centre for Human Drug Research (CHDR), Neurology, Leiden, the Netherlands; ^3^Yale School of Medicine, New Haven, USA

**Background and aims:** People with Parkinson's disease (PD) who carry a heterozygous GBA1 mutation (GBA‐PD) have approximately a 30% reduction in glucocerebrosidase (GCase) activity, resulting in lysosome dysfunction and accumulation of misfolded alpha synuclein (aSyn). In GBA‐PD‐derived dopaminergic neurons, VQ‐101 shows a concentration‐dependent increase in GCase activity, with 50% activation leading to significant blockage of insoluble aSyn accumulation. Safety/tolerability, pharmacokinetics, food effect, and pharmacodynamics of VQ‐101 are assessed in healthy volunteers (HVs) and PD patients with and without GBA1 mutations.

**Methods:** Phase 1a evaluated single and multiple ascending doses of VQ‐101 for up to 14 days in HVs. Target engagement was assessed by measuring lysosomal GCase activity using a validated live cell assay in fresh blood samples.

**Results:** 88 HVs were randomized to receive VQ‐101 or placebo in a double‐blind fashion. No serious adverse events or discontinuations due to adverse events were reported. VQ‐101 showed full CNS penetrance. Exposures increased with escalating doses and were not influenced by food intake. VQ‐101 showed dose‐dependent and sustained (> 50% activation observed at Ctrough) lysosomal GCase activation following multiple doses.

**Conclusion:** VQ‐101 is safe and well tolerated at all tested dose levels. VQ‐101 has a favorable CSF and plasma PK profile, supporting once daily oral dosing and demonstrates dose‐dependent target engagement in HVs. These results, together with preclinical data, support the potential for VQ‐101 to slow or stop the progression of PD by increasing GCase activity and blocking the accumulation of misfolded aSyn. Enrolment of PD patients in phase 1b, evaluating multiple doses of VQ‐101, is ongoing.

**Disclosure:** MFF, DY, MH, JS, KH and OS are employees of Vanqua Bio and may own equity in the company. JMC is a clinical consultant for Vanqua Bio and may own equity in the company. JPVDV, ET, LP, GV, and PHCK have nothing to disclose.

## EPR‐259

### Age at onset of depression/anxiety for people with Friedreich ataxia: Real‐world data from medical claims

#### S. Kuo ^1^; S. Nayar^2^; B. Bian^3^; D. Gomes^4^; S. England^3^; J. McKay^3^; T. Wang^4^; R. Avila^3^; S. Perlman^5^

##### ^1^Columbia University Medical Center, New York, USA; ^2^Medstar Georgetown University Hospital, Washington, USA; ^3^Biogen, Inc., Cambridge, USA; ^4^Voxanalytica, San Francisco, USA; ^5^UCLA Medical Center, Los Angeles, USA

**Background and aims:** Friedreich ataxia (FA) is a rare, genetic, multisystem disorder presenting mainly with ataxia, but features a complex phenotype of non‐motor symptoms with variable onset. Because limited data are available on the onset of non‐motor manifestations in patients with FA, this study aimed to determine the age at onset of depression/anxiety relative to key progression milestones (i.e., cardiomyopathy; loss of ambulation) based on real‐world data from US medical claims.

**Methods:** We conducted a retrospective study based on de‐identified medical claims linked to mortality data covering October 2015 to March 2024. We stratified the cohort by age at FA diagnosis: 0‐7, 8‐14, 15‐24, and 25‐39 years. Key endpoints were age at FA diagnosis, as well as age at onset for depression/anxiety, cardiomyopathy, and loss of ambulation.

**Results:** The cohort included 927 patients with FA. For patients aged 0‐7, 8‐14, 15‐24, and 25‐39 years, the median age at FA diagnosis was 4.0 (*n* = 129), 11.0 (*n* = 225), 19.0 (*n* = 261), and 32.0 years (*n* = 312), respectively. Depression/anxiety were observed in 6% (*n* = 8; median age, not reached), 36.9% (*n* = 83; 17.1 years), 44.1% (*n* = 115; 23.7 years), and 43.3% (*n* = 135; 37.1 years) for patients aged 0‐7, 8‐14, 15‐24, and 25‐39, respectively (Figure). In patients diagnosed between age 8‐39 years, depression/anxiety were observed in temporal proximity to loss of ambulation (Table).

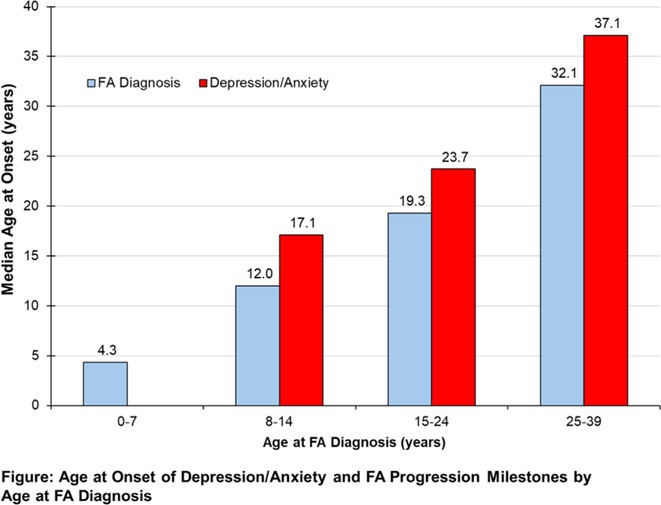

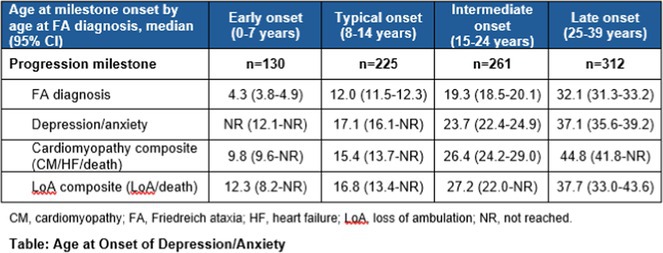

**Conclusion:** The real‐world data reveal depression and anxiety are common in FA, reinforcing the importance of comprehensive care for patients, particularly in partnering with trained mental health professionals to counsel patients on the non‐motor symptoms.

**Disclosure:** This study was funded by Biogen. BB, SME, JM, RLA are employees of Biogen and may hold stock/stock options.

## EPR‐260

### Impact of the LRRK2‐G2019S mutation on neuropsychiatric features in Parkinson's disease patients

#### G. Ali Barreh; I. Sghaier; Y. Abida; M. Elloumi; A. Gharbi; I. Kacem; A. Gargouri; R. Gouider

##### Department of Neurology, Razi Universitary Hospital, Tunis, Tunisia

**Background and aims:** Parkinson's disease (PD) is a complex neurodegenerative disorder characterized by both motor and non‐motor symptoms, including neuropsychiatric symptoms (NPSs). Among the genetic factors, the LRRK2‐G2019S mutation is the most prevalent in certain populations. This study aimed to investigate the prevalence of the correlation of LRRK2‐G2019S with NPSs.

**Methods:** This longitudinal retrospective study was conducted in the Department of Neurology, Razi University Hospital. We included PD patients based on the Movement Disorders Society criteria. Medical records were reviewed for clinical, treatment, and neuropsychological assessments. All patients were screened for the LRRK2‐G2019S mutation using Sanger sequencing. Correlations between the mutation and NPSs were analyzed, adjusting for sex, disease duration, and Levodopa equivalent dose.

**Results:** We included 393 PD patients. The prevalence of the LRRK2‐G2019S mutation was 41.5%. Mutation carriers showed significantly fewer sleep disturbances, hallucinations, depression, appetite and eating disorders, delirium, apathy, euphoria, disinhibition, and aberrant motor behaviors (adjusted *p*‐values < 0.05).

**Conclusion:** This study highlights a distinct neuropsychiatric profile among LRRK2‐G2019S mutation carriers, characterized by a lower prevalence of NPSs. These findings could guide personalized management strategies and enhance understanding of genotype‐phenotype correlations in PD. Further research is needed to explore the underlying mechanisms and potential therapeutic implications.

**Disclosure:** Nothing to disclose.

## MS and related disorders 3

## EPR‐261

### Nutritional status and Mediterranean diet adherence in pediatric multiple sclerosis, clinical‐radiological correlations

#### A. Castiello ^1^; C. Di Monaco^1^; A. Carotenuto^1^; M. Petracca^2^; F. Felicetti^2^; C. Pozzilli^2^; V. Torri Clerici^3^; L. Lorefice^4^; M. Valeriani^5^; A. Gajofatto^6^; F. Pinardi^7^; E. Scarpato^8^; M. Serra^8^; A. Esposito^1^; M. Moccia^1^; A. Staiano^8^; V. Brescia Morra^1^; C. Mandato^9^; R. Lanzillo^1^

##### ^1^Department of Neuroscience, Reproductive Science and Odontostomatology, Federico II University of Naples, Naples, Italy.; ^2^Department of Human Neurosciences, Sapienza University, Rome, Italy.; ^3^IRCCS Istituto Neurologico C. Besta, Neuroimmunology Unit, Milan, Italy.; ^4^Multiple Sclerosis Centre, ASL Cagliari, 09126 Cagliari, Italy.; ^5^Bambino Gesù Children's Hospital (Istituti di Ricovero e Cura a Carattere Scientifico), Rome, Italy.; ^6^Department of Neurosciences, Biomedicine and Movement Sciences, University of Verona, Verona, Italy.; ^7^IRCCS Istituto delle scienze neurologiche di Bologna, UOSI Riabilitazione Sclerosi Multipla Bologna, Bologna, Italy.; ^8^Department of Translational Medical Science, Section of Pediatrics, University of Naples Federico II, 80138 Naples, Naples, Italy.; ^9^Department of Medicine, Surgery and Dentistry “Scuola Medica Salernitana”, Pediatrics Section, University of Salerno, 84081 Baronissi, Salerno, Italy.

**Background and aims:** Recent studies have established a relationship between dietary patterns and Multiple Sclerosis (MS), with dietary habits influencing disease onset and progression. The Mediterranean Diet (MD) has been shown to have a protective role in inflammation and neurodegenerative processes in MS, but data on pediatric onset MS (POMS) is limited.

**Methods:** This multicenter observational study included POMS or Clinically Isolated Syndrome (CIS) patients, aged 24 years or less. Adherence to MD was assessed using the KIDMED questionnaire. Collected data included clinical and radiological features, age at onset/diagnosis, Expanded Disability Status Scale (EDSS), disease duration, MRI lesion load, and Annualized Relapse Rate (ARR). Height, weight, and BMI were also recorded.

**Results:** The study included 100 POMS and 81 HCs. The mean KIDMED score was 5.1  ± 2.9 in MS patients and 5.0  ± 2.4 in HCs, with no significant difference between the two groups (*p* = 0.82). Linear regression analyses revealed no significant correlation between KIDMED scores and weight, height, or BMI. However, a direct correlation was observed between KIDMED scores and age at diagnosis (r = 0.24, *p* = 0.03), an inverse correlation with disease duration at diagnosis (r = ‐0.24, *p* = 0.03) and no significant correlations between KIDMED scores and EDSS at diagnosis, ARR, or the number of relapses pre‐ and post‐diagnosis. Moreover, an higher BMI was associated with increased EDSS (r = 0.29, *p* = 0.01) and a higher probability of switching from the first‐line therapy (r = 0.43, *p* = 0.001).

**Conclusion:** MD appears related to age of onset in POMS, and BMI influences prognosis. Nutrition and lifestyle interventions, especially in at‐risk individuals, could help prevent MS progression.

**Disclosure:** A.E. has received honoraria from Novartis. M.M. has received research grants from ECTRIMS‐MAGNIMS, the UK MS Society, and Merck, and honoraria from Biogen, BMS Celgene, Ipsen, Janssen, Merck, Novartis, Roche, and Sanofi‐Genzyme. M.P. has received research grants from the Italian MS Foundation and Baroni Foundation, honoraria from Health & Life and Biogen, and sponsorship for travel/meeting expenses from Novartis, Roche, and Merck. R.L. has received honoraria from Biogen, Merck, Novartis, Roche, and Teva. V.B.M. has received research grants from the Italian MS Society and Roche, and honoraria from Bayer, Biogen, Merck, Mylan, Novartis, Roche, Sanofi‐Genzyme, and Teva. A.C. has received research grants from Almirall, research grants from ECTRIMS‐MAGNIMS, and honoraria from Almirall, Biogen, Roche, Sanofi‐Genzyme, Merck, Ipsen, and Novartis.CP has served on scientific advisory boards for Actelion, Biogen, Genzyme, Hoffmann‐La Roche Ltd, Merck, Novartis, Sanofi, Teva, and has received consulting and/or speaking fees, research support and travel grants from Allergan, Almirall, Biogen, Genzyme, Hoffmann‐La Roche Ltd, Merck, Novartis, Sanofi and Teva; None of the other authors has any conflict of interest to disclose.

## EPR‐262

### Choroid plexus volume and serum neurofilament light levels in relation to brain atrophy and lesion load in MS

#### C. Tafrali ^1^; M. Martinez‐Serrat^1^; R. Demjaha^1^; A. Buchmann^1^; S. Hechenberger^2^; B. Helmlinger^2^; P. Opriessnig^2^; D. Pinter^2^; A. Damulina^2^; S. Wurth^2^; B. Heschl^2^; D. Leppert^3^; P. Benkert^3^; J. Kuhle^3^; S. Ropele^2^; C. Enzinger^2^; M. Khalil^1^

##### ^1^Neurology Biomarker Research Unit, Department of Neurology, Medical University of Graz, Graz, Austria; ^2^Department of Neurology, Medical University of Graz, Graz, Austria; ^3^Department of Neurology, University Hospital and University of Basel, Basel, Switzerland

**Background and aims:** The choroid plexus (CP), responsible for cerebrospinal fluid (CSF) production and the blood‐CSF barrier, plays a key role in central nervous system inflammation. Increased CP volume (CPV) in multiple sclerosis (pwMS) links to clinical and MRI indicators of disease progression, suggesting CPV's potential as an imaging biomarker. However, the predictive value of CPV, alongside serum neurofilament light (sNfL), a blood‐based marker for neuro‐axonal damage, for lesion load (LL) and brain atrophy (BA) remains unclear. This study aimed to determine how well CPV and sNfL indicate lesion burden and brain volume changes over a median follow‐up of 5.3 years (IQR: 4.6‐5.5).

**Methods:** Ninety‐six pwMS (17 with clinically isolated syndrome, 70 with relapsing‐remitting MS, and 9 with progressive MS) and 49 age‐ and sex‐matched healthy controls (HC) participated. Participants underwent 3T MRI to evaluate normalized brain volume and lesion load using FreeSurfer and SIENA. sNfL was measured with Simoa HD‐X analyzer. Longitudinal data were available for 60 pwMS. We used adjusted partial correlations and multiple linear regression to identify predictors of LL and brain volume.

**Results:** CPV (*p* = 0.002) and sNfL (*p* < 0.001) were significantly higher in pwMS compared to HC. Cross‐sectional regression showed CPV was independently linked to reduced brain volume and higher LL (β = ‐0.02; *p* < 0.001 and β = 1.55; *p* < 0.001). In longitudinal regression, only sNfL remained a significant predictor of BA (β = 1.33; *p* = 0.003), not CPV.

**Conclusion:** Although both CPV and sNfL correlate with MRI signs of brain damage in pwMS, longitudinal analysis identified sNfL as the sole marker linked to more pronounced BA.

**Disclosure:** C.T: travel funding (TF) and speaker honoraria (SH) from Merck. M.M, A.B, P.O, A.D, S.W, B.H, P.B: Nothing to disclose R.D: TF from from Janssen, Novartis and Sanofi S.H: SH from Roche and Bristol‐Myers Squibb B.H: SH from Roche, Sanofi, and Bristol‐Myers Squibb, and TF from Janssen. D.P: a member of the advisory board for “Cognition and MS” for Novartis and has received SH from Biogen, Novartis, MedAhead and Bristol‐Myers Squibb D.L: was Chief Medical Officer of GeNeuro until end of 2023 J.K: SH, research support (RS), TF, and/or advisory boards by Swiss MS Society, Swiss National Research Foundation (320030_212534/1), University of Basel, Progressive MS Alliance, Alnylam, Bayer, Biogen, Bristol Myers Squibb, Celgene, Immunic, Merck, Neurogenesis, Novartis, Octave Bioscience, Quanterix, Roche, Sanofi, Stata DX C.E: received TF and SH from Biogen Idec, Bayer Schering Pharma, Merck Serono, Novartis, Genzyme and Teva Pharmaceutical Industries Ltd./Sanofi‐Aventis, Shire; received RS from Merck Serono, Biogen Idec, and Teva Pharmaceutical Industries Ltd./Sanofi‐Aventis; and scientific advisory boards for Bayer Schering Pharma, Biogen Idec, Merck Serono, Novartis, Genzyme, Roche, and Teva Pharmaceutical Industries Ltd./Sanofi‐ Aventis M.K: TF and SH from Bayer, Biogen, Novartis, Merck, Sanofi and Teva and scientific advisory boards for Biogen, Bristol‐Myers Squibb, Gilead, Merck, Novartis, and Roche. He received RS from Biogen, Novartis and Teva.

## EPR‐263

### Childhood infections affect timing of multiple sclerosis onset in adults: EnvIMS study

#### C. Ferri ^1^; F. Rovito^2^; M. Laudisi^1^; K. Myhr^3^; T. Riise^4^; C. Wolfson^5^; M. Pugliatti^6^

##### ^1^Department of Neuroscience, S. Anna University Hospital, Ferrara, Italy; ^2^Department of Neuroscience and Rehabilitation, University of Ferrara, Italy.; ^3^Department of Clinical Medicine, University of Bergen, Norway; The Norwegian Multiple Sclerosis Registry and Biobank, Department of Neurology, Haukeland University Hospital, Bergen, Norway.; ^4^Department of Global Public Health and Primary Care, University of Bergen, Bergen, Norway/The Norwegian Multiple Sclerosis Competence Centre, Department of Neurology, Haukeland University Hospital, Bergen, Norway.; ^5^Department of Epidemiology, Biostatistics, and Occupational Health, School of Population and Global Health, McGill University, Montreal, Quebec, Canada.; ^6^Department of Neuroscience and Rehabilitation, University of Ferrara, Italy; S. Anna University Hospital, Ferrara, Italy.

**Background and aims:** Since early‐life challenge of the immune system can affect the risk of later development of autoimmune diseases, childhood infections have been suggested as risk factors/triggers for multiple sclerosis (MS). Except for Epstein Barr Virus, the role of other infectious agents in the pathogenesis of MS remains poorly understood. We aimed at investigating the association between MS and exposure to childhood infections and its role on timing of disease onset.

**Methods:** We analyzed data collected within the EnvIMS study, a multinational case‐control population‐based study, including information on measles, rubella, mumps, and chickenpox of Italian and Norwegian populations. Crude and adjusted odds ratio for index age, smoking habit, infectious mononucleosis, and low sun exposure are presented with 95% confidence intervals. An ANCOVA was performed to investigate the association between infections and age at MS onset, with smoking habit and infectious mononucleosis as covariates. Stratification by sex is presented.

**Results:** 2,040 Italians, and 2,674 Norwegians were included. MS was not associated with any of the infections considered. Measles was significantly associated with delayed MS onset in all populations, as were rubella and mumps in Norwegians, especially in women. An earlier MS onset was instead associated with chickenpox in Italians and Norwegians and with rubella in Italian men.

**Figure:**
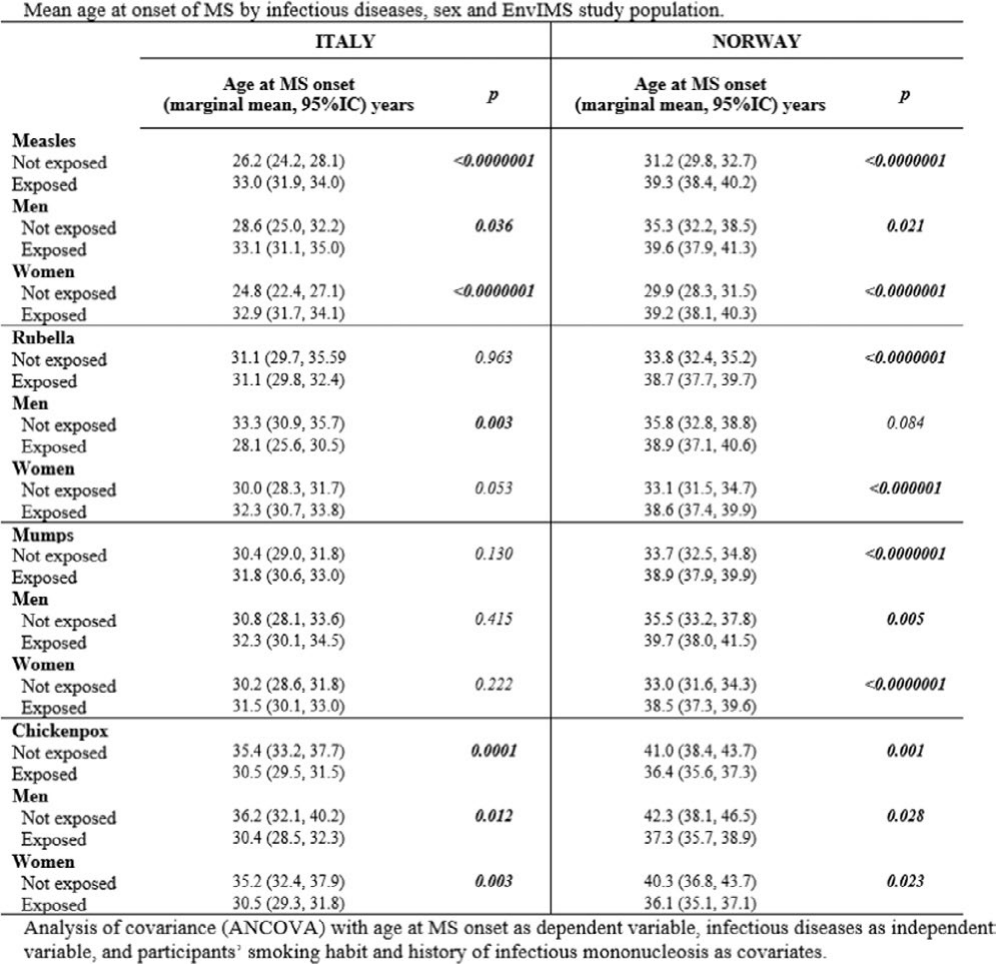
TABLE 1

**Conclusion:** Childhood infections are not associated with MS status. Measles consistently delays the MS onset, while chickenpox anticipates it. Mumps and rubella show different effects depending on the population considered. These findings suggest an influence of childhood infections, especially measles and chickenpox, on MS course.

**Disclosure:** The authors declare no conflict of interest. The EnvIMS study was supported by Fondazione Italiana Sclerosi Multipla, FISM, grants n. 2007/R/14, and n. 2008/R/19; Financially supported by the EuropeanUnion – Next Generation EU – NRRP M6C2 – Investment 2.1 Enhancement and strengthening of biomedical research in the NHS ‐ PNRR‐MAD‐2022‐12376868.

## EPR‐264

### Environmental risk factors and risk of MS in subjects with radiologically isolated syndrome: A case‐control study

#### C. Zanetta ^1^; F. Laserra^2^; A. Giordano^2^; F. Esposito^3^; V. Viti^1^; M. Rocca^4^; M. Filippi^5^

##### ^1^Neurology Unit, Neurorehabilitation Unit and Multiple Sclerosis Center, IRCCS San Raffaele Scientific Institute, Milan, Italy; ^2^Vita‐Salute San Raffaele University, Milan, Italy; ^3^Neurology Unit and Multiple Sclerosis Center, IRCCS San Raffaele Scientific Institute, Milan, Italy; ^4^Neurology Unit and Multiple Sclerosis Center, IRCCS San Raffaele Scientific Institute, Milan, Italy; Neuroimaging Research Unit, Division of Neuroscience, IRCCS San Raffaele Scientific Institute; Vita‐Salute San Raffaele University, Milan, Italy; ^5^Neurology Unit, Neurorehabilitation Unit, Multiple Sclerosis Center, Neurophysiology Service, Neuroimaging Research Unit, Division of Neuroscience, IRCCS San Raffaele Scientific Institute; Vita‐Salute San Raffaele University, Milan, Italy

**Background and aims:** Radiologically Isolated Syndrome (RIS) is the incidental finding of demyelinating lesions at MRI without history of symptoms of Multiple Sclerosis (MS). Environmental factors play an important role in the development of MS. We investigated the impact of environmental factors and their association with conversion to MS in RIS patients.

**Methods:** Subjects with a RIS diagnosis (cases) and patients with a Clinically Isolated Syndrome (CIS)/Relapsing‐Remitting MS (RRMS) onset (controls) were included. Patients were administered an environmental questionnaire, asking for exposure to environmental factors prior to onset. The distribution of clinical, demographic characteristics and environmental factors between the two groups and their impact on the risk of conversion to MS were analyzed.

**Results:** Forty‐two RIS subjects and 216 controls were included. RIS patients had more frequently a clinical onset with supratentorial symptoms (28.2% vs. 9.8%), while controls with the involvement of the visual pathway (5.1% vs. 24.7%). A higher proportion of controls reported outdoor activity during childhood (44.3% vs. 23.7%). RIS patients that converted within 5 years since onset had more frequently an onset with supratentorial symptoms (33.3% vs. 10.0%), were more likely to be underweight during adolescence (14.3% vs. 0.0%), to have had pregnancy losses before RIS diagnosis (18.2% vs. 0.0%) and to have used Assisted Reproduction Technology (ART) before RIS diagnosis (9.1% vs. 0.0%).

**Conclusion:** Being underweight during adolescence, the use of ART and a history of pregnancy losses were associated to a higher risk of conversion from RIS to MS. Outdoor activity during childhood was more frequent in patients with CIS/RRMS.

**Disclosure:** CZ received compensation from Alexion, Amgen, Astrazeneca, Biogen, Bristol Myers Squibb, Janssen, Merck, Novartis, Roche, Sandoz, Sanofi. FL and VV have nothing to disclose. AG and FE received consulting fees from Novartis, Merck Serono and Genzyme. MAR received consulting fees from Biogen, Bristol Myers Squibb, Eli Lilly, Janssen, Roche, and speaker honoraria from AstraZaneca, Biogen, Bristol Myers Squibb, Bromatech, Celgene, Genzyme, Horizon Therapeutics Italy, Merck Serono SpA, Novartis, Roche, Sanofi and Teva, she receives research support from the MS Society of Canada, the Italian Ministry of Health, the Italian Ministry of University and Research, and Fondazione Italiana Sclerosi Multipla, she is Associate Editor for Multiple Sclerosis and Related Disorders. MF is Editor‐in‐Chief of the Journal of Neurology, Associate Editor of Human Brain Mapping, Neurological Sciences, and Radiology; received compensation from Alexion, Almirall, Biogen, Merck, Novartis, Roche, Sanofi; Bayer, Biogen, Celgene, Chiesi Italia SpA, Eli Lilly, Genzyme, Janssen, Merck‐Serono, Neopharmed Gentili, Novartis, Novo Nordisk, Roche, Sanofi, Takeda, and TEVA; scientific direction of educational events for Biogen, Merck, Roche, Celgene, Bristol‐Myers Squibb, Lilly, Novartis, Sanofi‐Genzyme; he receives research support from Biogen Idec, Merck‐Serono, Novartis, Roche, the Italian Ministry of Health, the Italian Ministry of University and Research, and Fondazione Italiana Sclerosi Multipla.

## EPR‐265

### Vidofludimus calcium shows potential neuroprotective effects in an in vivo multiple sclerosis model by Nurr1 modulation

#### E. Peelen ^1^; H. Wu^2^; A. Herrmann^1^; T. Wulff^1^; M. Jafari^1^; C. Gege^1^; A. Muehler^1^; D. Vitt^1^; Z. Sun^2^; H. Kohlhof^1^

##### ^1^Immunic AG, Graefelfing, Germany; ^2^Department of Immunology & Theranostics, Beckman Research Institute of City of Hope, Duarte, USA

**Background and aims:** The transcription factor nuclear receptor‐related 1 (Nurr1) regulates genes that enhance neuronal development, function and survival. Vidofludimus calcium (VidoCa), currently in phase 2 and 3 clinical trials for progressive and relapsing MS, respectively, exhibited potent Nurr1 activation in vitro. In this study, the potential neuroprotective effects of VidoCa through activating Nurr1 were further elucidated in vivo.

**Methods:** Mice were immunized with myelin oligodendrocyte glycoprotein (MOG) 35‐55 to induce experimental autoimmune encephalomyelitis (EAE) followed by daily scoring for disease progression and treatment with VidoCa or vehicle. At end of study, gene expression in the central nervous system (CNS) was evaluated by qRT‐PCR and plasma brain‐derived neurotrophic factor (BDNF) and neurofilament light chain (NfL) levels were determined by ELISA. Expression of amyloid precursor protein (APP), ionized calcium‐binding adaptor molecule‐1 (Iba‐1) and myelin basic protein (MBP) was assessed by immunohistochemical staining.

**Results:** Next to attenuating disease severity, VidoCa induced Nurr1‐regulated gene expression in the CNS, including tyrosine hydroxylase (Th), glycosylphosphatidylinositol‐specific phospholipase D1 (Gpld1) and delta‐like non‐canonical Notch ligand‐1 (Dlk1), is elevated in the CNS of EAE mice treated with VidoCa. Interestingly, in VidoCa‐treated mice a potential peripheral Nurr1 activation biomarker, BDNF, is augmented, while plasma NfL levels as well as APP expression in spinal cord are reduced indicating diminished axonal/neuronal damage. Also, expression of the microglial activation marker Iba‐1 was reduced while an increase in MBP as myelin marker was observed.

**Conclusion:** These data indicate that VidoCa potentially enhances neuroprotection in vivo by activating Nurr1.

**Disclosure:** This project was funded by Immunic Therapeutics. E.P., A.H., T.W., M.J., C.G., and H.K. are employees of Immunic AG, holding shares and/or stock options of the parent company, Immunic, Inc. A.M. and D.V. are employees of Immunic AG and Immunic Inc. and are shareholders of Immunic Inc. E.P., C.G. A.M., D.V., and H.K. are inventors on a patent application covering the topic. The works of Z.S. and H.W. are supported by Immunic AG.

## EPR‐266

### Wearable‐derived data could unveil subtle disability changes in multiple sclerosis

#### G. Corsini ^1^; M. Recano^1^; A. Esposito^1^; V. Nicolella^1^; D. Ranucci^1^; A. Castiello^1^; M. Petracca^2^; M. Moccia^3^; R. Lanzillo^1^; V. Brescia Morra^1^; A. Carotenuto^1^

##### ^1^1. Department of Neurosciences, Reproductive Sciences and Odontostomatology, University of Naples Federico II, Naples, Italy., ^2^2. Department of Human Neurosciences, Sapienza University of Rome, Rome, Italy., ^3^3. Department of Molecular Medicine and Medical Biotechnology, Federico II University of Naples, Naples, Italy.

**Background and aims:** Multiple Sclerosis (MS) is a disabling disorder affecting young adults. Conventional clinical assessment through the widely used Expanded Disability Status Scale (EDSS) often misses subtle changes occurring along the disease course. Wearable technology provides real‐world data, hence enabling a more subtle evaluation of disability in a longer timeframe. This study aimed to validate the correlation between wearable‐generated metrics and EDSS.

**Methods:** We enrolled 70 MS patients (aged 18‐60 years old; EDSS < 5 and absence of smartwatch use). Patients underwent EDSS, Timed 25‐Foot Walk (T25FW), and Nine‐Hole Peg Test (9HPT). Phone device data, including step count, gait asymmetry, and walking speed, were collected via smartphone applications (for iOS and Android). Cross‐sectional correlations between wearable metrics, EDSS, T25FW and 9HPT were assessed using pairwise correlations, while longitudinal trends in wearable data and EDSS were analyzed to detect changes over 12 months before enrollment.

**Results:** Lower walking speed and higher gait asymmetry correlated with higher EDSS (corr.coeff = ‐0.62, *p* < 0.001 and corr.coeff. = 0.63, *p* < 0.001, respectively). and higher T25FW (corr.coeff. = ‐0.63, *p* < 0.001 and corr. Coeff. = ‐0.88, *p* < 0.001, respectively). Moreover, while there were no significant changes for the EDSS over one year before enrollment, we reported a step count decrease (mean: 4536 vs. 4063 steps, *p* = 0.04) during the same period.

**Conclusion:** Wearable devices offer the potential to remodel disability assessment in MS by detecting subtle progression earlier, allowing a timely diagnosis ultimately resulting in proper pharmacological strategies.

**Disclosure:** A.E. has received honoraria from Novartis. V.N. has received sponsorship for travel/meeting expenses from Alexion and Fujirebio. M.M. has received research grants from ECTRIMS‐MAGNIMS, the UK MS Society, and Merck, and honoraria from Biogen, BMS Celgene, Ipsen, Janssen, Merck, Novartis, Roche, and Sanofi‐Genzyme. M.P. has received research grants from the Italian MS Foundation and Baroni Foundation, honoraria from Health & Life and Biogen, and sponsorship for travel/meeting expenses from Novartis, Roche, and Merck. R.L. has received honoraria from Biogen, Merck, Novartis, Roche, and Teva. V.B.M. has received research grants from the Italian MS Society and Roche, and honoraria from Bayer, Biogen, Merck, Mylan, Novartis, Roche, Sanofi‐Genzyme, and Teva. A.C. has received research grants from Almirall, research grants from ECTRIMS‐MAGNIMS, and honoraria from Almirall, Biogen, Roche, Sanofi‐Genzyme, Merck, Ipsen, and Novartis. None of the other authors has any conflict of interest to disclose.

## EPR‐267

### Impact of pain, spasticity and bladder dysfunction in MOGAD and NMOSD: Preliminary results of a RIREMS group study

#### R. Orlandi ^1^; V. Torri Clerici^2^; D. Ferraro^3^; R. Lanzillo^4^; M. Moccia^4^; A. Gallo^5^; V. Nociti^6^; S. Mariotto^1^; M. Calabrese^1^; P. Annovazzi^7^; P. Ragonese^8^; F. Buttari^9^; L. Lorefice^10^; C. Gasperini^11^; E. Cocco^10^; C. Solaro^12^; A. Gajofatto^1^

##### ^1^Department of Neurosciences, Biomedicine and Movement Sciences, University of Verona, Verona, Italy; ^2^Neuroimmunology Unit, IRCCS Istituto Neurologico C. Besta, Milan, Italy; ^3^Neurology Unit, Department of Neurosciences, Azienda Ospedaliero‐Universitaria di Modena, Modena, Italy; ^4^Department of Neuroscience, Federico II University of Naples, Naples, Italy; ^5^Department of Advanced Medical and Surgical Sciences, University of Campania “L. Vanvitelli”, Naples, Italy; ^6^Multiple Sclerosis Center, Fondazione Policlinico Universitario “A. Gemelli” IRCCS, Rome, Italy; ^7^Neuroimmunology Unit‐Multiple Sclerosis Center, Gallarate Hospital, Gallarate, Italy; ^8^Neurology Unit, Department of Biomedicine, Neurosciences and Advanced Diagnostics, University of Palermo, Palermo Italy; ^9^Department of System Medicine, University of Tor Vergata, Rome, Italy; IRCCS Neuromed Institute, Pozzilli (IS), Italy, ^10^Multiple Sclerosis Center, Binaghi Hospital, Cagliari, Italy, ^11^MS Center, Department of Neuroscience, San Camillo Forlanini Hospital, Rome, Italy, ^12^Neurology Unit, Galliera Hospital, Genoa, Italy

**Background and aims:** The impact of pain, fatigue, depression, spasticity and bladder dysfunction in patients with myelin oligodendrocyte glycoprotein antibody‐associated disease (MOGAD) and aquaporin‐4‐IgG‐seropositive neuromyelitis optica spectrum disorder (NMOSD‐AQP4+) is still unclear. This ongoing multicenter longitudinal study aimed to compare the frequency of these symptoms in an Italian cohort of adult MOGAD and NMOSD‐AQP4+ patients.

**Methods:** As of January 15 2025, 15 MOGAD (5 F) and 23 NMOSD‐AQP4+ (21 F) have been enrolled. Pain is assessed through the Numerical Rating Scale (NRS) and Douleur Neuropathique en 4 Questions (DN4) questionnaire to discriminate neuropathic from non‐neuropathic pain. Spasticity is investigated through Ashworth scale. Fatigue Scale ‐ Motor and Cognitive (FSMC) and Beck's Depression Index‐2 (BDI‐II) are administered to assess fatigue and depression; urinary urgency/incontinence is assessed through International Consultation on Incontinence Questionnaire (ICIQ‐UI). Questionnaires and tests are performed at baseline and after 18+/‐6 months.

**Results:** Median ICIQ‐UI score was significantly higher in NMOSD‐AQP4+ (8, range 0‐21) compared to MOGAD patients (0, range 0‐7, *p* < 0.001). Median NRS score was 1 (0‐9) in MOGAD and 5 (0‐9) in NMOSD‐AQP4+ (*p* = 0.011); moderate‐to‐severe pain was less frequent in MOGAD cases (2, 13.3%) compared to NMOSD‐AQP4+ (14, 60.9%; *p* = 0.006). Spasticity was observed in 2 MOGAD (13.3%) and 12 NMOSD (57.1%) patients (*p* = 0.008). Neuropathic pain occurrence, FSMC and BDI‐II scores did not differ significantly between groups.

**Conclusion:** Impact of urinary urgency/incontinence, pain and spasticity appears to be lower in MOGAD compared to NMOSD‐AQP4+ patients. This data confirms that MOGAD is relatively less disabling compared to NMOSD‐AQP4+.

**Disclosure:** P. Annovazzi received honoraria for lecturing and participation in advisory boards, and/or travel expenses for attending congresses and meetings from Alexion, Almirall, Amgen, Biogen, BMS, Janssen, Lundbeck, Merck, Novartis, Roche, Sanofi‐Genzyme, Teva and Viatris. M. Calabrese received speaker honoraria from Biogen, Bristol Myers Squibb, Merck Serono, Novartis, and Roche and received research support from the Progressive MS Alliance and Italian Minister of Health and Biogen, Bristol Myers Squibb, Merck Serono, Novartis, and Roche. All the other Authors have nothing to disclose.

## EPR‐268

### Reduced duration of breastfeeding is associated with an increased risk of pediatric MS: The PEDIGREE study

#### S. Pilotto ^1^; M. Fronza^1^; M. Simone^2^; S. Bova^3^; A. Gallo^4^; R. Lanzillo^5^; S. Malucchi^6^; S. Rasia^7^; G. Lus^8^; V. Torri Clerici^9^; C. Canavese^10^; S. Sotgiu^11^; A. Protti^12^; L. Moiola^13^; A. Berardinelli^14^; P. Annovazzi^15^; M. Viri^16^; M. Amato^17^; E. Cocco^1^; M. Trojano^18^; F. Martinelli Boneschi^19^; S. D'Alfonso^20^; A. Ghezzi^20^; R. Bergamaschi^21^; M. Pugliatti^22^

##### ^1^Department of Medical Sciences and Public Health, University of Cagliari, Cagliari, Italy; ^2^Child Neuropsychiatric Unit, Department of Precision and Regenerative Medicine and Jonic Area, University 'Aldo Moro' of Bari, Bari, Italy; ^3^Child Neurology Unit, Buzzi Children Hospital, Milan, Italy; ^4^Department of Advanced Medical and Surgical Sciences, and 3T MRI‐Center, University of Campania 'Luigi Vanvitelli', Naples, Italy; ^5^Department of Neuroscience, Reproductive Sciences and Dentistry, School of Medicine, University of Naples Federico II, Naples, Italy; ^6^Department of Neurology and Multiple Sclerosis Regional Referral Centre, AOU San Luigi Gonzaga, Orbassano, Turin, Italy; ^7^Multiple Sclerosis Center, ASST ‐ Spedali Civili of Brescia, Brescia, Montichiari, Italy; ^8^Second Division of Neurology, Department of Clinical and Experimental Medicine, University of Campania Luigi Vanvitelli, Naples, Italy; ^9^Neuroimmunology and Neuromuscular Disease Unit, Fondazione IRCCS Istituto Neurologico Carlo Besta, Milan, Italy, ^10^Child and Adolescent Neuropsychiatry Unit, University of Torino, Regina Margherita Hospital, Turin, Italy, ^11^Child Neuropsychiatry Unit, University Hospital of Sassari, Sassari, Italy, ^12^Department of Neuroscience, Niguarda Hospital, Milano, Italy, ^13^Neurology Unit, IRCCS San Raffaele Scientific Institute, Milan, Italy, ^14^Child Neuropsychiatry, IRCCS Mondino Foundation, Pavia, Italy, ^15^Neuroimmunology Unit‐Multiple Sclerosis Center, Hospital of Gallarate, ASST della Valle Olona, Gallarate, Italy, ^16^Department of Child Neurology and Psychiatry, AOU Maggiore della Carità Novara, Novara, Italy, ^17^Department NEUROFARBA, Section of Neurosciences, University of Florence, Florence, Italy; IRCCS Fondazione Don Carlo Gnocchi, Florence, Italy, ^18^Department of Translational Biomedicine and Neurosciences – DiBraiN, University of Bari “Aldo Moro”, Bari, Italy, ^19^Laboratory of Precision Medicine of Neurological Diseases, Department of Health Science; Clinical Neurology Unit, Azienda Socio‐Sanitaria Territoriale Santi Paolo e Carlo and Department of Health Sciences, University of Milan, Milan, Italy, ^20^Department of Health Sciences, University of Eastern Piedmont, Novara, Italy, ^21^Centro Sclerosi Multipla, IRCCS Fondazione Mondino, Pavia, Italy, ^22^Department of Neurosciences and Rehabilitation, University of Ferrara, Ferrara, Italy

**Background and aims:** Investigating the influence of early‐life environmental exposures on adult‐onset multiple sclerosis (MS) is challenging due to the long interval between the timing of exposures and disease onset, as well as the potential for recall biases. We studied the association between breastfeeding and pediatric MS (pedMS) in an Italian cohort.

**Methods:** The PEDIGREE (Pediatric Italian Genetic and Environment Exposure) study is a multicenter case‐control study assessing genetic and environmental interactions influencing MS risk before the age of 18. The study utilizes the PEQ‐IT questionnaire (Pilotto et al., MSJ‐ETC. 2021) was used to record environmental and perinatal exposures. Breastfeeding was classified as reference (≥4 months) or absent/reduced (0–3 months).

**Results:** PEDIGREE data on breastfeeding were available for 96 pedMS cases and 96 controls, of whom, 75 (78.1%) and 55 (57.3%) were females, respectively. The mean (SD) age at study time was 16.8 (2.87) years for cases and 13.6 (5.01) years for controls. Clinical onset occurred at a mean age of 14.1 (2.66) years, with a disease duration of 29.0 (23.33) months. A significant association between pedMS and reduced/absent breastfeeding was observed (OR = 2.04; 95%CI 1.13–3.68;*p* = 0.018). This association was even stronger when exclusively breastfed children were considered (OR = 2.54; 95%CI 1.36–4.75;*p* = 0.003), particularly among females (OR = 2.35; 95%CI 1.10–5.01;*p* = 0.027). These associations remained significant after adjusting for age at study time, preterm birth, and birth order.

**Figure:**
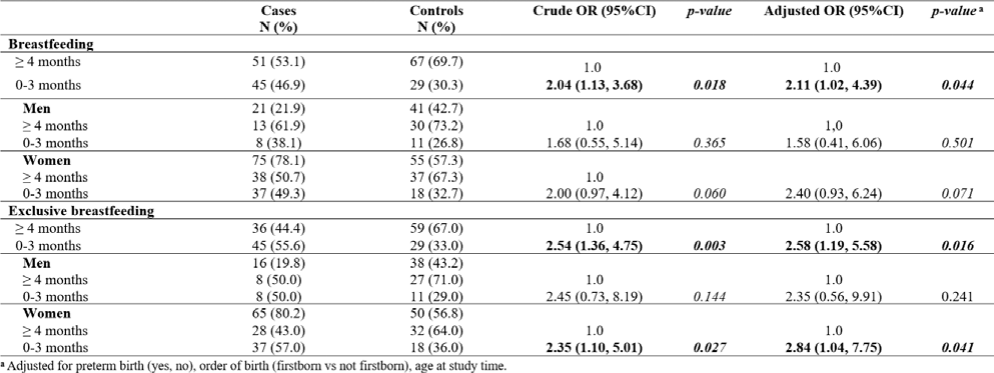
**TABLE 1** Crude and adjusted odds ratios (95 %CIs) for the association between MS and duration of breastfeeding.

**Figure:**
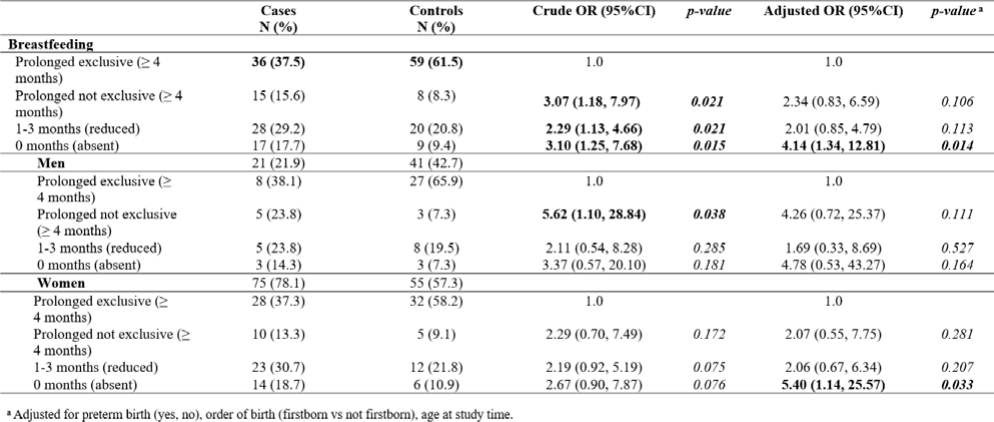
**TABLE 2** Crude and adjusted odds ratios (95 %CIs) for the association between MS and duration of breastfeeding duration and type.

**Conclusion:** Reduced/absent exposure to breastfeeding is associated to a over 2‐fold increased risk for pedMS, particularly in females, suggesting immunological effect of breast milk and sex‐specific immune response to early‐life exposures.

**Disclosure:** All authors declare no conflict of interest in relation to the present study. This study was supported by FISM – Fondazione Italiana Sclerosi Multipla ‐ (grant N. Progetto 2022/R‐Multi/013, Progetto Speciale di Ricerca FISM‐2022, P.I. Angelo Ghezzi).

## EPR‐269

### Paramagnetic rim lesions as a prognostic and predictive biomarker in Tolebrutinib Phase 3 trials for disability outcomes

#### J. Oh^1^; R. J.Fox^2^; D. L.Arnold^3^; S. Syed^4^; W. S.Vargas^5^; Y. Li^5^; T. J.Turner ^4^; D. S.Reich^6^

##### ^1^St. Michael's Hospital, University of Toronto, Toronto, Canada; ^2^Mellen Center for Multiple Sclerosis, Cleveland Clinic, Cleveland, USA; ^3^McGill University, Montréal, Canada, NeuroRx Research, Montréal, Canada; ^4^Sanofi, Cambridge, USA; ^5^Sanofi, Bridgewater, New Jersey, USA; ^6^Translational Neuroradiology Section, National Institute of Neurological Disorders and Stroke, National Institutes of Health, Bethesda, USA

**Background and aims:** Tolebrutinib, a brain‐penetrant Bruton's tyrosine kinase inhibitor that, in phase‐3 trials, reduced disability accumulation by 31% and 29% relative to placebo and teriflunomide in non‐relapsing secondary progressive multiple sclerosis (MS) and relapsing MS, respectively. This post‐hoc analysis evaluated paramagnetic rim lesions (PRLs) at baseline, as prognostic and predictive biomarkers for disability accumulation and treatment response.

**Methods:** HERCULES (NCT04411641), GEMINI 1 (NCT04410978), and GEMINI 2 (NCT04410991) were phase‐3, double‐blind trials of 60mg tolebrutinib once‐daily. HERCULES randomized participants 2:1 (tolebrutinib:placebo); and GEMINI, 1:1 (tolebrutinib:teriflunomide [14mg once‐daily]) with matching placebos. PRLs were evaluated in 39% (437/1131) of HERCULES, and 34% (631/1873) of GEMINI participants from imaging‐capable sites. Effect of tolebrutinib on time to onset of 6‐month confirmed disability worsening (6‐mo‐CDW) was analyzed in participants with PRLs (0, 1–3, ≥4) at baseline, manually identified by susceptibility weighted imaging generated from three‐dimensional gradient echoes (6‐echoes ranging from 4.9‐41ms, 0.8‐mm isotropic resolution).

**Results:** Across both trials, 653 participants (61%) had PRLs, and the proportion of participants with 0, 1‐3, or ≥4‐PRLs at baseline was 40%, 36%, and 24%, respectively. In both trials, the risk of 6‐mo‐CDW increased as a function of baseline PRLs in placebo and teriflunomide comparator groups. Tolebrutinib mitigated 6‐mo‐CDW risk by 54% in participants with ≥4‐PRLs in HERCULES, and 46% and 49% in participants with 1‐3 and ≥4‐PRLs, respectively, in GEMINI. In tolebrutinib‐treated participants with and without PRLs in both trials, the risk of 6‐mo‐CDW was numerically similar.

**Conclusion:** This post‐hoc analysis suggests greater impact of tolebrutinib in those with higher number of PRLs.

**Disclosure:** JO: Consulting and/or speaking (Amgen, Biogen, Eli Lilly and Company, EMD Serono, Novartis, Roche, Sanofi); research (Biogen, Roche). RJF: Consulting (AB Science, Biogen, Bristol Myers Squibb, EMD Serono, Genentech, Greenwich Biosciences, Immunic, INmune Bio, Eli Lilly and Company, Janssen, Novartis, Sanofi, Siemens, TG Therapeutics) and research support (Biogen, Novartis, Sanofi). DLA: Personal compensation for serving as a consultant (Alexion, Biogen, Celgene, Eli Lilly and Company, EMD Serono, Frequency Therapeutics, Genentech, Merck, Novartis, Roche, Sanofi, Shionogi); equity interest (NeuroRx). SS, WSV, YL and TJT: Employees of Sanofi (may hold shares and/or stock options in the company). DSR: Supported by the Intramural Research Program of NINDS, NIH; and additional grant/research support (Abata, Sanofi).

## MS and related disorders 4

## EPR‐270

### Role of free Kappa light chains in CSF as a biomarker in multiple sclerosis

#### A. Roldão Alferes ^1^; R. Martins^2^; Â. Maresch^2^; A. Mendes^2^; R. Cunha^2^; I. Monteiro^1^; R. Machado^1^; I. Correia^1^; C. Cecília Nunes^1^; C. Macário^1^; S. Batista^1^

##### ^1^Serviço de Neurologia, Unidade Local de Saúde de Coimbra, Coimbra, Portugal; ^2^Serviço de Patologia Clínica, Unidade Local de Saúde de Coimbra, Coimbra, Portugal

**Background and aims:** Free Kappa Light Chains (FKLC) in cerebrospinal fluid (CSF) are biomarkers of intrathecal IgG synthesis, similarly to oligoclonal bands (OCBs). Their potential in the diagnosis of Multiple Sclerosis (MS) has been a matter of research. This study aimed to ascertain the diagnostic accuracy of FKLC in CSF for MS and to evaluate potential clinical, imaging, and laboratory correlations.

**Methods:** All patients with a definitive diagnosis who underwent FKLC quantification in CSF from November 2019 to November 2024 were selected. Clinical, demographic, laboratory, and imaging variables were evaluated. Sensitivity, specificity, positive predictive value, and negative predictive value of FKLC and OCBs were calculated, and correlations with clinical, imaging and laboratory variables were tested.

**Results:** Of the 434 patients eligible for analysis, 122 had a definitive diagnosis of MS, 11 had Clinically Isolated Syndrome, 301 had other diagnoses. Using the cut‐off point proposed in the literature of 1 mg/L, FKLC in CSF demonstrated similar sensitivity compared to OCBs (72.93% vs. 72.18%, respectively), while both maintained high specificity (82.33% vs. 92.00%). CSF FKLC levels positively correlated with OCB positivity (*p* < 0.001), with the presence of active MRI lesions (*p* = 0.044), of infratentorial lesions (*p* = 0.001), of spinal cord lesions (*p* = 0.017) and with high lesion load (*p* < 0.001).

**Conclusion:** This study confirms the role of FKLC in CSF as a biomarker for the diagnosis of MS and suggests a possible relationship between FKLC levels and MRI features associated with poor prognosis.

**Disclosure:** The authors have nothing to declare.

## EPR‐271

### Steroid‐refractory tumefactive multiple sclerosis with favorable outcome following natalizumab

#### A. Apostol ^1^; M. Vicol^1^; L. Vlad^2,3^; B. Hodorogea^1^; D. Carp^1^; D. Gramada^1^; C. Gatcan^1,2^; L. Cucu^1,2^; C. Lupu^2^; D. Alexa^1,2^

##### ^1^Neurology Department, Clinical Rehabilitation Hospital, Iasi, Romania; ^2^University of Medicine and Pharmacy “Gr. T. Popa”, Iasi, Romania; ^3^Neurology Department, Clinical Emergency Hospital “Prof. Dr. N. Oblu”, Iasi, Romania

**Background and aims:** Tumefactive demyelinating lesions of the central nervous system (CNS) are an aggressive type of multiple sclerosis (MS) that can resemble tumors or brain abscesses on magnetic resonance imaging (MRI). We report a case of tumefactive MS with inadequate response to intravenous (IV) methylprednisolone and plasma exchange (PLEX), which showed a favorable outcome following natalizumab initiation.

**Methods:** We reviewed the medical history of a 23‐year‐old man who presented with right hemiplegia and global aphasia.

**Results:** Initial MRI revealed a T2‐FLAIR hyperintense tumefactive lesion in the left frontal white‐matter, and a smaller left posterior‐thalamus lesion. MRI spectroscopy suggested a demyelinating etiology (Fig. 1). Cerebrospinal fluid (CSF) analysis showed oligoclonal bands. Blood tests and immunophenotyping of the lymphocytes from the CSF ruled out CNS lymphoma, autoimmune encephalitis, neuromyelitis optica, vasculitis, and sarcoidosis. The patient was started on IV methylprednisolone without a satisfactory response, prompting a 7‐day course of PLEX. This resulted in slow recovery of symptoms. After two months, the patient developed left optic neuritis, with partial recovery following another course of IV methylprednisolone. Subsequent MRI (Fig. 2) showed progression of the fronto‐parietal lesion with perilesional oedema and a new necrotic area in the postcentral parietal gyrus. The patient received the first IV natalizumab infusion, which was well‐tolerated. Follow‐up MRIs revealed a reduction of the lesions (Fig. 3). The patient demonstrated significant clinical improvement.

**Figure:**
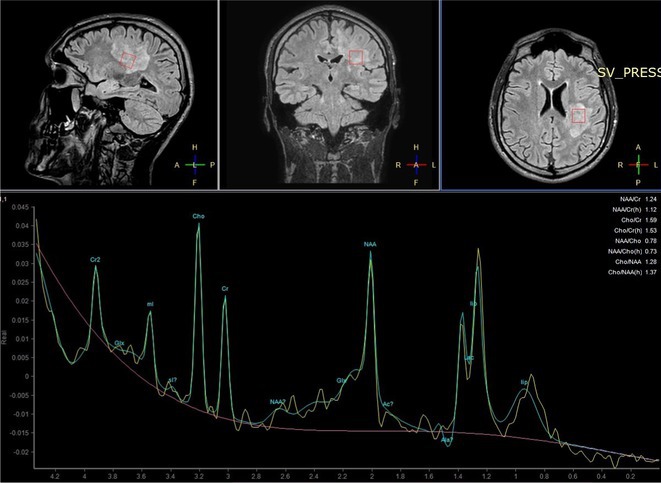
**FIGURE 1** Brain MRI spectroscopy showing white matter lesion measuring 56 x 37 x 32.5 mm, suggesting a demyelinating tumefactive lesion according to metabolites measurings

**Figure:**
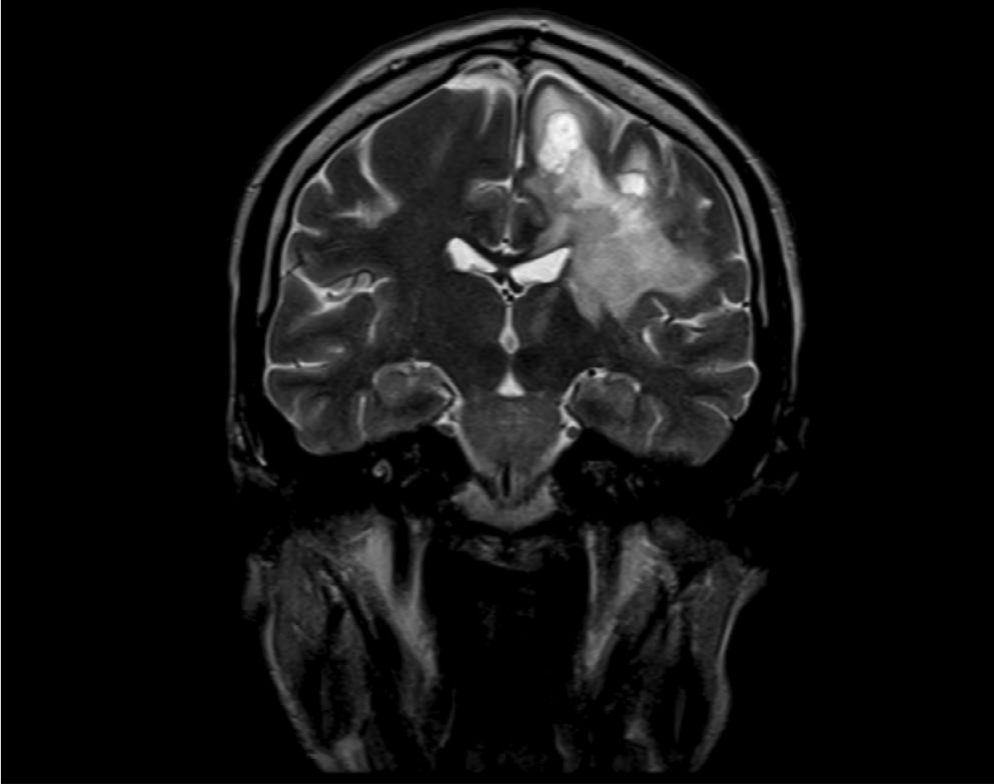
**FIGURE 2** T2 weighted image of the lesion in coronal section before natalizumab initiation, measuring 69 × 42 × 59 mm, with perilesional oedema and necrosis

**Figure:**
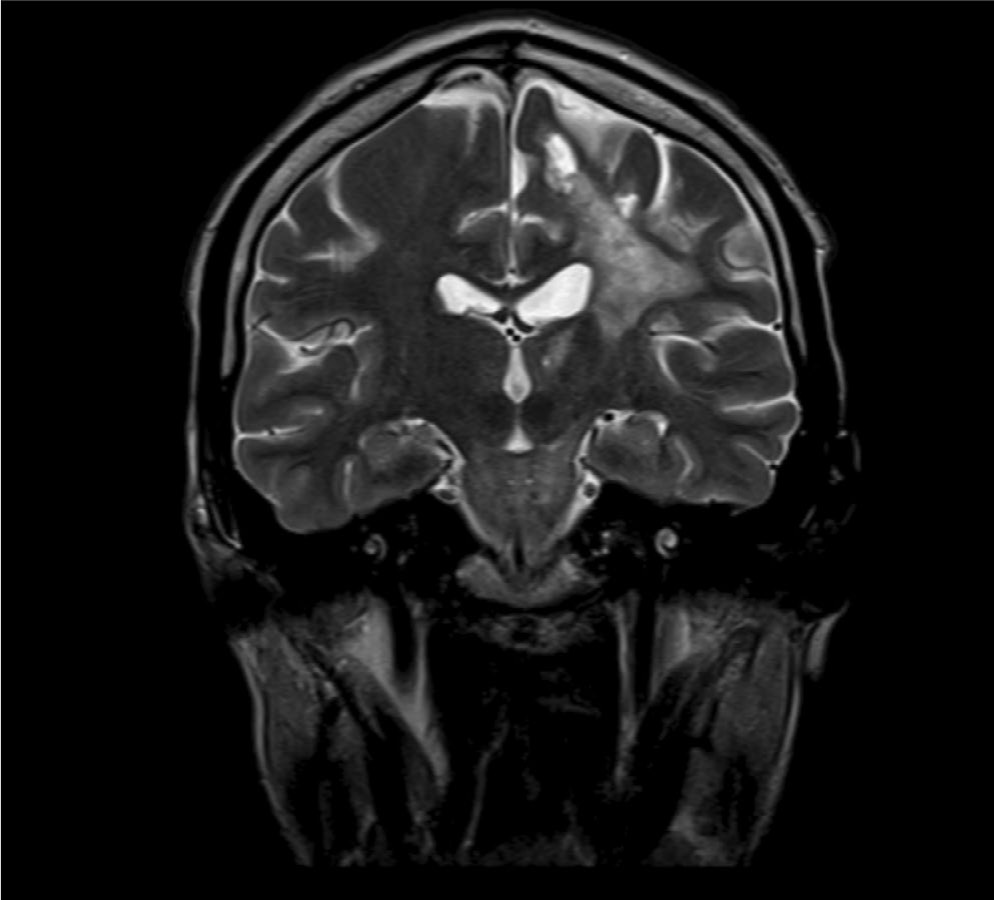
**FIGURE 3** The reduced size of the lesion 58 × 37 × 54 mm after two natalizumab iv infusions

**Conclusion:** The limited case reports regarding the management of tumefactive MS can delay diagnosis and treatment. Further publications and case studies are essential to streamline the diagnostic and therapeutic approach for these patients.

**Disclosure:** Nothing to disclose.

## EPR‐272

### Blood‐based biomarkers of type IV collagen are upregulated in patients with multiple sclerosis

#### A. San Torcuato; S. Holm Nielsen; C. Bramlev; M. Karsdal; D. Leeming; K. Henriksen; J. Bülow Sand

##### Nordic Bioscience, Herlev, Denmark

**Background and aims:** Multiple sclerosis (MS) is characterized as an inflammatory neurodegenerative disease in the central nervous system. The pathological hallmarks of active MS lesions are blood brain barrier disruption, inflammation, and demyelination with axonal damage. Type IV collagen is located in the basement membrane and remodeled in MS lesions. Blood‐based biomarkers reflecting type IV collagen changes may reflect MS pathology.

**Methods:** Biomarkers of type IV collagen reflecting matrix metalloproteinase‐degraded of type IV α1 collagen (nordicC4M), NC1 domain of type IV α2 collagen (nordicCAN), NC1 domain of type IV α3 collagen (nordicTUM), and type IV collagen 7S domain (nordicPRO‐C4), were developed, validated and measured in 23 serum samples from patients with MS (9 RRMS, 14 PPMS) and 11 healthy donors. Differences between MS groups and healthy donors were assessed with a Mann‐Whitney test, and diagnostic accuracy by AUROC. A clinically useful AUROC was set as 0.85.

**Results:** Mean age of the MS patients was 35.7 years (34.8% male), while healthy donors were 41.9 years (55% male). Patients with MS had significantly higher levels of CAN, TUM, and PRO‐C4 compared to healthy donors (*p* = 0.0045, *p* < 0.0001, and *p* = 0.0067, respectively). There were no differences between RRMS and PPMS detected. TUM had an AUROC of 0.996, while C4M, CAN, and PRO‐C4 has AUROC of 0.697‐0.796.

**Conclusion:** We developed and evaluated serum biomarkers reflecting type IV collagen in patients with MS. These may be used to assess patients’ eligibility for targeted treatments and potentially fill a part of the gap for biomarkers in clinical management and trials.

**Disclosure:** Authors are full‐time employees of Nordic Bioscience.

## EPR‐273

### The cause but not the clinical course of early multiple sclerosis runs in the family

#### C. Corsten ^1^; A. Marques^2^; Y. van Hasselt^2^; I. Smets^1^; M. van Luijn^2^; R. Neuteboom^1^; B. Wokke^1^; J. Smolders^3^

##### ^1^Department of Neurology, MS Center ErasMS, Erasmus MC, Rotterdam, the Netherlands; ^2^Department of Immunology, MS Center ErasMS, Erasmus MC, Rotterdam, the Netherlands; ^3^Department of Neurology/Immunology, MS Center ErasMS, Erasmus MC, Rotterdam, the Netherlands. Neuroimmunology Research Group, Netherlands Institute for Neuroscience, Amsterdam, the Netherlands

**Background and aims:** Familial aggregation in multiple sclerosis (MS) can partially be explained by shared genetic and environmental determinants. The effect of familial MS on the disease course after a clinically isolated syndrome (CIS) is uncertain, but could reflect impact of known and unknown factors. We determined the association of familial MS with clinical presentation and early disease course after CIS and explored the mediating role of genetic and environmental risk factors.

**Methods:** CIS participants were included in a prospective cohort study within six months after symptom onset. Family history was assessed at baseline. We evaluated common genetic variants related to vitamin D and body mass index, determined HLA‐DRB1*15:01 carriership and weighted genetic risk scores (wGRS) for MS susceptibility and severity and measured Epstein‐Barr virus Nuclear Antigen‐1 (anti‐EBNA1) Immunoglobulin‐G (IgG) antibodies. Associations with disease course were estimated using Cox regression.

**Results:** Family members with MS were reported for 81/415 (19.5%) of CIS participants. More participants with first‐degree relatives were HLA‐DRB1*15:01 carriers (first 66.7% vs. other‐degree 27.9% vs. no 38.0%, *p* < 0.01). MS susceptibility wGRS were higher in participants with familial MS (7.19( ± 1.22) vs. 7.54( ± 1.17), *p* = 0.03). After controlling for HLA‐status, anti‐EBNA1 IgG titres were higher in familial MS. Baseline characteristics and early disease course were similar between participants with and without familial MS.

**Conclusion:** Our results confirm that familial MS is associated with common genetic and environmental MS risk factors, but does not reflect distinct clinical phenotype or disease course. These data support that MS risk and disease course are mediated by different pathophysiological processes.

**Disclosure:** C.C., A.M. Y.v.H. R.F.N. and B.W. report no competing interests. I.S. has received lecture fees from Merck, Biogen Idec and Sanofi. M.v.L. received research support from EMD Serono, Merck, Novartis, GSK and Idorsia Pharmaceutical Ltd. J.S. received lecture and/or consultancy fee from Biogen, Merck, Novartis, Roche and Sanofi Genzyme.

## EPR‐274

### Ocrelizumab stabilizes fatigue levels in relapsing MS: Key findings from the MoOzaRt study's second interim analysis

#### I. Penner^1^; J. Leemhuis^2^; T. Maier^2^; E. Weber^2^; H. Schreiber ^3^

##### ^1^Department of Neurology, Inselspital, Bern University Hospital, University of Bern, Switzerland; COGITO Center for Applied Neurocognition and Neuropsychological Research, Düsseldorf, Germany; ^2^Roche Pharma AG, Grenzach‐Wyhlen, Germany; ^3^Neurological Practice Center, Ulm, Germany

**Background and aims:** Fatigue is considered the most common and one of the most debilitating symptoms in multiple sclerosis (MS). MoOzaRt aimed to assess the impact of ocrelizumab on patient‐reported long term (trait) and transient (state) fatigue in patients with relapsing forms of MS (RMS) on therapy with ocrelizumab.

**Methods:** The ongoing non‐interventional MoOzaRt study (ISRCTN55332718) recruited 272 RMS patients initiating ocrelizumab therapy. The primary combined endpoint is a clinically meaningful reduction in trait fatigue (≥9 points) or stabilization from baseline (range  ± 8 points), measured by the Fatigue Scale for Motor and Cognitive Functions (FSMC) total score over 24 months. Secondary endpoints include state fatigue (Visual Analogue Scale), Expanded Disability Status Scale (EDSS), Patient Reported Outcomes and safety.

**Results:** The second interim analysis (data cut‐off Dec 16, 2024) included a total of 193 patients (69.9% female, mean age 38.3, SD 10.8 years with a mean EDSS score of 2.43, SD 1.50; table 1), among them 186 patients in the analysis set with a follow‐up of at least 12 months. FSMC total scores remained stable or were reduced in 91.4% of patients over 24 months‐follow‐up. Of those, 28% displayed a clinically meaningful improvement. Patient numbers in FSMC categories over time are presented in table 2.

**Figure:**
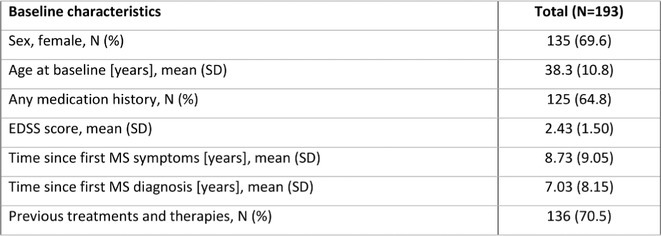
TABLE 1

**Figure:**
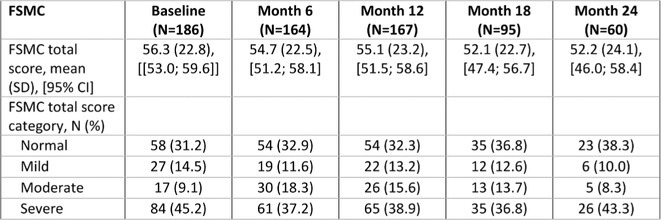
TABLE 2

**Conclusion:** The second interim analysis of the MoOzaRt study showed stable FSMC total scores in a very high proportion of patients after 24 months, with a trend toward more patients without fatigue. Further analyses will specify long‐term impact of ocrelizumab on fatigue in RMS patients and factors influencing it.

**Disclosure:** IKP: Almirall, Biogen, BMS, Celgene, Genzyme, Janssen, Merck, Novartis, Roche, Teva // speakers bureau or advisory board, consulting fees; The German MS Society, Celgene, Novartis, Roche, Teva // research grants. JL: Roche // employee TM: Roche // employee EW: Roche // employee HS: Almirall, Biogen, BMS, Genzyme, Janssen, Merck, Novartis, Roche, Teva // speakers bureau or advisory board, consulting fees, travel reimbursement; Biogen, Novartis, Teva // research grants; Biogen, Novartis, Roche // data monitoring or steering committees.

## EPR‐275

### Relationship between SDMT and brain volume following short course cladribine tablets: Results from MAGNIFY‐MS

#### Nicola De Stefano ^1^; Heinz Wiendl^2^; Patrick Vermersch^3^; Tobias Derfuss^4^; Xavier Montalban^5^; Anat Achiron^6,7^; Suzanne Hodgkinson^8^; Andrew Chan^9^; Alexandre Prat^10^; Letizia Leocani^11–13^; Klaus Schmierer^14,15^; Finn Sellebjerg^16,17^; Caroline Petit^18^; Alexander Helman^19^; Lidia Gardner^19^; Frederik Barkhof^20,21^

##### ^1^Department of Medicine, Surgery and Neuroscience, University of Siena, Siena, Italy; ^2^Department of Neurology with Institute of Translational Neurology, University of Münster, Münster, Germany; ^3^Univ. Lille, Inserm U1172 LilNCog, CHU Lille, FHU Precise, Lille, France; ^4^Department of Neurology, University Hospital Basel, Basel, Switzerland; ^5^Department of Neurology‐Neuroimmunology, Centre d’Esclerosi Múltiple de Catalunya (Cemcat), Hospital Universitario Vall d’Hebron, Barcelona, Spain; ^6^Multiple Sclerosis Center, Sheba Academic Medical Center, Ramat Gan, Israel; ^7^Faculty of Medicine, Tel‐Aviv University, Israel; ^8^Ingham Institute for Applied Medical Research, University of New South Wales Medicine and Liverpool Hospital, Sydney, NSW, Australia; ^9^Department of Neurology, Inselspital, Bern University Hospital, University of Bern, Bern, Switzerland; ^10^Department of Neurosciences, Université de Montréal, Montréal, QC, Canada; ^11^University Vita‐Salute San Raffaele, Milan, Italy; ^12^Scientific Institute IRCCS San Raffaele, Milan, Italy; ^13^Department of Neurorehabilitation Science, Casa di Cura Igea, Milan, Italy; ^14^The Blizard Institute, Centre for Neuroscience, Surgery and Trauma, Barts and The London School of Medicine & Dentistry, Queen Mary University of London, London, UK; ^15^Clinical Board Medicine (Neuroscience), The Royal London Hospital, Barts Health NHS Trust, London, UK; ^16^Danish MS Center, Department of Neurology, Copenhagen University Hospital ‐ Rigshospitalet, Glostrup, Denmark; ^17^Department of Clinical Medicine, University of Copenhagen, Copenhagen, Denmark; ^18^Merck Healthcare KGaA, Darmstadt, Germany; ^19^EMD Serono Research & Development Institute, Inc., Billerica, USA, an affiliate of Merck KGaA; ^20^Department of Radiology and Nuclear Medicine, Amsterdam UMC, Vrije Universiteit, Amsterdam, the Netherlands; ^21^Queen Square Institute of Neurology and Centre for Medical Image Computing, University College London, London, UK

**Background and aims:** Excessive brain atrophy and cognitive decline can predict disease progression in multiple sclerosis (MS). We explored the relationship between total and regional brain volume (BV) and Symbol Digit Modalities Test (SDMT) scores in MAGNIFY‐MS (NCT03364036) participants treated with cladribine tablets (CladT).

**Methods:** BV and SDMT were assessed at regular intervals over 2 years. The relationship between total and regional (gray matter [GM], deep GM [dGM], and white matter [WM]) BV with SDMT was analyzed using a generalized linear mixed‐effects model. SDMT scores (absolute) and 4‐point improvement, worsening, or stable status were compared between baseline and months (M)12 and 24. Participants were categorized into low (< 53.5) or high (≥53.5) baseline SDMT groups.

**Results:** MAGNIFY‐MS included 270 participants (66.7% female; 43.7% aged > 40 years). Following CladT initiation, annualized brain atrophy rates were similar to those reported for the normal aging population. The median (quartile [Q]1;Q3) annualized BV percentage change compared with baseline was ‐0.452 (‐0.880;‐0.165) at M12 and ‐0.420 (‐0.719;‐0.157) at M24. SDMT changes are shown in Table 1. Significant relationships were observed between total BV, GM, and SDMT (Table 2). dGM volume had the strongest association with SDMT (35.5%, *p* = < 0.0001). In the low baseline SDMT group, GM and dGM were significantly linked to SDMT (Table 2).

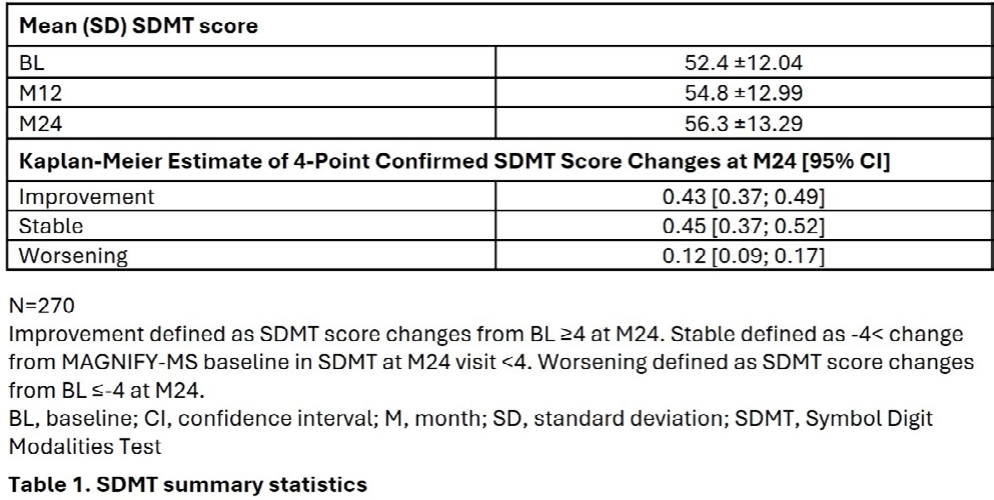

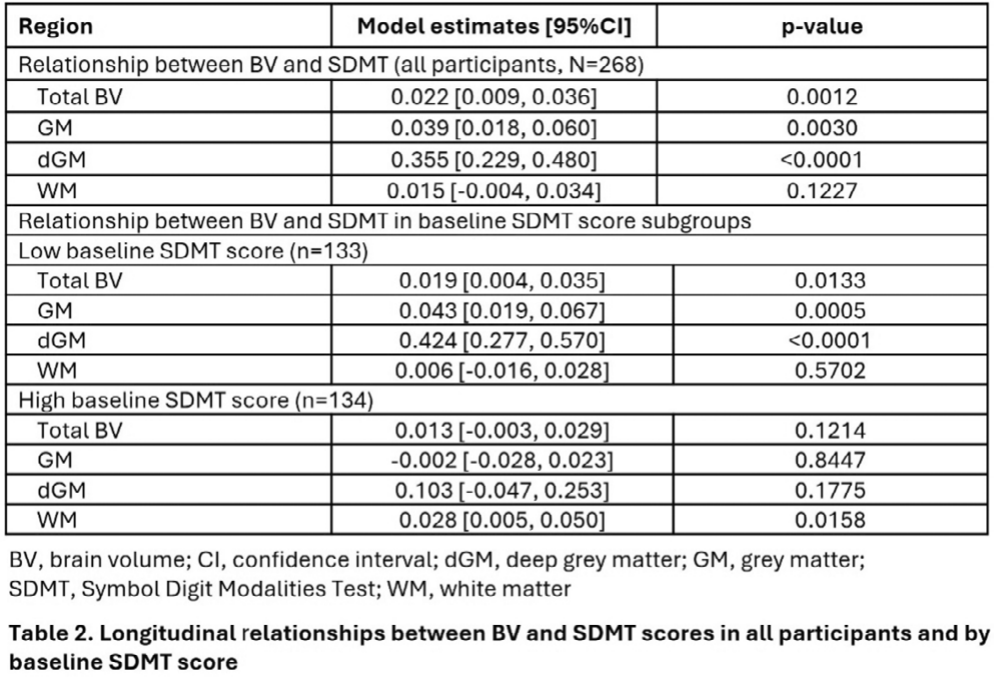

**Conclusion:** As expected, total or regional brain atrophy coincided with lower SDMT scores. Participants with low brain atrophy also showed no cognitive decline. This highlights the potential of CladT in supporting cognitive health in MS patients.

**Disclosure:** NDS has received honoraria from Biogen, Celgene (Bristol Myers Squibb), EMD Serono Research & Development Institute, Inc., Billerica, MA, USA, an affiliate of Merck KGaA, Genzyme, Immunic, Novartis, Roche, and Teva for consulting services, speaking, and travel support. He serves on advisory boards for Biogen, Genzyme, Immunic, Merck, Novartis, and Roche, and has received research grant support from the Italian MS Society. HW is member of scientific advisory boards/steering committees for Bayer, Biogen, Merck, Novartis, Roche, Sanofi, and Teva. He received speaker honoraria and travel support from Bayer, Biogen, CSL Behring, EMD Serono Research & Development Institute, Inc., Billerica, MA, USA, an affiliate of Merck KGaA, Fresenius Medical Care, Merck, Omniamed, Novartis, Sanofi, and Teva. He received compensation as a consultant from Biogen, Merck, Novartis, Omniamed, Roche, and Sanofi. He has received research support from Bayer, Biogen, Merck, Novartis, Sanofi, and Teva, as well as the German Ministry for Education and Research (BMBF), German Research Foundation (DFG), Else Kröner Fresenius Foundation, Fresenius Foundation, Hertie Foundation, NRW Ministry of Education and Research, Interdisciplinary Center for Clinical Studies (IZKF) Münster, and RE Children’s Foundation. PV has received honoraria or consulting fees from AB Science, Ad Scientiam, Biogen, Celgene (Bristol Myers Squibb), Imcyse, Janssen (J&J), Merck, Novartis, Roche, Sanofi, and Teva; and research support from Novartis, Roche, and Sanofi. TD serves on scientific advisory boards for Actelion (Janssen/J&J), Bayer, Biogen, Celgene (Bristol Myers Squibb), GeNeuro, MedDay, Merck, Mitsubishi Pharma, Novartis, Roche, and Sanofi; has received funding for travel and/or speaker honoraria from Bayer, Biogen, Merck, Novartis, Roche, and Sanofi; and receives research support from Actelion, the European Union, Novartis, Roche, the Swiss MS Society, and the Swiss National Foundation. XM has received compensation for lecture honoraria and travel expenses, participation in scientific meetings, clinical trial steering committee membership, or clinical advisory board participation in recent years from Abbvie, Actelion, Alexion, Bial PD, Biogen, Bristol‐Myers Squibb/Celgene, EMD Serono Research & Development Institute, Inc., Billerica, MA, USA, an affiliate of Merck KGaA, Genzyme, Hoffmann‐La Roche, Immunic Therapeutics, Janssen Pharmaceuticals, Medday, Medscape, Merck, Mylan, Nervgen, Neuraxpharm, Novartis, Peervoice, Samsung‐Biosys, Sandoz, Sanofi‐Genzyme, Teva Pharmaceutical, TG Therapeutics, Excemed, ECTRIMS, MSIF, and NMSS or any of their affiliates. AA has received over the last 5 years honoraria or consulting fees for participating in advisory boards related to clinical trial design, trial steering committees, and data and safety monitoring committees from Biogen, Bristol Myers Squibb, Merck, Novartis, Roche, and Sanofi; and research support for investigator‐initiated trials and MS patients’ benefits activities from Biogen, Bristol Myers Squibb, Merck, Novartis, Roche, and Sanofi. SH serves on advisory boards for Bayer, Biogen, Merck, Novartis, Roche, and Sanofi. She has received money for travel and speaker honoraria from Bayer, Biogen, Merck, Novartis, Roche, and Sanofi. AC has received speakers’/board honoraria from Actelion (Janssen/J&J), Almirall, Bayer, Biogen, Celgene (Bristol Myers Squibb), Merck, Novartis, Roche, Sanofi, and Teva, all for hospital research funds. He received research support from Biogen, Sanofi, and UCB, the European Union, and the Swiss National Foundation. He serves as associate editor of the European Journal of Neurology, on the editorial board for Clinical and Translational Neuroscience, and as topic editor for the Journal of International Medical Research. AP has received speaking honoraria and travel expenses for participation in scientific meetings, has been a steering committee member of clinical trials or participated in advisory boards of clinical trials in the past years, and/or received operating grants from Alexion, Bayer, Biogen, Celgene (Bristol Myers Squibb), EMD Serono Research & Development Institute, Inc., Billerica, MA, USA, an affiliate of Merck KGaA, Novartis, Roche, Sanofi, and Teva. LL has received honoraria for consulting services or speaking activities from Biogen, Bristol Myers Squibb, Janssen‐Cilag, Merck, Novartis, and Roche; and research support from Biogen, Merck, and Novartis. KS has received research support, through Queen Mary University of London, from Biogen, Merck, Novartis, and Sandoz; speaking honoraria from, and/or served in an advisory role for, Biogen, EMD Serono Research & Development Institute, Inc., Billerica, MA, USA, an affiliate of Merck KGaA, Merck, Neuraxpharm, Novartis, Roche, Sanofi, and Teva; and remuneration for teaching activities from AcadeMe and Medscape. FS has served on scientific advisory boards, been on the steering committees of clinical trials, served as a consultant, received support for congress participation, received speaker honoraria, or received research support for his laboratory from Biogen, Celgene (Bristol Myers Squibb), EMD Serono Research & Development Institute, Inc., Billerica, MA, USA, an affiliate of Merck KGaA, Merck, Novartis, Roche, Sanofi, and Teva. CP is an employee of Merck Healthcare KGaA, Darmstadt, Germany. LG and AH are employees of EMD Serono Research & Development Institute, Inc., Billerica, MA, USA, an affiliate of Merck KGaA. FB is supported by the NIHR Biomedical Research Centre at UCLH and is a steering committee or Data Safety Monitoring Board member for ATRI/ACTC, Biogen, Merck, and Prothena. He is a consultant for Celltrion, Combinostics, IXICO, Janssen (J&J), Merck, Rewind Therapeutics, and Roche. Research agreements with Biogen, GE Healthcare, Merck, and Roche. Co‐founder and shareholder of Queen Square Analytics Ltd. Funding: This study was sponsored by Merck (CrossRef Funder ID: 10.13039/100009945). Joe Ward of inScience Communications, Springer Healthcare, UK, provided medical writing support, which was funded and supported by Merck in accordance with the Good Publication Practice 2022 Guidelines.

## EPR‐276

### Plasma neurofilament light chain associates with cognitive but not with patient‐reported outcomes in multiple sclerosis

#### V. Nicolella ^1^; F. Novarella^1^; F. Falco^1^; C. Polito^2^; R. Sirica^2^; E. La Civita^2^; V. Criscuolo^3^; G. Corsini^1^; A. Spiezia^1^; A. Carotenuto^1^; M. Petracca^4^; R. Lanzillo^1^; G. Castaldo^3^; V. Brescia Morra^1^; D. Terracciano^2^; M. Moccia^3^

##### ^1^Department of Neuroscience, Reproductive Science and Odontostomatology, Federico II University of Naples, Naples, Ital; ^2^Department of Translational Medical Sciences, Federico II University of Naples, Naples, Italy; ^3^Department of Molecular Medicine and Medical Biotechnology, Federico II University of Naples, Italy; ^4^Department of Human Neuroscience, Sapienza University of Rome, Rome, Italy

**Background and aims:** To explore associations between plasma neurofilament light chain (pNfL) and cognition and patient‐reported outcome measures (PROMs) in multiple sclerosis (MS)

**Methods:** In this cross‐sectional study, we included 211 people with MS (PwMS) and collected EDSS, education, cognition (SDMT, CVLT and BVMT), Modified Fatigue Impact Scale (MFIS), Beck Depression Inventory (BDI‐II), Beck Anxiety Inventory (BAI), and Pittsburgh Sleep Quality Index (PSQI). pNfL was evaluated using fully automated chemiluminescent enzyme immunoassay.

**Results:** On linear regression models, higher educational attainments were associated with lower pNfL (high school: Coeff = ‐0.22; 95%CI = ‐0.41, ‐0.04; *p* = 0.019; university: Coeff = ‐0.22; 95%CI = ‐0.42, ‐0.02; *p* = 0.030). On logistic regression models, each EDSS step was associated with 56% higher probability of pNfL above normality values (OR = 1.56; 95%CI = 1.23, 1.98; *p* < 0.001), and impaired SDMT with 2.5 higher probability of pNfL above normality values (OR = 2.50; 95%CI = 2.20, 5.21; *p* = 0.014). No associations were found for BDI‐II, MFIS, BDI‐II, BAI, PSQI

**Conclusion:** Neuro‐axonal injury can express clinically into worse disability and worse attention and processing speed in PwMS, and could be mitigated by increased resilience, as reflected by higher educational attainment. Included PROMs were not associated with pNfL, and could possibly not be sufficiently sensitive to neuro‐axonal injury or simply reflect other MS pathophysiologies.

**Disclosure:** Valerio Nicolella discloses travel/meeting expenses from Alexion. Antonio Carotenuto has received research grants from Almirall and ECTRIMS‐ MAGNIMS and honoraria from Almirall, BMS Celgene, Biogen, Roche, Sanofi‐Genzyme, Merck, Ipsen and Novartis. Maria Petracca discloses travel/meeting expenses from Novartis, Janssen, Roche, Merck and Alexion; speaking honoraria from HEALTH&LIFE S.r.l., AIM Education S.r.l., Biogen, Novartis and FARECOMUNICAZIONE E20; honoraria for consulting services and advisory board participation from Biogen; research grants from Baroni Foundation and the Italian Ministry of University and Research (PRIN 2022LP5X2E). Vincenzo Brescia Morra and Roberta Lanzillo received research grants from the Italian MS Society, and Roche, and honoraria from Bayer, Biogen, BMS Celgene, Merck, Mylan, Novartis, Roche, Sanofi‐Genzyme, and Teva Marcello Moccia is editorial board member of Neurology (AAN, MN, US), and the Multiple Sclerosis Journal (Sage, UK); has received research grants from MUR PNRR Extended Partnership (MNESYS no. PE00000006, DHEAL‐COM no. PNC‐E3‐2022‐23683267), ECTRIMS‐MAGNIMS, UK MS Society, and Merck; and has received honoraria from Abbvie, Biogen, BMS Celgene, Ipsen, Janssen, Merck, Novartis, Roche, Sanofi‐Genzyme.

## EPR‐277

### Immunoglobulin dynamics in multiple sclerosis patients switching from ocrelizumab or rituximab to ofatumumab

#### K. Mertes; L. Woitschach; W. Brownlee

##### Queen Square MS Centre, London, UK

**Background and aims:** Immunoglobulin (Ig) M levels fall over time in multiple sclerosis (MS) patients treated with both ocrelizumab and ofatumumab, whereas falling IgG levels have only been reported with ocrelizumab. We wanted to investigate immunoglobulin dynamics in patients switching from high‐dose, pulsed anti‐CD20 therapies to ofatumumab in a large, real‐world cohort.

**Methods:** We identified consecutive patients with MS switching from ocrelizumab or rituximab to ofatumumab at our center between 2022 and 2024. Serum immunoglobulins were measured every 6 months and mixed effects models used to estimate the rate of change in IgG and IgM levels over time, adjusting for age and sex.

**Results:** We studied 116 patients (mean age 40.6 years, 73% female) switching to ofatumumab from ocrelizumab (*n* = 112) or rituximab (*n* = 4). The most common reason for treatment switch was convenience. At the time of ofatumumab initiation, 19 (16%) patients had IgG levels less than the lower limit of normal (< LLN, < 7.0g) and 34 (29%) patients had IgM < LLN (< 0.4g/L). Over a mean follow‐up of 1.62 years, IgG levels were stable (mean change ‐0.006g/L/month, *p* = NS), whereas IgM levels fell (mean change ‐0.005g/L/month, *p* < 0.01). The findings were similar in patients with and without hypogammaglobulinemia at the time of ofatumumab initiation.

**Conclusion:** In patients switching from pulsed, high‐dose antiCD20 therapies to ofatumumab, IgG levels remain stable over the short‐term while IgM levels decline. Longer follow‐up is needed to determine whether switching to ofatumumab is an effective strategy for managing low IgG levels in MS patients treated with ocrelizumab or rituximab.

**Disclosure:** Ms Mertes has nothing to disclose. Ms Woitschach has nothing to disclose. Dr Brownlee has acted as a consultant and/or received speaker honoraria for educational activities for Astra‐Zeneca, Biogen, Juvise, Merck, Novartis, Roche, Sandoz and Sanofi.

## EPR‐278

### Measuring spasticity and treatment in MS: a prospective register pseudo‐trial

#### J. Rodgers ^1^; R. Nicholas^2^; R. Middleton^1^

##### ^1^Data Science, School of Medicine, Swansea University, Wales; ^2^Department of Brain Sciences, Faculty of Medicine, Imperial College, London, UK

**Background and aims:** Spasticity, characteriszed by muscle stiffness and spasms, is common in people with MS impacting mobility and quality of life. UKMS Register participants have completed the Multiple Sclerosis Impact Scale (MSIS‐29) since 2011. There are 2 sub‐items related to severity of stiffness and spasms. Typically MSIS‐29 is scored by total/psychological/physical components. We developed an MSIS‐29 based metric to measure spasticity change (MSIS‐spast).

**Methods:** We created a pseudo‐trial of those answering MSIS‐29 ≥3 times consecutively ('streaks’), follow‐up within 270 days without omission. First visit within 180 days of starting treatment: Baclofen or Sativex. Time to (Kaplan‐Meier estimator) MSIS29‐physical (6‐point) and MSIS‐spast sum (1‐point) changes (+/‐) were used to gauge treatment effect. Using propensity score matching we created controls for treatment groups. Matching: visit number, age, gender, MS‐Type, and MSIS‐spast, MSIS‐physical, and MSIS‐psychological scores. Cox proportional hazards modeling was used to control for confounders.

**Results:** 283 receiving Baclofen were 1:1 matched to 267 controls; Sativex was 1:3 matched (*n* = 48:172) Lack of complete streaks affected ratios. In both treatments there was no significant MSIS‐physical effect. Baclofen (*p* = 0.079) and Sativex (*p* = 0.055) showed a trend toward spasticity improvement. Controlling for confounders Baclofen had a hazard ratio for spasticity improvement of 1.29 (95% CI: 1.04–1.61; *p* = 0.021). There was no significant treatment effect for Sativex.

**Conclusion:** Using MSIS‐spast components proved more useful than MSIS‐physical in determining spasticity change in a treated population. Baclofen showed significant treatment effect, Sativex less so. This may be due to lack of study power or treatment resistance.

**Disclosure:** JR RM and RN are supported by the UK Multiple Sclerosis Society RN is supported by Multiple Sclerosis Trials Collaboration (MSTC) and the Berkeley Foundation.

## Neuro‐oncology and palliative care

## EPR‐279

### Progressive supranuclear palsy – Are we planning for the future? A North‐East UK perspective

#### A. Coldstream ^1^; J. Roelofs^2^; L. Wiblin^1^

##### ^1^Neurosciences Department, James Cook University Hospital, Middlesbrough, UK; ^2^Neurology Specialist Trainee, North East Region, UK

**Background and aims:** Progressive supranuclear palsy (PSP) is a rare neurodegenerative disorder, with an average life expectancy from symptom onset of around seven years (Testa et al., 2001). The intention of this work was to look at the advanced care planning that occurred for patients admitted to hospital with a diagnosis of PSP.

**Methods:** Data was collected for patients admitted through James Cook Hospital's Accident and Emergency department with a coded diagnosis of PSP on the discharge summary from 2018 to 2022. 91 admissions, and 56 individual patients were identified. Non‐PSP patients were removed. Data was recorded for reason for admission, length of stay, advanced care planning completed, and survival at 12 months.

**Results:** Half of all admissions were related to infection or mobility. 66% of admissions had the patient return to their own home on discharge, with 24% discharged to a care home, and 10% dying. 26% had a do not attempt resuscitation order in place before admission rising to 44% following admission. 15% had an emergency healthcare plan on admission, rising to 16% on discharge. For any single admission, there was a 50% chance the patient died within 12 months.

**Conclusion:** The primary conditions that lead to hospital admission are falls and infections, with a high chance of death in the following 12 months after admission. There is a lack of advanced care planning done, and hospital admissions can be used to identify at risk, frail patients, enabling them to have more agency and input into their future care.

**Disclosure:** Nothing to disclose.

## EPR‐280

### Neurology training and palliative care: Insights from Iberian Peninsula Residents

#### A. Costa ^1^; C. Coronado^2^; S. Lima^1^; M. Soares^3^; J. Alves^4^; S. Bernardo^5^; A. Fernandes^6^; A. Paula^7^; L. Rufo Costa^8^; D. Valente^9^; Â. Fonseca^10^; T. Jesus^11^; M. Sainda Duarte^12^; R. Lopes^13^; J. Bandeira Costa^14^; R. Gagigal^15^; S. Lopes^16^; M. Miranda^17^; A. Cordeiro^18^; D. Oliveira^19^; C. Fernandes^20^; M. Serôdio^21^; I. Vidal^22^; R. Mendes Franco^23^; A. Graça Velon^1^

##### ^1^Department of Neurology, Local Health Unit of Trás‐os‐Montes and Alto Douro, Vila Real, Portugal; ^2^Department of Neurology, Puerta del Mar University Hospital, Cádiz, Spain; ^3^Department of Neurology, Local Health Unit of São José, Lisbon, Portugal; ^4^Department of Neurology, Local Health Unit of Arrábida, Setúbal, Portugal; ^5^Department of Neurology, Local Health Unit of Amadora/Sintra, Amadora, Portugal; ^6^Department of Neurology, Local Health Unit of São João, Porto, Portugal; ^7^Department of Neurology, Local Health Unit of Santa Maria, Lisbon, Portugal; ^8^Department of Neurology, Local Health Unit of Alto Minho, Viana do Castelo, Portugal; ^9^Department of Neurology, Local Health Unit of Algarve, Faro, Portugal, ^10^Department of Neurology, Local Health Unit of Matosinhos, Matosinhos, Portugal, ^11^Department of Neurology, Local Health Unit of Estuário do Tejo, Vila Franca de Xira, Portugal, ^12^Department of Neurology, Local Health Unit of Loures‐Odivelas, Loures, Portugal, ^13^Department of Neurology, Local Health Unit of Santo António, Porto, Portugal, ^14^Department of Neurology, Francisco Gentil Portuguese Institute of Oncology, Lisbon, Portugal, ^15^Department of Neurology, Local Health Unit of Gaia and Espinho, Vila Nova de Gaia, Portugal, ^16^Department of Neurology, Local Health Unit of Braga, Braga, Portugal, ^17^Functional Neurology Unit, Cascais Hospital Dr. José de Almeida, Cascais, Portugal, ^18^Department of Neurology, Local Health Unit of Almada‐Seixal, Almada, Portugal, ^19^Department of Neurology, Local Health Unit of Entre‐Douro and Vouga, Santa Maria da Feira, Portugal, ^20^Department of Neurology, Local Health Unit of Coimbra, Coimbra, Portugal, ^21^Department of Neurology, Local Health Unit Lisboa Ocidental, Lisbon, Portugal, ^22^Department of Neurology, Divino Espírito Santo Hospital, Ponta Delgada, Portugal, ^23^Department of Neurology, Dr. Nélio Mendonça Hospital, Funchal, Portugal

**Background and aims:** Palliative care (PC) is essential in managing severe chronic and progressive neurological diseases. Its importance is increasingly recognized by neurologists and medical educators worldwide. This study evaluates neurology residents’ perceptions of PC's relevance in their training and assesses their self‐reported competencies in this field.

**Methods:** An online questionnaire, developed via Google Forms and based on Mehta et al. (2018), was distributed to neurology residents in Portugal and Spain. It comprised 11 questions, including two with 10 Likert‐scale items each and nine multiple‐choice questions. Residents evaluated the importance of various palliative care topics in postgraduate training and self‐assessed their competencies in these areas. Each Likert item offered five response options, scored from 1 to 5. For each participant, two scores were calculated by summing the responses to the 10 Likert items in each question. These scores were analyzed using descriptive and comparative statistical methods.

**Results:** A total of 127 responses were collected (71% from Portugal), representing neurology residents at various training stages and regions across Portugal and Spain. Approximately 28% referred patients to palliative care at least monthly, while only 15% had access to PC education during residency. “Communication” was rated the most important training topic (mean = 4,65), while “ethical and legal issues” received the lowest self‐assessed competency score (mean = 2,22). Around 95% supported integrating palliative care into the residency curriculum, and the mean score reflecting the perceived importance of PC was significantly higher in this subgroup (*p* = 0,008).

**Conclusion:** These findings highlight residents' recognition of palliative care's relevance and training benefits.

**Disclosure:** Nothing to disclose.

## EPR‐281

### Advanced care planning of patients with neurologic diseases. First year of experience at a specialized outpatient clinic

#### A. Suarez Plaza; I. Zamarbide Capdepon; L. Esteban; A. Gandara del Castillo; J. Pardo Moreno

##### Hospital Universitario Fundacion Jimenez Diaz

**Background and aims:** Advanced Care Planning (ACP) involves setting objectives and having discussions with patients and family members/caregivers about care preferences especially ‐but not only‐ at the end of life in the context of chronic diseases. Managing patients with neurologic diseases in this setting impose specific challenges that require skilled care. The aim of this study was to describe data gathered in a specialized neurology ACP outpatient clinic.

**Methods:** We reviewed the medical records of patients seen during the first year of our neurology ACP clinic. We described demographic data, diagnoses, cognitive, functional, and motor severity scales, indicators of palliative care needs, mortality, end‐of‐life preferences and care outcomes.

**Results:** We attended 86 patients with a mean age of 78 years. The most common diagnoses were dementia, stroke, and multiple sclerosis. 68% had severe cognitive impairment, and 88% presented moderate‐to‐severe dependency. The most prevalent indicator of advanced palliative care needs was dysphagia (58%). After our consultation, visits to the emergency department were reduced from 48% to 18%, and hospital admissions from 33% to 13%. Out of the 23 patients who died, the majority (75%) passed away in their preferred place.

**Conclusion:** Most of our patients presented degenerative diseases involving cognitive, language and decision‐making impairment, which highlights the importance of addressing preferences, needs, and strategies from an early stage and with the support of caregivers. Our clinic helped neurologic patients achieve their care preferences and reduced futile interventions.

**Disclosure:** Nothing to disclose.

## EPR‐282

### End‐of‐life and palliative sedation in the neurology inpatient unit of a tertiary hospital

#### C. Morales‐González; Á. Lambea‐Gil; I. Barroeta‐Espar; G. Olmedo‐Saura; L. Prats‐Sánchez; A. Ramos‐Pachón; V. Ros‐Castelló; A. Vesperina‐Castro; A. Martínez‐Domeño

##### Department of Neurology, Hospital de la Santa Creu I Sant Pau, Barcelona, Spain

**Background and aims:** Neurology departments report higher in‐hospital mortality rates compared with other units. Given the complexity of neurological diseases, addressing palliative sedation (PS) at the end of life (EoL) is critical, yet understudied. This study aimed to describe the use of PS at the EoL in patients who died in a Neurology Inpatient Unit (NIU).

**Methods:** An observational, retrospective clinical audit was conducted in the NIU of a tertiary hospital in Spain. Demographic, medical history, and therapeutic data were reviewed for patients who died in 2024. Descriptive and univariable analyses were performed.

**Results:** Among 45 patients (mean age 79.7 ± 11.9 years; 46.7% women) who died in 2024, 40(88.9%) deaths were related to stroke. PS was administered to 43 (95.6%; 40 via continuous infusion) with a median duration of 1day (IQR 1–2) from initiation to death. Only 3 (6.7%) had advance directives, while 14 (31.1%) had documented some form of EoL conversation with relatives. Dyspnoea was the most frequently registered refractory symptom (39.5%), although 15 patients (34.9%) lacked documented symptoms. The principal sedatives used were midazolam (97.7%, median dose 45mg/24h, IQR 26‐60) and morphine (97.7%, median dose 40mg/24h, IQR 30‐50); while anticholinergics were used in 40(93.0%). 10 patients (23.8%) had concurrent active treatments at the moment of PS.

**Conclusion:** Nearly all patients who died in the NIU received PS, yet only 31.1% had documented EoL conversations with proxies, indicating a need for better communication and documentation. Additionally, the frequent use of opioids despite guideline recommendations underscores the necessity for more standardized PS protocols in neurology settings.

**Disclosure:** Nothing to disclose.

## EPR‐283

### Identification of a new travelling oncoprotein associated with poor prognosis in high grade gliomas

#### E. Vigneul

##### Laboratory of Neural Differentiation (NEDI), Animal Molecular and Cellular Biology Group, Louvain Institute of Biomolecular Science and Technology, Université Catholique de Louvain, Louvain‐La‐Neuve, Belgium

**Background and aims:** Considering the various roles of Homeodomain proteins (HPs) in cellular migration, proliferation and differentiation, their implication in cancers is attracting increasing interest. Homeobox A2 (HOXA2) was recently found to be overexpressed and an independent prognostic marker in high grade gliomas.1–3 Interestingly, some HPs have the astonishing property to travel between cell.4 More strikingly, these travelling HPs are able to enter target cell nucleus and thereby modify transcriptional activity, potentially spreading oncogenic dysregulation in the healthy neighboring cells.5–7 In this work, we investigated if HOXA2 is a travelling HP.

**Methods:** To investigate HOXA2 intercellular travel, Human embryonic kidney (HEK) 293 cells were transfected with plasmids containing Flag‐HOXA2 and Enhanced Green Fluorescent Protein (EGFP) coding sequences. EGFP was used as positive control for transfection and as negative control of intercellular transfer. ONECUT1, a known travelling HP, was used as positive control for intercellular travel. Anti‐Flag and anti‐ONECUT immunofluorescence labelings were performed 48 hours after cell transfection.

**Results:** Intercellular travel was assessed by immunofluorescence. Since cells were either cotransfected or untransfected, HOXA2‐positive and EGFP‐negative cells were considered homeoprotein recipient cells. Recipient cells were indeed observed in the neighboring of producing EGFP‐ and HOXA2‐positive cells. The ratio between the receiving cells were the Flag‐HOXA2 homeoprotein was detected and the transfected producing cells was 0.87. This ratio is comparable with the 0.81 ratio obtained for the travelling HP ONECUT1, and significantly superior to negative control EGFP (0.09).

**Conclusion:** These results underscore the potential capacities of HOXA2 oncoprotein to travel to, and penetrate surroundings cells in vivo.

**Disclosure:** Nothing to disclose.

## EPR‐284

### Clinical, molecular, and transcriptomic features associated with seizures at onset in patients with glioblastoma

#### J. Rossi ^1^; A. Picca^2^; P. Pugliese^3^; J. Bhalshankar^3^; M. Touat^2^; C. Carpentier^3^; A. Dridi Aloulou^3^; B. Mathon^4^; F. Bielle^5^; B. Donati^6^; E. Froio^7^; S. Serra^7^; A. Ciarrocchi^6^; A. Pisanello^1^; F. Valzania^1^; K. Hoang‐Xuan^2^; M. Sanson^2^

##### ^1^Neurology Unit, Neuromotor & Rehabilitation Department, Azienda USL‐IRCCS of Reggio Emilia, Reggio Emilia, IT; ^2^Service de Neuro‐oncologie, Institut de Neurologie, Hôpital Pitié‐Salpêtrière, AP‐HP, Paris, FR; ^3^Paris Brain Institute (ICM), Sorbonne Université, Inserm, CNRS, UMR S 1127, Paris, FR; ^4^Service de Neurochirurgie, Hôpital Pitié‐Salpêtrière, AP‐HP, Sorbonne Université, Paris, FR; ^5^Service de Neuropathologie, Hôpital Pitié‐Salpêtrière, AP‐HP, Sorbonne Université, Paris, FR; ^6^Laboratory of Translational Research, Azienda USL‐IRCCS of Reggio Emilia, Reggio Emilia, IT; ^7^Pathological Anatomy Service, Oncology Department and Advanced Technologies, Azienda USL‐IRCCS of Reggio Emilia, Reggio Emilia, IT

**Background and aims:** Seizures occur in up to 60% of patients with glioblastoma (GBM), revealing the tumor in 25‐30% of cases. The understanding of the tumor biology associated with epilepsy as the presenting symptom remains limited. Moreover, despite growing preclinical evidence of a link between epileptogenesis and tumorigenesis, the prognostic role of seizures at onset remains debated, partly due to study heterogeneity.

**Methods:** We leveraged a homogeneous cohort of WHO 2021 GBM patients from our Institution with available clinical, molecular, and transcriptomic data. We retrospectively queried clinical records to identify the presence and characteristic of seizures at disease onset.

**Results:** in our cohort of 231 GBM patients, 36% presented with seizures: of them, 47% were focal, 11% focal with impaired awareness, and 42.2% generalized. We did not observe significant differences in terms of age, sex, type of surgery, and tumor localization between the two groups. Patients presenting with seizures had a higher Karnofsky Performance Status score (median KPS: 90 vs. 80, *p* < 0.01) and tended to have a less frequently mutated TERT promoter (86% vs. 94%, *p* = 0.07). Seizures at onset were not associated with overall survival neither in univariate (HR 1.10, C.I. 0.83 ‐ 1.46, *p* = 0.50) nor in multivariate Cox regression analysis (HR 1.16, C.I. 0.86 ‐ 1.57, *p* = 0.33). Differences in terms of gene expression will be assessed on 3' RNA sequencing data.

**Conclusion:** Despite the presence of seizures at diagnosis was associated with higher performance status and a distinct molecular profile, it does not predict survival

**Disclosure:** Nothing to disclose.

## EPR‐285

### Exploratory analyses from Phase 3 INDIGO study show lower seizure rates in patients treated with vorasidenib vs. placebo

#### M. Touat ^1^; I. Mellinghoff^2^; M. van den Bent^3^; D. Blumenthal^4^; K. Peters^5^; J. Clarke^6^; J. Mendez^7^; L. Welsh^8^; W. Mason^9^; A. Hottinger^10^; J. Sepulveda^11^; W. Wick^12^; R. Soffietti^13^; D. Zhao^14^; D. Yi^14^; D. Weidl^15^; L. Steelman^14^; I. Hassan^14^; P. Wen^16^; T. Cloughesy^17^

##### ^1^Pitié Salpêtrière Hospital, Assistance Publique‐Hôpitaux de Paris (AP‐HP) Sorbonne Université, Paris, France; ^2^Memorial Sloan Kettering Cancer Center, New York City, USA; ^3^Erasmus Medical Center, Rotterdam, Netherlands; ^4^Tel Aviv Sourasky Medical Center, Tel Aviv University, Tel Aviv, Israel; ^5^Duke University Medical Center, Durham, USA; ^6^University of California, San Francisco, USA; ^7^Huntsman Cancer Institute at the University of Utah, Salt Lake City, USA; ^8^The Royal Marsden Hospital, London, UK; ^9^Toronto General Hospital, Toronto, Canada, ^10^University Hospital of Lausanne, Lausanne, Switzerland, ^11^Hospital Universitario 12 de Octubre, Madrid, Spain, ^12^Universitatsklinikum Heidelberg, Heidelberg, Germany, ^13^University of Turin, Turin, Italy, ^14^Servier Pharmaceuticals, Boston, USA, ^15^Servier Deutschland GmbH, Munich, Germany, ^16^Dana‐Farber Cancer Institute, Boston, USA, ^17^University of California, Los Angeles, USA

**Background and aims:** Grade 2 isocitrate dehydrogenase 1 or 2 mutant (mIDH1/2) gliomas are slowly progressive, malignant, incurable brain tumors with poor long‐term prognosis. Patients may experience tumor‐related symptoms (e.g., seizures) that impact their daily lives. Vorasidenib, an oral, brain‐penetrant, dual inhibitor of mIDH1/2, has shown significant clinical benefits and a manageable safety profile in the Phase 3 INDIGO study (NCT04164901). Herein, we investigate the potential relationship between vorasidenib and seizure rate, and between tumor volume and seizure activity in patients with mIDH1/2 glioma.

**Methods:** Patients aged ≥12 years with grade 2 mIDH1/2 oligodendroglioma or astrocytoma, with no prior treatment for glioma other than surgery, and no uncontrolled seizures, were randomized 1:1 to receive vorasidenib 40 mg or placebo daily. Exploratory analyses for the number of on‐treatment seizures were conducted in patients with > = 1 seizure during the study using a negative binomial regression model. The potential association between seizure activity and tumor volume was assessed using the mixed effect model with repeated measurements.

**Results:** Patients treated with vorasidenib had lower on‐treatment rates of seizures than those treated with placebo; these differences were more pronounced in patients with oligodendroglioma than with astrocytoma (Table 1). There was a highly positive correlation between tumor volume and seizure number (log tumor size estimate of coefficient: 0.7; standard error: 0.26; *p* = 0.007; Table 2).

**Figure:**
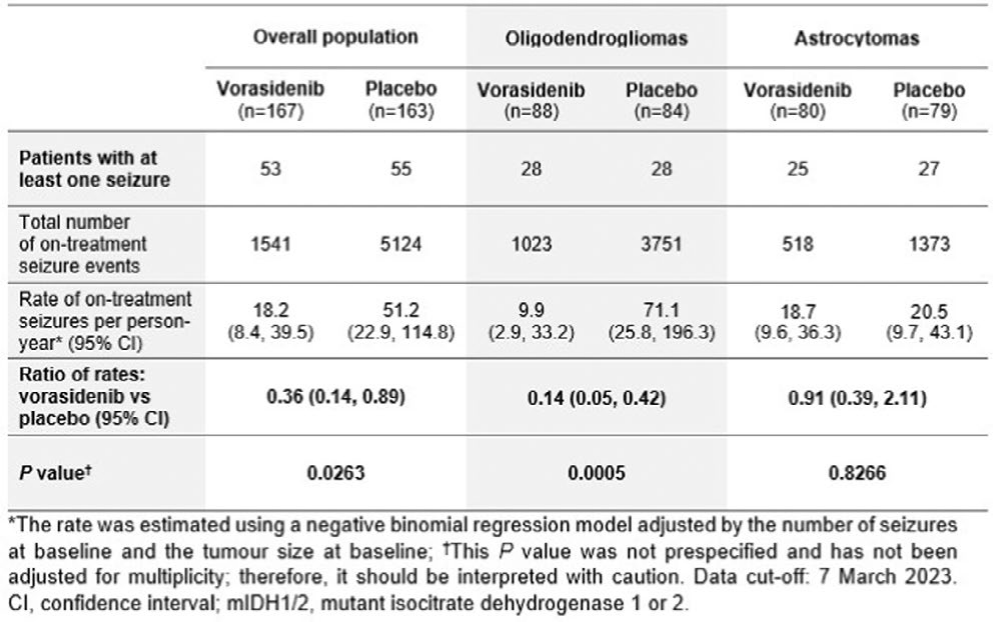
**TABLE 1** Exploratory subgroup analyses of vorasidenib treatment and seizure rate among patients with mIDH1/2 glioma who reported > = 1 on‐treatment seizure.

**Figure:**
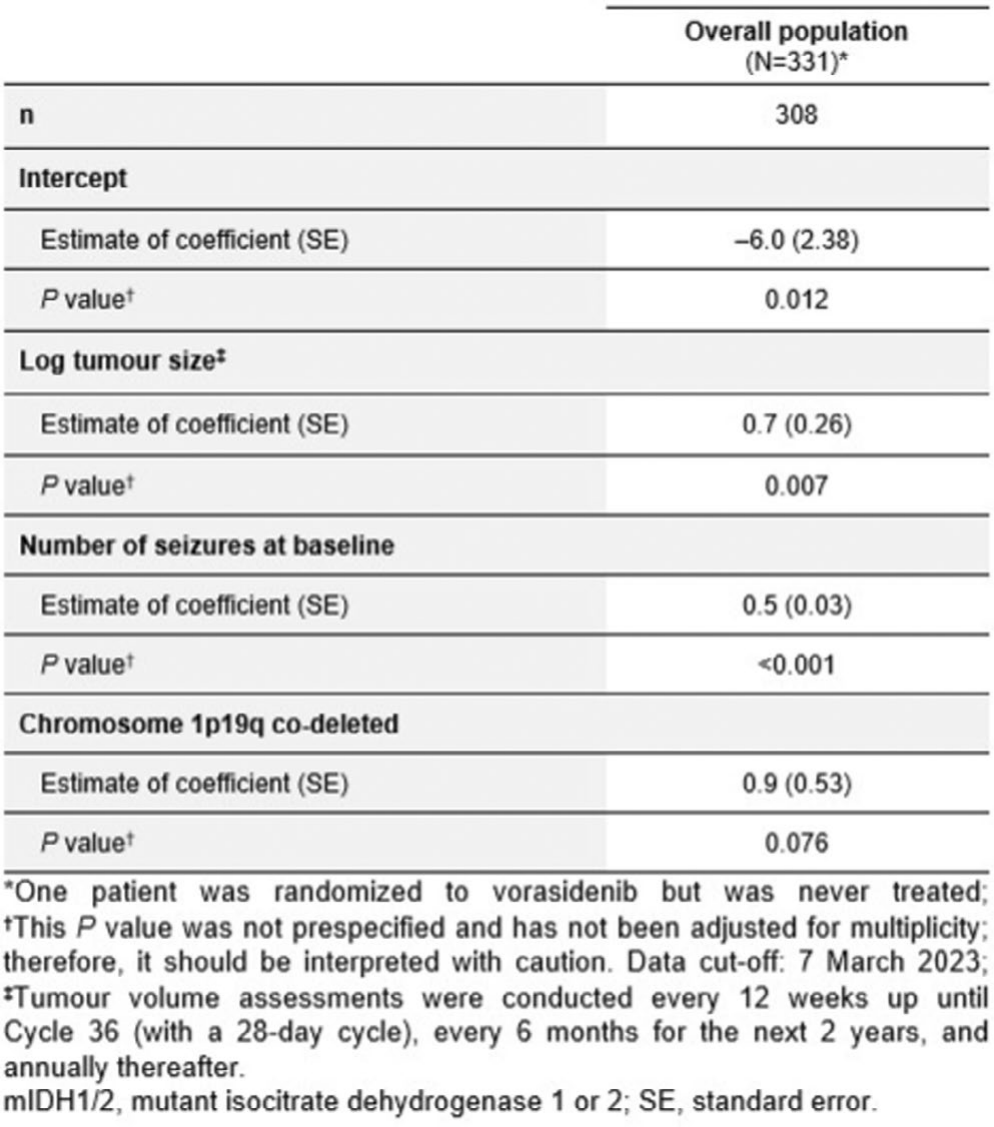
**TABLE 2** Exploratory analysis of the potential association between seizure activity and tumor volume in patients with mIDH1/2 glioma.

**Conclusion:** Treatment with vorasidenib was associated with lower seizure activity than with placebo in patients with mIDH1/2 glioma, particularly in those with oligodendroglioma. Smaller tumor volume was associated with a lower seizure rate.

**Figure:**
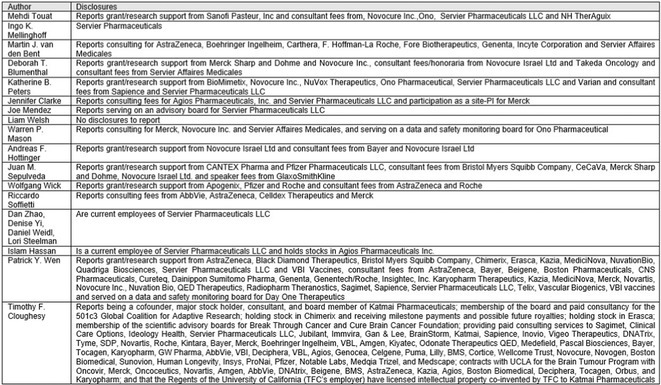
**TABLE 3** Author disclosures.

**Disclosure:** Author disclosures are included in Table 3. This study was sponsored by Servier Pharmaceuticals.

## EPR‐286

Abstract withdrawn

## Neuroimmunology 3

## EPR‐287

### Predictors and phenotypic features of the wearing‐off effect in anti‐CD20 therapy for multiple sclerosis: Insights from patient‐reported outcomes

#### A. Fernandes; C. Correia; B. Barreto; D. Ferro; M. Seabra; T. Mendonça; R. Soares dos Reis; J. Guimarães; P. Abreu

##### Neurology Service, Local Health Unit of São João, Porto, Portugal

**Background and aims:** Wearing‐off phenomenon may be experienced by people with Multiple Sclerosis (pwMS) under anti‐CD20 monoclonal antibodies, which may negatively impact disease control perception and functionality.

**Methods:** A cross‐sectional cohort study of pwMS treated with anti‐CD20 drugs and followed at a tertiary care center. A questionnaire was administered to assess symptoms consistent with the wearing‐off phenomenon.

**Results:** A total of 139 pwMS were included of whom 94 (67.6%) had the relapsing‐remitting form and 45 (32.4%) had progressive forms. 72 patients (51.8%) were under ocrelizumab, 47 (33.8%) under ofatumumab and 20 (14.4%) patients under rituximab. Wearing‐off phenomenon was reported by 43 patients (30.9%), with the majority (72.1%) experiencing at least two concomitant symptoms, most commonly fatigue (*n* = 37) and balance disturbances (*n* = 16). The majority (67.4%) reported symptom onset more than one week before infusion, with complete symptom resolution occurring after the infusion, Most patients (76.7%) classified their symptoms as moderate to severe, with 26 patients (60.5%) reporting a moderate to severe functional impact. In 28 patients (65.1%), wearing‐off was associated with altered perception of disease control. No statistically significant differences were observed in the prevalence of wearing‐off among the different anti‐CD20 therapies. Wearing‐off was more frequent in females (*p* = 0.046). A positive correlation was found between body weight and the occurrence of wearing‐off, but only in males (*p* = 0.041). No statistically significant associations were found with body mass index, drug exposure time, EDSS score, or age.

**Conclusion:** The wearing‐off phenomenon seems to be frequently reported and transversal to different anti‐CD20 therapies.

**Disclosure:** All authors: Nothing to disclose.

## EPR‐288

### Clinical spectrum of anti‐IgLON5 disease: A case series with three distinct movement disorder phenotypes

#### B. Alberti‐Vall; G. Olmedo‐Saura; M. Domine; M. Borrell‐Pichot; M. Caballero‐Avila; A. Vidal‐Jordana; J. Pagonabarraga‐Mora; J. Kulisevsky; J. Perez‐Perez

##### Movement Disorders Unit (Neurology), Hospital de la Santa Creu i Sant Pau, Barcelona, Spain

**Background and aims:** Anti‐IgLON5 antibody‐mediated disease is a neurodegenerative disorder associated with antibodies targeting neuronal cell adhesion proteins. This study describes three clinical cases with distinct movement disorder phenotypes within the spectrum of the disease.

**Methods:** We reviewed three female patients (75, 75, and 77 years old) with confirmed diagnoses of anti‐IgLON5 encephalitis. Data included age, symptom onset duration, clinical phenotypes, diagnostic delay, serological and CSF findings, video‐polysomnography (V‐PSG), and treatment responses. The IgLON5 Composite Score was calculated to assess clinical severity.

**Figure:**
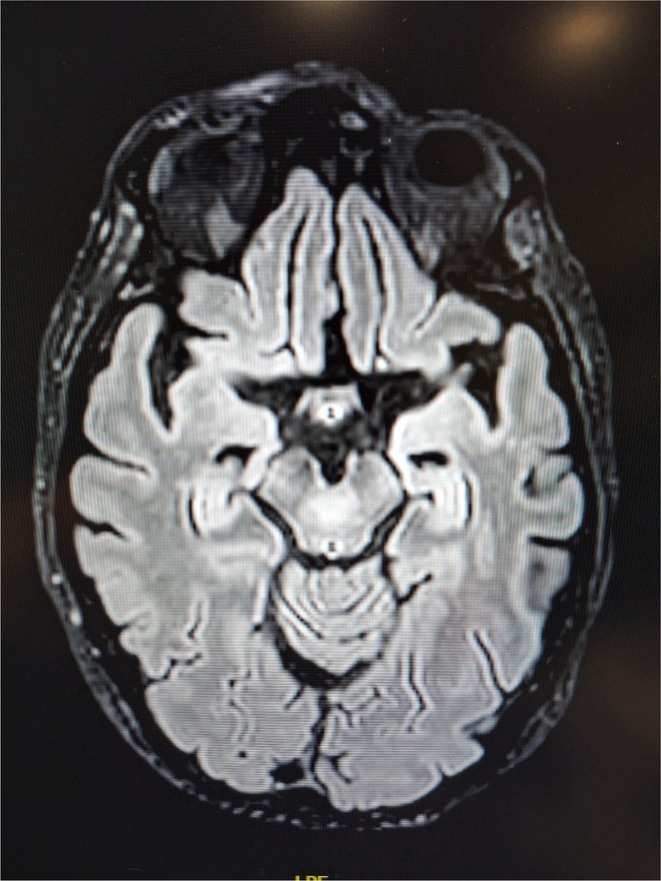
**FIGURE 1** Brain MRI of the first patient showed mutiple white matter hyperintensities in T2 and FLAIR in midbrain, pons, both thalami, compatible with IgLON5 encephalitis.

**Figure:**
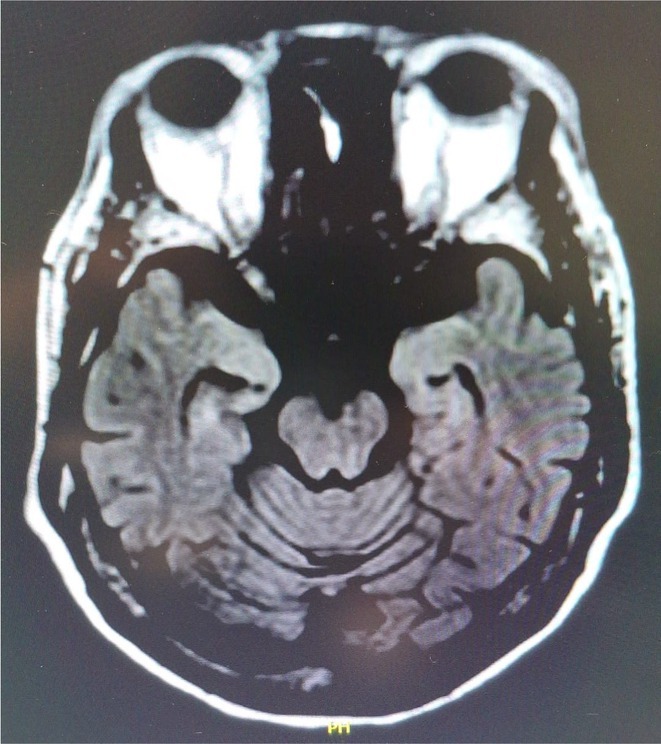
**FIGURE 2** Brain MRI of the second patient showed bilateral hippocampus hyperintensity associated with mild volume loss, compatible with subacute autoimmune encephalitis.

**Results:** The observed phenotypes were: 1. Atypical PSP‐like parkinsonism with midbrain involvement and rigid‐akinetic features (diagnostic delay: 12 months; composite score: 8). Treated with corticosteroids and intravenous immunoglobulins (IgIV) without optimal response, currently receiving rituximab, awaiting the second dose. 2. Chorea with cognitive decline, basal ganglia involvement, and nocturnal behavioral disturbances (diagnostic delay: 6 months; composite score: 9). Treated with IgIV, rituximab, and cyclophosphamide, pending a rituximab booster dose due to clinical worsening. 3. Ataxia and severe bulbar syndrome with bilateral vocal cord paresis and lower brainstem involvement (diagnostic delay: 1 month; composite score: 10). Treated with corticosteroids and five sessions of plasmapheresis, currently on rituximab with good response.

**Conclusion:** Anti‐IgLON5 disease presents heterogeneous phenotypes, complicating early diagnosis. It should be included in the differential diagnosis of movement disorders and specific testing must be conducted to avoid diagnostic delays, as an early therapeutic intervention is essential to improve patient outcomes. This study underscores the importance of a multidisciplinary approach in managing these cases.

**Disclosure:** Nothing to disclose.

## EPR‐289

### Suicidality and depression in pediatric multiple sclerosis

#### B. Nikolić ^1^; N. Ivančević^1^; D. Stevanović^2^; K. Marković^3^; I. Zaletel^4^; D. Nešić^5^; J. Jančić^1^

##### ^1^Clinic of Neurology and Psychiatry for Children and Youth, Belgrade, Serbia, Faculty of Medicine, University of Belgrade, Belgrade, Serbia Faculty of Medicine, University of Belgrade, Belgrade, Faculty of Medicine, University of Belgrade, Belgrade, Serbia; ^2^Clinic of Neurology and Psychiatry for Children and Youth, Belgrade, Serbia; ^3^Paediatric Internal Diseases Clinic, University Clinical Centre Nis, Serbia; ^4^Institute of Histology and Embryology “Aleksandar Đ. Kostić”, Belgrade, Faculty of Medicine, University of Belgrade, Belgrade, Serbia; ^5^Institute of Medical Physiology, Belgrade, Faculty of Medicine, University of Belgrade, Belgrade, Serbia

**Background and aims:** Pediatric multiple sclerosis (PedMS) is immune‐mediated, rare, demyelinating and neurodegenerative diseases of the central nervous system. Studies have shown that depression is being observed in MS patients in approximately 60%, while the prevalence in PedMS patients reaches almost 30% according to studies. So far, only one pilot study has examined rates of suicidal ideation in PedMS. The aim of this study was to examine the presence of depressive symptoms and suicidality in PedMS.

**Methods:** The assessment of suicidal ideation and depressive symptoms was performed using the C‐SSRS and SMFQ, SCARED questionnaires. We used the Extended Neurological Disability Scale (EDSS) to assess neurological disability. Patients with high scores were referred to a child psychiatrist for further evaluation and diagnostic assessment.

**Results:** In total, 27 patients aged up to 18 years with PedMS were analyzed. The mean age of patients was 15.9  ± 1.9 years, with the male to female ratio of 11 (40.7%): 16 (59.3%). All patients had a relapsing‐remitting form of PedMS. The median EDSS is 1.0 (0‐3.0). The average SMFQ was 3.67 (0‐19), while the average SCARED was 11 (0‐54). Depressive symptoms were present in 6 (22.2%) patients, while 1 (3.7%) patient had suicidality. The diagnosis of depression was confirmed in 4 (14.8%).

**Figure:**
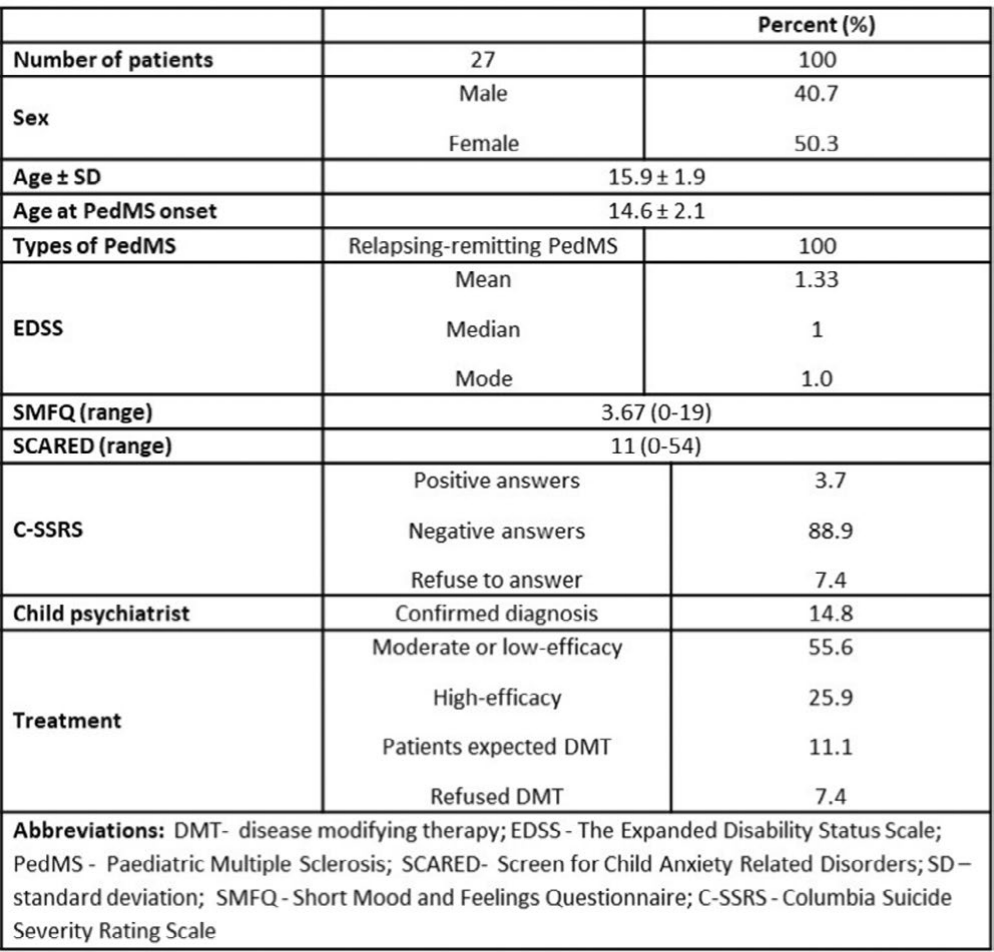
**TABLE 1** Pediatric Multiple Sclerosis – clinical characteristics, depression, suicidality, treatment.

**Conclusion:** In our cohort a significant number of children with PedMS had subclinical depressive symptoms or fully blown depression. Additional research is needed to identify the rates of suicidality in PedMS.

**Disclosure:** Nothing to disclose.

## EPR‐290

### Rapidly progressive cerebellar syndrome caused by anti‐ITPR1 antibodies in a patient with follicular lymphoma

#### E. Huertas Muñoz ^1^; M. Centeno Pons^2^; I. Fojo Suárez^3^; L. Guirao Guillén^4^; M. Serrano Alarcón^5^; M. García Ruiz^6^; P. Mayo Rodríguez^7^; E. López Valdés^8^; A. Fernández Revuelta^9^; A. Maruri Pérez^10^; R. Ginestal López^11^

##### ^1^Neurology Department, Hospital Clinico San Carlos, Madrid, Spain; ^2^Neurology Department, Hospital Clinico San Carlos, Madrid, Spain; ^3^Neurology Department, Hospital Clinico San Carlos, Madrid, Spain; ^4^Neurology Department, Hospital Clinico San Carlos, Madrid, Spain; ^5^Neurology Department, Hospital Clinico San Carlos, Madrid, Spain; ^6^Neurology Department, Hospital Clinico San Carlos, Madrid, Spain; ^7^Neurology Department, Hospital Clinico San Carlos, Madrid, Spain; ^8^Neurology Department, Hospital Clinico San Carlos, Madrid, Spain; ^9^Neurology Department, Hospital Clinico San Carlos, Madrid, Spain, ^10^Neurology Department, Hospital Clinico San Carlos, Madrid, Spain, ^11^Neurology Department, Hospital Clinico San Carlos, Madrid, Spain

**Background and aims:** Rapidly progressive cerebellar syndrome (RPCS) is a high‐risk neurological paraneoplastic phenotype. It is associated with cancers such as small cell lung carcinoma and gynecological cancers. It typically begins with dizziness, nausea, and vomiting, followed by dysarthria, diplopia, nystagmus and ataxia.

**Methods:** We report a 92‐year‐old woman with a history of follicular non‐Hodgkin lymphoma (NHL) who presented with a 3‐week history of dizziness, nausea, and vomiting. On examination, she exhibited mild dysarthria, bilateral gaze‐evoked and downbeat nystagmus, truncal ataxia and dysmetria in all limbs.

**Results:** Blood tests showed mild hyponatremia. Cranial magnetic resonance showed a small acute left occipital infarct and multiple cerebellar hyperintense foci. Cerebrospinal fluid analysis showed mild pleocytosis and positive oligoclonal bands. Immunohistochemistry using indirect immunofluorescence on rodent cerebellar tissue revealed a pattern suggestive of the presence of anti‐inositol 1,4,5‐trisphosphate receptor type 1 (ITPR1) antibodies, confirmed by a cell‐based assay. A whole‐body computed tomography scan showed progression of the lymphoma. Despite treatment with prednisone and intravenous immunoglobulins, the patient passed away.

**Conclusion:** ITPR1, an intracellular antigen in Purkinje cells, is targeted by antibodies in paraneoplastic RPCS. This is the first described case related to NHL.

**Disclosure:** None of the authors has any conflict of interest to disclose.

## EPR‐291

### Sleep disturbances in patients with myasthenia gravis A cross‐sectional study

#### J. Yan; K. Choi; P. Fu; J. Lin; Y. Li; M. Gui; B. Bu; Z. Li

##### Department of Neurology, Tongji Hospital, Tongji Medical College, Huazhong University of Science and Technology, Wuhan 430030, Hubei, China

**Background and aims:** To explore sleep dysfunction among patients with clinically stable patients with Myasthenia Gravis and seek to identify sleep disturbances and uncover their associated risk factors.

**Methods:** A cross‐sectional study was conducted, involving the recruitment of 306 patients with MG from three MG centers. Participants completed an online self‐report questionnaire covering demographics, clinical characteristics, and assessments using the Pittsburgh Sleep Quality Index (PSQI) scale, Stop‐Bang scale, 15‐item quality‐of‐life instrument for myasthenia gravis (MG‐QOL 15) scale, patient health questionnaire (PHQ‐9), and self‐rating anxiety scale (SAS) to evaluate sleep quality among patients with MG.

**Results:** Approximately 68% of patients with MG experienced sleep disturbances (PSQI ≥ 6). Univariate analysis revealed that older age (> 60 years), lower education level (≤12 years), unfavorable marital status, late disease onset (> 55 years old), generalized subtype, myasthenia crisis, positive AchR‐antibody, thymoma, thymectomy, and pathological type B thymoma were risk factors for sleep dysfunction in patients with MG. Within the sleep disturbance group, 51% of patients scored ≥ 3 on the Stop‐Bang scale, indicating a higher risk of obstructive sleep apnea. Global PQSI scores showed significant linear correlations with MG‐QOL 15, Stop‐Bang, PHQ‐9, and SAS scores (*p* < 0.001). Multivariate analysis revealed that sex, marital status, Stop‐Bang score, SAS score, and MG QOL‐15 score exhibited correlations with the PQSI score.

**Figure:**
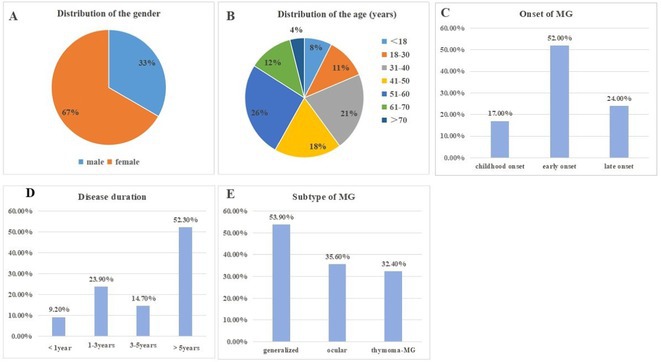
**FIGURE 1** Demographics and Clinical Characteristics of myasthenia gravis patients.

**Figure:**
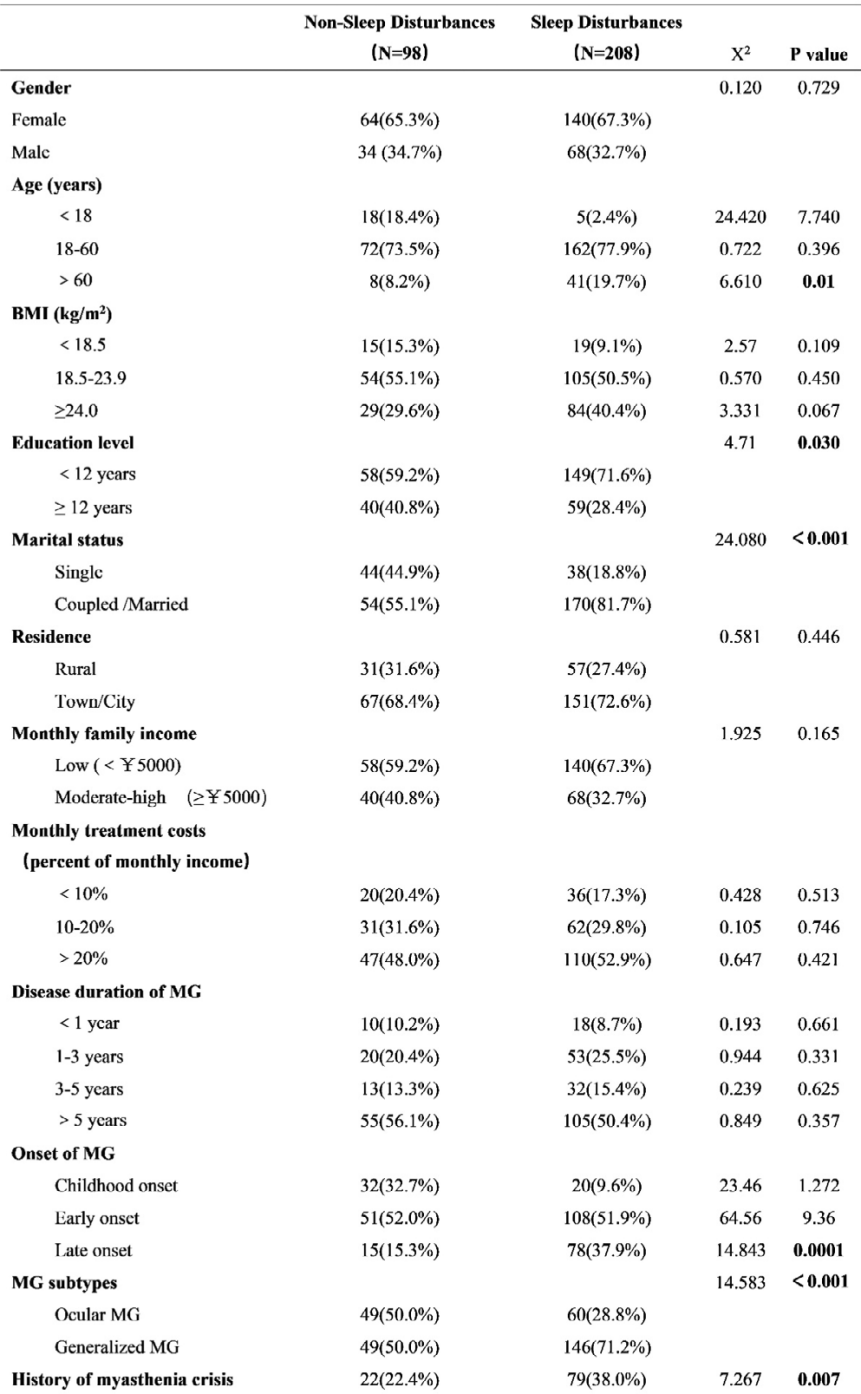
**TABLE 1** Comparison of clinical characteristics of MG patients between the non‐sleep disturbances group and sleep disturbances group (*n*, %).

**Conclusion:** Sleep disturbances are prevalent among patients with MG, even in clinically stable cases. Psychological factors such as anxiety and health‐related quality of life warrant increased attention in the management of these patients.

**Disclosure:** All authors claim that there are no conflicts of interest.

## EPR‐292

### Fc‐glycosylation reveals brain‐specific antibody profiles in NMDARe, tracking post‐herpetic, tumor or idiopathic origin.

#### L. Marmolejo ^1^; C. Papi^2^; C. Milano^3^; E. Maudes^1^; I. Duvniak Oreskovic^4^; J. Stambuk^4^; M. Pucic Jacovic^4^; G. Olivé‐Cirera^5^; T. Armangué^5^; J. Dalmau^1^; M. Spatola^1^

##### ^1^Neuroimmunology Program, Fundació de Recerca Clínic Barcelona‐Institut d'Investigacions Biomédiques August Pi i Sunyer (FRCB‐IDIBAS), University of Barcelona, Spain and Caixa Research Institute (CRI), Barcelona, Spain; ^2^Department of Neuroscience, Catholic University of the Sacred Heart, Rome, Italy; ^3^Department of Clinical and Experimental Medicine, University of Pisa, Pisa, Italy; ^4^Genos Glycoscience Research Laboratory, Zagreb, Croatia; ^5^Pediatric Neuroimmunology Unit, Neurology Service, Sant Joan de Déu (SJD) Children's Hospital, University of Barcelona, Barcelona, Spain

**Background and aims:** Anti‐NMDA receptor encephalitis (NMDARe) is driven by pathogenic IgG1 antibodies, sometimes accompanied by IgG2/3, leading to neuronal dysfunction. These antibodies are always present in CSF and may be present in serum.Triggers include tumors (mainly ovarian teratomas) and herpes simplex virus encephalitis, though many cases remain idiopathic. Prior B‐cell receptor studies suggest compartmentalized antibody responses in the brain, but differences in Fc‐glycosylation between serum and CSF, or by disease trigger, are unknown. This study aimed to identify Fc‐glycosylation profiles in NMDARe that reflect antibody compartmentalization and disease triggers.

**Methods:** Using liquid‐chromatography‐mass‐spectrometry we determined the Fc‐glycosylation profiles of IgG1 and IgG2/3 in paired serum and CSF samples from (age‐ and sex‐matched) patients with NMDARe (*n* = 50). Patients were classified based on disease trigger into post herpetic, tumor‐related or idiopathic. Glycoprofiles were determined by quantification of fucosylation, sialylation, galactosylation and bisecting N‐glucosamination.

**Results:** CSF IgG1 and IgG2/3 displayed a more inflammatory Fc‐glycan profile than serum, with lower sialylation and galactosylation (*p* < 0.001). Post‐herpetic NMDARe showed the most inflammatory glycoprofile, with reduced sialylation, lower galactose levels, and increased bisecting N‐glucosamination (*p* < 0.001), differences absent in serum. Fucosylation levels remained stable across compartments and triggers.

**Conclusion:** In patients with NMDARe, CSF shows distinct Fc‐glycosilation profiles, supporting a compartmentalized antibody response within the brain. Post‐herpetic NMDARe is associated with more inflammatory glycoprofiles than other triggers. As glycan signatures determine different interactions with the innate immunity (e.g., complement, NK cells), these findings suggest distinct pathogenic mechanisms that might be harnessed to develop compartment‐specific and trigger‐specific therapeutic strategies in NMDARe

**Disclosure:** Nothing to disclose.

## EPR‐293

### Types and prognosis in peripheral nervous system involvement related to immune checkpoint inhibitors

#### M. İriş ^1^; Z. Birsin^2^; Z. Turna^2^; N. Uzun Adatepe^1^; M. Özgüroğlu^2^; A. Gündüz^1^

##### ^1^Neurology, Istanbul University‐Cerrahpasa, Cerrahpasa Medical Faculty, Istanbul, Turkey; ^2^Oncology, Istanbul University‐Cerrahpasa, Cerrahpasa Medical Faculty, Istanbul, Turkey

**Background and aims:** This study aimed to identify the frequency and types of peripheral nervous system involvement, clinical features, treatment options, and prognosis in patients receiving immune checkpoint inhibitors (ICIs) due to malignancy.

**Methods:** This study is a retrospective cross‐sectional study. We included data of patients who presented to our electromyography laboratory with neurological complaints and were using ICIs between January 2019 and August 2024. We retrieved medical and electrophysiological records. Patients with complaints before the use of ICIs were excluded. In cases with multiple examinations, the first appropriate examination was included. Demographic data, oncological and neurological diagnosis, and medications used were detected from patient files.

**Results:** During the study, we reviewed the records of 31 patients. Seven were excluded. Two of the remaining 24(female = 11, male = 13; age:34‐78) had multiple examinations. The most commonly used ICIs were pembrolizumab(*n* = 7) and nivolumab(*n* = 6). The most frequent malignancies were non‐small cell lung cancer(*n* = 13) and breast cancer(*n* = 3). Electrophysiologically, sensory or sensory‐motor axonal polyneuropathy was detected in 13(5 accompanied by myopathy), demyelinating polyneuropathy in 1, isolated myopathy in 4, and anterior root/anterior horn involvement in 1. The drugs most commonly used were nivolumab, pembrolizumab, atezolizumab. The treatment option was immune modulation in cases with demyelinating neuropathy and myopathy. The prognosis was poor in myopathies due to cardiovascular complications and complications related to malignancy.

**Conclusion:** ICIs have been increasingly used in cancer treatment and can cause immune‐related effects. As demonstrated here, the most common peripheral nervous system involvement is axonal polyneuropathy, followed by myopathy.

**Disclosure:** Nothing to disclose.

## EPR‐294

### Immune checkpoint dysregulation in multiple sclerosis: Insights into AhR‐driven regulatory B cell function

#### T. Tsaktanis; D. Farrenkopf; V. Rothhammer

##### Department of Neurology, University Hospital Erlangen, Friedrich‐Alexander University Erlangen‐Nürnberg, Erlangen, Germany

**Background and aims:** This study aimed to characterize regulatory B cell populations in MS patients, assessing disease‐related changes in these subsets. Given recent evidence linking regulatory B cells to immune checkpoint molecules regulated by the aryl hydrocarbon receptor (AhR), we investigated the correlation between regulatory B cell populations, AhR serum levels, and the expression of co‐inhibitory molecules.

**Methods:** Peripheral blood mononuclear cells (PBMCs) were analyzed using high‐dimensional flow cytometry from the serum of patients with relapsing‐remitting MS (RRMS, *n* = 68), secondary progressive MS (SPMS, *n* = 17), and non‐inflammatory neurological diseases (NINDS, *n* = 76). Regulatory B cells were identified through subset‐specific markers and examined for immune checkpoint molecule expression. AhR serum levels were quantified using a Dual‐Luciferase Reporter Assay.

**Results:** Regulatory B cell subsets (CD24^+^CD38ʰⁱ, CD5^+^CD1d^+^, and CD39^+^CD73^+^) were significantly reduced in MS patients compared to individuals with NINDS, alongside altered checkpoint molecule expression. Conversely, CD27^‐^IgD^‐^ “double‐negative” (DN) B cells were increased. Moreover, a positive correlation was observed between AhR serum levels and Tim‐1 expression on B cells.

**Conclusion:** This study elucidates MS‐specific alterations in B cell subsets, demonstrating impaired regulatory properties mediated by immune checkpoint dysfunction. The observed reduction in co‐inhibitory molecule expression suggests that AhR signaling, known for its immunomodulatory effects, may restore regulatory B cell function. These findings position AhR as a potential therapeutic target for selectively modulating B cell populations, offering a more precise alternative to broad‐spectrum B cell depletion in MS. The study's insights could have meaningful clinical implications, enhancing diagnostic precision and guiding therapeutic strategies for MS management.

**Disclosure:** Nothing to disclose.

## Muscle and neuromuscular junction disorder 3

## EPR‐295

### Improvement in myasthenia gravis‐specific outcome subdomain scores with zilucoplan: RAISE‐XT 120‐week post hoc analysis

#### A. Maniaol ^1^; S. Bresch^2^; C. Hewamadduma^3^; M. Leite^4^; M. Smilowski^5^; K. Utsugisawa^6^; M. Weiss^7^; B. Boroojerdi^8^; F. Grimson^9^; N. Savic^10^; J. Howard^11^

##### ^1^Department of Neurology, Oslo University Hospital, Oslo, Norway; ^2^Service de Neurologie, Hospital Pasteur, Centre Hospitalier Universitaire de Nice, Nice, France; ^3^Academic Neuroscience Unit, Sheffield Teaching Hospitals NHS Foundation Trust & Sheffield Institute for Translational Neuroscience (SITraN), University of Sheffield, Sheffield, UK; ^4^Nuffield Department of Clinical Neurosciences, University of Oxford, Oxford, UK; ^5^Department of Hematology and Bone Marrow Transplantation, Medical University of Silesia, Katowice, Poland; ^6^Department of Neurology, Hanamaki General Hospital, Hanamaki, Japan; ^7^Department of Neurology, University of Washington Medical Center, Seattle, USA; ^8^UCB, Monheim, Germany; ^9^UCB, Slough, UK, ^10^UCB, Bulle, Switzerland, ^11^Department of Neurology, The University of North Carolina at Chapel Hill, Chapel Hill, USA

**Background and aims:** In the Phase 3 RAISE study (NCT04115293), zilucoplan, a complement component 5 inhibitor, demonstrated clinically meaningful improvements in Myasthenia Gravis Activities of Daily Living (MG‐ADL) and Quantitative Myasthenia Gravis (QMG) total scores versus placebo in patients with anti‐acetylcholine receptor antibody‐positive generalized myasthenia gravis. Improvements were sustained during long‐term use in the ongoing open‐label extension, RAISE‐XT (NCT04225871). Treatment may have differential effects across different muscle groups; this post hoc analysis evaluated the effect of zilucoplan on MG‐ADL and QMG subdomain scores.

**Methods:** Patients who completed a qualifying double‐blind study (NCT03315130/RAISE) could enter RAISE‐XT to self‐administer once‐daily subcutaneous injections of zilucoplan 0.3mg/kg. Mean changes from double‐blind baseline to Week 120 in MG‐ADL and QMG subdomain scores (ocular, bulbar, respiratory and limb/gross motor) in patients with baseline scores > = 1 in that subdomain were assessed post hoc (interim data cut‐off: 11 November 2023).

**Results:** Overall, 200 patients entered RAISE‐XT; 183 received placebo or zilucoplan 0.3mg/kg in the double‐blind studies. At Week 120, mean (standard error [SE]) change from baseline (CFB) was −7.14 (0.44; *n* = 86) for MG‐ADL and −9.84 (0.65; *n* = 83) for QMG total scores. Mean (SE) CFB in MG‐ADL subdomain scores were: ocular, −1.37 (0.27; *n* = 41); bulbar, −1.32 (0.32; *n* = 22); respiratory, −0.07 (0.11; *n* = 30); and limb/gross motor, −0.87 (0.17; *n* = 39). Mean (SE) CFB in QMG subdomain scores were: ocular, −2.52 (0.30; *n* = 52); bulbar, −1.00 (0.45; *n* = 16); respiratory, −0.06 (0.18; *n* = 33); and limb/gross motor, −4.27 (0.43; *n* = 79).

**Conclusion:** Treatment with zilucoplan for up to 120 weeks demonstrated sustained improvements in all MG‐ADL and QMG subdomain scores.

**Disclosure:** This study was funded by UCB. Babak Boroojerdi, Fiona Grimson and Natasa Savic are employees and shareholders of UCB. Full disclosure of all industry relationships will be made during congress presentation if accepted.

## EPR‐296

### Centers of excellence: Establishing a harmonized, holistic approach for managing Pompe disease with gene therapy

#### B. Schoser ^1^; C. Domínguez‐González^2^; P. Laforet^3^; A. Hahn^4^; P. Gissen^5^; A. Kostera‐Pruszczyk^6^; A. Thalmeier^7^; J. Vissing^8^

##### ^1^Department of Neurology, Friedrich‐Baur‐Institute, Ludwig‐Maximilians‐University, Munich, Germany; ^2^Hospital Universitario 12 de Octubre, imas12 Research Institute, Madrid, Spain; ^3^Raymond‐Poincaré Hospital, Paris, France; ^4^Justus‐Liebig University, Giessen, Germany; ^5^University College London, London, UK; ^6^Department of Neurology, Medical University of Warsaw, Warsaw, Poland; ^7^LMU‐Klinikum, Munich, Germany; ^8^University of Copenhagen, Copenhagen, Denmark

**Background and aims:** Emerging gene therapies have substantial logistical and clinical challenges requiring specialized centers with defined protocols. A scientific steering committee of 8 European experts deliberated the requirements for establishing centers of excellence (CoEs) for Pompe disease capable of incorporating gene therapy into patient management.

**Methods:** A modified think‐tank approach was used to develop a consensus based on nominal group techniques, whereby discussions were led by a chairperson and informed by qualitative research (Figure 1).

**Figure:**
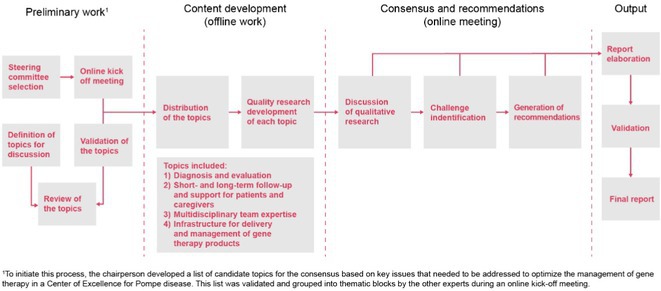
**FIGURE 1** Methodological process used to develop the consensus.

**Results:** Improving the diagnosis and evaluation of Pompe disease is crucial for optimizing patient management and ensuring timely access to treatment. The committee recommended expanding newborn screening programs for infantile‐onset Pompe disease and developing and implementing protocols for presymptomatic late‐onset Pompe disease follow‐up. A specialized multidisciplinary team trained in Pompe disease and gene therapy management is necessary to manage all stages of the patient's journey effectively (Figure 2). Pre‐gene therapy assessments were recommended to mitigate risks. During gene therapy infusions, patients are recommended to undergo continuous vital sign monitoring and be hospitalized for up to 1 week. Post‐gene therapy guidelines encompass corticosteroid immunosuppression, monitoring of adverse events (including hepatoxicity, troponin‐I levels, thrombocytopenia, and thrombotic microangiopathy), and monitoring of Pompe disease (eg, functional assessments, muscle and cardiac magnetic resonance imaging every 6–12 months, and patient‐reported outcomes). CoEs may need infrastructure upgrades to meet standard operating procedures for gene therapy products.

**Figure:**
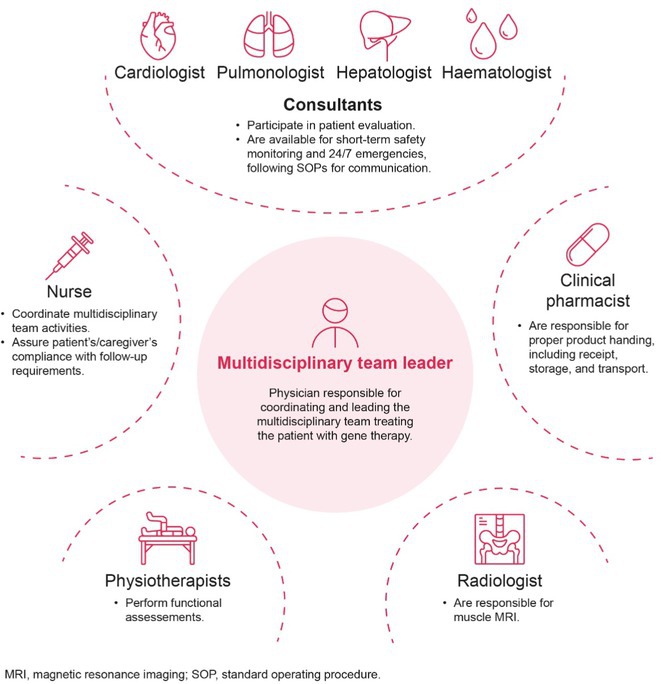
**FIGURE 2** Multidisciplinary team profiles recommended by the experts for a center of excellence specializing in gene therapy for Pompe disease.

**Conclusion:** Successful implementation of gene therapy for Pompe disease requires a coordinated multidisciplinary effort to overcome existing gaps in knowledge, infrastructure, and care delivery.

**Disclosure:** Medical writing support was provided by Bassaam Mulk, PharmD, of Alpha (a division of Prime, Knutsford, UK), funded by Astellas. The authors disclose: BS: research grants from AMDA Foundation, Amicus, EU Horizon programs ComPaSS and PaLaDIn, Marigold Foundation, Roche Diagnostics; honoraria from Alexion, Amicus, Argenx, Astellas, Kedrion, Sanofi; and scientific advisorship for Amicus, Alexion, Astellas, Sanofi, Taysha. CDG: research grants from UCB, Pretzel Therapeutics; honoraria from Amicus, Sanofi, Astellas, Roche, UCB; and consulting fees from Amicus, Sanofi, Pretzel, UCB. PL: research grants from Amicus, Sanofi; advisory boards for Amicus, Astellas, Sanofi; honoraria from Amicus; and meeting attendance/travel support from Amicus, Sanofi. AH: Nothing to disclose. PG: consulting fees from BioMarin, Travere Therapeutics; honoraria from BioMarin; and share ownership in Bloomsbury Genetic Therapies; none in relation to the submitted work. AK‐P: research grants from Biogen, Kedrion, Sanofi; honoraria from Alexion, AstraZeneca, Biogen, CSL Behring, Kedrion, Novartis, Roche, Takeda, UCB; meeting attendance/travel support from Alexion, AstraZeneca, Biogen, CSL Behring, Novartis, Roche, Takeda, UCB; and scientific advisorship for Merck. AT: consulting fees from Astellas, Roche. JV: research grant from Novo Nordisk Foundation; consulting fees/advisory boards from Dyne Therapeutics, Roche, Sanofi; and honoraria from Alexion, Edgewise Therapeutics, Janssen, UCB.

## EPR‐297

### Concomitant immunosuppressive therapy use with Ravulizumab: Analysis of a generalized myasthenia gravis global registry

#### R. Nowak^1^; A. Habib^2^; A. Meisel^3^; C. Scheiner ^4^; L. Zeinali^5^; C. Liu^6^; M. Pulley^7^; G. Cutter^8^; A. Gordon^9^; P. Narayanaswami^10^

##### ^1^Yale School of Medicine, New Haven, USA; ^2^University of California, Irvine, USA; ^3^Charité Universitätsmedizin Berlin, Berlin, Germany; ^4^University of Tennessee Medical Center, Knoxville, USA; ^5^Alexion, AstraZeneca Rare Disease, Mississauga, Canada; ^6^Alexion, AstraZeneca Rare Disease, Baar, Switzerland; ^7^University of Florida, Jacksonville, USA; ^8^University of Alabama at Birmingham, Birmingham, USA; ^9^Northwest Neurology, Ltd., Lake Barrington, USA, ^10^Beth Israel Deaconess Medical Center/Harvard Medical School, Boston, USA

**Background and aims:** High‐dose and long‐term use of oral corticosteroids (OCS), including concomitant immunosuppressive therapies (con‐ISTs), may be associated with short‐ and long‐term adverse events and other health risks. The global MG SPOTLIGHT Registry (NCT04202341) assesses clinical practice outcomes with ravulizumab and eculizumab in adults with anti‐acetylcholine receptor antibody‐positive (AChR‐Ab+) generalized myasthenia gravis (gMG). Here we describe changes in con‐IST use after ravulizumab initiation.

**Methods:** This analysis included patients treated with ravulizumab for ≥6mo with available con‐IST (azathioprine, mycophenolate mofetil, intravenous immunoglobulin/plasma exchange, methotrexate, and OCS) data. Descriptive analyses characterized treatment changes after ravulizumab initiation. No adjustment for covariates was performed. Safety was assessed in all patients.

**Results:** As of 01Jul2024, data from 44 patients fulfilled inclusion criteria for analysis (male: 70.5%; mean ± SD age at enrolment: 68.3 ± 13.0yrs; mean ± SD ravulizumab treatment duration: 1.3 ± 0.8yrs). At ravulizumab initiation, 19/44 (43.2%), 13/44 (29.5%), and 1/44 (2.3%) patients were receiving 1, 2, and ≥3 con‐ISTs, respectively. Thereafter, 10/33 (30.3%) patients discontinued ≥1 con‐IST, with 3/10 (30%) and 8/10 (80%) patients discontinuing within 3mo and 6mo of ravulizumab initiation, respectively. Following ravulizumab treatment, the number of patients receiving ≤5 and ≤10 mg/day OCS increased from 11/26 (42.3%) and 16/26 (61.5%), respectively, to 14/26 (53.8%) and 20/26 (76.9%) 3mo after ravulizumab initiation and 16/26 (61.5%) and 20/26 (76.9%) 6mo after ravulizumab initiation. Ravulizumab was well tolerated, consistent with previous analyses and clinical trial data.

**Conclusion:** Reduced con‐IST and OCS burden was observed in patients with AChR‐Ab+ gMG treated with ravulizumab in clinical practice, supporting a steroid‐sparing role for ravulizumab.

**Disclosure:** RJN: research suppt/consultant/advisor: Alexion, Annexon, argenx, Cabaletta, Cour, Genentech, Grifols, Immunovant, MGFA, Momenta, NIH, Ra, S.A., Viela. AAH: research suppt: Alexion, AstraZeneca Rare Disease, argenx, Cabaletta, Genentech, Immunovant, Pfizer, Regeneron, UCB, Viela. AM: research suppt/speaker/consultant: Alexion, AstraZeneca Rare Disease, argenx, Octapharma, Grifols, Hormosan, Janssen, UCB; ad board chairman: German Myasthenia Gravis Society. CAS: ad board/speaker: Alexion, AstraZeneca Rare Disease, argenx, CSL Behring. LZ,CL,AY:employees/stock (options):Alexion, AstraZeneca Rare Disease. MTP: ad board: Alexion, AstraZeneca Rare Disease, Amgen, argenx, Catalyst, CSL Behring, Immunovant, UCB. GC:consultant/data&safety monitoring/ad boards:AI Therapeutics, Alexion, AstraZeneca Rare Disease, AMO, Antisense Therapeutics, Applied Therapeutics, AveXis, Avotres, Biogen, BMS, Clene Nanomedicine, Clinical Trial Solutions, CSL Behring, Entelexo, Genentech, Genzyme, GW Pharma, Hoya, Horizon Pharma, Immunic, Immunosis, Karuna, Kezar Life Sciences, Klein Buendel, Linical, Mapi, Merck/Serono, Mitsubishi Tanabe, NHLBI, Novartis, Opko Biologics, Perception Neuroscience, Protalix, Prothena, Reata, Regeneron, Roche, S.A., SAB Bio, Teva, UT Southwestern, UPenn, Visioneering Technologies. AJG: honoraria: Alexion, AstraZeneca Rare Disease, argenx, Janssen, UCB. PN:research/ad boards/data monitoring chair/speaker:Alexion, AstraZeneca Rare Disease, argenx, Momenta/Janssen, PCORI, Ra, Sanofi, UCB.

## EPR‐298

### Overcoming challenges in digital biomarker collection for myasthenia gravis: Insights from the ME&MGopen study

#### C. Barnett‐Tapia^1^; S. Lehnerer^2^; S. Bieuvelet^3^; L. Carment ^3^; C. Gorin^3^; N. Pesic‐Heuvrard^3^; D. Ravindra^3^; N. Sellami^3^; B. Dutta^4^; A. Yegin^4^; S. Zinaï^3^; J. Howard; Jr^5^

##### ^1^Ellen and Martin Prosserman Centre for Neuromuscular Diseases, Division of Neurology, Department of Medicine, University Health Network, University of Toronto, Toronto, Canada; ^2^Charité – Universitätsmedizin Berlin, Corporate Member of Freie Universität Berlin and Humboldt‐Universität zu Berlin, Department of Neurology with Experimental Neurology, Berlin, Germany; ^3^Ad Scientiam, Paris, France; ^4^Alexion Pharmaceuticals Inc.; ^5^The University of North Carolina, Department of Neurology, Chapel Hill, USA

**Background and aims:** Generalized myasthenia gravis (gMG) is a rare neuromuscular disorder causing fluctuating muscle weakness. Digital biomarkers (dBMKs) enable remote symptom tracking with potential benefits for gMG management, but challenges related to data quality and adherence remain. The ME&MGopen study evaluates the feasibility of using a smartphone app (ME&MGopenTM) to collect dBMKs that measure gMG symptoms under real‐world conditions.

**Methods:** The decentralized ME&MGopen study enrolled 236 anti‐acetylcholine receptor antibody‐positive patients, with 125 included in analyses after one‐year follow‐up. ME&MGopenTM provided monthly tests to assess dBMKs for ptosis, limb fatigability, dysarthria, and respiratory impairment. DBMKs were compared with human‐assigned annotations to evaluate precision and identify improvements. Adherence, satisfaction and perceived usefulness were assessed.

**Results:** The patients (70% female) spanned diverse gMG severity (MGFA Class II: 21%, III: 61%, IV: 18%), with mean age of 59 ± 16 years, and disease duration from symptom onset of 12 ± 13 years. Across > 1500 data points in each test, most data met quality criteria (% of tests meeting criteria: ptosis = 85%, dysarthria = 94%, respiratory = 94%, upper limb = 74%, lower limb = 82%). Correlation coefficients between dBMKs and manual annotations exceeded 0.7, indicating high accuracy. Factors affecting app performance were identified: arm compensation (upper limb test), face positioning (ptosis test). After one year, > 65% of patients remained adherent, most showing interest in ME&MGopenTM.

**Conclusion:** These data highlight the feasibility of dBMKs collection in gMG, addressing real‐world challenges in a broad population and revealing opportunities for app improvements to support its ongoing validation study.

**Disclosure:** C.Barnett‐Tapia: Research funding (paid to her institution) (Ad Scientiam, Alexion, Cartesian Therapeutics, US Department of Defence, Muscular Dystrophy Canada, MGNet, Grifols and Octapharma); Honoraria (consulting and/or Advisory board) fees (AcademicCME, Alexion AZ Rare Disease, argenx, Sanofi, Novartis, UCB Pharma and Janssen). S.Lehnerer has received speaker or consultancy honoraria or financial research support (paid to his institution) from Alexion, argenx, Biogen, Hormosan, HUMA, Johnson & Johnson, Merck, UCB and Roche. S.Bieuvelet, L.Carment, C.Gorin, N.Pesic‐Heuvrard, D.Ravindra, N.Sellami, S.Zinaï: employees of Ad Scientiam. B.Dutta, A.Yegin: employees of Alexion Pharmaceuticals Inc. J.F.Howard: Research funding (paid to his institution) (Alexion AstraZeneca Rare Disease, argenx, Cartesian Therapeutics, Centers for Disease Control and Prevention, MGFA, Muscular Dystrophy Association, NIH, NMD Pharma, PCORI, UCB Pharma); honoraria/consulting fees (AcademicCME, Alexion AstraZeneca Rare Disease, Amgen, argenx, Biohaven Ltd, Biologix Pharma, CheckRare CME, F. Hoffmann‐LaRoche Ltd, Horizon Therapeutics plc CoreEvitas, Curie.bio, Medscape CME, Merck EMD Serono, NMD Pharma, Novartis Pharma, PeerView CME, Physicians' Education Resource (PER) CME, PlatformQ CME, Regeneron Pharmaceuticals, Sanofi US, UCB Pharma, Zai Labs); non‐financial support (Alexion AstraZeneca Rare Disease, argenx, Biohaven Ltd. Cartesian Therapeutics, Toleranzia AB, UCB Pharma and Zai Labs).

## EPR‐299

### The role of antisense oligonucleotides in Duchenne muscular dystrophy: A comprehensive meta‐analysis

#### F. Abdelrahman ^3^; M. Mustafa^2^; A. Shariff^4^; M. Hegazy^3^; Z. Sayed^3^; N. Bekhit^3^; M. Elsayed^1^

##### ^1^MME Foundation; ^2^Modern University for Technology and Information; ^3^Newgiza University, Giza, Egypt; ^4^Badr University in Cairo, Egypt

**Background and aims:** Duchenne muscular dystrophy (DMD) is a severe X‐linked disorder caused by mutations in the DMD gene, leading to dystrophin deficiency and progressive muscle degeneration. Antisense oligonucleotides (AONs) are a promising therapeutic strategy that induces exon skipping to restore the DMD reading frame, enabling production of functional dystrophin. Despite encouraging results, variability in outcomes highlights the need for comprehensive evaluation.

**Methods:** A systematic search of PubMed, Embase, Cochrane Library, and ClinicalTrials.gov identified preclinical and clinical studies published up to 2024. Eligible studies assessed AON therapies targeting exons 44, 45, or 51. Data were synthesized following PRISMA guidelines, and study quality was evaluated using risk of bias tools.

**Results:** Thirty‐five studies (20 preclinical and 15 clinical trials) were included. Preclinical models showed dystrophin restoration levels of 30%–45% of normal, with significant improvements in muscle strength (mean increase: 40%, 95% CI: 32%–48%, *p* < 0.001). Clinical trials demonstrated dystrophin restoration levels of 5%–15%, accompanied by functional gains in the 6‐minute walk test (mean increase: 25 meters, 95% CI: 15–35 meters, *p* < 0.01). Safety profiles were favorable, with common adverse events including mild injection site reactions and transient creatine kinase elevations. Subgroup analyses indicated improved outcomes with early treatment and optimized dosing.

**Conclusion:** AON therapies offer significant potential for treating DMD, achieving meaningful dystrophin restoration and functional improvements with manageable safety concerns. Further refinement of AON chemistries and delivery systems is essential to maximize therapeutic impact. These findings reinforce the role of AONs as a key pillar in the evolving landscape of DMD therapy.

**Disclosure:** Nothing to disclose.

## EPR‐300

### MG‐ADL subdomain score changes with Eculizumab or Ravulizumab: A generalized myasthenia gravis global registry analysis

#### V. Juel^1^; S. Macwan^2^; F. Saccà^3^; A. Gordon^4^; L. Zeinali^5^; J. Winkley^6^; C. Liu^7^; G. Cutter^8^; D. Dodig^9^; J. Alpers^10^; R. Tandan ^11^

##### ^1^Duke University, Durham, USA; ^2^Eisenhower Health Center, Rancho Mirage, USA; ^3^NSRO Department, University of Naples Federico II, Napoli, Italy; ^4^Northwest Neurology, Ltd., Lake Barrington, USA; ^5^Alexion, AstraZeneca Rare Disease, Mississauga, Canada; ^6^Baptist Health Medical Group Neurology, Lexington, USA; ^7^Alexion, AstraZeneca Rare Disease, Baar, Switzerland; ^8^University of Alabama at Birmingham, Birmingham, USA; ^9^University of Toronto/Toronto Western Hospital, Toronto, Canada, ^10^Erlanger Medical Center, Chattanooga, USA, ^11^University of Vermont Medical Center, Burlington, USA

**Background and aims:** The global MG SPOTLIGHT Registry (NCT04202341) collects data on the real‐world clinical safety/effectiveness of eculizumab and ravulizumab, complement component 5 (C5) inhibitor therapies (C5ITs), in adults with generalized myasthenia gravis (gMG). Here, we assess changes in MG Activities of Daily Living (MG‐ADL) subdomain scores after C5IT initiation.

**Methods:** MG‐ADL subdomain scores were assessed in Registry patients who received eculizumab (eculizumab subgroup), ravulizumab (ravulizumab subgroup), or transitioned from eculizumab to ravulizumab (switch subgroup) with data available prior to C5IT initiation and during treatment.

**Results:** This analysis (data cutoff: 01Jul2024) includes 178 Registry patients (male: 55.7%; mean ± SD age at MG diagnosis: 54.7 ± 19.0yrs) with 89, 49, and 40 patients in the eculizumab, ravulizumab, and switch subgroups, respectively. Statistically significant reductions (*p* < 0.05) in mean scores were observed for all MG‐ADL subdomains after C5IT initiation (Figure 1). The proportions of patients with complete or partial improvement in individual MG‐ADL subdomains during C5IT treatment were similar between the eculizumab and ravulizumab subgroups: 60.7% and 63.3% (ocular), 58.4% and 59.2% (bulbar), 51.7% and 49.0% (limbs), and 33.7% and 26.5% (respiratory), respectively. Among the switch subgroup, the proportions of patients with complete or partial improvement in individual subdomains were 67.5% (ocular), 67.5% (bulbar), 65.0% (limbs), and 45.0% (respiratory) at last assessment during ravulizumab treatment.

**Figure:**
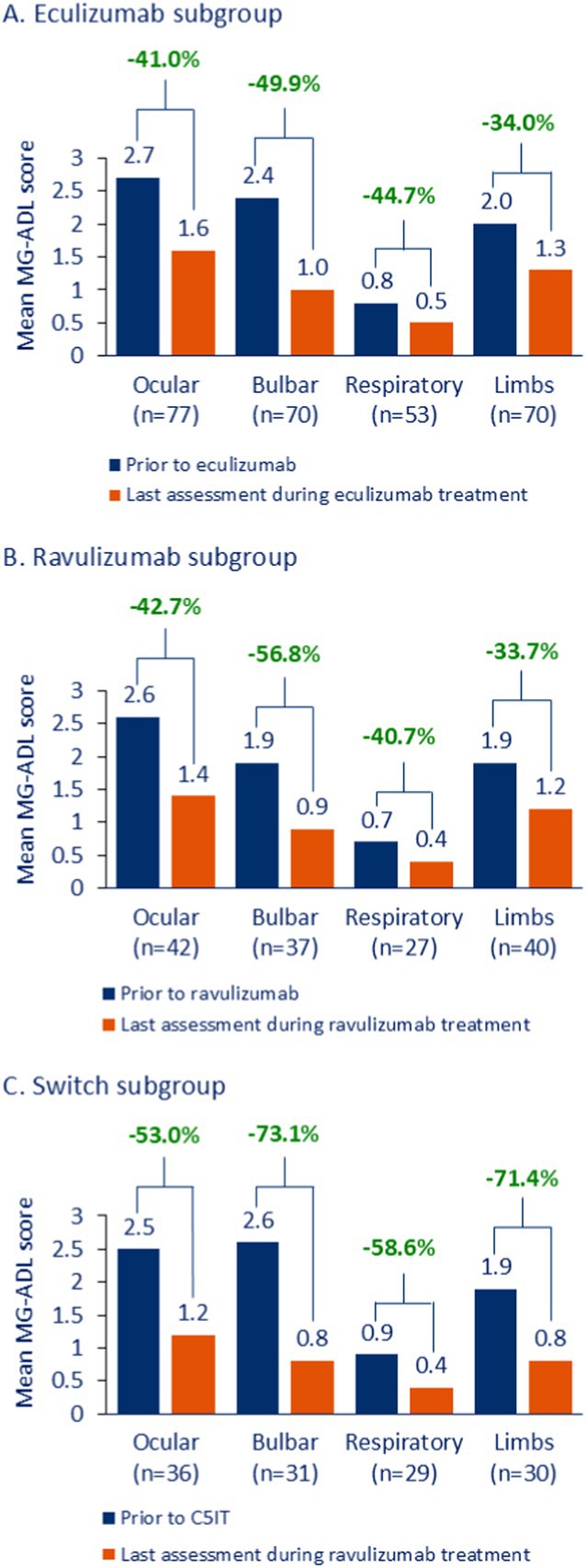
**FIGURE 1** Change in MG‐ADL subdomain scores after C5IT initiation.For each subdomain, *n* represents patients with nonmissing scores at both timepoints. Percent change in scores calculated for patients with nonzero scores prior to C5IT initiation. Max subdomain scores: 6 (ocular, limbs), 9 (bulbar), 3 (respiratory).

**Conclusion:** These results from clinical practice show the broad benefit of complement C5 inhibition with eculizumab and ravulizumab in improving ocular, bulbar, respiratory, and limb function as indicated by MG‐ADL subdomain score in patients with gMG.

**Disclosure:** VJ: consultant/advisor/principal site investigator (PI): Alexion, AZ Rare Disease, Accordant, argenx, Immunovant, Janssen. SPM: consultant: AbbVie, Alexion, argenx, Catalyst, Grifols, Kabafusion, Supernus, UCB. FS: honoraria/consultant/PI: Alexion, Alexis, Amgen, argenx, Biogen, Dianthus, Genpharm, Johnson&Johnson, Leadiant, Lexeo, MedPharm, Medison, Neopharm Israel Pharma, Novartis, Prilenia, Reata, Remegen, Roche, Sandoz, Sanofi, Takeda, UCB, Zai Lab. AJG: honoraria: Alexion, AZ Rare Disease, argenx, Janssen, UCB. JMW: consultant: Alexion, AZ Rare Disease, Biogen, BMS, Teva. LZ, AY, CL: hold stock/options in AZ. GC: consultant/advisor: AI Ther., Alexion, AZ Rare Disease, AMO Pharma, Antisense Ther., Applied Ther., AveXis, Avotres, Biogen, BMS/Celgene, Clene Nanomedicine, Clinical Trial Solutions, CSL Behring, Entelexo Biother., Genentech, Genzyme, GW Pharma, Hoya Corp, Horizon Pharma, Immunic, Immunosis, Karuna, Kezar Life Sciences, Klein Buendel, Linical, Mapi Pharma, Merck/Serono, Mitsubishi Tanabe, NHLBI (Protocol Review Committee), Novartis, Opko Biologics, Perception Neurosci., Protalix BioTher., Prothena Biosci., Reata, Regeneron, Roche, SAB Biother., Sanofi‐Aventis, Teva, UTSouthwestern, UPenn, Visioneering Technologies JA: speaker/consultant/PI: Alexion, argenx, UCB Pharma, Janssen, Amgen. RT: speaker/consultant/PI: Amylyx, Apellis, Alexion, Biogen, Cytokinetics, Mitsubishi Tanabe.

## EPR‐301

### Comparative efficacy of nipocalimab with other FcRn blocker therapies in generalized myasthenia gravis

#### S. Jacob ^1^; M. Hashim^2^; B. Hutton^3^; K. Gandhi^4^; R. Slowik^4^; C. Drudge^5^; A. El Khoury^4^; M. Ait‐Tihyaty^4^; M. Keng^2^; X. Lin^4^; S. Singh^5^; N. Gilhus^6^

##### ^1^University Hospitals Birmingham NHS Foundation Trust, Birmingham, UK; ^2^Johnson & Johnson, Beerse, Belgium; ^3^Ottawa Hospital Research Institute, Ottawa, Canada; ^4^Johnson & Johnson, Raritan, USA; ^5^EVERSANA, Burlington, Canada; ^6^Department of Clinical Medicine, University of Bergen, Bergen, Norway

**Background and aims:** Nipocalimab demonstrated improved and sustained efficacy on Myasthenia Gravis Activities of Daily Living (MG‐ADL) versus placebo (VIVACITY‐MG3, NCT04951622) in generalized myasthenia gravis (gMG). Without available head‐to‐head comparisons, we indirectly compared nipocalimab efficacy versus other FcRn blockers, efgartigimod and rozanolixizumab, as measured by change from baseline (CFB) on MG‐ADL.

**Methods:** Data were drawn from published registrational trials. Efgartigimod and rozanolixizumab have symptom‐based cyclic dosing and nipocalimab has biweekly dosing. Indirect treatment comparisons (ITCs) were conducted using a placebo‐anchored Bucher method to compare efficacy onset using 1‐week timepoint, and for consistency of disease control, comparisons were at 8‐weeks for efgartigimod (1‐cycle duration) and 14‐weeks for rozanolixizumab (final visit data reported). Unanchored population‐adjusted indirect comparisons (without placebo) were also conducted given cross‐trial differences in background standard‐of‐care. Differences < 0 favored nipocalimab for all comparisons.

**Results:** Bucher Mean CFB difference [95% confidence interval (CI)] was comparable at 1‐week versus efgartigimod [‐0.04(‐1.21,1.13)] and rozanolixizumab [7mg/kg = ‐0.11(‐0.98,0.76); 10mg/kg = ‐0.02(‐0.92,0.89)]. MG‐ADL CFB was numerically greater versus efgartigimod at 8‐weeks [‐1.02(‐2.51,0.47)] and significantly greater versus rozanolixizumab at 14‐weeks [7mg/kg = ‐1.30(‐2.40,‐0.20), *p* = 0.021; 10mg/kg = ‐1.40(‐2.47,‐0.34), *p* = 0.01]. Unanchored ITCs showed statistically significant mean differences (95%CI) favoring nipocalimab versus efgartigimod at 8‐weeks [‐2.50(‐4.02,‐0.98), *p* = 0.001] and versus rozanolixizumab at 14‐weeks [7mg/kg = ‐3.36(‐4.75,‐1.96), *p* < 0.001; 10mg/kg = ‐3.68(‐6.18,‐1.18), *p* = 0.004].

**Conclusion:** Nipocalimab demonstrated comparable rapid onset of action and indicated favorable consistency of disease control versus symptom‐based cyclic‐dosed FcRn blockers. Future studies should investigate long‐term sustained disease control, an essential consideration in managing a chronic condition like gMG.

**Disclosure:** S. Jacob has served as an international advisory board member or has been in the data monitoring committee for clinical trials for Alexion, Alnylam, Argenx, Johnson and Johnson, Immunovant, Merck, Novartis, Regeneron and UCB pharmaceuticals, is currently an expert panel member of Myasthenia Gravis consortium for Argenx pharmaceuticals and has received speaker fees from Argenx, Eisai, Terumo BCT and UCB pharmaceuticals. He is also a board member (trustee) of the UK myasthenia patient charity, Myaware. M. Hashim, K. Gandhi, R. Slowik, A. C. El Khoury, M. Ait‐Tihyaty, M. J. Keng, and X. Lin are employees of Johnson and Johnson and may hold stock/stock options of Johnson and Johnson. B. Hutton has previously received honoraria from EVERSANA and Evidinno Outcomes Research Inc. for provision of methodologic advice related to the conduct of systematic reviews, meta‐analyses and ITCs. C. Drudge and S. Singh are employees of EVERSANA. EVERSANA receives consultancy fees from pharmaceutical and device companies, including Johnson and Johnson. N. E. Gilhus has received consultative or speaker's honoraria from Johnson and Johnson, UCB, Argenx, Alexion, Merck, Dianthus, Amgen, Roche, Grifols, Immunovant, Huma, Denka, and Takeda.

## EPR‐302

### Cross‐reactivity of glutamate transporter EAA1 and truncated EAA2 isoforms revealed via structural neuroinformatics

#### T. Karagöl

##### Istanbul Faculty of Medicine, Istanbul University, Istanbul, Turkey

**Background and aims:** The glutamate transporter subfamily is essential for maintaining neurotransmitter homeostasis and its dysregulations have been linked to the etiologies of many conditions, including neurodegenerative diseases. Our previous study indicated an inhibitory potential of truncated isoforms on glutamate transporter bio‐assembly. However, EAA2 interactions showed twofold higher binding affinity than EAA1 interactions (approximately ‐30 and ‐16 kcal/mol, respectively). In this study, we aimed to further understand these dynamics by focusing on the cross‐reactions between EAA1 and isoforms of EAA2.

**Methods:** Utilizing a multi‐level computational approach, including gene‐centric isoform mapping and AlphaFold structural predictions, we identified several truncated isoforms of EAA2. Their complexes with canonical EAA1 were subjected to a wide range of multimer predictions/docking analyses, 50ns molecular dynamics simulations, and evolutionary coupling analyses. Accordingly, the root‐mean‐square‐fluctuation (RMSF), Poisson‐Boltzmann Surface‐Area (MMPBSA) and trajectory analyses were conducted.

**Results:** Our findings reveal that EAA2 truncated isoforms (UniProt‐IDs: A0A2R8Y642 and C9J9N5) form stable complexes with the canonical EAA1, with binding free energies of ‐23.16 and ‐22.83 kcal/mol after 50ns simulations (Figures 1 and 2). Subsequently, evolutionary conservation studies revealed these isoforms were not only structurally conserved but also demonstrates a significant evolutionary coupling with EAA1 (Figure 3). The conserved binding interfaces suggest a coevolutionary relationship that likely influences transporter oligomerization.

**Figure:**
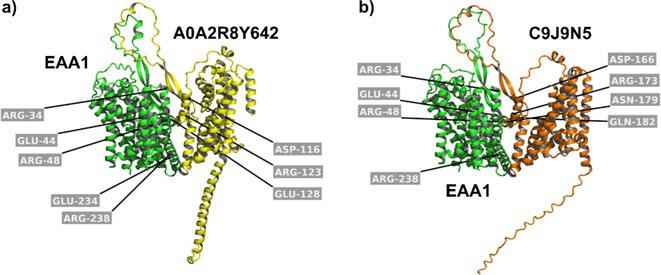
**FIGURE 1** Heterodimeric complexes of EAA1 canonical structure and EAA2 truncated isoforms a) A0A2R8Y642 (yellow) and b) C9J9N5 (orange). The interface surface is displayed with the eight highest energy‐contributing residues labeled.

**Figure:**
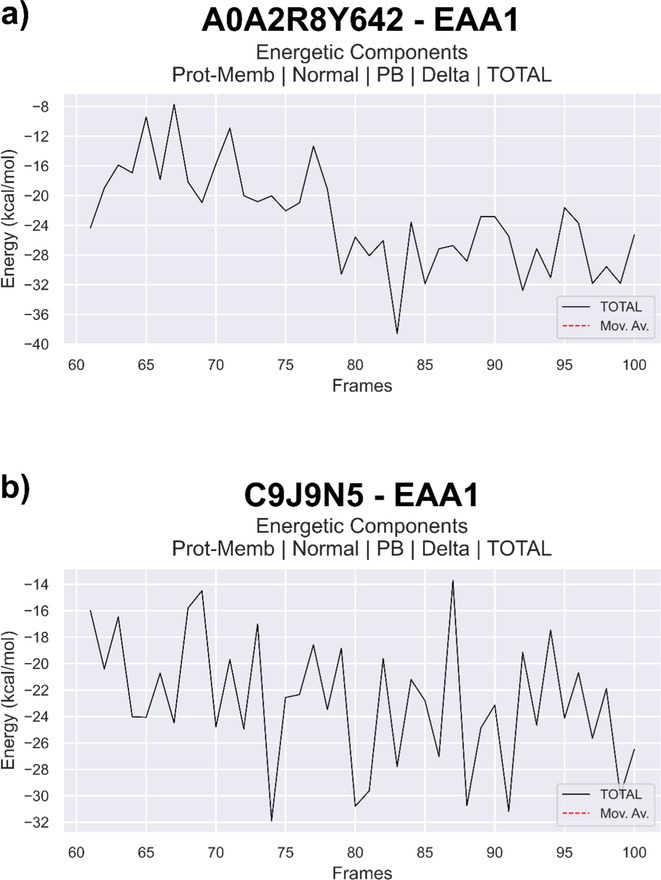
**FIGURE 2** MMPBSA binding energy calculations of complexes a) A0A2R8Y642‐EAA1 and b) C9J9N5‐EAA1 through 30‐50ns equilibrated molecular dynamics simulations.

**Figure:**
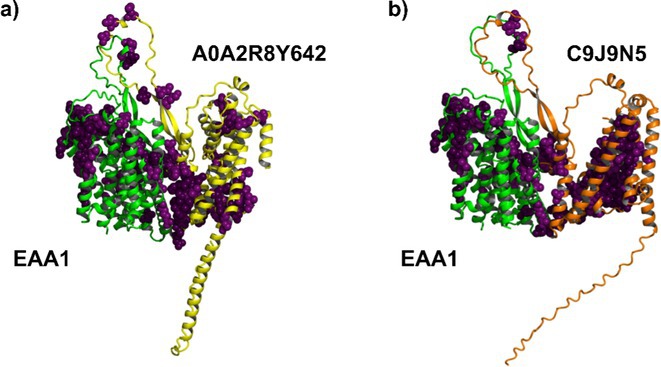
**FIGURE 3** Evolutionary coupling analysis of EAA1 canonical protein, EAA2 truncated isoforms a) A0A2R8Y642 (yellow) and b) C9J9N5 (orange). A strong co‐evolution (colored purple) of the residues contributing to the binding interfaces.

**Conclusion:** These findings not only deepen our understanding of glutamate transporter modulation but also identify promising therapeutic targets for neurological disorders. The study emphasizes the critical role of cross‐reactivity in transporter function and provides a foundation for future research into their therapeutic and diagnostic potential.

**Disclosure:** Nothing to disclose.

## Muscle and neuromuscular junction disorder 4

## EPR‐303

### Computational models for new patient stratification strategies of neuromuscular disorders: The CoMPaSS‐NMD project

#### A. Nuredini ^1^; M. Socha^2^; A. Suwalska^2^; S. Pini^1^; R. Corrias^3^; C. Dreyling^4^; A. Topf^5^; M. Vandroux^6^; J. Verdú Díaz^5^; A. Garzo Manzanares^7^; N. Albano^1^; A. Saak^4^; J. Laporte^6^; J. Diaz Manera^5^; M. Obach^7^; M. Scipioni^3^; M. Savarese^8^; F. Santorelli^9^; V. Straub^5^; B. Schoser^4^; J. Polańska^2^; R. Tupler^1^

##### ^1^Department of Biomedical, Metabolic and Neural Sciences, University of Modena and Reggio Emilia, Modena, Italy; ^2^Department of Data Science and Engineering, Silesian University of Technology, Gliwice, Poland; ^3^Fincons Group, Lugano, Switzerland; ^4^Friedrich‐Baur‐Institut, Department of Neurology, Ludwig Maximilian University (LMU), Munich, Germany; ^5^John Walton Muscular Dystrophy Research Centre, Translational and Clinical Research Institute, Newcastle Hospitals NHS Foundation Trust, Newcastle University, Newcastle Upon Tyne, UK; ^6^Institut de Génétique et de Biologie Moléculaire et Cellulaire (IGBMC), Université de Strasbourg, Illkirch, France; ^7^Tecnalia Research and Innovation, San Sebastian, Spain; ^8^Folkhälsan Research Center, Helsinki, Finland; ^9^Molecular Medicine for Neurodegenerative and Neuromuscular Diseases Unit, IRCCS Fondazione Stella Maris, Pisa, Italy

**Background and aims:** Hereditary Neuromuscular Diseases (HNMDs) are genetic disorders leading to progressive muscle wasting and weakness, affecting 111.9/100,000 individuals in the EU. Despite advances in diagnostics, over 60% of patients remain molecularly unsolved or misdiagnosed, hindering prognosis and treatment development. Artificial Intelligence (AI) offers transformative opportunities for data integration and precision diagnosis.

**Methods:** CoMPaSS‐NMD employs AI‐based tools for patient stratification through deep integration of clinical, genetic, histopathological, and imaging data. The study consists of: 1. Retrospective study: 3,900 genetic, 2,000 histopathological, 2,000 MRI existing data from European centers have been analyzed through unsupervised machine learning (ML) to develop stratification algorithms. 2. Prospective study: 500 undiagnosed HNMD patients undergoing standardized clinical, whole genome sequencing, histological, and MRI evaluations to generate new patient stratification means established on the ML‐based algorithms. 3. Creation of CoMPaSS‐NMD ATLAS as public repository of clinical, genetic, histopathological, and imaging data.

**Results:** Standard operating procedures have been developed to acquire robust datasets of uniform multimodal data. A new ranking tool for genetic variants has been proposed. Algorithms for histopathological and MRI analysis have been developed, addressing challenges such as data variability and identification of cluster‐specific signatures. The platform of the ATLAS is a novel tool to address the fragmentation of information leading to delays in diagnosis and incomplete knowledge of multifaceted diseases.

**Conclusion:** CoMPaSS‐NMD leverages AI to revolutionize the diagnosis of HNMDs, beyond the one gene‐one phenotype paradigm. This multidimensional approach wil boost diagnostic rates by 30%, optimize healthcare strategies, and significantly improve the lives of patients and caregivers.

**Disclosure:** This project has received funding from the European Union (Horizon 2020) under Grant Agreement n° 101080874.

## EPR‐304

### Exploring the lived experiences and needs of people with generalized myasthenia gravis: A mixed methods study

#### J. Raab^1^; S. Lehnerer^2^; P. Narayanaswami^3^; K. Wiltz^4^; J. Smith^4^; C. Schlemminger^5^; S. Tatlock^1^; G. Harty ^1^

##### ^1^The healthcare business of Merck KGaA, Darmstadt, Germany,^2^Department of Neurology with Experimental Neurology, Charité – Universitätsmedizin Berlin, Berlin, Germany,^3^Department of Neurology, Beth Israel Deaconess Medical Center/Harvard Medical School Boston, USA,^4^Patient Author,^5^Patient Author and DMG ‐ Deutsche Myasthenie Gesellschaft e. V., Bremen, Germany

**Background and aims:** Generalized myasthenia gravis (gMG) is an autoimmune neuromuscular disorder with profound everyday impacts. This study aimed to understand the lived experiences and needs of people with gMG (PWgMG).

**Methods:** PWgMG and specialist healthcare professionals (HCPs) were interviewed to inform development of a survey of PWgMG (themes: pre‐diagnosis, diagnosis, treatment, and living with gMG) in Germany, Italy, UK, and US. Interview data underwent thematic analysis. Statistics are descriptive.

**Results:** Fourteen PWgMG (71% female) and 10 neuromuscular specialists/neurologists were interviewed; 90 PWgMG were surveyed (64% female). PWgMG experienced several symptoms pre‐diagnosis (Figure 1) and most (89%) reported daily life impacts. Time to seeking medical advice and diagnosis varied by country, symptoms, and age. PWgMG typically visited 2–3 HCPs pre‐diagnosis; 25–65% were initially misdiagnosed, most commonly with mental health (40%) and neurological disorders (30%). Patients approached patient organizations (POs) for information at diagnosis more commonly in UK (68%) than Italy (26%), Germany (11%), or US (10%). PWgMG wanted PO resources to facilitate shared decision‐making with HCPs and increased gMG awareness among primary care physicians (PCPs). At treatment initiation, side‐effects and long‐term safety were the most common concerns of PWgMG (54%). Despite treatment, gMG continued to impact daily life (Figure 2). HCPs identified needs for improved HCP/PCP awareness of gMG and reported challenges in MG‐management related to insurance, comorbidities, speed‐of‐action and side effects of treatment, and family planning.

**Figure:**
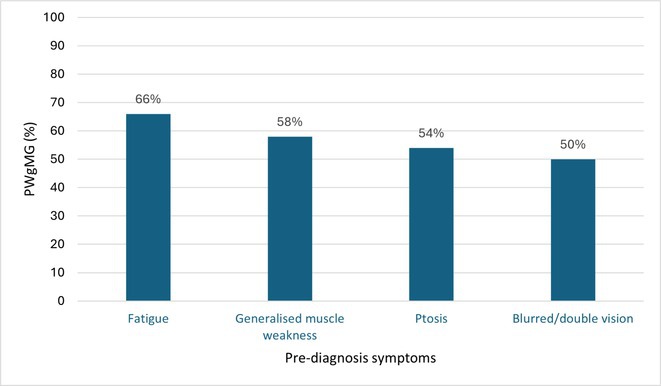
**FIGURE 1** Symptoms experienced by PWgMG before diagnosis. N = 90. Symptoms reported by ≥50% of surveyed PWgMG shown.

**Figure:**
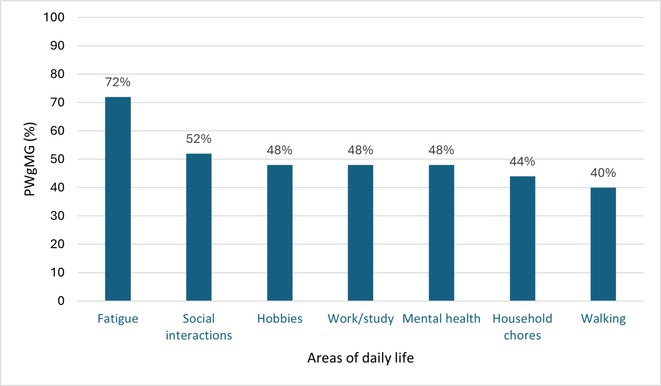
**FIGURE 2** Areas of daily life ‘always’ or ‘often’ negatively affected by gMG despite treatment. N = 90. Areas of daily life impacted in ≥40% of surveyed PWgMG shown.

**Conclusion:** PWgMG have concerns around treatment side‐effects/safety and experience limitations in daily activities. Resources to support shared decision‐making and HCP communication are needed.

**Disclosure:** Acknowledgment: This study was sponsored by the healthcare business of Merck KGaA, Darmstadt, Germany (CrossRef Funder ID: 10.13039/100009945). Medical writing support was provided by Simon Stones, PhD, ISMPP CMPP™, of Amica Scientific, and funded by the healthcare business of Merck KGaA, Darmstadt, Germany. Author disclosures: Sophi Tatlock and Jana Raab are employees of the healthcare business of Merck KGaA, Darmstadt, Germany. Sophie Lehnerer has received speaker or consultancy honoraria or financial research support (paid to their institution) from Alexion, argenx, Biogen, Hormosan, HUMA, Johnson & Johnson, Merck KGaA, UCB, and Roche. Pushpa Narayanaswami has received research support from AHRQ, Alexion/AstraZeneca, Momenta/Janssen/J&J, PCORI, and Ra/UCB; has been involved in advisory boards/consultations with Alexion/AstraZeneca, Amgen, argenx, CVS, Dianthus, GSK, ImmuneAbs, Janssen/J&J, Novartis, Merck KGaA, Roche and UCB; is the Data Monitoring Committee Chair for argenx and NMD pharma, has served as DMC Chair for Sanofi; and receives royalties from Springer Nature. Kathryn Wiltz has received financial research support from Huma and Merck on previous work. Janet Smith is a paid adviser to the healthcare business of Merck KGaA, Darmstadt, Germany, unrelated to this study. Claudia Schlemminger has no financial conflicts of interest to disclose. They are a patient representative for the Gemeinsamer Bundesausschus.

## EPR‐305

### Efficacy of Nipocalimab in open‐label extension in patients transitioned from placebo: Results from Vivacity‐MG3 trial

#### K. Claeys ^1^; M. Ait‐Tihyaty^2^; K. Gandhi^2^; I. Turkoz^3^; Z. Choudhry^4^; W. Noel^5^; C. Gary^6^; S. Ramchandren^3^; T. Vu^7^

##### ^1^Department of Neurology, University Hospitals Leuven, and Laboratory for Muscle Diseases and Neuropathies, KU Leuven, Leuven, Belgium; ^2^Johnson & Johnson, Raritan, USA; ^3^Johnson & Johnson, Titusville, USA; ^4^Johnson & Johnson, Horsham, USA; ^5^Johnson & Johnson, Beerse, Belgium; ^6^Johnson & Johnson, Issy‐les‐Moulineaux, France; ^7^University of South Florida, Morsani College of Medicine, Tampa, USA

**Background and aims:** In the 24‐week (W) double‐blind phase‐3 Vivacity‐MG3 study (NCT04951622), nipocalimab+standard‐of‐care (SOC) demonstrated statistically significant and clinically meaningful improvements versus placebo+SOC in patients with generalized myasthenia gravis (gMG). After completing the double‐blind phase, patients from placebo‐arm may receive nipocalimab+SOC in ongoing open‐label extension (OLE) to W24.

**Methods:** In OLE, 98 patients from placebo+SOC transitioned to nipocalimab+SOC. Data were collected up to OLE W24 (cutoff: 23‐August‐2024). Mean changes in MG‐ADL and QMG scores from OLE baseline were evaluated. Within‐group mean changes were examined using paired t‐test. Percentage of patients achieving Meaningful Clinical Improvement (MCI > = 2‐point within‐patient improvement versus baseline in MG‐ADL and sustained MCI (for > = 8W) and percentage‐of‐time spent in MCI were summarized.

**Results:** The mean (standard deviation [SD]) MG‐ADL and QMG scores at OLE baseline were 6.33(3.37) and 13.47(5.70), respectively. Improvements in MG‐ADL score were observed as early as OLE W2 in placebo+SOC patients transitioned to nipocalimab+SOC: mean (SD) change of −1.33(2.13), *n* = 87, *p* < 0.001, improving to −2.68(3.26), *n* = 59, *p* < 0.001 at OLE W24. At W24, 63.3% of patients achieved MCI, with 79.5% achieving it at any time during OLE phase; 51.1% of patients had sustained MCI. Similarly, as early as OLE W4, QMG improvement was observed with mean (SD) change of −2.65([3.95], *n* = 79, *p* < 0.001), continuing to −3.24([4.95], *n* = 58, *p* < 0.001) at OLE W24.

**Conclusion:** Placebo+SOC arm patients with gMG from Vivacity‐MG3 who transitioned to nipocalimab+SOC exhibited improvements in MG‐ADL as early as W2 after transition, with continued improvement through W24. This supports the potential of nipocalimab as an effective maintenance treatment option in this gMG population.

**Disclosure:** This work was funded by Johnson & Johnson. Kristl Claeys: Speaker/advisory board honoraria from Alexion, Alnylam, Amicus Therapeutics, argenx, Biogen, Ipsen, Johnson & Johnson Lupin, Pfizer, Roche, Sanofi‐Genzyme, UCB, and Research funding from CSL Behring, Roche, Vertex. Maria Ait‐Tihyaty, Kavita Gandhi, Ibrahim Turkoz, Zia Choudhry, Wim Noel, Charlotte Gary, and Sindhu Ramchandren: Employees of Johnson & Johnson, may hold stocks/stock options in Johnson & Johnson. Tuan Vu: MG related research or grant support from Alexion/AstraZeneca Rare Disease, Amgen, argenx, Cartesians, COUR, Dianthus, Johnson & Johnson, Immunovant, NMD Pharma, Regeneron, and UCB; consultant and/or speaker bureau for Alexion, argenx, CSL Behring, Johnson & Johnson, ImmunAbs, and Dianthus.

## EPR‐306

### Efficacy of Nipocalimab in adult patients with moderate to severe ocular manifestations of gMG in phase 3 VIVACITY‐MG3

#### K. G. Claeys ^1^; K. Gandhi^2^; M. Ait‐Tihyaty^2^; I. Turkoz^3^; S. Pease^2^; C. Gary^4^; Z. Choudhry^5^; S. Ramchandren^3^

##### ^1^Department of Neurology, University Hospitals Leuven, and Laboratory for Muscle Diseases and Neuropathies, KU Leuven, Leuven, Belgium; ^2^Johnson & Johnson, Raritan, USA; ^3^Johnson & Johnson, Titusville, USA; ^4^Johnson & Johnson, Issy‐les‐Moulineaux, France; ^5^Johnson & Johnson, Horsham, USA

**Background and aims:** In generalized myasthenia gravis (gMG), 15‐50% patients have ocular manifestations (ptosis, diplopia) limiting daily‐activities and impacting quality‐of‐life. Nipocalimab+standard‐of‐care (SOC, nipocalimab) demonstrated sustained efficacy versus placebo+SOC (placebo) in VIVACITY‐MG3 (NCT04951622). This analysis evaluated efficacy of nipocalimab versus placebo in the subgroup of patients with moderate‐severe ocular manifestations (MSOM).

**Methods:** In this post‐hoc analysis, MSOM was defined as baseline score of > = 2‐points on either diplopia or ptosis items of Myasthenia Gravis‐Activities of Daily Living (MG‐ADL) scale. Least‐squares (LS) mean changes from baseline (CFB) to week 24 (W24) on the MG‐ADL‐ocular domain (MG‐ADL‐ocular) and total scores (MG‐ADL‐total) were analyzed using repeated measures models. Chi‐square test statistics were used to evaluate the proportion of patients achieving meaningful within‐person improvement (MWPI) of > = 2‐points at 24‐weeks from baseline; logistic regression models were used to examine likelihood (OR) of achieving MWPI.

**Results:** At baseline, within MSOM subgroup, nipocalimab (*n* = 54/77) and placebo (*n* = 51/76) arms were comparable in mean age (52.5, 53.5 years), percentage of female patients (63%, 55%) and mean (Standard Deviation) MG‐ADL‐ocular (4.1[1.2]; 3.5[1.0]) and MG‐ADL‐total (10.1[2.8]; 9.4[1.9]) scores. At W24, the CFB LS mean differences (95%CI) for nipocalimab versus placebo were MG‐ADL‐ocular (‐1.6[‐2.6, ‐0.6]; ‐1.1[‐2.0, ‐0.2]; *p* = 0.024); and MG‐ADL‐total (‐4.6[‐5.4, ‐3.7]; ‐3.2[‐4.1, ‐2.3], *p* = 0.010), favoring nipocalimab. Greater proportion of patients achieved MWPI at W24 on MG‐ADL‐ocular (difference = 29.3%; OR = 3.6; *p* = 0.006) and MG‐ADL‐total (difference = 24.4%; OR = 3.5; *p* = 0.01), favoring nipocalimab.

**Conclusion:** Nipocalimab‐treated patients with gMG and MSOM showed superior improvements on the MG‐ADL‐ocular and MG‐ADL‐total scores versus placebo‐treated‐patients and were significantly more likely to achieve MWPI at W24.

**Disclosure:** This work was funded by Johnson & Johnson. Kristl Claeys: Speaker/advisory board honoraria from Alexion, Alnylam, Amicus Therapeutics, argenx, Biogen, Ipsen, Janssen Pharmaceuticals, Lupin, Pfizer, Roche, Sanofi‐Genzyme, UCB, and Research funding from CSL Behring, Roche, Vertex. Kavita Gandhi, Maria Ait‐Tihyaty, Ibrahim Turkoz, Sheryl Pease, Charlotte Gary, Zia Choudhry, and Sindhu Ramchandren: Employees of Johnson & Johnson, may hold stocks/stock options in Johnson & Johnson.

## EPR‐307

### Analysis of long‐term efficacy of nipocalimab in myasthenia gravis: Open‐label extension of the Vivacity‐MG3 trial

#### N. Silvestri^1^; M. Ait‐Tihyaty^2^; K. Gandhi^2^; I. Turkoz^3^; Z. Choudhry^4^; W. Noel ^5^; C. Gary^6^; S. Ramchandren^3^

##### ^1^University at Buffalo, Jacobs School of Medicine and Biomedical Sciences, Buffalo, New York, USA; ^2^Johnson & Johnson, Raritan, USA; ^3^Johnson & Johnson, Titusville, USA; ^4^Johnson & Johnson, Horsham, USA; ^5^Johnson & Johnson, Beerse, Belgium; ^6^Johnson & Johnson, Issy‐les‐Moulineaux, France

**Background and aims:** New targeted treatments are needed to provide sustained disease control of generalized myasthenia gravis (MG). Nipocalimab+standard‐of‐care (SOC) demonstrated sustained efficacy over 24‐weeks (W) in double‐blind phase of the Vivacity‐MG3 Phase‐3 trial (NCT04951622); ongoing open‐label extension (OLE) allows assessment of long‐term efficacy of nipocalimab.

**Methods:** 98 patients from the Vivacity‐MG3 nipocalimab+SOC arm transitioned from double‐blind to OLE phase. Mean changes from double‐blind baseline (CFB) in MG‐ADL and QMG scores were computed for nipocalimab+SOC‐treated patients at W24 and W48. Proportions of patients reaching Meaningful Clinical Improvement (MCI > = 2‐point within‐patient improvement versus baseline in MG‐ADL) were evaluated at post‐baseline visits and W48. Proportions of patients with sustained response (MCI maintained for > = 8W) were summarized. Similar analyses were conducted using QMG scores.

**Results:** The mean (standard deviation [SD]) MG‐ADL and QMG scores at double‐blind baseline (W0) were 9.51 (2.69) and 15.05 (4.80), respectively. The mean (SD) CFB in MG‐ADL score in nipocalimab+SOC‐treated patients was −4.46([3.59], *n* = 87, *p* < 0.001) at W24; improvement was maintained through W48 with a mean change of −5.56([3.72], *n* = 52, *p* < 0.001) in the OLE phase. 84.6% of the nipocalimab+SOC‐treated patients achieved MCI at W48 and 93.9% at any time post‐baseline. 77.6% of nipocalimab+SOC‐treated patients sustained MCI for > = 8W. Mean (SD) CFB in QMG score in nipocalimab+SOC‐treated patients was −4.21([4.87], *n* = 81, *p* < 0.001) at W24; improvement was maintained through W48 with mean (SD) change in QMG score of −4.73([4.45], *n* = 47, *p* < 0.001) in the OLE.

**Conclusion:** Nipocalimab+SOC demonstrated significant and clinically meaningful efficacy at W24 from double‐blind baseline. Disease control with nipocalimab+SOC was sustained up to 48‐weeks.

**Disclosure:** This work was funded by Johnson & Johnson. Nicholas J. Silvestri: Consultant/advisor for Alexion, Amgen, Annexon, argenx, Immunovant, Johnson & Johnson, and UCB. Speaker for Alexion, argenx, Takeda, and UCB. Maria Ait‐Tihyaty, Kavita Gandhi, Ibrahim Turkoz, Zia Choudhry, Wim Noel, Charlotte Gary, and Sindhu Ramchandren: Employees of Johnson & Johnson, may hold stocks/stock options in Johnson & Johnson.

## EPR‐308

### A real‐life experience with zilucoplan in AChR‐seropositive generalized myasthenia gravis

#### N. Rini; P. Luppino; P. Alonge; U. Quartetti; C. Messina; F. Brighina; V. Di Stefano

##### Department of Biomedicine, Neuroscience and Advanced Diagnostic, University of Palermo, Italy

**Background and aims:** Myasthenia gravis is a rare chronic autoimmune disease affecting the post‐synaptic membrane of the muscle junction characterized by debilitating, and potentially fatal, muscle weakness. Treatment options available for generalized myasthenia gravis (gMG) have grown in recent years with the introduction of new drugs, such as the complement factor C5 inhibitors. The objective of the Compassionate Use Program (GM0025IT) is to provide early access to Zilucoplan for gMG patients with a high unmet medical need and severe disease burden.

**Methods:** Zilucoplan was administered by daily subcutaneous self‐injection as for protocol. Efficacy was assessed by using the MG‐ADL scale and QMG. The patients' baseline therapy remained unchanged during the entire course of treatment.

**Results:** Fifteen patients (F/M 12/3, mean age 55 y) affected by AChR‐seropositive gMG received Zilucoplan. A reduction in MG‐ADL scores was observed after one week from the first injection compared to baseline (MG‐ADL 10.4  ± 3.5) with a mean change of – 1,67 points (ranging from ‐4 to 0). The main change at W4 was – 3,8 points and – 7,27 points at W24. Regarding QMG, a reduction of ‐3,86 points was achieved at W4 and – 7,28 points at W24, compared to baseline (QMG 16.5  ± 5.7).

**Figure:**
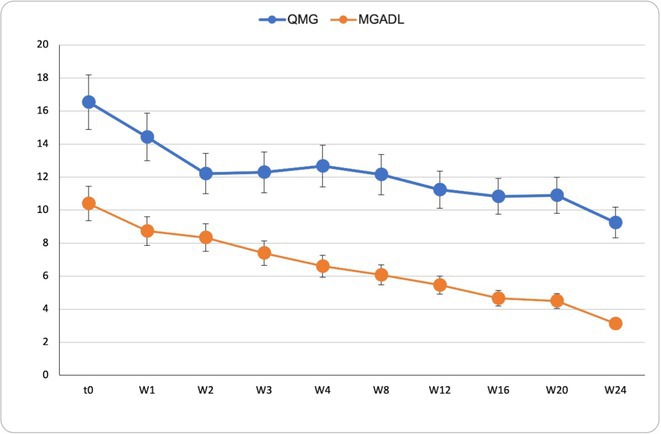
**FIGURE 1** Treatment effect on the MG‐ADL and QMG scales

**Figure:**
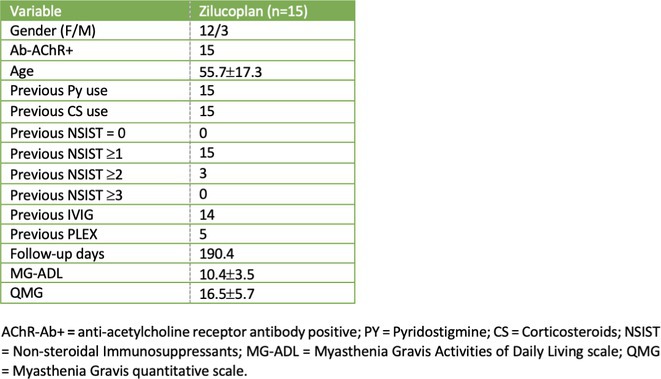
**TABLE 1** Patient demographics.

**Conclusion:** The decline in scores obtained testifies to the efficacy of treatment with Zilucoplan and the rapidity of its action onset. Also, Zilucoplan appears to be very easy to administer and has a favorable safety profile. Further data and longer observations are needed to confirm our data.

**Disclosure:** Nothing to disclose.

## EPR‐309

### A Bayesian ordinal transition model of Guillain‐Barré syndrome (GBS) disability progression with anti‐C1q treatment

#### P. Lin^1^; M. Rohde^2^; H. Kroon^1^; E. Steyerberg^3^; G. Morrison ^1^; K. Gorson^4^; Q. Mohammad^5^; Z. Islam^6^; K. Azad^7^; J. Navarro^8^; P. Collins^1^; F. Harrell^2^

##### ^1^Annexon Biosciences, Brisbane, USA; ^2^Department of Biostatistics, Vanderbilt University School of Medicine, Nashville, USA; ^3^Julius Center for Health Sciences and Primary Care, Utrecht, Netherlands; ^4^St. Elizabeth's Medical Center, Boston, USA; ^5^National Institute of Neurosciences & Hospital, Dhaka, Bangladesh; ^6^Gut–Brain Axis Laboratory, icddr,b, Dhaka, Bangladesh; ^7^Dhaka Medical College and Hospital, Dhaka, Bangladesh; ^8^José R. Reyes Memorial Medical Center, Manila, Philippines

**Background and aims:** Guillain‐Barré syndrome (GBS) is a complement‐mediated peripheral neuropathy. In a double‐blind, placebo‐controlled phase 3 study (NCT04701164), ANX005 30 mg/kg rapidly inhibited C1q, demonstrating early and sustained improvements. Although the GBS Disability Scale (GBS‐DS) tracks functional impairment, traditional analyses may overlook disability state transitions. We aimed to capture transitions in functional outcomes over time.

**Methods:** A Bayesian ordinal transition model with a first‐order Markov transition structure was fit to the longitudinal ordinal GBS‐DS data from the Phase 3 study (placebo, *n* = 81; 30 mg/kg, *n* = 79; 75 mg/kg, *n* = 81). Non‐informative priors were used for all parameters. Baseline prognostic factors were used as covariates. The effect of treatment was allowed to vary over time by treatment group using splines. Conditional quantities were computed using covariate median values.

**Results:** ANX005 increased the number of visits spent in a good health status (GBS‐DS 0 or 1) with a high probability (99.6%). Compared to placebo, ANX005 30 mg/kg increased the likelihood of transitioning to a better health state on the GBS‐DS rapidly, starting in the first three weeks. The cumulative treatment effect assessed by model‐based estimates of the probabilities of scores over time demonstrated a higher probability of having a good health status through 6 months of follow‐up at each visit compared to placebo (Figure).

**Figure:**
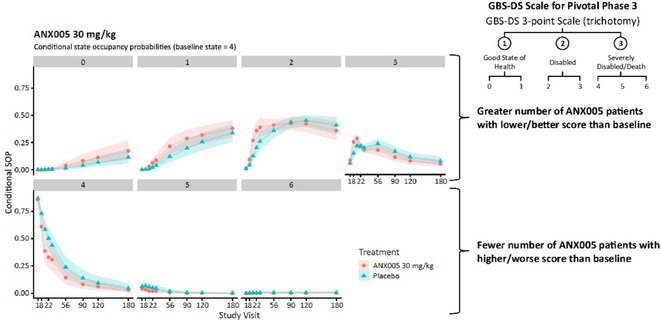
**FIGURE 1** Conditional State Occupancy Probabilities (30 mg/kg) Baseline State = 4.

**Conclusion:** ANX005 30 mg/kg demonstrated rapid and sustained improvement, highlighting its potential benefit in GBS. This Bayesian ordinal transition model effectively uses the raw data, thereby increasing power, and presents a longitudinal view of treatment effect on disease progression compared to traditional cross‐sectional methods.

**Disclosure:** The study was sponsored by Annexon Biosciences (Brisbane, CA, USA). PL: Employee and shareholder of Annexon Biosciences MR: Employee and shareholder of Annexon Biosciences HAK: Employee and shareholder of Annexon Biosciences ES: Consultancy/advisory role with Annexon Biosciences GM: Employee and shareholder of Annexon Biosciences KCG: Consultancy/advisory role with Annexon Biosciences, Argenx, Janssen, and Sanofi QDM: Consultancy/advisory role with Annexon Biosciences. ZI: Research funding from Fogarty International Center, National Institute of Neurological Disorders and Stroke of the National Institutes of Health, USA, and Annexon Biosciences. KAKA: No disclosures JN: Consultancy/advisory role with Annexon Biosciences PC: Employee and shareholder of Annexon Biosciences FEH: Consultancy/advisory role with Annexon Biosciences, Baylor Scott & White Research Institute, and Regeneron

## EPR‐310

### Long‐term outcomes of steroid dosing regimens and withdrawal in myasthenia gravis: A single‐center cohort study

#### Y. Huang; Y. Tan; J. Yan; Y. Guan

##### Department of Neurology, Peking Union Medical College and Chinese Academy of Medical Sciences, Peking Union Medical College Hospital, Beijing, China

**Background and aims:** Corticosteroids (steroids) are the first‐line immunotherapy for myasthenia gravis (MG). However, the optimal steroid dosing regimen and the effects of discontinuing immunotherapy remain unclear. This study aimed to investigate the impact of different steroid regimens and steroid withdrawal in MG with steroid monotherapy.

**Methods:** This is a cohort study based on a single‐center prospective registry, including patients who achieved sustained minimal manifestations or better status with steroid monotherapy. The primary outcome was relapse. Group‐based trajectory modeling (GBTM) identified distinct steroid regimens, and Cox proportional hazards models and propensity score matching (PSM) assessed the impact of regimens and steroid withdrawal.

**Results:** In 209 patients (median follow‐up 54.0 months), 113 (54.1%) experienced relapse, and 65 (31.1%) discontinued steroids. GBTM identified three regimens: “High Start, Fast Taper” (Regimen 1), “Low Start, Slow Taper” (Regimen 2), and “Moderate Start, Gradual Taper” (Regimen 3). Compared to Regimen 1, Regimen 2 (HR = 0.15, 95% CI 0.07‐0.32, *p* < 0.001) and Regimen 3 (HR = 0.28, 95% CI 0.15‐0.53, *p* < 0.001) had significantly lower relapse risks. In patients who withdrew steroids, the median time to relapse was 7.0 (3.0, 22.0) months. After PSM, the steroid withdrawal group had a significantly higher 1‐year relapse risk (HR = 1.58, 95% CI 1.03‐2.44, *p* = 0.039) compared to the low‐dose maintenance group.

**Conclusion:** High initial dose, rapid‐tapering regimen increases relapse risk, while steroid withdrawal also significantly raises relapse risk compared to low‐dose maintenance. Therefore, caution is advised when rapid tapering or discontinuing steroids in patients on steroid monotherapy.

**Disclosure:** Nothing to disclose.

## EPR‐311

### Evaluating the efficacy of nonviral gene delivery systems in DMD: A comprehensive meta‐analysis

#### Y. Hamdi ^3^; M. Farghaly^2^; N. Kasem^2^; H. Eltamawy^2^; F. Abdelbar^2^; H. Elshazly^2^; M. Elsayed^1^

##### ^1^MME Foundation; ^2^Newgiza University, Giza, Egypt; ^3^Badr University in Cairo, Egypt

**Background and aims:** Duchenne muscular dystrophy (DMD) is a progressive neuromuscular disorder caused by mutations in the dystrophin gene, resulting in a complete absence of the dystrophin protein. Nonviral gene delivery systems have emerged as promising alternatives to viral vectors, offering reduced immunogenicity and improved safety profiles.

**Methods:** A systematic search of PubMed, Embase, Cochrane Library, and ClinicalTrials.gov identified relevant randomized controlled trials (RCTs), non‐randomized studies, and preclinical research. Data were extracted and analyzed per PRISMA guidelines using R and Python. Risk of bias was assessed with the Cochrane Risk of Bias 2.0 and SYRCLE tools.

**Results:** The analysis included 20 studies with 1,325 participants and animal models. Nonviral systems, including liposomes, nanoparticles, and polymer‐based carriers, significantly increased dystrophin expression (mean increase: 18.7%, 95% CI: 12.4–24.9%, *p* < 0.001). Functional outcomes, evaluated through grip strength and locomotion tests, improved significantly (mean increase: 2.6 points, 95% CI: 1.8–3.4, *p* < 0.001). Safety analysis showed a lower incidence of immune‐related adverse events with nonviral systems (RR: 0.42, 95% CI: 0.29–0.59, *p* < 0.001) compared to viral vectors. Polymer‐based carriers achieved the highest dystrophin expression, while lipid‐based systems exhibited superior safety profiles.

**Conclusion:** Nonviral gene delivery systems demonstrate significant potential for dystrophin restoration, functional improvement, and enhanced safety in DMD therapy. Further research is needed to optimize delivery methods, dosing, and long‐term outcomes to facilitate clinical application.

**Disclosure:** Nothing to disclose.

